# Organocatalytic intramolecular (4 + 2) annulation of enals with ynamides: atroposelective synthesis of axially chiral 7-aryl indolines†

**DOI:** 10.1039/d3sc01880f

**Published:** 2023-05-05

**Authors:** Zhi-Xin Zhang, Li-Gao Liu, Yi-Xi Liu, Jian Lin, Xin Lu, Long-Wu Ye, Bo Zhou

**Affiliations:** a State Key Laboratory of Physical Chemistry of Solid Surfaces, Key Laboratory of Chemical Biology of Fujian Province, College of Chemistry and Chemical Engineering, Xiamen University Xiamen 361005 China zhoubo@xmu.edu.cn xinlu@xmu.edu.cn; b State Key Laboratory of Organometallic Chemistry, Shanghai Institute of Organic Chemistry, Chinese Academy of Sciences Shanghai 200032 China; c State Key Laboratory of Elemento-Organic Chemistry, Nankai University Tianjin 300071 China

## Abstract

Catalytic enantioselective transformation of alkynes has become a powerful tool for the synthesis of axially chiral molecules. Most of these atroposelective reactions of alkynes rely on transition-metal catalysis, and the organocatalytic approaches are largely limited to special alkynes which act as the precursors of Michael acceptors. Herein, we disclose an organocatalytic atroposelective intramolecular (4 + 2) annulation of enals with ynamides. This method allows the efficient and highly atom-economical preparation of various axially chiral 7-aryl indolines in generally moderate to good yields with good to excellent enantioselectivities. Computational studies were carried out to elucidate the origins of regioselectivity and enantioselectivity. Furthermore, a chiral phosphine ligand derived from the synthesized axially chiral 7-aryl indoline was proven to be potentially applicable to asymmetric catalysis.

## Abstract 2

An organocatalytic intramolecular (4 + 2) annulation of enals with ynamides is disclosed, providing a convenient way to axially chiral indolines. The synthesized axially chiral skeleton was proved to be potentially applicable as a chiral ligand.

## Graphical abstract

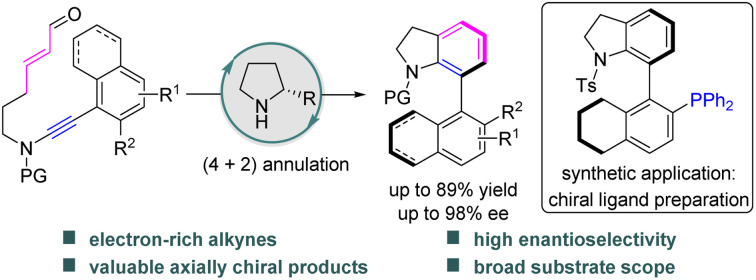

## Introduction

Axially chiral biaryls, one of the prominent structural motifs in organic synthesis, ubiquitously exist in natural products, bioactive compounds, materials, privileged chiral ligands and organocatalysts.^1^ Numerous strategies have been developed to access axially chiral molecules.^2^ Among the wide variety of axially chiral biaryls, axially chiral indoles and their derivatives are particularly important frameworks with wide practical application.^3^ However, the enantioselective preparation of indole-derived atropisomers is still challenging because of their lower rotational barriers compared with common binaphthyl skeletons.^4^ For instance, the methodologies directed towards axially chiral indolines are especially scanty, while they appear frequently in natural products, such as diazonamide A and B.^5^ To meet the increasing demand of diverse axially chiral biaryl skeletons in chiral ligands, catalysts and biologically important molecules, more efficient catalytic methods, especially organocatalytic approaches, for the construction of axially chiral indole derivatives become imperative.

**Scheme 1:**
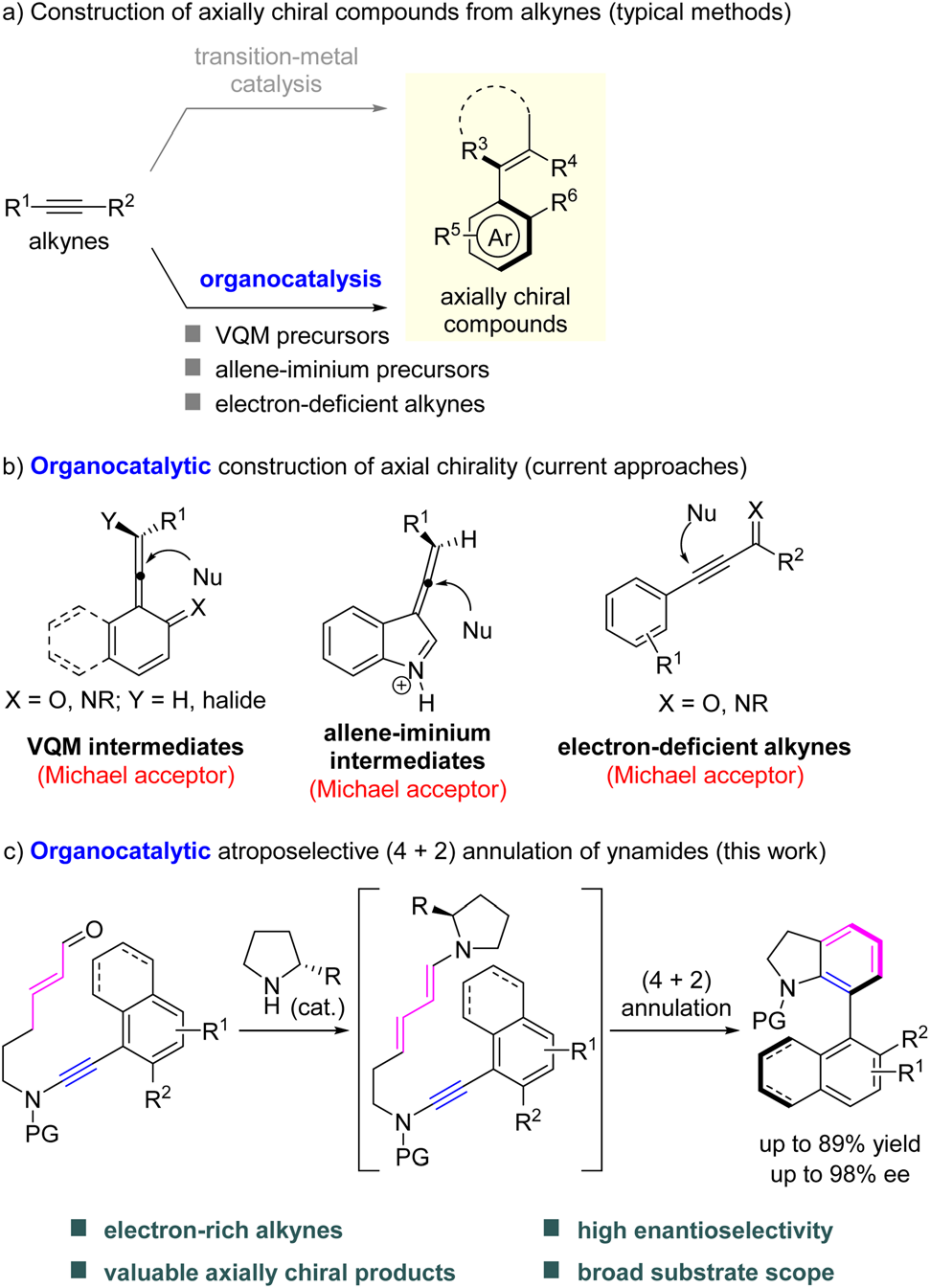
Construction of axially chiral compounds from alkynes.

Alkynes are among the fundamental building blocks in organic synthesis. The catalytic enantioselective transformations of alkynes have gained considerable interest over the past decades, for the assembly of axially chiral compounds (Scheme 1a).^6^ Elegant transition-metal catalyzed strategies have been established for the preparation of optically pure arenes and alkenes, such as (2 + 2 + 2) cycloaddition,^7^ intramolecular cyclization,^8^ Diels–Alder reaction,^9^ alkyne insertion^10^ and others.^11^ In contrast to transition-metal catalysis, the related organocatalytic atroposelective reactions of alkynes were developed by using vinylidene *ortho*-quinone methides (VQMs) and their analogues,^12^ allene–iminium intermediates^13^ and electron-deficient alkynes,^14^ although some intrinsic requirements are associated.^15^ For example, naphthol type of substrates are typically required for the VQM strategy, alkynal indoles are needed for the generation of allene–iminium intermediates and carbonyl group substituted alkynes are used for other amine or *N*-heterocyclic carbene (NHC) catalyzed reactions. It should be pointed out that alkynes were used as the precursors of Michael acceptors in the existing organocatalytic pathways (Scheme 1b). To overcome the structural limitations originating from Michael acceptors, a new metal-free atroposelective methodology based on alkynes is highly desirable.

To diversify the enantioselective transformations of alkynes, our group has been developing ynamide chemistry^16^ as a powerful tool to achieve chiral heterocycles under the promotion of transition-metal^17^ and Brønsted acid catalysts.^18,19^ Despite these achievements, the majority of them exhibited central chirality. Recently, a chiral phosphoric acid (CPA) catalyzed ynamide cyclization was discovered to generate C–N axially chiral indoles, representing the only organocatalytic example for the construction of axial chirality from ynamides.^19^ However, this kind of transformation remains elusive, which is probably due to the high reactivity of ynamides (such as hydration and dimerization of substrates under acidic conditions). Considering the special requirements for acid-sensitive substrates, it would be attractive if a transition-metal- and acid-free protocol could be established to achieve the atroposelective transformation of ynamides. Inspired by the above results and by our recent study on chiral secondary amine catalyzed enantioselective Conia-ene-type carbocyclization of ynamides,^20^ we envisioned that ynamides preinstalled with a sterically demanding group might react with enals through atroposelective dienamine catalysis^21^ for the assembly of axially chiral biaryls. Herein, we report the realization of such an organocatalytic atroposelective intramolecular (4 + 2) annulation of enals with ynamides, leading to the practical and atom-economical synthesis of axially chiral 7-aryl indolines in generally moderate to good yields with good to excellent enantioselectivities (Scheme 1c). Preliminary studies demonstrated the potential utility of the synthesized axially chiral 7-aryl indoline derivatives as chiral phosphine ligands.

## Results and discussion

To examine the feasibility of the proposed strategy, enal-tethered naphthyl ynamide 1a was chosen as the model substrate, and selected results are listed in Table 1. The proof-of-concept experiment was performed with a typically prevalent diarylprolinol silyl ether catalyst 3a at 60 °C, which delivered the corresponding 7-naphthyl indoline 2a in 71% yield with 34% ee (entry 1). After systematic evaluation of chiral amine catalysts, the less bulky diaryl derivatives 3b–d were found to be effective catalysts (entries 2–4), and 86% ee was obtained in the presence of 3d (entry 4). However, proline (3e) and chiral imidazolidine (3f) catalysts failed to catalyze the reaction (entries 5 and 6). A survey of different solvents (entries 7–12) indicated that aromatic solvents are superb for this transformation, and 1,2-dichlorobenzene (DCB) gave the best enantioselectivity (entry 12). On further optimization of reaction conditions by lowering the reaction temperature (entries 13 and 14), we determined the following optimal conditions to survey the substrate generality: enal-tethered naphthyl ynamide 1a (0.05 mmol) was treated with chiral secondary amine catalyst 3d (0.01 mmol) in DCB (1 mL) at 50 °C for 18 h, and the desired product 2a was obtained in 62% yield with 93% ee (entry 13).

**Table 1:** Optimization of reaction conditions^a^

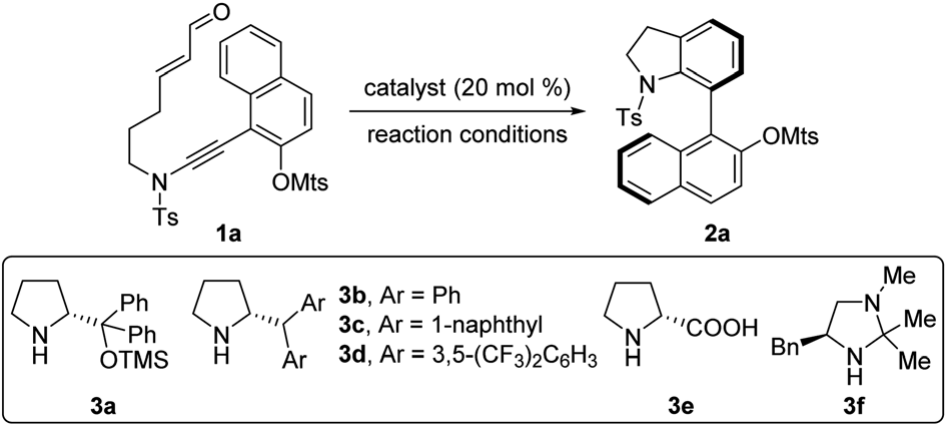				
Entry	Catalyst	Reaction conditions	Yield^b^ (%)	ee^c^ (%)
1	3a	DCE, 60 °C, 20 h	71 (<5)	34
2	3b	DCE, 60 °C, 48 h	65 (<5)	40
3	3c	DCE, 60 °C, 48 h	49 (<5)	51
4	3d	DCE, 60 °C, 12 h	65 (<5)	86
5	3e	DCE, 60 °C, 48 h	<5 (95)	—
6	3f	DCE, 60 °C, 48 h	<5 (95)	—
7	3d	MeCN, 60 °C, 12 h	70 (<5)	85
8	3d	Toluene, 60 °C, 24 h	60 (<5)	90
9	3d	PhF, 60 °C, 36 h	63 (<5)	90
10	3d	PhCI, 60 °C, 12 h	68 (<5)	91
11	3d	PhCF_3_, 60 °C, 12 h	62 (<5)	90
12	3d	DCB, 60 °C, 12 h	64 (<5)	92
**13**	3d	**DCB, 50 °C, 18 h**	**62 (<5)**	**93**
14	3d	DCB, 40 °C, 72 h	52 (9)	93

a Reaction conditions: 1a (0.05 mmol), catalyst (0.01 mmol), solvent (1 mL), 40 °C to 60 °C, 12–72 h, in vials.

b Measured by ^1^H NMR using diethyl phthalate as the internal reference. Recovered starting material given within parentheses.

c Determined by HPLC analysis. Mts = 2-mesitylenesulfonyl, DCB = 1,2-dichlorobenzene.

**Fig. 1:**
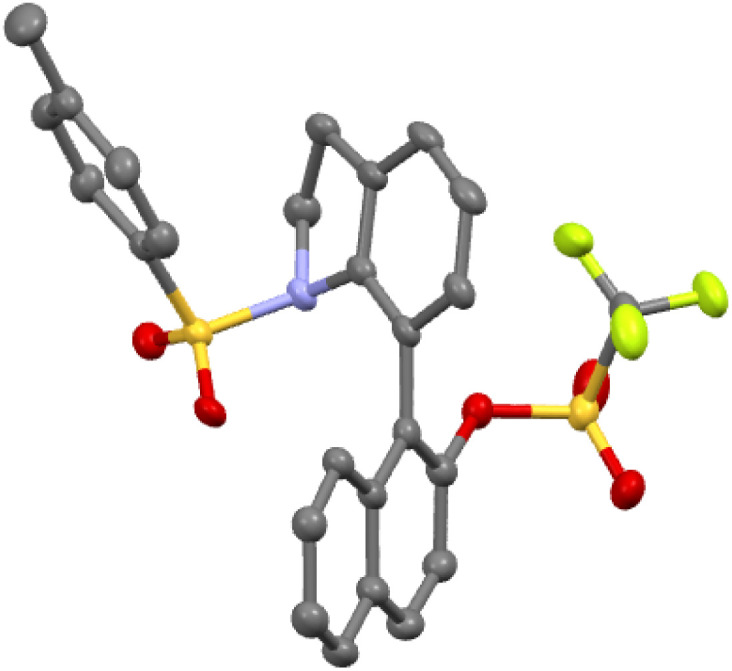
X-ray diffraction structure of compound 2o.

With the optimized reaction conditions in hand (Table 1, entry 13), the scope of this organocatalytic asymmetric (4 + 2) annulation was subsequently investigated. As depicted in Table 2, this atroposelective annulation proceeded smoothly with a range of ynamides with different *N*-protecting groups, delivering the corresponding 7-naphthyl indolines 2a–f in 45–80% yields with 88–94% ees. Owing to the mild reaction conditions, ynamides containing different sulfonyloxy groups at the 2-position of naphthalene ring were competent for this transformation (2g–n). It should be mentioned that the replacement of sulfonyloxy groups with other smaller substituents, such as alkoxy groups and halogens, resulted in low enantioselectivities (see the ESI† for details), probably due to their relatively lower rotational barriers. Delightfully, substrate 1o equipped with a reactive OTf group successfully produced the target product 2o in moderate yield with excellent enantioselectivity, which showed remarkable conformational stability of the C–C stereogenic axis. The easily convertible OTf group provides lots of opportunities for further applications. Various enal-tethered ynamides with both electron-donating and -withdrawing substituents at the 3-, 6- and 7-positions of naphthalene ring were all compatible, leading to the formation of the desired products 2p–y in 55–89% yields with excellent enantioselectivities. In addition to the naphthyl ynamides, substrate 1z bearing the 5,6,7,8-tetrahydronaphthyl group reacted efficiently, furnishing the desired product 2z in 42% yield with 97% ee. The absolute configuration of 2o was unambiguously confirmed by X-ray diffraction (Fig. 1).^22^

**Table 2:** Reaction scope for the (4 + 2) annulation of ynamides 1^a^

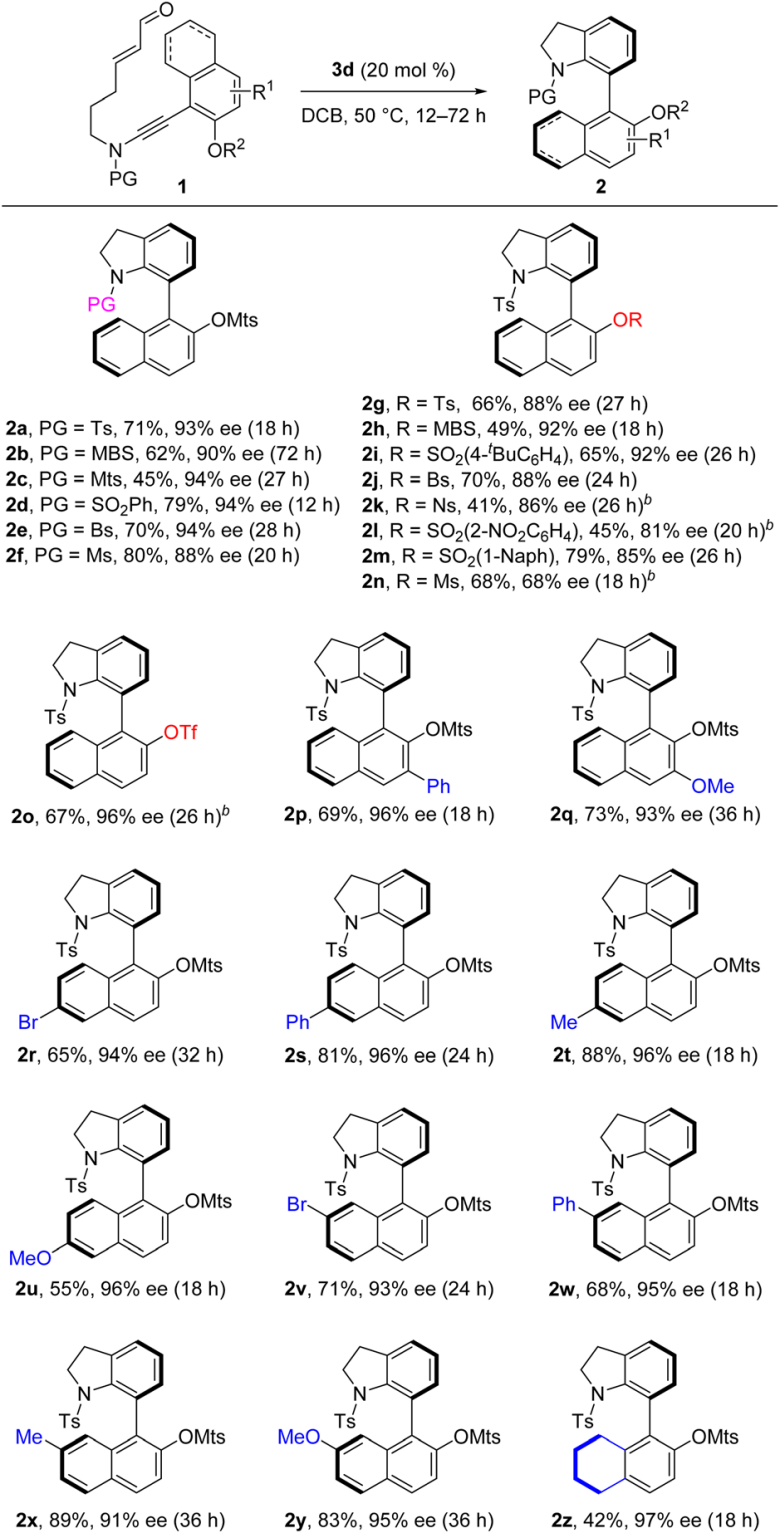

a Reaction conditions: 1 (0.1 mmol), 3d (0.02 mmol), DCB (2 mL), 50 °C, 12–72 h, in vials. Yields are those of isolated products; the ee values are determined by HPLC analysis.

b Reaction was carried out at 40 °C. PG = protecting group, MBS = 4-methoxybenzene sulfonyl, Bs = 4-bromo-benzene sulfonyl, Ns = 4-nitrobenzene sulfonyl, Ms = methanesulfonyl, Tf = trifluoromethanesulfonyl.

Considering the important synthetic manipulations of aryl trifluoromethanesulfonates, the substrate scope was further explored with regard to OTf-substituted ynamides 1 (Table 3). The switch of *N*-protecting groups from Ts to (4-*tert*-butyl) benzene sulfonyl and Ns group led to the formation of the desired 7-naphthyl indolines 2aa and 2ab in moderate yields with excellent enantioselectivities. It is notable that 8-position substituted naphthyl substrates reacted well to generate 2ac and 2ad in 65–69% yields albeit with a significantly reduced enantiocontrol in the latter case. Our attempts to extend the reaction to phenanthryl ynamide 1ae and 5,6,7,8-tetrahydronaphthyl ynamide 1af were fruitful, and the corresponding cyclization products could be isolated in 53–73% yields with 90−98% ees (2ae and 2af). Therefore, this protocol provides an efficient pathway for the generation of divergent axially chiral biaryls.

**Table 3:** Further scope study of OTf-substituted ynamides 1^a^

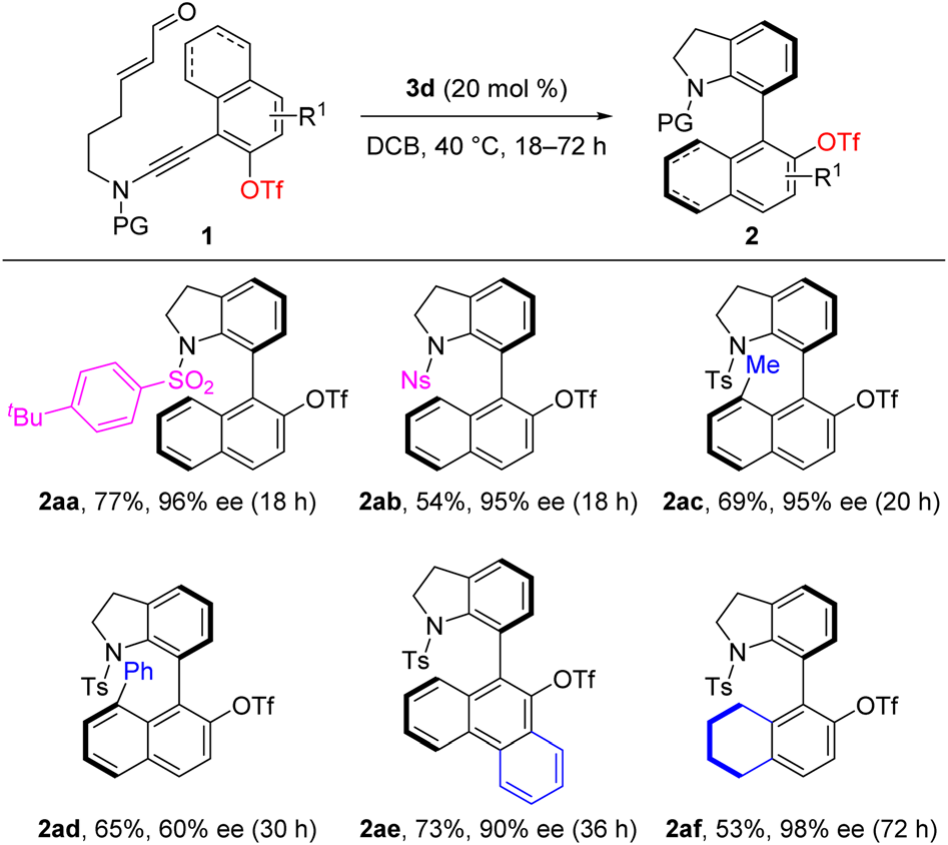

a Reaction conditions: 1 (0.1 mmol), 3d (0.02 mmol), DCB (2 mL), 40 °C, 18–72 h, in vials. Yields are those of isolated products; the ee values are determined by HPLC analysis.

**Scheme 2:**
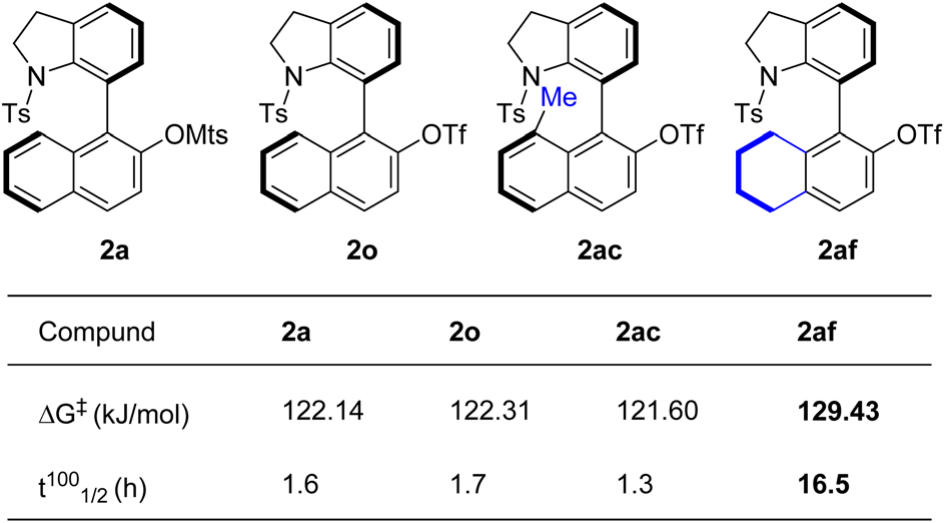
Racemization experiments.

The configurational stability of axially chiral 7-aryl indolines is one of the key factors for their utility. To measure the rotational barriers, racemization experiments were performed with selected representative examples in toluene at 100 °C (Scheme 2).^23^ Comparison of the rotational barriers of 2a (122.14 kJ mol^−1^), 2o (122.31 kJ mol^−1^) and 2ac (121.60 kJ mol^−1^) indicated that the installation of a methyl group on the 8-position of the naphthalene ring (2ac) does not have a measurable effect on the configurational stability. Importantly, 5,6,7,8-tetrahydronaphthyl product 2af demonstrated a higher rotational barrier (129.43 kJ mol^−1^) and much longer half-life at 100 °C (16.5 h). This phenomenon could be attributed to the greater steric hindrance of 5,6,7,8-tetrahydronaphthyl than the naphthyl group, which resulted in a stronger interaction with the Ts group.

**Scheme 3:**
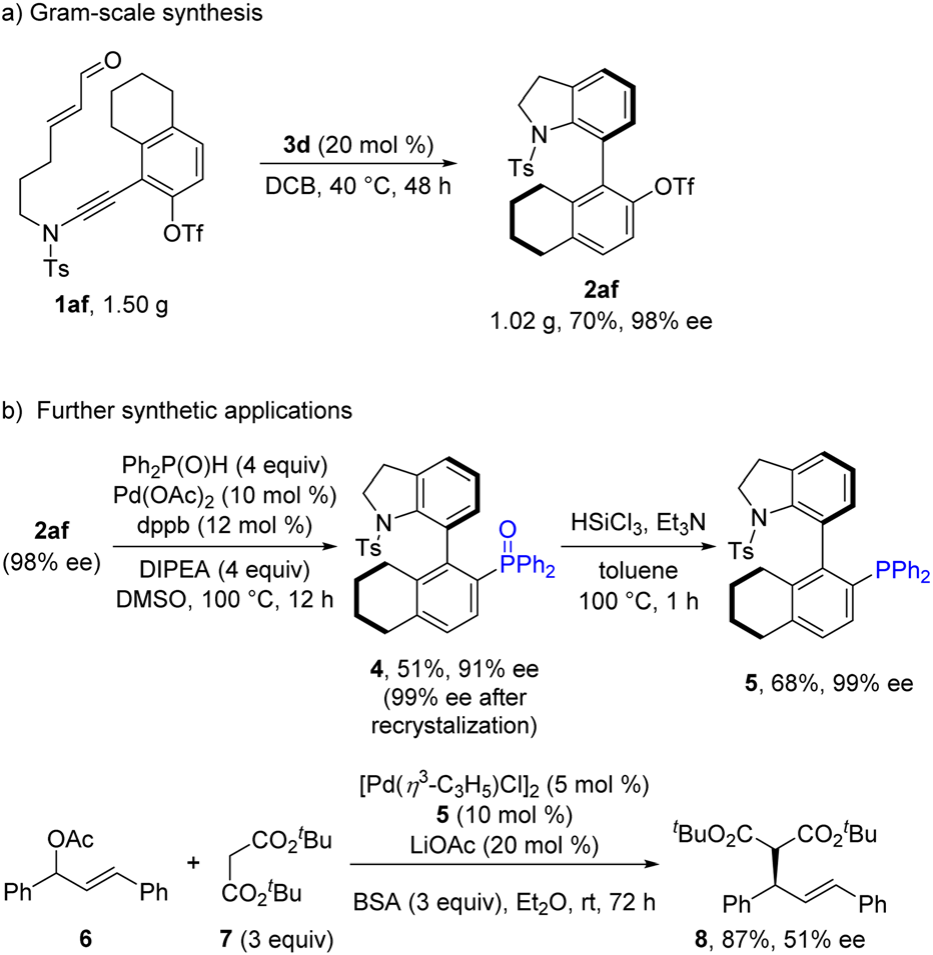
Synthetic utility study.

To explore the synthetic utility of this strategy, a gram-scale reaction was first carried out, which provided the (4 + 2) annulation product 2af in 70% yield with 98% ee (Scheme 3a). Furthermore, a chiral phosphine ligand based on the synthesized axially chiral skeleton was successfully prepared to prove the practicability of this method. The OTf-substituted 7-aryl indoline 2af could undergo palladium-catalyzed C–P coupling to give chiral phosphine oxide 4 in moderate yield with 91% ee. Subsequent recrystallization and reduction furnished the desired phosphine 5 with 99% ee.^4*g*^ Notably, compound 5 was used as a chiral ligand in the palladium-catalyzed enantioselective allyl substitution reaction of 1,3-diphenylallyl acetate 6 and di-*tert*-butyl malonate 7, affording the target product 8 in 87% yield with 51% ee (Scheme 3b). Although the enantioselectivity needs to be further improved, the constructed axially chiral 7-aryl indoline scaffold is promising for the development of a new type of chiral ligand.

**Scheme 4:**
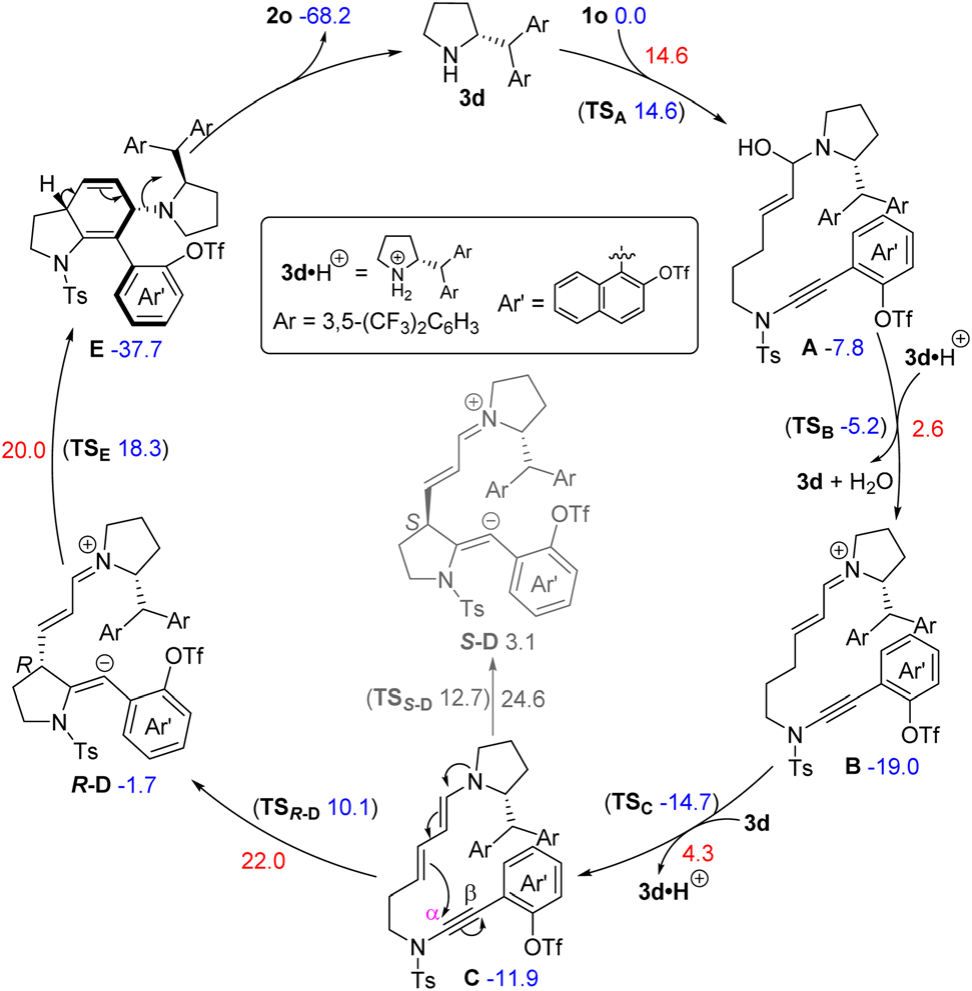
Plausible reaction mechanism. The relative free energies are given in kcal mol^−1^.

To gain a deeper understanding of the reaction mechanism, density functional theory (DFT) calculations were performed with organocatalyst 3d and ynamide 1o as the model (see the ESI† for details). The DFT-computed free energy profile of the operative catalytic cycle is shown in Scheme 4. Initially, the amine–aldehyde condensation between chiral amine catalyst 3d and enal-tethered ynamide 1o occurs *via* amino alcohol intermediate A, which affords the iminium-ion intermediate B. Subsequent iminium–enamine isomerization takes place to deliver dienamine intermediate C, followed by intramolecular nucleophilic addition of the dienamine group onto the α position of ynamide to form the major vinyl anion intermediate *R*-D^20,24^ with a free energy barrier of 22.0 kcal mol^−1^, which is considered as the rate-determining step.^20^ Importantly, detailed calculations indicate that the enantioselectivity of this cyclization step (from intermediate C to intermediate D) could be well controlled, and intermediate *R*-D is kinetically and thermodynamically more favorable than intermediate *S*-D.^21*f*^ Further cyclization of intermediate *R*-D leads to the axially chiral intermediate E. Finally, aromatization of E produces axially chiral 7-aryl indoline 2o and regenerates catalyst 3d. In addition, the attempt to locate a transition state for the formation of intermediate E from C directly through a concerted cycloaddition was unsuccessful, revealing a stepwise (4 + 2) annulation mechanism.

**Scheme 5:**
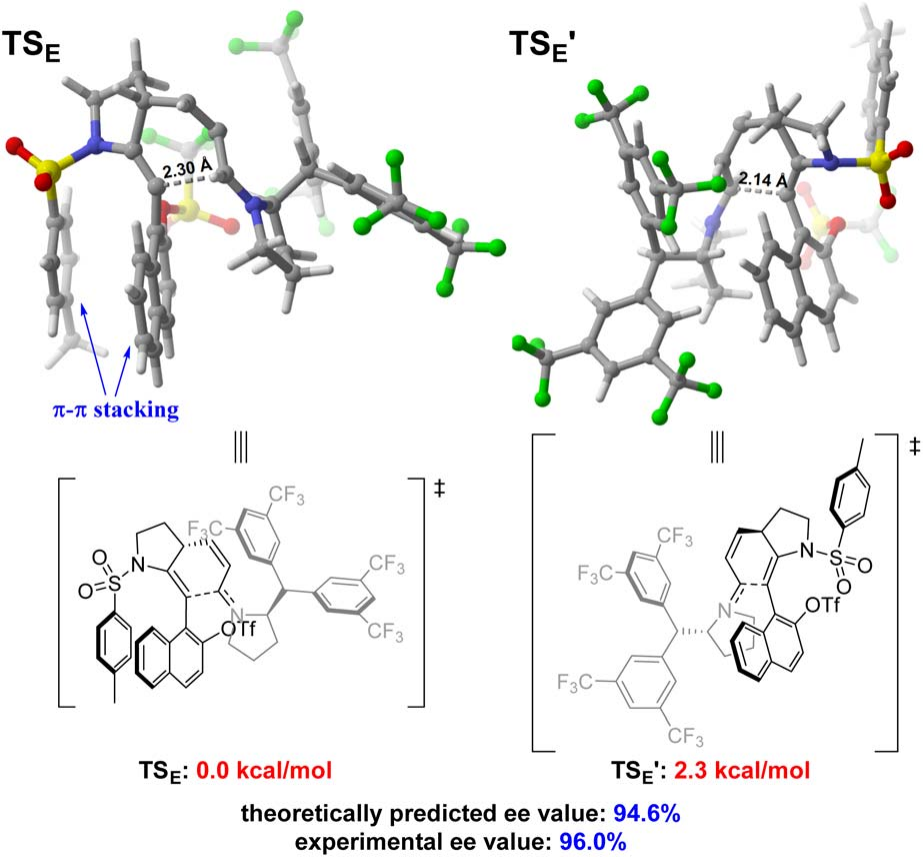
Optimized structures and relative free energies of the enantiomeric transition states.

Further theoretical calculations have been conducted to explain the origin of enantioselectivity in the enantio-determining cyclization step (from intermediate *R*-D to intermediate E). The optimized structures and relative free energies of the enantiomeric transition states are shown in Scheme 5. The free energy of transition state TS_E_ (leading to the major enantiomer) is 2.3 kcal mol^−1^ lower than TS_E′_ (leading to the minor enantiomer) and the theoretically predicted enantioselectivity matches well with the experimental ee value (94.6% *versus* 96%). Inspection of the structures of transition states shows that TS_E′_ has a shorter C⋯C distance than TS_E_ (2.14 Å *versus* 2.30 Å), suggesting that TS_E′_ has stronger steric repulsion and lower stability. The π–π stacking effect between the naphthyl and Ts group further stabilizes TS_E_. Thus, the observed enantioselectivity originates from steric effects and π–π stacking effect.

## Conclusions

In summary, an organocatalytic intramolecular (4 + 2) annulation of enals with ynamides has been developed, which provides a convenient way to prepare a range of axially chiral 7-aryl indolines with generally good to excellent enantioselectivities. The reaction represents the first atroposelective reaction of ynamides through chiral secondary amine catalysis. The mild reaction conditions enabled good functional group tolerance and wide substrate scope. Notably, the synthesized axially chiral 7-aryl indoline skeleton was proven to be potentially useful as a chiral phosphine ligand. Finally, theoretical calculations were carried out to understand the origins of regioselectivity and enantioselectivity. Efforts on developing organocatalytic intermolecular enantioselective reactions based on other heteroatom-substituted alkynes are ongoing in our laboratory.

## Data availability

Data for the crystal structure reported in this paper have been deposited at the Cambridge Crystallographic Data Centre (CCDC) under the deposition number CCDC 2234664 (2o). All other data supporting the findings of this study, including experimental procedures and compound characterization, are available within the paper and its ESI files,† or from the corresponding authors on request.

## Author contributions

Z.-X. Z., Y.-X. L. and J. L. performed experiments. L.-G. L. and X. L. performed DFT calculations. X. L., L.-W. Y. and B. Z. revised the paper. B. Z. conceived and directed the project and wrote the paper. All authors discussed the results and commented on the manuscript.

## Conflicts of interest

There are no conflicts to declare.

## Supplementary Material

SC-014-D3SC01880F-s001

SC-014-D3SC01880F-s002
