# Supplementary material for: Organocatalytic intramolecular (4 + 2) annulation of enals with ynamides: atroposelective synthesis of axially chiral 7-aryl indolines
Source: Chem Sci. 2023 May 5;14(22):5918–24. doi: 10.1039/d3sc01880f (PMC10246658; doi:10.1039/d3sc01880f)

Electronic Supplementary Material (ESI) for Chemical Science.
This journal is © The Royal Society of Chemistry 2023

Supporting Information

Organocatalytic intramolecular (4 + 2) annulation of enals with ynamides: atroposelective synthesis of axially chiral 7-aryl indolines

Zhi-Xin Zhang,‡[a] Li-Gao Liu,‡[a] Yi-Xi Liu,[a] Jian Lin,[a] Xin Lu,*,[a] Long-Wu Ye,[a,b,c] and Bo Zhou*,[a]

[a]State Key Laboratory of Physical Chemistry of Solid Surfaces, Key Laboratory of Chemical Biology of Fujian Province, and College of Chemistry and Chemical Engineering, Xiamen University, Xiamen 361005, China

[b]State Key Laboratory of Organometallic Chemistry, Shanghai Institute of Organic Chemistry, Chinese Academy of Sciences, Shanghai 200032, China

[c]State Key Laboratory of Elemento-Organic Chemistry, Nankai University, Tianjin 300071, China

E-mail: zhoubo@xmu.edu.cn; xinlu@xmu.edu.cn

Content	Page Number
1. General Information	2
2. More Reaction Condition and Scope Studies	3
3. Preparation of Starting Materials	5
4. General Procedures for the Intramolecular (4 + 2) Annulation	31
5. Racemization Experiments	55
6. Synthetic Utility Study	60
7. Crystal Data	63
8. Computational Studies	64
9. HPLC Chromatograms	133
10. NMR Spectra	172

1. General Information

Acetonitrile (ACS grade), toluene (ACS grade), ethyl acetate (ACS grade), 1,2-dichlorobenzene (ACS grade), chlorobenzene (ACS grade), fluorobenzene (ACS grade), methanol (ACS grade) and hexanes (ACS grade) were obtained commercially and used without further purification. Methylene chloride, tetrahydrofuran and diethyl ether were purified according to standard methods unless otherwise noted. Commercially available reagents were used without further purification. All reactions were carried out with a Titan HMS-14 digital magnetic stirrer with hot plate. Reactions were monitored by thin layer chromatography (TLC) using silicycle pre-coated silica gel plates. Flash column chromatography was performed over silica gel (300-400 mesh). Infrared spectra were recorded on a Nicolet AVATER FTIR330 spectrometer as thin film and are reported in reciprocal centimeter (cm^{-1}). Mass spectra were recorded with Micromass QTOF2 Quadrupole/Time-of-Flight Tandem mass spectrometer using electron spray ionization. X-ray diffraction analysis was recorded on a Rigaku AFC7R X-ray single crystal diffractometer. HPLC analyses were carried out in a chromatograph equipped with a UV diode-array detector using chiral stationary columns from Daicel.

^{1}H NMR spectra and ^{13}C NMR spectra were recorded on a Bruker AV-400 spectrometer and Zhongke Oxford WNMR-I-400MHz spectrometer in chloroform-d_3. Chemical shifts are reported in ppm with the internal TMS signal at 0.0 ppm as a standard for ^{1}H NMR spectra and with the internal chloroform signal at 77.0 ppm as a standard for ^{13}C NMR spectra. The data is being reported as (s = singlet, d = doublet, t = triplet, m = multiplet or unresolved, brs = broad singlet, coupling constant(s) in Hz, integration).

2. More Reaction Condition and Scope Studies

Supplementary Table 1. Screening of more reaction conditions for the intramolecular (4 + 2) annulation.[a]

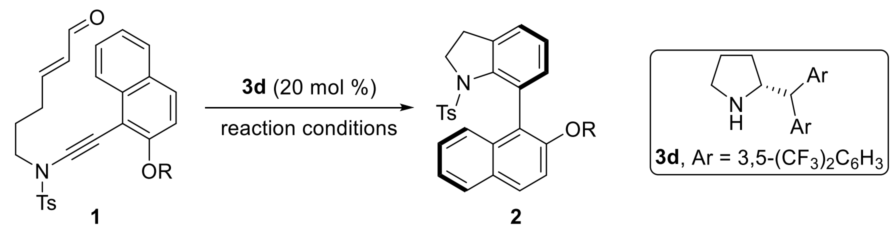

Entry	R	Reaction conditions	Additives	Yield[b] (%)	Ee[c] (%)
1	Me	DCE, rt, 12 h	-	62 (<5)	47
2	^{i}Pr	DCE, rt, 30 h	-	47 (<5)	13
3	MOM	DCE, rt, 48 h	-	53 (<5)	56
4	Ts	DCE, 60 °C, 12 h	-	60 (<5)	75
5	Mts	DCE, 60 °C, 12 h	-	65 (<5)	86
6	Mts	C_6F_6, 60 °C, 48 h	-	46 (<5)	85
7	Mts	MeOH, 60 °C, 12 h	-	<5 (95)	n.d.
8	Mts	DCE, 60 °C, 72 h	3 Å MS	28 (35)	86
9	Mts	DCE, 60 °C, 36 h	4 Å MS	60 (<5)	86
10	Mts	DCE, 60 °C, 72 h	5 Å MS	35 (26)	86
11	Mts	DCE, 60 °C, 12 h	H_2O (20 mol %)	46 (<5)	86
12	Mts	DCE, 60 °C, 24 h	H_2O (1 equiv)	31 (<5)	85
13	Mts	DCE, 60 °C, 18 h	Et_3N (20 mol %)	62 (<5)	86
14	Mts	DCE, 60 °C, 18 h	K_2CO_3 (20 mol %)	57 (<5)	86

[a] Reaction conditions: **1** (0.05 mmol), **3d** (0.01 mmol), solvent (1 mL), rt to 60 °C, 12–72 h, in vials. [b] Measured by ^{1}H NMR using diethyl phthalate as the internal reference. Recovered unreacted starting material given within parentheses. [c] Determined by HPLC analysis. Mts = 2-mesitylenesulfonyl, DCB = 1,2-dichlorobenzene.

Supplementary Table 2. Screening of other ynamides **1ag–1aj** for the intramolecular (4 + 2) annulation.[a]

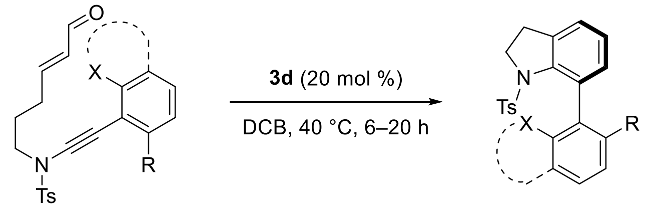

1ag–1aj **2ag–2aj**

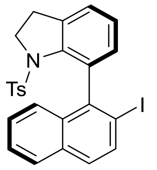

2ag, 79%, 25% ee (6 h)

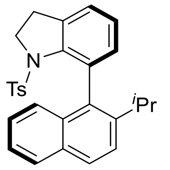

2ah, 72%, 35% ee (12 h)

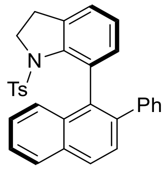

2ai, 76%, 46% ee (18 h)

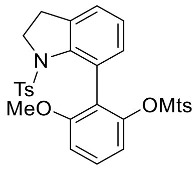

2aj, 30%, <5% ee (20 h)

[a] Reaction conditions: **1** (0.1 mmol), **3d** (0.02 mmol), DCB (2 mL), 40 °C, 6–20 h, in vials. Yields are those of isolated products; the ee values are determined by HPLC analysis.

3. Preparation of Starting Materials

Compounds **1a–1aj** were prepared according to the following procedures.[1–4]

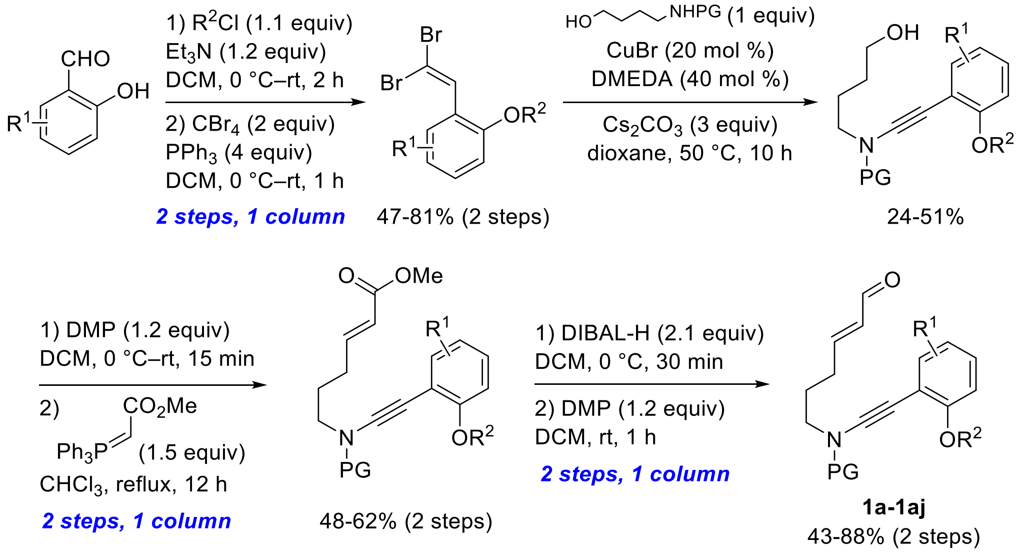

To a solution of salicylaldehyde derivative (20 mmol) in DCM (40 mL), was added Et_3N (24 mmol, 3.3 mL). After cooling to 0 ºC, sulfonyl chroloride (R^2Cl, 22 mmol) was added to this solution and the reaction mixture was stirred at room temperature for 2 h. Upon completion (monitored by TLC), the reaction was quenched with 1 N HCl, extracted with DCM for three times, dried over $MgSO_4$ and filtered. The resulting mixture was concentrated under reduced pressure to give crude product without further purification. To the solution of PPh_3 (80 mmol, 20.98 g) in DCM (60 mL) was added CBr_4 (40 mmol, 13.26 g) carefully at 0 ºC, and the reaction was stirred at this temperature for additional 15 min. The solution of the above crude product in DCM (20 mL) was then added to the mixture at 0 ºC. The reaction mixture was warmed to room temperature and stirred for 1 h. Upon completion (monitored by TLC), the reaction mixture was filtered through a Celite pad and the filtrate was concentrated under reduced pressure. The residue was purified by column chromatography on silica gel (eluent: PE/DCM) to afford the desired product (47–81% yield, 2 steps).

To a solution of the above product (8 mmol) in 1,4-dioxane (40 mL) were added *N*-protected 4-aminobutan-1-ol (8 mmol), Cs_2CO_3 (24 mmol, 7.82 g), DMEDA (3.2 mmol, 0.35 mL) and CuBr (1.6 mmol, 0.23 g). The reaction was stirred at 50 ºC for 10 h. Upon completion (monitored by TLC), the reaction mixture was filtered through a Celite pad

and the filtrate was concentrated under reduced pressure. The residue was purified by column chromatography on silica gel (eluent: PE/EtOAc) to afford the target product (24–51% yield).

To a solution of the above product (2.0 mmol) in DCM (10 mL) was added DMP (2.4 mmol, 1.02 g) at 0 °C. The reaction mixture was warmed to room temperature and stirred for 1 h. Upon completion (monitored by TLC), the reaction was quenched with $NaHCO_3$ (aq), extracted with DCM for three times, dried over $MgSO_4$ and filtered. The filtrate was concentrated under reduced pressure to give crude aldehyde product without further purification. To a solution of the above crude aldehyde in $CHCl_3$ (6 mL) was added methyl 2-(triphenyl-λ^5-phosphanylidene)acetate (3 mmol, 1.00 g). The reaction was heated to reflux and stirred for 12 h. The progress of the reaction was monitored by TLC. Upon completion, the reaction mixture was concentrated and purified by column chromatography on silica gel (eluent: PE/EtOAc) to give unsaturated ester (48–62% yield, 2 steps).

To a solution of the above unsaturated ester (1.0 mmol) in DCM (5 mL) was added DIBAL-H (1.5 N in toluene, 2.1 mmol, 1.4 mL) at 0 °C dropwise. After stirring at 0 °C for 30 min, the reaction was carefully quenched with 1 N HCl, extracted with DCM for three times, dried over $MgSO_4$ and filtered. The filtrate was concentrated under reduced pressure to give crude alcohol product without further purification. The residue was dissolved in DCM (5 mL), and DMP (1.2 mmol, 0.51 g) was added at 0 °C. The reaction mixture was warmed to room temperature and stirred for 1 h. Upon completion (monitored by TLC), the reaction was diluted by DCM and was quenched with $NaHCO_3$ (aq). The mixture was extracted with DCM for three times, dried over $MgSO_4$ and filtered. The filtrate was concentrated under reduced pressure and purified by column chromatography on silica gel (eluent: PE/EtOAc) to afford the desired ynamide **1a**–**1aj** (43–88% yield, 2 steps).

(*E*)-1-(((4-methyl-*N*-(6-oxohex-4-en-1-yl)phenyl)sulfonamido)ethynyl)naphthalen-2-yl 2,4,6-trimethylbenzenesulfonate (1a)

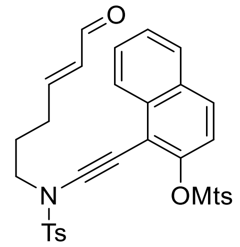

1a

Compound **1a** was prepared in 78% yield (481 mg) with *E*/*Z* = 15/1 according to the general procedure as a pale yellow oil. ^{1}H NMR (400 MHz, $CDCl_3$) δ 9.49 (d, *J* = 8.0 Hz, 1H), 8.34 (d, *J* = 8.4 Hz, 1H), 7.91 (d, *J* = 8.0 Hz, 2H), 7.76 (d, *J* = 8.0 Hz, 1H), 7.63 – 7.48 (m, 3H), 7.34 (d, *J* = 8.0 Hz, 2H), 6.98 (s, 2H), 6.94 – 6.87 (m, 1H), 6.60 (d, *J* = 9.2 Hz, 1H), 6.13 (dd, *J* = 15.6, 8.0 Hz, 1H), 3.56 (t, *J* = 6.8 Hz, 2H), 2.54 (s, 6H), 2.51 – 2.46 (m, 2H), 2.42 (s, 3H), 2.33 (s, 3H), 2.14 – 2.06 (m, 2H); ^{13}C NMR (100 MHz, $CDCl_3$) δ 194.0, 157.2, 147.2, 144.9, 143.9, 140.0, 134.4, 133.5, 133.3, 131.8, 131.7, 131.2, 129.9, 128.5, 127.9, 127.6, 127.5, 126.6, 126.4, 119.0, 115.2, 92.1, 65.2, 50.9, 29.3, 25.9, 22.6, 21.5, 21.0; IR (neat): 3059 (bs), 2234 (s), 1688, 1595, 1507, 1367, 1170, 1090, 951, 790, 568; HRESIMS Calcd for $[C_{34}H_{33}NNaO_6S_2]^+$ ($M + Na^+$) 638.1642, found 638.1645.

(*E*)-1-(((4-methoxy-*N*-(6-oxohex-4-en-1-yl)phenyl)sulfonamido)ethynyl)naphthalen-2-yl 2,4,6-trimethylbenzenesulfonate (1b)

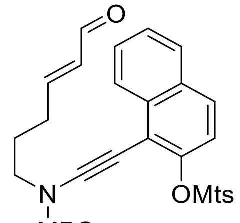

1b

Compound **1b** was prepared in 56% yield (352 mg) with *E*/*Z* = 19/1 according to the general procedure as a pale yellow oil. ^{1}H NMR (400 MHz, $CDCl_3$) δ 9.49 (d, *J* = 8.0 Hz, 1H), 8.33 (d, *J* = 8.4 Hz, 1H), 7.96 (d, *J* = 8.8 Hz, 2H), 7.77 (d, *J* = 8.1 Hz, 1H), 7.63 – 7.59 (m, 1H), 7.56 – 7.48 (m, 2H), 7.03 – 6.99 (m, 4H), 6.94 – 6.87 (m, 1H), 6.60 (d, *J* = 8.8 Hz, 1H), 6.14 (dd, *J* = 15.6, 8.0 Hz, 1H), 3.85 (s, 3H), 3.55 (t, *J* = 6.8

Hz, 2H), 2.54 (s, 6H), 2.52 – 2.46 (m, 2H), 2.34 (s, 3H), 2.14 – 2.06 (m, 2H); ^{13}C NMR (100 MHz, $CDCl_3$) δ 194.0, 163.8, 157.2, 147.3, 143.9, 140.1, 133.6, 133.3, 131.8, 131.3, 129.8, 128.9, 128.4, 128.0, 127.6, 126.6, 126.5, 119.1, 115.3, 114.5, 92.4, 65.3, 55.6, 50.8, 29.4, 26.0, 22.7, 21.0; IR (neat): 2940 (bs), 2241 (s), 1688, 1467, 1363, 1191, 1074, 798, 541; HRESIMS Calcd for $[C_{34}H_{33}NNaO_7S_2]^+$ (M + Na$^+$) 654.1591, found 654.1595.

(*E*)-1-(((2,4,6-trimethyl-*N*-(6-oxohex-4-en-1-yl)phenyl)sulfonamido)ethynyl)naphthalen-2-yl 2,4,6-trimethylbenzenesulfonate (1c)

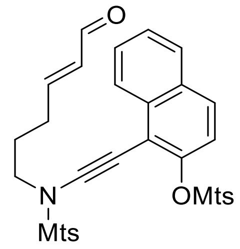

1c

Compound **1c** was prepared in 43% yield (277 mg) with *E*/*Z* > 20/1 according to the general procedure as a pale yellow solid (mp 149–151 ºC). ^{1}H NMR (400 MHz, $CDCl_3$) δ 9.48 (d, *J* = 7.6 Hz, 1H), 7.81 (d, *J* = 7.6 Hz, 1H), 7.72 (d, *J* = 7.6 Hz, 1H), 7.50 (d, *J* = 8.8 Hz, 1H), 7.47 – 7.39 (m, 2H), 7.06 – 6.98 (m, 4H), 6.93 – 6.86 (m, 1H), 6.60 (d, *J* = 8.8 Hz, 1H), 6.13 (dd, *J* = 15.6, 7.8 Hz, 1H), 3.65 (t, *J* = 6.8 Hz, 2H), 2.70 (s, 6H), 2.56 (s, 6H), 2.52 – 2.49 (m, 2H), 2.35 (s, 3H), 2.34 (s, 3H), 2.23 – 2.16 (m, 2H); ^{13}C NMR (100 MHz, $CDCl_3$) δ 194.0, 157.3, 147.2, 143.9, 141.0, 140.1, 133.4, 132.2, 131.9, 131.5, 131.3, 128.2, 127.9, 127.1, 126.5, 119.1, 115.6, 92.0, 66.4, 49.8, 29.5, 26.2, 23.0, 22.7, 21.0; IR (neat): 2940 (bs), 2235 (s), 1682, 1649, 1364, 1173, 949, 727, 542; HRESIMS Calcd for $[C_{36}H_{37}NNaO_6S_2]^+$ (M + Na$^+$) 666.1955, found 666.1951.

(*E*)-1-((*N*-(6-oxohex-4-en-1-yl)phenylsulfonamido)ethynyl)naphthalen-2-yl 2,4,6-trimethylbenzenesulfonate (1d)

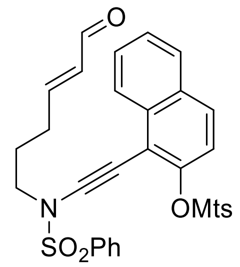

1d

Compound **1d** was prepared in 51% yield (305 mg) with *E*/*Z* = 16/1 according to the general procedure as a pale yellow oil. ^{1}H NMR (400 MHz, $CDCl_3$) δ 9.49 (d, *J* = 8.0 Hz, 1H), 8.33 (d, *J* = 8.0 Hz, 1H), 8.04 (dd, *J* = 8.4, 0.8 Hz, 2H), 7.77 (d, *J* = 8.0 Hz, 1H), 7.68 – 7.49 (m, 6H), 6.99 (s, 2H), 6.93 – 6.86 (m, 1H), 6.60 (d, *J* = 9.2 Hz, 1H), 6.13 (dd, *J* = 15.6, 8.0 Hz, 1H), 3.57 (t, *J* = 7.2 Hz, 2H), 2.54 (s, 6H), 2.51 – 2.45 (m, 2H), 2.34 (s, 3H), 2.14 – 2.06 (m, 2H); ^{13}C NMR (100 MHz, $CDCl_3$) δ 194.0, 157.1, 147.4, 143.9, 140.1, 137.5, 133.8, 133.6, 133.4, 131.8, 131.7, 131.3, 129.4, 128.6, 128.0, 127.7, 127.5, 126.7, 126.5, 119.1, 115.2, 91.9, 65.3, 51.0, 29.3, 26.0, 22.7, 21.0; IR (neat): 2956 (bs), 2236 (s), 1680, 1649, 1364, 1172, 949, 729, 552; HRESIMS Calcd for $[C_{33}H_{31}NNaO_6S_2]^+$ (M + Na$^+$) 624.1485, found 624.1486.

(*E*)-1-(((4-bromo-*N*-(6-oxohex-4-en-1-yl)phenyl)sulfonamido)ethynyl)naphthalen-2-yl 2,4,6-trimethylbenzenesulfonate (1e)

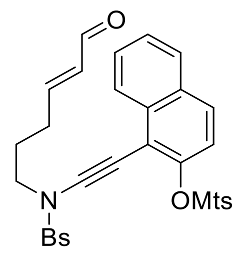

1e

Compound **1e** was prepared in 62% yield (423 mg) with *E*/*Z* = 8/1 according to the general procedure as a pale yellow oil. ^{1}H NMR (400 MHz, $CDCl_3$) δ 9.50 (d, *J* = 7.6 Hz, 1H), 8.29 (d, *J* = 8.4 Hz, 1H), 7.90 (d, *J* = 8.4 Hz, 2H), 7.78 (d, *J* = 8.0 Hz, 1H), 7.70 (d, *J* = 8.4 Hz, 2H), 7.64 – 7.50 (m, 3H), 7.00 (s, 2H), 6.94 – 6.87 (m, 1H), 6.58 (d, *J* = 8.8 Hz, 1H), 6.16 (dd, *J* = 15.6, 7.6 Hz, 1H), 3.58 (t, *J* = 7.2 Hz, 2H), 2.54 (s,

6H), 2.52 – 2.48 (m, 2H), 2.35 (s, 3H), 2.19 – 2.09 (m, 2H); ^{13}C NMR (100 MHz, $CDCl_3$) δ 194.0, 156.9, 147.7, 144.0, 140.1, 136.3, 133.6, 133.4, 132.7, 131.9, 131.7, 131.3, 129.1, 129.0, 128.9, 128.1, 127.8, 126.7, 126.3, 119.1, 115.0, 91.5, 65.4, 51.2, 29.3, 26.1, 22.7, 21.1; IR (neat): 2942 (bs), 2237 (s), 1689, 1367, 1175, 952, 782, 534; HRESIMS Calcd for $[C_{33}H_{30}BrNNaO_6S_2]^+$ (M + Na^+) 702.0590, found 702.0598.

(E)-1-((*N*-(6-oxohex-4-en-1-yl)methylsulfonamido)ethynyl)naphthalen-2-yl 2,4,6-trimethylbenzenesulfonate (1f)

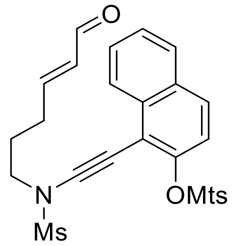

1f

Compound **1f** was prepared in 44% yield (235 mg) with *E*/*Z* > 20/1 according to the general procedure as a pale yellow oil. ^{1}H NMR (400 MHz, $CDCl_3$) δ 9.52 (dd, J = 8.0, 0.8 Hz, 1H), 8.29 (d, J = 8.4 Hz, 1H), 7.77 (d, J = 8.0 Hz, 1H), 7.63 – 7.49 (m, 3H), 7.03 (s, 2H), 6.98 – 6.91 (m, 1H), 6.54 (d, J = 8.8 Hz, 1H), 6.20 (dd, J = 15.6, 8.8 Hz, 1H), 3.74 (t, J = 7.2 Hz, 2H), 3.27 (s, 3H), 2.58 (s, 6H), 2.60 – 2.54 (m, 2H), 2.37 (s, 3H), 2.24 – 2.16 (m, 2H); ^{13}C NMR (100 MHz, $CDCl_3$) δ 194.0, 156.8, 147.9, 144.0, 140.2, 133.6, 133.5, 131.9, 131.8, 131.4, 129.0, 128.0, 127.8, 126.8, 126.5, 119.1, 115.3, 91.5, 65.5, 51.2, 38.7, 29.4, 26.5, 22.8, 21.1; IR (neat): 3061 (bs), 2234 (s), 1687, 1367, 1171, 950, 824, 671, 544, 521; HRESIMS Calcd for $[C_{28}H_{29}NNaO_6S_2]^+$ (M + Na^+) 562.1329, found 562.1333.

(*E*)-1-(((4-methyl-*N*-(6-oxohex-4-en-1-yl)phenyl)sulfonamido)ethynyl)naphthalen-2-yl 4-methylbenzenesulfonate (1g)

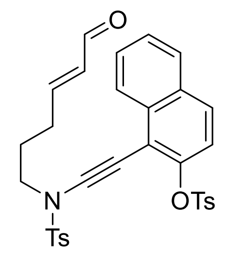

1g

Compound **1g** was prepared in 56% yield (332 mg) with *E*/*Z* = 8/1 according to the general procedure as a white solid (mp 127–129 ºC). ^{1}H NMR (400 MHz, $CDCl_3$) δ 9.50 (d, *J* = 7.6 Hz, 1H), 8.23 (d, *J* = 8.4 Hz, 1H), 7.92 (d, *J* = 8.0 Hz, 2H), 7.79 (d, *J* = 8.0 Hz, 1H), 7.75 (d, *J* = 8.0 Hz, 2H), 7.66 – 7.49 (m, 3H), 7.37 (d, *J* = 8.0 Hz, 2H), 7.28 (d, *J* = 8.0 Hz, 2H), 6.98 (d, *J* = 8.8 Hz, 1H), 6.93 – 6.85 (m, 1H), 6.13 (dd, *J* = 15.6, 7.8 Hz, 1H), 3.54 (t, *J* = 6.8 Hz, 2H), 2.52 – 2.46 (m, 2H), 2.44 (s, 3H), 2.42 (s, 3H), 2.11 – 2.04 (m, 2H); ^{13}C NMR (100 MHz, $CDCl_3$) δ 194.0, 156.9, 147.4, 145.5, 145.0, 134.5, 133.5, 133.4, 132.9, 131.4, 130.0, 129.8, 128.6, 128.5, 128.1, 127.6, 126.7, 126.4, 120.0, 114.8, 92.1, 65.0, 51.0, 29.3, 26.1, 21.7, 21.6; IR (neat): 2925 (bs), 2234 (s), 1689, 1595, 1495, 1371, 1175, 1092, 951, 817, 672; HRESIMS Calcd for $[C_{32}H_{29}NNaO_6S_2]^+$ (M + Na^+) 610.1329, found 610.1325.

(*E*)-1-(((4-methyl-*N*-(6-oxohex-4-en-1-yl)phenyl)sulfonamido)ethynyl)naphthalen-2-yl-4-methoxybenzenesulfonate (1h)

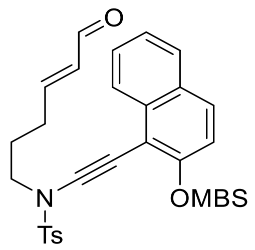

1h

Compound **1h** was prepared in 52% yield (316 mg) with *E*/*Z* > 20/1 according to the general procedure as a pale yellow solid (mp 123–125 ºC). ^{1}H NMR (400 MHz, $CDCl_3$) δ 9.50 (d, *J* = 7.6 Hz, 1H), 8.20 (d, *J* = 8.4 Hz, 1H), 7.92 (d, *J* = 8.0 Hz, 2H), 7.80 – 7.77 (m, 3H), 7.65 (d, *J* = 9.2 Hz, 1H), 7.59 – 7.49 (m, 2H), 7.37 (d, *J* = 8.0 Hz, 2H), 7.05 (d, *J* = 9.2 Hz, 1H), 6.93 – 6.85 (m, 3H), 6.13 (dd, *J* = 15.6, 7.6 Hz, 1H), 3.85 (s, 3H), 3.54 (t, *J* = 6.8 Hz, 2H), 2.51 – 2.46 (m, 2H), 2.44 (s, 3H), 2.10 – 2.03 (m, 2H);

^{13}C NMR (100 MHz, $CDCl_3$) δ 193.9, 164.1, 156.8, 147.5, 145.1, 134.4, 133.5, 133.4, 131.4, 130.7, 130.0, 128.6, 128.1, 127.6, 127.5, 127.0, 126.7, 126.3, 120.1, 114.7, 114.3, 92.0, 64.9, 55.7, 50.9, 29.3, 26.1, 21.6; IR (neat): 2929 (bs), 2235 (s), 1687, 1595, 1499, 1370, 1187, 1095, 951, 822, 717; HRESIMS Calcd for $[C_{32}H_{29}NNaO_7S_2]^+$ (M + Na^+) 626.1278, found 626.1286.

(*E*)-1-(((4-methyl-*N*-(6-oxohex-4-en-1-yl)phenyl)sulfonamido)ethynyl)naphthalen-2-yl 4-(*tert*-butyl)benzenesulfonate (1i)

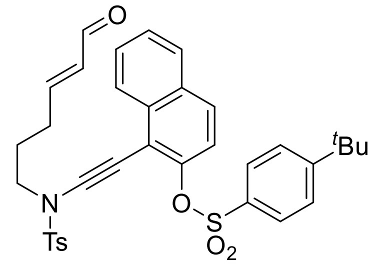

1i

Compound **1i** was prepared in 67% yield (424 mg) with *E*/*Z* > 20/1 according to the general procedure as a pale yellow oil. ^1H NMR (400 MHz, $CDCl_3$) δ 9.49 (d, *J* = 7.6 Hz, 1H), 8.25 (d, *J* = 8.0 Hz, 1H), 7.92 (d, *J* = 8.0 Hz, 2H), 7.83 – 7.79 (m, 3H), 7.65 – 7.51 (m, 5H), 7.36 (d, *J* = 7.6 Hz, 2H), 6.98 (d, *J* = 7.6 Hz, 1H), 6.92 – 6.85 (m, 1H), 6.13 (dd, *J* = 15.6, 7.6 Hz, 1H), 3.55 (t, *J* = 6.8 Hz, 2H), 2.49 – 2.47 (m, 2H), 2.43 (s, 3H), 2.09 – 2.06 (m, 2H), 1.33 (s, 9H); ^{13}C NMR (100 MHz, $CDCl_3$) δ 193.9, 158.4, 156.8, 147.4, 145.0, 134.5, 133.5, 133.4, 133.0, 131.4, 130.0, 128.6, 128.3, 128.1, 127.6, 126.7, 126.4, 126.2, 120.0, 114.9, 92.2, 65.0, 51.0, 35.3, 30.9, 29.3, 26.1, 21.6; IR (neat): 2966 (bs), 2234 (s), 1693, 1423, 1216, 1174, 1141, 948, 835, 616, 590; HRESIMS Calcd for $[C_{35}H_{35}NNaO_6S_2]^+$ (M + Na^+) 652.1798, found 652.1802.

(*E*)-1-(((4-methyl-*N*-(6-oxohex-4-en-1-yl)phenyl)sulfonamido)ethynyl)naphthalen-2-yl 4-bromobenzenesulfonate (1j)

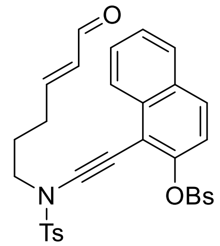

1j

Compound **1j** was prepared in 54% yield (350 mg) with *E*/*Z* > 20/1 according to the general procedure as a pale yellow oil. ^{1}H NMR (400 MHz, $CDCl_3$) δ 9.50 (d, *J* = 7.6 Hz, 1H), 8.16 (d, *J* = 8.4 Hz, 1H), 7.92 (d, *J* = 8.4 Hz, 2H), 7.81 (d, *J* = 7.6 Hz, 1H), 7.74 – 7.67 (m, 3H), 7.62 – 7.50 (m, 4H), 7.38 (d, *J* = 8.0 Hz, 2H), 7.10 (d, *J* = 9.2 Hz, 1H), 6.91 – 6.84 (m, 1H), 6.17 – 6.11 (m, 1H), 3.53 (t, *J* = 6.8 Hz, 2H), 2.51 – 2.47 (m, 2H), 2.45 (s, 3H), 2.08 – 2.01 (m, 2H); ^{13}C NMR (100 MHz, $CDCl_3$) δ 193.8, 156.6, 147.2, 145.1, 134.8, 134.3, 133.5, 132.4, 131.5, 130.1, 130.0, 129.6, 128.9, 128.2, 127.7, 127.6, 126.9, 126.2, 119.9, 114.6, 92.2, 64.7, 50.9, 29.2, 26.2, 21.6; IR (neat): 2965 (bs), 2237 (s), 1687, 1370, 1239, 1098, 962, 823, 525; HRESIMS Calcd for $[C_{31}H_{26}BrNNaO_6S_2]^+$ (M + Na$^+$) 674.0277, found 674.0282.

(*E*)-1-(((4-methyl-*N*-(6-oxohex-4-en-1-yl)phenyl)sulfonamido)ethynyl)naphthalen-2-yl 4-nitrobenzenesulfonate (1k)

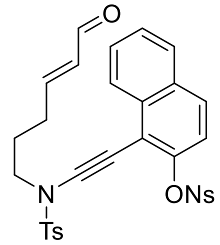

1k

Compound **1k** was prepared in 58% yield (359 mg) with *E*/*Z* > 20/1 according to the general procedure as a yellow solid (mp 145–147 ºC). ^{1}H NMR (400 MHz, $CDCl_3$) δ 9.50 (d, *J* = 7.6 Hz, 1H), 8.31 (d, *J* = 7.2 Hz, 2H), 8.10 (d, *J* = 8.4 Hz, 2H), 7.91 (d, *J* = 7.2 Hz, 2H), 7.83 (d, *J* = 8.0 Hz, 1H), 7.71 (d, *J* = 8.8 Hz, 1H), 7.64 – 7.52 (m, 3H), 7.39 (d, *J* = 7.6 Hz, 2H), 7.21 – 7.14 (m, 1H), 6.90 – 6.83 (m, 1H), 6.14 (dd, *J* = 15.6, 7.6 Hz, 1H), 3.54 (t, *J* = 6.4 Hz, 2H), 2.54 – 2.49 (m, 2H), 2.46 (s, 3H), 2.07 – 2.01 (m, 2H); ^{13}C NMR (100 MHz, $CDCl_3$) δ 193.8, 156.2, 151.0, 146.9, 145.3, 141.2, 134.3,

133.5, 133.4, 131.6, 130.1, 130.0, 129.1, 128.3, 127.9, 127.6, 127.1, 126.2, 124.2, 119.8, 114.5, 92.6, 64.5, 51.0, 29.2, 26.2, 21.6; IR (neat): 2933 (bs), 2234 (s), 1687, 1548, 1366, 1170, 950, 737, 672, 587; HRESIMS Calcd for $[C_{31}H_{26}N_2NaO_8S_2]^+$ (M + Na^+) 641.1023, found 641.1025.

(*E*)-1-(((4-methyl-*N*-(6-oxohex-4-en-1-yl)phenyl)sulfonamido)ethynyl)naphthalen-2-yl 2-nitrobenzenesulfonate (1l)

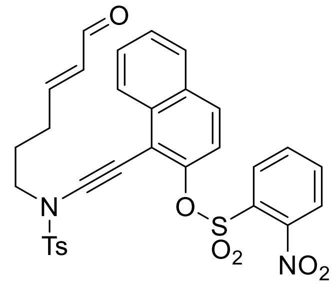

1l

Compound **1l** was prepared in 43% yield (266 mg) with *E*/*Z* = 9/1 according to the general procedure as a pale yellow oil. ^{1}H NMR (400 MHz, $CDCl_3$) δ 9.49 (d, *J* = 8.0 Hz, 1H), 8.30 (d, *J* = 8.0 Hz, 1H), 8.03 (d, *J* = 8.0 Hz, 1H), 7.89 – 7.81 (m, 6H), 7.73 – 7.61 (m, 2H), 7.57 – 7.54 (m, 1H), 7.36 (d, *J* = 8.0 Hz, 2H), 7.01 (d, *J* = 9.2 Hz, 1H), 6.90 – 6.83 (m, 1H), 6.11 (dd, *J* = 15.6, 8.0 Hz, 1H), 3.54 (t, *J* = 6.8 Hz, 2H), 2.49 – 2.47 (m, 2H), 2.45 (s, 3H), 2.09 – 2.01 (m, 2H); ^{13}C NMR (100 MHz, $CDCl_3$) δ 194.1, 156.9, 148.5, 147.01, 145.1, 135.4, 134.5, 133.7, 133.5, 132.3, 131.7(3), 131.7(0), 130.1, 129.5, 128.9, 128.2, 127.9, 127.6, 127.1, 126.6, 124.9, 119.8, 115.3, 92.8, 64.7, 51.0, 29.4, 26.2, 21.6; IR (neat): 2930 (bs), 2233 (s), 1687, 1534, 1351, 1206, 1169, 1091, 952, 742, 545; HRESIMS Calcd for $[C_{31}H_{26}N_2NaO_8S_2]^+$ (M + Na^+) 641.1023, found 641.1027.

(*E*)-1-(((4-methyl-*N*-(6-oxohex-4-en-1-yl)phenyl)sulfonamido)ethynyl)naphthalen-2-yl naphthalene-1-sulfonate (1m)

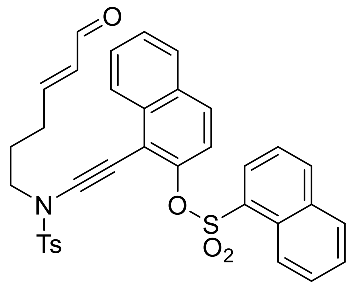

1m

Compound **1m** was prepared in 66% yield (412 mg) with *E*/*Z* = 19/1 according to the general procedure as a pale yellow oil. ^{1}H NMR (400 MHz, $CDCl_3$) δ 9.48 (d, *J* = 8.0 Hz, 1H), 8.80 (d, *J* = 8.4 Hz, 1H), 8.32 (d, *J* = 8.4 Hz, 1H), 8.15 (d, *J* = 8.0 Hz, 1H), 8.11 (d, *J* = 7.6 Hz, 1H), 7.98 (d, *J* = 8.0 Hz, 1H), 7.94 – 7.92 (m, 2H), 7.73 – 7.58 (m, 4H), 7.51 – 7.42 (m, 3H), 7.36 (d, *J* = 8.0 Hz, 2H), 6.91 – 6.83 (m, 1H), 6.44 (d, *J* = 9.2 Hz, 1H), 6.15 – 6.09 (m, 1H), 3.50 (t, *J* = 6.8 Hz, 2H), 2.48 – 2.46 (m, 2H), 2.44 (s, 3H), 2.09 – 2.02 (m, 3H); ^{13}C NMR (100 MHz, $CDCl_3$) δ 194.0, 157.1, 147.2, 145.0, 135.8, 134.5, 134.1, 133.6, 133.4, 131.9, 131.4, 130.8, 130.0, 129.0, 128.9, 128.5, 128.3, 128.0, 127.7, 127.6, 127.4, 126.8, 126.5, 125.1, 124.0, 119.4, 115.3, 92.4, 65.2, 50.9, 29.4, 26.0, 21.6; IR (neat): 2923(bs), 2229(s), 1688, 1361, 1169, 825, 736, 570; HRESIMS Calcd for $[C_{35}H_{29}NNaO_6S_2]^+$ (M + Na$^+$) 646.1329, found 646.1320.

(*E*)-1-(((4-methyl-N-(6-oxohex-4-en-1-yl)phenyl)sulfonamido)ethynyl)naphthalen-2-yl methanesulfonate (1n)

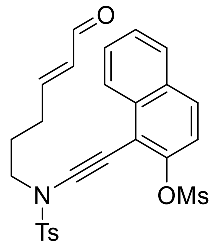

1n

Compound **1n** was prepared in 80% yield (412 mg) with *E*/*Z* > 20/1 according to the general procedure as a yellow oil. ^{1}H NMR (400 MHz, $CDCl_3$) δ 9.49 (d, *J* = 8.0 Hz, 1H), 8.13 (d, *J* = 8.0 Hz, 1H), 7.92 (d, *J* = 8.0 Hz, 2H), 7.85 (d, *J* = 8.0 Hz, 1H), 7.80 (d, *J* = 9.2 Hz, 1H), 7.60 – 7.51 (m, 3H), 7.36 (d, *J* = 8.0 Hz, 2H), 6.89 – 6.82 (m, 1H), 6.14 (dd, *J* = 15.6, 7.6 Hz, 1H), 3.58 (t, *J* = 6.8 Hz, 2H), 3.26 (s, 3H), 2.52 – 2.48 (m,

2H), 2.42 (s, 3H), 2.10 – 2.03 (m, 2H); ^{13}C NMR (100 MHz, $CDCl_3$) δ 193.7, 156.3, 147.3, 145.2, 134.0, 133.4, 133.1, 131.5, 130.0, 129.2, 128.3, 127.7, 127.5, 126.8, 125.8, 121.0, 113.5, 92.2, 64.7, 50.8, 37.8, 29.1, 26.1, 21.5; IR (neat): 2937 (bs), 2234 (s), 1683, 1367, 1172, 949, 818, 588; HRESIMS Calcd for $[C_{26}H_{25}NNaO_6S_2]^+$ (M + Na^+) 534.1016, found 534.1022.

(*E*)-1-(((4-methyl-*N*-(6-oxohex-4-en-1-yl)phenyl)sulfonamido)ethynyl)naphthalen-2-yl-trifluoromethanesulfonate (1o)

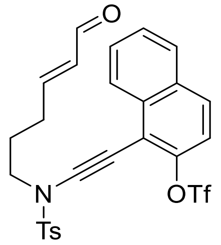

1o

Compound **1o** was prepared in 67% yield (382 mg) with *E*/*Z* = 7/1 according to the general procedure as a pale yellow oil. ^{1}H NMR (400 MHz, $CDCl_3$) δ 9.50 (d, *J* = 8.0 Hz, 1H), 8.37 (d, *J* = 8.0 Hz, 1H), 7.90 – 7.84 (m, 3H), 7.82 (d, *J* = 9.2 Hz, 1H), 7.71 – 7.67 (m, 1H), 7.63 – 7.59 (m, 1H), 7.35 (d, *J* = 8.0 Hz, 2H), 7.32 (d, *J* = 9.2 Hz, 1H), 6.88 – 6.81 (m, 1H), 6.16 – 6.10 (m, 1H), 3.57 (t, *J* = 7.2 Hz, 2H), 2.51 – 2.42 (m, 2H), 2.44 (s, 3H), 2.08 – 2.01 (m, 2H); ^{13}C NMR (100 MHz, $CDCl_3$) δ 193.8, 156.4, 147.0, 145.2, 134.4, 133.5, 132.0, 130.8, 130.0, 129.5, 128.4, 128.2, 127.6, 127.5, 126.7, 118.8, 118.6 (q, *J* = 320.7 Hz), 115.2, 93.9, 63.8, 51.0, 29.2, 26.3, 21.6; ^{19}F NMR (376 MHz, $CDCl_3$) δ -73.7; IR (neat): 3015 (bs), 2234 (s), 1689, 1422, 1215, 948, 834, 614, 545; HRESIMS Calcd for $[C_{26}H_{22}F_3NNaO_6S_2]^+$ (M + Na^+) 588.0733, found 588.0730.

(*E*)-1-(((4-methyl-*N*-(6-oxohex-4-en-1-yl)phenyl)sulfonamido)ethynyl)-3-phenylnaphthalen-2-yl 2,4,6-trimethylbenzenesulfonate (1p)

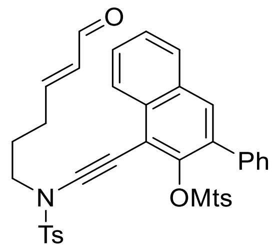

1p

Compound **1p** was prepared in 83% yield (574 mg) with *E*/*Z* = 14/1 according to the general procedure as a pale yellow oil. ^{1}H NMR (400 MHz, CDCl$_3$) δ 9.47 (d, *J* = 8.0 Hz, 1H), 8.44 (d, *J* = 8.0 Hz, 1H), 7.94 (d, *J* = 8.4 Hz, 2H), 7.79 (d, *J* = 8.0 Hz, 1H), 7.65 – 7.51 (m, 3H), 7.34 (d, *J* = 8.0 Hz, 2H), 7.15 – 7.13 (m, 2H), 7.02 – 6.97 (m, 3H), 6.93 – 6.86 (m, 1H), 6.57 (s, 2H), 6.17 – 6.10 (m, 1H), 3.63 (t, *J* = 7.2 Hz, 2H), 2.53 – 2.47 (m, 2H), 2.42 (s, 3H), 2.24 (s, 6H), 2.19 (s, 3H), 2.17 – 2.13 (m, 2H); ^{13}C NMR (100 MHz, CDCl$_3$) δ 194.0, 157.5, 145.5, 144.8, 142.7, 138.9, 136.8, 134.8, 134.3, 133.3, 133.0, 132.7, 131.8, 131.5, 129.9, 129.7, 128.5, 127.9, 127.6, 127.5, 127.0, 126.9, 126.7, 117.7, 92.4, 66.0, 51.1, 29.4, 26.3, 22.8, 21.6, 20.8; IR (neat): 2967 (bs), 2235 (s), 1687, 1637, 1367, 1187, 970, 845, 813; HRESIMS Calcd for $[C_{40}H_{37}NNaO_6S_2]^+$ (M + Na$^+$) 714.1955, found 714.1952.

(*E*)-3-methoxy-1-(((4-methyl-*N*-(6-oxohex-4-en-1-yl)phenyl)sulfonamido)ethynyl)naphthalen-2-yl 2,4,6-trimethylbenzenesulfonate (1q)

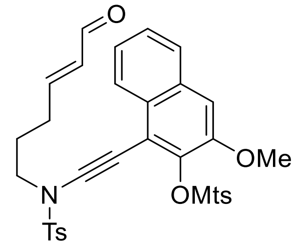

1q

Compound **1q** was prepared in 45% yield (289 mg) with *E*/*Z* = 4/1 according to the general procedure as a pale yellow oil. ^{1}H NMR (400 MHz, CDCl$_3$) δ 9.49 (d, *J* = 8.0 Hz, 1H), 8.29 – 8.25 (m, 1H), 7.92 – 7.89 (m, 2H), 7.68 – 7.65 (m, 1H), 7.50 – 7.46 (m, 2H), 7.35 (d, *J* = 8.0 Hz, 2H), 7.00 – 6.87 (m, 4H), 6.13 (dd, *J* = 15.6, 7.6 Hz, 1H), 3.56 (t, *J* = 7.2 Hz, 2H), 3.37 (s, 3H), 2.55 (s, 6H), 2.52 – 2.46 (m, 2H), 2.44 (s, 3H), 2.33 (s,

3H), 2.18 – 2.10 (m, 2H); ^{13}C NMR (100 MHz, $CDCl_3$) δ 194.1, 157.4, 150.2, 145.0, 142.9, 139.7, 139.5, 134.7, 134.1, 133.3, 132.2, 131.2, 130.0, 128.4, 127.6, 127.1, 126.6, 126.5, 125.2, 117.7, 106.7, 92.4, 65.4, 55.1, 51.1, 29.4, 26.2, 22.7, 21.6, 21.0; IR (neat): 2926 (bs), 2235 (s), 1627, 1360, 1168, 794, 741, 683; HRESIMS Calcd for $[C_{35}H_{35}NNaO_7S_2]^+$ (M + Na^+) 668.1747, found 668.1754.

(*E*)-6-bromo-1-(((4-methyl-*N*-(6-oxohex-4-en-1-yl)phenyl)sulfonamido)ethynyl)naphthalen-2-yl 2,4,6-trimethylbenzenesulfonate (1r)

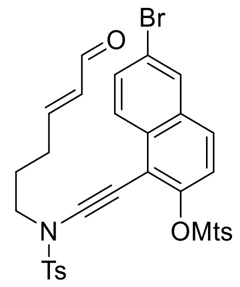

1r

Compound **1r** was prepared in 50% yield (347 mg) with *E*/*Z* = 16/1 according to the general procedure as a pale yellow oil. ^{1}H NMR (400 MHz, $CDCl_3$) δ 9.49 (d, *J* = 8.0 Hz, 1H), 8.21 (d, *J* = 9.2 Hz, 1H), 7.92 – 7.88 (m, 3H), 7.67 (dd, *J* = 8.8, 2.0 Hz, 1H), 7.44 (d, *J* = 9.2 Hz, 1H), 7.35 (d, *J* = 8.0 Hz, 2H), 6.99 (s, 2H), 6.93 – 6.86 (m, 1H), 6.61 (d, *J* = 9.2 Hz, 1H), 6.16 – 6.09 (m, 1H), 3.55 (t, *J* = 7.2 Hz, 2H), 2.53 (s, 6H), 2.50 – 2.47 (m, 2H), 2.44 (s, 3H), 2.34 (s, 3H), 2.13 – 2.06 (m, 2H); ^{13}C NMR (100 MHz, $CDCl_3$) δ 193.9, 157.0, 147.3, 145.1, 144.0, 140.1, 134.5, 133.4, 132.3, 132.1, 131.9, 131.6, 130.9, 130.0, 129.9, 128.4, 127.5, 127.3, 120.9, 120.3, 115.8, 92.7, 65.1, 50.9, 29.3, 26.0, 22.7, 21.6, 21.1; IR (neat): 2941 (bs), 2235 (s), 1694, 1597, 1367, 1169, 1091, 972, 819, 672; HRESIMS Calcd for $[C_{34}H_{32}BrNNaO_6S_2]^+$ (M + Na^+) 716.0747, found 716.0748.

(*E*)-1-(((4-methyl-*N*-(6-oxohex-4-en-1-yl)phenyl)sulfonamido)ethynyl)-6-phenylnaphthalen-2-yl 2,4,6-trimethylbenzenesulfonate (1s)

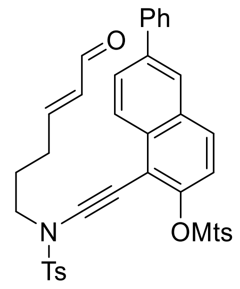

1s

Compound **1s** was prepared in 58% yield (402 mg) with *E*/*Z* = 17/1 according to the general procedure as a pale yellow oil. ^{1}H NMR (400 MHz, $CDCl_3$) δ 9.50 (d, *J* = 7.6 Hz, 1H), 8.42 (d, *J* = 8.8 Hz, 1H), 7.96 – 7.90 (m, 4H), 7.70 (d, *J* = 7.6 Hz, 2H), 7.59 (d, *J* = 8.8 Hz, 1H), 7.50 – 7.47 (m, 2H), 7.41 – 7.35 (m, 3H), 7.00 (s, 2H), 6.95 – 6.87 (m, 1H), 6.61 (d, *J* = 8.8 Hz, 1H), 6.14 (dd, *J* = 15.6, 7.6 Hz, 1H), 3.56 (t, *J* = 6.8 Hz, 2H), 2.55 (s, 6H), 2.52 – 2.47 (m, 2H), 2.44 (s, 3H), 2.35 (s, 3H), 2.19 – 2.04 (m, 2H); ^{13}C NMR (100 MHz, $CDCl_3$) δ 194.0, 157.2, 147.2, 145.0, 143.9, 140.3, 140.1, 139.4, 134.6, 133.4, 132.8, 131.9, 131.6, 130.0, 128.9, 128.6, 127.7, 127.6, 127.3(4), 127.3(0), 127.2, 125.7, 119.6, 115.4, 92.2, 65.4, 50.9, 29.4, 26.1, 22.7, 21.6, 21.1; IR (neat): 3024 (bs), 2236 (s), 1683, 1597, 1167, 1090, 950, 890, 766; HRESIMS Calcd for $[C_{40}H_{37}NNaO_6S_2]^+$ (M + Na^+) 714.1955, found 714.1954.

(*E*)-6-methyl-1-(((4-methyl-*N*-(6-oxohex-4-en-1-yl)phenyl)sulfonamido)ethynyl)naphthalen-2-yl 2,4,6-trimethylbenzenesulfonate (1t)

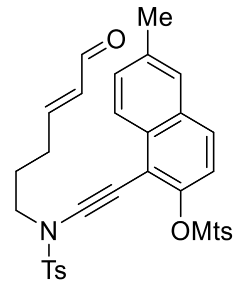

1t

Compound **1t** was prepared in 55% yield (345 mg) with *E*/*Z* > 20/1 according to the general procedure as a pale yellow oil. ^{1}H NMR (400 MHz, $CDCl_3$) δ 9.49 (d, *J* = 8.0 Hz, 1H), 8.23 (d, *J* = 8.4 Hz, 1H), 7.93 – 7.88 (m, 2H), 7.53 (s, 1H), 7.46 – 7.44 (m,

2H), 7.35 (d, J = 8.0 Hz, 2H), 6.99 (s, 2H), 6.94 – 6.87 (m, 1H), 6.54 (d, J = 9.2 Hz, 1H), 6.17 – 6.10 (m, 1H), 3.55 (t, J = 7.2 Hz, 2H), 2.53 (s, 6H), 2.50 (s, 3H), 2.54 – 2.46 (m, 2H), 2.44 (s, 3H), 2.35 (s, 3H), 2.14 – 2.07 (m, 2H); ^{13}C NMR (100 MHz, $CDCl_3$) δ 194.1, 157.2, 146.7, 144.9, 143.8, 140.2, 136.6, 134.6, 133.4, 131.8, 131.6, 130.0, 129.9, 127.8, 127.6, 127.0, 126.4, 119.1, 115.2, 91.9, 65.5, 51.0, 29.4, 26.1, 22.7, 21.6, 21.5, 21.1; IR (neat): 2940 (bs), 2236 (s), 1688, 1596, 1366, 1169, 893, 737, 546; HRESIMS Calcd for $[C_{35}H_{35}NNaO_6S_2]^+$ (M + Na^+) 652.1798, found 652.1805.

(*E*)-6-methoxy-1-(((4-methyl-*N*-(6-oxohex-4-en-1-yl)phenyl)sulfonamido)ethynyl)naphthalen-2-yl 2,4,6-trimethylbenzenesulfonate (1u)

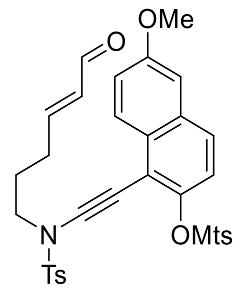

1u

Compound **1u** was prepared in 57% yield (370 mg) with E/Z = 11/1 according to the general procedure as a pale yellow oil. ^{1}H NMR (400 MHz, $CDCl_3$) δ 9.49 (d, J = 8.0 Hz, 1H), 8.22 (d, J = 9.2 Hz, 1H), 7.90 (d, J = 8.0 Hz, 2H), 7.42 (d, J = 9.2 Hz, 1H), 7.35 (d, J = 8.0 Hz, 2H), 7.28 (d, J = 2.4 Hz, 1H), 7.05 (d, J = 2.4 Hz, 1H), 6.99 (s, 2H), 6.94 – 6.87 (m, 1H), 6.53 (d, J = 9.2 Hz, 1H), 6.13 (dd, J = 15.6, 8.0 Hz, 1H), 3.90 (s, 3H), 3.55 (t, J = 6.8 Hz, 2H), 2.53 (s, 6H), 2.51 – 2.46 (m, 2H), 2.44 (s, 3H), 2.35 (s, 3H), 2.20 – 2.04 (m, 2H); ^{13}C NMR (100 MHz, $CDCl_3$) δ 194.1, 158.2, 157.2, 145.7, 144.9, 143.8, 140.1, 134.6, 133.4, 132.7, 131.8, 130.0, 128.9, 128.1, 127.6, 127.0, 120.3, 119.6, 115.4, 106.0, 91.8, 65.5, 55.3, 50.9, 29.4, 26.0, 22.7, 21.6, 21.1; IR (neat): 2944 (bs), 2236 (s), 1688, 1593, 1366, 1166, 897, 772, 552; HRESIMS Calcd for $[C_{35}H_{35}NNaO_7S_2]^+$ (M + Na^+) 668.1747, found 668.1743.

(*E*)-7-bromo-1-(((4-methyl-*N*-(6-oxohex-4-en-1-

yl)phenyl)sulfonamido)ethynyl)naphthalen-2-yl 2,4,6-trimethylbenzenesulfonate (1v)

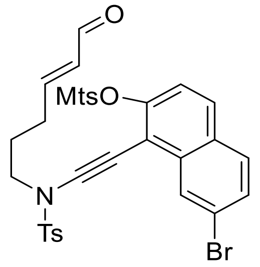

1v

Compound **1v** was prepared in 52% yield (360 mg) with *E*/*Z* > 20/1 according to the general procedure as a pale yellow oil. ^{1}H NMR (400 MHz, $CDCl_3$) δ 9.50 (d, *J* = 8.0 Hz, 1H), 8.47 (d, *J* = 1.6 Hz, 1H), 7.93 (d, *J* = 8.4 Hz, 2H), 7.63 (d, *J* = 8.8 Hz, 1H), 7.57 (dd, *J* = 8.8, 2.0 Hz, 1H), 7.51 (d, *J* = 8.8 Hz, 1H), 7.41 (d, *J* = 8.0 Hz, 2H), 7.00 (s, 2H), 6.95 – 6.87 (m, 1H), 6.63 (d, *J* = 9.2 Hz, 1H), 6.17 – 6.11 (m, 1H), 3.58 (t, *J* = 7.0 Hz, 2H), 2.54 (s, 6H), 2.59 – 2.47 (m, 2H), 2.45 (s, 3H), 2.35 (s, 3H), 2.14 – 2.07 (m, 2H); ^{13}C NMR (100 MHz, $CDCl_3$) δ 194.0, 157.1, 148.0, 145.1, 144.1, 140.1, 134.7, 134.4, 133.4, 131.9, 131.6, 130.1, 130.0, 129.7, 129.6, 128.8, 128.4, 127.5, 122.2, 119.7, 114.7, 92.6, 64.8, 51.0, 29.4, 26.1, 22.7, 21.7, 21.1; IR (neat): 2940 (bs), 2235 (s), 1690, 1598, 1367, 1171, 1090, 951, 819, 672; HRESIMS Calcd for $[C_{34}H_{32}BrNNaO_6S_2]^+$ (M + Na^+) 716.0747, found 716.0741.

(*E*)-1-(((4-methyl-*N*-(6-oxohex-4-en-1-yl)phenyl)sulfonamido)ethynyl)-7-phenylnaphthalen-2-yl 2,4,6-trimethylbenzenesulfonate (1w)

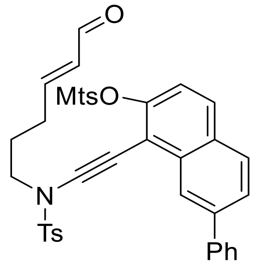

1w

Compound **1w** was prepared in 66% yield (458 mg) with *E*/*Z* > 20/1 according to the general procedure as a pale yellow oil. ^{1}H NMR (400 MHz, $CDCl_3$) δ 9.46 (d, *J* = 7.6

Hz, 1H), 8.66 (s, 1H), 7.88 – 7.79 (m, 6H), 7.57 – 7.48 (m, 3H), 7.41 – 7.38 (m, 1H), 7.21 (d, *J* = 7.6 Hz, 2H), 7.00 (s, 2H), 6.91 – 6.84 (m, 1H), 6.59 (d, *J* = 8.8 Hz, 1H), 6.11 (dd, *J* = 15.6, 7.6 Hz, 1H), 3.55 (t, *J* = 6.8 Hz, 2H), 2.56 (s, 6H), 2.51 – 2.45 (m, 2H), 2.36 (s, 3H), 2.35 (s, 3H), 2.13 – 2.09 (m, 2H); ^{13}C NMR (100 MHz, $CDCl_3$) δ 194.1, 157.2, 147.6, 144.9, 143.9, 140.3, 140.2, 134.6, 134.0, 133.4, 131.9, 130.5, 130.0, 129.0, 128.6, 128.1, 127.8, 127.4(9), 127.4(6), 126.2, 124.4, 119.1, 115.7, 92.6, 65.6, 50.9, 29.4, 26.1, 22.8, 21.6, 21.1; IR (neat): 2941 (bs), 2235 (s), 1682, 1361, 1170, 1091, 950, 732; HRESIMS Calcd for $[C_{40}H_{37}NNaO_6S_2]^+$ (M + Na^+) 714.1955, found 714.1952.

(*E*)-7-methyl-1-(((4-methyl-*N*-(6-oxohex-4-en-1-yl)phenyl)sulfonamido)ethynyl)naphthalen-2-yl 2,4,6-trimethylbenzenesulfonate (1x)

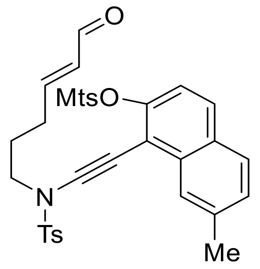

1x

Compound **1x** was prepared in 55% yield (344 mg) with *E*/*Z* > 20/1 according to the general procedure as a pale yellow oil. ^{1}H NMR (400 MHz, $CDCl_3$) δ 9.49 (d, *J* = 8.0 Hz, 1H), 8.16 (d, *J* = 0.8 Hz, 1H), 7.93 – 7.90 (m, 2H), 7.66 (d, *J* = 9.2 Hz, 1H), 7.49 (d, *J* = 8.8 Hz, 1H), 7.37 – 7.32 (m, 3H), 6.98 (s, 2H), 6.94 – 6.87 (m, 1H), 6.52 (d, *J* = 8.8 Hz, 1H), 6.12 (dd, *J* = 15.6, 8.0 Hz, 1H), 3.56 (t, *J* = 6.8 Hz, 2H), 2.57 (s, 3H), 2.53 (s, 6H), 2.50 – 2.46 (m, 2H), 2.43 (s, 3H), 2.34 (s, 3H), 2.14 – 2.07 (m, 2H); ^{13}C NMR (100 MHz, $CDCl_3$) δ 194.0, 157.2, 147.5, 144.9, 143.8, 140.1, 137.7, 134.7, 133.9, 133.4, 131.9, 131.8, 130.0, 129.6, 128.9, 128.2, 127.8, 127.5, 125.7, 118.1, 114.6, 92.0, 65.4, 50.9, 29.4, 26.1, 22.7, 22.0, 21.6, 21.1; IR (neat): 2931 (bs), 2224 (s), 1691, 1421, 1214, 1170, 1140, 937, 765, 750; HRESIMS Calcd for $[C_{35}H_{35}NNaO_6S_2]^+$ (M + Na^+) 652.1798, found 652.1793.

(*E*)-7-methoxy-1-(((4-methyl-*N*-(6-oxohex-4-en-1-yl)phenyl)sulfonamido)ethynyl)naphthalen-2-yl 2,4,6-trimethylbenzenesulfonate (1y)

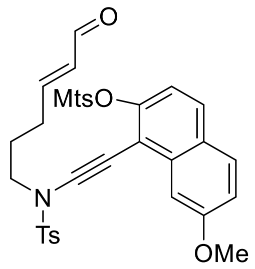

1y

Compound **1y** was prepared in 46% yield (297 mg) with *E*/*Z* > 20/1 according to the general procedure as a pale yellow oil. ^{1}H NMR (400 MHz, $CDCl_3$) δ 9.49 (d, *J* = 8.0 Hz, 1H), 7.91 – 7.87 (m, 3H), 7.66 (d, *J* = 8.8 Hz, 1H), 7.45 (d, *J* = 8.8 Hz, 1H), 7.33 (d, *J* = 8.0 Hz, 2H), 7.17 (dd, *J* = 8.8, 2.4 Hz, 1H), 6.99 (s, 2H), 6.95 – 6.87 (m, 1H), 6.39 (d, *J* = 8.8 Hz, 1H), 6.12 (dd, *J* = 15.6, 8.0 Hz, 1H), 4.03 (s, 3H), 3.50 (t, *J* = 6.8 Hz, 2H), 2.53 (s, 6H), 2.51 – 2.46 (m, 2H), 2.42 (s, 3H), 2.34 (s, 3H), 2.15 – 2.08 (m, 2H); ^{13}C NMR (100 MHz, $CDCl_3$) δ 194.0, 159.4, 157.2, 147.2, 145.0, 143.8, 140.1, 135.4, 134.6, 133.4, 131.8, 130.0, 129.4, 127.9, 127.3, 126.7, 119.9, 116.4, 114.3, 104.7, 92.1, 66.1, 55.8, 50.6, 29.4, 25.9, 22.7, 21.6, 21.1; IR (neat): 2940 (bs), 2236 (s), 1630, 1361, 1221, 1168, 955, 783, 742, 582; HRESIMS Calcd for $[C_{35}H_{35}NNaO_7S_2]^+$ (M + Na^+) 668.1747, found 668.1744.

(*E*)-1-(((4-methyl-*N*-(6-oxohex-4-en-1-yl)phenyl)sulfonamido)ethynyl)-5,6,7,8-tetrahydronaphthalen-2-yl 2,4,6-trimethylbenzenesulfonate (1z)

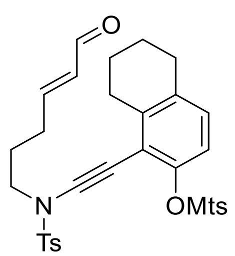

1z

Compound **1z** was prepared in 47% yield (289 mg) with *E*/*Z* = 11/1 according to the

general procedure as a pale yellow oil. ^{1}H NMR (400 MHz, $CDCl_3$) δ 9.49 (d, *J* = 8.0 Hz, 1H), 7.87 (d, *J* = 8.4 Hz, 2H), 7.36 (d, *J* = 8.0 Hz, 2H), 6.98 (s, 2H), 6.94 – 6.86 (m, 1H), 6.74 (d, *J* = 8.4 Hz, 1H), 6.17 – 6.09 (m, 2H), 3.51 (t, *J* = 7.2 Hz, 2H), 2.83 (t, *J* = 6.0 Hz, 2H), 2.66 (t, *J* = 6.0 Hz, 2H), 2.54 (s, 6H), 2.49 – 2.42 (m, 1H), 2.45 (s, 3H), 2.33 (s, 3H), 2.07 – 2.02 (m, 2H), 1.84 – 1.72 (m, 5H); ^{13}C NMR (100 MHz, $CDCl_3$) δ 194.0, 157.4, 147.2, 144.8, 143.6, 140.4, 140.0, 135.9, 134.6, 133.3, 131.9, 131.7, 129.9, 128.4, 127.5, 118.1, 117.1, 91.5, 65.4, 51.0, 29.4, 29.2, 28.4, 26.0, 22.6, 22.5, 22.4, 21.6, 21.0; IR (neat): 2943 (bs), 2235 (s), 1688, 1371, 1269, 1095, 951, 757, 559; HRESIMS Calcd for $[C_{34}H_{37}NNaO_6S_2]^+$ (M + Na^+) 642.1955, found 642.1959.

(*E*)-1-(((4-(*tert*-butyl)-*N*-(6-oxohex-4-en-1-yl)phenyl)sulfonamido)ethynyl)naphthalen-2-yl trifluoromethanesulfonate (1aa)

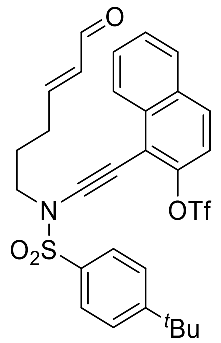

1aa

Compound **1aa** was prepared in 67% yield (405 mg) with *E*/*Z* = 18/1 according to the general procedure as a pale yellow oil. ^{1}H NMR (400 MHz, $CDCl_3$) δ 9.50 (d, *J* = 7.6 Hz, 1H), 8.40 (d, *J* = 8.4 Hz, 1H), 7.93 – 7.86 (m, 3H), 7.82 (d, *J* = 9.2 Hz, 1H), 7.71 – 7.67 (m, 1H), 7.62 – 7.55 (m, 3H), 7.32 (d, *J* = 9.2 Hz, 1H), 6.88 – 6.81 (m, 1H), 6.14 (dd, *J* = 15.6, 7.6 Hz, 1H), 3.59 (t, *J* = 7.2 Hz, 2H), 2.51 – 2.45 (m, 2H), 2.15 – 1.99 (m, 2H), 1.33 (s, 9H); ^{13}C NMR (100 MHz, $CDCl_3$) δ 193.8, 158.1, 156.5, 147.1, 134.3, 133.6, 133.5, 132.0, 129.5, 128.4, 128.2, 127.6, 127.4, 126.7, 126.4, 118.8, 118.6 (q, *J* = 320.4 Hz), 115.2, 94.0, 63.8, 51.0, 35.3, 30.9, 29.2, 26.3; ^{19}F NMR (376 MHz, $CDCl_3$) δ -73.7; IR (neat): 2966 (bs), 2234 (s), 1693, 1175, 1140, 948, 835, 523; HRESIMS Calcd for $[C_{29}H_{28}F_3NNaO_6S_2]^+$ (M + Na^+) 630.1202, found 630.1206.

(*E*)-1-(((4-nitro-*N*-(6-oxohex-4-en-1-yl)phenyl)sulfonamido)ethynyl)naphthalen-2-yl trifluoromethanesulfonate (1ab)

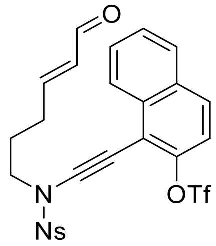

1ab

Compound **1ab** was prepared in 61% yield (362 mg) with *E*/*Z* > 20/1 according to the general procedure as a pale yellow oil. ^{1}H NMR (400 MHz, $CDCl_3$) δ 9.51 (d, *J* = 7.6 Hz, 1H), 8.38 (d, *J* = 8.8 Hz, 2H), 8.32 (d, *J* = 8.4 Hz, 1H), 8.17 (d, *J* = 8.8 Hz, 2H), 7.91 – 7.87 (m, 2H), 7.73 – 7.60 (m, 2H), 7.32 (d, *J* = 9.2 Hz, 1H), 6.89 – 6.82 (m, 1H), 6.16 (dd, *J* = 15.6, 7.6 Hz, 1H), 3.64 (t, *J* = 7.2 Hz, 2H), 2.55 – 2.48 (m, 2H), 2.12 – 2.05 (m, 2H); ^{13}C NMR (100 MHz, $CDCl_3$) δ 193.6, 155.8, 150.8, 147.7, 142.5, 133.6, 133.5, 132.0, 130.4, 128.8, 128.6, 128.4, 127.7, 126.3, 124.6, 118.8, 118.5 (q, *J* = 320.6 Hz), 114.4, 92.2, 64.0, 51.4, 29.1, 26.3; ^{19}F NMR (376 MHz, $CDCl_3$) δ -73.9; IR (neat):2925 (bs), 2235 (s), 1689, 1598, 1373, 1175, 1093, 962, 819, 650; HRESIMS Calcd for $[C_{25}H_{19}F_3N_2NaO_8S_2]^+$ (M + Na$^+$) 619.0427, found 619.0429.

(*E*)-8-methyl-1-(((4-methyl-*N*-(6-oxohex-4-en-1-yl)phenyl)sulfonamido)ethynyl)naphthalen-2-yl-trifluoromethanesulfonate (1ac)

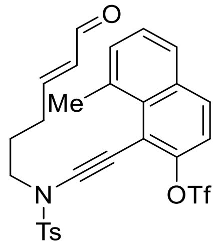

1ac

Compound **1ac** was prepared in 57% yield (322 mg) with *E*/*Z* > 20/1 according to the general procedure as a pale yellow oil. ^{1}H NMR (400 MHz, $CDCl_3$) δ 9.49 (d, *J* = 7.6 Hz, 1H), 7.84 – 7.79 (m, 3H), 7.70 (d, *J* = 7.6 Hz, 1H), 7.45 – 7.26 (m, 5H), 6.88 – 6.80 (m, 1H), 6.13 (dd, *J* = 15.6, 7.6 Hz, 1H), 3.57 (t, *J* = 6.8 Hz, 2H), 3.15 (s, 3H), 2.50 – 2.47 (m, 2H), 2.43 (s, 3H), 2.04 – 2.00 (m, 2H); ^{13}C NMR (100 MHz, $CDCl_3$) δ 193.8,

156.6, 150.0, 145.0, 137.0, 134.6, 133.5, 132.7, 131.1, 130.9, 130.0, 127.5, 127.3, 127.2, 118.5 (q, J = 319.8 Hz), 118.2, 114.5, 94.5, 66.4, 50.8, 29.2, 26.4, 24.1, 21.6; ^{19}F NMR (376 MHz, $CDCl_3$) δ -73.9; IR (neat): 2931 (bs), 2224 (s), 1691, 1421, 1214, 1140, 937, 765, 750, 546; HRESIMS Calcd for $[C_{27}H_{24}F_3NNaO_6S_2]^+$ (M + Na^+) 602.0889, found 602.0896.

(*E*)-1-(((4-methyl-*N*-(6-oxohex-4-en-1-yl)phenyl)sulfonamido)ethynyl)-8-phenylnaphthalen-2-yl trifluoromethanesulfonate (1ad)

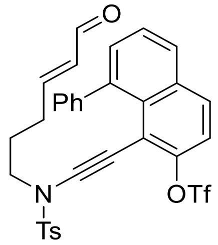

1ad

Compound **1ad** was prepared in 47% yield (299 mg) with E/Z = 13/1 according to the general procedure as a pale yellow oil. ^{1}H NMR (400 MHz, $CDCl_3$) δ 9.47 (d, J = 8.0 Hz, 1H), 7.97 (d, J = 8.8 Hz, 1H), 7.90 (d, J = 8.0 Hz, 1H), 7.68 (d, J = 8.0 Hz, 2H), 7.59 – 7.55 (m, 1H), 7.41 – 7.39 (m, 2H), 7.33 – 7.28 (m, 5H), 7.17 – 7.14 (m, 2H), 6.78 – 6.70 (m, 1H), 6.06 (dd, J = 15.6, 7.6 Hz, 1H), 2.92 (t, J = 6.8 Hz, 2H), 2.46 (s, 3H), 2.29 – 2.23 (m, 2H), 1.59 – 1.53 (m, 2H); ^{13}C NMR (100 MHz, $CDCl_3$) δ 193.8, 156.8, 151.8, 144.6, 142.2, 141.2, 134.9, 133.4, 133.3, 132.1, 131.4, 129.8, 129.7, 128.5, 127.8, 127.3, 126.7, 126.5, 119.0, 118.6 (q, J = 322.1 Hz), 114.8, 110.0, 96.8, 63.6, 50.4, 29.0, 25.9, 21.6; ^{19}F NMR (376 MHz, $CDCl_3$) δ -73.8; IR (neat): 2955 (bs), 2228 (s), 1690, 1423, 1214, 1171, 1141, 840, 564; HRESIMS Calcd for $[C_{32}H_{26}F_3NNaO_6S_2]^+$ (M + Na^+) 664.1046, found 664.1041.

(*E*)-10-(((4-methyl-N-(6-oxohex-4-en-1-yl)phenyl)sulfonamido)ethynyl)phenanthren-9-yl trifluoromethanesulfonate (1ae)

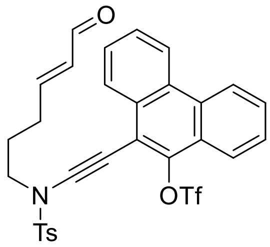

1ae

Compound **1ae** was prepared in 64% yield (392 mg) with *E*/*Z* = 10/1 according to the general procedure as a pale yellow oil. ^{1}H NMR (400 MHz, $CDCl_3$) δ 9.50 (d, *J* = 7.6 Hz, 1H), 8.69 – 8.66 (m, 2H), 8.52 – 8.50 (m, 1H), 8.01 (d, *J* = 8.0 Hz, 1H), 7.88 (d, *J* = 8.0 Hz, 2H), 7.78 – 7.67 (m, 4H), 7.34 (d, *J* = 8.0 Hz, 2H), 6.89 – 6.82 (m, 1H), 6.15 (dd, *J* = 15.6, 7.6 Hz, 1H), 3.60 (t, *J* = 7.2 Hz, 2H), 2.53 – 2.46 (m, 2H), 2.43 (s, 3H), 2.11 – 2.03 (m, 2H); ^{13}C NMR (100 MHz, $CDCl_3$) δ 193.8, 156.5, 145.2, 144.4, 134.5, 133.6, 131.0, 130.5, 130.1, 129.1, 128.4, 128.3, 128.2, 127.8, 127.7, 127.6, 124.8, 123.1, 122.8, 121.4, 118.5 (q, *J* = 320.7 Hz), 114.5, 94.3, 64.7, 50.9, 29.3, 26.4, 21.6; ^{19}F NMR (376 MHz, $CDCl_3$) δ -72.9; IR (neat): 2926 (bs), 2232 (s), 1959, 1691, 1420, 1212, 1170, 818, 750, 579; HRESIMS Calcd for $[C_{30}H_{24}F_3NNaO_6S_2]^+$ (M + Na^+) 638.0889, found 638.0881.

(*E*)-1-(((4-methyl-*N*-(6-oxohex-4-en-1-yl)phenyl)sulfonamido)ethynyl)-5,6,7,8-tetrahydronaphthalen-2-yl trifluoromethanesulfonate (1af)

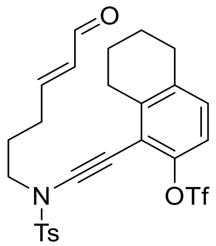

1af

Compound **1af** was prepared in 59% yield (340 mg) with *E*/*Z* = 14/1 according to the general procedure as a pale yellow oil. ^{1}H NMR (400 MHz, $CDCl_3$) δ 9.50 (d, *J* = 7.6 Hz, 1H), 7.83 (d, *J* = 8.4 Hz, 2H), 7.35 (d, *J* = 8.0 Hz, 2H), 7.03 (d, *J* = 8.8 Hz, 1H), 6.95 (d, *J* = 8.8 Hz, 1H), 6.87 – 6.79 (m, 1H), 6.12 (dd, *J* = 15.6, 7.6 Hz, 1H), 3.51 (t, *J* = 6.8 Hz, 2H), 3.26 – 3.21 (m, 1H), 2.85 (t, *J* = 6.0 Hz, 2H), 2.75 (t, *J* = 6.0 Hz, 2H), 2.45 (s, 3H), 2.43 – 2.41 (m, 1H), 2.02 – 1.95 (m, 2H), 1.87 – 1.72 (m, 4H); ^{13}C NMR

(100 MHz, $CDCl_3$) δ 193.8, 156.5, 147.3, 145.1, 141.5, 137.8, 134.6, 133.5, 130.0, 129.6, 129.3, 127.5, 121.7 (q, J = 299.7 Hz), 117.6, 93.0, 63.9, 51.0, 47.9, 29.4, 28.5, 26.3, 25.2, 22.4, 21.6; ^{19}F NMR (376 MHz, $CDCl_3$) δ -73.8; IR (neat): 2944 (bs), 2234 (s), 1689, 1422, 1170, 1140, 947, 705, 545; HRESIMS Calcd for $[C_{26}H_{26}F_3NNaO_6S_2]^+$ (M + Na^+) 592.1046, found 592.1047.

(*E*)-*N*-((2-iodonaphthalen-1-yl)ethynyl)-4-methyl-*N*-(6-oxohex-4-en-1-yl)benzenesulfonamide (1ag)

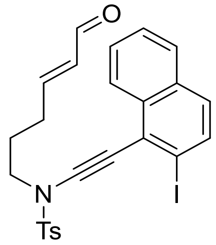

1ag

Compound **1ag** was prepared in 44% yield (241 mg) with $E/Z > 20/1$ according to the general procedure as a pale yellow oil. ^1H NMR (400 MHz, $CDCl_3$) δ 9.50 (d, J = 7.6 Hz, 1H), 8.29 (d, J = 8.4 Hz, 1H), 7.92 (d, J = 8.4 Hz, 2H), 7.81 – 7.77 (m, 2H), 7.58 – 7.50 (m, 2H), 7.45 (d, J = 8.8 Hz, 1H), 7.34 (d, J = 8.0 Hz, 2H), 6.89 – 6.81 (m, 1H), 6.14 (dd, J = 15.6, 7.6 Hz, 1H), 3.60 (t, J = 6.8 Hz, 2H), 2.54 – 2.46 (m, 2H), 2.43 (s, 3H), 2.16 – 2.09 (m, 2H); ^{13}C NMR (100 MHz, $CDCl_3$) δ 193.7, 156.3, 145.1, 134.8, 134.4, 133.9, 133.5, 132.1, 130.0, 128.6, 128.2, 127.7, 127.6, 127.1, 126.8, 126.5, 98.1, 90.6, 72.72, 50.9, 29.3, 26.2, 21.6; IR (neat): 2934 (bs), 2228 (s), 1686, 1363, 1169, 812, 737, 556; HRESIMS Calcd for $[C_{25}H_{22}INNaO_3S]^+$ (M + Na^+) 566.0257, found 566.0259.

(*E*)-*N*-((2-isopropylnaphthalen-1-yl)ethynyl)-4-methyl-*N*-(6-oxohex-4-en-1-yl)benzenesulfonamide (1ah)

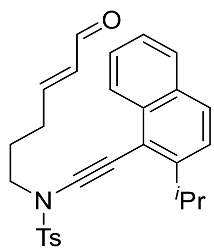

1ah

Compound **1ah** was prepared in 52% yield (237 mg) with *E*/*Z* > 20/1 according to the general procedure as a pale yellow oil. ^{1}H NMR (400 MHz, $CDCl_3$) δ 9.51 (d, *J* = 7.6 Hz, 1H), 8.19 (d, *J* = 8.4 Hz, 1H), 7.88 (d, *J* = 8.4 Hz, 2H), 7.80 – 7.76 (m, 2H), 7.52 – 7.41 (m, 3H), 7.34 (d, *J* = 8.0 Hz, 2H), 6.89 – 6.82 (m, 1H), 6.18 – 6.12 (m, 1H), 3.63 – 3.55 (m, 3H), 2.56 – 2.50 (m, 2H), 2.44 (s, 3H), 2.08 – 2.01 (m, 2H), 1.29 (d, *J* = 6.8 Hz, 6H); ^{13}C NMR (100 MHz, $CDCl_3$) δ 193.7, 156.3, 148.4, 144.9, 134.4, 133.5, 133.4, 131.6, 129.9, 128.3, 128.0, 127.6, 126.7, 125.9, 125.6, 123.1, 117.4, 90.3, 67.8, 50.9, 32.2, 29.2, 26.3, 23.2, 21.6; IR (neat): 2961 (bs), 2228 (s), 1690, 1363, 1186, 1135, 969, 819, 548; HRESIMS Calcd for $[C_{28}H_{29}NNaO_3S]^+$ (M + Na^+) 482.1760, found 482.1762.

(*E*)-4-methyl-*N*-(6-oxohex-4-en-1-yl)-*N*-((2-phenylnaphthalen-1-yl)ethynyl)benzenesulfonamide (1ai)

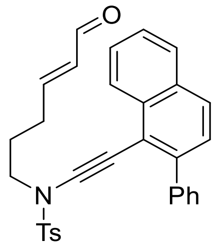

1ai

Compound **1ai** was prepared in 71% yield (305 mg) with *E*/*Z* > 20/1 according to the general procedure as a colorless oil. ^{1}H NMR (400 MHz, $CDCl_3$) δ 9.47 (d, *J* = 7.6 Hz, 1H), 8.37 (d, *J* = 8.4 Hz, 1H), 7.86 (d, *J* = 8.0 Hz, 1H), 7.81 (d, *J* = 8.4 Hz, 1H), 7.69 (d, *J* = 8.4 Hz, 2H), 7.62 – 7.51 (m, 4H), 7.46 (d, *J* = 8.4 Hz, 1H), 7.42 – 7.32 (m, 3H), 7.26 (d, *J* = 8.4 Hz, 2H), 6.72 – 6.64 (m, 1H), 6.06 – 5.98 (m, 1H), 3.34 (t, *J* = 6.8 Hz, 2H), 2.41 (s, 3H), 2.29 – 2.23 (m, 2H), 1.69 – 1.61 (m, 2H); ^{13}C NMR (100 MHz, $CDCl_3$) δ 193.7, 156.5, 144.8, 141.5, 141.2, 134.4, 133.3, 133.2, 132.2, 129.9, 129.6, 128.1, 128.0, 127.7, 127.5, 127.4, 127.3, 127.2, 126.5, 126.4, 118.1, 89.4, 69.3, 50.7,

29.1, 25.9, 21.6; IR (neat): 3056 (bs), 2228 (s), 1690, 1363, 1169, 1089, 765, 734, 703; HRESIMS Calcd for $[C_{31}H_{27}NNaO_3S]^+$ (M + Na^+) 516.1604, found 516.1607.

(*E*)-3-methoxy-2-(((4-methyl-*N*-(6-oxohex-4-en-1-yl)phenyl)sulfonamido)ethynyl)phenyl 2,4,6-trimethylbenzenesulfonate (1aj)

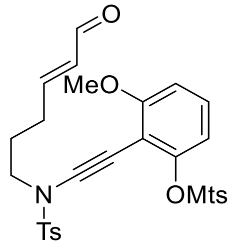

1aj

Compound **1aj** was prepared in 61% yield (365 mg) with *E*/*Z* > 20/1 according to the general procedure as a colorless oil. ^{1}H NMR (400 MHz, $CDCl_3$) 9.49 (d, *J* = 8.0 Hz, 1H), 7.95 (d, *J* = 8.0 Hz, 2H), 7.36 (d, *J* = 8.0 Hz, 2H), 7.03 – 6.99 (m, 3H), 6.92 – 6.85 (m, 1H), 6.74 (d, *J* = 8.4 Hz, 1H), 6.15 – 6.09 (m, 2H), 3.89 (s, 3H), 3.47 (t, *J* = 6.8 Hz, 2H), 2.56 (s, 6H), 2.51 – 1.43 (m, 2H), 2.45 (s, 3H), 2.34 (s, 3H), 2.03 – 1.99 (m, 2H); ^{13}C NMR (100 MHz, $CDCl_3$) δ 194.1, 161.0, 157.4, 150.4, 144.6, 143.8, 140.2, 134.6, 133.3, 131.8, 131.7, 129.8, 128.2, 127.8, 112.8, 108.8, 108.4, 90.9, 62.7, 56.2, 51.0, 29.4, 25.9, 22.7, 21.6, 21.1; IR (neat): 3052 (bs), 2241 (s), 1691, 1360, 1169, 1090, 763, 735; HRESIMS Calcd for $[C_{31}H_{33}NNaO_7S_2]^+$ (M + Na^+) 618.1591, found 618.1595.

4. General Procedures for the Intramolecular (4 + 2) Annulation

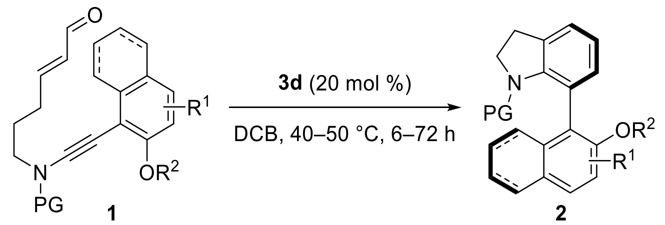

To a 10 mL vial charged with a stir bar were added ynamide **1** (0.10 mmol), 1,2-dichlorobenzene (2 mL) and chiral secondary amine catalyst **3d** (0.02 mmol, 10.2 mg) sequentially. The reaction mixture was stirred at 40–50 ºC for 6–72 h, and the progress of the reaction was monitored by TLC. Upon complection, the reaction mixture was directly purified by column chromatography on silica gel (eluent: PE/EtOAc) to afford the desired axially chiral 7-aryl indoline **2**.

(*S*)-1-(1-tosylindolin-7-yl)naphthalen-2-yl 2,4,6-trimethylbenzenesulfonate (2a)

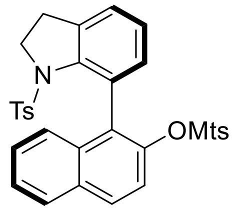

2a

Compound **2a** was prepared in 71% yield (42.4 mg) according to the general procedure at 50 ºC as a yellow solid (mp 104–106 ºC), $[\alpha]_D^{20}$ = +132.0º (c = 1.0, $CHCl_3$). 93% ee (determined by HPLC: Chiralpak IA Column, 30/70 ^{i}PrOH/hexane, 1.0 mL/min, 254 nm; TR = 7.54 min (major), 10.36 min (minor)). ^{1}H NMR (400 MHz, $CDCl_3$) δ 7.86 – 7.83 (m, 1H), 7.77 – 7.73 (m, 2H), 7.48 – 7.43 (m, 2H), 7.22 – 7.12 (m, 6H), 7.03 (d, *J* = 8.0 Hz, 2H), 6.89 (s, 2H), 4.07 – 4.01 (m, 1H), 3.82 – 3.74 (m, 1H), 2.48 – 2.40 (m, 1H), 2.42 (s, 6H), 2.35 – 2.32 (m, 1H), 2.33 (s, 3H), 2.30 (s, 3H); ^{13}C NMR (100 MHz, $CDCl_3$) δ 144.4, 143.4, 143.2, 141.9, 139.8, 137.6, 135.5, 132.9, 132.5, 131.9, 131.8, 131.6, 130.2, 129.1, 128.1, 127.7, 127.1, 126.5, 126.4, 126.0, 125.7, 124.5, 119.9, 52.3, 29.3, 22.6, 21.5, 21.0; IR (neat): 3020 (bs), 1635, 1485, 1470, 1322, 1160, 1104, 978, 561; HRESIMS Calcd for $[C_{34}H_{31}NNaO_5S_2]^+$ (M + Na^+) 620.1536, found 620.1550.

When the (4 + 2) annulation was carried out under standard conditions with **1a** (*E/Z* = 6/1), the desired product **2a** was obtained in 70% yield with 93% ee. This result is consistent with the experiment using (*E*)-**1a** as substrate.

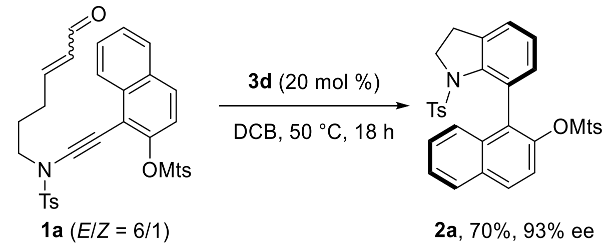

(*S*)-1-(1-((4-methoxyphenyl)sulfonyl)indolin-7-yl)naphthalen-2-yl 2,4,6-trimethylbenzenesulfonate (2b)

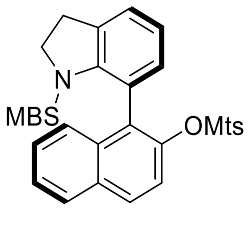

2b

Compound **2b** was prepared in 62% yield (48.8 mg) according to the general procedure at 50 ºC as a pale yellow oil. $[\alpha]_D^{20}$ = +242.5º (c = 1.0, $CHCl_3$). 90% ee (determined by HPLC: Chiralpak IA Column, 30/70 ^{i}PrOH/hexane, 1.0 mL/min, 254 nm; TR = 8.77 min (major), 12.22 min (minor)). ^{1}H NMR (400 MHz, $CDCl_3$) δ 7.85 – 7.83 (m, 1H), 7.78 – 7.73 (m, 2H), 7.48 – 7.43 (m, 2H), 7.31 – 7.27 (m, 2H), 7.23 – 7.11 (m, 4H), 6.89 (s, 2 H), 6.73 – 6.69 (m, 2H), 4.05 – 3.97 (m, 1H), 3.88 – 3.77 (m, 1H), 3.79 (s, 3H), 2.54 – 2.46 (m, 1H), 2.43 (s, 6H), 2.38 – 2.33 (m, 1H), 2.31 (s, 3H); ^{13}C NMR (100 MHz, $CDCl_3$) δ 162.9, 144.4, 143.2, 142.0, 139.8, 137.7, 132.9, 132.5, 132.0, 131.9, 131.6, 130.2, 130.0, 129.2, 129.1, 128.1, 127.8, 126.5, 126.4, 126.1, 125.7, 124.5, 119.9, 113.7, 55.5, 52.2, 29.3, 22.6, 21.0; IR (neat): 2940 (bs), 1636, 1345, 1234, 1171, 1009, 777, 613, 572; HRESIMS Calcd for $[C_{34}H_{31}NNaO_6S_2]^+$ (M + Na^+) 636.1485, found 636.1488.

(*S*)-1-(1-(mesitylsulfonyl)indolin-7-yl)naphthalen-2-yl 2,4,6-trimethylbenzenesulfonate (2c)

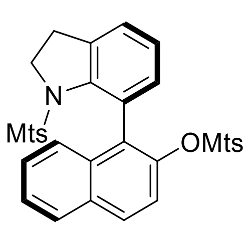

2c

Compound **2c** was prepared in 45% yield (48.8 mg) according to the general procedure at 50 °C as a pale yellow oil. $[\alpha]_D^{20}$ = +164.1° (c = 1.0, $CHCl_3$). 94% ee (determined by HPLC: Chiralpak IA Column, 30/70 ^{i}PrOH/hexane, 1.0 mL/min, 254 nm; TR = 5.61 min (major), 7.27 min (minor)). ^{1}H NMR (400 MHz, $CDCl_3$) δ 7.79 (d, *J* = 8.4 Hz, 1H), 7.73 (d, *J* = 8.0 Hz, 1H), 7.48 – 7.37 (m, 3H), 7.27 (d, *J* = 9.2 Hz, 1H), 7.03 – 6.99 (m, 1H), 6.94 (d, *J* = 9.2 Hz, 1H), 6.80 – 6.78 (m, 3H), 6.45 (s, 2H), 4.48 – 4.42 (m, 1H), 3.67 – 3.59 (m, 1H), 3.36 – 3.27 (m, 1H), 2.76 (dd, *J* = 15.2, 7.0 Hz, 1H), 2.29 (s, 3H), 2.25 (s, 6H), 2.15 (s, 3H), 2.00 (s, 6H); ^{13}C NMR (100 MHz, $CDCl_3$) δ 143.9, 143.3, 143.0, 141.2, 140.1, 138.7, 138.2, 134.8, 132.7, 132.0, 131.6, 131.4, 129.1, 128.8, 127.6, 127.5, 126.4, 126.3, 125.9, 125.4, 124.5, 120.2, 52.9, 30.8, 22.7, 22.1, 21.0, 20.8; IR (neat): 2944 (bs), 1615, 1494, 1361, 1265, 1168, 830, 578; HRESIMS Calcd for $[C_{36}H_{35}NNaO_5S_2]^+$ (M + Na^+) 648.1849, found 648.1853.

(*S*)-1-(1-(phenylsulfonyl)indolin-7-yl)naphthalen-2-yl 2,4,6-trimethylbenzenesulfonate (2d)

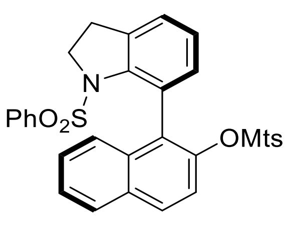

2d

Compound **2d** was prepared in 79% yield (48.8 mg) according to the general procedure at 50 °C as a pale yellow oil. $[\alpha]_D^{20}$ = +192.5° (c = 1.0, $CHCl_3$). 94% ee (determined by HPLC: Chiralpak ADH Column, 30/70 ^{i}PrOH/hexane, 1.0 mL/min, 254 nm; TR = 7.23 min (major), 10.55 min (minor)). ^{1}H NMR (400 MHz, $CDCl_3$) δ 7.85 – 7.77 (m, 2H), 7.75 (d, *J* = 9.2 Hz, 1H), 7.48 – 7.41 (m, 3H), 7.34 – 7.32 (m, 2H), 7.25 – 7.10 (m, 5H),

6.88 (s, 2H), 4.10 – 4.03 (m, 1H), 3.81 – 3.74 (m, 1H), 2.53 – 2.44 (m, 1H), 2.41 (s, 6H), 2.35 – 2.31 (m, 1H), 2.29 (s, 3H); ^{13}C NMR (100 MHz, $CDCl_3$) δ 144.3, 143.2, 141.8, 139.7, 138.3, 137.5, 132.9, 132.6, 131.9, 131.8, 131.6, 130.0, 129.2, 128.5, 128.0, 127.6, 127.0, 126.5, 126.4, 126.1, 125.7, 124.5, 119.9, 52.3, 29.3, 22.6, 21.0; IR (neat): 2945 (bs), 1605, 1366, 1203, 1157, 943, 680, 572; HRESIMS Calcd for $[C_{33}H_{29}NNaO_5S_2]^+$ (M + Na^+) 606.1379, found 606.1372.

(*S*)-1-(1-((4-bromophenyl)sulfonyl)indolin-7-yl)naphthalen-2-yl 2,4,6-trimethylbenzenesulfonate (2e)

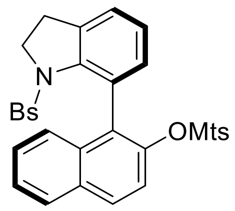

2e

Compound **2e** was prepared in 70% yield (48.8 mg) according to the general procedure at 50 °C as a pale yellow oil. $[\alpha]_D^{20}$ = +240.6° (c = 1.0, $CHCl_3$). 94% ee (determined by HPLC: Chiralpak IA Column, 30/70 ^{i}PrOH/hexane, 1.0 mL/min, 254 nm; TR = 7.12 min (major), 8.94 min (minor)). ^{1}H NMR (400 MHz, $CDCl_3$) δ 7.85 – 7.82 (m, 1H), 7.75 (d, *J* = 8.8 Hz, 1H), 7.69 (dd, *J* = 8.4, 6.4 Hz, 1H), 7.49 – 7.42 (m, 2H), 7.34 – 7.30 (m, 2H), 7.22 – 7.13 (m, 6H), 6.90 (s, 2H), 4.14 – 4.07 (m, 1H), 3.88 – 3.80 (m, 1H), 2.64 – 2.56 (m, 1H), 2.48 – 2.44 (m, 1H), 2.42 (s, 6H), 2.32 (s, 3H); ^{13}C NMR (100 MHz, $CDCl_3$) δ 144.5, 143.4, 141.6, 139.8, 137.7, 137.3, 132.8, 132.4, 132.0, 131.9, 131.7, 131.6, 129.8, 129.3, 128.3, 128.1, 127.5, 127.4, 126.6, 126.3, 126.2, 125.8, 124.7, 119.8, 52.5, 29.5, 22.6, 21.0; IR (neat): 3044 (bs), 1635, 1363, 1265, 1170, 1010, 953, 572; HRESIMS Calcd for $[C_{33}H_{28}BrNNaO_5S_2]^+$ (M + Na^+) 684.0484, found 684.0480.

(*S*)-1-(1-(methylsulfonyl)indolin-7-yl)naphthalen-2-yl 2,4,6-trimethylbenzenesulfonate (2f)

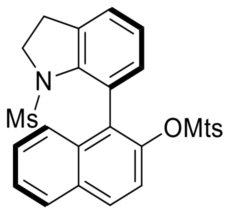

2f

Compound **2f** was prepared in 80% yield (48.8 mg) according to the general procedure at 50 °C as a pale yellow oil. $[\alpha]_D^{20}$ = +90.2° (c = 1.0, $CHCl_3$). 88% ee (determined by HPLC: Chiralpak IA Column, 30/70 ^{i}PrOH/hexane, 1.0 mL/min, 254 nm; TR = 6.46 min (major), 7.72 min (minor)). ^{1}H NMR (400 MHz, $CDCl_3$) δ 7.86 – 7.75 (m, 3H), 7.50 – 7.42 (m, 2H), 7.33 (d, *J* = 9.2 Hz, 1H), 7.27 (d, *J* = 8.4 Hz, 1H), 7.09 – 7.05 (m, 1H), 6.97 (d, *J* = 7.6 Hz, 1H), 6.86 (s, 2H), 4.32 – 4.25 (m, 1H), 3.81 – 3.73 (m, 1H), 3.29 – 3.21 (m, 1H), 2.87 – 2.80 (m, 1H), 2.35 (s, 6H), 2.31 (s, 3H), 2.26 (s, 3H); ^{13}C NMR (100 MHz, $CDCl_3$) δ 144.1, 143.3, 142.3, 139.8, 136.5, 133.0, 132.1, 131.9, 131.7, 131.3, 129.7, 129.5, 127.9, 127.0, 126.7, 126.2, 126.1, 125.5, 124.6, 120.1, 52.4, 40.2, 30.6, 22.7, 21.0; IR (neat): 2945 (bs), 1535, 1422, 1354, 1277, 940, 832, 732, 684, 577; HRESIMS Calcd for $[C_{28}H_{27}NNaO_5S_2]^+$ (M + Na^+) 544.1223, found 544.1219.

(*S*)-1-(1-tosylindolin-7-yl)naphthalen-2-yl 4-methylbenzenesulfonate (2g)

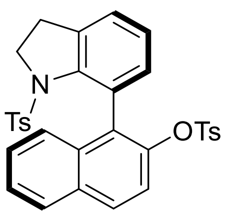

2g

Compound **2g** was prepared in 66% yield (48.8 mg) according to the general procedure at 50 °C as a pale yellow oil. $[\alpha]_D^{20}$ = +269.6° (c = 1.0, $CHCl_3$). 88% ee (determined by HPLC: Chiralpak IA Column, 30/70 ^{i}PrOH/hexane, 1.0 mL/min, 254 nm; TR = 10.64 min (major), 15.61 min (minor)). ^{1}H NMR (400 MHz, $CDCl_3$) δ 7.88 – 7.80 (m, 3H), 7.49 – 7.45 (m, 3H), 7.32 (d, *J* = 8.4 Hz, 2H), 7.18 – 7.08 (m, 6H), 7.03 – 6.97 (m, 3H), 4.09 – 4.03 (m, 1H), 3.70 – 3.62 (m, 1H), 2.48 – 2.41 (m, 1H), 2.38 (s, 3H), 2.36 – 2.35 (m, 1H), 2.33 (s, 3H); ^{13}C NMR (100 MHz, $CDCl_3$) δ 144.5, 143.7, 143.4, 142.2, 137.9,

135.5, 133.3, 133.0, 132.2, 131.7, 130.0, 129.5, 129.2, 129.1, 128.1, 128.0, 127.6, 127.1, 126.6, 125.8, 125.7, 124.3, 120.4, 52.2, 29.5, 21.6, 21.5; IR (neat): 3061 (bs), 1597, 1356, 1280, 1167, 1092, 956, 737; HRESIMS Calcd for $[C_{32}H_{27}NNaO_5S_2]^+$ (M + Na^+) 592.1223, found 592.1221.

(*S*)-1-(1-tosylindolin-7-yl)naphthalen-2-yl 4-methoxybenzenesulfonate (2h)

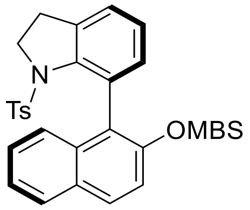

2h

Compound **2h** was prepared in 49% yield (48.8 mg) according to the general procedure at 50 ºC as a pale yellow oil. $[\alpha]_D^{20}$ = +228.1º (c = 1.0, $CHCl_3$). 92% ee (determined by HPLC: Chiralpak IA Column, 30/70 ^{i}PrOH/hexane, 1.0 mL/min, 254 nm; TR = 12.91 min (major), 19.25 min (minor)). ^{1}H NMR (400 MHz, $CDCl_3$) δ 7.88 – 7.81 (m, 3H), 7.49 – 7.46 (m, 3H), 7.33 (d, *J* = 8.8 Hz, 2H), 7.18 – 7.10 (m, 4H), 7.02 (d, *J* = 8.0 Hz, 2H), 6.98 (d, *J* = 7.2 Hz, 1H), 6.76 (d, *J* = 8.8 Hz, 2H), 4.09 – 4.03 (m, 1H), 3.81 (s, 3H), 3.71 – 3.63 (m, 1H), 2.47 – 2.37 (m, 2H), 2.33 (s, 3H); ^{13}C NMR (100 MHz, $CDCl_3$) δ 163.6, 143.7, 143.4, 142.2, 137.9, 135.4, 133.0, 132.2, 131.7, 130.1, 129.9, 129.2, 129.1, 128.1, 127.7, 127.5, 127.0, 126.5, 125.9, 125.7, 124.3, 120.5, 114.0, 55.7, 52.2, 29.5, 21.5; IR (neat): 3060 (bs), 1597, 1356, 1280, 1165, 1092, 934, 823, 688; HRESIMS Calcd for $[C_{32}H_{27}NNaO_6S_2]^+$ (M + Na^+) 608.1172, found 608.1180.

(*S*)-1-(1-tosylindolin-7-yl)naphthalen-2-yl 4-(*tert*-butyl)benzenesulfonate (2i)

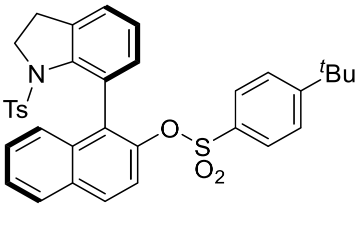

2i

Compound **2i** was prepared in 65% yield (48.8 mg) according to the general procedure

at 50 °C as a Pale yellow oil. $[\alpha]_D^{20}$ = +219.6° (c = 1.0, $CHCl_3$). 92% ee (determined by HPLC: Chiralpak IA Column, 30/70 ^{i}PrOH/hexane, 1.0 mL/min, 254 nm; TR = 9.03 min (major), 11.34 min (minor)). ^{1}H NMR (400 MHz, $CDCl_3$) δ 7.89 – 7.83 (m, 3H), 7.53 – 7.47 (m, 3H), 7.36 – 7.28 (m, 4H), 7.18 – 7.07 (m, 4H), 7.03 (d, *J* = 7.6 Hz, 2H), 6.94 (d, *J* = 7.6 Hz, 1H), 4.07 – 4.02 (m, 1H), 3.62 – 3.54 (m, 1H), 2.47 – 2.38 (m, 2H), 2.33 (s, 3H), 1.31 (s, 9H); ^{13}C NMR (100 MHz, $CDCl_3$) δ 157.5, 143.7, 143.4, 142.2, 137.9, 135.5, 133.1, 133.0, 132.2, 131.6, 129.9, 129.3, 129.1, 128.1, 127.7, 127.6, 127.1, 126.6, 125.9, 125.8, 125.7, 124.3, 120.5, 52.1, 35.2, 31.0, 29.5, 21.5; IR (neat): 2963 (bs), 1595, 1358, 1280, 1167, 1089, 955, 800, 679, 572; HRESIMS Calcd for $[C_{35}H_{33}NNaO_5S_2]^+$ (M + Na^+) 634.1692, found 634.1692.

(*S*)-1-(1-tosylindolin-7-yl)naphthalen-2-yl 4-bromobenzenesulfonate (2j)

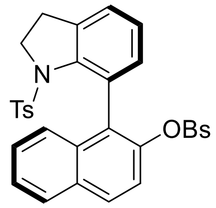

2j

Compound **2j** was prepared in 70% yield (48.8 mg) according to the general procedure at 50 °C as a pale yellow oil. $[\alpha]_D^{20}$ = +206.8° (c = 1.0, $CHCl_3$). 88% ee (determined by HPLC: Chiralpak IA Column, 30/70 ^{i}PrOH/hexane, 1.0 mL/min, 254 nm; TR = 9.84 min (major), 14.86 min (minor)). ^{1}H NMR (400 MHz, $CDCl_3$) δ 7.89 – 7.84 (m, 3H), 7.51 – 7.46 (m, 3H), 7.44 – 7.41 (m, 2H), 7.24 – 7.21 (m, 2H), 7.16 – 7.14 (m, 3H), 7.11 – 7.08 (m, 1H), 7.02 (d, *J* = 8.0 Hz, 2H), 6.90 (dd, *J* = 7.6, 1.2 Hz, 1H), 4.12 – 4.05 (m, 1H), 3.71 – 3.63 (m, 1H), 2.42 – 2.37 (m, 2H), 2.33 (s, 3H); ^{13}C NMR (100 MHz, $CDCl_3$) δ 143.5, 143.3, 142.2, 138.0, 135.3, 134.9, 133.0, 132.3, 132.2, 131.5, 129.8, 129.4, 129.2, 129.1, 128.8, 128.2, 127.3, 127.0, 126.7, 126.5, 126.0, 125.9, 124.4, 120.4, 52.2, 29.4, 21.5; IR (neat): 2943 (bs), 1635, 1357, 1201, 1166, 1091, 954, 800, 678; HRESIMS Calcd for $[C_{31}H_{24}BrNNaO_5S_2]^+$ (M + Na^+) 656.0171, found 656.0174.

(*S*)-1-(1-tosylindolin-7-yl)naphthalen-2-yl 4-nitrobenzenesulfonate (2k)

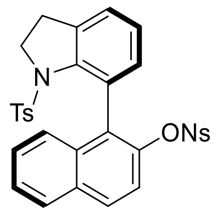

2k

Compound **2k** was prepared in 41% yield (48.8 mg) according to the general procedure at 40 °C as a pale yellow oil. $[\alpha]_D^{20}$ = +228.1° (c = 1.0, $CHCl_3$). 86% ee (determined by HPLC: Chiralpak IA Column, 50/50 ^{i}PrOH/hexane, 1.0 mL/min, 254 nm; TR = 8.36 min (major), 11.54 min (minor)). ^{1}H NMR (400 MHz, $CDCl_3$) δ 8.09 (d, J = 8.8 Hz, 2H), 7.89 – 7.83 (m, 3H), 7.56 – 7.48 (m, 5H), 7.16 – 7.13 (m, 3H), 7.05 – 7.00 (m, 3H), 6.84 (d, J = 7.6 Hz, 1H), 4.12 – 4.06 (m, 1H), 3.77 – 3.69 (m, 1H), 2.44 – 2.37 (m, 2H), 2.33 (s, 3H); ^{13}C NMR (100 MHz, $CDCl_3$) δ 150.4, 143.7, 143.2, 142.3, 141.4, 138.1, 135.2, 132.9, 132.4, 131.4, 129.6, 129.2, 129.1, 128.2, 127.0(3), 127.0(0), 126.9, 126.5, 126.2, 125.8, 124.5, 123.9, 120.2, 52.2, 29.4, 21.5; IR (neat): 2948 (bs), 1637, 1546, 1357, 1166, 803, 737, 680, 588; HRESIMS Calcd for $[C_{31}H_{24}N_2NaO_7S_2]^+$ (M + Na^+) 623.0917, found 623.0916.

(*S*)-1-(1-tosylindolin-7-yl)naphthalen-2-yl 2-nitrobenzenesulfonate (2l)

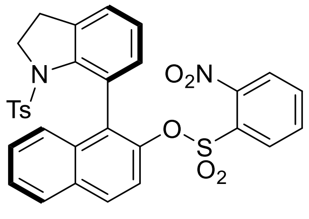

2l

Compound **2l** was prepared in 45% yield (48.8 mg) according to the general procedure at 40 °C as a pale yellow oil. $[\alpha]_D^{20}$ = +148.5° (c = 1.0, $CHCl_3$). 81% ee (determined by HPLC: Chiralpak IA Column, 50/50 ^{i}PrOH/hexane, 1.0 mL/min, 254 nm; TR = 8.10 min (major), 9.27 min (minor)). ^{1}H NMR (400 MHz, $CDCl_3$) δ 7.89 – 7.81 (m, 3H), 7.70 – 7.62 (m, 3H), 7.56 – 7.46 (m, 3H), 7.37 (d, J = 9.2 Hz, 1H), 7.21 – 7.19 (m, 2H), 7.07 – 7.03 (m, 3H), 6.90 – 6.86 (m, 1H), 6.78 (d, J = 7.6 Hz, 1H), 4.17 – 4.11 (m, 1H), 4.05 – 3.97 (m, 1H), 2.51 – 2.43 (m, 2H), 2.34 (s, 3H); ^{13}C NMR (100 MHz, $CDCl_3$) δ

143.9, 143.4, 142.4, 138.4, 134.6, 133.1, 132.3, 132.0, 131.2, 130.8, 129.9, 129.4, 129.2, 128.1, 126.9, 126.7, 126.6(8), 126.6(0), 126.2, 125.4, 124.8, 124.4, 120.8, 52.3, 29.5, 21.5; IR (neat): 2945 (bs), 1533, 1353, 1201, 1166, 1091, 954, 803, 680, 571; HRESIMS Calcd for $[C_{31}H_{24}N_2NaO_7S_2]^+$ (M + Na^+) 623.0917, found 623.0911.

(*S*)-1-(1-tosylindolin-7-yl)naphthalen-2-yl naphthalene-1-sulfonate (2m)

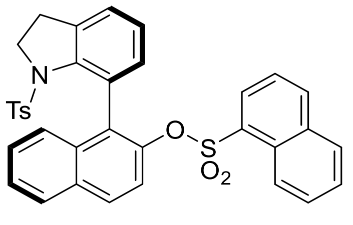

2m

Compound **2m** was prepared in 79% yield (48.8 mg) according to the general procedure at 50 ºC as a pale yellow oil. $[\alpha]_D^{20}$ = +137.0º (c = 1.0, $CHCl_3$). 85% ee (determined by HPLC: Chiralpak IA Column, 30/70 ^{i}PrOH/hexane, 1.0 mL/min, 254 nm; TR = 11.27 min (major), 15.53 min (minor)). ^{1}H NMR (400 MHz, $CDCl_3$) δ 8.57 – 8.54 (m, 1H), 8.02 (d, *J* = 8.0 Hz, 1H), 7.88 – 7.83 (m, 3H), 7.78 (d, *J* = 9.2 Hz, 2H), 7.57 – 7.52 (m, 2H), 7.48 – 7.41 (m, 2H), 7.38 – 7.32 (m, 2H), 7.11 (d, *J* = 8.0 Hz, 2H), 6.96 (d, *J* = 8.0 Hz, 2H), 6.87 (d, *J* = 6.8 Hz, 1H), 6.83 – 6.79 (m, 1H), 6.74 (d, *J* = 7.2 Hz, 1H), 3.99 – 3.92 (m, 1H), 3.42 – 3.34 (m, 1H), 2.30 (s, 3H), 2.30 – 2.23 (m, 1H), 2.05 – 1.98 (m, 1H); ^{13}C NMR (100 MHz, $CDCl_3$) δ 144.0, 143.3, 141.6, 137.3, 135.6, 135.0, 134.1, 133.2, 132.4, 132.2, 131.4, 130.0, 129.8, 129.1, 129.0, 128.6, 128.4, 128.3, 128.0, 127.0, 126.9, 126.8, 126.6, 126.5, 125.8, 125.3, 125.1, 124.1, 124.0, 121.0, 52.0, 29.3, 21.4; IR (neat): 2936 (bs), 1533, 1201, 1167, 1090, 954, 572; HRESIMS Calcd for $[C_{35}H_{27}NNaO_5S_2]^+$ (M + Na^+) 628.1223, found 628.1227.

(*S*)-1-(1-tosylindolin-7-yl)naphthalen-2-yl methanesulfonate (2n)

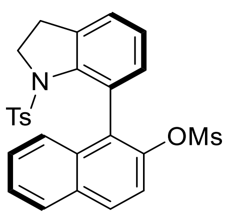

2n

Compound **2n** was prepared in 68% yield (48.8 mg) according to the general procedure at 40 °C as a pale yellow oil. $[\alpha]_D^{20}$ = +180.5° (c = 1.0, $CHCl_3$). 68% ee (determined by HPLC: Chiralpak IA Column, 20/80 ^{i}PrOH/hexane, 1.0 mL/min, 254 nm; TR = 20.26 min (major), 22.81 min (minor)). ^{1}H NMR (400 MHz, $CDCl_3$) δ 7.88 – 7.86 (m, 3H), 7.57 (d, *J* = 9.2 Hz, 1H), 7.53 – 7.49 (m, 2H), 7.34 – 7.27 (m, 3H), 7.18 (d, *J* = 7.6 Hz, 2H), 7.06 (d, *J* = 7.6 Hz, 2H), 4.18 – 4.13 (m, 1H), 4.06 – 3.98 (m, 1H), 2.66 (s, 3H), 2.62 – 2.49 (m, 2H), 2.35 (s, 3H); ^{13}C NMR (100 MHz, $CDCl_3$) δ 144.1, 143.6, 142.4, 138.2, 135.4, 132.8, 132.2, 131.8, 129.6, 129.2, 129.1, 128.2, 127.5, 127.0, 126.8, 126.5, 126.2, 125.9, 124.9, 120.1, 52.4, 37.6, 29.6, 21.5; IR (neat): 3044 (bs), 1635, 1360, 1281, 1166, 1090, 1038, 951, 677, 570; HRESIMS Calcd for $[C_{26}H_{23}NNaO_5S_2]^+$ (M + Na^+) 516.0910, found 516.0903.

(*S*)-1-(1-tosylindolin-7-yl)naphthalen-2-yl trifluoromethanesulfonate (2o)

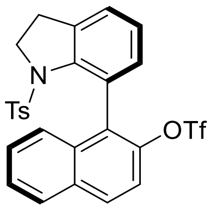

2o

Compound **2o** was prepared in 67% yield (48.8 mg) according to the general procedure at 40 °C as a yellow solid (mp 85–87 °C), $[\alpha]_D^{20}$ = +271.7° (c = 1.0, $CHCl_3$). 96% ee (determined by HPLC: Chiralpak ODH Column, 30/70 ^{i}PrOH/hexane, 1.0 mL/min, 254 nm; TR = 5.25 min (minor), 6.80 min (major)). ^{1}H NMR (400 MHz, $CDCl_3$) δ 7.95 – 7.90 (m, 3H), 7.56 – 7.55 (m, 2H), 7.41 (d, *J* = 9.2 Hz, 1H), 7.33 – 7.28 (m, 5H), 7.12 – 7.10 (m, 2H), 4.16 – 4.11 (m, 1H), 4.05 – 3.97 (m, 1H), 2.53 – 2.34 (m, 2H), 2.36 (s, 3H); ^{13}C NMR (100 MHz, $CDCl_3$) δ 144.1, 143.7, 142.1, 138.4, 135.2, 133.1, 132.7, 132.0, 130.6, 130.0, 129.3, 128.3, 127.2, 126.7, 126.6, 126.4, 126.3, 125.4, 119.0, 118.4 (q, *J* = 320.3 Hz), 52.3, 29.2, 21.5; ^{19}F NMR (376 MHz, $CDCl_3$) δ -74.5; IR (neat): 2944 (bs), 1419, 1359, 1216, 1157, 949, 830, 678; HRESIMS Calcd for $[C_{26}H_{20}F_3NNaO_5S_2]^+$ (M + Na^+) 570.0627, found 570.0636.

(*S*)-3-phenyl-1-(1-tosylindolin-7-yl)naphthalen-2-yl 2,4,6-trimethylbenzenesulfonate (2p)

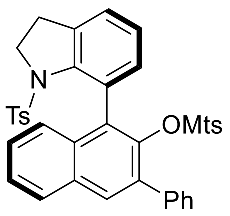

2p

Compound **2p** was prepared in 69% yield (46.5 mg) according to the general procedure at 50 °C as a pale yellow oil. $[\alpha]_D^{20}$ = +85.4° (c = 1.0, $CHCl_3$). 96% ee (determined by HPLC: Chiralpak ADH Column, 30/70 ^{i}PrOH/hexane, 1.0 mL/min, 254 nm; TR = 11.23 min (major), 14.96 min (minor)). ^{1}H NMR (400 MHz, $CDCl_3$) δ 7.96 – 7.94 (m, 1H), 7.87 – 7.85 (m, 1H), 7.76 (s, 1H), 7.51 – 7.49 (m, 2H), 7.40 (d, *J* = 7.2 Hz, 1H), 7.27 – 7.24 (m, 5H), 7.19 (d, *J* = 7.2 Hz, 1H), 7.07 – 6.99 (m, 5H), 6.56 (s, 2H), 4.28 – 4.23 (m, 1H), 4.08 – 4.00 (m, 1H), 2.55 – 2.51 (m, 2H), 2.29 (s, 3H), 2.26 (s, 6H), 2.17 (s, 3H); ^{13}C NMR (100 MHz, $CDCl_3$) δ 143.4, 142.9, 142.1, 142.0, 138.3, 137.8, 137.7, 135.9, 134.6, 133.8, 133.0, 132.8, 132.3, 131.2, 130.2, 129.2, 129.1, 128.4, 128.1, 127.5, 127.1, 126.8, 126.7, 126.4, 126.1, 125.8, 124.7, 52.5, 29.6, 22.6, 21.4, 20.8; IR (neat): 3042 (bs), 2924, 1601, 1408, 1360, 1279, 1166, 963, 801, 665; HRESIMS Calcd for $[C_{40}H_{35}NNaO_5S_2]^+$ (M + Na^+) 696.1849, found 696.1855.

(*S*)-3-methoxy-1-(1-tosylindolin-7-yl)naphthalen-2-yl 2,4,6-trimethylbenzenesulfonate (2q)

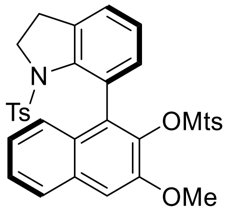

2q

Compound **2q** was prepared in 73% yield (45.8 mg) according to the general procedure at 50 °C as a pale yellow oil. $[\alpha]_D^{20}$ = +139.0° (c = 1.0, $CHCl_3$). 93% ee (determined by

HPLC: Chiralpak IA Column, 30/70 ^{i}PrOH/hexane, 1.0 mL/min, 254 nm; TR = 10.10 min (major), 11.58 min (minor)). ^{1}H NMR (400 MHz, $CDCl_3$) δ 7.72 (d, *J* = 8.0 Hz, 1H), 7.68 (d, *J* = 8.4 Hz, 1H), 7.44 – 7.28 (m, 5H), 7.22 – 7.18 (m, 1H), 7.11 – 7.06 (m, 4H), 6.89 (s, 2H), 4.14 – 4.07 (m, 1H), 4.02 – 3.95 (m, 1H), 3.52 (s, 3H), 2.54 (s, 6H), 2.47 – 2.37 (m, 2H), 2.34 (s, 3H), 2.30 (s, 3H); ^{13}C NMR (100 MHz, $CDCl_3$) δ 150.3, 143.3, 142.0, 141.7, 138.7, 137.5, 137.3, 135.6, 134.9, 132.8, 132.1, 131.0, 129.1, 127.9, 127.6, 127.1, 126.8, 126.5, 126.2, 126.0, 124.7, 124.1, 106.8, 55.1, 52.3, 29.3, 22.6, 21.5, 20.9; IR (neat): 2943 (bs), 1635, 1467, 1362, 1277, 1167, 1077, 751, 676, 578; HRESIMS Calcd for $[C_{35}H_{33}NNaO_6S_2]^+$ (M + Na^+) 650.1642, found 650.1648.

(*S*)-6-bromo-1-(1-tosylindolin-7-yl)naphthalen-2-yl 2,4,6-trimethylbenzenesulfonate (2r)

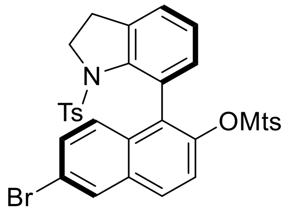

2r

Compound **2r** was prepared in 65% yield (48.8 mg) according to the general procedure at 50 °C as a pale yellow oil. $[\alpha]_D^{20}$ = +130.0° (c = 1.0, $CHCl_3$). 94% ee (determined by HPLC: Chiralpak IA Column, 15/85 ^{i}PrOH/hexane, 1.0 mL/min, 254 nm; TR = 13.99 min (major), 16.38 min (minor)). ^{1}H NMR (400 MHz, $CDCl_3$) δ 8.00 (d, *J* = 2.0 Hz, 1H), 7.72 (d, *J* = 9.2 Hz, 1H), 7.68 (d, *J* = 9.2 Hz, 1H), 7.52 – 7.49 (m, 1H), 7.22 – 7.20 (m, 3H), 7.16 – 7.13 (m, 3H), 7.04 (d, *J* = 8.0 Hz, 2H), 6.89 (s, 2H), 4.05 – 3.99 (m, 1H), 3.80 – 3.73 (m, 1H), 2.46 (dd, *J* = 15.6, 8.0 Hz, 1H), 2.41 (s, 6H), 2.36 – 2.33 (m, 1H), 2.34 (s, 3H), 2.30 (s, 3H); ^{13}C NMR (100 MHz, $CDCl_3$) δ 144.6, 143.5, 143.4, 141.9, 139.8, 137.7, 135.4, 133.0, 132.3, 131.7, 131.5, 130.6, 130.0, 129.9, 129.2, 128.4, 128.2, 127.1, 126.1, 124.7, 121.2, 119.9, 52.2, 29.3, 22.6, 21.5, 21.0; IR (neat): 2951 (bs), 2923, 1585, 1495, 1361, 1168, 953, 792, 679; HRESIMS Calcd for $[C_{34}H_{30}BrNNaO_5S_2]^+$ (M + Na^+) 698.0641, found 698.0645.

(*S*)-6-phenyl-1-(1-tosylindolin-7-yl)naphthalen-2-yl 2,4,6-trimethylbenzenesulfonate (2s)

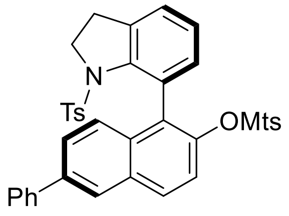

2s

Compound **2s** was prepared in 81% yield (48.8 mg) according to the general procedure at 50 °C as a pale yellow oil. $[\alpha]_D^{20}$ = -103.8° (c = 1.0, $CHCl_3$). 96% ee (determined by HPLC: Chiralpak IA Column, 30/70 ^{i}PrOH/hexane, 1.0 mL/min, 254 nm; TR = 10.74 min (major), 14.32 min (minor)). ^{1}H NMR (400 MHz, $CDCl_3$) δ 8.03 (d, J = 1.6 Hz, 1H), 7.85 – 7.80 (m, 2H), 7.72 – 7.69 (m, 3H), 7.48 – 7.44 (m, 2H), 7.38 – 7.34 (m, 1H), 7.26 (d, J = 8.4 Hz, 2H), 7.22 – 7.12 (m, 4H), 7.04 (d, J = 8.0 Hz, 2H), 6.89 (s, 2H), 4.09 – 4.02 (m, 1H), 3.83 – 3.76 (m, 1H), 2.51 – 2.45 (m, 1H), 2.43 (s, 6H), 2.37 – 2.34 (m, 1H), 2.33 (s, 3H), 2.30 (s, 3H); ^{13}C NMR (100 MHz, $CDCl_3$) δ 144.4, 143.4, 143.2, 141.9, 140.8, 139.8, 138.3, 137.6, 135.5, 132.5, 132.2, 132.1, 131.9, 131.6, 130.1, 129.4, 129.2, 128.8, 127.6, 127.4, 127.1, 127.0, 126.3, 126.1, 125.9, 124.5, 120.4, 52.3, 29.3, 22.6, 21.5, 21.0; IR (neat): 2943 (bs), 2924, 1601, 1408, 1360, 1167, 963, 801, 665; HRESIMS Calcd for $[C_{40}H_{35}NNaO_5S_2]^+$ (M + Na$^+$) 696.1849, found 696.1852.

(*S*)-6-methyl-1-(1-tosylindolin-7-yl)naphthalen-2-yl 2,4,6-trimethylbenzenesulfonate (2t)

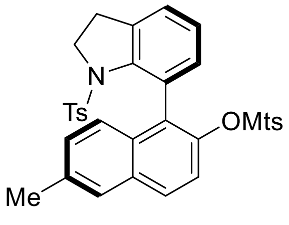

2t

Compound **2t** was prepared in 88% yield (48.8 mg) according to the general procedure at 50 °C as a pale yellow oil. $[\alpha]_D^{20}$ = +111.5° (c = 1.0, $CHCl_3$). 96% ee (determined by HPLC: Chiralpak IA Column, 30/70 ^{i}PrOH/hexane, 1.0 mL/min, 254 nm; TR = 7.52

min (major), 9.27 min (minor)). ^{1}H NMR (400 MHz, $CDCl_3$) δ 7.69 – 7.65 (m, 2H), 7.60 (s, 1H), 7.28 (dd, *J* = 8.8, 1.6 Hz, 1H), 7.23 – 7.21 (m, 2H), 7.19 – 7.14 (m, 2H), 7.12 (d, *J* = 6.4 Hz, 2H), 7.03 (d, *J* = 8.0 Hz, 2H), 6.87 (s, 2H), 4.07 – 4.01 (m, 1H), 3.80 – 3.72 (m, 1H), 2.48 (s, 3H), 2.45 – 2.42 (m, 1H), 2.40 (s, 6H), 2.36 – 2.35 (m, 1H), 2.33 (s, 3H), 2.29 (s, 3H); ^{13}C NMR (100 MHz, $CDCl_3$) δ 143.7, 143.3, 143.1, 141.9, 139.8, 137.5, 135.5, 135.3, 132.4, 132.2, 131.8, 131.6, 131.1, 130.0, 129.1, 128.8, 128.4, 127.8, 127.1, 126.3, 125.9, 124.4, 120.0, 52.2, 29.3, 22.6, 21.5, 21.0; IR (neat): 2940 (bs), 1689, 1597, 1367, 1171, 950, 815, 734; HRESIMS Calcd for $[C_{35}H_{33}NNaO_5S_2]^+$ (M + Na^+) 634.1692, found 634.1693.

(*S*)-6-methoxy-1-(1-tosylindolin-7-yl)naphthalen-2-yl 2,4,6-trimethylbenzenesulfonate (2u)

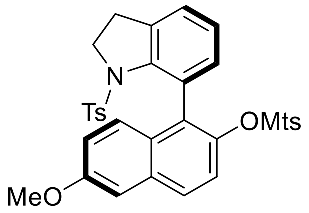

2u

Compound **2u** was prepared in 55% yield (48.8 mg) according to the general procedure at 50 °C as a pale yellow oil. $[\alpha]_D^{20}$ = +2.3° (c = 1.0, $CHCl_3$). 96% ee (determined by HPLC: Chiralpak ODH Column, 30/70 ^{i}PrOH/hexane, 1.0 mL/min, 254 nm; TR = 7.61 min (minor), 10.46 min (major)). ^{1}H NMR (400 MHz, $CDCl_3$) δ 7.67 (d, *J* = 8.8 Hz, 1H), 7.64 (d, *J* = 9.2 Hz, 1H), 7.23 (d, *J* = 8.4 Hz, 2H), 7.17 – 7.10 (m, 6H), 7.04 (d, *J* = 8.0 Hz, 2H), 6.87 (s, 2H), 4.08 – 4.01 (m, 1H), 3.90 (s, 3H), 3.80 – 3.72 (m, 1H), 2.50 – 2.44 (m, 2H), 2.40 (s, 6H), 2.34 (s, 3H), 2.30 (s, 3H); ^{13}C NMR (100 MHz, $CDCl_3$) δ 157.4, 143.3, 143.1, 142.8, 141.9, 139.8, 137.6, 135.6, 133.3, 132.4, 131.8, 131.6, 130.3, 129.1, 128.2, 128.1, 127.8, 127.7, 127.1, 126.0, 124.4, 120.5, 119.3, 106.0, 55.3, 52.3, 29.4, 22.6, 21.5, 21.0; IR (neat): 2940 (bs), 1689, 1623, 1507, 1367, 1231, 1169, 829, 737, 673; HRESIMS Calcd for $[C_{35}H_{33}NNaO_6S_2]^+$ (M + Na^+) 650.1642, found 650.1639.

(*S*)-7-bromo-1-(1-tosylindolin-7-yl)naphthalen-2-yl 2,4,6-trimethylbenzenesulfonate (2v)

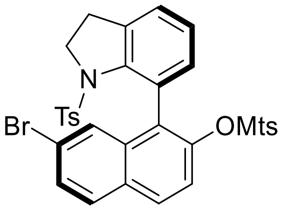

2v

Compound **2v** was prepared in 71% yield (48.8 mg) according to the general procedure at 50 °C as a pale yellow oil. $[\alpha]_D^{20}$ = +107.6° (c = 1.0, $CHCl_3$). 93% ee (determined by HPLC: Chiralpak ADH Column, 30/70 ^{i}PrOH/hexane, 1.0 mL/min, 254 nm; TR = 8.58 min (minor), 11.05 min (major)). ^{1}H NMR (400 MHz, $CDCl_3$) δ 7.85 (d, *J* = 1.6 Hz, 1H), 7.75 – 7.70 (m, 2H), 7.53 (dd, *J* = 8.7, 1.9 Hz, 1H), 7.26 – 7.22 (m, 3H), 7.19 – 7.11 (m, 3H), 7.05 (d, *J* = 8.1 Hz, 2H), 6.89 (s, 2H), 4.08 – 4.01 (m, 1H), 3.85 – 3.78 (m, 1H), 2.50 – 2.44 (m, 1H), 2.43 (s, 6H), 2.34 (s, 3H), 2.31 (s, 3H), 2.29 – 2.27 (m, 1H); ^{13}C NMR (100 MHz, $CDCl_3$) δ 145.2, 143.5, 143.3, 141.7, 139.8, 137.7, 135.3, 134.1, 132.4, 131.7, 131.6, 130.3, 129.8, 129.5, 129.2, 129.1, 128.6, 127.2, 126.9, 126.3, 124.8, 121.1, 120.5, 52.4, 29.2, 22.6, 21.5, 21.0; IR (neat): 2923 (bs), 1585, 1496, 1361, 1187, 953, 792, 679; HRESIMS Calcd for $[C_{34}H_{30}BrNNaO_5S_2]^+$ (M + Na^+) 698.0641, found 698.0640.

(*S*)-7-phenyl-1-(1-tosylindolin-7-yl)naphthalen-2-yl 2,4,6-trimethylbenzenesulfonate (2w)

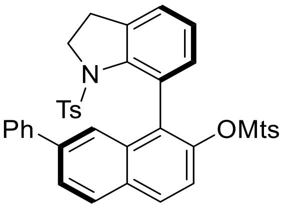

2w

Compound **2w** was prepared in 68% yield (48.8 mg) according to the general procedure at 50 °C as a pale yellow oil. $[\alpha]_D^{20}$ = +100.2° (c = 1.0, $CHCl_3$). 95% ee (determined by HPLC: Chiralpak ADH Column, 15/85 ^{i}PrOH/hexane, 1.0 mL/min, 254 nm; TR =

15.44 min (major), 21.18 min (minor)). ^{1}H NMR (400 MHz, $CDCl_3$) δ 7.93 – 7.89 (m, 2H), 7.77 (d, J = 8.8 Hz, 1H), 7.71 (d, J = 8.4 Hz, 1H), 7.56 (d, J = 7.2 Hz, 2H), 7.42 – 7.30 (m, 3H), 7.26 – 7.12 (m, 6H), 6.98 (d, J = 7.6 Hz, 2H), 6.89 (s, 2H), 4.11 – 4.05 (m, 1H), 3.85 – 3.78 (m, 1H), 2.56 – 2.50 (m, 2H), 2.43 (s, 6H), 2.31 (s, 3H), 2.30 (s, 3H); ^{13}C NMR (100 MHz, $CDCl_3$) δ 144.8, 143.4, 143.2, 141.8, 141.3, 139.8, 139.2, 137.6, 135.6, 133.2, 132.5, 131.9, 131.6, 131.1, 130.4, 129.1, 128.8, 128.7, 128.6, 127.5, 127.3, 127.1, 126.1, 125.5, 124.7, 124.6, 120.0, 52.4, 29.4, 22.6, 21.5, 21.0; IR (neat): 2942 (bs), 1645, 1361, 1212, 1167, 972, 632, 580; HRESIMS Calcd for $[C_{40}H_{35}NNaO_5S_2]^+$ (M + Na^+) 696.1849, found 696.1856.

(*S*)-7-methyl-1-(1-tosylindolin-7-yl)naphthalen-2-yl 2,4,6-trimethylbenzenesulfonate (2x)

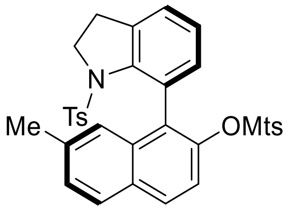

2x

Compound **2x** was prepared in 89% yield (48.8 mg) according to the general procedure at 50 °C as a pale yellow oil. $[\alpha]_D^{20}$ = +161.7° (c = 1.0, $CHCl_3$). 91% ee (determined by HPLC: Chiralpak ODH Column, 15/85 ^{i}PrOH/hexane, 1.0 mL/min, 254 nm; TR = 9.39 min (minor), 10.71 min (major)). ^{1}H NMR (400 MHz, $CDCl_3$) δ 7.74 – 7.68 (m, 2H), 7.49 (s, 1H), 7.28 (dd, J = 8.4, 1.6 Hz, 1H), 7.24 – 7.09 (m, 6H), 7.02 (d, J = 8.0 Hz, 2H), 6.87 (s, 2H), 4.10 – 4.00 (m, 1H), 3.82 – 3.73 (m, 1H), 2.56 – 2.46 (m, 2H), 2.42 (s, 3H), 2.41 (s, 6H), 2.32 (s, 3H), 2.29 (s, 3H); ^{13}C NMR (100 MHz, $CDCl_3$) δ 144.5, 143.3, 143.1, 141.8, 139.7, 137.5, 136.1, 135.6, 133.0, 132.5, 131.9, 131.6, 130.2, 129.4, 129.1, 128.8, 127.9, 127.8, 127.7, 127.1, 125.9, 125.4, 124.4, 118.9, 52.3, 29.4, 22.6, 22.1, 21.4, 21.0; IR (neat): 2947 (bs), 1632, 1598, 1360, 1167, 953, 796, 682; HRESIMS Calcd for $[C_{35}H_{33}NNaO_5S_2]^+$ (M + Na^+) 634.1692, found 634.1698.

(*S*)-7-methoxy-1-(1-tosylindolin-7-yl)naphthalen-2-yl 2,4,6-

trimethylbenzenesulfonate (2y)

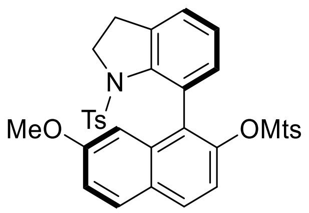

2y

Compound **2y** was prepared in 83% yield (48.8 mg) according to the general procedure at 50 ºC as a pale yellow oil. $[\alpha]_D^{20}$ = +135.2º (c = 1.0, $CHCl_3$). 95% ee (determined by HPLC: Chiralpak ODH Column, 15/85 ^{i}PrOH/hexane, 1.0 mL/min, 254 nm; TR = 11.46 min (minor), 13.65 min (minor)). ^{1}H NMR (400 MHz, $CDCl_3$) δ 7.73 (d, *J* = 8.8 Hz, 1H), 7.66 (d, *J* = 9.2 Hz, 1H), 7.25 – 7.22 (m, 3H), 7.17 – 7.11 (m, 3H), 7.06 – 6.99 (m, 4H), 6.89 (s, 2H), 4.09 – 4.02 (m, 1H), 3.83 – 3.78 (m, 1H), 3.75 (s, 3H), 2.59 – 2.47 (m, 2H), 2.43 (s, 6H), 2.33 (s, 3H), 2.30 (s, 3H); ^{13}C NMR (100 MHz, $CDCl_3$) δ 158.1, 145.1, 143.3, 143.1, 141.8, 139.7, 137.6, 135.7, 134.3, 132.6, 131.7, 131.6, 129.5, 129.1, 129.0, 128.8, 127.8, 127.4, 127.0, 126.1, 124.5, 118.0, 117.4, 105.4, 55.1, 52.4, 29.4, 22.6, 21.5, 21.0; IR (neat): 2943 (bs), 1636, 1362, 1277, 1167, 803, 751, 676, 578; HRESIMS Calcd for $[C_{35}H_{33}NNaO_6S_2]^+$ (M + Na^+) 650.1642, found 650.1646.

(*S*)-1-(1-tosylindolin-7-yl)-5,6,7,8-tetrahydronaphthalen-2-yl 2,4,6-trimethylbenzenesulfonate (2z)

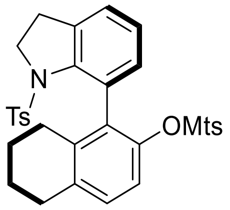

2z

Compound **2z** was prepared in 42% yield (48.8 mg) according to the general procedure at 50 ºC as a pale yellow oil. $[\alpha]_D^{20}$ = -9.9º (c = 1.0, $CHCl_3$). 97% ee (determined by HPLC: Chiralpak ADH Column, 30/70 ^{i}PrOH/hexane, 1.0 mL/min, 254 nm; TR = 5.75 min (major), 8.02 min (minor)). ^{1}H NMR (400 MHz, $CDCl_3$) δ 7.34 (d, *J* = 8.0 Hz, 2H), 7.19 – 7.11 (m, 2H), 7.07 – 7.02 (m, 3H), 6.94 (d, *J* = 8.4 Hz, 1H), 6.91 (s, 2H), 6.66

(d, J = 8.4 Hz, 1H), 3.97 – 3.83 (m, 2H), 2.80 – 2.76 (m, 2H), 2.69 – 2.61 (m, 1H), 2.48 (s, 6H), 2.46 – 2.37 (m, 2H), 2.35 (s, 3H), 2.31 (s, 3H), 2.24 – 2.15 (m, 1H), 1.80 – 1.68 (m, 4H); ^{13}C NMR (100 MHz, $CDCl_3$) δ 145.6, 143.4, 143.0, 141.3, 139.6, 137.5, 137.3, 135.4, 133.1, 132.8, 131.6, 131.2, 129.2, 129.1, 129.0, 127.3, 126.2, 124.0, 117.1, 52.4, 29.5, 29.2, 27.8, 22.9, 22.5, 21.5, 21.0; IR (neat): 2939 (bs), 1635, 1466, 1361, 1169, 788, 736, 560; HRESIMS Calcd for $[C_{34}H_{35}NNaO_5S_2]^+$ (M + Na^+) 624.1849, found 624.1858.

(*S*)-1-(1-((4-(*tert*-butyl)phenyl)sulfonyl)indolin-7-yl)naphthalen-2-yl trifluoromethanesulfonate (2aa)

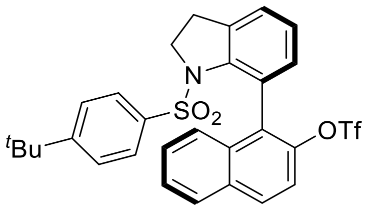

2aa

Compound **2aa** was prepared in 77% yield (48.8 mg) according to the general procedure at 40 °C as a pale yellow oil. $[\alpha]_D^{20}$ = -1.5° (c = 1.0, $CHCl_3$). 96% ee (determined by HPLC: Chiralpak ODH Column, 30/70 ^{i}PrOH/hexane, 1.0 mL/min, 254 nm; TR = 4.34 min (minor), 4.86 min (major)). ^{1}H NMR (400 MHz, $CDCl_3$) δ 7.98 – 7.95 (m, 1H), 7.92 – 7.90 (m, 1H), 7.88 (d, J = 9.2 Hz, 1H), 7.57 – 7.53 (m, 2H), 7.35 (d, J = 9.2 Hz, 1H), 7.32 – 7.26 (m, 7H), 4.20 – 4.14 (m, 1H), 4.04 – 3.97 (m, 1H), 2.56 – 2.38 (m, 2H), 1.29 (s, 9H); ^{13}C NMR (100 MHz, $CDCl_3$) δ 156.7, 144.1, 142.1, 138.3, 135.3, 133.2, 132.6, 132.0, 130.6, 130.0, 128.2, 127.2, 126.9, 126.6, 126.2, 125.6, 125.3, 118.9, 118.4 (q, J = 320.4 Hz), 52.4, 35.1, 31.0, 29.3; ^{19}F NMR (376 MHz, $CDCl_3$) δ -74.5; IR (neat): 2950 (bs), 1419, 1400, 1213, 1170, 1142, 950, 839, 584; HRESIMS Calcd for $[C_{29}H_{26}F_3NNaO_5S_2]^+$ (M + Na^+) 612.1097, found 612.1094.

(*S*)-1-(1-((4-nitrophenyl)sulfonyl)indolin-7-yl)naphthalen-2-yl trifluoromethanesulfonate (2ab)

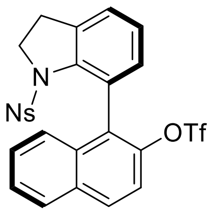

2ab

Compound **2ab** was prepared in 54% yield (48.8 mg) according to the general procedure at 40 °C as a pale yellow oil. $[\alpha]_D^{20}$ = +261.1° (c = 1.0, $CHCl_3$). 95% ee (determined by HPLC: Chiralpak IG Column, 15/85 ^{i}PrOH/hexane, 1.0 mL/min, 254 nm; TR = 10.38 min (major), 12.07 min (minor)). ^{1}H NMR (400 MHz, $CDCl_3$) δ 8.10 – 8.06 (m, 2H), 7.92 – 7.86 (m, 3H), 7.59 – 7.55 (m, 2H), 7.47 – 7.44 (m, 2H), 7.36 – 7.30 (m, 4H), 4.33 – 4.27 (m, 1H), 4.16 – 4.08 (m, 1H), 2.75 – 2.61 (m, 2H); ^{13}C NMR (100 MHz, $CDCl_3$) δ 149.9, 144.2, 141.3, 137.7, 132.8, 132.6, 132.2, 130.3, 129.9, 128.3, 127.9, 127.5, 126.9, 126.5, 125.9, 125.8, 123.7, 118.8, 118.4 (q, *J* = 320.6 Hz), 52.8, 29.6; ^{19}F NMR (376 MHz, $CDCl_3$) δ -74.4; IR (neat): 2945 (bs), 1533, 1355, 1156, 1099, 954, 808, 772, 680; HRESIMS Calcd for $[C_{25}H_{17}F_3N_2NaO_7S_2]^+$ (M + Na^+) 601.0321, found 601.0327.

(*S*)-8-methyl-1-(1-tosylindolin-7-yl)naphthalen-2-yl trifluoromethanesulfonate (2ac)

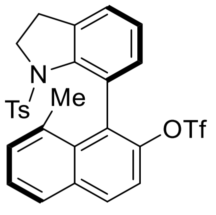

2ac

Compound **2ac** was prepared in 69% yield (48.8 mg) according to the general procedure at 40 °C as a pale yellow oil. $[\alpha]_D^{20}$ = +48.5° (c = 1.0, $CHCl_3$). 95% ee (determined by HPLC: Chiralpak IA Column, 15/85 ^{i}PrOH/hexane, 1.0 mL/min, 254 nm; TR = 6.63 min (major), 7.60 min (minor)). ^{1}H NMR (400 MHz, $CDCl_3$) δ 7.90 (d, *J* = 9.2 Hz, 1H), 7.77 (d, *J* = 8.0 Hz, 1H), 7.45 – 7.41 (m, 1H), 7.36 – 7.32 (m, 4H), 7.27 – 7.25 (m, 1H), 7.24 – 7.21 (m, 2H), 7.12 (d, *J* = 8.0 Hz, 2H), 4.09 – 3.96 (m, 2H), 2.66 – 2.58 (m, 1H),

2.51 – 2.44 (m, 1H), 2.38 (s, 3H), 2.27 (s, 3H); ^{13}C NMR (100 MHz, $CDCl_3$) δ 146.0, 143.6, 141.8, 137.9, 136.4, 136.0, 134.1, 132.5, 132.0, 131.4, 131.3, 130.4, 129.4, 129.3, 127.7, 127.1, 126.3, 125.5, 125.3, 118.4 (q, J = 320.1 Hz), 118.0, 52.7, 29.4, 24.7, 21.5; ^{19}F NMR (376 MHz, $CDCl_3$) δ -74.6; IR (neat): 2947 (bs), 1699, 1422, 1370, 1216, 1170, 837, 524; HRESIMS Calcd for $[C_{27}H_{22}F_3NNaO_5S_2]^+$ (M + Na^+) 584.0784, found 584.0781.

(*S*)-8-phenyl-1-(1-tosylindolin-7-yl)naphthalen-2-yl trifluoromethanesulfonate (2ad)

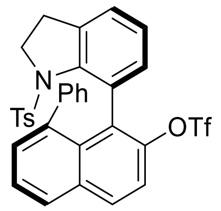

2ad

Compound **2ad** was prepared in 65% yield (48.8 mg) according to the general procedure at 40 °C as a pale yellow oil. $[\alpha]_D^{20}$ = +70.5° (c = 1.0, $CHCl_3$). 60% ee (determined by HPLC: Chiralpak IA Column, 30/70 ^{i}PrOH/hexane, 1.0 mL/min, 254 nm; TR = 4.90 min (minor), 6.25 min (major)). ^{1}H NMR (400 MHz, $CDCl_3$) δ 8.04 (d, J = 9.2 Hz, 1H), 7.93 (d, J = 8.0 Hz, 1H), 7.63 (d, J = 9.2 Hz, 1H), 7.55 – 7.51 (m, 1H), 7.37 – 7.22 (m, 3H), 7.22 (d, J = 6.8 Hz, 2H), 7.10 – 7.06 (m, 1H), 7.03 – 6.91 (m, 3H), 6.89 – 6.85 (m, 1H), 6.76 – 6.66 (m, 3H), 3.85 – 3.79 (m, 1H), 3.68 – 3.60 (m, 1H), 2.29 (s, 3H), 2.06 – 2.01 (m, 1H), 1.96 – 1.87 (m, 1H); ^{13}C NMR (100 MHz, $CDCl_3$) δ 147.7, 143.6, 141.8, 140.9, 140.4, 137.0, 134.8, 133.7, 132.8, 132.7, 131.9, 131.3, 131.1, 130.1, 129.1, 128.9, 128.2, 127.6, 127.2, 126.5, 126.2, 125.7, 125.1, 118.8, 118.3 (q, J = 319.9 Hz), 51.6, 28.0, 21.4; ^{19}F NMR (376 MHz, $CDCl_3$) δ -73.7; IR (neat): 2940 (bs), 1635, 1417, 1215, 1169, 1142, 705, 599, 564; HRESIMS Calcd for $[C_{32}H_{24}F_3NNaO_5S_2]^+$ (M + Na^+) 646.0940, found 646.0945.

(*S*)-10-(1-tosylindolin-7-yl)phenanthren-9-yl trifluoromethanesulfonate (2ae)

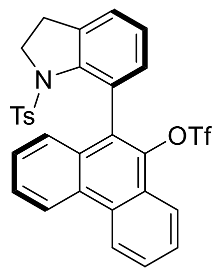

2ae

Compound **2ae** was prepared in 73% yield (43.6 mg) according to the general procedure at 40 °C as a pale yellow oil. $[\alpha]_D^{20}$ =+256.3 (c = 1.0, $CHCl_3$). 90% ee (determined by HPLC: Chiralpak ODH Column, 15/85 ^{i}PrOH/hexane, 1.0 mL/min, 254 nm; TR = 8.66 min (minor), 9.57 min (major)). ^{1}H NMR (400 MHz, $CDCl_3$) δ 8.73 (d, J = 8.4 Hz, 2H), 8.10 (d, J = 8.4 Hz, 1H), 8.03 (d, J = 8.0 Hz, 1H), 7.75 – 7.71 (m, 2H), 7.68 – 7.63 (m, 2H), 7.38 – 7.35 (m, 1H), 7.32 – 7.30 (m, 2H), 7.07 (d, J = 8.0 Hz, 2H), 6.87 (d, J = 8.0 Hz, 2H), 4.37 – 4.32 (m, 1H), 4.03 – 3.95 (m, 1H), 2.68 – 2.59 (m, 2H), 2.21 (s, 3H); ^{13}C NMR (100 MHz, $CDCl_3$) δ 143.3, 142.5, 140.7, 138.4, 135.8, 132.7, 131.6, 129.9, 129.5, 129.0, 128.2, 127.8, 127.4, 127.2, 126.4, 126.2, 125.9, 125.5, 125.3, 122.8, 122.7, 122.4, 118.2 (q, J = 320.4 Hz), 52.5, 29.8, 21.4; ^{19}F NMR (376 MHz, $CDCl_3$) δ -73.7; IR (neat): 2924 (bs), 1598, 1417, 1358, 1137, 975, 828, 734; HRESIMS Calcd for $[C_{30}H_{22}F_3NNaO_5S_2]^+$ (M + Na^+) 620.0784, found 620.0786.

(*S*)-1-(1-tosylindolin-7-yl)-5,6,7,8-tetrahydronaphthalen-2-yl trifluoromethanesulfonate (2af)

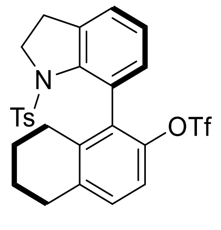

2af

Compound **2af** was prepared in 53% yield (48.8 mg) according to the general procedure at 40 °C as a pale yellow oil. $[\alpha]_D^{20}$ = +98.5° (c = 1.0, $CHCl_3$). 98% ee (determined by HPLC: Chiralpak ADH Column, 10/90 ^{i}PrOH/hexane, 1.0 mL/min, 254 nm; TR = 6.95 min (minor), 7.80 min (major)). ^{1}H NMR (400 MHz, $CDCl_3$) δ 7.34 (d, J = 8.0 Hz, 2H),

7.22 – 7.10 (m, 6H), 7.04 (d, *J* = 8.4 Hz, 1H), 4.06 – 3.94 (m, 2H), 2.90 – 2.81 (m, 2H), 2.76 – 2.61 (m, 2H), 2.38 (s, 3H), 2.35 – 2.27 (m, 2H), 1.86 – 1.73 (m, 4H); ^{13}C NMR (100 MHz, $CDCl_3$) δ 145.2, 143.8, 141.4, 138.7, 138.1, 137.5, 135.3, 132.6, 131.0, 129.6, 129.3, 127.9, 127.3, 126.4, 124.8, 118.3 (q, *J* = 320.1 Hz), 117.4, 52.4, 29.6, 29.1, 28.3, 22.7, 22.4, 21.5; ^{19}F NMR (376 MHz, $CDCl_3$) δ -74.6; IR (neat): 2945 (bs), 1694, 1422, 1377, 1150, 974, 836, 707, 534; HRESIMS Calcd for $[C_{26}H_{24}F_3NNaO_5S_2]^+$ (M + Na^+) 574.0940, found 574.0942.

(*S*)-7-(2-iodonaphthalen-1-yl)-1-tosylindoline (2ag)

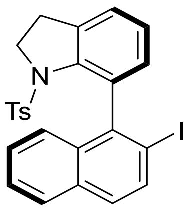

2ag

Compound **2ag** was prepared in 79% yield (48.8 mg) according to the general procedure at 40 °C as a pale yellow oil. $[\alpha]_D^{20}$ = +34.6° (c = 1.0, $CHCl_3$). 25% ee (determined by HPLC: Chiralpak ADH Column, 30/70 ^{i}PrOH/hexane, 1.0 mL/min, 254 nm; TR = 6.65 min (minor), 7.54 min (major)). ^{1}H NMR (400 MHz, $CDCl_3$) δ 7.86 (d, *J* = 8.8 Hz, 1H), 7.80 (d, *J* = 8.0 Hz, 1H), 7.68 (d, *J* = 8.4 Hz, 1H), 7.49 – 7.45 (m, 2H), 7.41 – 7.38 (m, 1H), 7.29 – 7.26 (m, 4H), 7.16 – 7.14 (m, 1H), 7.05 (d, *J* = 8.0 Hz, 2H), 4.20 – 4.04 (m, 2H), 2.69 – 2.61 (m, 1H), 2.57 – 2.50 (m, 1H), 2.35 (s, 3H); ^{13}C NMR (100 MHz, $CDCl_3$) δ 143.2, 142.2, 141.5, 138.2, 136.0, 135.6, 134.9, 133.2, 132.8, 131.4, 129.1, 128.8, 127.9, 127.3, 127.0, 126.3, 126.1, 126.0, 124.8, 99.0, 52.5, 29.7, 21.5; IR (neat): 2945 (bs), 1645, 1355, 1176, 1123, 884, 790, 584, 533; HRESIMS Calcd for $[C_{25}H_{20}INNaO_2S]^+$ (M + Na^+) 548.0152, found 548.0149.

(*S*)-7-(2-isopropylnaphthalen-1-yl)-1-tosylindoline (2ah)

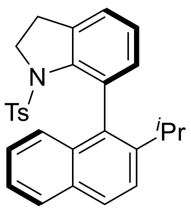

2ah

Compound **2ah** was prepared in 72% yield (48.8 mg) according to the general procedure at 40 °C as a pale yellow oil. $[\alpha]_D^{20}$ = -43.8° (c = 1.0, $CHCl_3$). 35% ee (determined by HPLC: Chiralpak ADH Column, 15/85 ^{i}PrOH/hexane, 1.0 mL/min, 254 nm; TR = 5.04 min (minor), 6.10 min (major)). ^{1}H NMR (400 MHz, $CDCl_3$) δ 7.81 – 7.75 (m, 2H), 7.56 (dd, *J* = 8.4, 7.6 Hz, 1H), 7.48 (d, *J* = 8.8 Hz, 1H), 7.38 – 7.29 (m, 2H), 7.23 – 7.20 (m, 2H), 7.17 – 7.14 (m, 1H), 7.03 – 6.95 (m, 4H), 4.13 – 4.07 (m, 1H), 4.00 – 3.93 (m, 1H), 2.94 – 2.87 (m, 1H), 2.73 – 2.64 (m, 2H), 2.31 (s, 3H), 1.31 (d, *J* = 6.8 Hz, 3H), 1.09 (d, *J* = 6.8 Hz, 3H); ^{13}C NMR (100 MHz, $CDCl_3$) δ 143.5, 142.9, 142.3, 137.6, 136.2, 134.2, 132.4, 132.0, 131.9, 131.8, 129.0, 128.0, 127.7, 126.8, 126.7, 125.9, 125.3, 124.5, 124.0, 123.8, 52.7, 30.8, 30.0, 25.2, 22.4, 21.4; IR (neat): 2962 (bs), 1644, 1361, 1279, 1166, 819, 724, 672; HRESIMS Calcd for $[C_{28}H_{27}NNaO_2S]^+$ (M + Na^+) 464.1655, found 464.1654.

(*S*)-7-(2-phenylnaphthalen-1-yl)-1-tosylindoline (2ai)

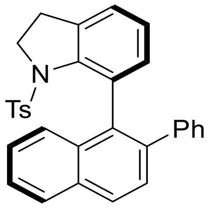

2ai

Compound **2ai** was prepared in 76% yield (48.8 mg) according to the general procedure at 40 °C as a pale yellow oil. $[\alpha]_D^{20}$ = +461.2° (c = 1.0, $CHCl_3$). 46% ee (determined by HPLC: Chiralpak ADH Column, 15/85 ^{i}PrOH/hexane, 1.0 mL/min, 254 nm; TR = 8.98 min (minor), 11.20 min (major)). ^{1}H NMR (400 MHz, $CDCl_3$) δ 8.14 – 8.12 (m, 1H), 7.92 – 7.89 (m, 2H), 7.53 – 7.48 (m, 2H), 7.45 (d, *J* = 8.4 Hz, 1H), 7.39 (d, *J* = 7.6 Hz, 1H), 7.21 – 7.18 (m, 1H), 7.11 – 7.01 (m, 8H), 6.92 – 6.89 (m, 2H), 3.78 – 3.72 (m,

1H), 2.45 – 2.37 (m, 1H), 2.32 (s, 3H), 2.29 – 2.20 (m, 1H), 2.14 – 2.09 (m, 1H); ^{13}C NMR (100 MHz, $CDCl_3$) δ 143.1, 142.6, 141.7, 138.1, 137.8, 135.9, 135.2, 133.0, 132.7, 132.2, 132.1, 129.9, 129.0, 128.2, 127.9, 127.7, 127.1, 127.0, 125.9, 125.6, 125.5, 125.4, 123.8, 51.6, 29.2, 21.5; IR (neat): 2940 (bs), 1417, 1215, 1169, 1142, 705, 599, 565; HRESIMS Calcd for $[C_{31}H_{25}NNaO_2S]^+$ (M + Na^+) 498.1498, found 498.1494.

3-methoxy-2-(1-tosylindolin-7-yl)phenyl 2,4,6-trimethylbenzenesulfonate (2aj)

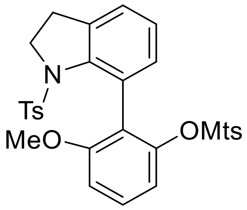

2aj

Compound **2aj** was prepared in 30% yield (40.3 mg) according to the general procedure at 40 °C as a pale yellow oil. <5% ee (determined by HPLC: Chiralpak ODH Column, 30/70 ^{i}PrOH/hexane, 1.0 mL/min, 254 nm; TR = 7.65 min, 8.86 min) ^{1}H NMR (400 MHz, $CDCl_3$) δ 7.34 (d, *J* = 8.0 Hz, 2H), 7.25 – 7.21 (m, 1H), 7.14 (d, *J* = 7.6 Hz, 1H), 7.09 – 7.04 (m, 3H), 6.97 (d, *J* = 7.2 Hz, 1H), 6.90 (d, *J* = 8.4 Hz, 1H), 6.87 (s, 2H), 6.68 (d, *J* = 8.4 Hz, 1H), 4.05 – 3.98 (m, 1H), 3.83 (s, 3H), 3.77 – 3.69 (m, 1H), 2.41 (s, 6H), 2.34 (s, 3H), 2.30 (s, 3H), 2.24 – 2.21 (m, 2H); ^{13}C NMR (100 MHz, $CDCl_3$) δ 158.4, 147.5, 143.4, 143.0, 141.3, 139.8, 137.1, 135.4, 132.5, 131.5, 129.1, 128.6, 127.4, 126.4, 125.9, 124.0, 123.7, 113.6, 109.4, 56.3, 52.3, 29.0, 22.6, 21.5, 21.0; IR (neat): 2940 (bs), 1635, 1361, 1195, 1167, 614, 580, 492; HRESIMS Calcd for $[C_{31}H_{31}NNaO_6S_2]^+$ (M + Na^+) 600.1485, found 600.1489.

5. Racemization Experiments

Since the configurational stability of axially chiral 7-aryl indolines is one of the key factors for their utility, the racemization experiments of selected compounds were performed at 100 °C.

Compound **2a**, **2o**, **2ac** or **2af** (1 mg) was dissolved in toluene (1 mL) and stirred at 100 °C for 10–30 h. At given interval of time, small samples (20 μL) of this solution were removed via syringe, diluted by *i*PrOH (0.5 mL) and subjected into the HPLC to measure the enantiomeric excess. Importantly, 5,6,7,8-tetrahydronaphthyl product **2af** demonstrated highest rotational barrier (129.43 kJ/mol) and longest half-life at 100 °C (16.5 h). Thus, the steric effect of different substituents on naphthalene rings is crucial, and details are given below.

Compound **2a**: solvent: toluene; temperature: 373K.

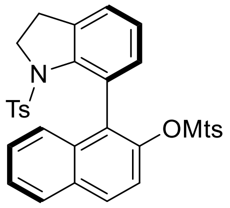

t (s)	ee of **2a** (%)	$\ln(ee_0/ee_t)$
0	93.92	0
1800	70.34	0.2891027
3600	52.48	0.5820112
7200	32.3	1.0673761
10800	19.36	1.5792343
18000	5.46	2.8449946
36000	1.26	4.3113316

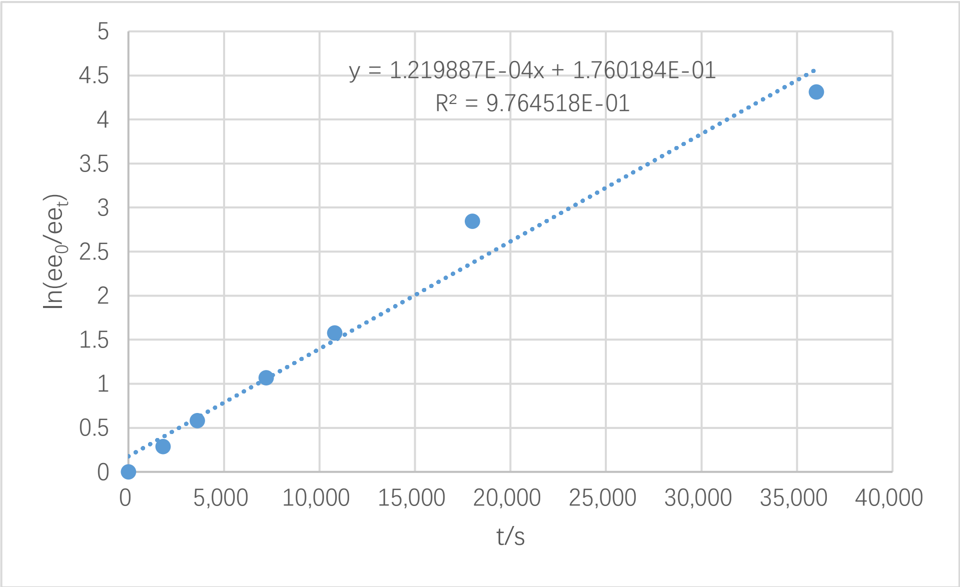

K racemization = 0.0001219887

K enantiomerization = 0.0000609944

$t_{1/2}$ = 1.6 h

$\Delta G^{\ddagger}$ = 122.14 kJ/mol

Compound **2o**: solvent: toluene; temperature: 373K.

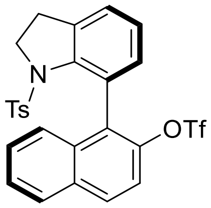

t (s)	ee of **2o** (%)	$\ln(ee_0/ee_t)$
0	94.5	0
1800	70.3	0.295828
3600	55.14	0.5387244
7200	35.1	0.9903987
10800	23.46	1.393303
18000	10.18	2.2281748
36000	1.38	4.2265163

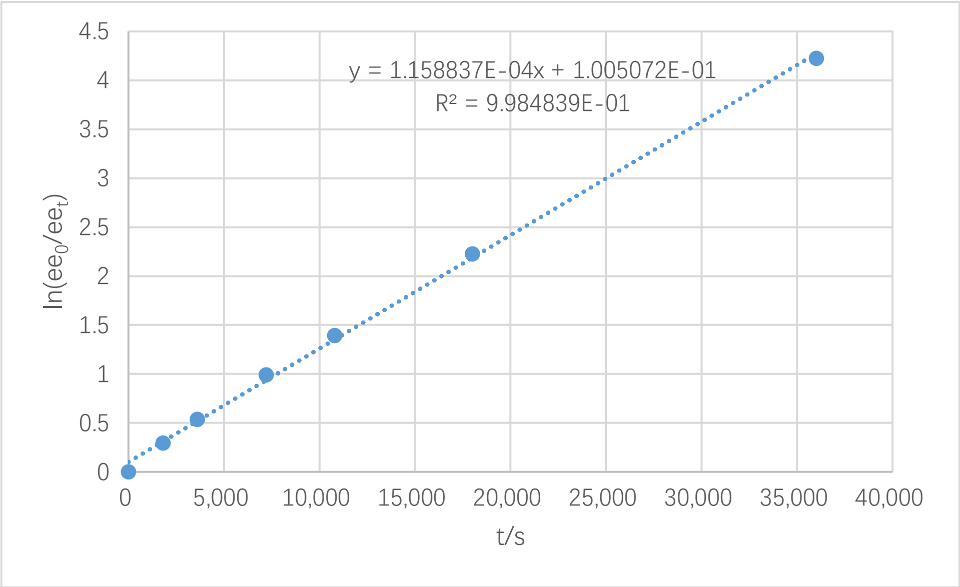

K racemization = 0.0001158837

K enantiomerization = 0.0000579419

$t_{1/2}$ = 1.7 h

$\Delta G^{\ddagger}$ = 122.31 kJ/mol

Compound **2ac**: solvent: toluene; temperature: 373K.

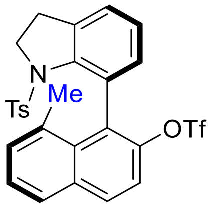

t (s)	ee of **2ae** (%)	$\ln(ee_0/ee_t)$
0	95.04	0
1800	80.04	0.1717713
3600	55.38	0.5400793
7200	41.3	0.8334354
10800	21.96	1.4650752
18000	6.52	2.6794235
36000	0.56	5.1341164

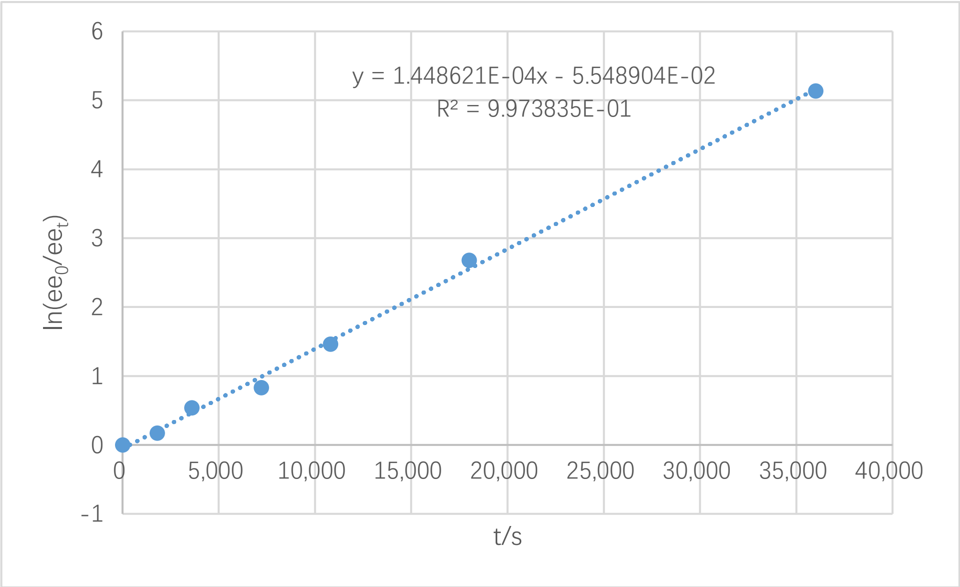

K racemization = 0.0001448621

K enantiomerization = 0.0000724311

$t_{1/2}$ = 1.3 h

$\Delta G_{\ddagger}$ = 121.60 kJ/mol

Compound **2af**: solvent: toluene; temperature: 373K.

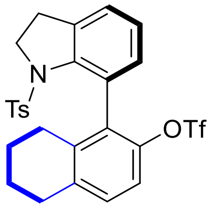

t (s)	ee of **2af** (%)	$\ln(ee_0/ee_t)$
0	98.58	0
1800	96.8	0.0182214
3600	94.06	0.0469355
7200	90.66	0.0837522
10800	83.7	0.1636294
18000	79.62	0.2136031
36000	64.84	0.4189457
72000	34.32	1.0551401
108000	31.08	1.1543039

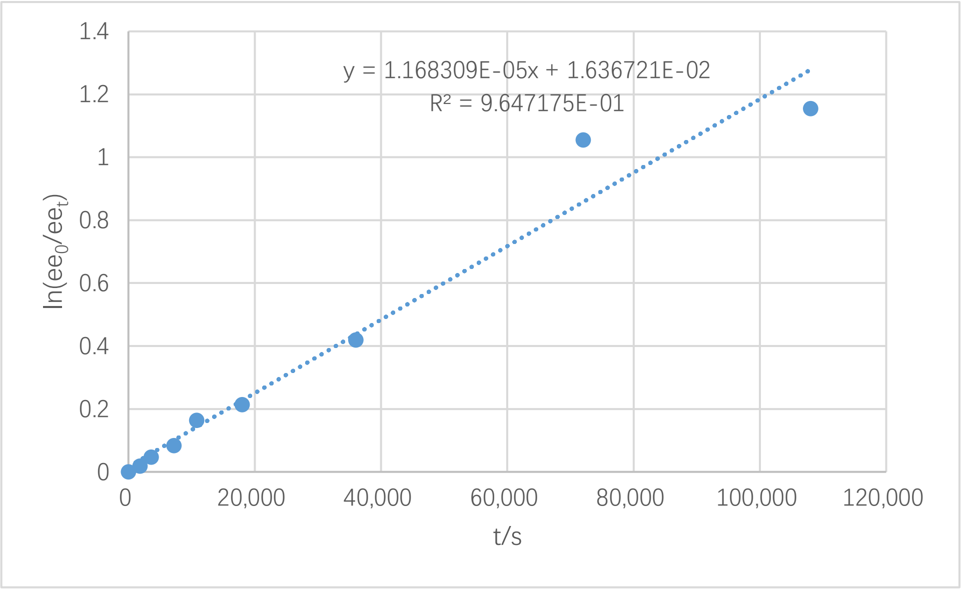

K racemization = 0.00001168309

K enantiomerization = 0.00000584155

$t_{1/2}$ = 16.5 h

$\Delta G^{\ddagger}$ = 129.43 kJ/mol

6. Synthetic Utility Study

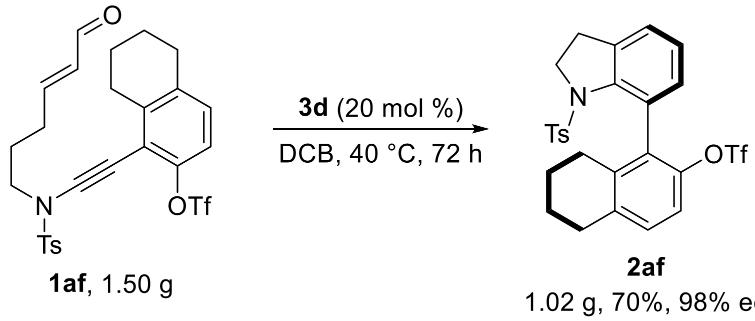

1af, 1.50 g

2af
1.02 g, 70%, 98% ee

To a 50 mL vial charged with a stir bar were added ynamide **1af** (1.50 g, 2.63 mmol), 1,2-dichlorobenzene (26 mL) and chiral secondary amine catalyst **3d** (0.53 mmol, 268 mg) sequentially. The reaction mixture was stirred at 40 ºC for 72 h, and the progress of the reaction was monitored by TLC. Upon complection, the reaction mixture was directly purified by column chromatography on silica gel (eluent: PE/EtOAc= 6/1) to afford the desired **2af** in 70% yield with 98% ee (1.02 g).

(*S*)-diphenyl(1-(1-tosylindolin-7-yl)-5,6,7,8-tetrahydronaphthalen-2-yl)phosphine oxide (4)

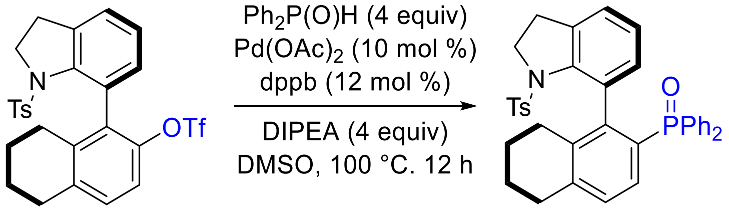

2af
(98% ee)

4, 51%, 91% ee
(99% ee after recrystalization)

Compound **4** was prepared according to the known procedure (2 mmol scale)[1] in 51% yield (615 mg) as a white solid (mp 241–243 ºC). $[\alpha]_D^{20}$ = +127.1º (c = 1.0, $CHCl_3$). 91% ee (determined by HPLC: Chiralpak ODH Column, 10/90 *i*-PrOH/hexane, 1.0 mL/min, 254 nm; TR = 5.48 min (minor), 6.04 min (major)). ^{1}H NMR (400 MHz, $CDCl_3$) δ 7.78 (d, *J* = 8.1 Hz, 2H), 7.65 – 7.58 (m, 2H), 7.53 – 7.31 (m, 8H), 7.16 (d, *J* = 8.1 Hz, 2H), 6.98 (d, *J* = 7.0 Hz, 2H), 6.91 – 6.79 (m, 2H), 6.62 (d, *J* = 7.6 Hz, 1H), 3.98 – 3.84 (m, 2H), 2.91 – 2.76 (m, 3H), 2.34 (s, 3H), 2.37 – 2.27 (m, 2H), 2.22 – 2.13 (m, 1H), 1.84 – 1.76 (m, 3H), 1.70 – 1.65 (m, 1H); ^{13}C NMR (100 MHz, $CDCl_3$) δ 144.2 (d, *J* = 6.7 Hz), 142.9, 141.3, 141.1 (d, *J* = 2.3 Hz), 137.8 (d, *J* = 10.0 Hz), 137.3, 135.9,

135.4, 134.4, 132.2, 132.1, 131.7 (d, J = 9.3 Hz), 130.9, 130.7 (d, J = 13.6 Hz), 130.5, 129.3, 129.2, 128.0, 127.9 (d, J = 8.2 Hz), 127.6, 127.2 (d, J = 14.0 Hz), 126.6, 125.0, 123.8, 52.3, 30.2, 28.9, 27.6, 23.1, 22.4, 21.4; ^{31}P NMR (162 MHz, $CDCl_3$) δ 30.1; IR (neat): 2934 (bs), 1598, 1436, 1356, 1167, 909, 701, 539; HRESIMS Calcd for $[C_{37}H_{34}NNaO_3PS]^+$ (M + Na^+) 626.1889, found 626.1897.

(*S*)-7-(2-(diphenylphosphanyl)-5,6,7,8-tetrahydronaphthalen-1-yl)-1-tosylindoline (5)

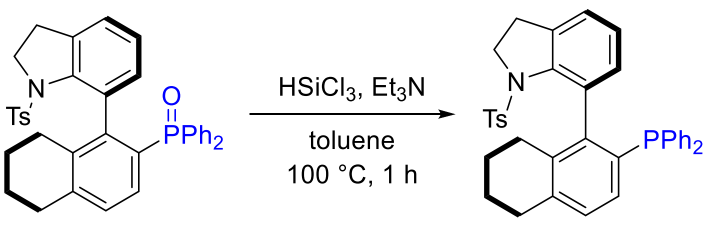

Compound **5** was prepared according to the known procedure (1 mmol scale)[5] in 68% yield (400 mg). The product was isolated through neutral Al_2O_3 column chromatography (PE/EA = 30/1) as a white solid (mp 204–206 °C). $[\alpha]_D^{20}$ = +76.5° (c = 1.0, $CHCl_3$). 99% ee (determined by HPLC: Chiralpak IA Column, 20/80 *i*-PrOH/hexane, 1.0 mL/min, 254 nm; TR = 4.30 min (major), 5.20 min (minor)). ^{1}H NMR (400 MHz, $CDCl_3$) 7.54 (d, J = 7.6 Hz, 2H), 7.31 – 7.24 (m, 5H), 7.24 – 7.14 (m, 5H), 7.13 (d, J = 8.0 Hz, 2H), 7.05 (d, J = 7.2 Hz, 1H), 7.00 (d, J = 8.0 Hz, 1H), 6.90 – 6.87 (m, 2H), 6.63 (d, J = 7.6 Hz, 1H), 4.07 – 4.00 (m, 1H), 3.95 – 3.88 (m, 1H), 2.81 (t, J = 4.8 Hz, 2H), 2.76 – 2.71 (m, 1H), 2.47 – 2.39 (m, 1H), 2.37 (s, 3H), 2.34 – 2.28 (m, 2H), 1.78 – 1.70 (m, 3H), 1.68 – 1.63 (m, 1H); ^{13}C NMR (100 MHz, $CDCl_3$) δ 145.7, 145.4, 143.2, 140.9, 138.2, 137.5, 136.1, 135.7 (d, J = 6.0 Hz), 134.2, 134.0, 133.2, 133.1, 131.6, 131.4 (d, J = 3.3 Hz), 129.3, 128.5, 128.2, 128.1, 128.0 (d, J = 5.4 Hz), 127.6 (d, J = 4.3 Hz), 127.4, 125.4, 123.9, 52.5, 30.1, 29.4, 27.8, 23.3, 22.7, 21.5. ^{31}P NMR (162 MHz, $CDCl_3$) δ -12.6; IR (neat): 2934 (bs), 1433, 1357, 1163, 741, 698, 670; HRESIMS Calcd for $[C_{37}H_{34}N_2NaO_2PS]^+$ (M + Na^+) 610.1940, found 610.1945.

di-*tert*-butyl (*R*,*E*)-2-(1,3-diphenylallyl)malonate (8)

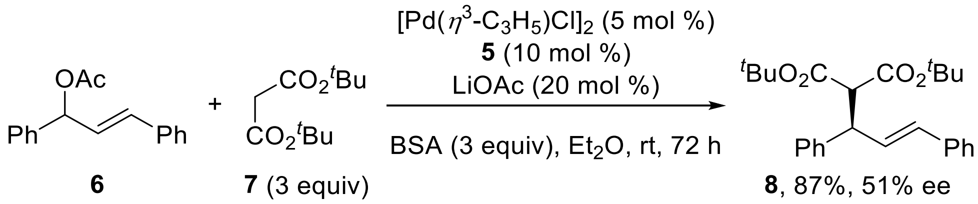

Compound **8** was prepared in 87% yield (35.6 mg) according to the known procedure (0.10 mmol scale)[6] by replacing ligand with compound **5**. This compound is known and the spectroscopic data match those reported. 51% ee. (determined by HPLC: Chiralpak ADH Column, 10/90 *i*-PrOH/hexane, 1.0 mL/min, 254 nm; TR = 5.95 min (major), 7.92 min (minor)). ^{1}H NMR (400 MHz, $CDCl_3$) δ 7.34 – 7.28 (m, 7H), 7.24 – 7.14 (m, 3H), 6.44 (d, *J* = 15.6 Hz, 1H), 6.36 – 6.30 (m, 1H), 4.15 (dd, *J* = 10.8, 8.4 Hz, 1H), 3.73 (d, *J* = 10.8 Hz, 1H), 1.42 (s, 9H), 1.22 (s, 9H); ^{13}C NMR (100 MHz, $CDCl_3$) δ 167.3, 166.8, 140.8, 137.0, 131.2, 130.1, 128.5, 128.4, 128.2, 127.3, 126.8, 126.3, 81.8, 81.6, 59.3, 49.0, 27.9, 27.6.

7. Crystal data of compound 2o. CCDC Number = 2234664.

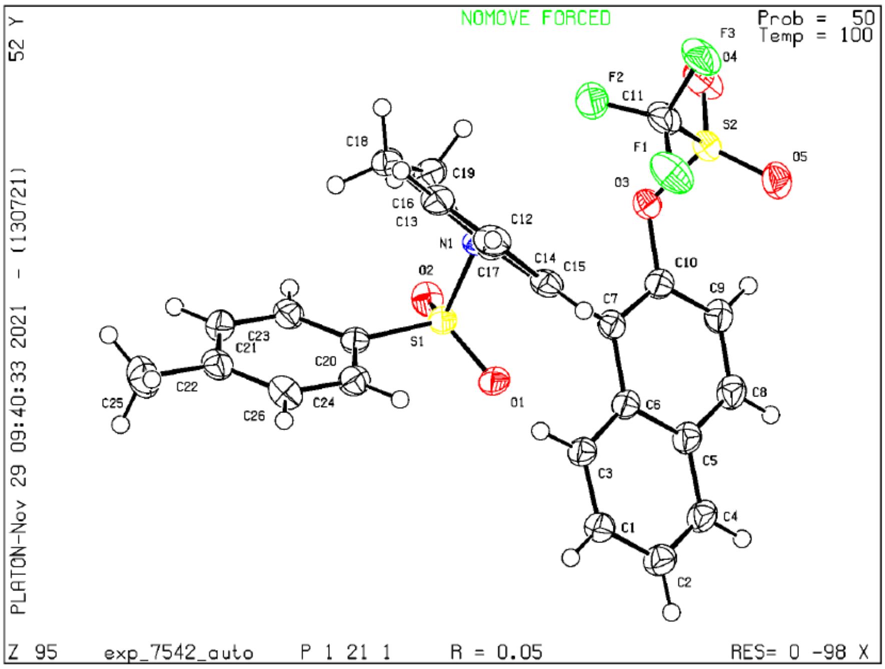

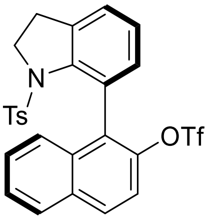

2o

Bond precision: C-C = 0.0062 A Wavelength=1.54184

Cell:	a=6.9978(1)	b=14.4109(3)	c=12.2598(2)
	alpha=90	beta=91.455(2)	gamma=90
Temperature:	100 K		

	Calculated	Reported
Volume	1235.94(4)	1235.94(4)
Space group	P 21	P 1 21 1
Hall group	P 2yb	P 2yb
Moiety formula	C26 H20 F3 N O5 S2	C26 H20 F3 N O5 S2
Sum formula	C26 H20 F3 N O5 S2	C26 H20 F3 N O5 S2
Mr	547.55	547.55
Dx,g cm-3	1.471	1.471
Z	2	2
Mu (mm-1)	2.505	2.505
F000	564.0	564.0
F000’	567.21	
h,k,lmax	8,18,15	8,18,15
Nref	5091[2650]	4648
Tmin,Tmax	0.942,0.951	0.181,1.000
Tmin’	0.882	

Correction method= # Reported T Limits: Tmin=0.181 Tmax=1.000
AbsCorr = MULTI-SCAN

Data completeness= 1.75/0.91 Theta(max)= 75.214

R(reflections)= 0.0478(4522) wR2(reflections)= 0.1351(4648)

S = 1.097 Npar= 335

8. Computational Studies

All calculations were performed using Gaussian 16 package.[7] Geometry optimizations and vibration frequencies were calculated by using B3LYP-D3(BJ) level of density function theory[8–10] with the 6-31G(d,p) basis set[11,12] for all the atoms in the system. All local minimums were confirmed with no imaginary frequency and all transition states had only one imaginary frequency. And every transition state was checked by intrinsic reaction coordinate (IRC). The SMD solvation model[13] with DCB was used for all calculations. The ball stick models of molecules were drawn by CYLview 2.0.[14]

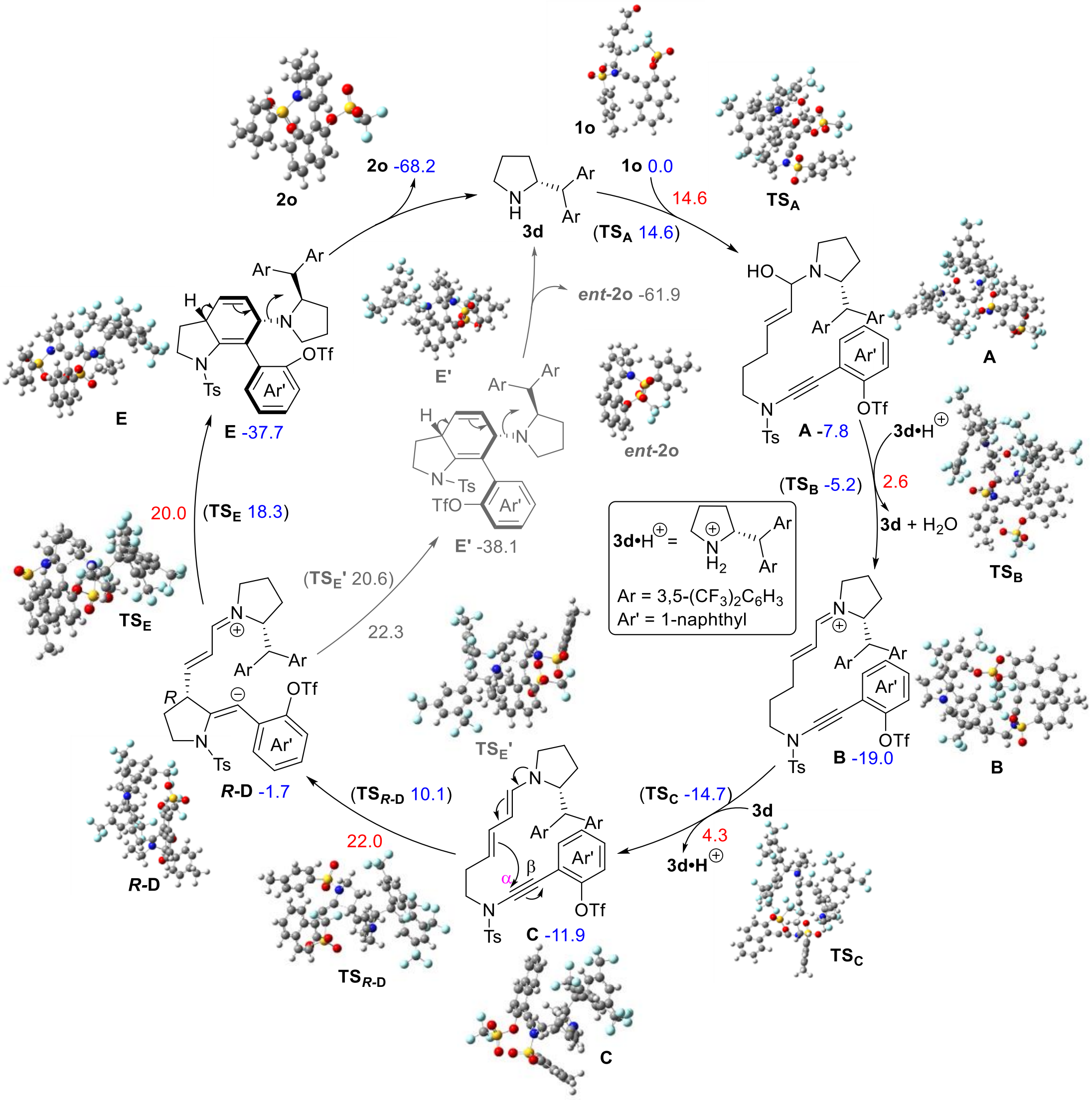

Figure S1. Plausible reaction mechanism (through the pathway of ***R*-D**). Relative free energies (ΔG, in kcal/mol) of all the transition states and intermediates were computed at the SMD(DCB)-B3LYP-D3BJ/6-31G(d,p) level of theory.

Our DFT calculations gave a clear explanation for the mechanism of (4 + 2) annulation for the synthesis of axially chiral 7-aryl indoline **2o** (Figure S1). The substrate **1o** and chiral secondary amine catalyst **3d** undergo condensation and isomerization to form dienamine intermediate **C** (ΔG= -11.9 kcal/mol). Subsequently, an intramolecular nucleophilic addition takes place to deliver the major vinyl anion intermediate ***R*-D** through transition state **TS*R*-D** with a free energy barrier of 22.0 kcal/mol, which is kinetically and thermodynamically more favorable than the minor vinyl anion intermediate ***S*-D** (with a free energy barrier of 24.6 kcal/mol through **TS*S*-D**, Figure S2). This first cyclization step is considered as the rate-determining step.

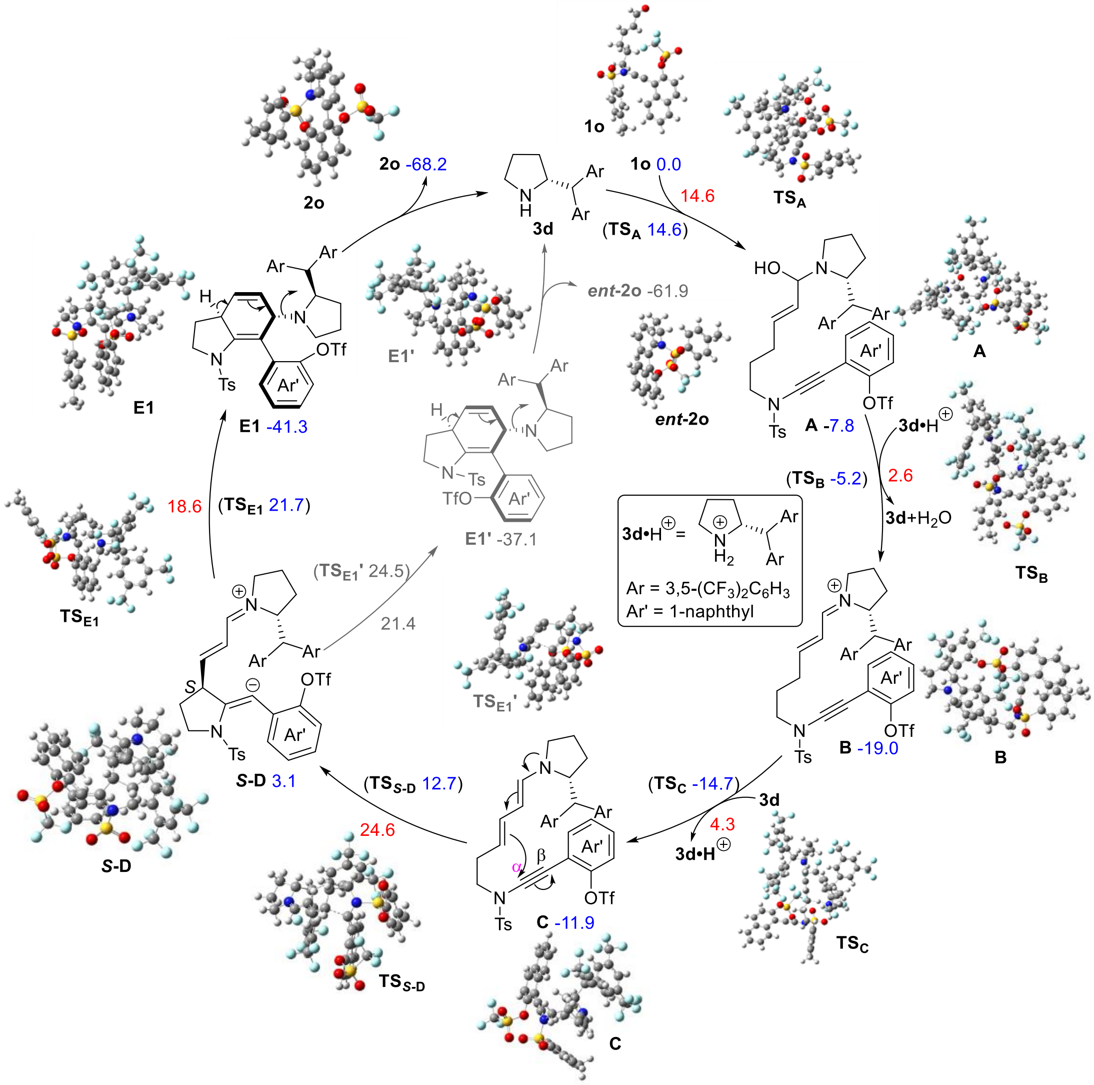

Figure S2. Plausible reaction mechanism (through the pathway of ***S*-D**). Relative free energies (ΔG, in kcal/mol) of all the transition states and intermediates were computed at the SMD(DCB)-B3LYP-D3BJ/6-31G(d,p) level of theory.

Further cyclization of intermediate ***R*-D** forms the axially chiral intermediate **E** through **TS$_E$** with a free energy barrier of 20.0 kcal/mol. Finally, aromatization of **E** furnishes axially chiral 7-aryl indoline **2o** and regenerates catalyst **3d**. The calculated free energy of the reaction is -68.2 kcal/mol.

To find out the enantio-determining step, we performed more theoretical calculations for the first cyclization step (from intermediate **C** to intermediate **D**), which produced a new chiral center. The optimized structures and relative free energies of the enantiomeric transition states **TS$_{R\text{-D}}$** and **TS$_{S\text{-D}}$** are shown in Figure S3. The free energy of transition state **TS$_{R\text{-D}}$** is 2.6 kcal/mol lower than **TS$_{S\text{-D}}$**, indicating that the intermediate ***R*-D** is the major intermediate. Thus, the enantioselectivity of this cyclization could be well controlled and the theoretically predicted ratio of **TS$_{R\text{-D}}$** and **TS$_{S\text{-D}}$** is 98.3:1.7 (e.r.). It should be noted that, both **TS$_{R\text{-D}}$** and **TS$_{S\text{-D}}$** could lead to the enantioselective generation **2o** and ***ent*-2o**. Therefore, the first cyclization step is not the enantio-determining step.

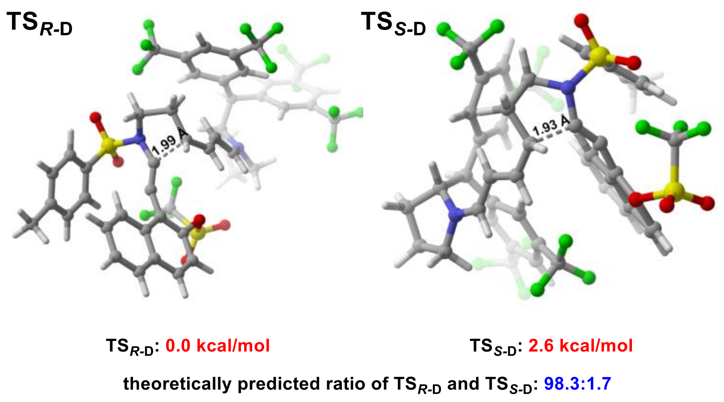

Figure S3. Optimized structures and relative free energies of the enantiomeric transition states **TS$_{R\text{-D}}$** and **TS$_{S\text{-D}}$**.

Further theoretical calculations have been conducted to explain the origin of enantioselectivity in the enantio-determining cyclization step (from intermediate **D** to intermediate **E**). The optimized structures and relative free energies of the enantiomeric transition states are shown in Figure S4. Starting from intermediate ***R*-D**, the free energy of transition state **TS$_E$** (leading to the major enantiomer) is 2.3 kcal/mol lower than **TS$_E$′** (leading to the minor enantiomer) and the theoretically predicted enantioselectivity matches well with the experimental ee value (94.6% *versus* 96%).

Inspection of the structures of transition states shows that **TS$_E$′** has a shorter C···C distance than **TS$_E$** (2.14 Å *versus* 2.30 Å), suggesting that **TS$_E$′** has stronger steric repulsion and lower stability. **TS$_E$** also possesses a π-π stacking between the naphthyl and Ts group, making **TS$_E$** more stable. Thus, the observed enantioselectivity originates from steric effects and π-π stacking effect. On the other hand, the enantiomeric transition states starting from ***S*-D** were also calculated. The free energy of transition state **TS$_{E1}$** (leading to the major enantiomer) is 2.8 kcal/mol lower than **TS$_{E1}$′** (leading to the minor enantiomer) and the theoretically predicted enantioselectivity also matches well with the experimental ee value (97.4% *versus* 96%). In conclusion, the observed enantioselectivity is majorly controlled by the ***R*-D** pathway, and the ***S*-D** pathway has much smaller contribution due to the enantioinduction in the first cyclization step (Figure S3).

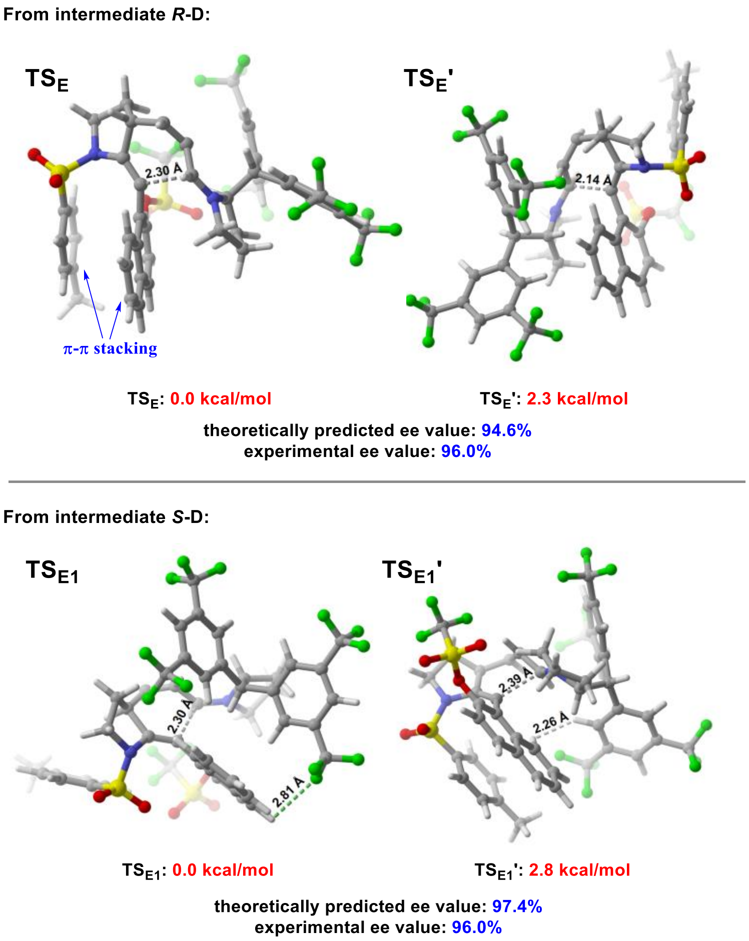

Figure S4. Optimized structures and relative free energies of the enantiomeric transition states.

XYZ Coordinates

1o

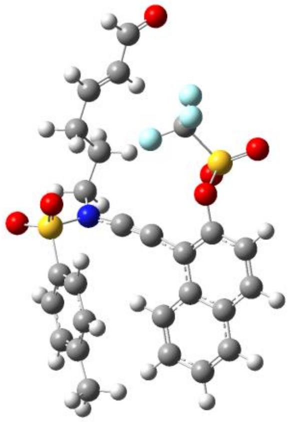

N	0.04350400	-2.08297000	-0.77566500
S	0.65198000	-3.16665300	0.41288500
O	0.56295900	-4.50214200	-0.19452700
O	-0.01498700	-2.92381900	1.70154000
C	2.33725800	-2.62688800	0.54095100
C	2.70593900	-1.78913200	1.59551200
C	3.24214300	-2.99462400	-0.45512900
C	4.01146900	-1.31583300	1.64481000
H	1.98155400	-1.50888400	2.35017800
C	4.54571400	-2.51112400	-0.38279900
H	2.93611300	-3.64257600	-1.26869800
C	4.94753300	-1.66460200	0.65982500
H	4.30775700	-0.65520500	2.45354900
H	5.26051200	-2.79083700	-1.14993100
C	6.34730600	-1.11869700	0.72237500
H	6.33186700	-0.02416800	0.74573000
H	6.85660000	-1.45545400	1.63191500
H	6.93978900	-1.43606600	-0.13866500
C	0.28605700	-0.78504200	-0.54997300
C	0.59120600	0.36234400	-0.29218000
C	1.14021900	1.65722600	-0.10794700
C	2.50413700	1.93303400	-0.47816300
C	0.39586600	2.69500700	0.43029400
C	3.34722600	0.93166100	-1.01928100
C	3.02576800	3.25272700	-0.28688900
C	0.88846500	3.99754700	0.60559700
C	4.65135800	1.22187100	-1.35297200
H	2.95251000	-0.06676100	-1.16261200
C	4.37524200	3.51708500	-0.64086300

C	2.18982600	4.26713200	0.24957800
H	0.24153800	4.75620100	1.03000400
C	5.17254100	2.52331200	-1.16111800
H	5.28590100	0.44371300	-1.76398200
H	4.76358200	4.51990700	-0.49167700
H	2.59450800	5.26511700	0.38284600
H	6.20282400	2.73475700	-1.42834500
S	-2.16709900	2.59702200	-0.12197200
O	-1.85366100	2.04920100	-1.43583100
O	-2.74000400	3.93122100	0.00997600
C	-0.92034100	-2.52422200	-1.81479800
H	-0.57311000	-2.09720600	-2.75871400
H	-0.83020400	-3.60910500	-1.88269600
C	-2.35325000	-2.09596100	-1.52484800
H	-2.95890600	-2.37860300	-2.39325700
H	-2.39189100	-1.00382400	-1.45358900
C	-2.92180600	-2.73126500	-0.25848500
H	-2.86107400	-3.82735100	-0.32382500
H	-2.30078100	-2.47058800	0.60977600
C	-4.32670400	-2.35811600	0.07702700
H	-4.73020400	-2.81643600	0.98045100
C	-5.11508000	-1.50546300	-0.60093100
H	-4.79065900	-0.99718400	-1.50372400
C	-6.44337000	-1.18692300	-0.09963800
H	-6.74172900	-1.72787800	0.82174400
O	-7.20787800	-0.37815100	-0.62159200
O	-0.90648600	2.41892400	0.90592200
C	-3.27271800	1.39702100	0.79221400
F	-3.48135800	1.84186200	2.02445600
F	-4.42187800	1.33619800	0.12631700
F	-2.69350700	0.20338300	0.82829900

TS$_A$

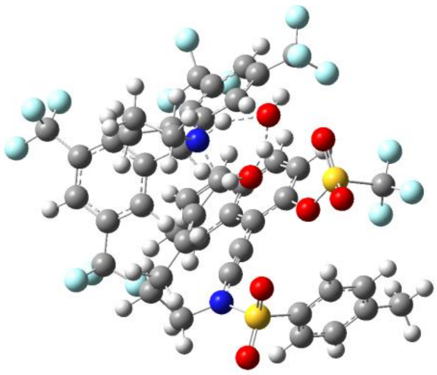

N	2.09795100	-3.57559000	-0.29183600
S	3.69106700	-3.66121600	0.36925600
O	4.13503500	-5.03762800	0.09025700
O	3.72081400	-3.20083700	1.76295800
C	4.54413300	-2.48259300	-0.64264500
C	5.17508500	-1.40724700	-0.02568100
C	4.56309900	-2.65066900	-2.03125600
C	5.83382900	-0.47332800	-0.82250200
H	5.12772800	-1.28623000	1.04808500
C	5.21489900	-1.70152800	-2.80561500
H	4.06449300	-3.49408500	-2.49568200
C	5.85256800	-0.59626200	-2.21541700
H	6.32299400	0.37277100	-0.35210600
H	5.22596900	-1.81096900	-3.88563400
C	6.51324000	0.44579900	-3.07572700
H	7.18312900	-0.01507300	-3.80837800
H	5.76147600	1.01212900	-3.63691800
H	7.08876300	1.15354400	-2.47528100
C	1.65349700	-2.37968200	-0.68969200
C	1.33324300	-1.27935600	-1.09510200
C	0.94164500	0.00615900	-1.54506900
C	-0.15144500	0.16973400	-2.46522800
C	1.60431300	1.14682600	-1.11262100
C	-0.84152400	-0.94304600	-3.00254000
C	-0.56040600	1.48496400	-2.84411400
C	1.22129200	2.44486300	-1.49673800
C	-1.92542200	-0.76820600	-3.83339600
H	-0.50566100	-1.93756500	-2.74433800
C	-1.66998600	1.63316900	-3.71703100
C	0.14153800	2.60560100	-2.33335700
H	1.74247800	3.31441500	-1.12194500
C	-2.35001600	0.53332700	-4.18812300

H	-2.45813600	-1.63571000	-4.20895400
H	-1.98227600	2.63426200	-3.99052400
H	-0.18476800	3.60227100	-2.60848200
H	-3.20768900	0.66235800	-4.84034200
S	3.35134600	1.88987800	0.77986400
O	4.21708600	1.08885400	1.62523700
O	2.33891500	2.78573000	1.31253400
C	1.23616200	-4.78324700	-0.30883900
H	0.79738000	-4.83963000	-1.30682600
H	1.90815700	-5.63457800	-0.19536100
C	0.14347600	-4.82393600	0.75908200
H	-0.37598300	-5.77684200	0.60878700
H	-0.60186600	-4.04901200	0.57004000
C	0.65122700	-4.74509600	2.20463500
H	-0.05235900	-5.27578300	2.86110400
H	1.59273500	-5.30167200	2.29750900
C	0.85321500	-3.36978600	2.78229700
H	1.43494800	-3.34253700	3.70281200
C	0.39352800	-2.21507400	2.29479900
H	-0.16013400	-2.19160800	1.36865500
C	0.68997300	-0.90433600	2.94751100
H	1.31241200	-1.08071500	3.83737400
O	1.23424000	0.04226000	2.11719700
O	2.75681200	0.88372500	-0.34188500
C	4.49377400	2.94452500	-0.30782400
F	5.68762500	2.99959700	0.27266200
F	4.60592700	2.41339500	-1.52209800
F	3.98063500	4.16903000	-0.40366800
C	-1.86662000	-0.01704900	2.82214100
C	-0.95060700	-0.98277600	4.87925500
C	-2.47169400	-1.09278100	4.86521800
C	-2.79700100	-1.10434500	3.37289500
H	-2.22766400	0.94376400	3.19861100
H	-0.49317600	-1.97246700	4.83569100
H	-0.54057100	-0.44068000	5.73200900
H	-2.81487400	-1.98820800	5.38723600
H	-2.92968700	-0.22101800	5.34289200
H	-2.55273100	-2.07373000	2.92650200
H	-3.84612700	-0.89060400	3.17141100
C	-1.72460100	0.11094000	1.25869800
H	-0.71473700	-0.19539500	0.98709800
C	-1.82862000	1.56879700	0.79327000
C	-2.67204000	1.96887500	-0.24748900
C	-1.00811300	2.53642700	1.38866100

C	-2.72942700	3.30497700	-0.64349700
H	-3.28752200	1.24990700	-0.76826600
C	-1.05956700	3.86577200	0.97329200
H	-0.32131000	2.25078700	2.17290000
C	-1.92803800	4.26651600	-0.03927700
H	-1.97189700	5.30280500	-0.35369200
C	-2.70323500	-0.80026000	0.53759700
C	-2.24279500	-1.85431900	-0.25488300
C	-4.08318600	-0.61049000	0.65063400
C	-3.14056000	-2.71646300	-0.88474300
H	-1.17923100	-1.99390300	-0.38899600
C	-4.97715000	-1.46936800	0.01230900
H	-4.46833800	0.21741600	1.23599600
C	-4.51675000	-2.53434400	-0.75536400
H	-5.21362600	-3.20197300	-1.24690900
C	-3.66100300	3.74349700	-1.73435800
C	-0.13433400	4.89835900	1.54830100
C	-6.44630000	-1.21827300	0.18607100
C	-2.66097600	-3.88069800	-1.70212700
F	-3.00293400	4.43716900	-2.70182800
F	-4.28569600	2.71278700	-2.33951900
F	-4.62432700	4.57430100	-1.26862700
F	-0.77314300	6.07188100	1.77038800
F	0.88878600	5.17901300	0.70363900
F	0.41468200	4.52259200	2.72536900
F	-6.81090400	-1.29974100	1.48945300
F	-7.20843100	-2.09575700	-0.49748400
F	-6.79066300	0.02384400	-0.22899700
F	-1.32231700	-3.86733000	-1.90937800
F	-2.94566100	-5.06199900	-1.10453200
F	-3.24643900	-3.91253100	-2.92261700
N	-0.57580500	-0.24948100	3.61649600
H	-0.12190600	0.70148900	3.86828500
O	1.11648800	1.66625600	3.91699900
H	1.01056900	2.57674700	3.61835900
H	1.33210500	0.90169900	2.82900600

A

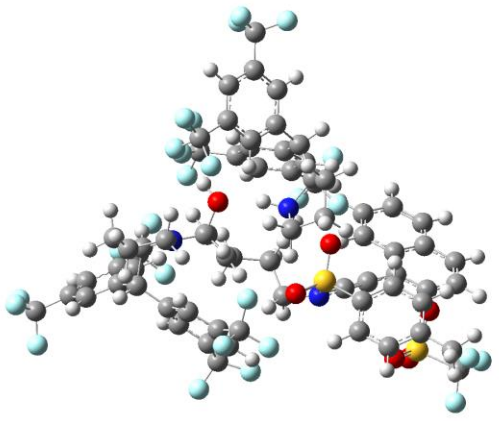

N	-2.88101300	-1.05047400	-0.17087800
S	-2.41858400	0.13619800	-1.34476600
O	-1.34791600	-0.48738500	-2.13591000
O	-2.10893900	1.42025300	-0.66900900
C	-3.86306000	0.35154000	-2.33229000
C	-4.83715300	1.27015700	-1.94039200
C	-3.98291800	-0.40775800	-3.50025600
C	-5.96537100	1.41832400	-2.74156800
H	-4.71936900	1.85002900	-1.03348300
C	-5.11395900	-0.23285400	-4.28571700
H	-3.19942900	-1.09781900	-3.79116100
C	-6.12156800	0.67358300	-3.91761900
H	-6.73879300	2.11904500	-2.44563200
H	-5.22067900	-0.80760900	-5.20027900
C	-7.34967200	0.82229900	-4.77221300
H	-7.07889400	1.02499300	-5.81329700
H	-7.93512100	-0.10293700	-4.76485200
H	-7.98949600	1.63255500	-4.41631300
C	-3.90144400	-0.68089200	0.62440200
C	-4.85012900	-0.34611900	1.30253700
C	-5.98852900	0.07160300	2.03621800
C	-5.93017400	0.35427900	3.44191900
C	-7.20358700	0.23843000	1.38899200
C	-4.72987200	0.21019900	4.17770900
C	-7.11239300	0.80562300	4.11070600
C	-8.37162900	0.67128700	2.03284100
C	-4.68899500	0.52443200	5.51712700
H	-3.84376500	-0.14168400	3.66701900
C	-7.03946200	1.10471300	5.49705800

C	-8.32084300	0.95270200	3.37927400
H	-9.28441100	0.77456200	1.45849900
C	-5.85399500	0.97294500	6.18412800
H	-3.75570700	0.42859600	6.06226300
H	-7.93864600	1.44358200	6.00246500
H	-9.21090300	1.29290200	3.89842700
H	-5.80923400	1.21136100	7.24184000
S	-7.41564300	-1.50206900	-0.56537000
O	-7.63008400	-2.42648500	0.53935100
O	-6.41592600	-1.74399000	-1.59249300
C	-1.76683800	-1.85477100	0.40785600
H	-2.23815700	-2.74181600	0.83199500
H	-1.17743600	-2.18018900	-0.44444700
C	-0.91343700	-1.10560100	1.44388600
H	-1.31844700	-1.26536500	2.44573100
H	-0.96814100	-0.03157400	1.25015300
C	0.56317000	-1.54171000	1.38254400
H	1.11018800	-1.16060500	2.24974500
H	0.61473900	-2.63636000	1.43440600
C	1.19777100	-1.05562900	0.11443200
H	0.63742800	-1.21677300	-0.80155500
C	2.37079100	-0.43436500	0.00885800
H	2.99281400	-0.25127400	0.87905300
C	2.87438900	0.09559200	-1.29857400
H	2.09688200	0.01013000	-2.06732600
O	3.00635700	1.55095700	-1.18509100
O	-7.25158300	0.03430700	-0.00701200
C	-9.04724900	-1.23539600	-1.43581900
F	-9.97623400	-0.92985500	-0.53398500
F	-8.92071300	-0.24197800	-2.31064900
F	-9.37468700	-2.35849100	-2.06511400
C	4.82847200	-1.53188300	-1.04815700
C	4.73703400	-0.03010400	-2.96072300
C	5.96520200	-0.94361700	-3.07829000
C	6.24252100	-1.34704000	-1.62065900
H	4.81303600	-1.35581500	0.02880900
H	4.06153100	-0.11432200	-3.82541700
H	5.02540500	1.02430600	-2.88045800
H	5.72028600	-1.83134400	-3.67160100
H	6.81224600	-0.44572200	-3.55485500
H	6.85686600	-2.24571100	-1.52448700
H	6.74322200	-0.52979100	-1.09164200
C	4.35283400	-3.04654500	-1.27526400
H	4.98165900	-3.45024100	-2.07590000

C	2.91459100	-3.22439500	-1.74580100
C	2.49157700	-2.58443600	-2.91713500
C	1.98486800	-4.02325700	-1.07210100
C	1.16040100	-2.65321800	-3.33045700
H	3.19479400	-2.01006900	-3.50454500
C	0.66647200	-4.12466600	-1.51659500
H	2.27031600	-4.56499800	-0.18048000
C	0.23010200	-3.42274100	-2.63660100
H	-0.80183900	-3.47008000	-2.95638000
C	4.64976900	-3.81618400	-0.00958900
C	5.56377700	-4.86819500	0.02845600
C	3.98681000	-3.46644000	1.17260900
C	5.79376300	-5.56198400	1.22087900
H	6.09706000	-5.15119200	-0.87288600
C	4.19789300	-4.17779700	2.34903100
H	3.27805400	-2.64716400	1.16285500
C	5.10668700	-5.23393500	2.38822100
H	5.27727700	-5.78456900	3.30408200
C	0.74530100	-1.84602100	-4.52457400
C	-0.27845500	-5.01359300	-0.76252700
C	3.36810000	-3.80732500	3.54296800
C	6.74861400	-6.72070400	1.21224900
F	1.40356300	-2.22149600	-5.64575400
F	-0.57524100	-1.93496000	-4.78549800
F	1.02677800	-0.52260100	-4.35411900
F	-0.15101600	-4.85975600	0.58140900
F	-0.05487700	-6.32444600	-1.01575600
F	-1.57189600	-4.76721400	-1.06687600
F	2.05899100	-4.11126900	3.34247300
F	3.41017200	-2.47563300	3.79079800
F	3.75583800	-4.44033000	4.66760100
F	7.84277000	-6.46486700	0.45835600
F	7.17964100	-7.04155200	2.45105800
F	6.17768400	-7.83573600	0.69392700
N	4.09401200	-0.48920600	-1.73035000
H	3.65853700	1.75115000	-0.49515700
C	-0.02045700	3.98019500	-1.15997600
C	0.35619700	2.38388800	-2.99824000
C	-0.47186800	3.57237700	-3.47750300
C	-0.05894100	4.69051100	-2.51326900
H	-1.04542200	3.86765500	-0.80430300
H	1.38673000	2.44018500	-3.35232400
H	-0.05553500	1.40621500	-3.23736700
H	-0.26526200	3.80698900	-4.52294900

H	-1.53949700	3.35298100	-3.37529900
H	0.92488400	5.08420300	-2.78139500
H	-0.76444400	5.52289600	-2.48639400
C	0.78001600	4.62466800	-0.00942300
H	0.15674500	5.46858400	0.30451900
C	2.14734600	5.19214300	-0.34439900
C	3.03623200	4.57322300	-1.22509600
C	2.57972300	6.33150700	0.34140400
C	4.33421000	5.05785500	-1.38655900
H	2.75293900	3.69257600	-1.78181600
C	3.87112100	6.82497000	0.15891600
H	1.90530300	6.83199900	1.02806500
C	4.76697100	6.18684800	-0.69806100
H	5.77344400	6.56444600	-0.82612300
C	0.83692400	3.64529500	1.16641800
C	-0.35500600	3.24606600	1.77849000
C	2.03475600	3.07412600	1.59833000
C	-0.34737600	2.27926100	2.78371000
H	-1.29752200	3.67829100	1.46079200
C	2.03124600	2.09618100	2.59356200
H	2.97328800	3.37112700	1.15189100
C	0.84405300	1.68686100	3.19631700
H	0.84543400	0.92205000	3.96242000
C	5.27972800	4.27432500	-2.25106600
C	4.32069800	8.01762400	0.95453100
C	3.33326100	1.49032600	3.03221900
C	-1.65209600	1.89242200	3.42169700
F	4.67396200	3.80432600	-3.36605400
F	6.34724900	4.99716000	-2.64182600
F	5.76029100	3.18551800	-1.59089700
F	3.32002400	8.90811900	1.13542500
F	5.34163500	8.67038400	0.35942100
F	4.74961800	7.66016900	2.18928600
F	4.27366400	1.55605600	2.05464400
F	3.19703400	0.18399300	3.36041600
F	3.85342800	2.11220200	4.11258200
F	-2.63207900	1.76398600	2.49718800
F	-1.56519400	0.71615500	4.08738500
F	-2.07793000	2.81619700	4.31281100
N	0.40237900	2.54482100	-1.49296800
H	-0.28094400	1.91762900	-1.05043700
H	1.34696700	2.26869800	-1.15976900

TS$_B$

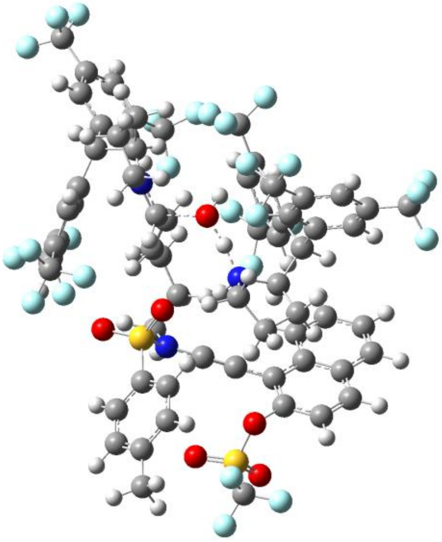

N	-1.79190600	-2.83268900	1.03529200
S	-1.55536900	-3.10064800	-0.65314600
O	-0.54439100	-4.16181100	-0.72154700
O	-1.28847600	-1.80745900	-1.30959000
C	-3.11323300	-3.69380100	-1.23644400
C	-3.96674300	-2.81646400	-1.90553900
C	-3.43015800	-5.04342500	-1.06246300
C	-5.16601000	-3.30976100	-2.40786100
H	-3.69497000	-1.77823300	-2.04224700
C	-4.63489500	-5.51102200	-1.57061000
H	-2.74536900	-5.71093000	-0.55215700
C	-5.52256400	-4.65399800	-2.24015300
H	-5.83650200	-2.63816600	-2.93269300
H	-4.89572400	-6.55684900	-1.44356700
C	-6.85191900	-5.16441400	-2.72130800
H	-6.76412300	-6.17272200	-3.13528600
H	-7.55852700	-5.21556500	-1.88494300
H	-7.28225400	-4.50781300	-3.48084500
C	-2.72951500	-1.93428200	1.36446100
C	-3.58035600	-1.13834900	1.71010300
C	-4.56992000	-0.18774700	2.06424600
C	-4.31027900	0.84888400	3.02428500
C	-5.81661800	-0.21225400	1.45916500
C	-3.09103000	0.90990700	3.74237200
C	-5.31460400	1.83949600	3.26660500
C	-6.80827600	0.75320700	1.68574800
C	-2.86798300	1.91546900	4.65852900
H	-2.34109900	0.14547300	3.56977100

C	-5.04594200	2.86903100	4.20657400
C	-6.55140800	1.77514700	2.57022800
H	-7.75483500	0.66807100	1.16514800
C	-3.84998100	2.90777600	4.88674500
H	-1.93015400	1.95112700	5.20203200
H	-5.80858100	3.62154000	4.38194200
H	-7.30122000	2.53617900	2.75840900
H	-3.65800900	3.69673400	5.60635000
S	-6.72915100	-2.63183000	1.14348400
O	-7.19248200	-2.41293100	2.50714300
O	-5.87266000	-3.75437700	0.80043800
C	-0.71653400	-3.14689100	2.02722700
H	-1.23532900	-3.63627500	2.85342900
H	-0.06287000	-3.88671200	1.56550100
C	0.05403100	-1.91837400	2.53904000
H	0.20996900	-2.04811200	3.61320800
H	-0.57462600	-1.02874200	2.43079200
C	1.43313500	-1.66324400	1.89710600
H	1.89503900	-0.79828600	2.38167600
H	2.07556600	-2.52565900	2.10781800
C	1.36653500	-1.45820600	0.41991800
H	0.95961900	-2.27692700	-0.16191000
C	1.79015200	-0.37571900	-0.23737300
H	2.26546500	0.44695500	0.28743600
C	1.66771900	-0.26584800	-1.69856900
H	0.86783400	-0.81964300	-2.17848900
O	0.60886600	1.35734300	-1.84046100
O	-6.08467400	-1.24301800	0.53044000
C	-8.22773600	-2.68192700	0.02272300
F	-9.06790600	-1.71918500	0.38799300
F	-7.85678700	-2.50334300	-1.23985400
F	-8.80458700	-3.87185400	0.16380400
C	4.08987700	0.23622800	-1.92705200
C	2.64475600	0.18771200	-3.90109800
C	4.08956900	0.46804300	-4.32036400
C	4.71111100	1.07371500	-3.05097300
H	4.00917900	0.80086800	-1.00170400
H	2.19246600	-0.67841300	-4.39366200
H	1.99749700	1.05555300	-4.06312900
H	4.59727200	-0.46525700	-4.58442200
H	4.14242600	1.13646700	-5.18117300
H	5.80223800	1.03415000	-3.03657500
H	4.40273500	2.11632100	-2.93529100
C	4.95663600	-1.06964800	-1.64833900

H	5.55260900	-1.23282500	-2.55146700
C	4.14592400	-2.34601700	-1.44838600
C	3.35385600	-2.80002700	-2.50836800
C	4.13203000	-3.08243700	-0.25971900
C	2.49457600	-3.88592800	-2.35072900
H	3.39141500	-2.29572600	-3.46523600
C	3.30427000	-4.19693900	-0.12458800
H	4.74676000	-2.78558400	0.57872000
C	2.45886100	-4.59809800	-1.15716200
H	1.78176500	-5.43171800	-1.03000500
C	5.91163000	-0.76389600	-0.51461900
C	7.29269600	-0.81653000	-0.69565900
C	5.41472200	-0.40172500	0.74355100
C	8.15703500	-0.51531100	0.36173700
H	7.69802800	-1.09044100	-1.66376200
C	6.27851300	-0.11271200	1.79437800
H	4.34516400	-0.36136400	0.90508000
C	7.66101700	-0.16365200	1.61378900
H	8.33360600	0.06565200	2.43069200
C	1.53441900	-4.18760900	-3.46229200
C	3.29622600	-4.98610000	1.15143900
C	5.70967600	0.31310200	3.11665200
C	9.63904100	-0.63169300	0.14635000
F	2.15265600	-4.24432500	-4.66552100
F	0.87545900	-5.34788900	-3.28929300
F	0.59510400	-3.20623900	-3.56862800
F	3.84991200	-4.30698600	2.18235800
F	3.97328500	-6.15139300	1.03870800
F	2.03287600	-5.31292900	1.52779700
F	4.47386300	-0.20091800	3.32360500
F	5.58189900	1.66413900	3.19935900
F	6.48425300	-0.06489600	4.15333500
F	10.00638200	-0.17473800	-1.07269600
F	10.34691200	0.05854500	1.06516400
F	10.05259200	-1.92018700	0.21600100
N	2.74642100	-0.04322400	-2.44634400
H	1.14575000	1.98770500	-1.34062700
C	-2.31261400	1.99743300	0.15145700
C	-2.59515800	1.00187800	-2.03710500
C	-3.97977400	1.14190200	-1.41250700
C	-3.75062600	2.23760500	-0.36317400
H	-2.34630900	1.43302300	1.08285800
H	-2.37838100	1.83130200	-2.71534300
H	-2.41605400	0.06549400	-2.56421600

H	-4.74627700	1.40778800	-2.14390600
H	-4.27051500	0.20015300	-0.93593600
H	-3.82047400	3.22018400	-0.83760700
H	-4.47095800	2.21111000	0.45482800
C	-1.54356500	3.31379000	0.46462900
H	-2.14006400	3.75635600	1.27046900
C	-1.54619700	4.32625200	-0.66565400
C	-0.99865200	4.05191800	-1.92240700
C	-2.14926800	5.57168600	-0.47001300
C	-1.06714600	4.98763100	-2.95152300
H	-0.50276100	3.11128100	-2.12031200
C	-2.20598000	6.50947300	-1.50380000
H	-2.58565600	5.81071600	0.49457700
C	-1.67131000	6.22695100	-2.75858300
H	-1.72483600	6.95106400	-3.56071100
C	-0.17189000	3.03364800	1.07491500
C	-0.12706800	2.35221300	2.29684300
C	1.03703300	3.41947000	0.49335400
C	1.08625100	2.05681600	2.91201200
H	-1.05287200	2.06999200	2.78109500
C	2.25399600	3.07781100	1.09247100
H	1.04676900	3.98135800	-0.43309900
C	2.29493500	2.40509900	2.30987600
H	3.23809600	2.15979800	2.77854400
C	-0.51931500	4.58083400	-4.28824400
C	-2.84712200	7.83889800	-1.22711600
C	3.53771100	3.43525900	0.39784200
C	1.08140900	1.40858900	4.26822300
F	0.72266600	4.04760500	-4.17972900
F	-1.29236300	3.62432000	-4.86323600
F	-0.44387300	5.60784500	-5.15689700
F	-4.08660300	7.69304400	-0.70129100
F	-2.96813200	8.59636300	-2.33630500
F	-2.13294700	8.55776700	-0.32814100
F	3.40710200	3.34815100	-0.95354100
F	4.54439800	2.60017000	0.74551400
F	3.94976300	4.69024400	0.66667100
F	0.08511400	0.49618000	4.39324500
F	2.23919000	0.77133900	4.53510200
F	0.88727400	2.31644500	5.25450000
N	-1.66728400	1.08827000	-0.86739600
H	-1.63040900	0.14880300	-0.46722600
H	-0.47059900	1.27351100	-1.28148900

B

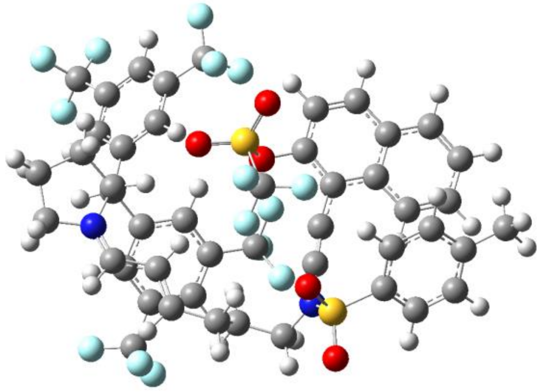

N	3.46133700	-2.08166200	-0.39151000
S	4.68277000	-2.35211600	0.79474800
O	5.27075900	-3.65453900	0.44994800
O	4.13478200	-2.15777200	2.14667100
C	5.79699300	-1.01948600	0.43724100
C	5.81317100	0.10021900	1.27089400
C	6.59096300	-1.09451800	-0.70784400
C	6.64169100	1.16516100	0.93855500
H	5.18341000	0.13671800	2.15003700
C	7.41648900	-0.01764800	-1.01750000
H	6.56355000	-1.97154500	-1.34476200
C	7.45026600	1.12583700	-0.20700500
H	6.65445000	2.04670500	1.57181000
H	8.03967800	-0.06212600	-1.90489500
C	8.32101500	2.30061500	-0.55684700
H	8.84786200	2.14171500	-1.50028400
H	7.71981600	3.21113100	-0.64646600
H	9.06613200	2.47922200	0.22604800
C	2.97792000	-0.83239500	-0.44178000
C	2.64717700	0.33567100	-0.41598900
C	2.42907100	1.73540400	-0.47408300
C	3.36032900	2.58575600	-1.16730600
C	1.34154100	2.33446700	0.14271800
C	4.50716100	2.06337300	-1.81402900
C	3.13189500	3.99868100	-1.18971800
C	1.09122800	3.71481800	0.10671300
C	5.39232000	2.90365100	-2.45137300
H	4.68160400	0.99459900	-1.79257300
C	4.06484800	4.83648000	-1.85590400

C	1.98298200	4.53113800	-0.55003500
H	0.21240400	4.11450700	0.59127100
C	5.17294500	4.30161800	-2.47206400
H	6.26927200	2.49015500	-2.93824500
H	3.88504300	5.90706800	-1.86644200
H	1.81113900	5.60197600	-0.58378400
H	5.88183800	4.94957100	-2.97719000
S	0.31231000	1.48046400	2.41834000
O	-0.96493700	0.85150900	2.72471300
O	0.70722700	2.76030300	2.98386400
C	2.75668300	-3.21289000	-1.04033500
H	2.71549400	-2.98379900	-2.10767200
H	3.38433300	-4.09485500	-0.90870900
C	1.35221700	-3.42881600	-0.49746300
H	0.85379900	-4.16820800	-1.13092900
H	0.78727100	-2.49865500	-0.59208300
C	1.32160200	-3.90088100	0.96780000
H	1.87740800	-4.83637000	1.06852900
H	1.82090800	-3.15562400	1.59581100
C	-0.08030400	-4.08555000	1.44950500
H	-0.39861800	-5.09702800	1.69552400
C	-0.99044600	-3.08908700	1.56532100
H	-0.74730400	-2.06375300	1.31176100
C	-2.29795900	-3.41932100	2.02789900
H	-2.48235100	-4.45209800	2.31624800
C	-4.60809900	-3.07902800	2.75152200
C	-3.36740400	-1.16829800	1.86680400
C	-5.39147700	-1.78653400	3.02489900
H	-4.38787300	-3.64106800	3.66015400
H	-5.09707800	-3.74220100	2.03531400
C	-4.33080300	-0.67648500	2.95335000
H	-2.37391200	-0.73616100	1.95612100
H	-6.15689900	-1.63388700	2.26056500
H	-5.88907900	-1.82239200	3.99461900
H	-4.75073400	0.30193200	2.72352600
H	-3.78259400	-0.59977700	3.89638800
N	-3.30936300	-2.61583300	2.17699900
C	-3.92199200	-0.96644900	0.41812600
H	-4.84024500	-1.55468900	0.34207600
C	-2.96827700	-1.51572100	-0.63294900
C	-1.97871500	-0.73096700	-1.23648500
C	-3.06225900	-2.86385800	-0.98615600
C	-1.12131200	-1.28731700	-2.18239600
H	-1.87822400	0.31785800	-0.99128500

C	-2.17309700	-3.42077800	-1.90511600
H	-3.82696700	-3.48722300	-0.53685400
C	-1.20579000	-2.63663200	-2.52363800
H	-0.52199200	-3.06764700	-3.24443800
C	-4.29753700	0.48156300	0.17243900
C	-3.44384900	1.53373300	0.51980300
C	-5.51802100	0.77711800	-0.43650100
C	-3.80463300	2.85096800	0.24328300
H	-2.50060800	1.33408900	1.01103400
C	-5.87545000	2.10009700	-0.70016000
H	-6.19135900	-0.02845300	-0.70890900
C	-5.02208100	3.14847800	-0.36685400
H	-5.29977300	4.17408500	-0.57480400
O	0.39969400	1.48820900	0.78653600
C	1.63342400	0.23038900	2.87207200
F	1.55807200	0.02387700	4.18146600
F	1.39223600	-0.90154400	2.21999600
F	2.82702000	0.71458100	2.55393900
C	-0.09885000	-0.43293400	-2.87540800
C	-2.17913700	-4.90360100	-2.12775600
C	-2.83634100	3.95923200	0.53885600
C	-7.22433400	2.39073000	-1.29403700
F	1.09529300	-1.06618400	-2.97315500
F	-0.47438400	-0.12754300	-4.13885900
F	0.11372500	0.73535200	-2.23346800
F	-1.71306200	-5.24684600	-3.34497300
F	-3.41075200	-5.43923300	-1.99367000
F	-1.38456300	-5.52717700	-1.21392500
F	-2.03994000	3.67262000	1.59962500
F	-3.45597800	5.12555500	0.80777500
F	-2.00817100	4.18630300	-0.51105800
F	-7.25848900	3.58487100	-1.92182000
F	-7.59110900	1.44926800	-2.19239600
F	-8.19097400	2.41692600	-0.34443100

TS$_C$

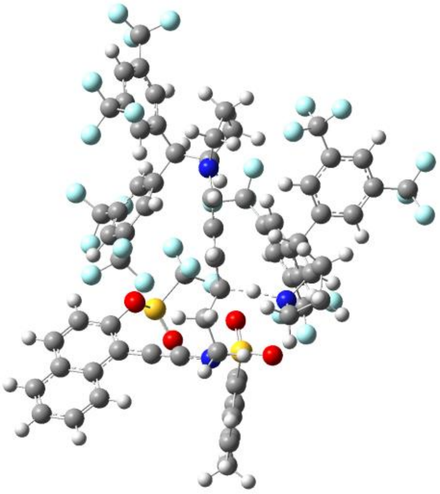

N	4.19892800	1.15366900	2.05607200
S	4.00684700	2.24513900	0.74149500
O	3.71182500	3.54459400	1.37998300
O	3.05525100	1.71027900	-0.23976800
C	5.62174800	2.25240300	0.01765100
C	5.72155300	2.18456600	-1.37064700
C	6.74809700	2.39512700	0.83306500
C	6.98660100	2.25223100	-1.94884900
H	4.83711700	2.06499000	-1.98066400
C	7.99852500	2.45514300	0.23314800
H	6.64957200	2.44249700	1.91137600
C	8.13744900	2.38610800	-1.16345600
H	7.07957300	2.19163800	-3.02831300
H	8.88354900	2.55278300	0.85419900
C	9.50153900	2.45921700	-1.79250100
H	9.95175000	3.44461000	-1.62731200
H	10.17652300	1.71950300	-1.35003600
H	9.45428000	2.28339500	-2.86935700
C	4.46355600	-0.12018400	1.71796300
C	4.71151100	-1.27475000	1.44183900
C	4.95344400	-2.62847900	1.09790300
C	5.87144400	-3.44159800	1.84706300
C	4.29870200	-3.21536300	0.02545900
C	6.58252200	-2.92905300	2.96002200
C	6.07053200	-4.80594600	1.46101200
C	4.49613800	-4.54693900	-0.37360000
C	7.45114200	-3.73243200	3.66444300
H	6.43213300	-1.89322500	3.24427400
C	6.97204100	-5.60644300	2.21100400

C	5.37138000	-5.33026300	0.34252100
H	3.94777300	-4.93180200	-1.22464800
C	7.64704700	-5.08293600	3.28952800
H	7.99237500	-3.32805800	4.51372300
H	7.11668400	-6.64061800	1.91391200
H	5.53437300	-6.36467400	0.05848100
H	8.33430300	-5.70323400	3.85559200
S	3.78787500	-1.68683200	-2.02009600
O	3.95408800	-2.63103700	-3.11924100
O	4.83190900	-0.71329000	-1.72934700
C	3.52366100	1.42447600	3.35599700
H	4.22725400	1.11113300	4.13106600
H	3.41769300	2.50453900	3.42646000
C	2.20005900	0.68175500	3.52208400
H	1.80970200	0.93072300	4.51545500
H	2.41485600	-0.39314900	3.53170500
C	1.14976900	0.96666000	2.44927400
H	0.95006200	2.32010100	2.46100000
H	1.50014000	0.79343000	1.42930000
C	-0.16679100	0.52074900	2.67905500
H	-0.52024300	0.50608500	3.71192100
C	-1.08509300	0.17381900	1.68946500
H	-0.74033600	0.07811700	0.66586200
C	-2.40696400	-0.09110600	2.03509800
H	-2.69713800	0.04597600	3.07470400
C	-4.79872200	-0.51266300	1.73113500
C	-3.31468100	-0.63048600	-0.22225300
C	-5.64661400	-0.36649900	0.45739000
H	-4.92557900	0.33283200	2.40842100
H	-5.01673800	-1.43151000	2.27948400
C	-4.64131800	0.02639600	-0.64213900
H	-2.45578900	-0.05878500	-0.55802800
H	-6.13890400	-1.30880600	0.21737100
H	-6.41786900	0.39203700	0.58497700
H	-4.95741500	-0.28427700	-1.63965400
H	-4.50723600	1.11179200	-0.65538400
N	-3.39023400	-0.52219300	1.25094000
C	-3.14241900	-2.06276200	-0.81980300
H	-3.27191300	-1.88360900	-1.89061500
C	-4.23300400	-3.03547100	-0.40415700
C	-4.27244800	-3.58310500	0.88005400
C	-5.24569200	-3.37140100	-1.30526800
C	-5.33682300	-4.39344700	1.27013500
H	-3.48501300	-3.36777000	1.59180100

C	-6.29499200	-4.20496800	-0.91543200
H	-5.22028000	-2.97137100	-2.31310000
C	-6.36104200	-4.71037100	0.38084800
H	-7.18430700	-5.34172100	0.68796000
C	-1.76570800	-2.69357200	-0.67099700
C	-1.06951100	-2.75832700	0.53894200
C	-1.18818800	-3.28413800	-1.79932400
C	0.17852900	-3.37598500	0.60653100
H	-1.48028000	-2.32623600	1.43795100
C	0.03829600	-3.94232600	-1.71299100
H	-1.70215500	-3.23152800	-2.75265500
C	0.73262300	-3.99693500	-0.50883700
H	1.68575700	-4.50049700	-0.44240500
O	3.32319300	-2.45515900	-0.65692200
C	2.14942500	-0.78643400	-2.27967100
F	2.45321900	0.36432800	-2.87396700
F	1.39027500	-1.52175500	-3.08237700
F	1.52382200	-0.56667100	-1.13216400
C	-0.19932700	4.47515100	2.06868700
C	1.17246200	4.01524000	4.02535300
C	0.19842100	5.15282400	4.33200700
C	-0.93992200	4.88653600	3.34711500
H	0.15897100	5.37851900	1.56340800
H	0.90886500	3.11626800	4.59242100
H	2.21478000	4.26089900	4.23356300
H	-0.12163500	5.15104500	5.37614700
H	0.66364700	6.12037700	4.11725400
H	-1.56552600	4.05634700	3.69668600
H	-1.58607900	5.74898000	3.17825500
N	0.99299700	3.71329200	2.56944000
H	1.83439500	3.94777400	2.04288000
C	-1.05728900	3.64431600	1.10720000
H	-1.26424000	2.69644400	1.61006600
C	-2.40007000	4.30842600	0.85280300
C	-3.58097400	3.65938400	1.22013800
C	-2.47727100	5.55068400	0.22060800
C	-4.81822500	4.24554300	0.95222400
H	-3.53010800	2.69575400	1.71497300
C	-3.71836000	6.13777900	-0.02663100
H	-1.56838300	6.05908700	-0.08582500
C	-4.89876200	5.49098400	0.33096700
H	-5.86026600	5.94506200	0.12821100
C	-0.41675600	3.30683300	-0.23120300
C	-1.08810200	2.38376600	-1.03941400

C	0.76144100	3.88008800	-0.70888100
C	-0.58415500	2.02291700	-2.28454300
H	-2.02013500	1.95809300	-0.68823500
C	1.25236400	3.52645900	-1.96839100
H	1.31863900	4.59073200	-0.11248200
C	0.58854800	2.60095600	-2.76632300
H	0.98248200	2.32315900	-3.73435900
C	-3.75182500	7.47566300	-0.70926100
C	-6.08123000	3.49665700	1.25902400
C	2.50594600	4.16509300	-2.49277900
C	-1.27092600	0.94326500	-3.06666200
F	-5.00346100	7.95835600	-0.84364500
F	-3.21025500	7.41196900	-1.94865800
F	-3.03506200	8.39868500	-0.02525000
F	-7.09775600	4.31577300	1.59428900
F	-5.92022900	2.61419000	2.27367800
F	-6.49952700	2.77678900	0.18398200
F	3.20936200	3.31882900	-3.28306900
F	2.23816100	5.26001800	-3.24446300
F	3.33306900	4.57049000	-1.50278300
F	-2.62077900	1.04369100	-3.00623300
F	-0.92180900	0.93496700	-4.36562100
F	-0.96595800	-0.29050400	-2.56986400
C	-7.39071400	-4.49371000	-1.90062300
C	-5.38606800	-4.85352400	2.69726000
F	-6.89763600	-4.80170000	-3.12210300
F	-8.19762300	-3.41778800	-2.07243300
F	-8.17663900	-5.51904400	-1.51231000
F	-6.34594600	-5.77265700	2.91761400
F	-4.20984300	-5.38900400	3.09650600
F	-5.63296900	-3.81117000	3.53546400
C	0.59731500	-4.61682500	-2.93219900
C	0.98278900	-3.26643900	1.86771500
F	0.02891700	-5.82911100	-3.14052900
F	1.93079800	-4.82241400	-2.83819300
F	0.38070000	-3.89152900	-4.05205700
F	0.20486800	-3.21608400	2.97291100
F	1.84664300	-4.29197200	2.02299400
F	1.72710100	-2.12859900	1.86935400

C

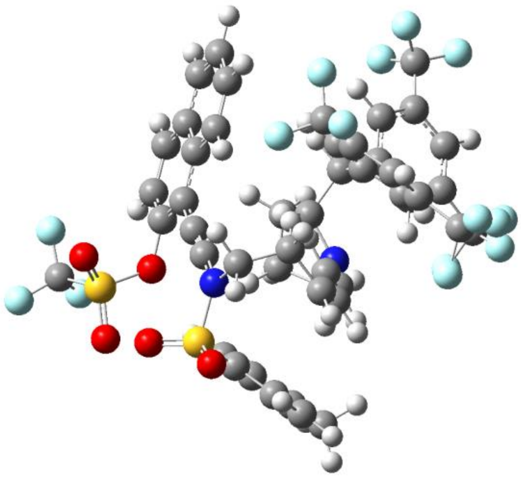

N	-3.40541500	-2.29865700	0.82567700
S	-4.45471600	-2.56573000	-0.51722600
O	-5.52395600	-1.56731700	-0.40023400
O	-4.76861400	-3.99863700	-0.48321800
C	-3.51358000	-2.19448900	-1.97372900
C	-3.02090300	-3.24396000	-2.75011400
C	-3.30624000	-0.86072000	-2.32927700
C	-2.30540400	-2.94162800	-3.90290500
H	-3.19759900	-4.27104100	-2.45742700
C	-2.58183800	-0.58229200	-3.48400400
H	-3.71516000	-0.05837800	-1.73450600
C	-2.06837700	-1.61214600	-4.28313300
H	-1.92283800	-3.75009500	-4.51825400
H	-2.42010800	0.45172300	-3.77039200
C	-1.25826200	-1.30723400	-5.51334800
H	-0.19317600	-1.49239000	-5.32846300
H	-1.55416400	-1.94616800	-6.35074500
H	-1.36836900	-0.26338200	-5.81635800
C	-3.01583500	-1.03977500	1.02993000
C	-2.68982600	0.12032400	1.19308400
C	-2.27927400	1.45919800	1.38841500
C	-1.38691200	1.80781600	2.46338100
C	-2.70133800	2.48205500	0.55157500
C	-0.94878900	0.84202300	3.40053500
C	-0.93417800	3.15933100	2.59128100
C	-2.26526000	3.81216200	0.67080900
C	-0.08878100	1.19100600	4.41943500
H	-1.29709900	-0.17898000	3.30565600

C	-0.03514300	3.48136800	3.64171300
C	-1.38686800	4.14509400	1.67409800
H	-2.61726800	4.54687700	-0.04293600
C	0.38039700	2.51996600	4.53554900
H	0.24317900	0.43603000	5.12419400
H	0.31295300	4.50612000	3.72826800
H	-1.03037600	5.16457600	1.77476000
H	1.06476900	2.78027300	5.33636200
S	-5.17246600	2.11783600	-0.29663900
O	-5.49422700	1.73213000	1.06859200
O	-5.70599000	1.43599500	-1.46364600
C	-2.93746400	-3.40610000	1.70027600
H	-3.16067500	-3.10692200	2.72839400
H	-3.55028200	-4.27305400	1.45741600
C	-1.44594100	-3.70971400	1.53096500
H	-1.19384000	-4.46218600	2.28949800
H	-0.88200600	-2.81326200	1.79145500
C	-1.09469000	-4.19500500	0.15606000
H	-1.38406100	-5.21520000	-0.08679200
C	-0.51450600	-3.44066100	-0.80056300
H	-0.34690500	-3.89266200	-1.77618800
C	-0.09000500	-2.06827000	-0.65809700
H	-0.13876800	-1.62651900	0.32712600
C	0.39447100	-1.34315500	-1.69306800
H	0.41757100	-1.78444400	-2.68617400
C	0.73944200	0.81092600	-2.82828900
C	0.89145000	0.73710100	-0.41350900
C	-0.01585400	2.01276500	-2.26004000
H	0.20054500	0.28264600	-3.61477100
H	1.71217100	1.12257200	-3.23006300
C	0.65280800	2.18288000	-0.89308400
H	0.04030200	0.41508200	0.19547500
H	0.06225500	2.90185800	-2.89103300
H	-1.07548100	1.76166400	-2.13851900
H	1.60801800	2.70360600	-1.01240800
H	0.05351700	2.74274700	-0.17608900
N	0.92415300	-0.06777800	-1.65289700
C	2.15591000	0.61747000	0.49433600
H	1.93338200	1.27475000	1.34197200
C	3.43036100	1.14038500	-0.14008200
C	3.85444300	0.74142600	-1.41059800
C	4.21663000	2.06120500	0.56006700
C	5.01893900	1.26708600	-1.97006500
H	3.25865900	0.03745700	-1.97550600

C	5.38995800	2.56878700	0.00211000
H	3.90819000	2.38389400	1.54889700
C	5.80140100	2.18216700	-1.27294100
H	6.70671400	2.58409700	-1.70886800
C	2.30258400	-0.78463800	1.06472800
C	2.87407500	-1.83843300	0.34688300
C	1.74553600	-1.05864000	2.31357700
C	2.83178100	-3.13768700	0.84884400
H	3.32959700	-1.66353200	-0.61906900
C	1.70150200	-2.36302000	2.80943300
H	1.31553300	-0.25041900	2.89071600
C	2.23231500	-3.41930900	2.07538700
H	2.18481500	-4.43394900	2.44737100
O	-3.54968200	2.16890100	-0.53794000
C	-5.62310200	3.92958600	-0.48104200
F	-5.02525400	4.42650600	-1.55949800
F	-5.23585900	4.59616100	0.60225900
F	-6.94254000	4.00633700	-0.61478500
C	6.17626700	3.59783000	0.76125300
C	5.38534900	0.82622300	-3.35632400
F	5.66925700	4.84387100	0.58854600
F	6.16578900	3.36031100	2.09272800
F	7.46827600	3.64573000	0.37058800
F	6.52319200	1.39461800	-3.80341600
F	5.54992200	-0.51601000	-3.42804000
F	4.40687200	1.13598300	-4.24602600
C	1.04050000	-2.60259000	4.13482500
C	3.35030300	-4.25115200	-0.01184000
F	3.53766600	-5.39609900	0.67870700
F	4.53174800	-3.93152700	-0.59136600
F	2.49484400	-4.53761900	-1.02517700
F	1.61671200	-1.87729800	5.12496800
F	1.07634700	-3.89655000	4.50903900
F	-0.27128100	-2.23485600	4.12324900

TS$_{R\text{-D}}$

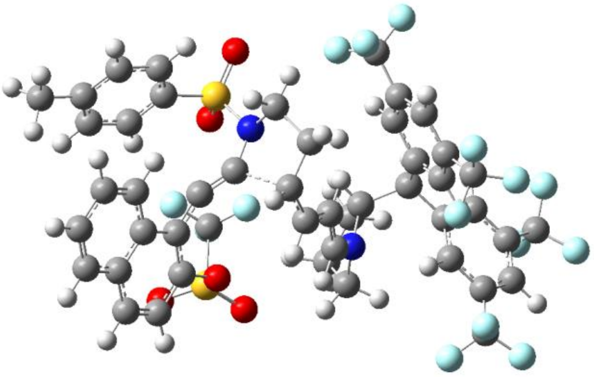

N	-2.45497800	1.67184400	-0.39687000
S	-3.19351600	1.82296900	-1.92251600
O	-2.73245500	0.69579900	-2.74111800
O	-2.89521800	3.19649200	-2.36102000
C	-4.94581000	1.67005700	-1.67332700
C	-5.64642000	2.74784700	-1.13184100
C	-5.57767900	0.45513100	-1.94847200
C	-7.00764900	2.59996100	-0.86879200
H	-5.14481500	3.68727500	-0.92992400
C	-6.93689900	0.33283900	-1.68948600
H	-5.01129200	-0.37174000	-2.35531000
C	-7.66998000	1.39637200	-1.14121900
H	-7.56289400	3.43278100	-0.44886900
H	-7.43620200	-0.60851700	-1.89713500
C	-9.13292100	1.22488000	-0.83687100
H	-9.69592300	0.97937100	-1.74387300
H	-9.28249400	0.40130100	-0.13057800
H	-9.56114800	2.13149100	-0.40304500
C	-2.83511800	0.58025800	0.40343900
C	-3.64496700	-0.38769400	0.39466200
C	-4.22015300	-1.49433200	1.02849700
C	-5.54344000	-1.37979800	1.62233300
C	-3.61637200	-2.75269400	1.12032500
C	-6.23995800	-0.14905000	1.60710100
C	-6.15845600	-2.51543700	2.23650900
C	-4.20957600	-3.86821400	1.72977300
C	-7.49260900	-0.03480400	2.17429400
H	-5.77089200	0.70580700	1.13492800
C	-7.44967700	-2.36765100	2.80797600
C	-5.46900800	-3.75930200	2.27296000

H	-3.65884900	-4.80078000	1.77446000
C	-8.10417700	-1.15510700	2.77969200
H	-8.01160300	0.91794900	2.14869200
H	-7.91157700	-3.23375800	3.27305100
H	-5.94014800	-4.61468200	2.74525300
H	-9.09096900	-1.05860400	3.22188500
S	-1.91456100	-3.76919700	-0.63816000
O	-2.99299500	-4.68939600	-0.97483400
O	-0.54610000	-4.22790900	-0.43962200
C	-2.32743500	2.89475500	0.43015400
H	-1.94670300	3.71228200	-0.17253400
H	-3.30524100	3.17056100	0.84501200
C	-1.35838000	2.47390600	1.53678300
H	-1.46198000	3.14279900	2.39614200
H	-0.33709100	2.56495000	1.16890000
C	-1.65471500	1.03803300	1.93241700
H	-2.36270500	0.90955800	2.74668200
C	-0.70789100	0.01094000	1.78549400
H	-0.92480500	-0.91990700	2.30743000
C	0.37500300	-0.01807600	0.89542900
H	0.61721500	0.85916100	0.31207700
C	1.03982200	-1.21105200	0.67497400
H	0.77700900	-2.06700400	1.29086500
C	2.39139100	-2.85692600	-0.54457300
C	2.30220700	-0.57810700	-1.37898200
C	2.22743500	-2.93764600	-2.06058100
H	1.76549600	-3.55626200	0.00748200
H	3.43416400	-3.00706100	-0.24600700
C	2.67878300	-1.54686500	-2.52792000
H	1.39679000	-0.01670100	-1.62779000
H	2.81958700	-3.74300300	-2.50050900
H	1.17657600	-3.10625800	-2.30689300
H	3.76135700	-1.53760000	-2.68161400
H	2.20751900	-1.23731000	-3.46265200
N	1.97772900	-1.47193000	-0.24828000
C	3.44869800	0.45626200	-1.12222200
H	3.64666200	0.85432700	-2.12292100
C	4.73446100	-0.18987200	-0.64954900
C	4.80633800	-0.92027700	0.54039900
C	5.89025400	-0.07798000	-1.42888100
C	5.99703900	-1.53765900	0.92371700
H	3.93140500	-1.02281600	1.16938100
C	7.08058900	-0.68801600	-1.03330800
H	5.85989900	0.49081400	-2.35183200

C	7.14459000	-1.43122900	0.14433900
H	8.06634500	-1.91052800	0.44812600
C	3.00153900	1.65150400	-0.29220900
C	3.37208300	1.86326900	1.03468200
C	2.14976000	2.58327300	-0.90207400
C	2.88690900	2.96875600	1.73893800
H	4.03958400	1.17748000	1.53999500
C	1.67188800	3.68152700	-0.19412100
H	1.84892900	2.43837100	-1.93418000
C	2.03487200	3.88644000	1.13822800
H	1.65439500	4.73488800	1.69151200
O	-2.27077400	-2.87391100	0.67358500
C	-1.87438400	-2.46073400	-1.97437200
F	-1.10949000	-1.44979200	-1.58550100
F	-3.11752600	-2.06120800	-2.20689100
F	-1.36482100	-3.01303100	-3.07661500
C	3.30200300	3.13051800	3.17251200
C	0.77736800	4.67283700	-0.88001400
C	8.28757200	-0.59778300	-1.92236500
C	5.98679400	-2.36687100	2.17381200
F	9.44034500	-0.71411800	-1.22778300
F	8.33624500	0.57503800	-2.59247500
F	8.29485700	-1.57917000	-2.85780800
F	7.22106600	-2.75284500	2.55407500
F	5.43232000	-1.69918800	3.21249900
F	5.24894000	-3.49453700	2.00748700
F	2.75148500	4.21638900	3.75315200
F	4.64596300	3.24921500	3.29228500
F	2.94663500	2.05442600	3.91501300
F	1.47971800	5.64876800	-1.50206400
F	-0.05357700	5.28947900	-0.00509800
F	0.00365800	4.08789300	-1.82081100

TS$_{\mathit{S}\text{-D}}$

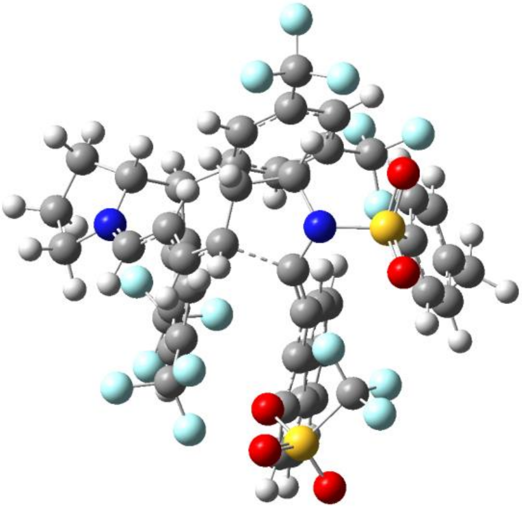

N	3.17807600	1.75212600	1.40026600
S	3.93570400	2.50945700	0.08119200
O	5.16537800	1.76156000	-0.20438700
O	4.03643700	3.92767100	0.46960400
C	2.86883900	2.39288500	-1.33603400
C	1.78945300	3.26949000	-1.44536500
C	3.12997700	1.43277500	-2.31682600
C	0.96727200	3.18115700	-2.56714000
H	1.60191800	4.01534200	-0.68250900
C	2.29559300	1.36120800	-3.42484000
H	3.97089100	0.76138600	-2.20849800
C	1.20329000	2.23007900	-3.56721100
H	0.13893400	3.86942500	-2.67459300
H	2.48652800	0.61279300	-4.18770300
C	0.29392800	2.11532800	-4.76002800
H	0.86754700	2.12195000	-5.69240700
H	-0.25732700	1.16889500	-4.72567700
H	-0.43303500	2.92870400	-4.79123400
C	2.67132000	0.44100300	1.21192900
C	2.60175000	-0.38175600	0.25542400
C	2.16143200	-1.52267900	-0.40987500
C	1.00532500	-1.45502800	-1.29578900
C	2.79605900	-2.76971100	-0.32896000
C	0.22904800	-0.27909700	-1.39692900
C	0.62814600	-2.59792000	-2.06827600
C	2.41377200	-3.89816300	-1.06471500
C	-0.87613200	-0.22592500	-2.22191100
H	0.51854000	0.58922900	-0.81659500

C	-0.51230900	-2.51183400	-2.90886700
C	1.36004000	-3.81024800	-1.94596700
H	2.94867700	-4.82937900	-0.92212800
C	-1.25089200	-1.35129100	-2.98608700
H	-1.44953700	0.68760600	-2.29493800
H	-0.79874000	-3.38939100	-3.48096600
H	1.05380000	-4.67562300	-2.52333000
H	-2.12639200	-1.30209400	-3.62624300
S	5.35505200	-3.20418500	0.25348800
O	5.45952400	-3.90203700	-1.02201300
O	5.98005000	-3.71745300	1.46435900
C	2.20139800	2.54705500	2.17421800
H	2.60554200	3.53040200	2.39688600
H	1.27694700	2.65144900	1.59691300
C	1.96887900	1.69253300	3.41765900
H	1.00927900	1.94971600	3.87082600
H	2.75087100	1.90360400	4.15196600
C	2.04758400	0.21746300	3.02122300
H	2.96159600	-0.27811600	3.34216500
C	0.90878700	-0.60815300	3.12752000
H	1.08124800	-1.68383800	3.14489600
C	-0.42472800	-0.19185700	3.14732100
H	-0.66159400	0.85647800	3.02445900
C	-1.43970100	-1.11758500	3.34055000
H	-1.16114500	-2.15526900	3.50973300
C	-3.70215400	-2.01087900	3.61940000
C	-3.45167400	0.35260200	3.01914300
C	-5.08732100	-1.36308400	3.51959100
H	-3.50192600	-2.39191200	4.62528100
H	-3.55171400	-2.82541300	2.90566100
C	-4.80062600	0.13191200	3.72646200
H	-2.90999500	1.17384300	3.48505200
H	-5.52108800	-1.53953500	2.53312600
H	-5.77438700	-1.76463800	4.26646400
H	-5.58485800	0.78343900	3.33709400
H	-4.67844200	0.34628800	4.79254800
N	-2.75684600	-0.90691000	3.33290900
C	-3.64199900	0.64966800	1.47654600
H	-4.66653400	1.02926600	1.41099700
C	-3.62584500	-0.60907300	0.62452600
C	-2.49993400	-1.44019700	0.57199500
C	-4.78969500	-1.02502000	-0.02214700
C	-2.60062000	-2.72250900	0.04233700
H	-1.56116600	-1.11603900	0.99516200

C	-4.84108600	-2.26397000	-0.66961900
H	-5.67217700	-0.39439800	0.00028200
C	-3.76458600	-3.14160800	-0.60375000
H	-3.83452500	-4.13568500	-1.02765300
C	-2.81351100	1.79108700	0.87907300
C	-3.00198300	2.07093700	-0.48034800
C	-2.02219500	2.67152700	1.62152800
C	-2.43539500	3.19990000	-1.07060100
H	-3.62592200	1.41662700	-1.07942000
C	-1.43075000	3.78286500	1.01781700
H	-1.87199200	2.53291800	2.68236900
C	-1.63860300	4.06802500	-0.32949600
H	-1.20232200	4.94830700	-0.78410700
O	3.80748800	-2.91304400	0.66215900
C	6.00075000	-1.47279600	-0.01928100
F	5.74643700	-0.74057200	1.05415900
F	5.41133800	-0.96863300	-1.09846900
F	7.31558700	-1.54597000	-0.21847600
C	-2.78811500	3.50910600	-2.49813100
C	-0.63309200	4.75102600	1.84477700
C	-6.08197500	-2.64195000	-1.42273600
C	-1.54637700	-3.73866000	0.36962400
F	-6.17387400	-3.97302300	-1.62851600
F	-6.12581300	-2.05137800	-2.64229500
F	-7.20369300	-2.25675000	-0.76976500
F	-1.31651100	-4.60608600	-0.63781000
F	-0.37042600	-3.18799900	0.73137800
F	-1.95270000	-4.49112900	1.43402500
F	-2.06439300	4.53152100	-3.00719600
F	-4.09143800	3.85186700	-2.61897300
F	-2.59918500	2.44295900	-3.31466800
F	-1.34059400	5.86708800	2.13389700
F	0.48643600	5.16344200	1.20080900
F	-0.23975700	4.21992900	3.02577400

***R*-D**

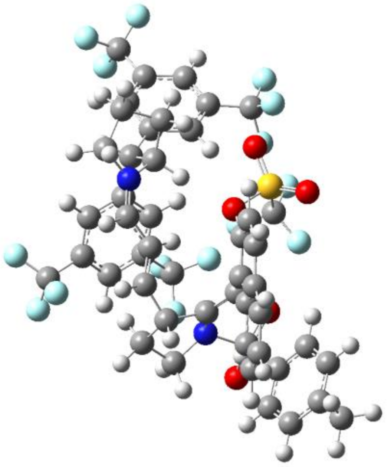

N	-2.57685500	1.59743800	-1.37580700
S	-3.12554900	0.83549500	-2.76863100
O	-2.18975700	-0.23955700	-3.12520800
O	-3.35502500	1.91934300	-3.74543800
C	-4.69970100	0.10265500	-2.37318700
C	-5.82728500	0.92154800	-2.30289300
C	-4.76537600	-1.25026100	-2.03255300
C	-7.03645600	0.37214600	-1.87743900
H	-5.76626700	1.96571400	-2.58713800
C	-5.98286500	-1.78167700	-1.62195500
H	-3.87709200	-1.86285500	-2.08453200
C	-7.12928600	-0.97992400	-1.52343000
H	-7.91886600	1.00222300	-1.82118600
H	-6.04117500	-2.83148300	-1.35122200
C	-8.41632700	-1.56138300	-1.00564300
H	-8.64263900	-2.51787000	-1.48701600
H	-8.33736600	-1.75107100	0.07139200
H	-9.25793500	-0.88340000	-1.16780500
C	-2.63733400	0.90517000	-0.08593400
C	-2.72020700	-0.40311200	0.08805800
C	-2.97688500	-1.08649700	1.29216600
C	-4.30519600	-1.07819200	1.91213200
C	-2.04576900	-1.91187400	1.94409000
C	-5.34656900	-0.28797100	1.37277500
C	-4.57779100	-1.86514600	3.07984000
C	-2.30088800	-2.67873000	3.08550500
C	-6.60061000	-0.24947300	1.95165200
H	-5.14526300	0.28614500	0.47747000
C	-5.87977900	-1.81290500	3.64640500
C	-3.55394200	-2.66652800	3.65888500

H	-1.49362300	-3.26687700	3.51107800
C	-6.86969300	-1.02273700	3.10097400
H	-7.38019900	0.36491100	1.51207900
H	-6.08218100	-2.41557300	4.52781200
H	-3.76800300	-3.25259800	4.54618600
H	-7.85718700	-0.99856400	3.55250200
S	-0.19107400	-3.15449500	0.52677800
O	-1.14851200	-4.25363500	0.51446900
O	1.21005400	-3.35769000	0.89129700
C	-2.98443600	3.00957700	-1.20345800
H	-2.54546400	3.62998000	-1.98209300
H	-4.07632300	3.11705000	-1.22986900
C	-2.44814500	3.33765700	0.18532300
H	-2.95801600	4.19024300	0.63659600
H	-1.38443000	3.56097600	0.12331700
C	-2.66123400	2.01691800	0.97367300
H	-3.65634000	2.04332300	1.43581200
C	-1.65782700	1.87822400	2.06283600
H	-1.87308000	2.40599600	2.99085600
C	-0.49632800	1.18901100	1.96488500
H	-0.24759300	0.65425700	1.05832600
C	0.35784300	1.14333500	3.09853300
H	0.03398400	1.66388200	3.99712200
C	2.23550800	0.45667500	4.50318200
C	2.17467100	-0.30588700	2.17945900
C	3.31626400	-0.61083400	4.28234800
H	1.52753400	0.18398900	5.28744200
H	2.63932700	1.44719800	4.72212000
C	2.83418700	-1.38485000	3.04563700
H	1.43386100	-0.70700700	1.49285600
H	4.28362700	-0.14123800	4.08761000
H	3.42517800	-1.25250700	5.15752800
H	3.63663900	-1.90412000	2.52340500
H	2.07395600	-2.12124900	3.31805600
N	1.48832400	0.50522900	3.21220100
C	3.20064500	0.58898300	1.41108500
H	3.79665700	1.11106000	2.16369400
C	2.48301200	1.65758900	0.60070800
C	2.03056400	1.42585100	-0.70224900
C	2.20055000	2.88682700	1.19984200
C	1.30820300	2.40273100	-1.38287800
H	2.23707000	0.48735500	-1.19818400
C	1.45478100	3.85040700	0.52084900
H	2.54764100	3.08730600	2.20758900

C	1.00924100	3.62245600	-0.77801600
H	0.43764100	4.37516200	-1.30611100
C	4.16607800	-0.22933400	0.57463200
C	5.52312800	0.10669400	0.59599200
C	3.74966700	-1.28468100	-0.24096000
C	6.43914400	-0.59238900	-0.18709100
H	5.86347100	0.91795900	1.23011000
C	4.67289000	-1.97488600	-1.02882500
H	2.71038200	-1.58431600	-0.26879000
C	6.02216600	-1.63722800	-1.01130400
H	6.73500800	-2.17921900	-1.61913100
O	-0.70515300	-1.91289500	1.45230500
C	-0.14485400	-2.41597800	-1.20352100
F	0.14856300	-1.11818500	-1.12972000
F	0.82107500	-3.04553700	-1.87391600
F	-1.30263200	-2.61458100	-1.80748900
C	1.02432800	5.08471600	1.25607600
C	0.89136400	2.15827900	-2.80658800
C	4.16862000	-3.07663700	-1.91771700
C	7.88335500	-0.18019800	-0.18728300
F	8.25774500	0.35447400	0.99662100
F	8.70702700	-1.22119600	-0.43815800
F	8.13704200	0.75552000	-1.13389300
F	3.46204600	-3.99732200	-1.22340600
F	3.33969200	-2.59409100	-2.87441300
F	5.16742900	-3.72908100	-2.54848100
F	-0.06318200	4.82878200	2.03405500
F	0.68190500	6.09085600	0.42701100
F	1.98571700	5.54667900	2.08585700
F	-0.27337200	2.77562000	-3.10742700
F	0.74143600	0.84527200	-3.07156800
F	1.81521000	2.63247700	-3.67953000

***S*-D**

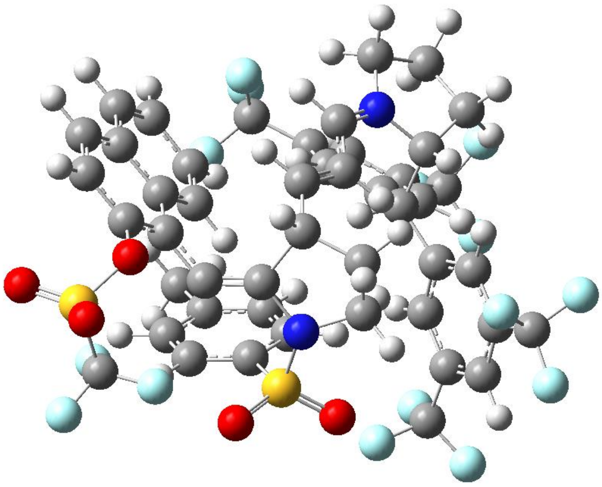

N	2.30344100	2.18992700	0.41754000
S	2.10367400	2.41783500	-1.23576200
O	3.42799300	2.56140800	-1.85740000
O	1.14441900	3.53529200	-1.35456300
C	1.33235500	0.96363300	-1.91219100
C	-0.03129100	0.78212300	-1.69401700
C	2.08350400	0.03551000	-2.63467900
C	-0.65769600	-0.34798600	-2.21693900
H	-0.59491700	1.52349500	-1.14517900
C	1.44024000	-1.08081300	-3.15700100
H	3.14520300	0.18809000	-2.77497000
C	0.06664700	-1.29072800	-2.95794600
H	-1.72143900	-0.49335500	-2.05914000
H	2.01369800	-1.81064700	-3.72021800
C	-0.60063000	-2.51325900	-3.52467600
H	-0.50023500	-2.54043200	-4.61515900
H	-0.13472300	-3.42353300	-3.13389100
H	-1.66254200	-2.54184900	-3.27547300
C	2.80524400	0.92895800	0.92834100
C	3.45976500	0.01196400	0.25928200
C	3.81556000	-1.29537000	0.62207800
C	2.86536400	-2.40210300	0.53959300
C	5.11220800	-1.66171400	1.02034800
C	1.55158900	-2.19151400	0.06466200
C	3.25446200	-3.73044400	0.91803300
C	5.49620000	-2.94911400	1.40196500
C	0.64589000	-3.22816700	-0.02645700
H	1.26831300	-1.19427000	-0.24135500

C	2.28736900	-4.77184900	0.84096700
C	4.58032600	-3.98213600	1.35843600
H	6.51277800	-3.11650000	1.74014500
C	1.01176800	-4.53105400	0.37976200
H	-0.33005100	-3.05069400	-0.45631000
H	2.58476000	-5.77293000	1.14097100
H	4.86534800	-4.98598500	1.65440100
H	0.28715100	-5.33659800	0.31195000
S	7.28182900	-0.48109400	0.08494300
O	7.64100100	-1.78033400	-0.47314300
O	8.26945500	0.37765400	0.72660000
C	1.30118300	2.78154500	1.32550800
H	1.26890500	3.86216700	1.21192400
H	0.30105900	2.38138200	1.12582300
C	1.82519500	2.34091900	2.69095700
H	1.04481300	2.37528500	3.45380500
H	2.63153500	3.00921100	3.00109800
C	2.40582800	0.93127100	2.47015600
H	3.32948300	0.77487400	3.03089400
C	1.49245200	-0.20141100	2.71596700
H	1.95687900	-1.17529000	2.84670800
C	0.12895000	-0.13421500	2.73433100
H	-0.35995500	0.82173700	2.59829000
C	-0.66075600	-1.28307800	2.96541400
H	-0.17738300	-2.23762400	3.15772300
C	-2.80489800	-2.45186300	3.35603200
C	-2.78392300	-0.08561600	2.65103400
C	-4.25270600	-1.98177600	3.15910500
H	-2.59451900	-2.71495600	4.39586400
H	-2.53659800	-3.29819600	2.71987600
C	-4.18009500	-0.44224300	3.17870500
H	-2.37641900	0.75417800	3.20893000
H	-4.64513500	-2.34214900	2.20769500
H	-4.89822300	-2.36433700	3.95121800
H	-4.97052800	0.02374100	2.58842900
H	-4.26702800	-0.07357800	4.20376700
N	-1.97665500	-1.27709900	2.99658700
C	-2.64193500	0.19237600	1.11715700
H	-1.59755400	-0.04361500	0.87791000
C	-2.76394200	1.65487600	0.71104300
C	-2.95421700	1.97472000	-0.64059000
C	-2.44751800	2.69432100	1.58803300
C	-2.78269200	3.27785400	-1.09866200
H	-3.18500800	1.19363900	-1.35423700

C	-2.28818400	4.00173700	1.12220900
H	-2.28065200	2.50654900	2.64052200
C	-2.43806300	4.30805300	-0.22400100
H	-2.27986900	5.31530700	-0.58655400
C	-3.47418900	-0.79767600	0.32980100
C	-2.92742100	-2.06018100	0.09386000
C	-4.79725900	-0.55790200	-0.04929600
C	-3.69491500	-3.08034900	-0.46467000
H	-1.90500000	-2.25675000	0.38959500
C	-5.55100200	-1.57359400	-0.63821400
H	-5.24482700	0.41528100	0.11536800
C	-5.01545200	-2.84636300	-0.83856300
H	-5.61459600	-3.63453800	-1.27608300
O	6.06466100	-0.60464100	1.15516100
C	6.55127900	0.51481300	-1.32392800
F	6.01538900	1.63008000	-0.85530300
F	7.55802400	0.81113300	-2.14958400
F	5.64940700	-0.21251100	-1.97389800
C	-1.87965900	5.05130700	2.11463900
C	-2.81058400	3.54021100	-2.57718400
C	-6.98935500	-1.30995400	-0.98400700
C	-3.06883300	-4.43456500	-0.63675300
F	-2.41019300	-4.81728700	0.48900800
F	-3.97285600	-5.39095500	-0.91882500
F	-2.14800200	-4.44865200	-1.63194200
F	-7.80187300	-1.50866700	0.08238200
F	-7.18381800	-0.03508800	-1.38744900
F	-7.43238200	-2.11924100	-1.96937200
F	-0.70133600	4.72858100	2.71250000
F	-2.78545900	5.17548400	3.11329900
F	-1.72446300	6.26571700	1.55683200
F	-3.19692000	4.80131400	-2.86546800
F	-3.64656200	2.70081700	-3.22800000
F	-1.58178800	3.36986200	-3.12298200

TS$_E$

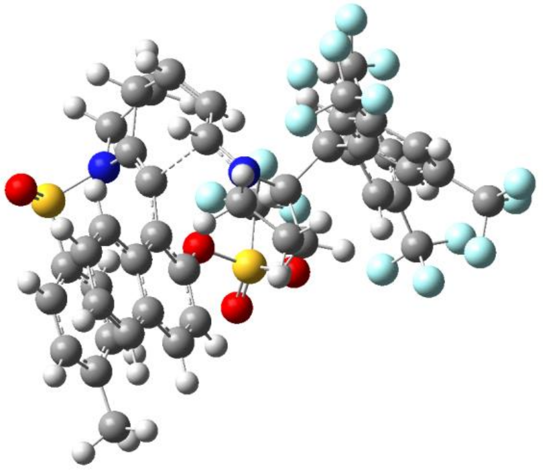

N	-3.96210800	0.11119000	2.00776300
S	-5.37392900	-0.71570600	1.64596100
O	-6.47451700	0.14746800	2.11609800
O	-5.29547500	-2.10250300	2.14137700
C	-5.41721900	-0.77211800	-0.12842600
C	-5.49704900	-2.00563500	-0.77407100
C	-5.39474700	0.42588400	-0.84264000
C	-5.54520200	-2.03192600	-2.16337500
H	-5.49942300	-2.92213200	-0.19772200
C	-5.43976200	0.37770600	-2.23192500
H	-5.31532500	1.37698500	-0.32804900
C	-5.50750800	-0.84687200	-2.91054900
H	-5.58807500	-2.98743600	-2.67637900
H	-5.40057300	1.30175100	-2.79886100
C	-5.47370200	-0.89955600	-4.41274400
H	-4.47543000	-1.19867200	-4.75577300
H	-6.18273000	-1.63563500	-4.80314100
H	-5.70108600	0.07352200	-4.85457500
C	-2.72515300	-0.60027000	2.17377300
C	-2.04816800	-1.32652300	1.30156400
C	-2.21725200	-1.67506000	-0.07803400
C	-2.26656700	-3.06106700	-0.47707300
C	-2.22417500	-0.74441100	-1.11067500
C	-2.34253000	-4.08871400	0.49476600
C	-2.22919200	-3.42005300	-1.86018700
C	-2.21790100	-1.07519300	-2.47570600
C	-2.36610900	-5.41498700	0.11983900
H	-2.38615900	-3.80622000	1.54064300

C	-2.24855300	-4.79464400	-2.21472800
C	-2.18786100	-2.39957500	-2.84579900
H	-2.25879200	-0.30066300	-3.23207200
C	-2.31338800	-5.77267700	-1.24773300
H	-2.42765000	-6.19075600	0.87650300
H	-2.21653400	-5.05825700	-3.26789500
H	-2.16923700	-2.67169300	-3.89566300
H	-2.32959600	-6.82011800	-1.53168900
S	-1.71872300	1.82534000	-1.57064600
O	-2.72070800	2.36987900	-2.47931400
O	-0.38057800	1.50844400	-2.05281400
C	-4.01774200	1.29760900	2.92141900
H	-4.26755600	2.19004200	2.34612500
H	-4.77301100	1.15498100	3.69666200
C	-2.60763500	1.34501200	3.53426800
H	-2.62502400	1.82561700	4.51468200
H	-1.91492800	1.88869400	2.88778000
C	-2.24708800	-0.14524400	3.61230100
H	-2.93764100	-0.59623400	4.33745500
C	-0.89614500	-0.73290900	3.77597900
H	-0.90862400	-1.71070300	4.25852600
C	-0.10789300	-0.61651100	2.67019800
H	-0.09067200	0.32641700	2.13082100
C	0.11979900	-1.81078600	1.90238500
H	-0.06140000	-2.76241700	2.39285400
C	1.17963900	-3.14877300	0.10200300
C	1.25120500	-0.68902600	0.03266000
C	1.92589900	-2.71406200	-1.16609000
H	0.25319000	-3.67708300	-0.12999800
H	1.79321200	-3.79157200	0.74123000
C	1.52589200	-1.24248700	-1.37543700
H	0.39973900	-0.00723000	0.01441300
H	3.00421900	-2.80378800	-1.02178100
H	1.64960000	-3.33489100	-2.02075000
H	2.29611500	-0.68327700	-1.90886000
H	0.60291100	-1.17969500	-1.95597700
N	0.88219200	-1.88236100	0.79769900
C	2.42315100	0.07583900	0.75538800
H	2.20963000	-0.03097200	1.82111500
C	2.34219300	1.56011000	0.47192200
C	2.11710900	2.42309200	1.54700200
C	2.32429400	2.09219900	-0.82322600
C	1.87052300	3.77979300	1.33607100
H	2.08973800	2.02471300	2.55549000

C	2.09050000	3.45046000	-1.02455900
H	2.44802500	1.45093900	-1.68496500
C	1.85935000	4.30781800	0.04988700
H	1.64355800	5.35624600	-0.11607500
C	3.74986800	-0.61327600	0.52581900
C	4.65346100	-0.24662400	-0.47393600
C	4.03136700	-1.74444500	1.29764200
C	5.79078300	-1.01922200	-0.71347100
H	4.48132500	0.64113400	-1.06959400
C	5.15677700	-2.52408100	1.03727500
H	3.34904700	-2.03082300	2.09022800
C	6.04675500	-2.17332200	0.02589900
H	6.92210000	-2.77666100	-0.17635600
O	-2.32480200	0.60897600	-0.69583400
C	-1.61838600	2.97590200	-0.09925100
F	-2.83482600	3.13047900	0.41485400
F	-0.80247200	2.45536400	0.81258300
F	-1.15111500	4.14587700	-0.51810000
C	5.34838800	-3.77545500	1.84190100
C	6.70715000	-0.63942900	-1.84080400
C	1.42169300	4.62673800	2.48829900
C	1.94816000	3.99705100	-2.41488500
F	5.35968700	-3.52367100	3.17160200
F	4.33209300	-4.65243100	1.62985100
F	6.49485700	-4.41884500	1.54372900
F	6.27373100	-1.13705700	-3.02566700
F	7.96232800	-1.10448000	-1.65946500
F	6.79169200	0.70131100	-1.99396200
F	1.65585200	5.94102500	2.28831600
F	2.01921700	4.27933300	3.64881900
F	0.08190500	4.49323100	2.68584700
F	2.39409100	3.14253800	-3.35800800
F	2.62199500	5.16029600	-2.57090600
F	0.64933600	4.26815800	-2.70675400

TS$_E$′

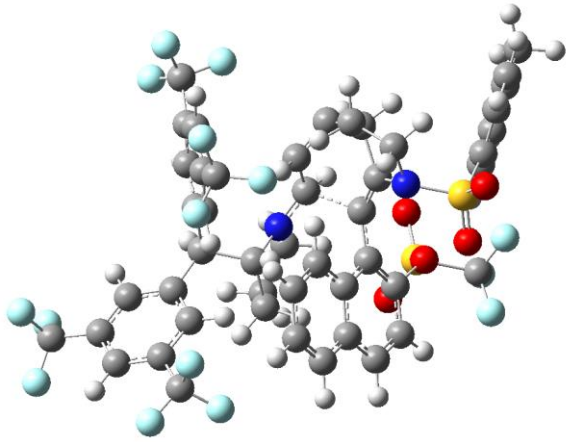

N	2.91230300	1.19660800	2.12007900
S	4.37119300	0.41928400	2.48556800
O	4.15134900	-1.02805600	2.37278500
O	4.81346000	0.98343100	3.77217600
C	5.50089000	0.91067900	1.20494800
C	6.20042500	2.11415900	1.32229700
C	5.56527100	0.14449700	0.04023200
C	6.97428300	2.54512500	0.24848800
H	6.14916400	2.69419800	2.23625500
C	6.34417800	0.59260400	-1.02128600
H	5.02264000	-0.78665800	-0.02196100
C	7.05498600	1.79794200	-0.93656500
H	7.52617900	3.47655000	0.32979900
H	6.39551700	-0.00294400	-1.92772600
C	7.91390400	2.26521700	-2.08024700
H	8.93260500	1.87063400	-1.98288000
H	7.52002000	1.91960500	-3.03965400
H	7.98681600	3.35574600	-2.10367100
C	2.41986700	1.12869300	0.76103200
C	1.81651300	0.09298200	0.17714000
C	1.48831800	-1.17839700	0.78633000
C	0.43201100	-1.29061000	1.76279400
C	2.12054400	-2.35389700	0.42448900
C	-0.25502600	-0.14966000	2.25284600
C	0.04106600	-2.58350700	2.23883500
C	1.78367400	-3.62706800	0.90944000
C	-1.28493700	-0.27697100	3.15980600
H	0.03675400	0.83030300	1.89926800

C	-1.03049500	-2.68065700	3.16563100
C	0.73201300	-3.74075800	1.78754500
H	2.35566400	-4.49086800	0.59238300
C	-1.67866600	-1.55483700	3.61988800
H	-1.79777200	0.60816200	3.52028400
H	-1.32988700	-3.66606600	3.50951700
H	0.43635700	-4.71349300	2.16621700
H	-2.50169800	-1.64543700	4.31875500
S	3.35457500	-2.75373300	-1.90201100
O	3.61323900	-1.63119500	-2.79631000
O	2.29278300	-3.71463900	-2.17770500
C	2.77209400	2.61169500	2.61416400
H	2.26051200	2.60022700	3.57696900
H	3.75230700	3.07544200	2.74675100
C	1.98406100	3.32935000	1.50843900
H	2.20590800	4.39861300	1.49933900
H	0.90825800	3.19701400	1.64121500
C	2.47972800	2.61073100	0.24854700
H	3.53445200	2.88263900	0.10998500
C	1.77578000	2.56414400	-1.06034700
H	2.45100800	2.36326700	-1.89322100
C	0.63013600	1.83198800	-1.03043100
H	-0.06133800	1.94441900	-0.20162000
C	0.74384100	0.51711400	-1.62823700
H	1.55934900	0.43215600	-2.34325500
C	-0.03618700	-1.37390900	-2.97420500
C	-1.37126600	-0.75664900	-1.03550700
C	-1.08234700	-2.46027800	-2.71139200
H	0.97520100	-1.77085400	-2.89753800
H	-0.13872500	-0.92242900	-3.96628300
C	-1.40685500	-2.28219200	-1.22516200
H	-1.12535100	-0.48541900	-0.01168600
H	-1.97637300	-2.29201800	-3.31977600
H	-0.69774500	-3.45516200	-2.94468200
H	-2.35415500	-2.73579800	-0.93500700
H	-0.61659300	-2.72441200	-0.61289800
N	-0.26249200	-0.36014700	-1.92047300
C	-2.70162600	-0.03658600	-1.42429200
H	-2.80041400	-0.08642300	-2.51183900
C	-2.59870200	1.42725500	-1.02957800
C	-2.32354500	2.40627600	-1.98469000
C	-2.66415400	1.80266400	0.31591600
C	-2.09085100	3.72739900	-1.59680100
H	-2.26688700	2.13471100	-3.03278500

C	-2.42007400	3.12052500	0.69605100
H	-2.87842900	1.06588500	1.07959300
C	-2.12477900	4.09670000	-0.25475300
H	-1.92686300	5.11836200	0.04297100
C	-3.94179000	-0.67338200	-0.82417200
C	-3.96216800	-1.20552500	0.46851300
C	-5.12018500	-0.70045800	-1.57693000
C	-5.14044100	-1.74252100	0.99280200
H	-3.06117200	-1.21845600	1.07158000
C	-6.29067400	-1.24011700	-1.04740600
H	-5.11941800	-0.29959300	-2.58470100
C	-6.31325000	-1.76421700	0.24469400
H	-7.22137100	-2.18771800	0.65323700
O	3.28938500	-2.21738200	-0.37487900
C	4.96288500	-3.68617600	-1.72647300
F	5.92772400	-2.83432700	-1.38968900
F	4.83160000	-4.62304400	-0.79519800
F	5.23991000	-4.24494500	-2.90053900
C	-1.68470500	4.73075600	-2.63626400
C	-2.38579300	3.44881800	2.15948000
C	-7.55576800	-1.20653200	-1.85554300
C	-5.11704600	-2.29627300	2.38862700
F	-3.35592000	2.80617000	2.84805400
F	-1.20626500	3.06226400	2.72658200
F	-2.51736400	4.76720600	2.40191200
F	-2.32388100	4.52871900	-3.81075700
F	-1.93896600	6.00024000	-2.25148500
F	-0.35676500	4.65997900	-2.90084400
F	-4.81693300	-1.33537900	3.29827600
F	-4.18002300	-3.26406400	2.52817600
F	-6.29949900	-2.83145000	2.75596100
F	-7.31207500	-1.29060500	-3.18264800
F	-8.39174400	-2.21856500	-1.53434400
F	-8.24513700	-0.05578800	-1.65987700

TS$_{E1}$

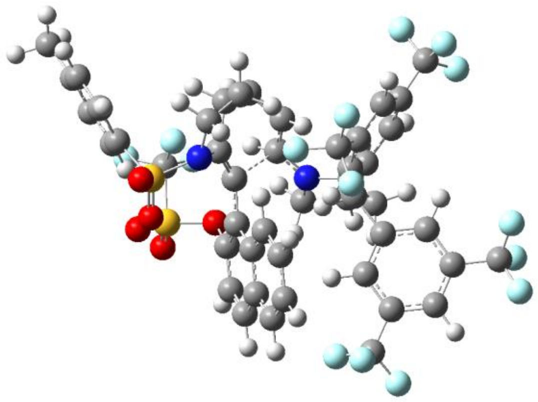

N	-2.66140000	1.34151100	1.61938600
S	-3.93228800	0.58696700	2.41527000
O	-3.68820700	-0.85922900	2.36653000
O	-4.04915800	1.27122500	3.71386200
C	-5.37436800	0.95277400	1.44269900
C	-6.10508400	2.11854500	1.68198800
C	-5.65989100	0.12989700	0.35207900
C	-7.13477200	2.45508900	0.80610200
H	-5.88151100	2.74086200	2.54040900
C	-6.68566800	0.48752700	-0.51537800
H	-5.09237400	-0.77634700	0.20123900
C	-7.43672500	1.65326100	-0.30389400
H	-7.71305400	3.35612100	0.98626100
H	-6.90518300	-0.14881800	-1.36742600
C	-8.56297900	2.01635300	-1.23354800
H	-8.78767000	3.08508600	-1.19015900
H	-9.47705600	1.47488800	-0.96160600
H	-8.32475700	1.75069200	-2.26740500
C	-2.28871400	0.93486600	0.28038300
C	-1.67124300	-0.16710300	-0.12143300
C	-1.20759500	-1.35037400	0.55153600
C	-0.38171000	-1.33770300	1.74235000
C	-1.44261900	-2.60537200	0.00098500
C	-0.02432500	-0.12928200	2.38955700
C	0.11342400	-2.56792700	2.28850800
C	-0.96785000	-3.81957600	0.52138300
C	0.79317400	-0.12550000	3.49995600
H	-0.40313100	0.80198300	1.99863900
C	0.95031500	-2.53220700	3.43510900

C	-0.19920000	-3.80193700	1.65937700
H	-1.20381600	-4.74593300	0.00916300
C	1.29297100	-1.33781400	4.02533700
H	1.06383000	0.81911500	3.96089400
H	1.33671600	-3.46962700	3.82006000
H	0.19105200	-4.72351600	2.07653300
H	1.94596000	-1.32457200	4.89199900
S	-3.67726800	-3.20416000	-1.26035600
O	-3.72611300	-4.52778500	-1.87004100
O	-4.35409700	-2.93497200	0.00131000
C	-2.59307900	2.83608500	1.73044900
H	-2.00519900	3.10445100	2.60762900
H	-3.59475100	3.26462400	1.83172700
C	-1.96260900	3.28109000	0.40292300
H	-2.24625500	4.30651100	0.15666800
H	-0.87192000	3.21563200	0.44498800
C	-2.54343100	2.24898300	-0.57061500
H	-3.62990100	2.40883800	-0.59659100
C	-2.04118500	1.93497300	-1.92597900
H	-2.80550600	1.49816400	-2.56913400
C	-0.82053400	1.32986700	-1.94264200
H	-0.03565400	1.70266100	-1.29402500
C	-0.81990700	-0.07372300	-2.25323500
H	-1.71712900	-0.46825600	-2.71174700
C	0.20051200	-2.14962600	-3.10917500
C	1.65978100	-0.35018400	-2.33730900
C	1.45667300	-2.13006100	-3.97611600
H	0.23947700	-2.97348200	-2.38805100
H	-0.73393500	-2.22835100	-3.66744000
C	2.46748300	-1.45747400	-3.04255400
H	1.70172400	0.56267500	-2.93833000
H	1.28466200	-1.52326000	-4.87133100
H	1.76879300	-3.12959900	-4.28702500
H	3.34226700	-1.04921500	-3.55283300
H	2.82117400	-2.18911600	-2.31199200
N	0.26765800	-0.84891600	-2.42042500
C	2.09559800	-0.00695100	-0.87050300
H	1.27116400	-0.32869200	-0.23256700
C	3.32165700	-0.76376000	-0.38379600
C	4.61182500	-0.38787400	-0.76788000
C	3.16267900	-1.85846400	0.46801500
C	5.71777800	-1.10348200	-0.31257900
H	4.75698000	0.46966500	-1.41551800
C	4.27590300	-2.56545300	0.92578500

H	2.16779500	-2.15887800	0.77244900
C	5.56203700	-2.19629600	0.53957700
H	6.42476400	-2.74428300	0.89623500
C	2.28472400	1.47990000	-0.60346500
C	2.02050000	1.93746300	0.68787600
C	2.72304300	2.40264000	-1.55643400
C	2.12291600	3.28767100	1.00930300
H	1.71759600	1.22628700	1.44401400
C	2.84426500	3.75434600	-1.22313100
H	2.96623600	2.08770600	-2.56415900
C	2.53159200	4.21553800	0.05457100
H	2.60952900	5.26681500	0.29934500
O	-2.14406100	-2.67355700	-1.23616700
C	-4.29500200	-2.00006500	-2.55227000
F	-3.61353300	-2.18453600	-3.68079000
F	-4.13851600	-0.74816400	-2.13375100
F	-5.58766400	-2.24559200	-2.75270600
C	7.08747600	-0.72200400	-0.79530200
C	4.08639300	-3.69800000	1.89411600
C	3.24561300	4.73298300	-2.28899500
C	1.75282000	3.70223100	2.40314700
F	4.01623300	-3.25865600	3.17504500
F	5.10331200	-4.58661800	1.84463400
F	2.94418700	-4.38325500	1.65601800
F	7.36498100	-1.27854500	-2.00042500
F	8.05959500	-1.12293100	0.05209000
F	7.21383300	0.61538200	-0.95107100
F	0.47617500	3.33657400	2.70279500
F	2.53688200	3.10362900	3.33141900
F	1.84102600	5.03191100	2.59560900
F	4.14722400	4.20310300	-3.14710700
F	3.79105300	5.85573700	-1.77353300
F	2.18357400	5.12167500	-3.03683400

TS$_{E1}$′

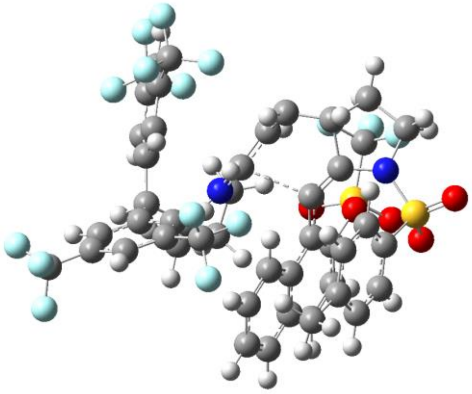

N	3.60863600	-1.00922700	2.04104700
S	4.26666200	-2.45194900	1.54595900
O	4.97394300	-2.35037600	0.25576800
O	5.07289700	-2.91545800	2.69542700
C	2.84050400	-3.48560000	1.31226400
C	1.84358100	-3.53125300	2.29032600
C	2.77026200	-4.27624200	0.16709000
C	0.75583900	-4.37455200	2.09989800
H	1.90575700	-2.90212000	3.17128700
C	1.68235900	-5.12991900	0.00482800
H	3.53784600	-4.20152000	-0.59314400
C	0.66017800	-5.18691700	0.95841700
H	-0.04225600	-4.39165000	2.83555000
H	1.60771700	-5.73005900	-0.89610500
C	-0.53252500	-6.08167300	0.76130800
H	-0.45217200	-6.97990100	1.38525100
H	-0.61780200	-6.40378000	-0.27925300
H	-1.45688300	-5.57162600	1.04361400
C	2.40553400	-0.41026100	1.57899200
C	1.75754200	-0.49195800	0.43232200
C	2.03224100	-1.05168300	-0.85064500
C	1.10957400	-1.97144700	-1.47880600
C	3.14157600	-0.69380600	-1.61378600
C	-0.05606900	-2.40445500	-0.80396600
C	1.36492400	-2.47214700	-2.79496300
C	3.42551800	-1.20057400	-2.89019900
C	-0.93941700	-3.27891800	-1.40029500
H	-0.22521300	-2.05066100	0.20487700
C	0.44161700	-3.37825200	-3.38053300
C	2.53939400	-2.07155000	-3.48209200

H	4.34571400	-0.92787900	-3.39439800
C	-0.68845200	-3.77237000	-2.70067200
H	-1.82239500	-3.61163000	-0.86982700
H	0.64786200	-3.75410000	-4.37845000
H	2.74214000	-2.47022100	-4.47027700
H	-1.38769700	-4.46538900	-3.15778800
S	4.81204500	1.33929200	-1.83481600
O	6.12182000	0.88573800	-2.28657000
O	3.89784700	1.99942900	-2.76166900
C	4.33676200	-0.14926400	3.01813700
H	5.34249100	0.07383600	2.65644900
H	4.41081700	-0.66076800	3.98073000
C	3.44678200	1.10265900	3.09992800
H	3.51563100	1.57319600	4.08272300
H	3.73354100	1.83034400	2.33848600
C	2.04930500	0.53567100	2.81310200
H	1.79649800	-0.11652100	3.65989000
C	0.84871500	1.29241600	2.40047400
H	-0.08126100	0.83262000	2.73291800
C	0.85889900	1.73341200	1.10504000
H	1.73658600	2.21157400	0.68432300
C	-0.03683400	1.06723600	0.21469400
H	-0.80916800	0.47456400	0.68540500
C	0.73379200	2.05109400	-1.91244100
C	-1.32135200	0.71750500	-1.87051700
C	0.51559600	1.45124400	-3.30045900
H	0.50675600	3.12444800	-1.88157800
H	1.74402900	1.90602300	-1.53563600
C	-0.98941700	1.15464900	-3.31156900
H	-1.26463400	-0.36968400	-1.78841800
H	1.09270700	0.52749300	-3.39390100
H	0.82011500	2.13009900	-4.09957500
H	-1.27415100	0.38273900	-4.02954500
H	-1.54738500	2.06294400	-3.56161400
N	-0.21896600	1.30615000	-1.08455700
C	-2.77031100	1.16892500	-1.40662000
H	-3.25977400	1.56762800	-2.29929700
C	-3.63119800	0.01547500	-0.92721300
C	-4.94478000	-0.09672600	-1.38577100
C	-3.16738300	-0.93535400	-0.00953800
C	-5.77319900	-1.12844400	-0.93714400
H	-5.32727600	0.62948700	-2.09483700
C	-3.99560800	-1.96171800	0.43462900
H	-2.15674100	-0.88624400	0.36775600

C	-5.30830400	-2.07038800	-0.02447200
H	-5.95229300	-2.86988200	0.32235900
C	-2.68433000	2.31712500	-0.41011700
C	-2.96748700	2.17399500	0.94784300
C	-2.17498700	3.54090100	-0.85658500
C	-2.70134900	3.21419800	1.84176100
H	-3.37765500	1.24608100	1.32671000
C	-1.89638300	4.56723000	0.04170900
H	-1.96161400	3.68327100	-1.91023100
C	-2.14746500	4.41293200	1.40445400
H	-1.92252500	5.20807800	2.10298600
O	4.07179300	0.18064800	-0.98422300
C	5.08754900	2.46772700	-0.37305900
F	5.67996300	1.79776400	0.60875700
F	3.91957900	2.95366500	0.04285800
F	5.86757900	3.46385400	-0.78004600
C	-1.24332600	5.81255700	-0.48170000
C	-2.94016500	2.97925100	3.30448400
C	-7.16137500	-1.25381300	-1.49705400
C	-3.48770400	-2.98551500	1.40937000
F	-1.85173600	6.26948300	-1.60056700
F	0.05307400	5.58330600	-0.81998800
F	-1.24275000	6.81713200	0.41743300
F	-1.95957200	2.21654600	3.85395100
F	-2.98475100	4.12685300	4.01211400
F	-4.10150600	2.32102200	3.52548000
F	-7.99363700	-1.90317400	-0.65443700
F	-7.71188300	-0.04685000	-1.75779400
F	-7.16942800	-1.94335700	-2.66386500
F	-2.20368300	-2.77056800	1.77534900
F	-4.22651800	-3.02258000	2.53910200
F	-3.53833600	-4.23698500	0.87763600

E

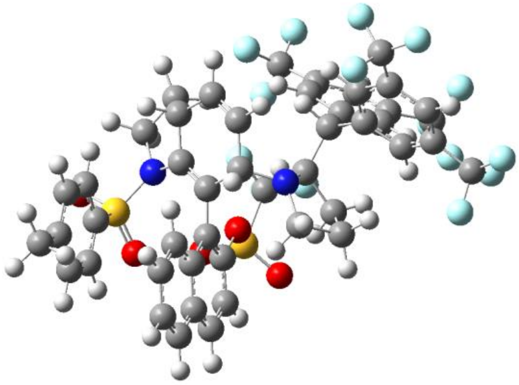

N	3.22113800	-1.38863300	1.26236400
S	4.75827600	-1.51400100	0.57659000
O	4.68228500	-1.76104100	-0.86945500
O	5.44791300	-2.53549000	1.38910100
C	5.54126800	0.06449300	0.81311400
C	5.47884000	0.70710100	2.04886800
C	6.31680200	0.57055700	-0.23187100
C	6.18876800	1.89208400	2.22484300
H	4.89463000	0.29473400	2.86330400
C	7.03455200	1.74234600	-0.02863800
H	6.34089700	0.06126500	-1.18680200
C	6.97465700	2.42478800	1.19438100
H	6.13549000	2.40858900	3.17804300
H	7.63139300	2.14769200	-0.83925200
C	7.70764200	3.72548800	1.37196700
H	7.74942300	4.02292000	2.42239300
H	8.72960900	3.66037500	0.98651500
H	7.20327300	4.52425400	0.81484300
C	2.39987400	-0.22326000	1.35429700
C	2.04711900	0.66057700	0.40614900
C	2.63723800	0.66525900	-0.95735700
C	3.47618800	1.75391200	-1.36835500
C	2.36406900	-0.31626400	-1.88512900
C	3.83439700	2.80258000	-0.48044200
C	3.98404300	1.79229900	-2.70567600
C	2.83948800	-0.29229500	-3.21034700
C	4.62702000	3.84512400	-0.90232200
H	3.51546000	2.75554400	0.55298000
C	4.81314900	2.87308300	-3.10552000
C	3.64254300	0.74988000	-3.60427600

H	2.57238200	-1.07057300	-3.91305200
C	5.12112200	3.88616300	-2.22696300
H	4.89331100	4.63355500	-0.20588300
H	5.19428500	2.88428200	-4.12228000
H	4.01969800	0.78546000	-4.62111100
H	5.75091200	4.71162900	-2.54263500
S	1.39012500	-2.80488800	-2.12402900
O	2.58930600	-3.60938200	-1.94121400
O	0.76128900	-2.70406600	-3.43779500
C	2.89269000	-2.34445000	2.37206500
H	2.69833300	-3.33542000	1.96004600
H	3.72826200	-2.41079000	3.07016100
C	1.66100000	-1.71402200	3.03165200
H	1.60405600	-1.96471300	4.09249100
H	0.74620000	-2.04912600	2.54247900
C	1.88744900	-0.21726700	2.77133900
H	2.73773400	0.08111300	3.40741900
C	0.78242800	0.75194200	3.01452000
H	0.32428900	0.74973600	4.00048400
C	0.42882400	1.65789400	2.10264200
H	-0.33881600	2.39290000	2.32720100
C	0.97061800	1.73230700	0.69678900
H	1.41875100	2.72456300	0.56443000
C	0.01646700	2.38276100	-1.54083000
C	-0.94579200	0.48460100	-0.37499100
C	-1.11739700	1.83182800	-2.40368900
H	0.98010000	2.14546700	-2.00308700
H	-0.02904500	3.47227100	-1.42568300
C	-1.27698300	0.39633800	-1.87793300
H	-0.34303600	-0.37967300	-0.07818400
H	-2.02955400	2.41535500	-2.26198500
H	-0.86763600	1.85929200	-3.46732800
H	-2.25753400	-0.04098200	-2.07764500
H	-0.54118200	-0.24771700	-2.36343700
N	-0.16562300	1.72209200	-0.23815200
C	-2.17152500	0.46037600	0.61406100
H	-1.71002700	0.58445200	1.59364400
C	-2.79115500	-0.92633900	0.64257500
C	-3.84789600	-1.34072200	-0.16926800
C	-2.20329400	-1.87156200	1.49356100
C	-4.28670500	-2.66734300	-0.14350200
H	-4.34654600	-0.64433700	-0.82914900
C	-2.63471800	-3.19423200	1.50536600
H	-1.39232900	-1.56398700	2.14235600

C	-3.68258000	-3.60873800	0.68279700
H	-4.01778600	-4.63874200	0.68720100
C	-3.14377500	1.61330400	0.47706700
C	-3.90229900	1.86899400	-0.66955400
C	-3.28029000	2.49679400	1.55487400
C	-4.74650200	2.97777200	-0.73886700
H	-3.83444000	1.22345500	-1.53402400
C	-4.13302100	3.59800300	1.48519300
H	-2.71229400	2.31999300	2.46180500
C	-4.87261000	3.85572100	0.33270400
H	-5.53002700	4.71328400	0.27359300
O	1.50700100	-1.34771600	-1.43051700
C	0.09200100	-3.40406300	-0.91608700
F	0.45961600	-3.10710300	0.32668600
F	-1.07036500	-2.83236800	-1.20731400
F	-0.00022600	-4.72095700	-1.06059100
C	-4.19722900	4.54728600	2.64585500
C	-5.46419500	3.23168200	-2.03093100
C	-5.46499100	-3.05884800	-0.98766100
C	-1.92190400	-4.22133900	2.33470100
F	-4.11428200	3.90155000	3.83197300
F	-3.17521600	5.43863600	2.62235300
F	-5.34085800	5.26594900	2.65773700
F	-4.60014100	3.61740900	-3.00514100
F	-6.39973200	4.19746600	-1.92752300
F	-6.08131500	2.11781200	-2.49099900
F	-5.47748200	-4.38004200	-1.26827900
F	-5.48370700	-2.39655500	-2.16718000
F	-6.63897900	-2.77858100	-0.36996800
F	-1.14396300	-5.02322800	1.56161700
F	-2.78178900	-5.03690100	2.98504900
F	-1.11110900	-3.66887400	3.26399000

E′

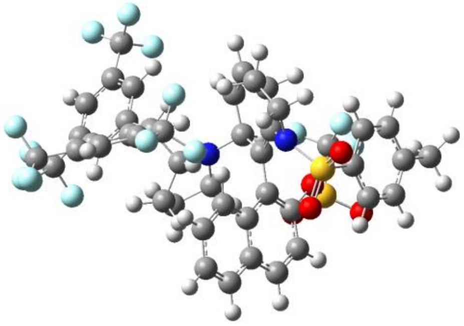

N	2.54356500	2.42323100	-1.32984200
S	3.99577700	2.84860700	-0.57406900
O	3.77540900	3.21945600	0.83061100
O	4.58995900	3.88027600	-1.44674300
C	4.99835400	1.38220800	-0.61417300
C	5.14429600	0.67600800	-1.81056500
C	5.70450500	1.02383800	0.53214400
C	5.98822200	-0.42646400	-1.83881400
H	4.61072700	0.98018000	-2.70383700
C	6.55702100	-0.07595500	0.47838500
H	5.57206400	1.58203500	1.45053500
C	6.70182600	-0.82289400	-0.69663100
H	6.09422800	-0.99318400	-2.75864400
H	7.10156700	-0.37066000	1.36895400
C	7.57033700	-2.05058900	-0.73553000
H	8.21954800	-2.04756400	-1.61665400
H	8.19430100	-2.12662100	0.15739900
H	6.95359400	-2.95455400	-0.79130600
C	1.95362000	1.12316900	-1.37916900
C	1.69302500	0.25921400	-0.38114300
C	2.11300700	0.48930800	1.02927000
C	1.56389500	1.54077300	1.83541300
C	3.02994700	-0.34952900	1.63020600
C	0.68081800	2.51927000	1.30818100
C	1.91541200	1.62612800	3.22137600
C	3.45148600	-0.23475500	2.96754000
C	0.12259100	3.48120400	2.11715300
H	0.46864600	2.52013300	0.24984000
C	1.31863700	2.62769200	4.03264300
C	2.87080200	0.72680300	3.75711900
H	4.22033000	-0.88659400	3.36459300
C	0.42973600	3.52971900	3.49729400

H	-0.54578700	4.21802600	1.68585400
H	1.58737600	2.66836700	5.08394400
H	3.15865400	0.82051400	4.79904600
H	-0.02228600	4.29117900	4.12462900
S	4.03684800	-2.78900900	1.36722500
O	3.01680400	-3.32553300	2.26001900
O	5.43943200	-2.79037300	1.76319900
C	2.14333600	3.22856700	-2.53043500
H	1.71812900	4.18086700	-2.21053500
H	3.01454700	3.42309200	-3.15650400
C	1.12533500	2.33322300	-3.24564900
H	1.16015600	2.48008500	-4.32677400
H	0.10947600	2.54441400	-2.90527800
C	1.55928600	0.92126400	-2.82125600
H	2.48915600	0.69858200	-3.37053500
C	0.61726200	-0.21317900	-3.03609900
H	0.18825000	-0.32822600	-4.02828700
C	0.37213500	-1.10678900	-2.07866600
H	-0.27463900	-1.95754700	-2.27186900
C	0.90118100	-1.03877300	-0.66514000
H	1.56978900	-1.89384500	-0.52981200
C	0.04646500	-2.03278400	1.49832100
C	-1.14434900	-0.18014900	0.51332900
C	-1.19362900	-1.76661300	2.34972500
H	0.93370700	-1.67076400	2.02875500
H	0.20432300	-3.09501200	1.28172900
C	-1.50759600	-0.30401300	2.00357300
H	-0.64033300	0.77185100	0.35243700
H	-2.01564100	-2.42608000	2.05782600
H	-0.99938200	-1.91787400	3.41445700
H	-2.53072300	0.00265500	2.22777500
H	-0.84585400	0.35376600	2.57485800
N	-0.21838600	-1.28682000	0.25883300
C	-2.34019500	-0.14815700	-0.50691100
H	-1.84872700	-0.18456500	-1.48138700
C	-2.99226700	1.22295500	-0.44623200
C	-4.26713300	1.50445600	0.04710900
C	-2.21271800	2.29380500	-0.89902700
C	-4.72812500	2.82348600	0.10090700
H	-4.91566100	0.70886000	0.39027300
C	-2.67407800	3.60462100	-0.84285300
H	-1.22492600	2.09462100	-1.29582800
C	-3.94113400	3.88703900	-0.33691200
H	-4.30556800	4.90478500	-0.28588600

C	-3.25455300	-1.34920700	-0.45953900
C	-4.07755700	-1.65704300	0.62901900
C	-3.24860000	-2.23532200	-1.54322600
C	-4.85249900	-2.81724400	0.63372500
H	-4.11631300	-1.00593200	1.49203900
C	-4.02872600	-3.39111500	-1.53554100
H	-2.62597400	-2.01751200	-2.40392700
C	-4.83650500	-3.69869100	-0.44290800
H	-5.43956500	-4.59723800	-0.43244800
O	3.61228300	-1.33112300	0.78627800
C	3.90972200	-3.68101600	-0.28339300
F	2.65521900	-4.08533600	-0.45677200
F	4.27170900	-2.88486200	-1.28043500
F	4.72382300	-4.72963100	-0.22012000
C	-3.93868200	-4.33993200	-2.69508400
C	-5.65127500	-3.12614300	1.86495700
C	-6.12392400	3.08927000	0.58665700
C	-1.77955300	4.68876000	-1.36768200
F	-3.81574700	-3.68688000	-3.87373900
F	-2.85911700	-5.15420200	-2.59328200
F	-5.02313500	-5.13960900	-2.78952300
F	-4.84786100	-3.51109400	2.88885200
F	-6.54571300	-4.11703900	1.67036000
F	-6.33584500	-2.04371400	2.30454200
F	-6.25158800	4.32457500	1.11990200
F	-6.50993900	2.20144100	1.53091200
F	-7.03069300	3.00334100	-0.41784400
F	-0.54099900	4.63555300	-0.80572300
F	-2.26490600	5.92390600	-1.14192600
F	-1.58315300	4.57034300	-2.70460000

E1

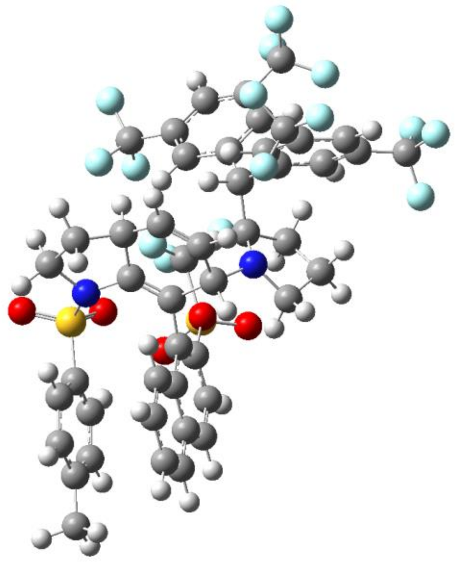

N	-2.96171700	0.05689500	2.30154300
S	-3.93429800	1.24109000	1.60821800
O	-3.18243400	1.97859500	0.58845300
O	-4.48548800	1.99189100	2.75113600
C	-5.27264200	0.37971600	0.82344900
C	-5.87100100	-0.70472800	1.46970400
C	-5.77482700	0.88291100	-0.37386900
C	-6.97974100	-1.30159400	0.88583700
H	-5.46185800	-1.08864300	2.39705600
C	-6.89116800	0.27061400	-0.93992700
H	-5.29007000	1.71745700	-0.86478000
C	-7.50452100	-0.82671400	-0.32697300
H	-7.43739700	-2.16087000	1.36578000
H	-7.27829100	0.64236900	-1.88322800
C	-8.68751200	-1.51044400	-0.95655600
H	-9.56164400	-1.47139800	-0.29736000
H	-8.95752000	-1.04687100	-1.90831500
H	-8.46813500	-2.56768100	-1.13941000
C	-1.84504200	-0.60832800	1.70983600
C	-1.68120500	-1.08025400	0.46090900
C	-2.70480900	-1.00648400	-0.61979900
C	-3.67679600	-2.06211500	-0.70071700
C	-2.67627500	-0.09156200	-1.65208800
C	-3.78002700	-3.05939900	0.30605000
C	-4.57264600	-2.12702000	-1.81451700
C	-3.53017500	-0.15712300	-2.77253000
C	-4.72271500	-4.05926800	0.21511000
H	-3.10835300	-3.02034800	1.15560500

C	-5.53712000	-3.16572000	-1.87544800
C	-4.46862900	-1.15594100	-2.84029800
H	-3.44132800	0.55782200	-3.58090900
C	-5.61290800	-4.11502300	-0.88233800
H	-4.78702100	-4.81016000	0.99626200
H	-6.21488700	-3.19286300	-2.72329400
H	-5.13584600	-1.21395100	-3.69380400
H	-6.35321100	-4.90660400	-0.93760100
S	-1.80771200	2.32797200	-2.33138900
O	-3.14718600	2.87954400	-2.18505600
O	-1.17587300	2.27004300	-3.64637600
C	-2.86069900	0.07003500	3.79356400
H	-2.51239500	1.04460800	4.14436800
H	-3.83540400	-0.13553600	4.23523800
C	-1.82978900	-1.01609400	4.06010200
H	-2.30206600	-2.00353100	4.05766200
H	-1.31864300	-0.87120800	5.01347500
C	-0.88836000	-0.89613500	2.85050800
H	-0.25855000	-0.00310300	3.00087900
C	-0.02334600	-2.08997800	2.60962500
H	0.49786600	-2.50675400	3.46832000
C	0.11992800	-2.61558200	1.39537400
H	0.76813700	-3.47315500	1.23866000
C	-0.53002700	-2.06126500	0.15202200
H	-1.01380700	-2.91123400	-0.34623700
C	0.25703700	-1.91931000	-2.21906200
C	1.11453100	-0.30883200	-0.65319700
C	1.41473200	-1.21429900	-2.91555200
H	-0.70181400	-1.51859300	-2.57827800
H	0.24487600	-3.00138300	-2.38917000
C	1.54669700	0.07544900	-2.09116200
H	0.37629400	0.41728000	-0.29491500
H	2.32079500	-1.82366100	-2.85567200
H	1.20796100	-1.01510400	-3.97005600
H	2.53988200	0.52634000	-2.13675600
H	0.85308900	0.81647100	-2.48593900
N	0.50598200	-1.63546200	-0.79978100
C	2.24597500	-0.28667100	0.43753100
H	1.73146100	-0.52451500	1.37035400
C	2.76273600	1.13183800	0.60191000
C	4.01140000	1.57033800	0.16258000
C	1.90317100	2.06684400	1.19624600
C	4.38136300	2.91236700	0.29464000
H	4.71349700	0.87623800	-0.28128900

C	2.27174700	3.40306300	1.31223100
H	0.93665200	1.74092100	1.56285600
C	3.51742300	3.84241800	0.86056700
H	3.80452100	4.88249500	0.95354900
C	3.29453400	-1.36057500	0.25843200
C	4.09021100	-1.48131000	-0.88548800
C	3.43855400	-2.32879800	1.25551500
C	4.96465800	-2.55751700	-1.03800000
H	4.03020500	-0.74840400	-1.67807700
C	4.30783700	-3.40745700	1.09485500
H	2.84168300	-2.25584800	2.15705300
C	5.07742100	-3.53999800	-0.05751800
H	5.74345000	-4.38251100	-0.18753300
O	-1.64744000	0.88134900	-1.60558600
C	-0.63121100	3.28649000	-1.20957700
F	-0.77999700	2.93542700	0.05517300
F	0.61937500	3.06461500	-1.60126300
F	-0.93599600	4.56984800	-1.37090700
C	4.34306600	-4.44739000	2.17444400
C	5.72514100	-2.66468200	-2.32609500
C	5.75761800	3.32733000	-0.13820700
C	1.30866700	4.41228600	1.86541500
F	4.65196300	-3.91313700	3.38007100
F	3.13020800	-5.04104600	2.32334200
F	5.23684000	-5.42692500	1.92895800
F	4.89915000	-2.95687900	-3.36424500
F	6.67311200	-3.62362600	-2.29490900
F	6.33789600	-1.50033700	-2.64583000
F	5.84980700	4.65704300	-0.35475300
F	6.13438000	2.70625300	-1.27998900
F	6.69170600	3.01550600	0.79403100
F	0.74340600	5.15155300	0.87381600
F	1.91637200	5.29128100	2.69384300
F	0.29733700	3.84164500	2.55245400

E1′

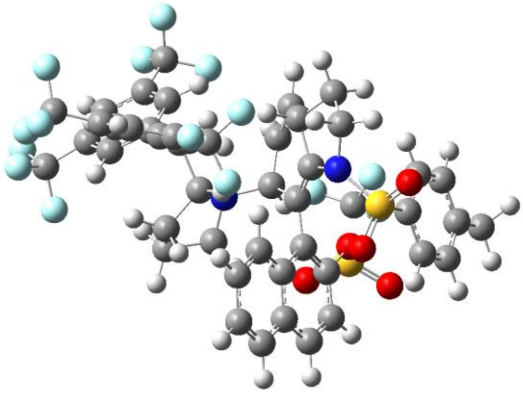

N	2.14175800	2.41561800	-1.49173100
S	3.66146200	2.94674300	-0.94274900
O	3.56306600	3.42366900	0.44169600
O	4.11806400	3.91747400	-1.95453900
C	4.73292500	1.52737900	-0.97434200
C	4.72960500	0.67833100	-2.08261900
C	5.63921500	1.35516100	0.07042800
C	5.63649000	-0.37156000	-2.12457200
H	4.02825000	0.82549500	-2.89471900
C	6.54045600	0.29472800	0.00875200
H	5.61999000	2.01961400	0.92537100
C	6.54551300	-0.58787800	-1.07750100
H	5.62808100	-1.04845500	-2.97308800
H	7.23484300	0.13795600	0.82767900
C	7.46826200	-1.77491800	-1.11782800
H	8.06467200	-1.77953800	-2.03622600
H	8.14758800	-1.78348500	-0.26303600
H	6.89122800	-2.70612700	-1.10236000
C	1.53903000	1.15570400	-1.19947500
C	1.60839600	0.36776200	-0.10834800
C	2.25745600	0.70445400	1.18463700
C	1.76579300	1.74755000	2.04195900
C	3.29600600	-0.06577800	1.66703900
C	0.74619300	2.64919700	1.63880000
C	2.32064400	1.90230400	3.35381100
C	3.90039100	0.11123200	2.92429100
C	0.26366900	3.60971400	2.49799400
H	0.36087300	2.59615100	0.63137000
C	1.80104700	2.90179300	4.21877300
C	3.39296100	1.07013800	3.76556400

H	4.75267400	-0.49180700	3.21478300
C	0.78625600	3.73292700	3.80662100
H	-0.51600300	4.28366900	2.16074400
H	2.22871500	2.99747800	5.21227300
H	3.82519700	1.21493600	4.75018200
H	0.39467900	4.49261400	4.47534200
S	4.18829100	-2.52283100	1.30956200
O	3.22332100	-2.96968000	2.30827000
O	5.62143300	-2.61936700	1.55662700
C	1.67727200	2.89049000	-2.83492300
H	0.79912700	3.52624300	-2.68369700
H	2.45380300	3.47522200	-3.31781100
C	1.31155400	1.60142700	-3.55978900
H	2.20562800	1.11611000	-3.96321500
H	0.60586900	1.77049700	-4.37545500
C	0.70734500	0.77967000	-2.41890500
H	-0.30316900	1.18778200	-2.25772500
C	0.57122800	-0.70199500	-2.58868300
H	0.33664400	-1.07900900	-3.58105600
C	0.65366400	-1.51062900	-1.53349400
H	0.48993300	-2.58029700	-1.63234400
C	0.92242100	-1.01095900	-0.13428900
H	1.61488700	-1.71839500	0.31816500
C	-0.04940900	-1.55522000	2.10009200
C	-1.23062100	-0.00800400	0.69996300
C	-1.37536900	-1.25600100	2.79026100
H	0.75806200	-0.98491000	2.58048100
H	0.23081400	-2.61305600	2.13163300
C	-1.77274600	0.07817500	2.14349100
H	-0.70013400	0.91597800	0.47296400
H	-2.10987500	-2.03571500	2.56799400
H	-1.27080200	-1.18608400	3.87590000
H	-2.84237700	0.29117700	2.18884800
H	-1.26261200	0.89946100	2.65535200
N	-0.28801800	-1.13304500	0.70732800
C	-2.30757500	-0.12501000	-0.43329900
H	-1.73183800	-0.15101100	-1.36051000
C	-3.11015300	1.16486700	-0.48220700
C	-4.48824400	1.26054300	-0.27998700
C	-2.40424100	2.34819100	-0.73482400
C	-5.12597500	2.50468000	-0.30719500
H	-5.08067200	0.37182800	-0.10418600
C	-3.04277900	3.58363800	-0.75911800
H	-1.33742700	2.31105300	-0.91755500

C	-4.41481400	3.67993800	-0.53981200
H	-4.91448800	4.63966200	-0.55237500
C	-3.06809500	-1.42847400	-0.38800500
C	-3.96107400	-1.77382400	0.63162800
C	-2.77884000	-2.39304000	-1.35754900
C	-4.52292300	-3.05003100	0.68420400
H	-4.21216100	-1.06304600	1.40810200
C	-3.34103000	-3.66730800	-1.29898400
H	-2.08034500	-2.15248300	-2.15050000
C	-4.21855200	-4.01299000	-0.27460900
H	-4.64655500	-5.00516100	-0.21990100
O	3.82138600	-1.03474800	0.77281500
C	3.82719500	-3.43505800	-0.30652100
F	2.59855700	-3.93809600	-0.24027600
F	3.92737800	-2.62373200	-1.34859500
F	4.71736600	-4.41703100	-0.40448200
C	-2.97533000	-4.65444100	-2.36764000
C	-5.39427300	-3.38613000	1.85745600
C	-6.61708000	2.55687200	-0.13680300
C	-2.19944200	4.79514800	-1.02638600
F	-3.53296600	-4.33534900	-3.56124600
F	-1.63484300	-4.68922000	-2.57213400
F	-3.36784400	-5.91080200	-2.07155000
F	-4.66110600	-3.51182900	2.99368200
F	-6.06841100	-4.54312100	1.69329000
F	-6.30772000	-2.41679300	2.10003400
F	-7.04289500	3.76855200	0.28155500
F	-7.05450600	1.64398200	0.76064100
F	-7.26438900	2.29444800	-1.29902500
F	-1.22272300	4.94039400	-0.09364200
F	-2.91173300	5.93720500	-1.04124900
F	-1.55950100	4.70246700	-2.22110500

2o

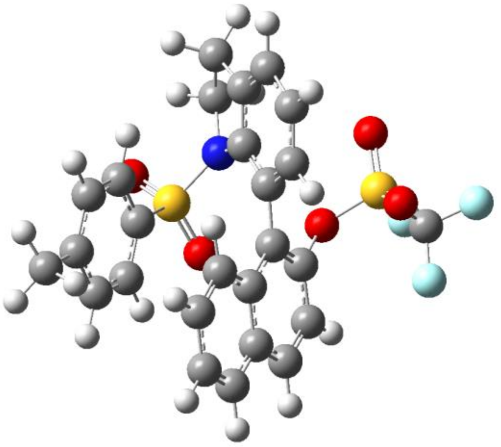

N	-0.21232600	-2.03107900	-0.71992100
S	0.78036000	-1.46016400	-1.96096400
O	0.17702500	-0.27586500	-2.59090900
O	1.03145700	-2.61746800	-2.84288500
C	2.29682900	-0.96408400	-1.18232200
C	2.90410800	-1.79837300	-0.24352800
C	2.90539800	0.21582400	-1.61334800
C	4.13879800	-1.42675700	0.28068300
H	2.42364200	-2.71570700	0.07850800
C	4.14622600	0.55875000	-1.09057100
H	2.40696200	0.85507900	-2.33125500
C	4.77628400	-0.24914100	-0.13377100
H	4.61526200	-2.06015800	1.02227500
H	4.62239400	1.47982300	-1.41072300
C	6.08602000	0.17341600	0.47115400
H	6.75881200	0.58620300	-0.28614600
H	5.92027200	0.95869100	1.21885700
H	6.58715300	-0.66106600	0.96743400
C	-0.13506000	-1.65819100	0.65703200
C	-0.02756200	-0.38823400	1.23931200
C	0.03194500	0.88222800	0.47601000
C	1.12148400	1.80289000	0.63013300
C	-0.99550700	1.24766200	-0.36616800
C	2.23073800	1.53463400	1.47512700
C	1.11457000	3.02627500	-0.11835900
C	-1.02808900	2.44497500	-1.10021600
C	3.26476200	2.43541900	1.59050100
H	2.27121400	0.59796700	2.01539000
C	2.20515000	3.92560300	0.01244800
C	0.02391700	3.31845500	-0.97956200

H	-1.87469000	2.64778700	-1.74467500
C	3.25664900	3.64182600	0.85308400
H	4.10349600	2.20948300	2.24091600
H	2.18838900	4.84546300	-0.56443100
H	0.03064000	4.24786200	-1.53960400
H	4.08321700	4.33854700	0.94873800
S	-3.22776900	0.15778100	0.52682700
O	-2.96266600	0.95352200	1.71780100
O	-3.54848700	-1.26044900	0.59330500
C	-0.72352200	-3.43601200	-0.78543100
H	-1.80734700	-3.38285100	-0.91790900
H	-0.28456700	-3.95816000	-1.63045600
C	-0.36204500	-4.03558700	0.57890800
H	0.59666300	-4.56806600	0.54059800
H	-1.12293200	-4.73864800	0.92419400
C	-0.25395400	-2.81215600	1.45372700
C	-0.25120400	-2.72504500	2.83582500
H	-0.34484200	-3.62239400	3.43935800
C	-0.13471400	-1.46645000	3.43721600
H	-0.14724300	-1.37350500	4.51787900
C	-0.02717700	-0.32823900	2.64841100
H	0.02946600	0.64837200	3.11563700
O	-2.05751400	0.33773200	-0.59103900
C	-4.61300600	0.99429600	-0.40602000
F	-5.71261900	0.93824200	0.33971200
F	-4.28036200	2.26434600	-0.62805100
F	-4.81100900	0.36822300	-1.55952500

***ent*-2o**

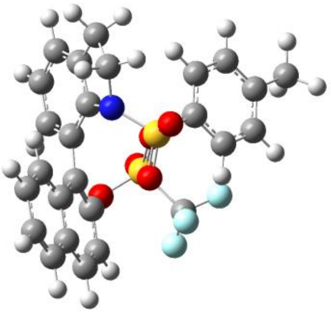

N	-1.16566700	-2.11096700	-0.22853100
S	-0.38072000	-1.52166100	-1.60540200
O	-0.74718000	-0.11402800	-1.79204600
O	-0.73716600	-2.48406400	-2.66388800
C	1.38864200	-1.55494200	-1.35661000
C	2.00333900	-2.54164800	-0.59187900
C	2.13886600	-0.55438300	-1.97871800
C	3.38584300	-2.50404400	-0.42415000
H	1.42961900	-3.32776800	-0.12118800
C	3.51692000	-0.53551600	-1.80472500
H	1.64671100	0.20999400	-2.56540700
C	4.16123600	-1.50037100	-1.01641300
H	3.86739500	-3.26358700	0.18373900
H	4.10164100	0.24886100	-2.27536800
C	5.64872300	-1.43620400	-0.79901300
H	6.18049100	-1.28779100	-1.74405200
H	5.90630800	-0.59252900	-0.14813800
H	6.02421600	-2.34841300	-0.32950700
C	-1.02366600	-1.58284200	1.10140300
C	-1.26326700	-0.28052300	1.57241200
C	-1.73584600	0.83713800	0.72069500
C	-2.95590900	0.72431400	-0.02873200
C	-1.05369300	2.03335400	0.63758100
C	-3.79715100	-0.41474700	0.06904100
C	-3.35804300	1.79298900	-0.89285900
C	-1.44180300	3.10086900	-0.19456300
C	-4.95302400	-0.50617800	-0.67335400
H	-3.52912600	-1.21512400	0.74652700
C	-4.55013000	1.66398300	-1.65202800
C	-2.56791500	2.96929200	-0.96578400
H	-0.83972800	4.00144200	-0.20991600
C	-5.33002100	0.53548800	-1.55219400
H	-5.58299400	-1.38515900	-0.58132800

H	-4.83508300	2.47992400	-2.30943000
H	-2.87796900	3.77414200	-1.62427700
H	-6.23987300	0.44569200	-2.13688700
S	1.55757500	1.75746300	1.27278700
O	1.59295400	0.34592300	0.93564500
O	2.31166400	2.29670400	2.39451900
C	-1.30979600	-3.59956700	-0.09723600
H	-2.38008900	-3.82002900	-0.12058900
H	-0.83836600	-4.10730500	-0.93466400
C	-0.70764900	-3.95044000	1.27387200
H	0.31163400	-4.34578000	1.19812500
H	-1.30838900	-4.70722500	1.78366900
C	-0.73697200	-2.62745900	1.99539000
C	-0.57562100	-2.38181800	3.35157000
H	-0.34714400	-3.19708100	4.03076100
C	-0.73278500	-1.07726500	3.82602000
H	-0.61638900	-0.86242900	4.88300300
C	-1.08797900	-0.05611600	2.95019700
H	-1.26491400	0.94225700	3.33347300
O	0.04879700	2.31684700	1.49447000
C	2.08709500	2.70485100	-0.25413600
F	3.35719600	2.39968600	-0.50703800
F	1.32364600	2.35938500	-1.28785900
F	1.97172600	4.00998600	-0.02525100

References:

1. D. Guo, J. Zhang, B. Zhang and J. Wang, *Org. Lett.,* 2018, **20**, 6284.

2. A. Grandane, S. Belyakov, P. Trapencieris and R. Zalubovskis, *Tetrahedron,* 2012, **68**, 5541.

3. K. Jouvin, A. Coste, A. Bayle, F. Legrand, G. Karthikeyan, K. Tadiparthi and G. Evano, *Organometallics,* 2012, **31**, 7933.

4. X. Yi and X. Hu, *Angew. Chem., Int. Ed.,* 2019, **58**, 4700.

5. H.-H. Zhang, C.-S. Wang, C. Li, G.-J. Mei, Y. Li and F. Shi, *Angew. Chem., Int. Ed.,* 2017, **56**, 116.

6. W. Xia, Q.-J. An, S.-H. Xiang, S. Li, Y.-B. Wang and B. Tan, *Angew. Chem., Int. Ed.,* 2020, **59**, 6775.

7. M. J. Frisch, G. W. Trucks, H. B. Schlegel, G. E. Scuseria, M. A. Robb, J. R. Cheeseman, G. Scalmani, V. Barone, G. A. Petersson, H. Nakatsuji, X. Li, M. Caricato, A. V. Marenich, J. Bloino, B. G. Janesko, R. Gomperts, B. Mennucci, H. P. Hratchian, J. V. Ortiz, A. F. Izmaylov, J. L. Sonnenberg, D. Williams-Young, F. Ding, F. Lipparini, F. Egidi, J. Goings, B. Peng, A. Petrone, T. Henderson, D. Ranasinghe, V. G. Zakrzewski, J. Gao, N. Rega, G. Zheng, W. Liang, M. Hada, M. Ehara, K. Toyota, R. Fukuda, J. Hasegawa, M. Ishida, T. Nakajima, Y. Honda, O. Kitao, H. Nakai, T. Vreven, K. Throssell, J. A. Montgomery, J. E. Jr., Peralta, F. Ogliaro, M. J. Bearpark, J. J. Heyd, E. N. Brothers, K. N. Kudin, V. N. Staroverov, T. A. Keith, R. Kobayashi, J. Normand, K. Raghavachari, A. P. Rendell, J. C. Burant, S. S. Iyengar, J. Tomasi, M. Cossi, J. M. Millam, M. Klene, C. Adamo, R. Cammi, J. W. Ochterski, R. L. Martin, K. Morokuma, O. Farkas, J. B. Foresman and D. J. Fox, *Gaussian 16*, Revision A.03; Gaussian Inc.: Wallingford, CT, 2016.

8. C. Lee, W. Yang and R. G. Parr, *Phys. Rev. B: Condens. Matter Mater. Phys.*, 1988, **37**, 785.

9. A. D. Becke, *J. Chem. Phys.*, 1993, **98**, 5648.

10. S. Grimme, J. Antony, S. Ehrlich and H. Krieg, *J. Chem. Phys.*, 2010, **132**, 154104.

11. R. Ditchfield, W. J. Hehre and J. A. Pople, *J. Chem. Phys.*, 1971, **54**, 724.

12. W. J. Hehre, R. Ditchfield and J. A. Pople, *J. Chem. Phys.*, 1972, **56**, 2257.

13. A. V. Marenich, C. J. Cramer and D. G. Truhlar, *J. Phys. Chem. B*, 2009, **113**, 6378.

14. C. Y. Legault, *CYLView*, *1.0b*; Université de Sherbrooke, 2020. *(http://www.cylview.org)*.

9. HPLC Chromatograms

Compound **2a:** HPLC (IA, *n*-hexane/2-propanol = 70/30, v = 1.0 mL/min, λ = 254 nm)

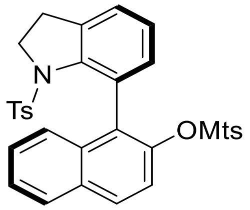

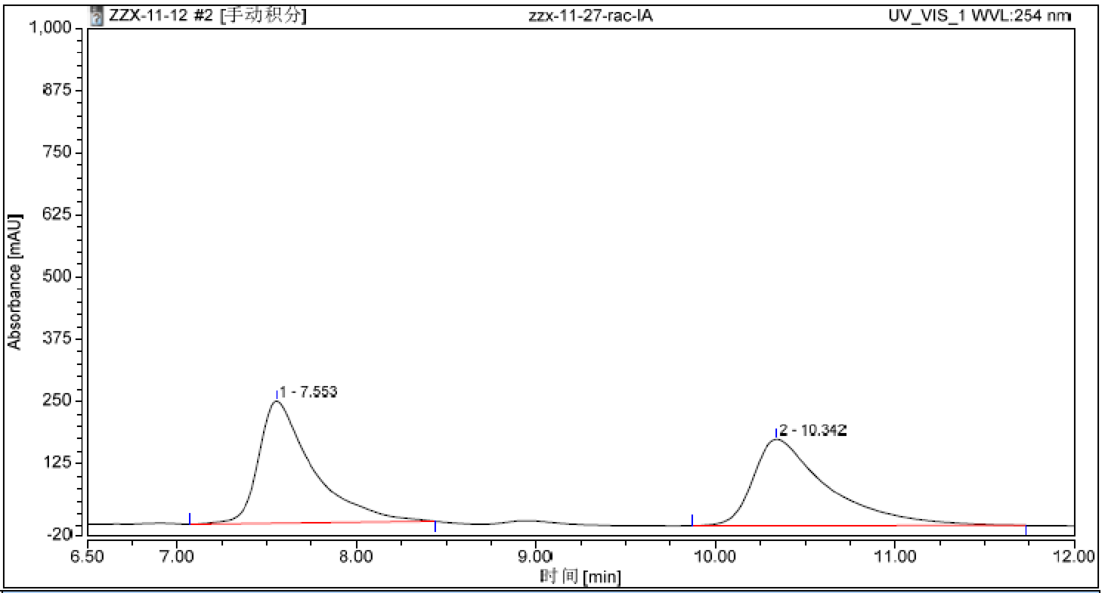

Integration Results							
No.	Peak Name	Retention Time min	Area mAU*min	Height mAU	Relative Area %	Relative Height %	Amount n.a.
1		7.553	85.128	244.591	50.58	58.56	n.a.
2		10.342	83.171	173.097	49.42	41.44	n.a.
Total:			168.299	417.688	100.00	100.00	

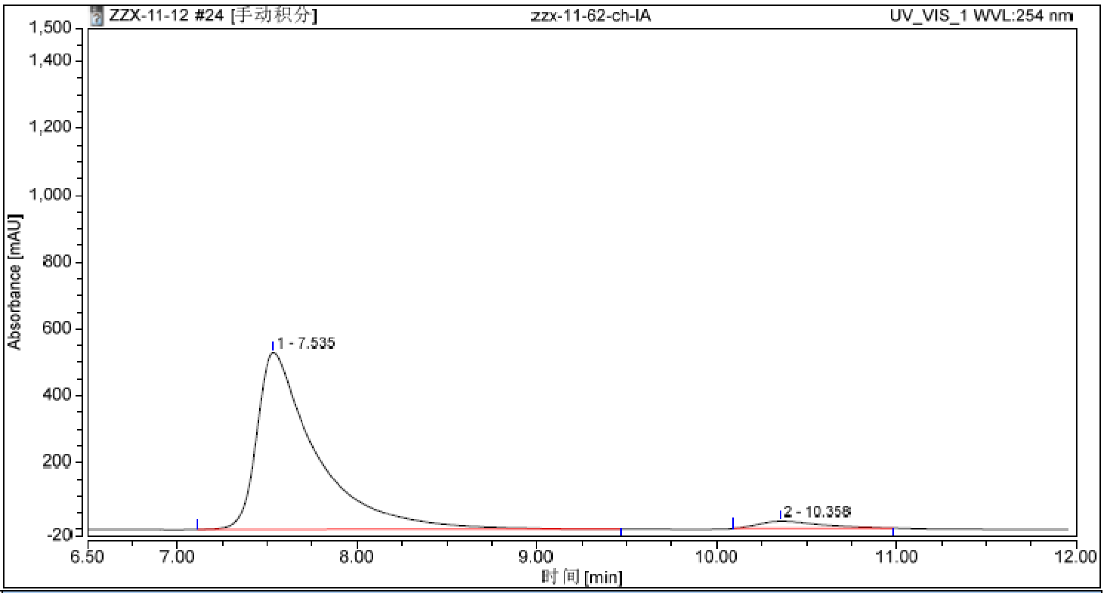

Integration Results							
No.	Peak Name	Retention Time min	Area mAU*min	Height mAU	Relative Area %	Relative Height %	Amount n.a.
1		7.535	202.673	529.135	96.00	96.15	n.a.
2		10.358	8.445	21.165	4.00	3.85	n.a.
Total:			211.119	550.300	100.00	100.00	

Compound **2b:** HPLC (IA, *n*-hexane/2-propanol = 70/30, v = 1.0 mL/min, λ = 254 nm)

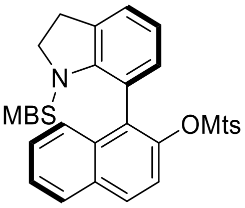

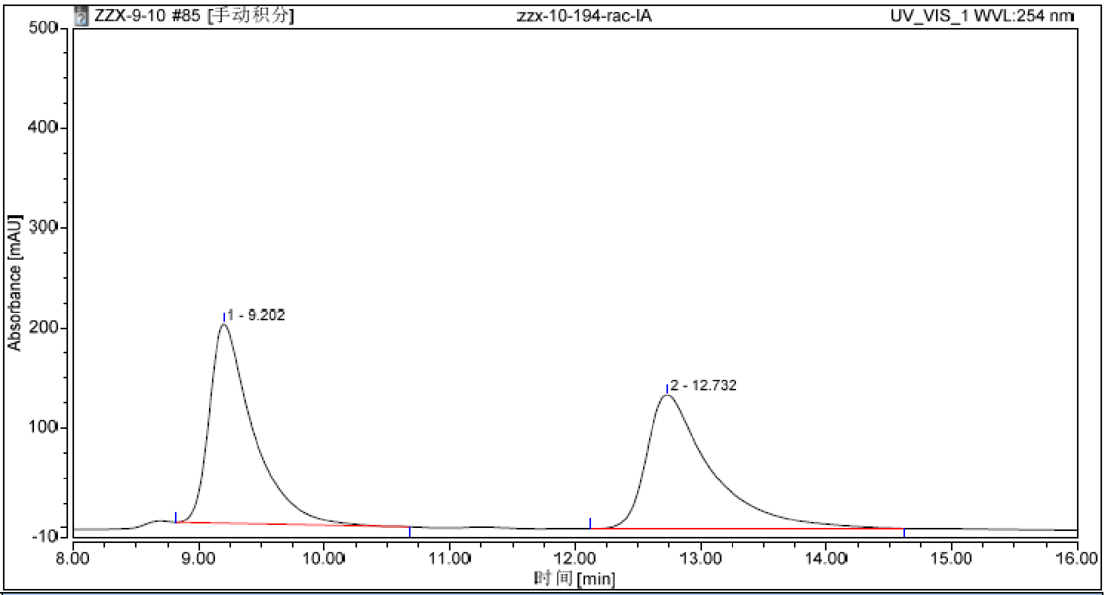

Integration Results

No.	Peak Name	Retention Time min	Area mAU*min	Height mAU	Relative Area %	Relative Height %	Amount n.a.
1		9.202	80.771	199.326	50.32	59.74	n.a.
2		12.732	79.747	134.338	49.68	40.26	n.a.
**Total:**			**160.518**	**333.664**	**100.00**	**100.00**	

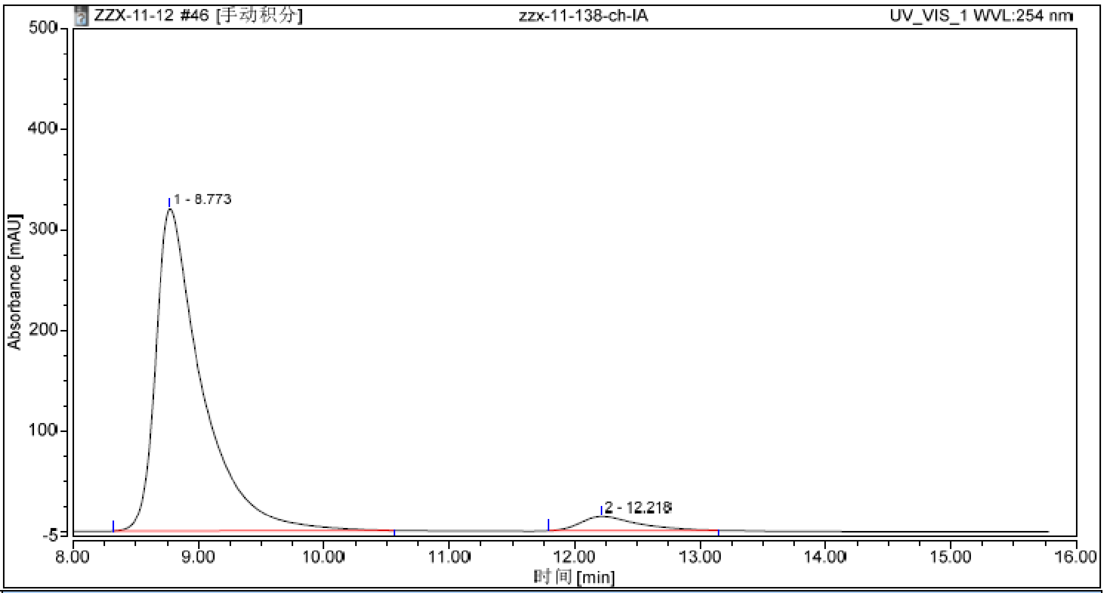

Integration Results

No.	Peak Name	Retention Time min	Area mAU*min	Height mAU	Relative Area %	Relative Height %	Amount n.a.
1		8.773	136.302	320.229	94.88	95.82	n.a.
2		12.218	7.362	13.967	5.12	4.18	n.a.
**Total:**			**143.664**	**334.196**	**100.00**	**100.00**	

Compound **2c:** HPLC (IA, *n*-hexane/2-propanol = 70/30, v = 1.0 mL/min, λ = 254 nm)

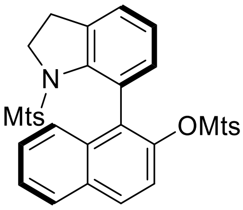

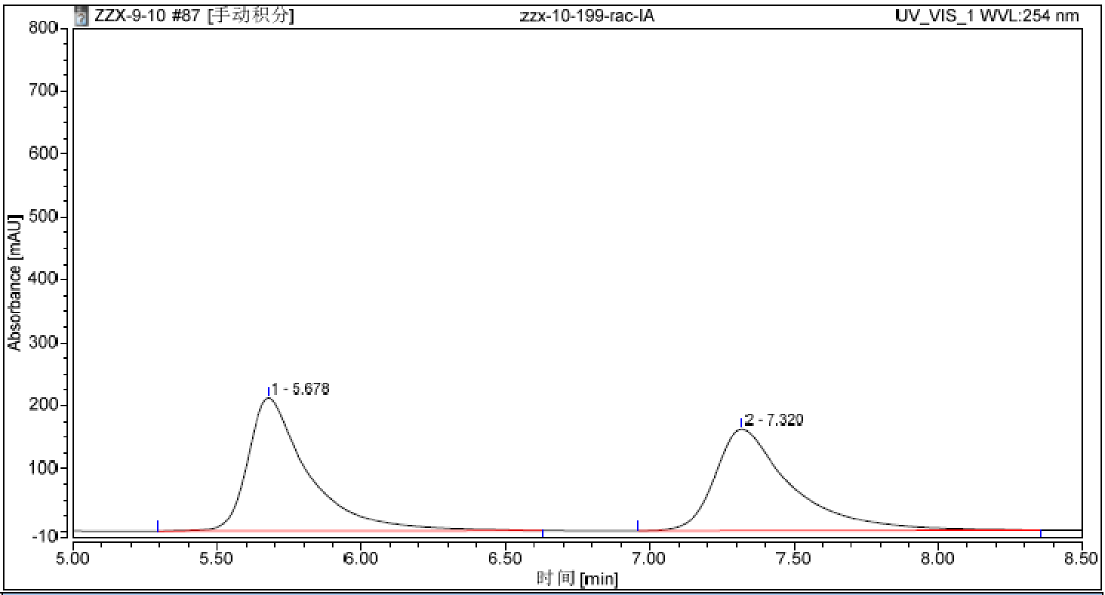

Integration Results

No.	Peak Name	Retention Time min	Area mAU*min	Height mAU	Relative Area %	Relative Height %	Amount n.a.
1		5.678	51.331	211.118	50.82	56.62	n.a.
2		7.320	49.676	161.781	49.18	43.38	n.a.
**Total:**			**101.008**	**372.900**	**100.00**	**100.00**	

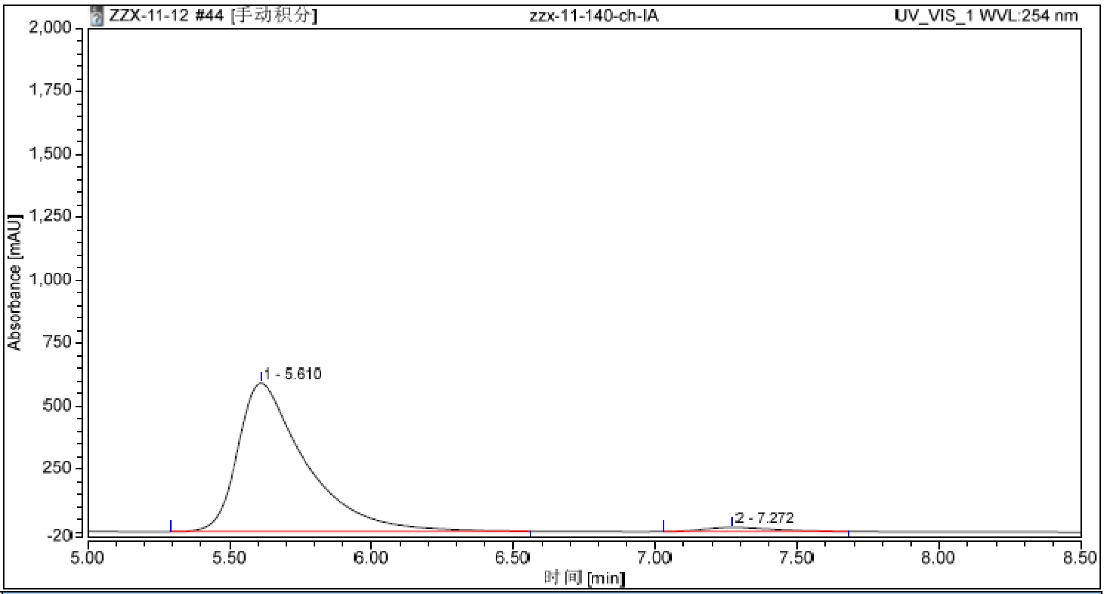

Integration Results

No.	Peak Name	Retention Time min	Area mAU*min	Height mAU	Relative Area %	Relative Height %	Amount n.a.
1		5.610	163.397	589.432	97.25	97.31	n.a.
2		7.272	4.624	16.314	2.75	2.69	n.a.
**Total:**			**168.021**	**605.746**	**100.00**	**100.00**	

Compound **2d:** HPLC (ADH, *n*-hexane/2-propanol = 70/30, v = 1.0 mL/min, λ = 254 nm)

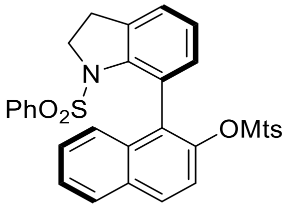

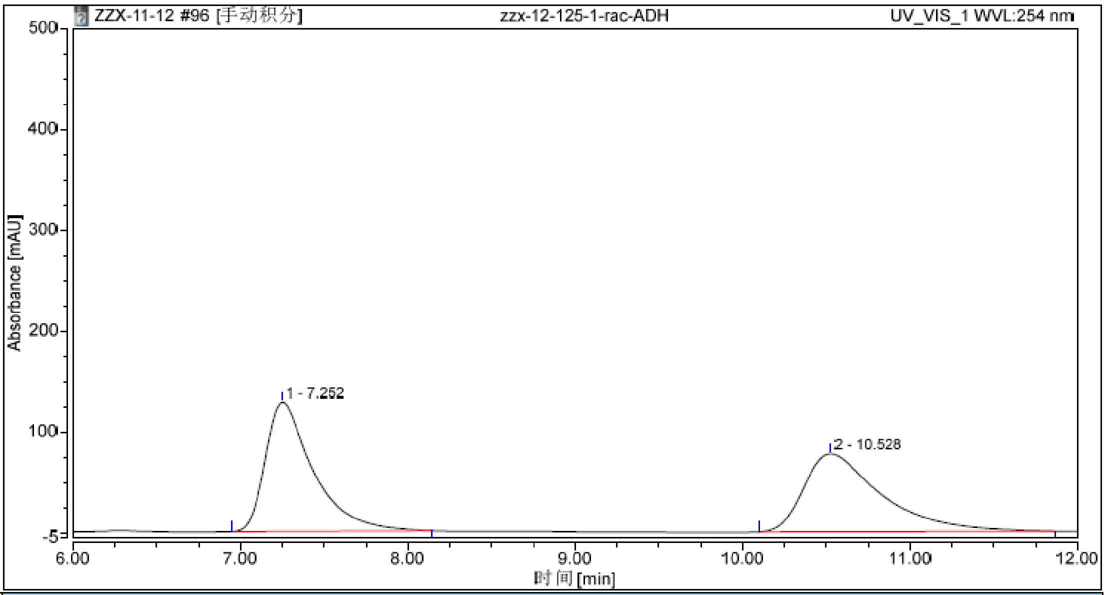

Integration Results

No.	Peak Name	Retention Time min	Area mAU*min	Height mAU	Relative Area %	Relative Height %	Amount n.a.
1		7.252	42.300	127.880	50.61	62.34	n.a.
2		10.528	41.281	77.251	49.39	37.66	n.a.
**Total:**			**83.581**	**205.131**	**100.00**	**100.00**	

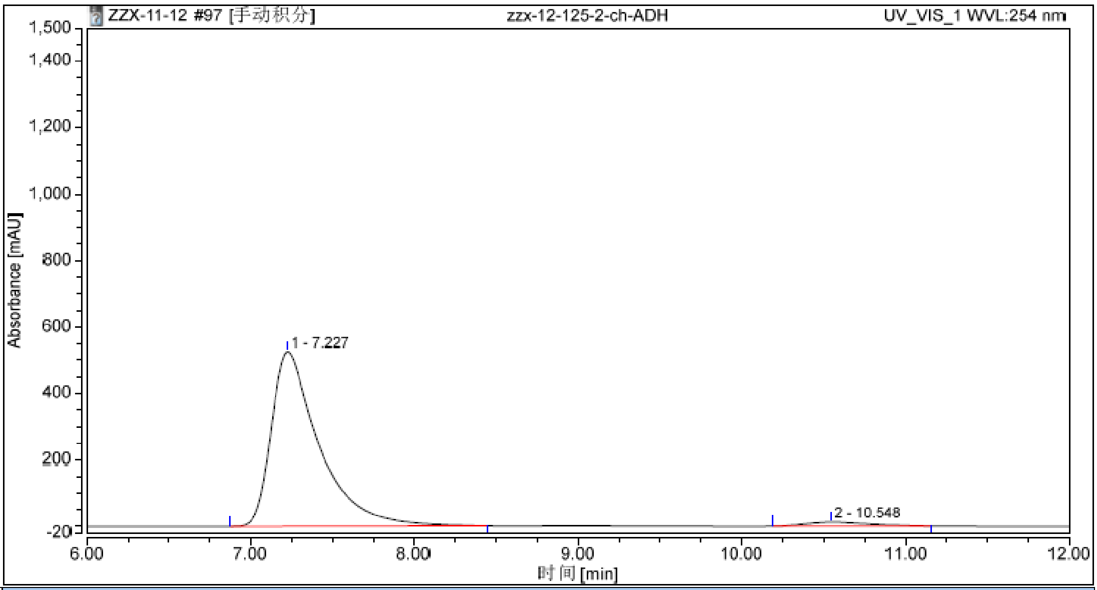

Integration Results

No.	Peak Name	Retention Time min	Area mAU*min	Height mAU	Relative Area %	Relative Height %	Amount n.a.
1		7.227	177.848	524.188	97.11	97.77	n.a.
2		10.548	5.299	11.949	2.89	2.23	n.a.
**Total:**			**183.148**	**536.137**	**100.00**	**100.00**	

Compound **2e:** HPLC (IA, *n*-hexane/2-propanol = 70/30, v = 1.0 mL/min, λ = 254 nm)

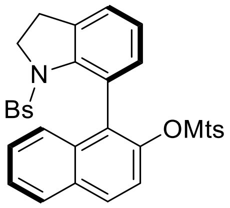

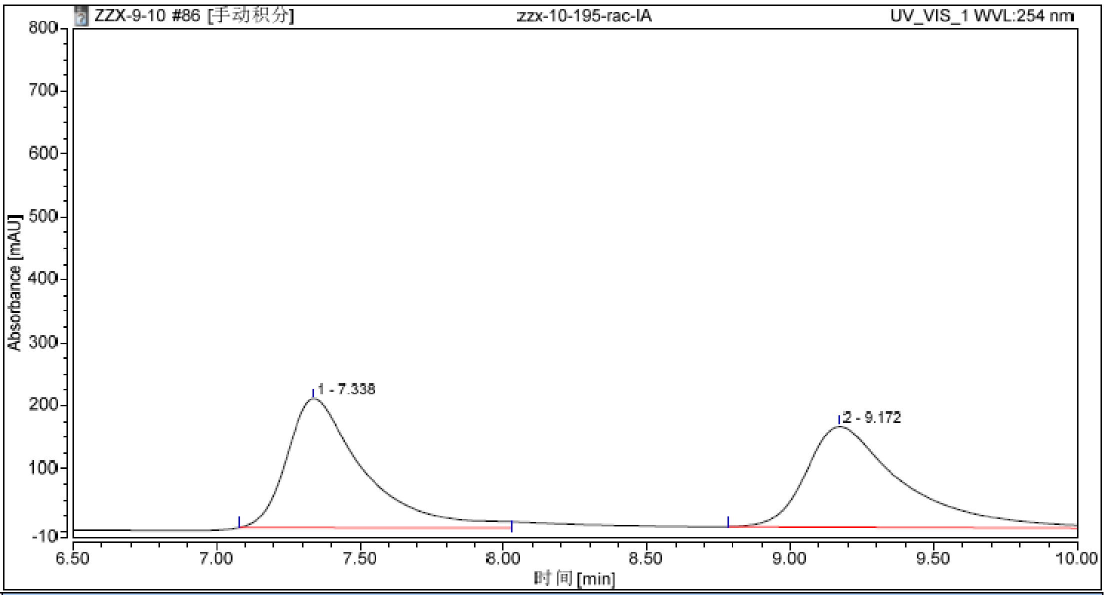

Integration Results

No.	Peak Name	Retention Time min	Area mAU*min	Height mAU	Relative Area %	Relative Height %	Amount n.a.
1		7.338	63.482	205.186	50.19	56.16	n.a.
2		9.172	63.006	160.142	49.81	43.84	n.a.
**Total:**			**126.488**	**365.328**	**100.00**	**100.00**	

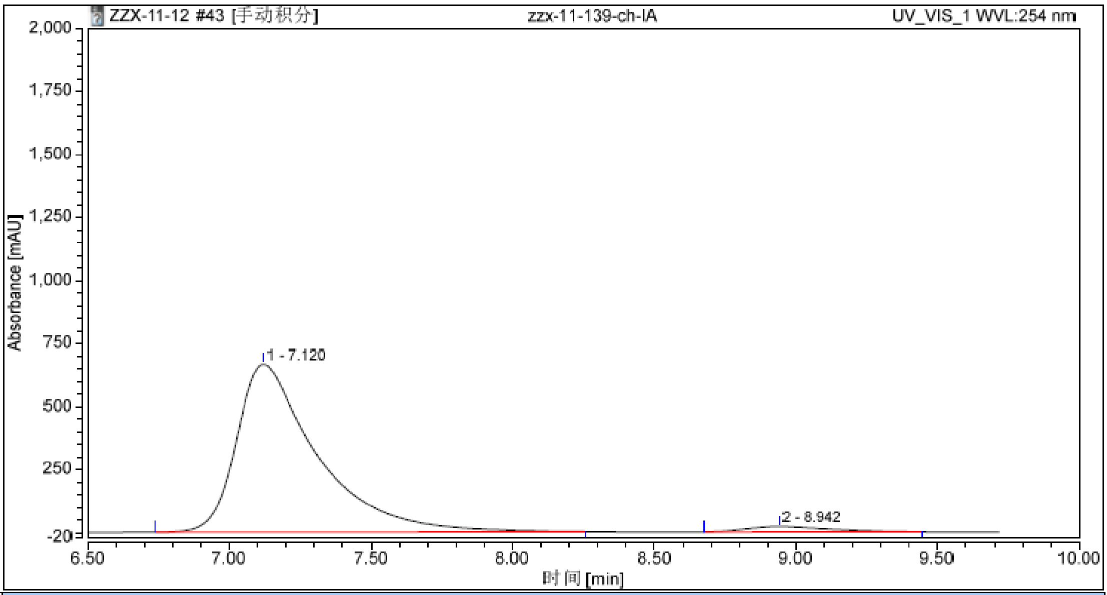

Integration Results

No.	Peak Name	Retention Time min	Area mAU*min	Height mAU	Relative Area %	Relative Height %	Amount n.a.
1		7.120	219.064	665.235	96.93	96.97	n.a.
2		8.942	6.940	20.804	3.07	3.03	n.a.
**Total:**			**226.004**	**686.039**	**100.00**	**100.00**	

Compound **2f:** HPLC (IA, *n*-hexane/2-propanol = 70/30, v = 1.0 mL/min, λ = 254 nm)

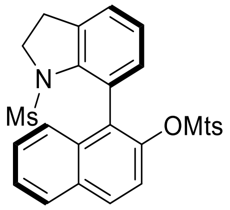

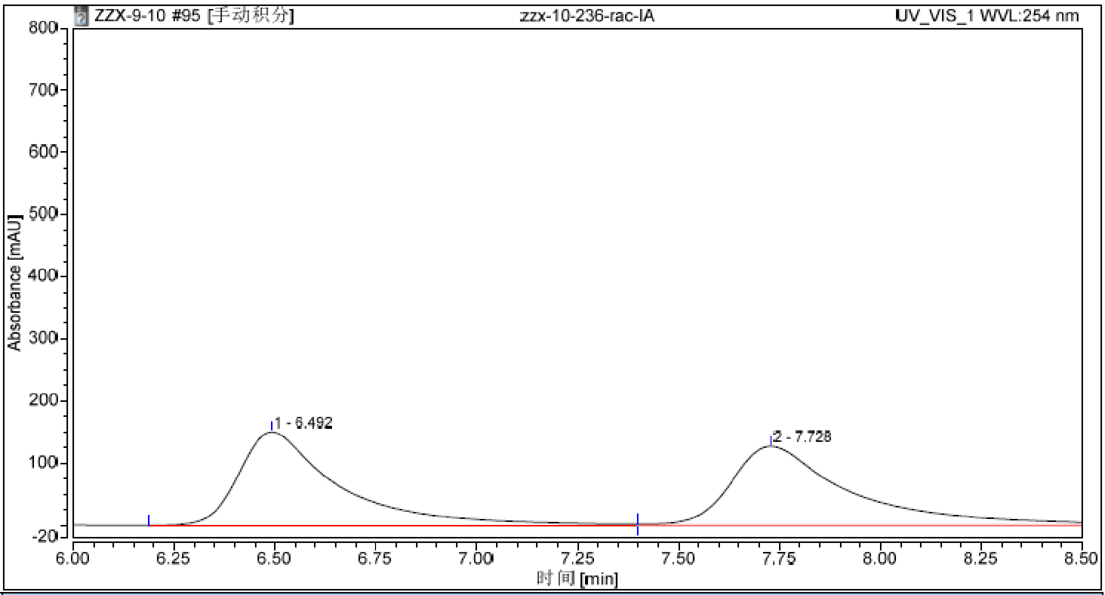

Integration Results

No.	Peak Name	Retention Time min	Area mAU*min	Height mAU	Relative Area %	Relative Height %	Amount n.a.
1		6.492	43.311	150.301	49.31	54.08	n.a.
2		7.728	44.516	127.617	50.69	45.92	n.a.
Total:			87.827	277.919	100.00	100.00	

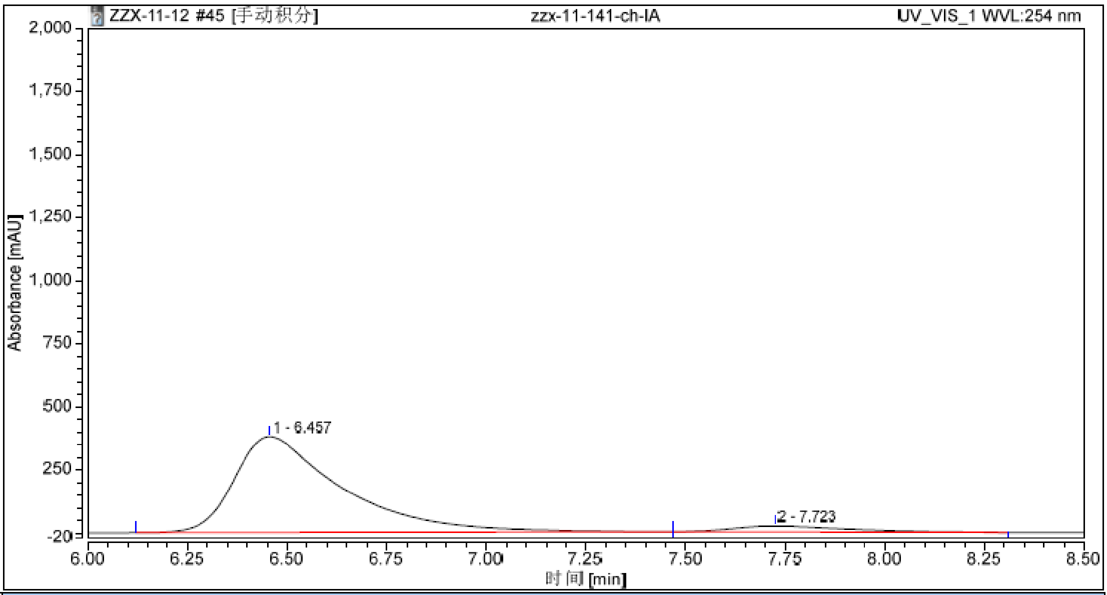

Integration Results

No.	Peak Name	Retention Time min	Area mAU*min	Height mAU	Relative Area %	Relative Height %	Amount n.a.
1		6.457	118.527	379.312	94.06	94.17	n.a.
2		7.723	7.490	23.490	5.94	5.83	n.a.
Total:			126.017	402.802	100.00	100.00	

Compound **2g:** HPLC (IA, *n*-hexane/2-propanol = 70/30, v = 1.0 mL/min, λ = 254 nm)

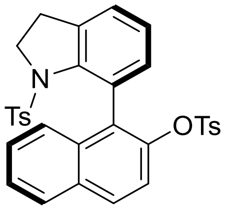

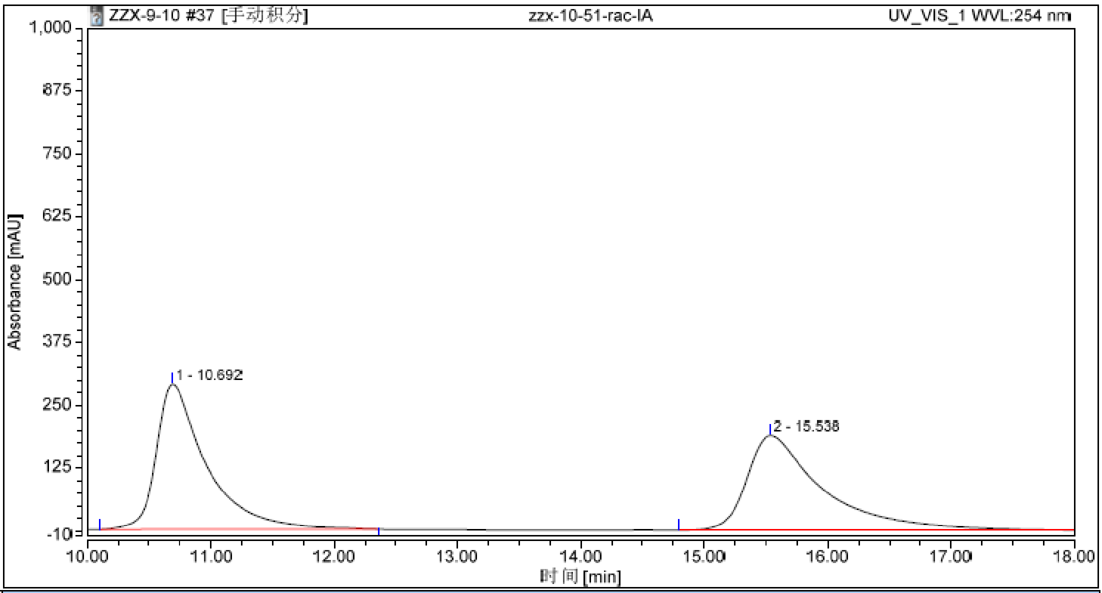

Integration Results							
No.	Peak Name	Retention Time min	Area mAU*min	Height mAU	Relative Area %	Relative Height %	Amount n.a.
1		10.692	135.800	288.920	50.40	60.55	n.a.
2		15.538	133.639	188.229	49.60	39.45	n.a.
**Total:**			**269.438**	**477.149**	**100.00**	**100.00**	

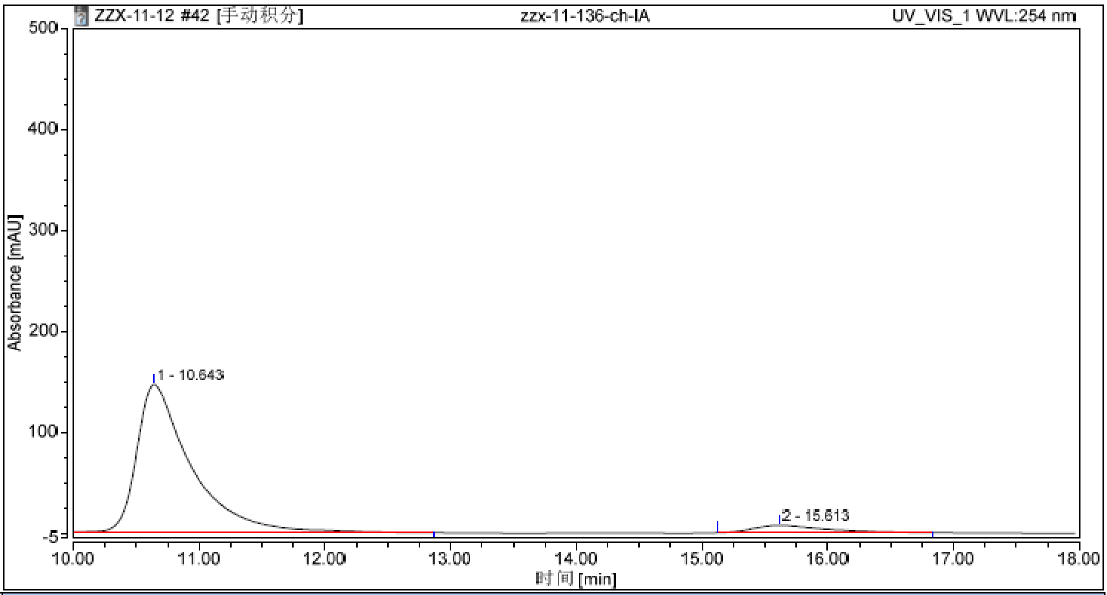

Integration Results							
No.	Peak Name	Retention Time min	Area mAU*min	Height mAU	Relative Area %	Relative Height %	Amount n.a.
1		10.643	73.929	146.777	93.86	95.26	n.a.
2		15.613	4.832	7.304	6.14	4.74	n.a.
**Total:**			**78.761**	**154.081**	**100.00**	**100.00**	

Compound **2h:** HPLC (IA, *n*-hexane/2-propanol = 90/10, v = 1.0 mL/min, λ = 254 nm)

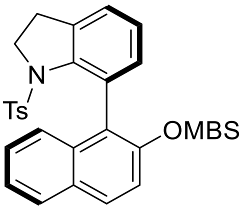

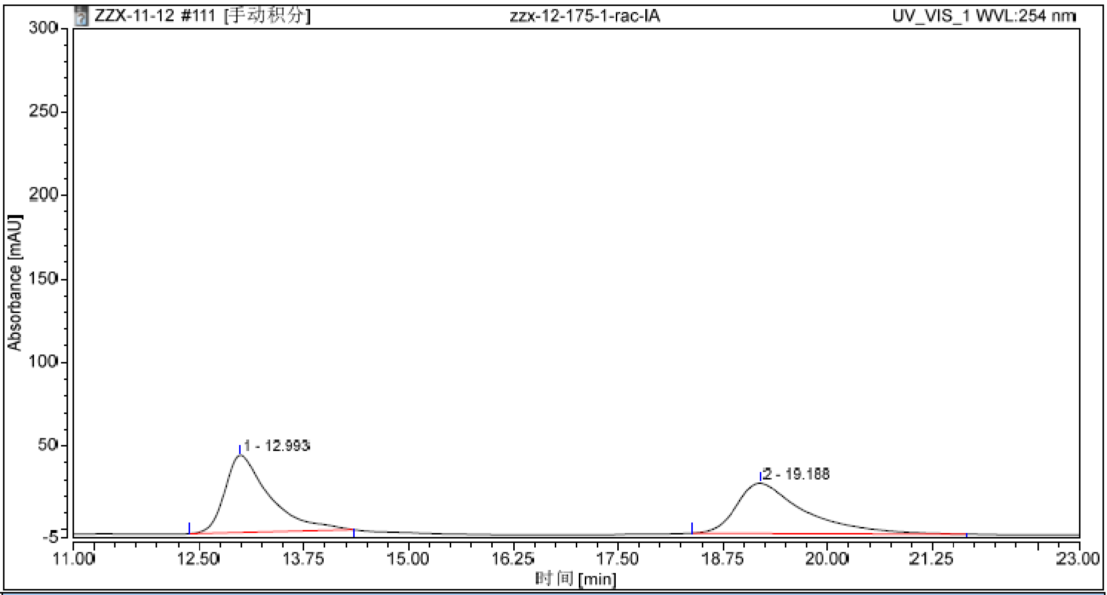

Integration Results

No.	Peak Name	Retention Time min	Area mAU*min	Height mAU	Relative Area %	Relative Height %	Amount n.a.
1		12.993	28.363	46.060	50.37	60.54	n.a.
2		19.188	27.942	30.019	49.63	39.46	n.a.
**Total:**			**56.304**	**76.079**	**100.00**	**100.00**	

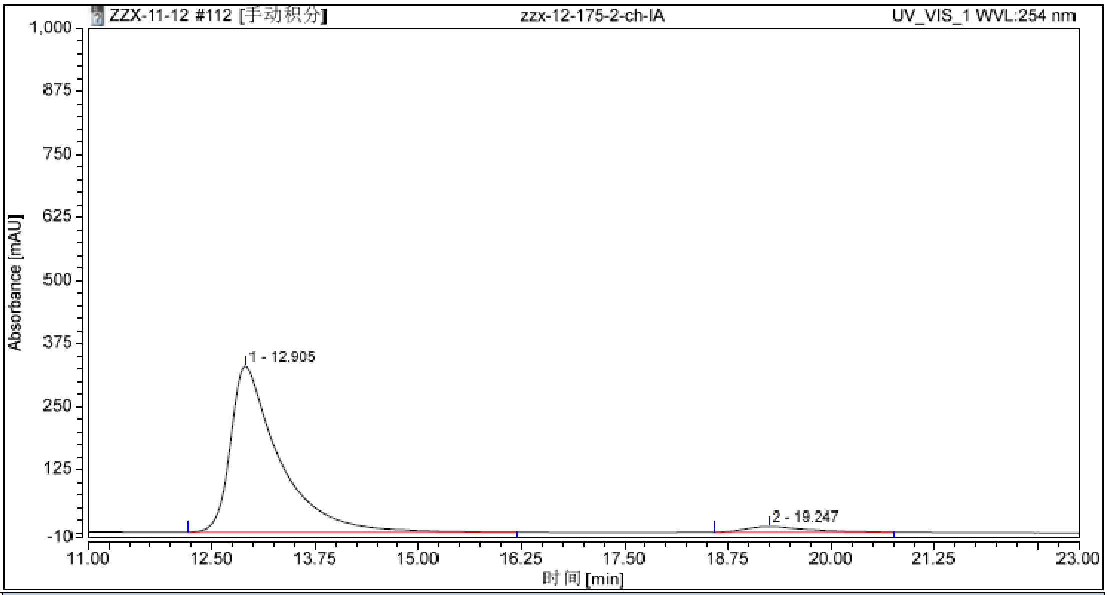

Integration Results

No.	Peak Name	Retention Time min	Area mAU*min	Height mAU	Relative Area %	Relative Height %	Amount n.a.
1		12.905	225.347	328.913	95.90	96.65	n.a.
2		19.247	9.632	11.386	4.10	3.35	n.a.
**Total:**			**234.978**	**340.299**	**100.00**	**100.00**	

Compound **2i:** HPLC (IA, *n*-hexane/2-propanol = 70/30, v = 1.0 mL/min, λ = 254 nm)

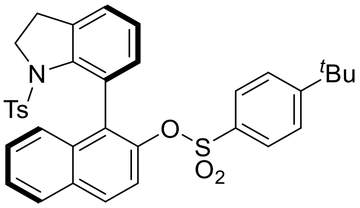

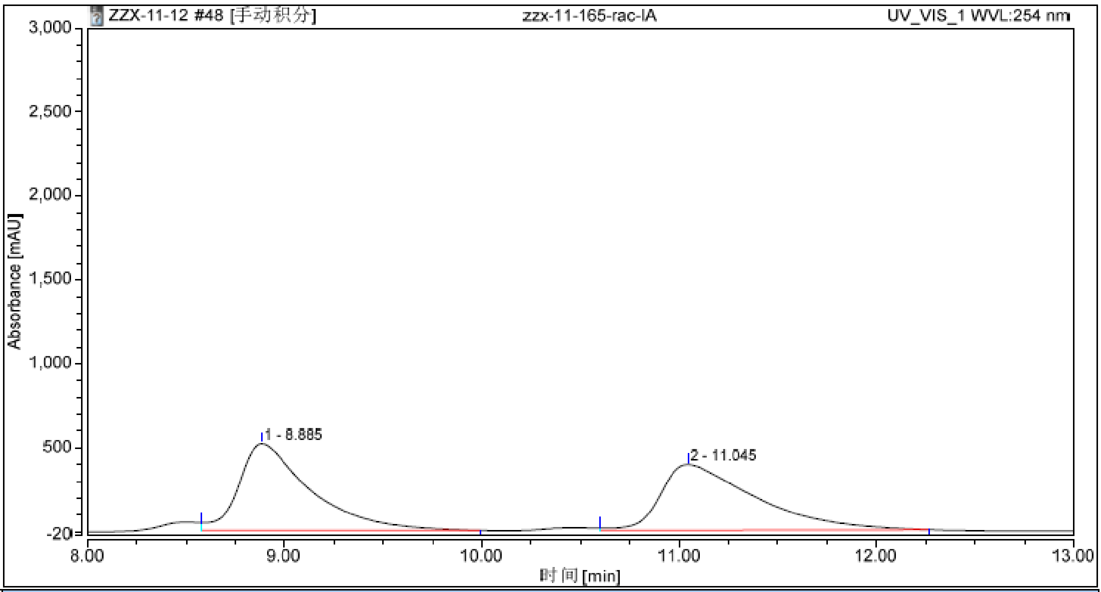

Integration Results

No.	Peak Name	Retention Time min	Area mAU*min	Height mAU	Relative Area %	Relative Height %	Amount n.a.
1		8.885	215.606	515.561	49.77	56.86	n.a.
2		11.045	217.600	391.101	50.23	43.14	n.a.
Total:			433.206	906.662	100.00	100.00	

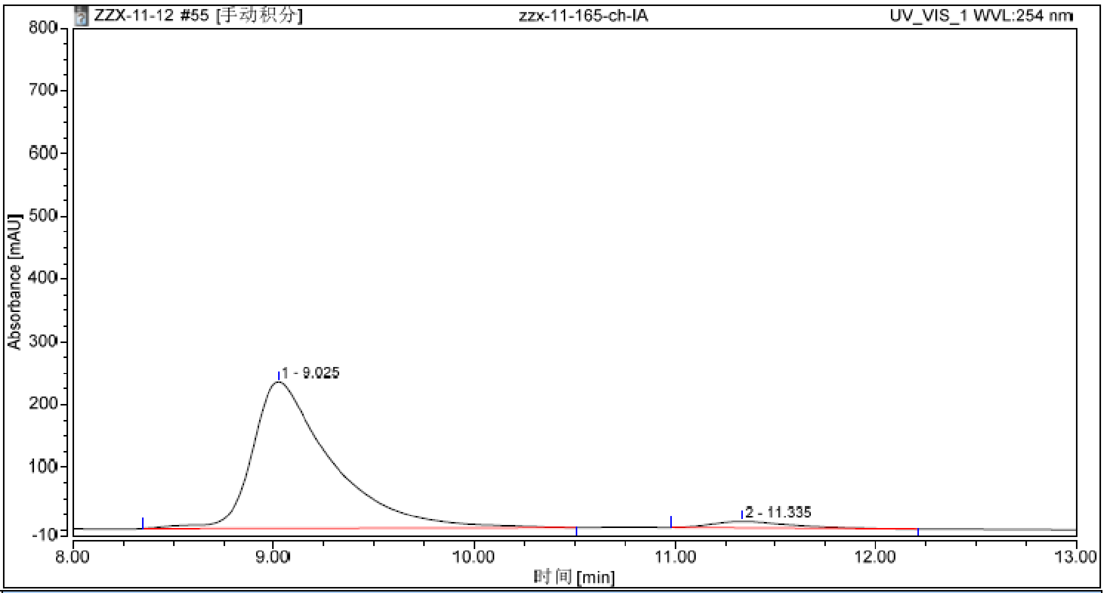

Integration Results

No.	Peak Name	Retention Time min	Area mAU*min	Height mAU	Relative Area %	Relative Height %	Amount n.a.
1		9.025	106.719	233.311	95.89	95.81	n.a.
2		11.335	4.579	10.194	4.11	4.19	n.a.
Total:			111.299	243.505	100.00	100.00	

Compound **2j:** HPLC (IA, *n*-hexane/2-propanol = 70/30, v = 1.0 mL/min, λ = 254 nm)

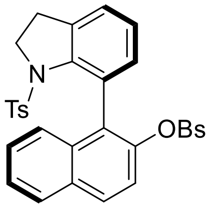

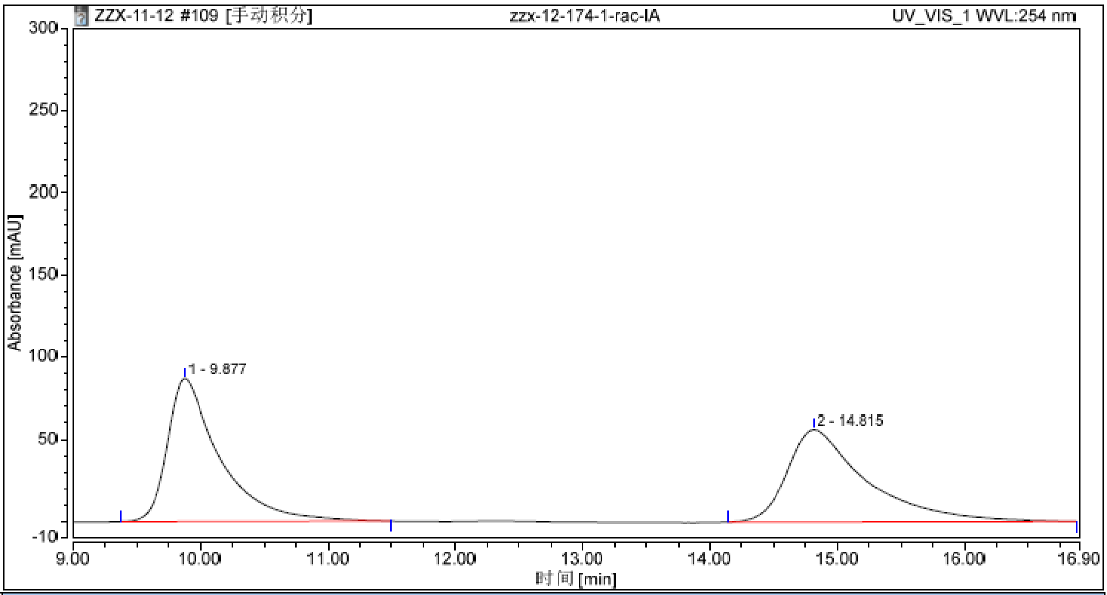

Integration Results

No.	Peak Name	Retention Time<br>min	Area<br>mAU*min	Height<br>mAU	Relative Area<br>%	Relative Height<br>%	Amount<br>n.a.
1		9.877	41.199	86.938	50.63	60.82	n.a.
2		14.815	40.173	56.004	49.37	39.18	n.a.
**Total:**			**81.372**	**142.943**	**100.00**	**100.00**	

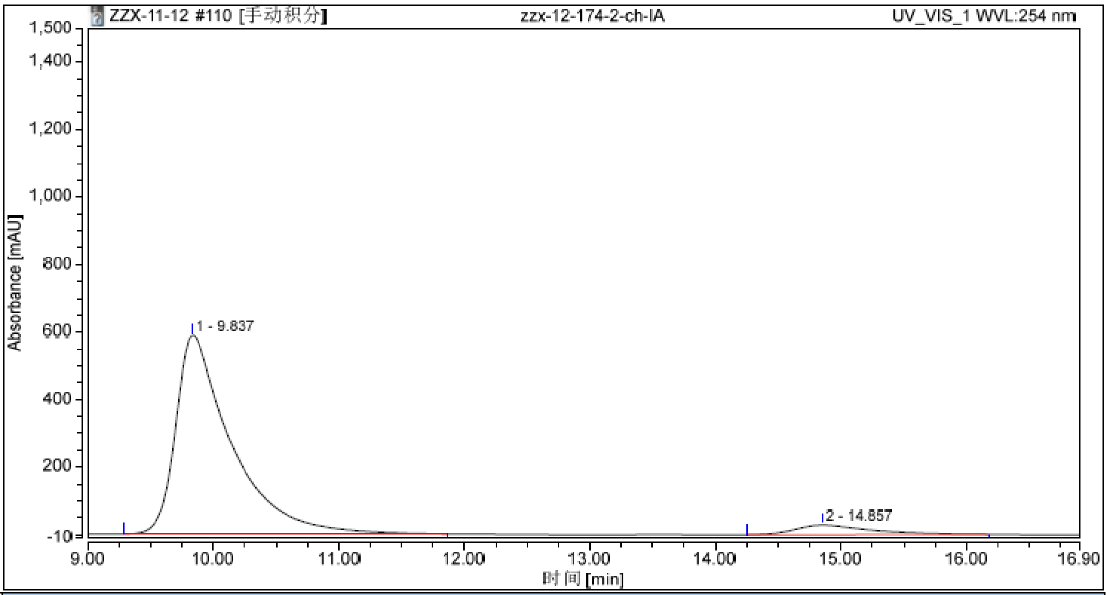

Integration Results

No.	Peak Name	Retention Time<br>min	Area<br>mAU*min	Height<br>mAU	Relative Area<br>%	Relative Height<br>%	Amount<br>n.a.
1		9.837	302.332	588.668	94.07	95.48	n.a.
2		14.857	19.065	27.854	5.93	4.52	n.a.
**Total:**			**321.397**	**616.522**	**100.00**	**100.00**	

Compound **2k:** HPLC (IA, *n*-hexane/2-propanol = 50/50, v = 1.0 mL/min, λ = 254 nm)

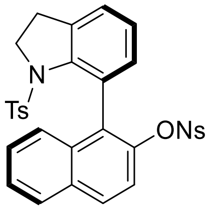

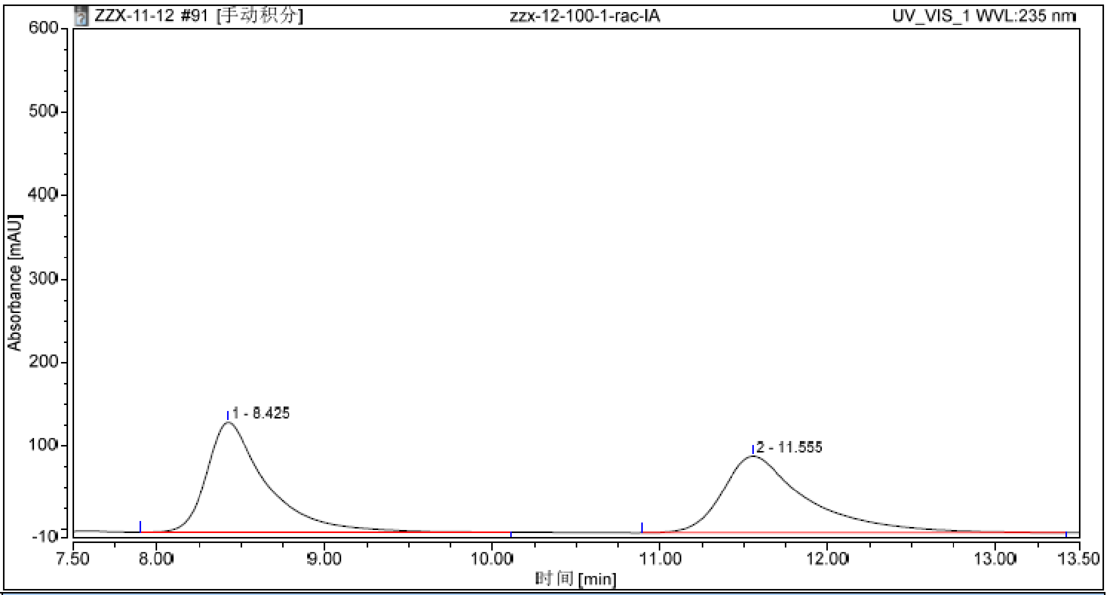

Integration Results

No.	Peak Name	Retention Time min	Area mAU*min	Height mAU	Relative Area %	Relative Height %	Amount n.a.
1		8.425	53.980	131.267	50.40	59.05	n.a.
2		11.555	53.118	91.043	49.60	40.95	n.a.
**Total:**			**107.098**	**222.310**	**100.00**	**100.00**	

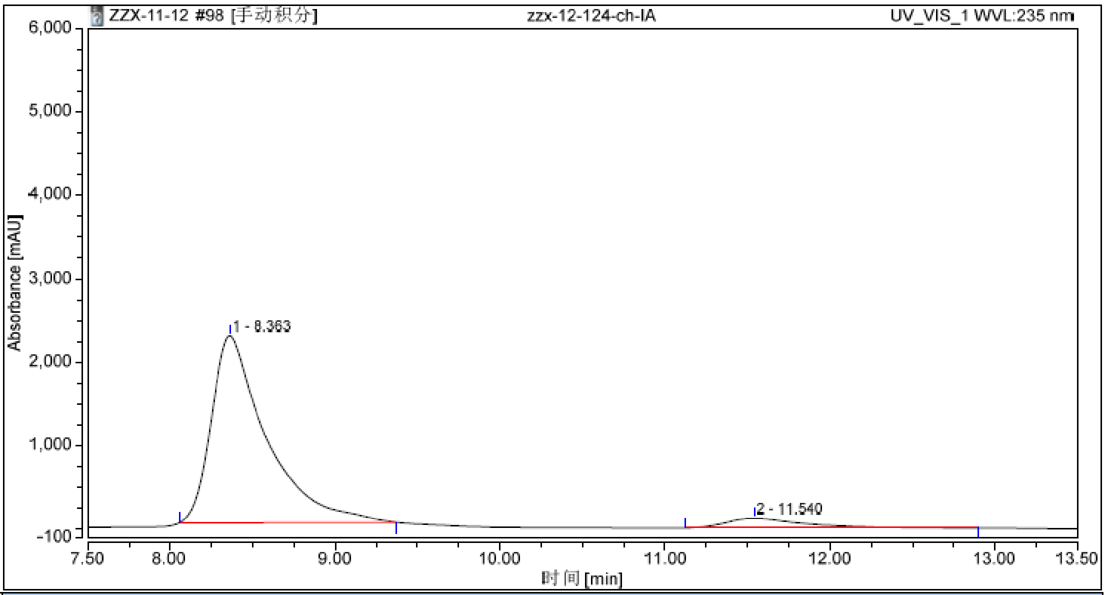

Integration Results

No.	Peak Name	Retention Time min	Area mAU*min	Height mAU	Relative Area %	Relative Height %	Amount n.a.
1		8.363	863.908	2238.703	93.13	95.03	n.a.
2		11.540	63.735	117.023	6.87	4.97	n.a.
**Total:**			**927.643**	**2355.725**	**100.00**	**100.00**	

Compound **2l:** HPLC (IA, *n*-hexane/2-propanol = 50/50, v = 1.0 mL/min, λ = 254 nm)

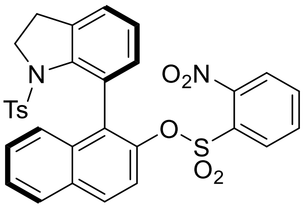

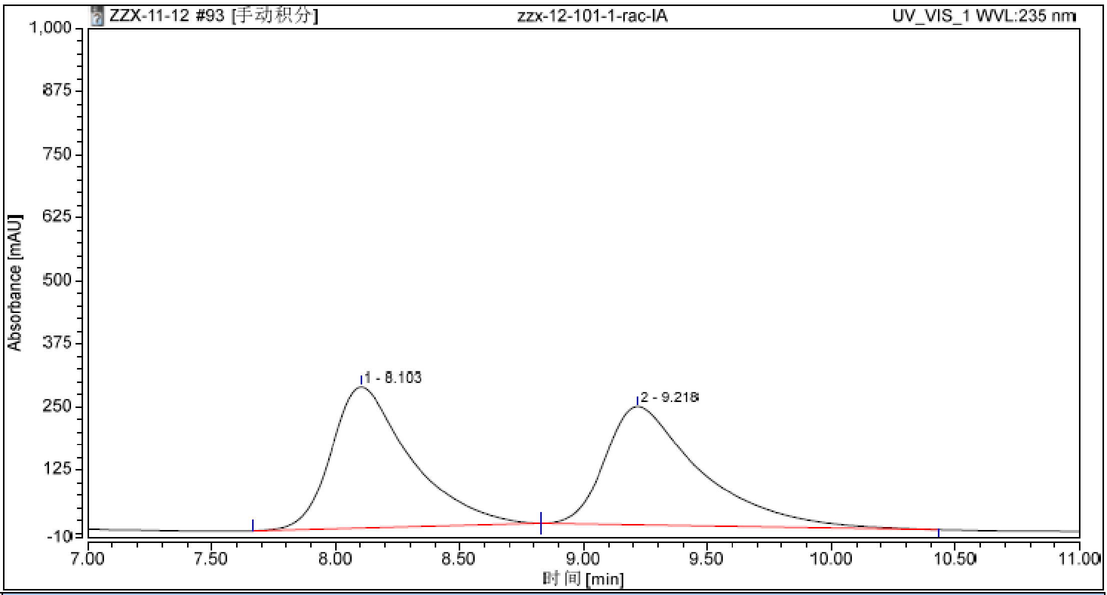

Integration Results

No.	Peak Name	Retention Time min	Area mAU*min	Height mAU	Relative Area %	Relative Height %	Amount n.a.
1		8.103	103.388	279.272	49.97	54.41	n.a.
2		9.218	103.515	233.954	50.03	45.59	n.a.
**Total:**			**206.903**	**513.226**	**100.00**	**100.00**	

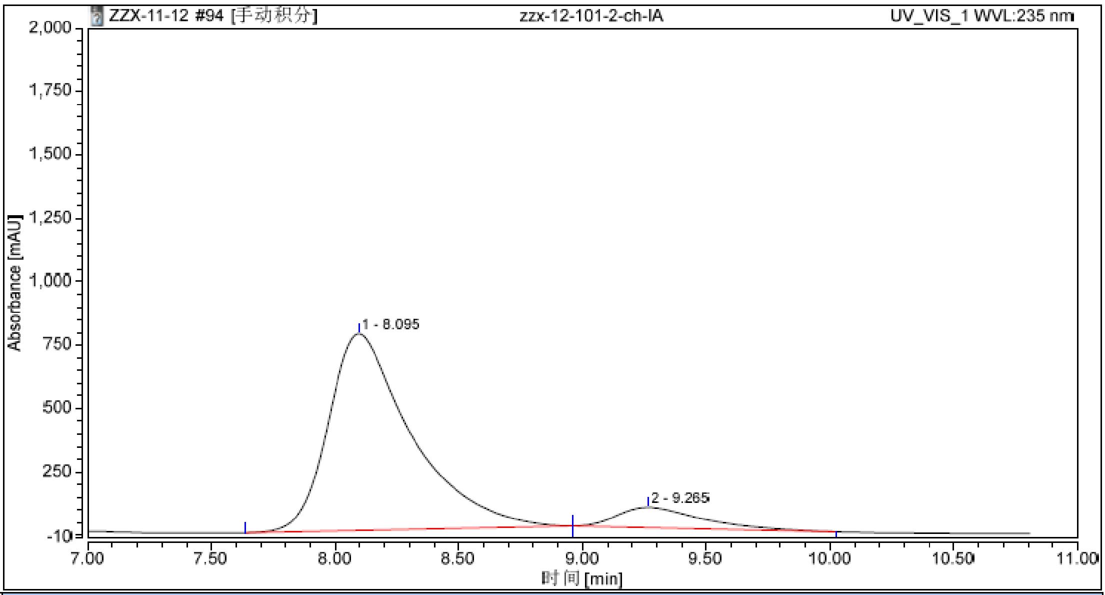

Integration Results

No.	Peak Name	Retention Time min	Area mAU*min	Height mAU	Relative Area %	Relative Height %	Amount n.a.
1		8.095	302.514	776.237	90.55	90.68	n.a.
2		9.265	31.563	79.807	9.45	9.32	n.a.
**Total:**			**334.077**	**856.044**	**100.00**	**100.00**	

Compound **2m:** HPLC (IA, *n*-hexane/2-propanol = 70/30, v = 1.0 mL/min, λ = 254 nm)

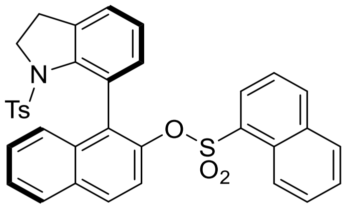

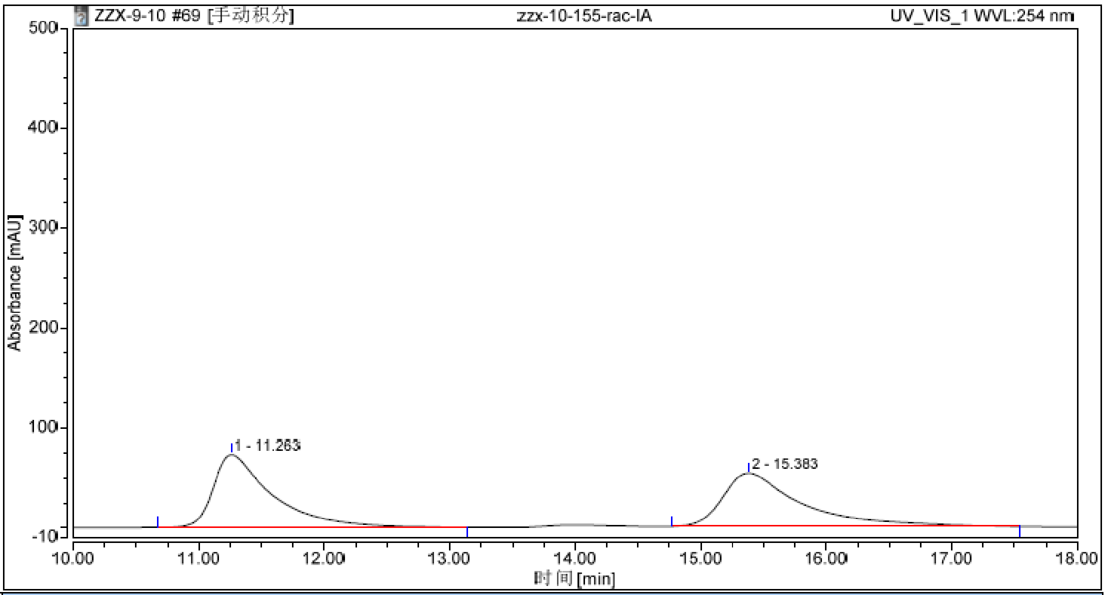

Integration Results

No.	Peak Name	Retention Time min	Area mAU*min	Height mAU	Relative Area %	Relative Height %	Amount n.a.
1		11.263	39.434	72.711	50.55	57.71	n.a.
2		15.383	38.583	53.286	49.45	42.29	n.a.
**Total:**			**78.017**	**125.996**	**100.00**	**100.00**	

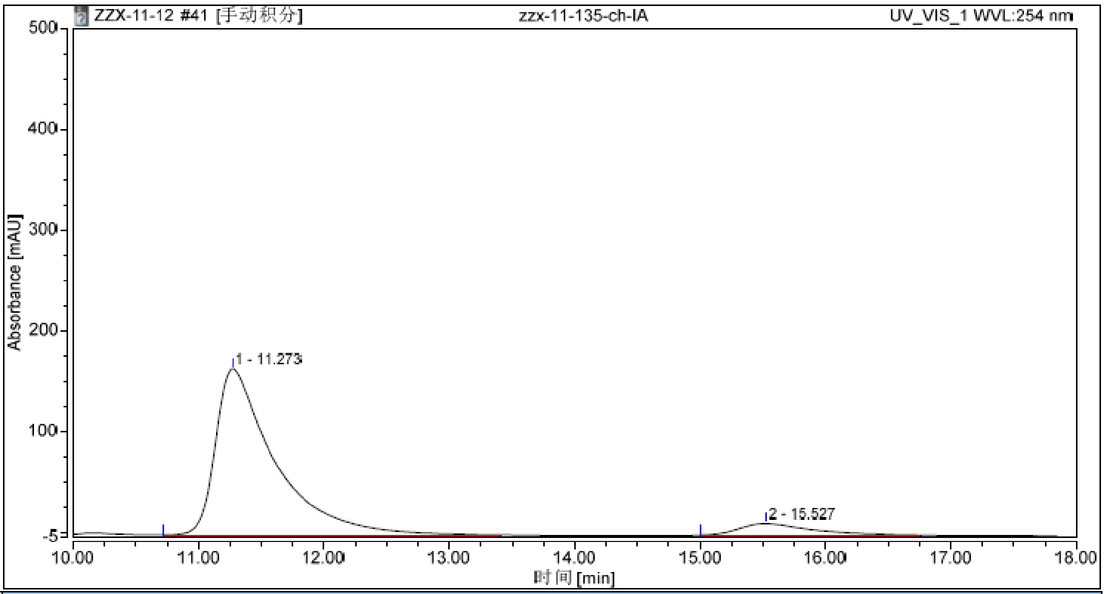

Integration Results

No.	Peak Name	Retention Time min	Area mAU*min	Height mAU	Relative Area %	Relative Height %	Amount n.a.
1		11.273	89.899	164.881	92.28	93.56	n.a.
2		15.527	7.516	11.345	7.72	6.44	n.a.
**Total:**			**97.415**	**176.226**	**100.00**	**100.00**	

Compound **2n:** HPLC (IA, *n*-hexane/2-propanol = 80/20, v = 1.0 mL/min, λ = 254 nm)

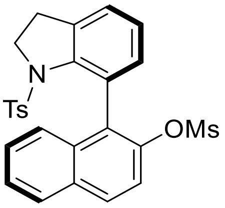

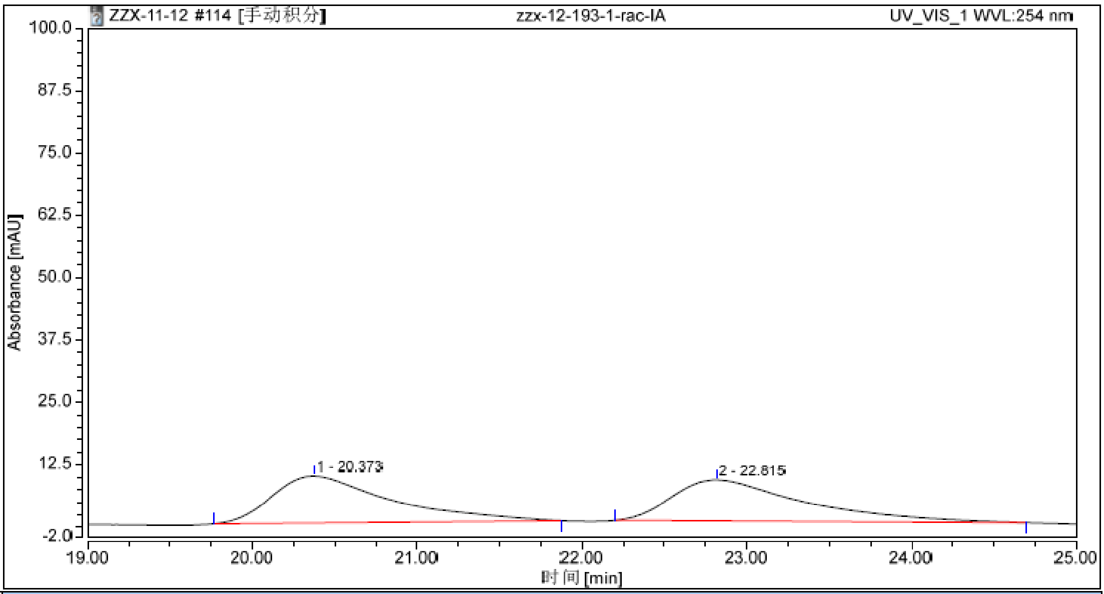

Integration Results

No.	Peak Name	Retention Time<br>min	Area<br>mAU*min	Height<br>mAU	Relative Area<br>%	Relative Height<br>%	Amount<br>n.a.
1		20.373	7.736	9.316	50.69	53.27	n.a.
2		22.815	7.525	8.173	49.31	46.73	n.a.
Total:			15.261	17.489	100.00	100.00	

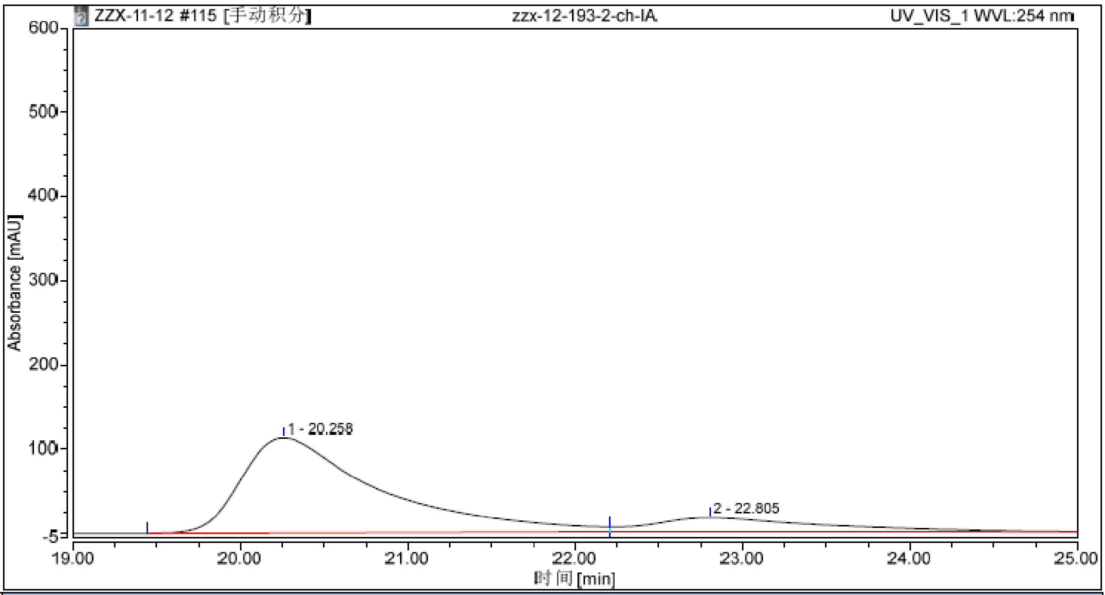

Integration Results

No.	Peak Name	Retention Time<br>min	Area<br>mAU*min	Height<br>mAU	Relative Area<br>%	Relative Height<br>%	Amount<br>n.a.
1		20.258	107.744	112.660	84.17	86.97	n.a.
2		22.805	20.259	16.874	15.83	13.03	n.a.
Total:			128.003	129.534	100.00	100.00	

Compound **2o:** HPLC (ODH, *n*-hexane/2-propanol = 70/30, v = 1.0 mL/min, λ = 254 nm)

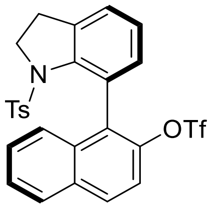

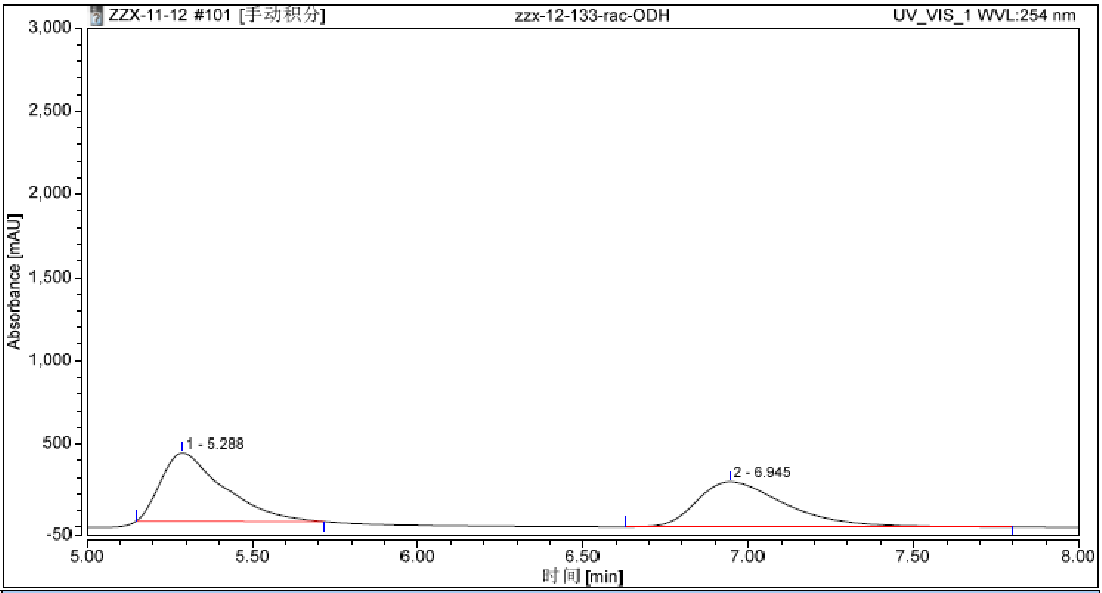

Integration Results

No.	Peak Name	Retention Time min	Area mAU*min	Height mAU	Relative Area %	Relative Height %	Amount n.a.
1		5.288	89.850	410.000	51.32	60.05	n.a.
2		6.945	85.214	272.723	48.68	39.95	n.a.
**Total:**			**175.063**	**682.724**	**100.00**	**100.00**	

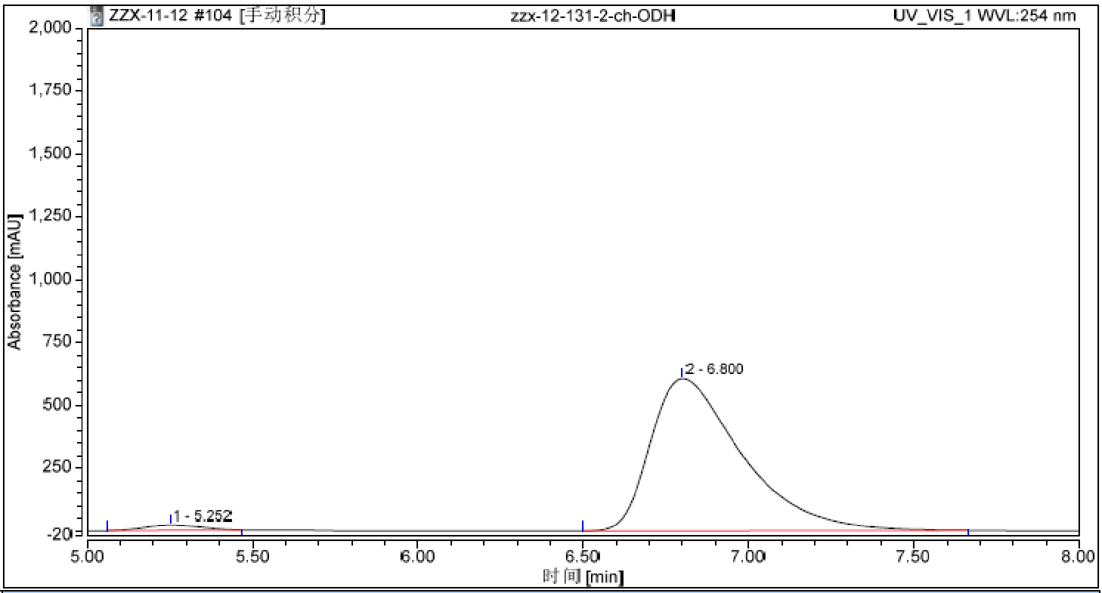

Integration Results

No.	Peak Name	Retention Time min	Area mAU*min	Height mAU	Relative Area %	Relative Height %	Amount n.a.
1		5.252	4.207	21.151	2.11	3.37	n.a.
2		6.800	194.736	606.247	97.89	96.63	n.a.
**Total:**			**198.943**	**627.399**	**100.00**	**100.00**	

Compound **2p:** HPLC (ADH, *n*-hexane/2-propanol = 70/30, v = 1.0 mL/min, λ = 254 nm)

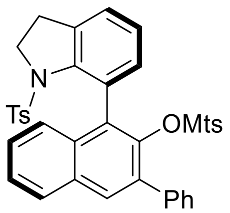

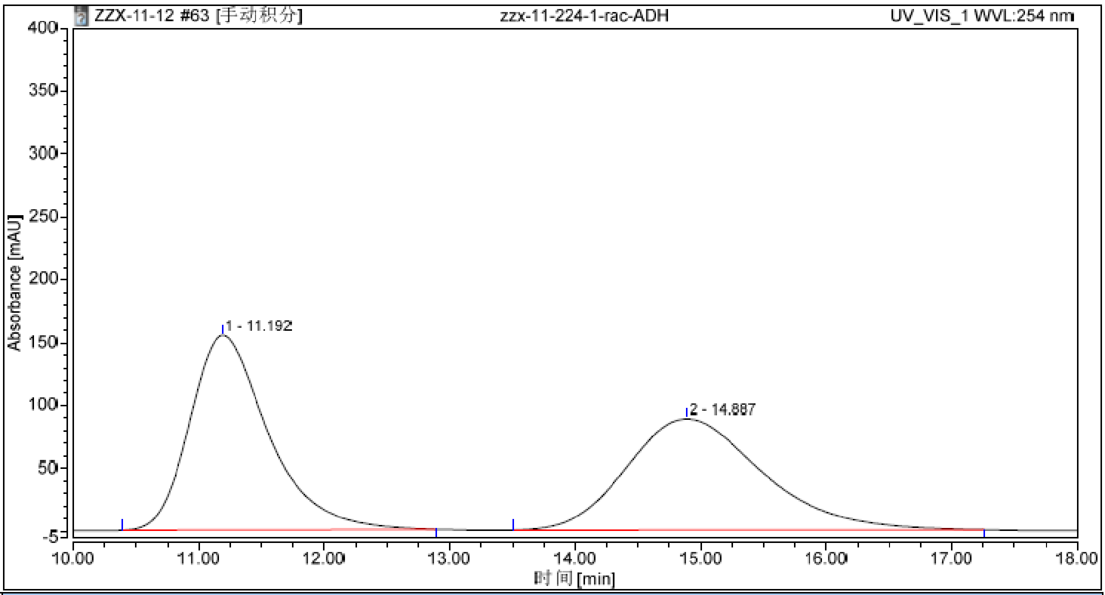

Integration Results

No.	Peak Name	Retention Time min	Area mAU*min	Height mAU	Relative Area %	Relative Height %	Amount n.a.
1		11.192	112.231	154.972	50.37	63.72	n.a.
2		14.887	110.581	88.241	49.63	36.28	n.a.
**Total:**			**222.813**	**243.213**	**100.00**	**100.00**	

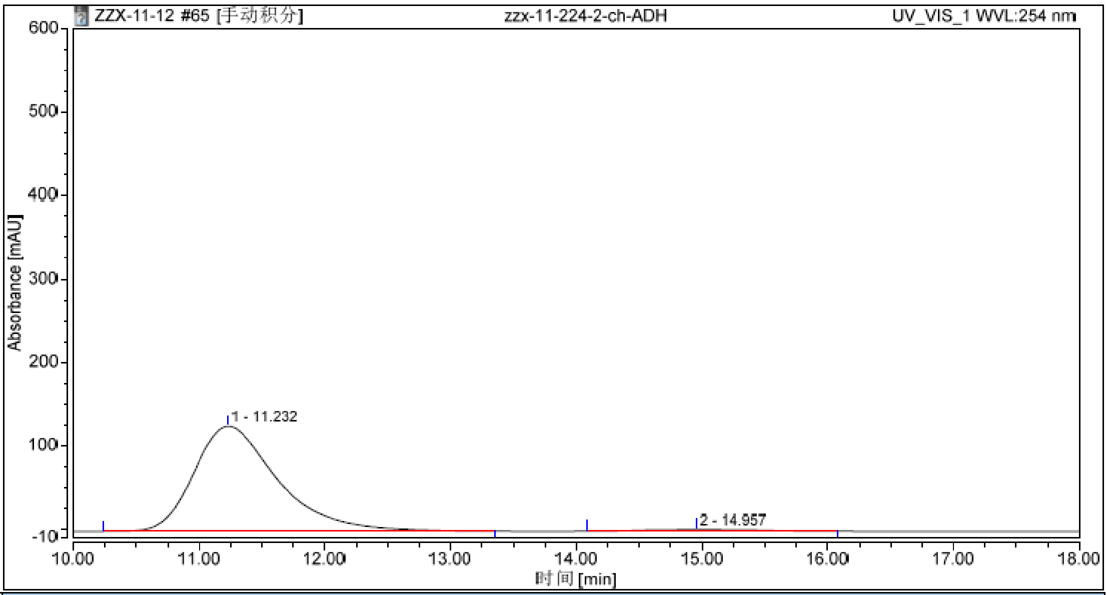

Integration Results

No.	Peak Name	Retention Time min	Area mAU*min	Height mAU	Relative Area %	Relative Height %	Amount n.a.
1		11.232	96.651	125.603	98.00	98.53	n.a.
2		14.957	1.971	1.868	2.00	1.47	n.a.
**Total:**			**98.622**	**127.471**	**100.00**	**100.00**	

Compound **2q:** HPLC (IA, *n*-hexane/2-propanol = 70/30, v = 1.0 mL/min, λ = 254 nm)

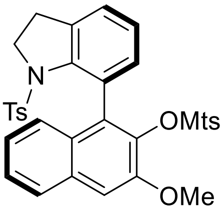

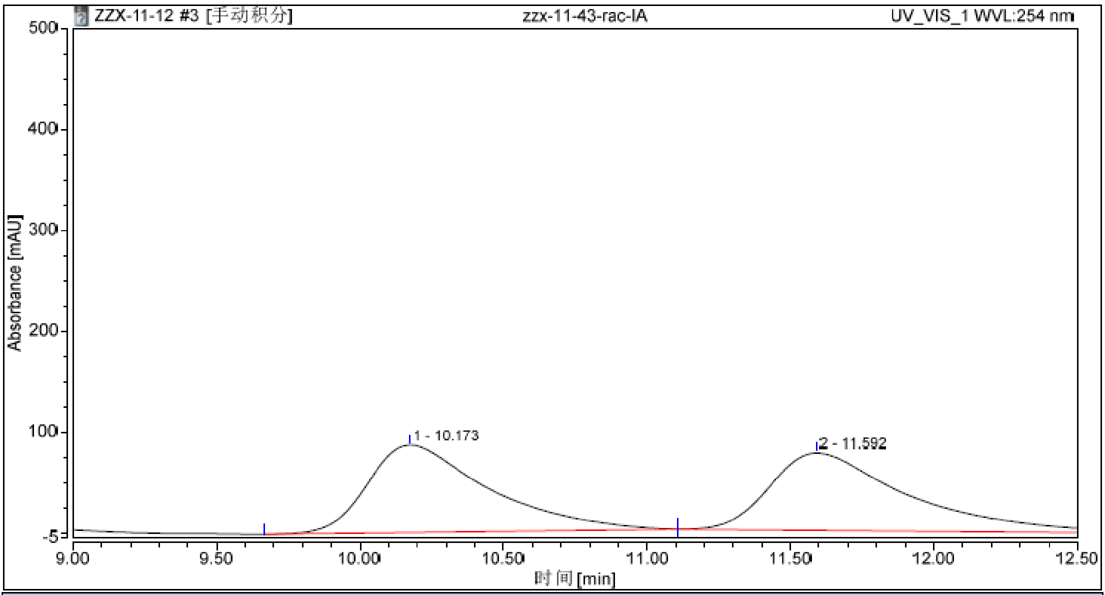

Integration Results							
No.	Peak Name	Retention Time min	Area mAU*min	Height mAU	Relative Area %	Relative Height %	Amount n.a.
1		10.173	42.256	86.755	49.71	53.12	n.a.
2		11.592	42.740	76.575	50.29	46.88	n.a.
**Total:**			**84.996**	**163.330**	**100.00**	**100.00**	

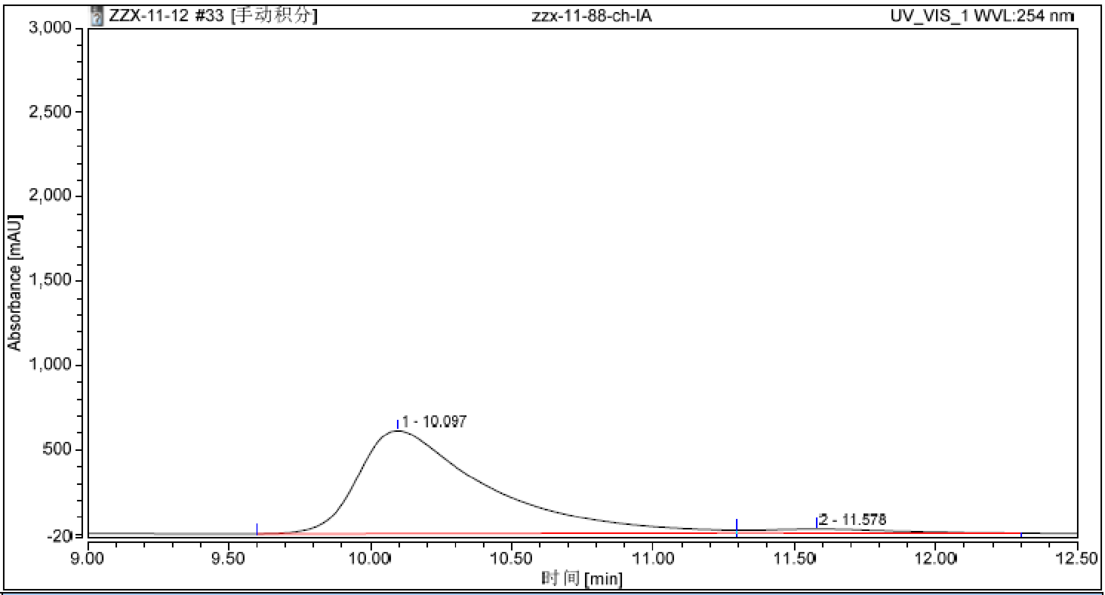

Integration Results							
No.	Peak Name	Retention Time min	Area mAU*min	Height mAU	Relative Area %	Relative Height %	Amount n.a.
1		10.097	321.863	610.472	96.27	96.33	n.a.
2		11.578	12.459	23.225	3.73	3.67	n.a.
**Total:**			**334.322**	**633.698**	**100.00**	**100.00**	

Compound **2r:** HPLC (IA, *n*-hexane/2-propanol = 85/15, v = 1.0 mL/min, λ = 254 nm)

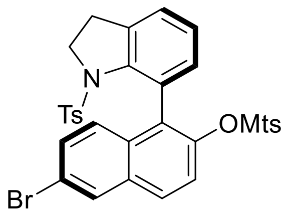

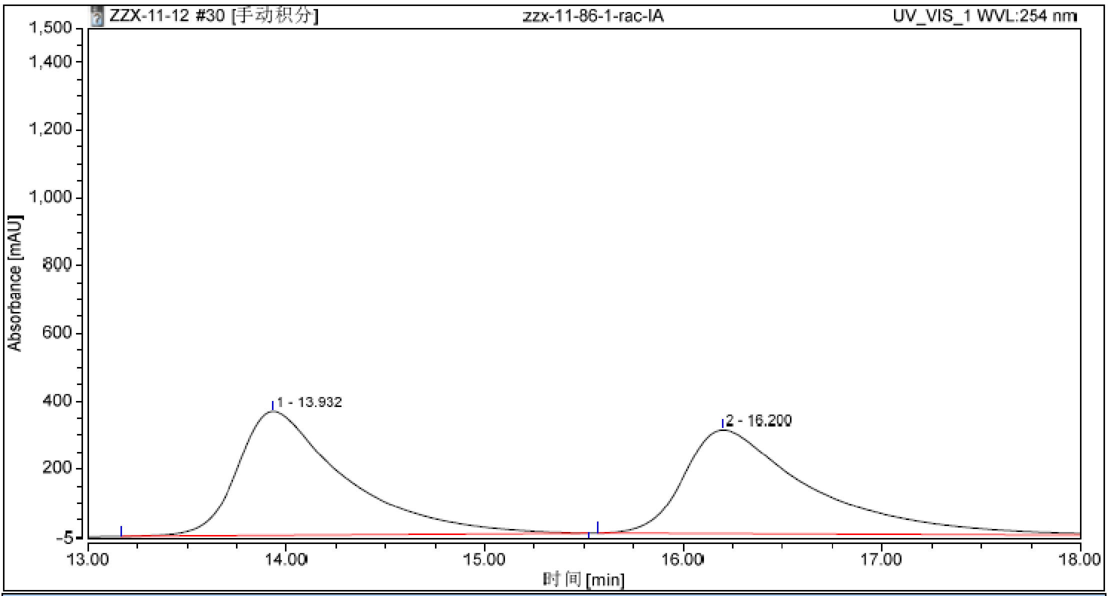

Integration Results

No.	Peak Name	Retention Time min	Area mAU*min	Height mAU	Relative Area %	Relative Height %	Amount n.a.
1		13.932	228.839	365.134	50.29	54.46	n.a.
2		16.200	226.176	305.387	49.71	45.54	n.a.
Total:			455.015	670.522	100.00	100.00	

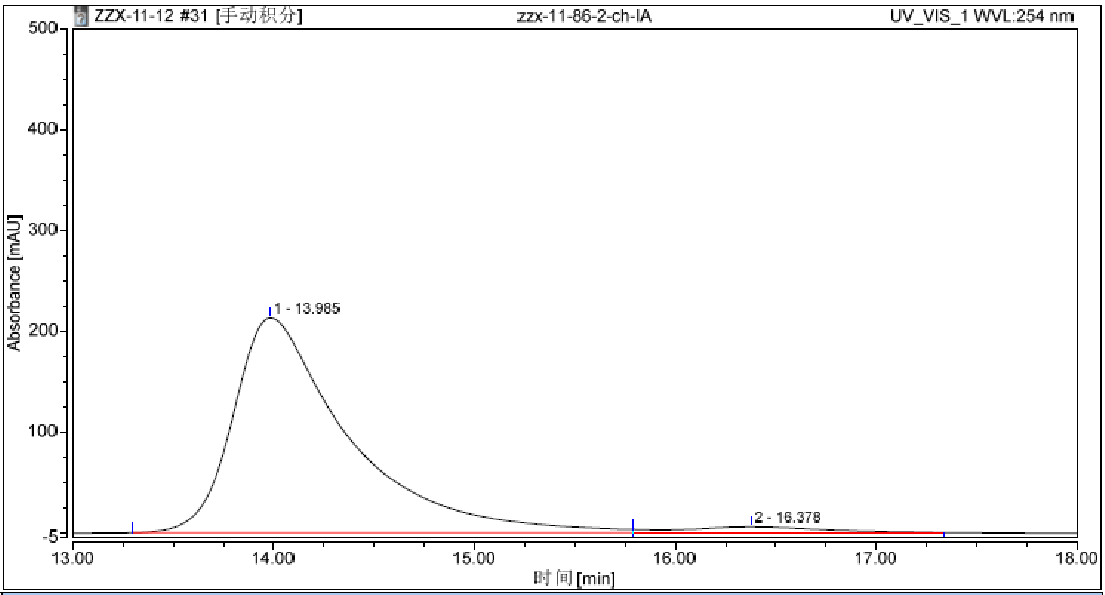

Integration Results

No.	Peak Name	Retention Time min	Area mAU*min	Height mAU	Relative Area %	Relative Height %	Amount n.a.
1		13.985	141.851	213.069	96.90	97.36	n.a.
2		16.378	4.540	5.774	3.10	2.64	n.a.
Total:			146.391	218.843	100.00	100.00	

Compound **2s:** HPLC (IA, *n*-hexane/2-propanol = 70/30, v = 1.0 mL/min, λ = 254 nm)

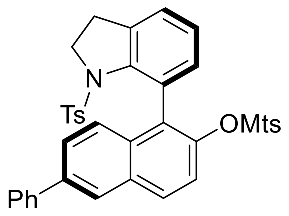

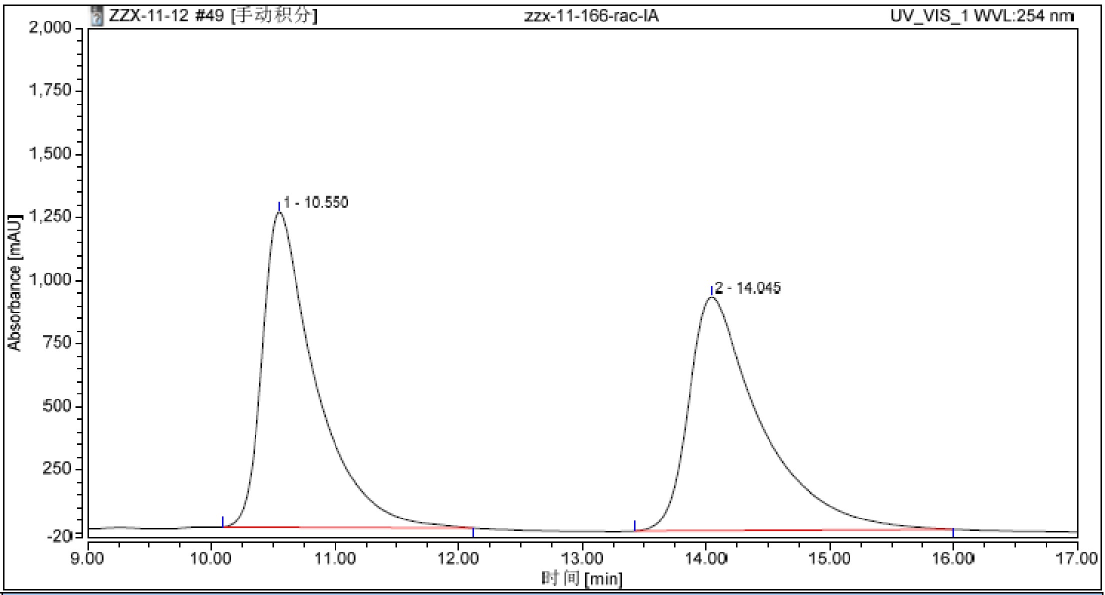

Integration Results

No.	Peak Name	Retention Time min	Area mAU*min	Height mAU	Relative Area %	Relative Height %	Amount n.a.
1		10.550	623.340	1251.600	49.93	57.46	n.a.
2		14.045	624.981	926.676	50.07	42.54	n.a.
**Total:**			**1248.321**	**2178.276**	**100.00**	**100.00**	

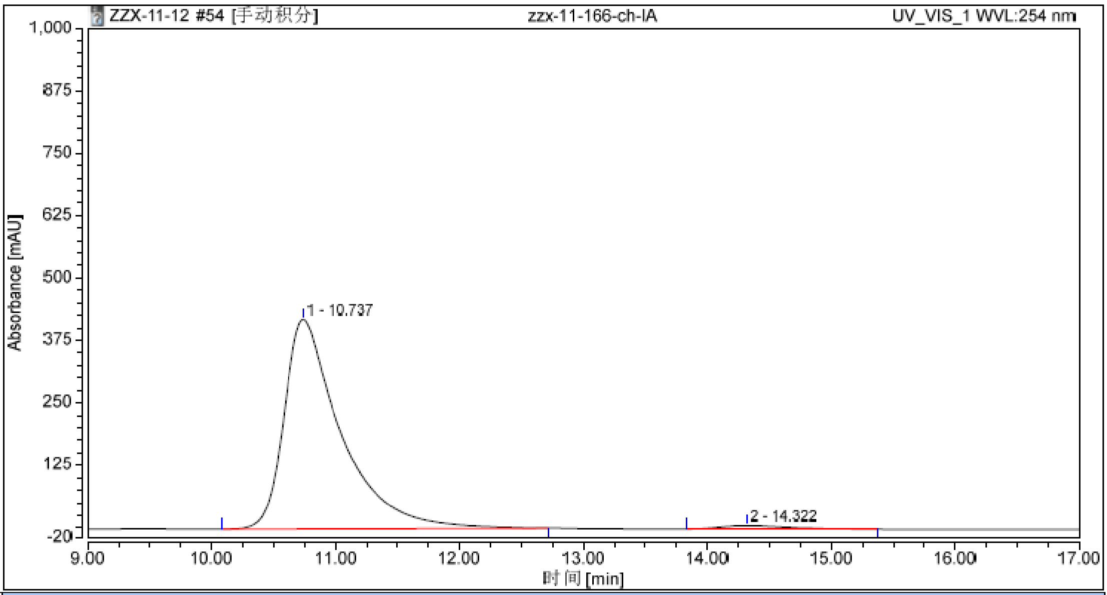

Integration Results

No.	Peak Name	Retention Time min	Area mAU*min	Height mAU	Relative Area %	Relative Height %	Amount n.a.
1		10.737	223.420	419.832	97.99	98.27	n.a.
2		14.322	4.574	7.378	2.01	1.73	n.a.
**Total:**			**227.994**	**427.211**	**100.00**	**100.00**	

Compound **2t:** HPLC (IA, *n*-hexane/2-propanol = 70/30, v = 1.0 mL/min, λ = 254 nm)

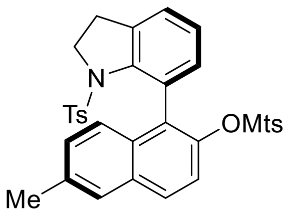

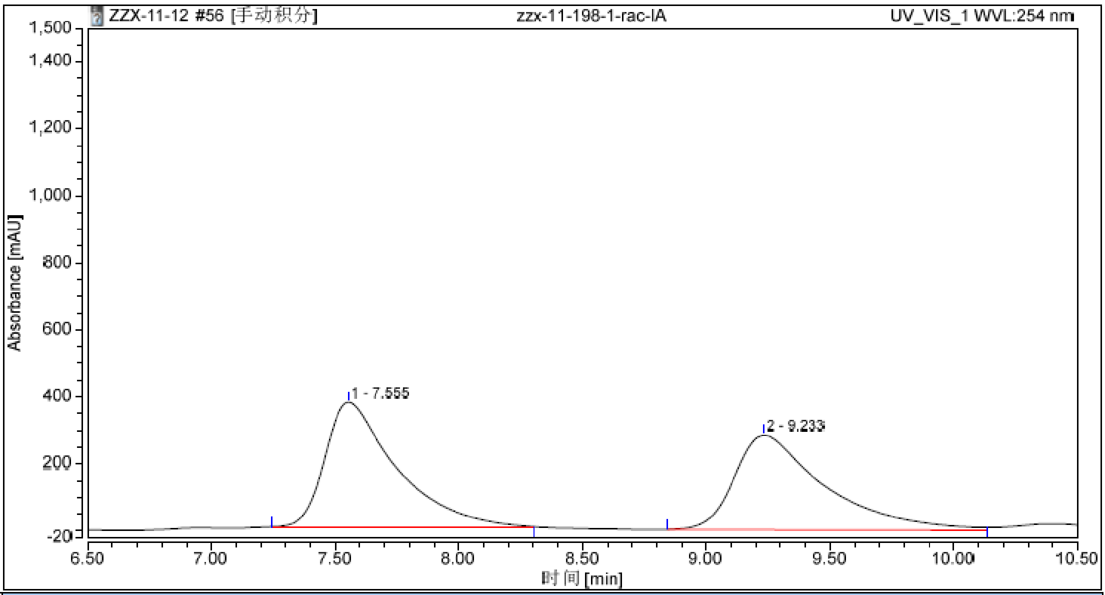

Integration Results

No.	Peak Name	Retention Time min	Area mAU*min	Height mAU	Relative Area %	Relative Height %	Amount n.a.
1		7.555	123.428	371.442	50.95	56.88	n.a.
2		9.233	118.845	281.585	49.05	43.12	n.a.
**Total:**			**242.273**	**653.027**	**100.00**	**100.00**	

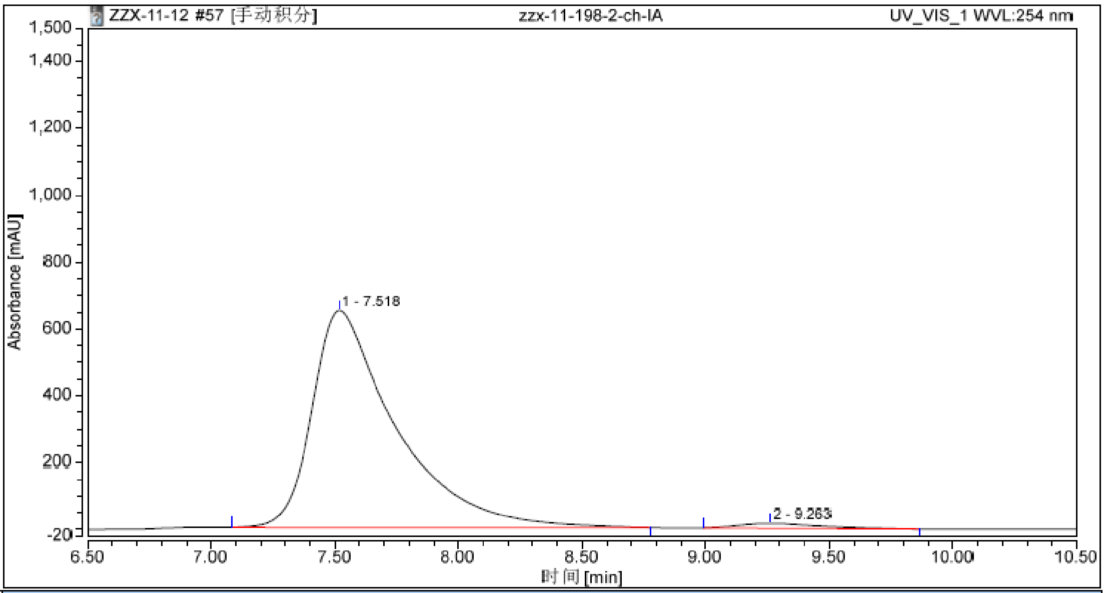

Integration Results

No.	Peak Name	Retention Time min	Area mAU*min	Height mAU	Relative Area %	Relative Height %	Amount n.a.
1		7.518	255.496	649.125	97.91	97.80	n.a.
2		9.263	5.442	14.625	2.09	2.20	n.a.
**Total:**			**260.938**	**663.749**	**100.00**	**100.00**	

Compound **2u:** HPLC (ODH, *n*-hexane/2-propanol = 70/30, v = 1.0 mL/min, λ = 254 nm)

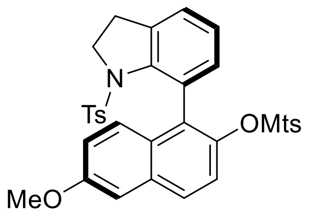

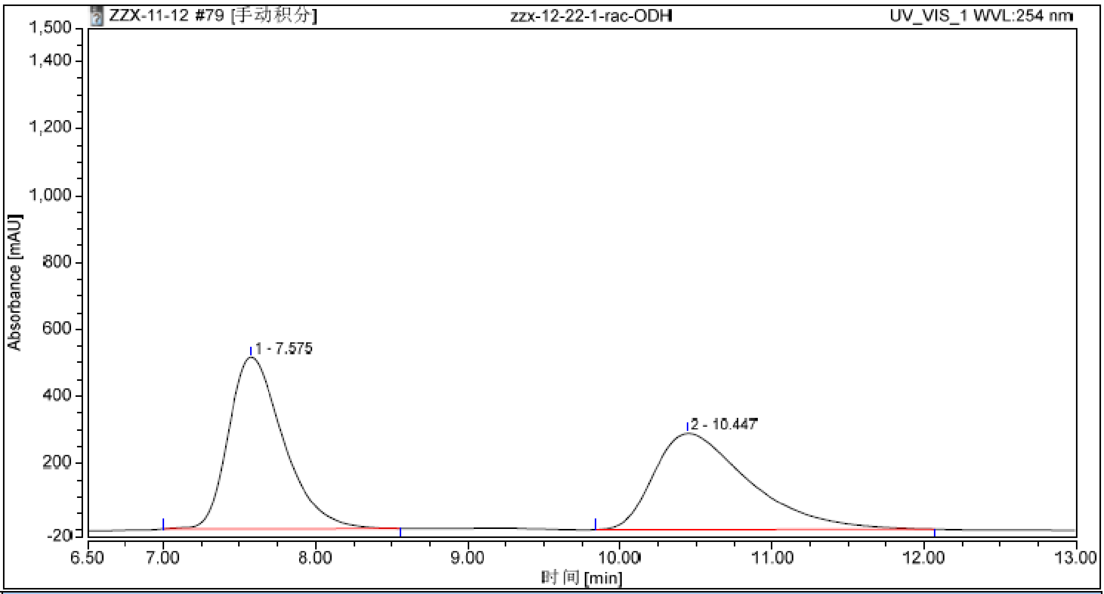

Integration Results

No.	Peak Name	Retention Time min	Area mAU*min	Height mAU	Relative Area %	Relative Height %	Amount n.a.
1		7.575	214.103	511.928	50.71	64.14	n.a.
2		10.447	208.082	286.260	49.29	35.86	n.a.
Total:			422.185	798.187	100.00	100.00	

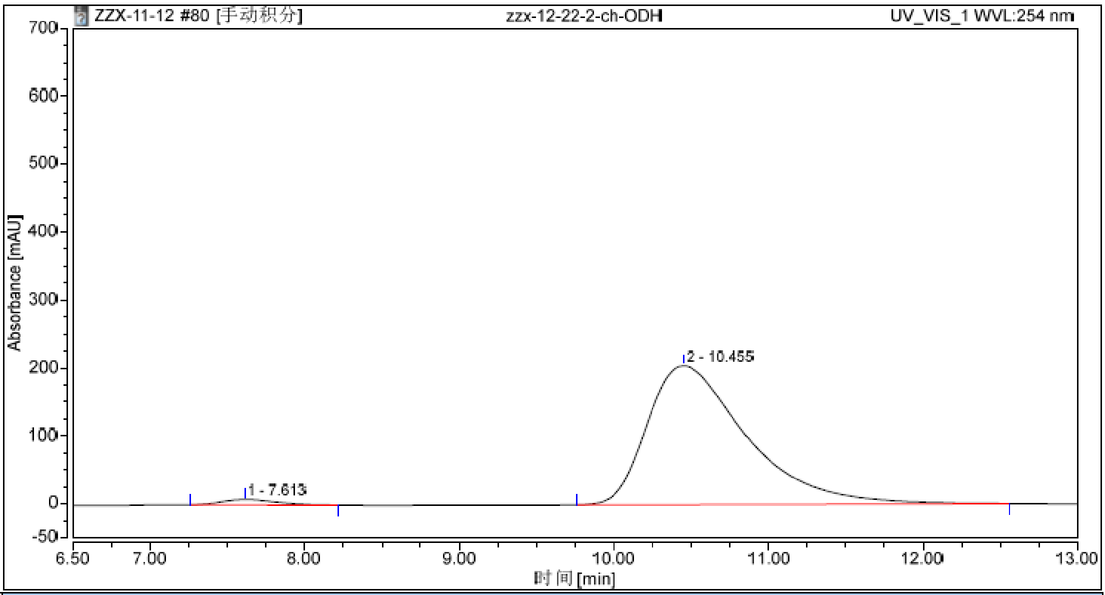

Integration Results

No.	Peak Name	Retention Time min	Area mAU*min	Height mAU	Relative Area %	Relative Height %	Amount n.a.
1		7.613	3.155	7.910	2.04	3.73	n.a.
2		10.455	151.692	203.956	97.96	96.27	n.a.
Total:			154.847	211.866	100.00	100.00	

Compound **2v:** HPLC (ADH, *n*-hexane/2-propanol = 70/30, v = 1.0 mL/min, λ = 254 nm)

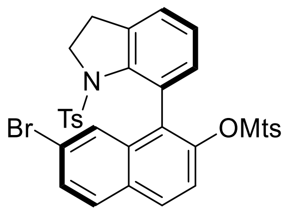

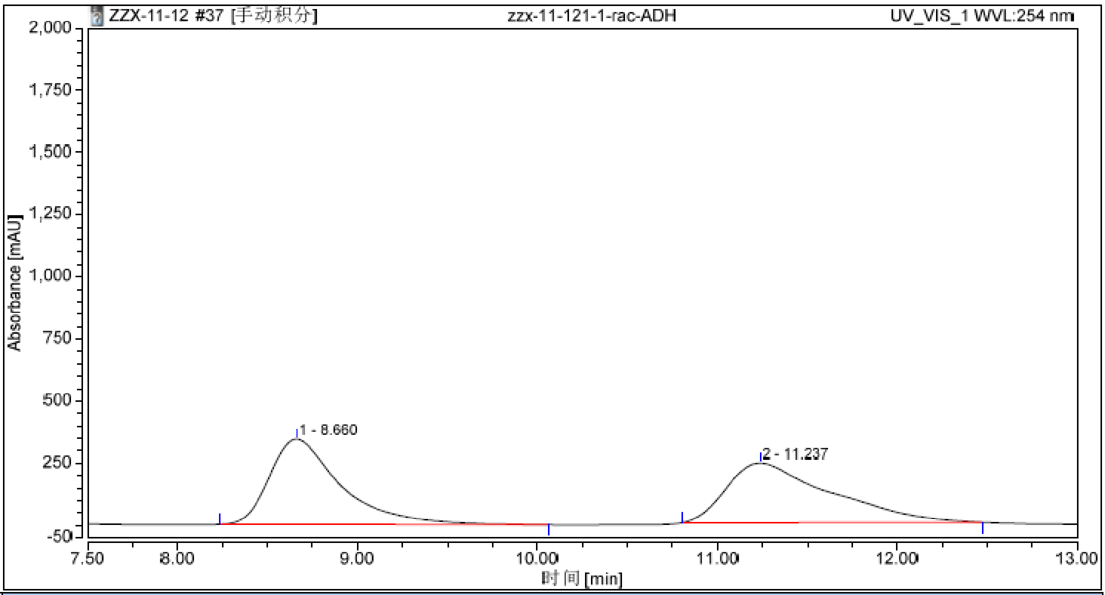

Integration Results

No.	Peak Name	Retention Time min	Area mAU*min	Height mAU	Relative Area %	Relative Height %	Amount n.a.
1		8.660	157.016	343.273	49.63	58.92	n.a.
2		11.237	159.386	239.294	50.37	41.08	n.a.
Total:			316.402	582.567	100.00	100.00	

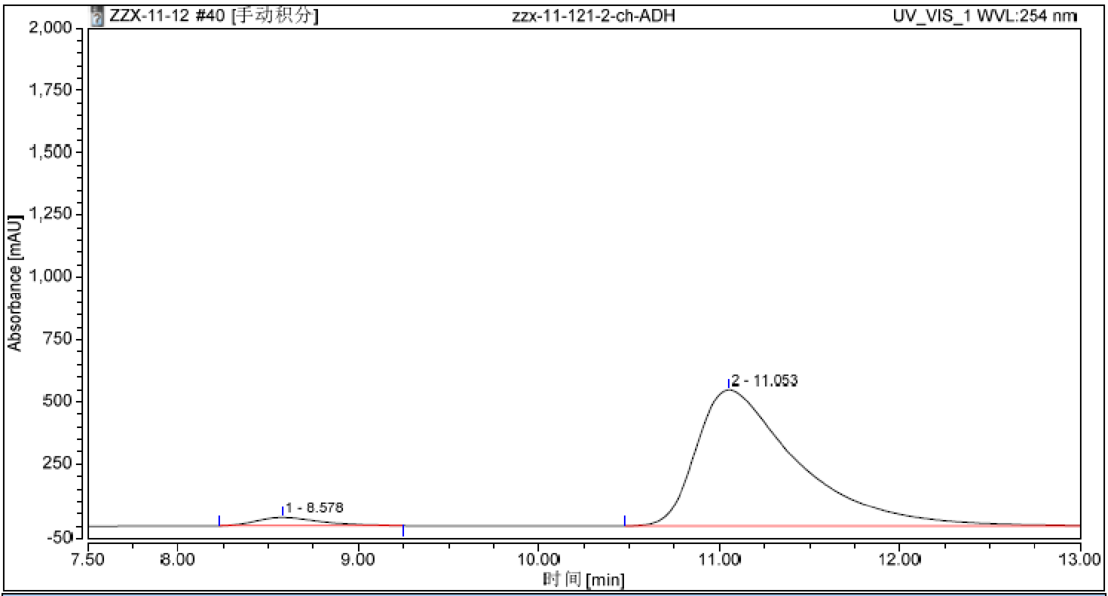

Integration Results

No.	Peak Name	Retention Time min	Area mAU*min	Height mAU	Relative Area %	Relative Height %	Amount n.a.
1		8.578	13.500	33.351	3.65	5.75	n.a.
2		11.053	356.243	547.151	96.35	94.25	n.a.
Total:			369.743	580.502	100.00	100.00	

Compound **2w:** HPLC (ADH, *n*-hexane/2-propanol = 85/15, v = 1.0 mL/min, λ = 254 nm)

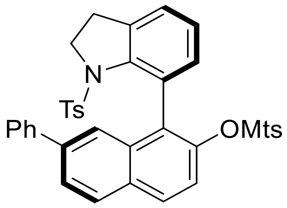

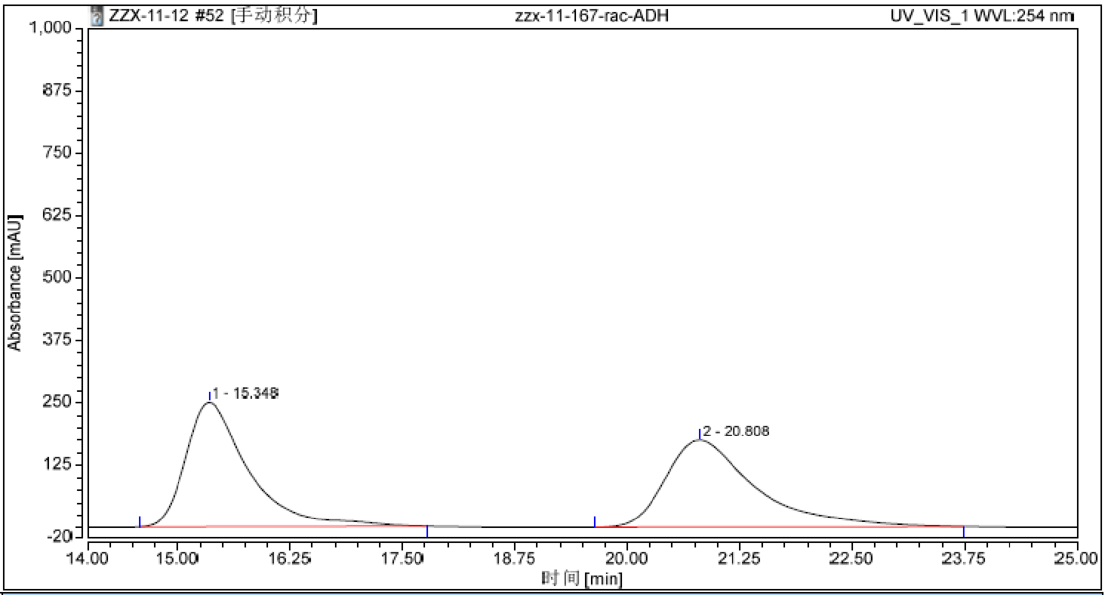

Integration Results

No.	Peak Name	Retention Time<br>min	Area<br>mAU*min	Height<br>mAU	Relative Area<br>%	Relative Height<br>%	Amount<br>n.a.
1		15.348	200.295	248.373	49.91	58.82	n.a.
2		20.808	201.038	173.859	50.09	41.18	n.a.
**Total:**			**401.333**	**422.232**	**100.00**	**100.00**	

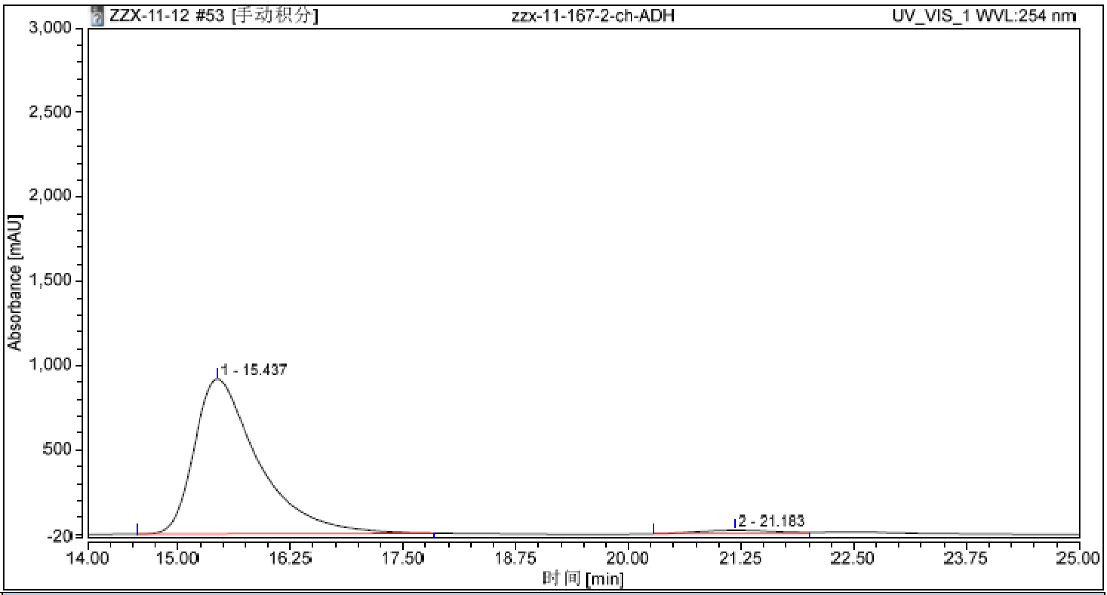

Integration Results

No.	Peak Name	Retention Time<br>min	Area<br>mAU*min	Height<br>mAU	Relative Area<br>%	Relative Height<br>%	Amount<br>n.a.
1		15.437	753.125	916.333	97.37	97.84	n.a.
2		21.183	20.329	20.258	2.63	2.16	n.a.
**Total:**			**773.454**	**936.592**	**100.00**	**100.00**	

Compound **2x:** HPLC (ODH, *n*-hexane/2-propanol = 85/15, v = 1.0 mL/min, λ = 254 nm)

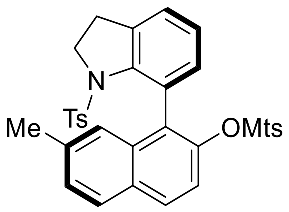

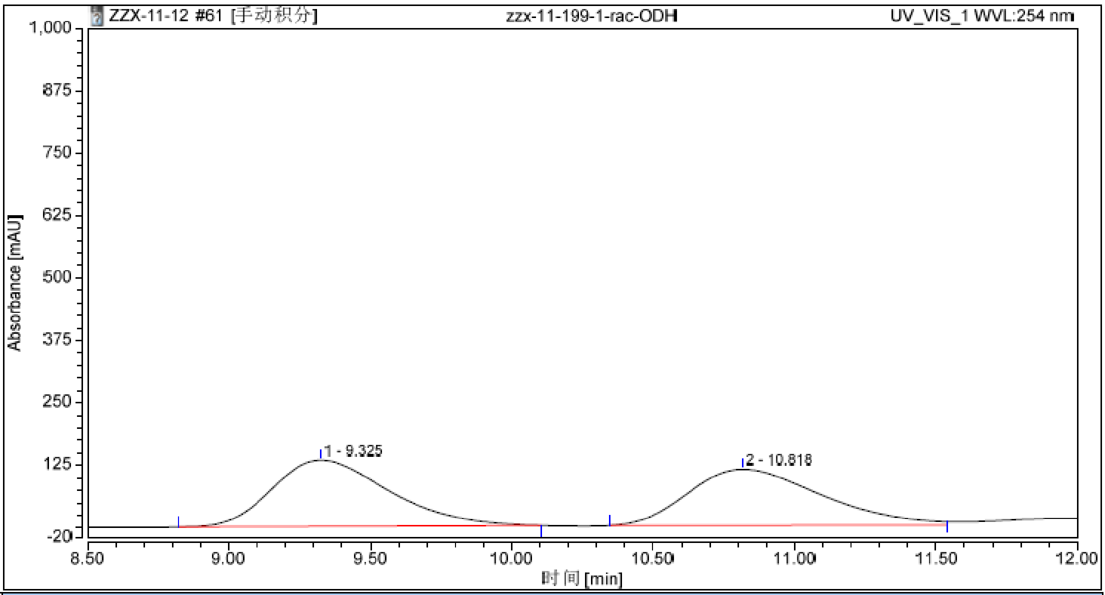

Integration Results

No.	Peak Name	Retention Time<br>min	Area<br>mAU*min	Height<br>mAU	Relative Area<br>%	Relative Height<br>%	Amount<br>n.a.
1		9.325	63.219	132.386	50.67	54.13	n.a.
2		10.818	61.558	112.163	49.33	45.87	n.a.
**Total:**			**124.777**	**244.548**	**100.00**	**100.00**	

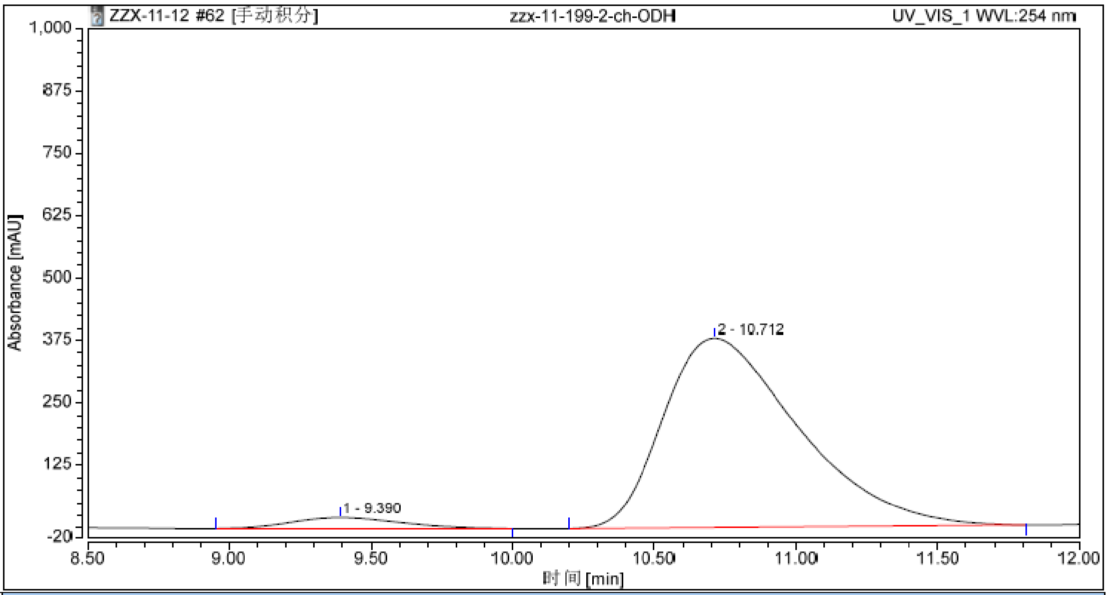

Integration Results

No.	Peak Name	Retention Time<br>min	Area<br>mAU*min	Height<br>mAU	Relative Area<br>%	Relative Height<br>%	Amount<br>n.a.
1		9.390	9.808	21.847	4.47	5.46	n.a.
2		10.712	209.579	378.454	95.53	94.54	n.a.
**Total:**			**219.386**	**400.300**	**100.00**	**100.00**	

Compound **2y:** HPLC (ODH, *n*-hexane/2-propanol = 85/15, v = 1.0 mL/min, λ = 254 nm)

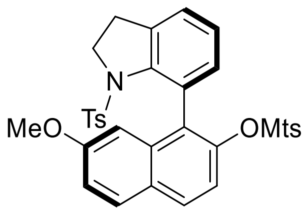

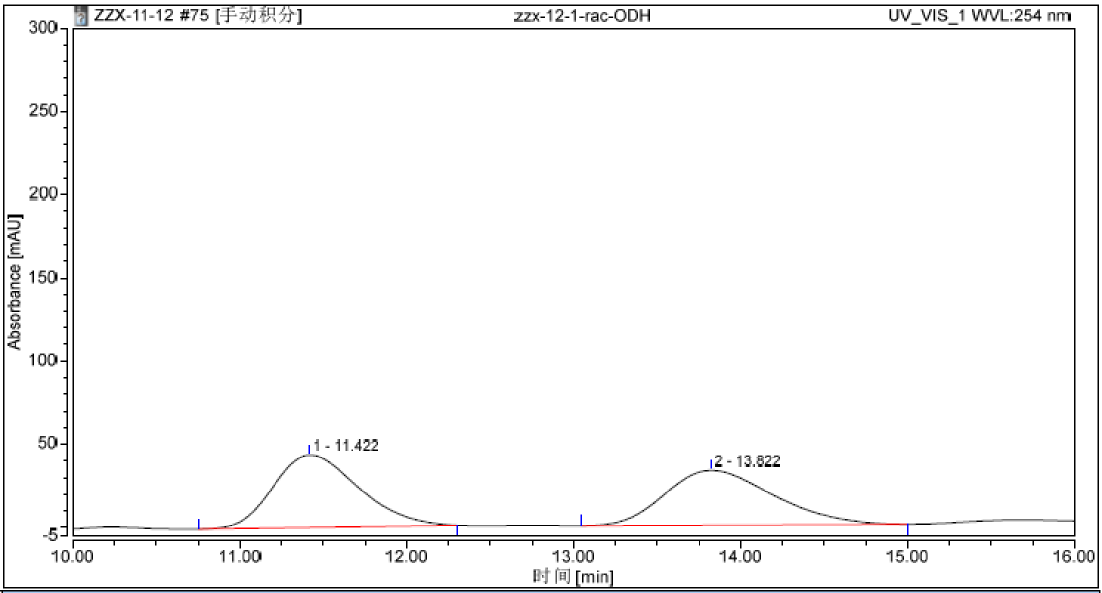

Integration Results							
No.	Peak Name	Retention Time min	Area mAU*min	Height mAU	Relative Area %	Relative Height %	Amount n.a.
1		11.422	25.789	43.522	50.98	56.96	n.a.
2		13.822	24.795	32.888	49.02	43.04	n.a.
Total:			**50.584**	**76.410**	**100.00**	**100.00**	

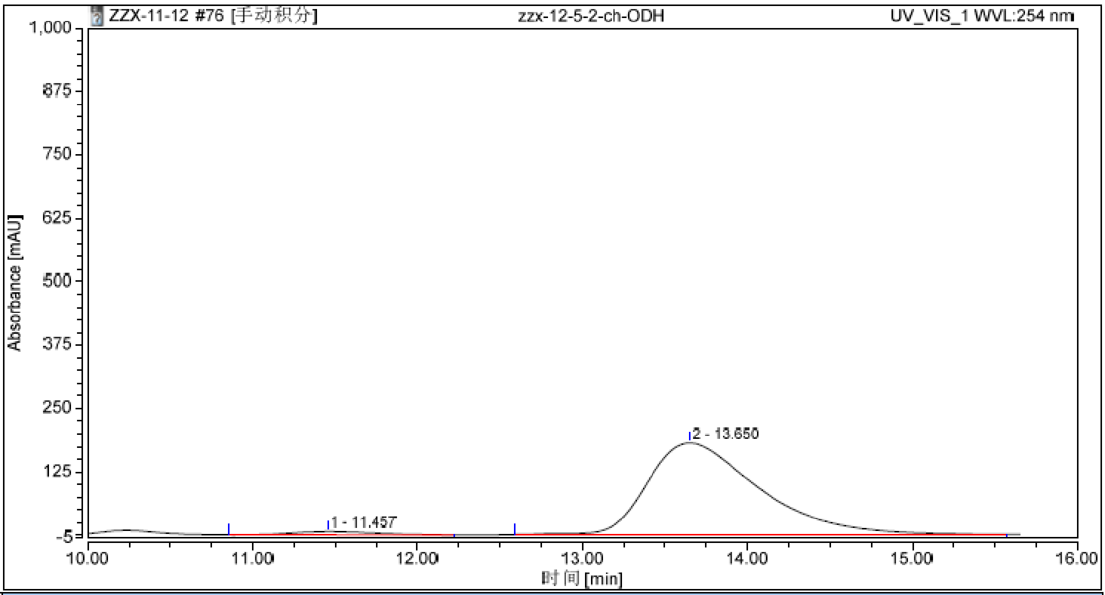

Integration Results							
No.	Peak Name	Retention Time min	Area mAU*min	Height mAU	Relative Area %	Relative Height %	Amount n.a.
1		11.457	3.788	6.628	2.61	3.54	n.a.
2		13.650	141.269	180.620	97.39	96.46	n.a.
Total:			**145.056**	**187.248**	**100.00**	**100.00**	

Compound **2z:** HPLC (ADH, *n*-hexane/2-propanol = 70/30, v = 1.0 mL/min, λ = 254 nm)

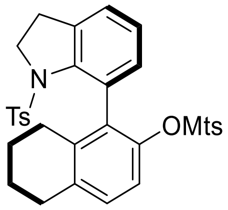

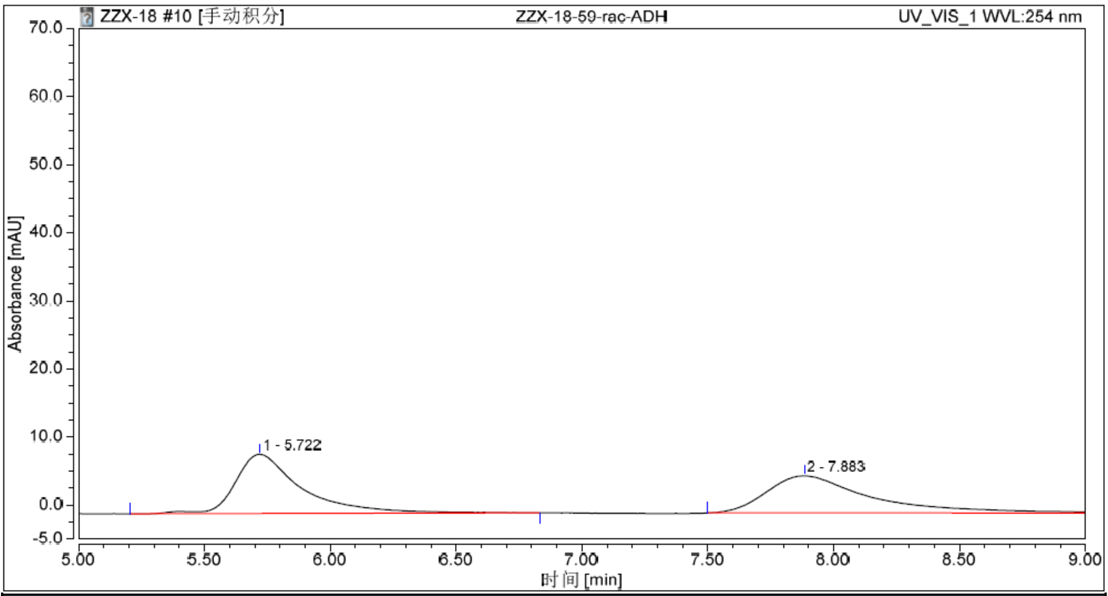

Integration Results							
No.	Peak Name	Retention Time min	Area mAU*min	Height mAU	Relative Area %	Relative Height %	Amount n.a.
1		5.722	2.638	8.693	50.18	61.48	n.a.
2		7.883	2.619	5.446	49.82	38.52	n.a.
**Total:**			**5.257**	**14.140**	**100.00**	**100.00**	

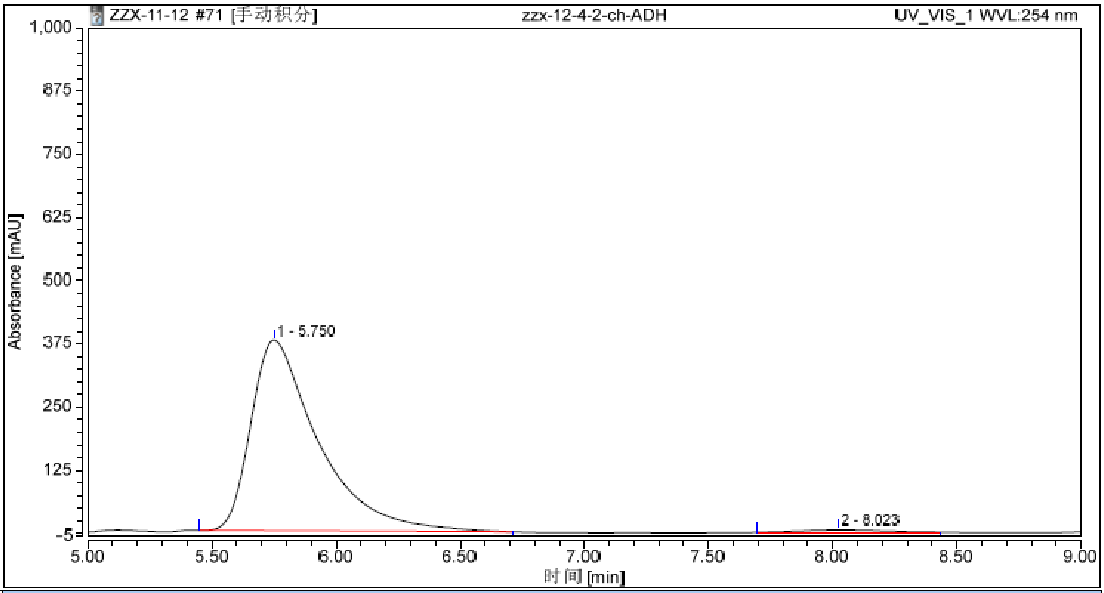

Integration Results							
No.	Peak Name	Retention Time min	Area mAU*min	Height mAU	Relative Area %	Relative Height %	Amount n.a.
1		5.750	118.562	376.645	98.60	98.80	n.a.
2		8.023	1.687	4.562	1.40	1.20	n.a.
**Total:**			**120.249**	**381.207**	**100.00**	**100.00**	

Compound **2aa:** HPLC (ODH, *n*-hexane/2-propanol = 70/30, v = 1.0 mL/min, λ = 254 nm)

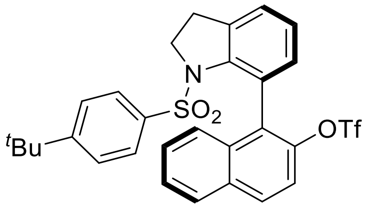

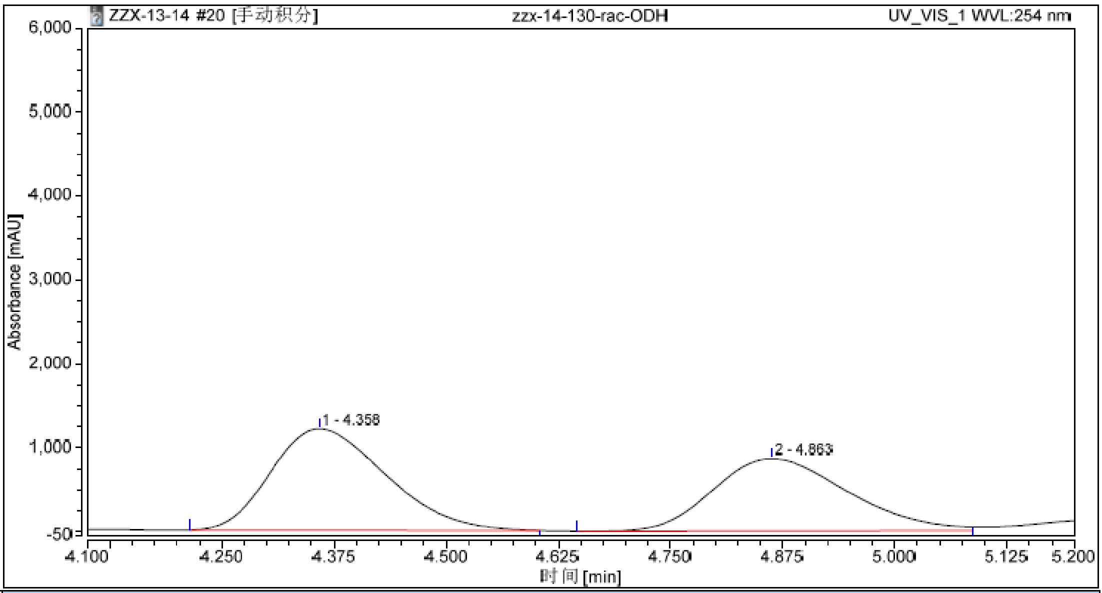

Integration Results

No.	Peak Name	Retention Time min	Area mAU*min	Height mAU	Relative Area %	Relative Height %	Amount n.a.
1		4.358	179.552	1208.526	54.50	58.38	n.a.
2		4.863	149.906	861.425	45.50	41.62	n.a.
Total:			329.458	2069.951	100.00	100.00	

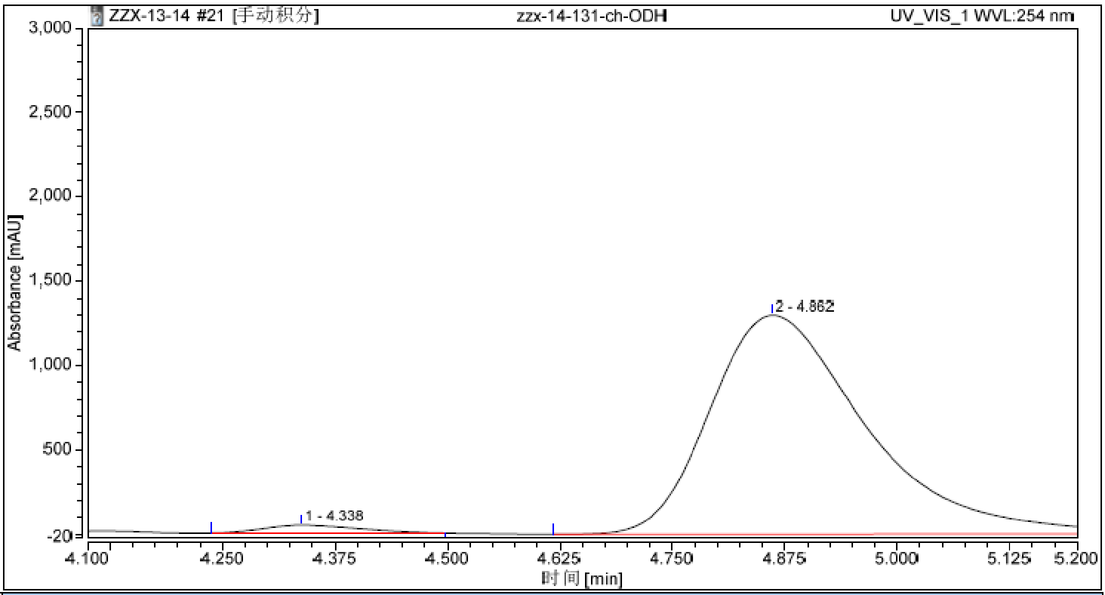

Integration Results

No.	Peak Name	Retention Time min	Area mAU*min	Height mAU	Relative Area %	Relative Height %	Amount n.a.
1		4.338	5.749	47.798	2.15	3.55	n.a.
2		4.862	262.216	1297.123	97.85	96.45	n.a.
Total:			267.965	1344.921	100.00	100.00	

Compound **2ab:** HPLC (IG, *n*-hexane/2-propanol = 85/15, v = 1.0 mL/min, λ = 254 nm)

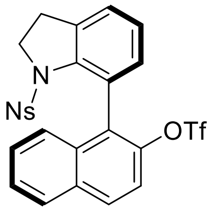

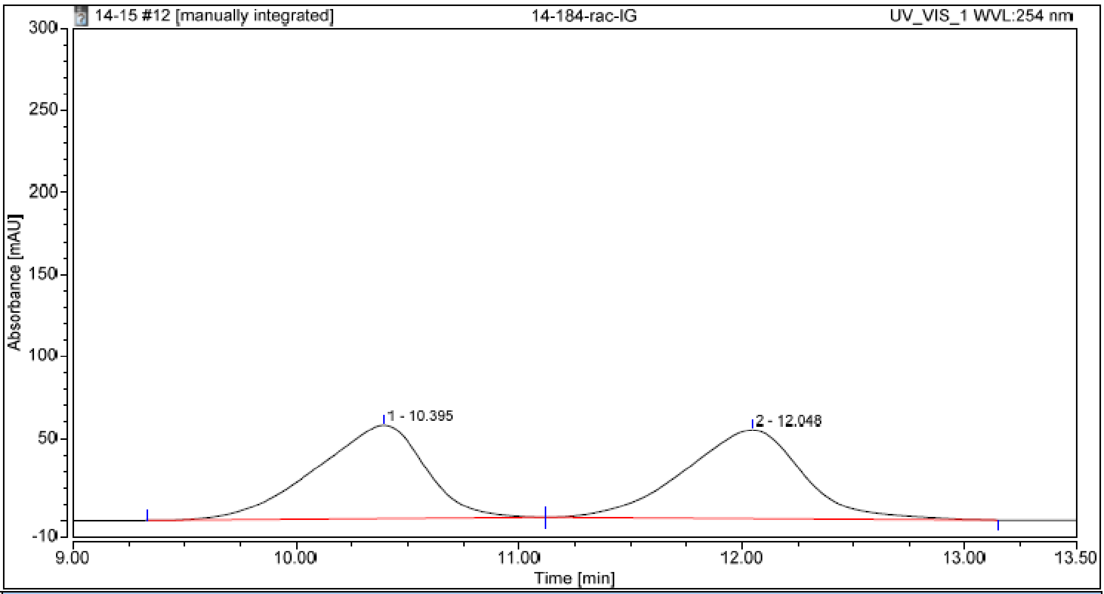

Integration Results							
No.	Peak Name	Retention Time min	Area mAU*min	Height mAU	Relative Area %	Relative Height %	Amount n.a.
1		10.395	32.550	56.983	49.50	51.37	n.a.
2		12.048	33.212	53.947	50.50	48.63	n.a.
Total:			65.763	110.930	100.00	100.00	

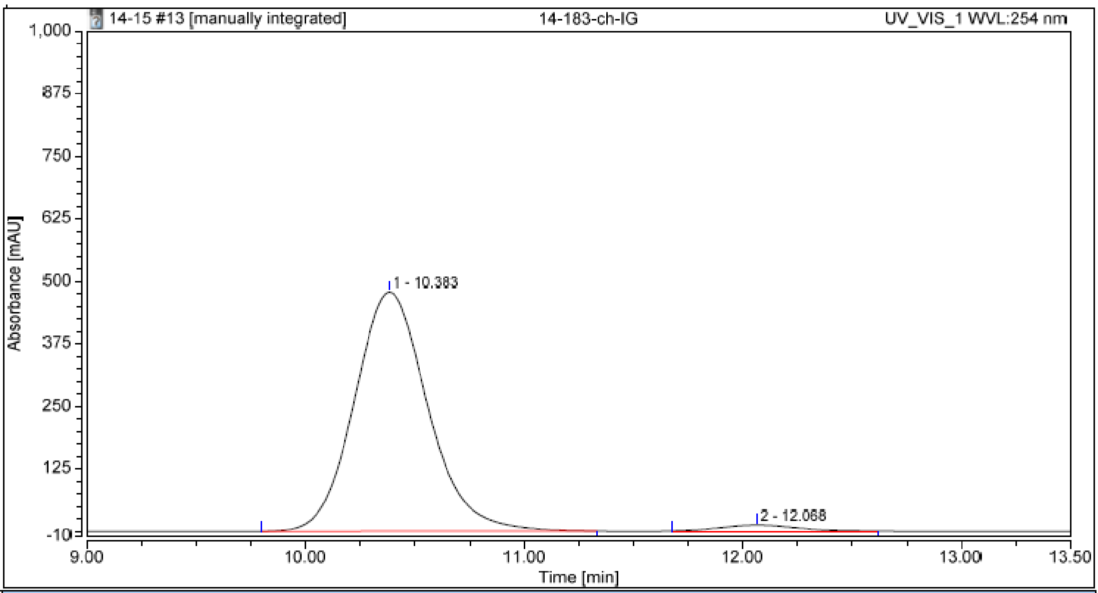

Integration Results							
No.	Peak Name	Retention Time min	Area mAU*min	Height mAU	Relative Area %	Relative Height %	Amount n.a.
1		10.383	186.491	478.230	97.41	97.46	n.a.
2		12.068	4.964	12.453	2.59	2.54	n.a.
Total:			191.455	490.683	100.00	100.00	

Compound **2ac:** HPLC (IA, *n*-hexane/2-propanol = 85/15, v = 1.0 mL/min, λ = 254 nm)

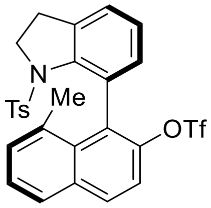

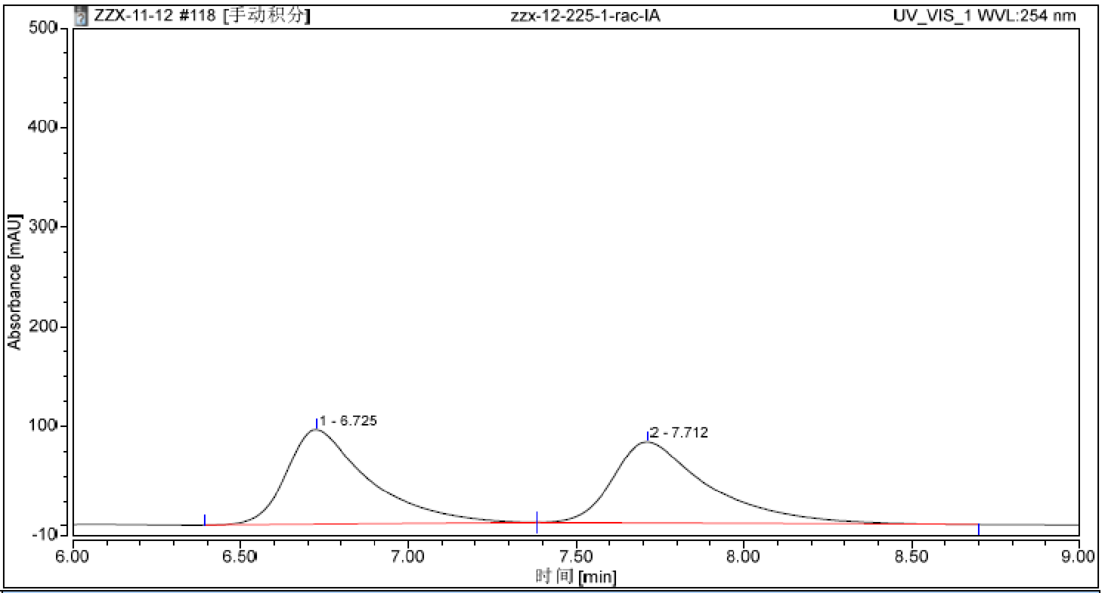

Integration Results

No.	Peak Name	Retention Time min	Area mAU*min	Height mAU	Relative Area %	Relative Height %	Amount n.a.
1		6.725	27.010	94.777	50.14	53.85	n.a.
2		7.712	26.860	81.232	49.86	46.15	n.a.
Total:			**53.870**	**176.009**	**100.00**	**100.00**	

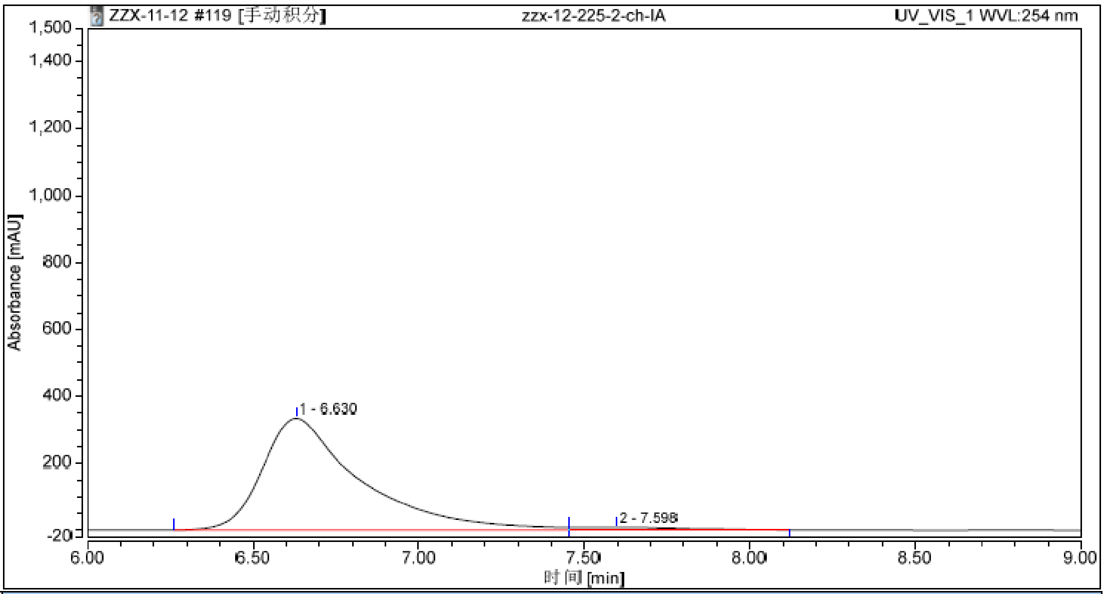

Integration Results

No.	Peak Name	Retention Time min	Area mAU*min	Height mAU	Relative Area %	Relative Height %	Amount n.a.
1		6.630	113.676	333.091	97.58	97.66	n.a.
2		7.598	2.817	7.976	2.42	2.34	n.a.
Total:			**116.493**	**341.067**	**100.00**	**100.00**	

Compound **2ad:** HPLC (IA, *n*-hexane/2-propanol = 70/30, v = 1.0 mL/min, λ = 254 nm)

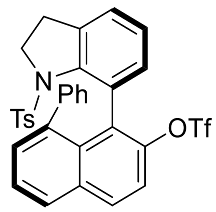

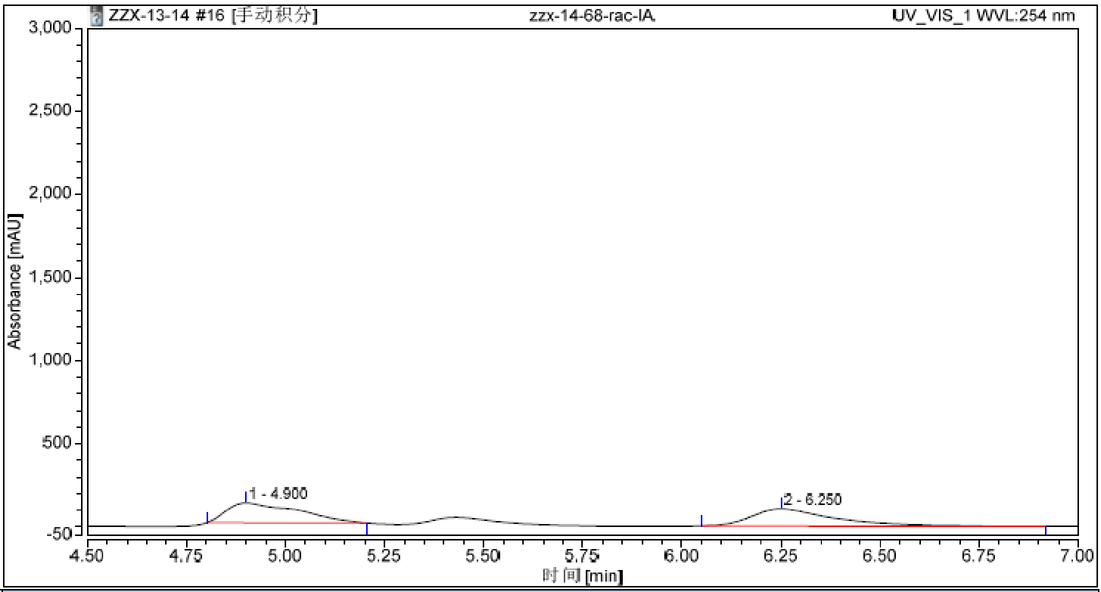

Integration Results

No.	Peak Name	Retention Time min	Area mAU*min	Height mAU	Relative Area %	Relative Height %	Amount n.a.
1		4.900	24.994	119.962	50.44	53.84	n.a.
2		6.250	24.562	102.869	49.56	46.16	n.a.
Total:			49.556	222.831	100.00	100.00	

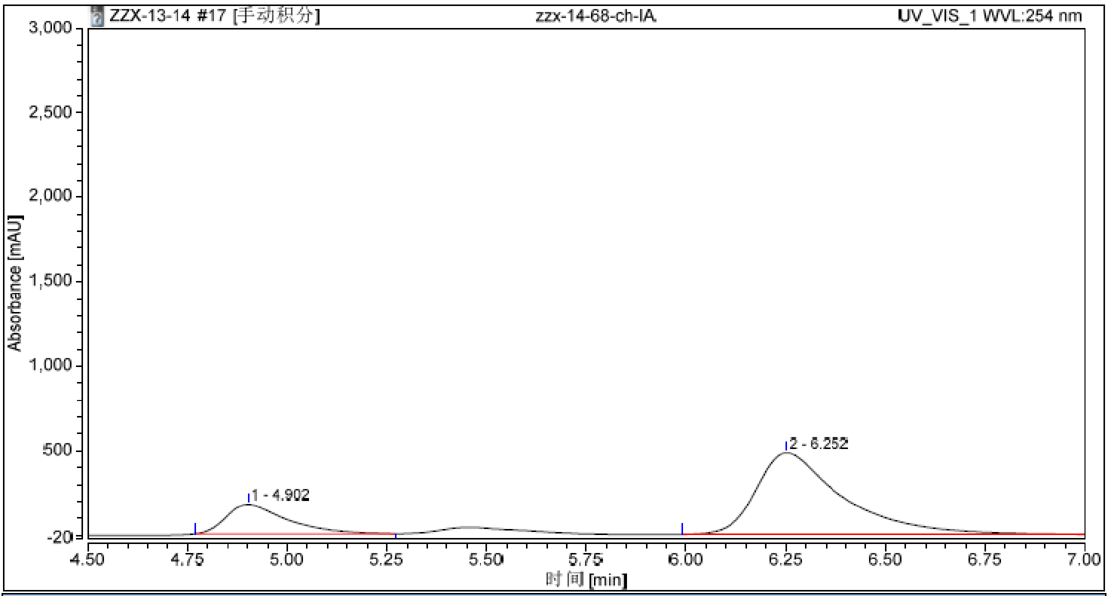

Integration Results

No.	Peak Name	Retention Time min	Area mAU*min	Height mAU	Relative Area %	Relative Height %	Amount n.a.
1		4.902	30.435	173.550	20.03	26.38	n.a.
2		6.252	121.509	484.393	79.97	73.62	n.a.
Total:			151.944	657.943	100.00	100.00	

Compound **2ae:** HPLC (ODH, *n*-hexane/2-propanol = 85/15, v = 1.0 mL/min, λ = 254 nm)

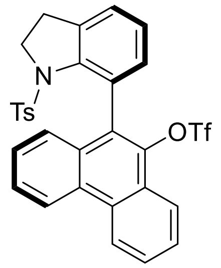

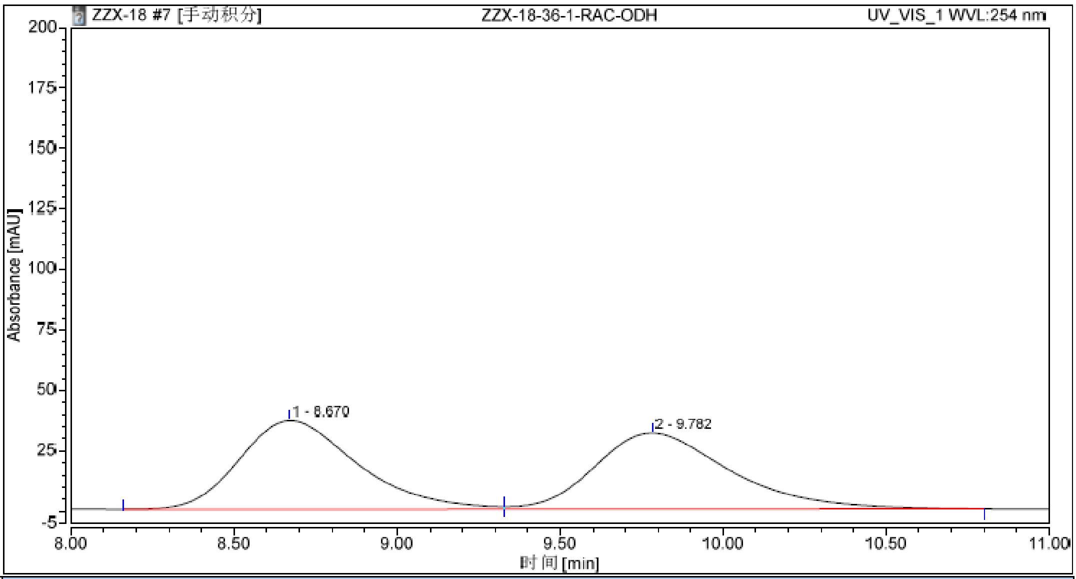

Integration Results

No.	Peak Name	Retention Time<br>min	Area<br>mAU*min	Height<br>mAU	Relative Area<br>%	Relative Height<br>%	Amount<br>n.a.
1		8.670	15.437	36.664	50.43	53.83	n.a.
2		9.782	15.171	31.443	49.57	46.17	n.a.
**Total:**			**30.608**	**68.107**	**100.00**	**100.00**	

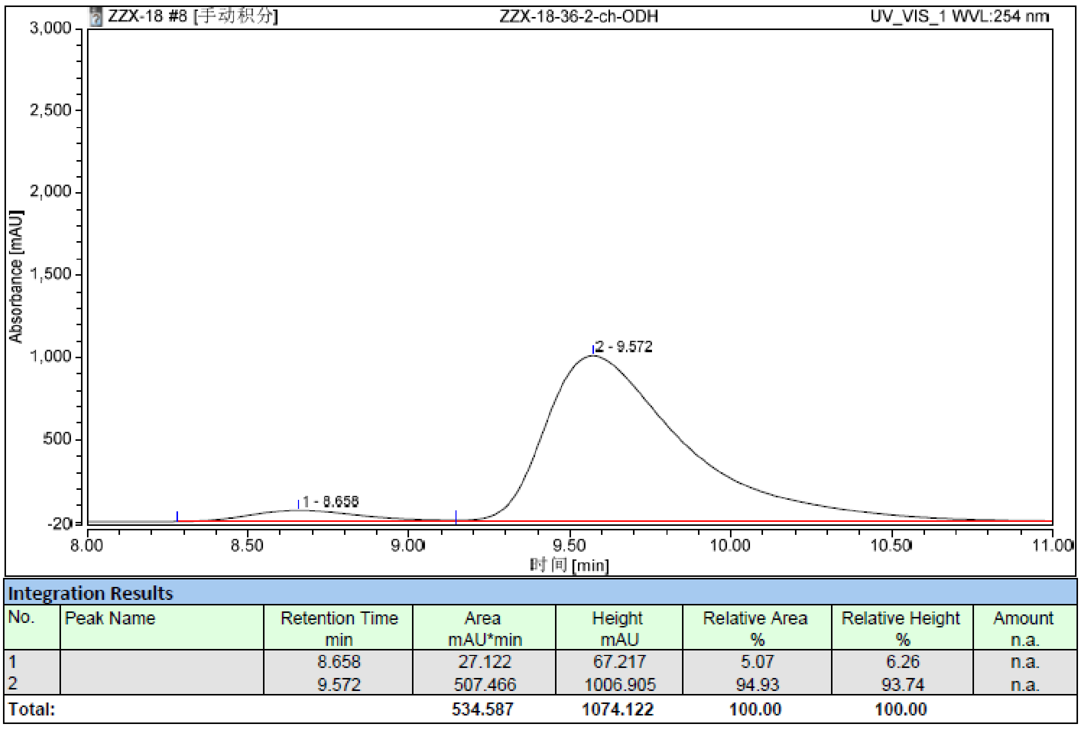

Integration Results

No.	Peak Name	Retention Time<br>min	Area<br>mAU*min	Height<br>mAU	Relative Area<br>%	Relative Height<br>%	Amount<br>n.a.
1		8.658	27.122	67.217	5.07	6.26	n.a.
2		9.572	507.466	1006.905	94.93	93.74	n.a.
**Total:**			**534.587**	**1074.122**	**100.00**	**100.00**	

Compound **2af:** HPLC (ADH, *n*-hexane/2-propanol = 90/10, v = 1.0 mL/min, λ = 254 nm)

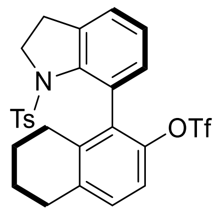

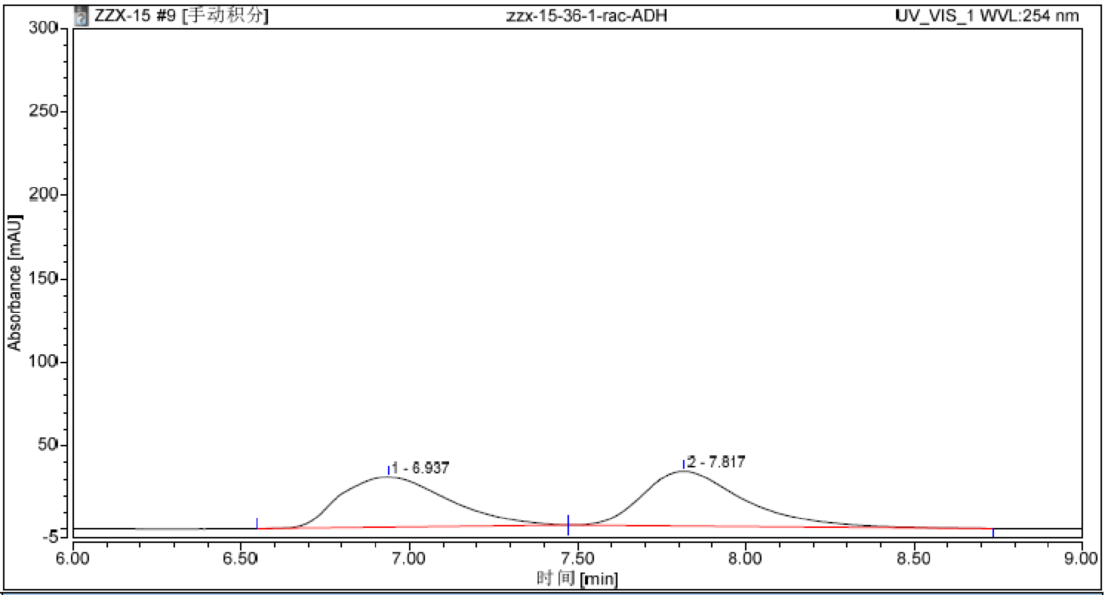

Integration Results

No.	Peak Name	Retention Time min	Area mAU*min	Height mAU	Relative Area %	Relative Height %	Amount n.a.
1		6.937	11.141	30.007	49.39	47.69	n.a.
2		7.817	11.418	32.908	50.61	52.31	n.a.
Total:			22.559	62.915	100.00	100.00	

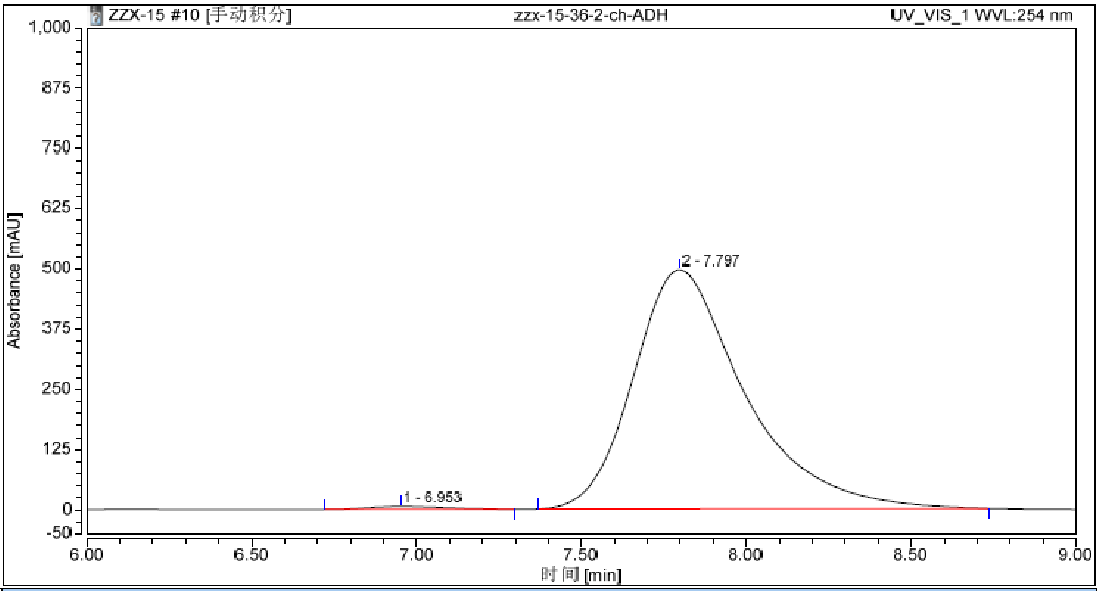

Integration Results

No.	Peak Name	Retention Time min	Area mAU*min	Height mAU	Relative Area %	Relative Height %	Amount n.a.
1		6.953	1.719	6.325	0.88	1.26	n.a.
2		7.797	194.300	495.637	99.12	98.74	n.a.
Total:			196.019	501.961	100.00	100.00	

Compound **2ag:** HPLC (ADH, *n*-hexane/2-propanol = 70/30, v = 1.0 mL/min, λ = 254 nm)

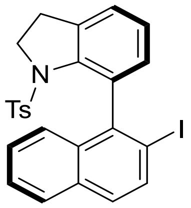

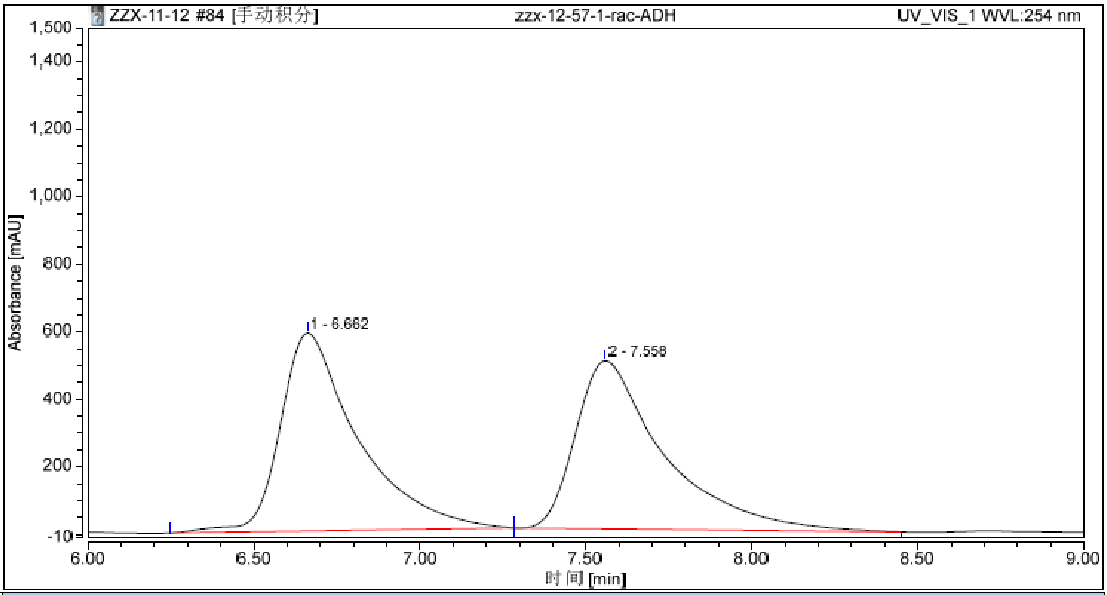

Integration Results

No.	Peak Name	Retention Time min	Area mAU*min	Height mAU	Relative Area %	Relative Height %	Amount n.a.
1		6.662	158.521	585.913	50.87	54.11	n.a.
2		7.558	153.079	496.981	49.13	45.89	n.a.
**Total:**			**311.600**	**1082.894**	**100.00**	**100.00**	

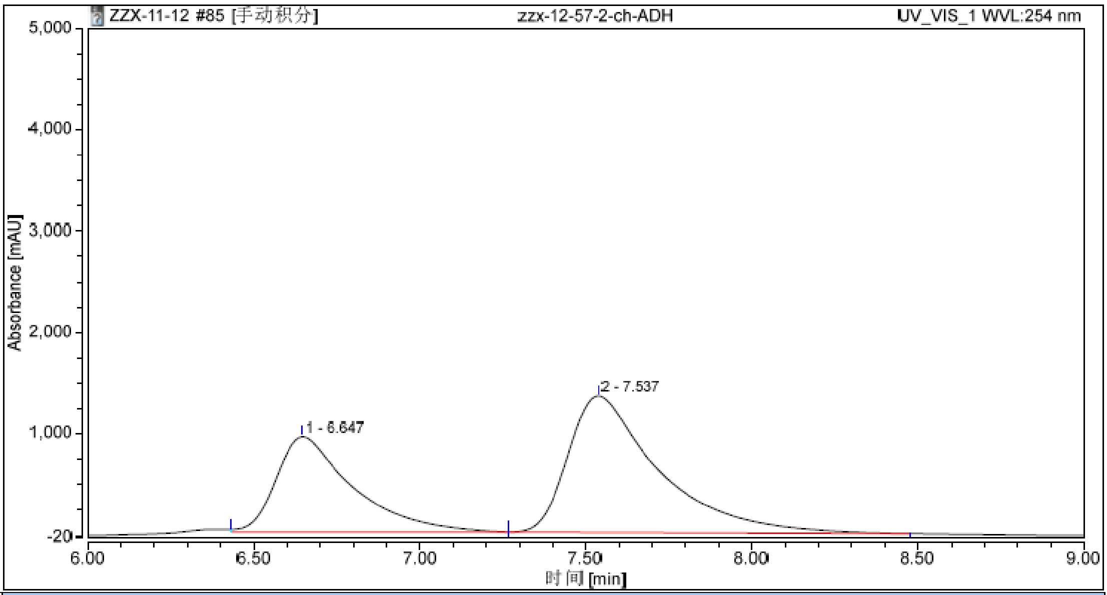

Integration Results

No.	Peak Name	Retention Time min	Area mAU*min	Height mAU	Relative Area %	Relative Height %	Amount n.a.
1		6.647	251.441	939.252	37.27	41.14	n.a.
2		7.537	423.159	1343.874	62.73	58.86	n.a.
**Total:**			**674.600**	**2283.126**	**100.00**	**100.00**	

Compound **2ah:** HPLC (ADH, *n*-hexane/2-propanol = 85/15, v = 1.0 mL/min, λ = 254 nm)

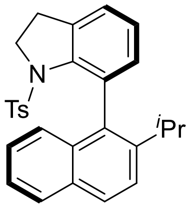

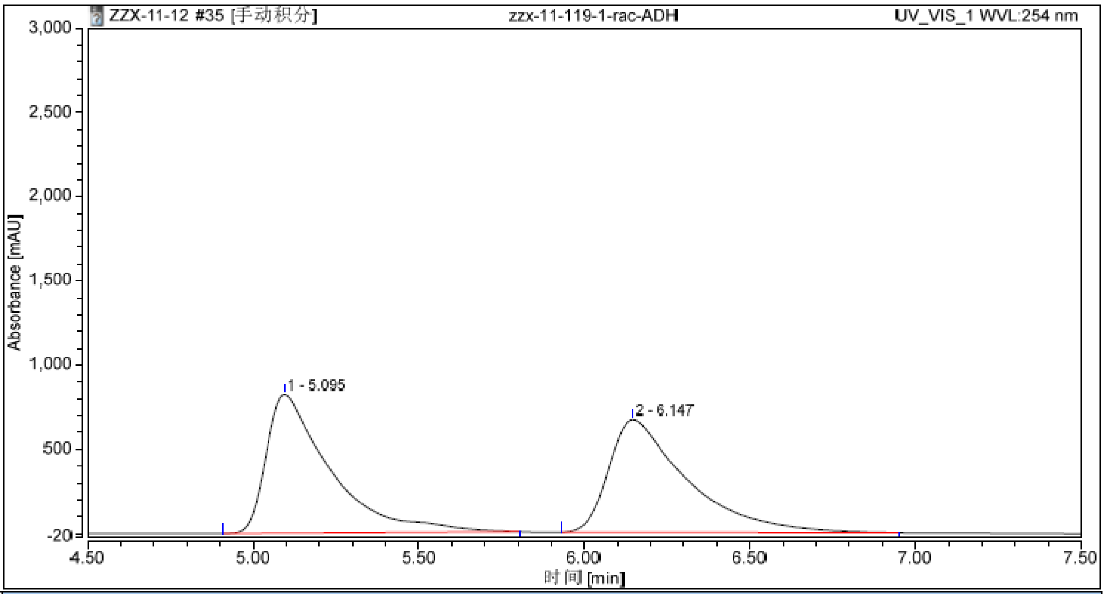

Integration Results

No.	Peak Name	Retention Time min	Area mAU*min	Height mAU	Relative Area %	Relative Height %	Amount n.a.
1		5.095	183.073	820.277	50.13	55.11	n.a.
2		6.147	182.139	668.224	49.87	44.89	n.a.
**Total:**			**365.212**	**1488.501**	**100.00**	**100.00**	

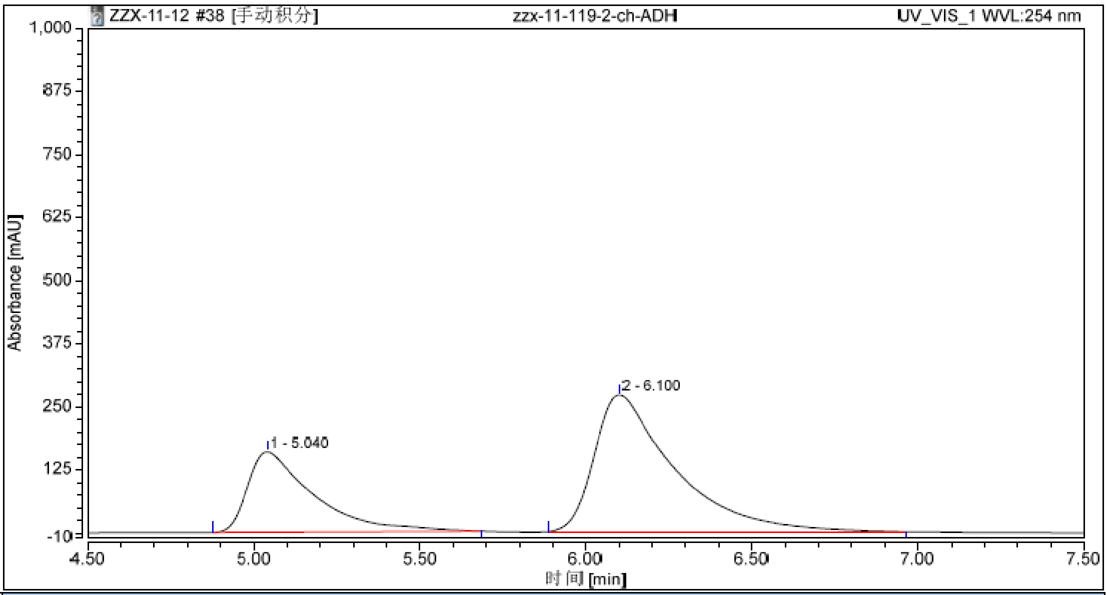

Integration Results

No.	Peak Name	Retention Time min	Area mAU*min	Height mAU	Relative Area %	Relative Height %	Amount n.a.
1		5.040	36.367	158.625	32.60	36.93	n.a.
2		6.100	75.196	270.934	67.40	63.07	n.a.
**Total:**			**111.563**	**429.559**	**100.00**	**100.00**	

Compound **2ai:** HPLC (ADH, *n*-hexane/2-propanol = 85/15, v = 1.0 mL/min, λ = 254 nm)

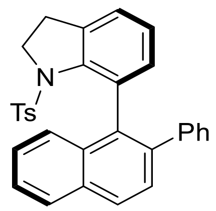

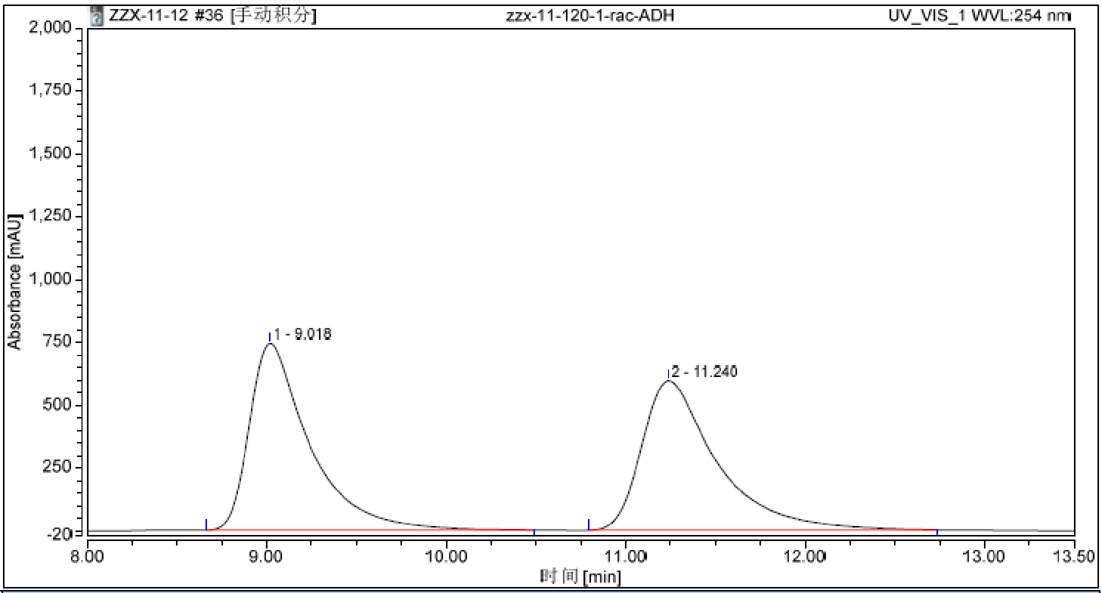

Integration Results

No.	Peak Name	Retention Time min	Area mAU*min	Height mAU	Relative Area %	Relative Height %	Amount n.a.
1		9.018	290.202	744.178	50.15	55.56	n.a.
2		11.240	288.437	595.281	49.85	44.44	n.a.
Total:			578.639	1339.459	100.00	100.00	

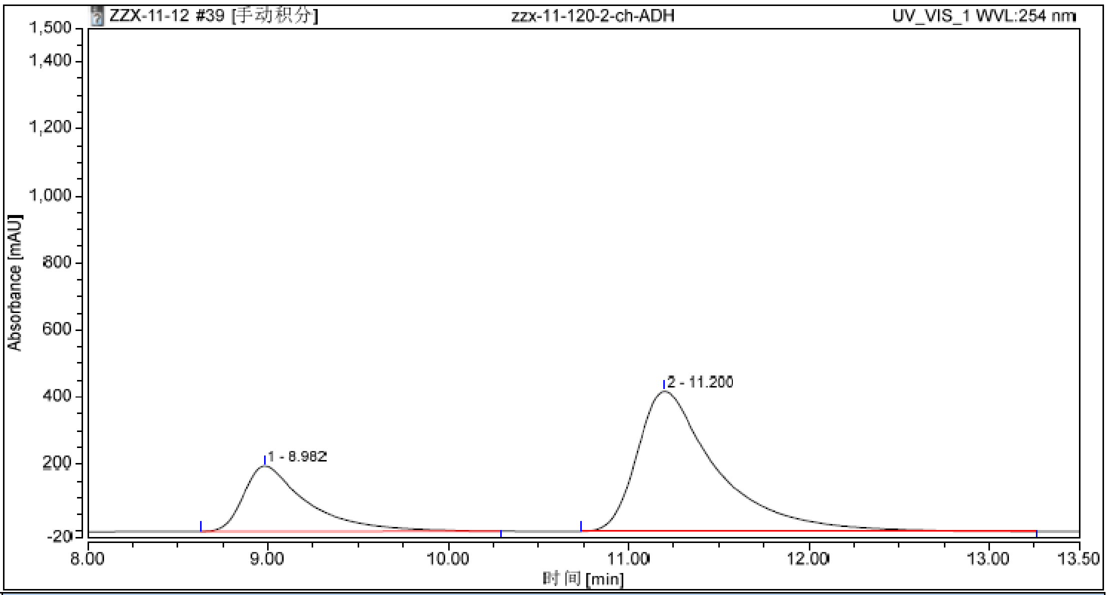

Integration Results

No.	Peak Name	Retention Time min	Area mAU*min	Height mAU	Relative Area %	Relative Height %	Amount n.a.
1		8.982	79.201	194.476	27.07	31.79	n.a.
2		11.200	213.353	417.312	72.93	68.21	n.a.
Total:			292.554	611.787	100.00	100.00	

2aj: HPLC (ODH, *n*-hexane/2-propanol = 70/30, v = 1.0 mL/min, λ = 254 nm)

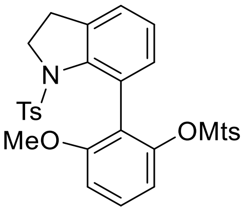

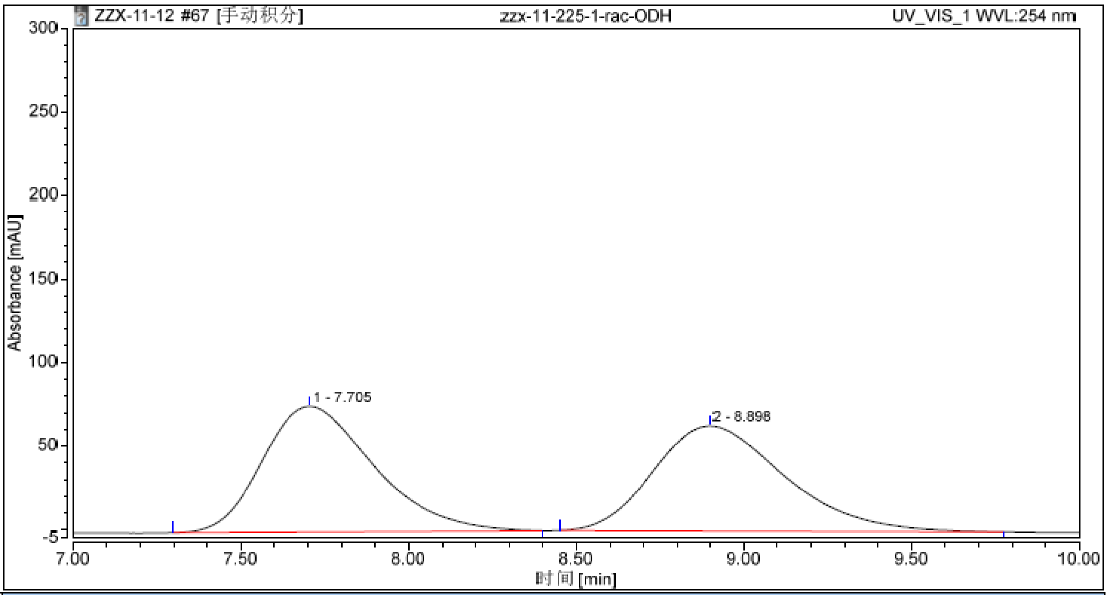

Integration Results

No.	Peak Name	Retention Time min	Area mAU*min	Height mAU	Relative Area %	Relative Height %	Amount n.a.
1		7.705	28.764	74.896	50.40	54.47	n.a.
2		8.898	28.307	62.597	49.60	45.53	n.a.
**Total:**			**57.072**	**137.493**	**100.00**	**100.00**	

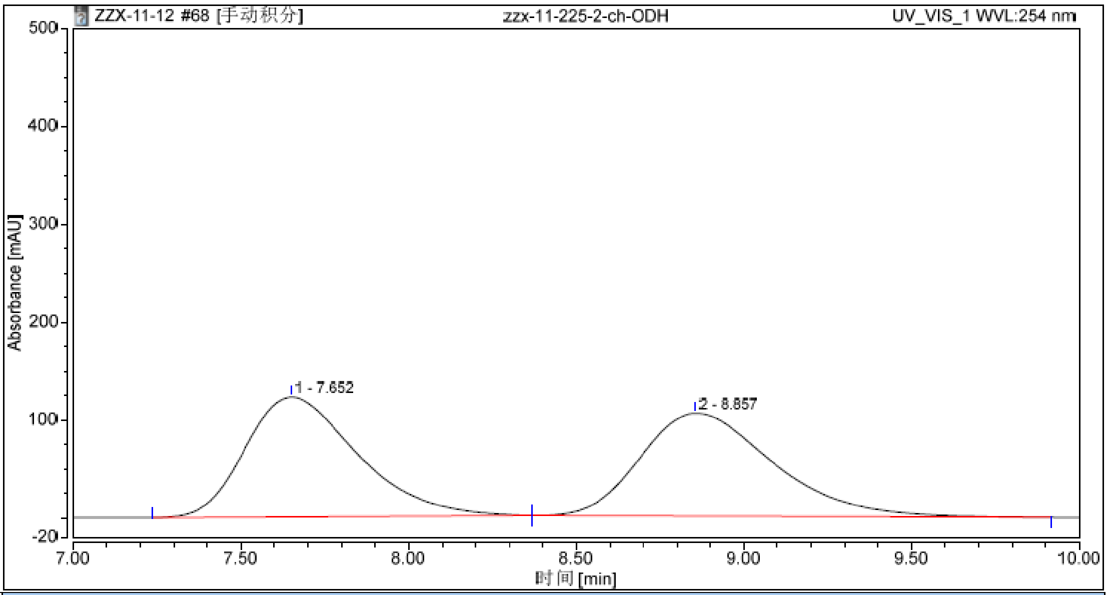

Integration Results

No.	Peak Name	Retention Time min	Area mAU*min	Height mAU	Relative Area %	Relative Height %	Amount n.a.
1		7.652	47.407	122.237	49.67	53.81	n.a.
2		8.857	48.040	104.925	50.33	46.19	n.a.
**Total:**			**95.447**	**227.162**	**100.00**	**100.00**	

Compound **4:** HPLC (ODH, *n*-hexane/2-propanol = 90/10, v = 1.0 mL/min, λ = 254 nm)

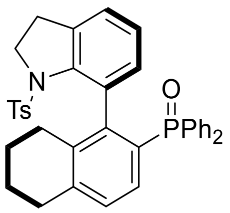

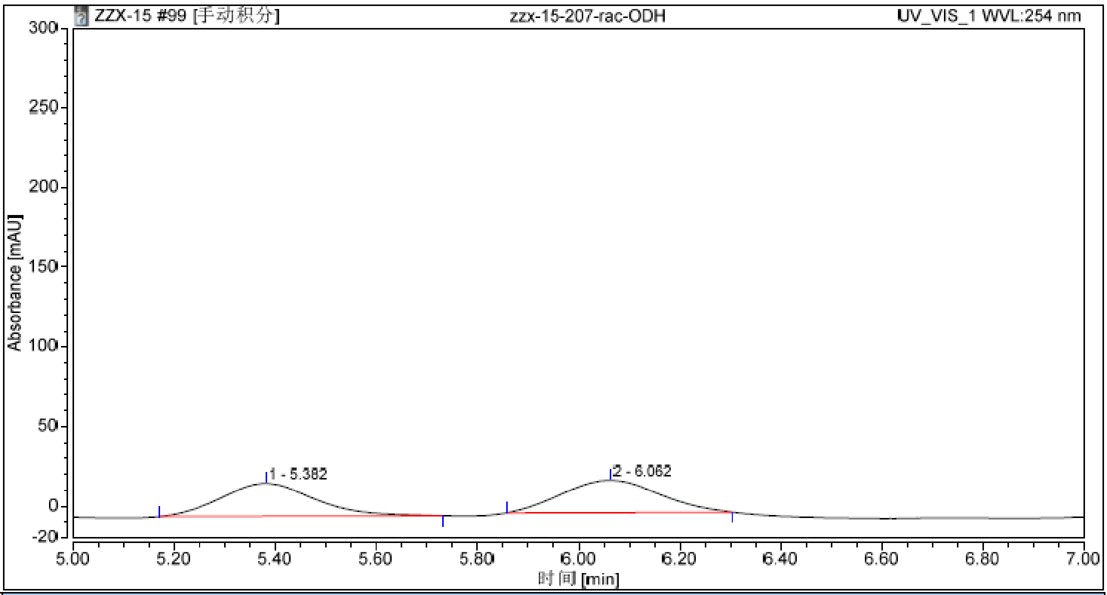

Integration Results

No.	Peak Name	Retention Time min	Area mAU*min	Height mAU	Relative Area %	Relative Height %	Amount n.a.
1		5.382	4.397	20.529	49.40	50.43	n.a.
2		6.062	4.503	20.175	50.60	49.57	n.a.
**Total:**			**8.900**	**40.704**	**100.00**	**100.00**	

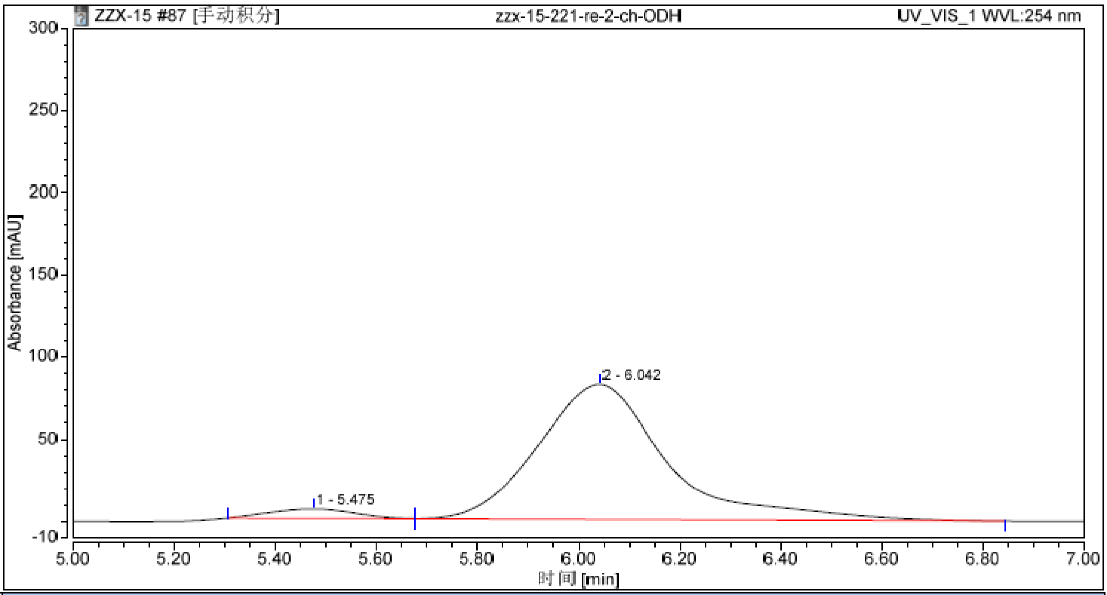

Integration Results

No.	Peak Name	Retention Time min	Area mAU*min	Height mAU	Relative Area %	Relative Height %	Amount n.a.
1		5.475	1.073	5.684	4.27	6.48	n.a.
2		6.042	24.070	82.068	95.73	93.52	n.a.
**Total:**			**25.143**	**87.752**	**100.00**	**100.00**	

Compound **5:** HPLC (IA, *n*-hexane/2-propanol = 80/20, v = 1.0 mL/min, λ = 254 nm)

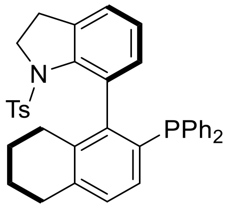

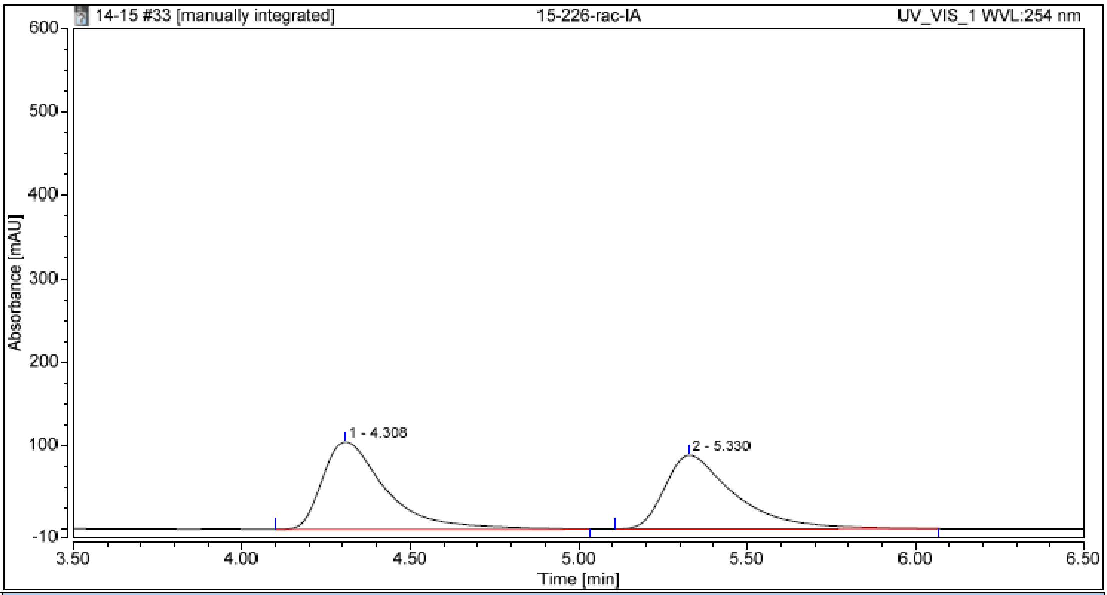

Integration Results

No.	Peak Name	Retention Time min	Area mAU*min	Height mAU	Relative Area %	Relative Height %	Amount n.a.
1		4.308	22.633	104.465	51.67	54.26	n.a.
2		5.330	21.166	88.075	48.33	45.74	n.a.
**Total:**			**43.799**	**192.541**	**100.00**	**100.00**	

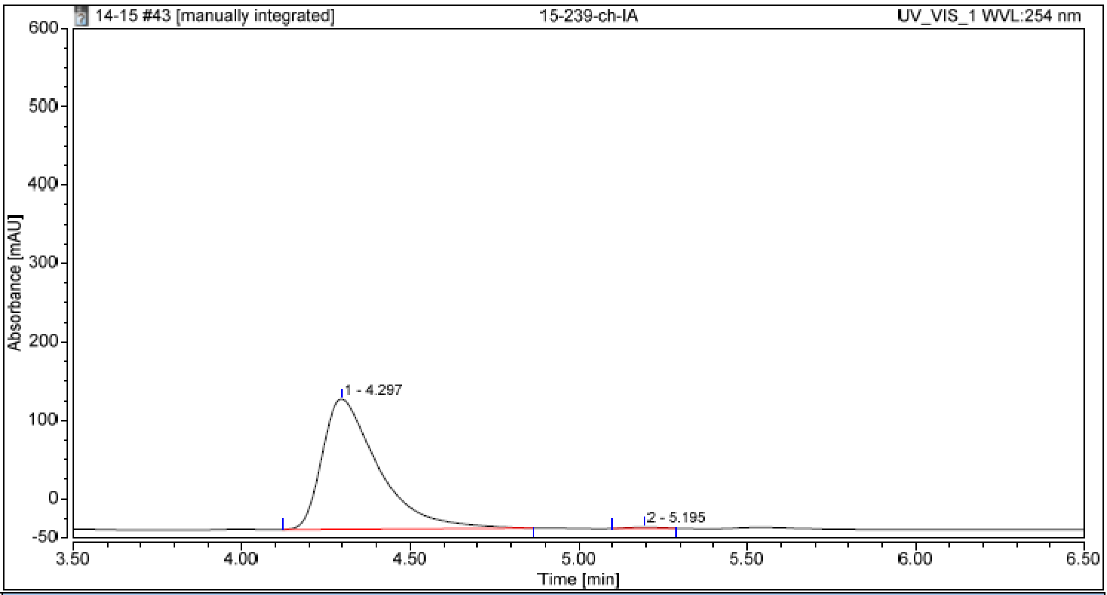

Integration Results

No.	Peak Name	Retention Time min	Area mAU*min	Height mAU	Relative Area %	Relative Height %	Amount n.a.
1		4.297	31.996	165.628	99.48	99.12	n.a.
2		5.195	0.166	1.469	0.52	0.88	n.a.
**Total:**			**32.162**	**167.098**	**100.00**	**100.00**	

8: HPLC (ADH, *n*-hexane/2-propanol = 90/10, v = 1.0 mL/min, λ = 254 nm)

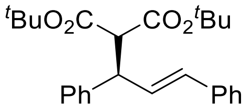

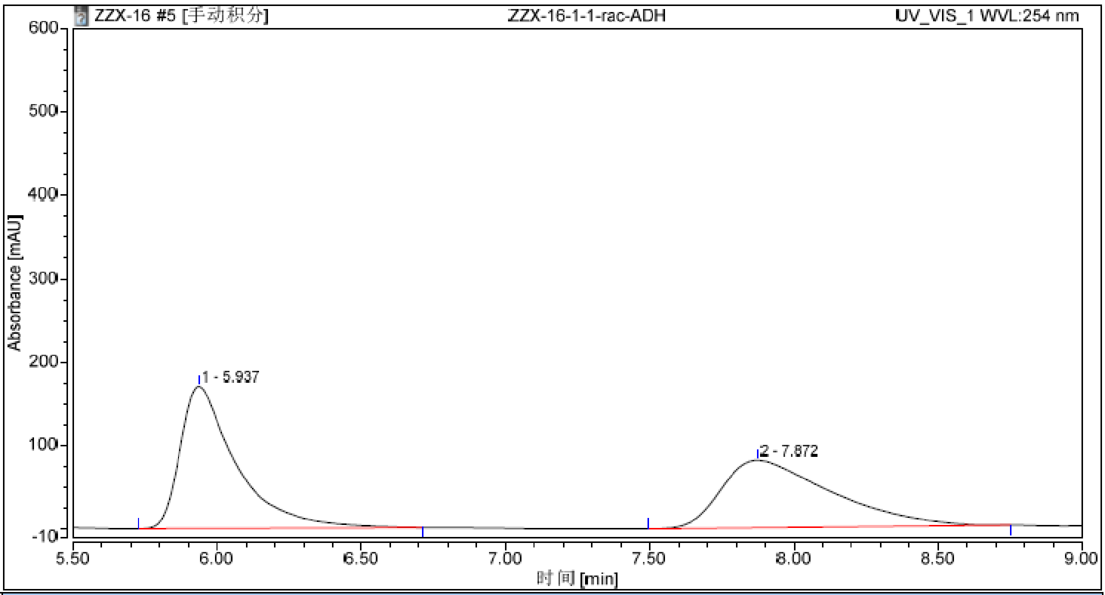

Integration Results

No.	Peak Name	Retention Time min	Area mAU*min	Height mAU	Relative Area %	Relative Height %	Amount n.a.
1		5.937	38.350	170.145	51.81	67.79	n.a.
2		7.872	35.674	80.838	48.19	32.21	n.a.
Total:			74.024	250.982	100.00	100.00	

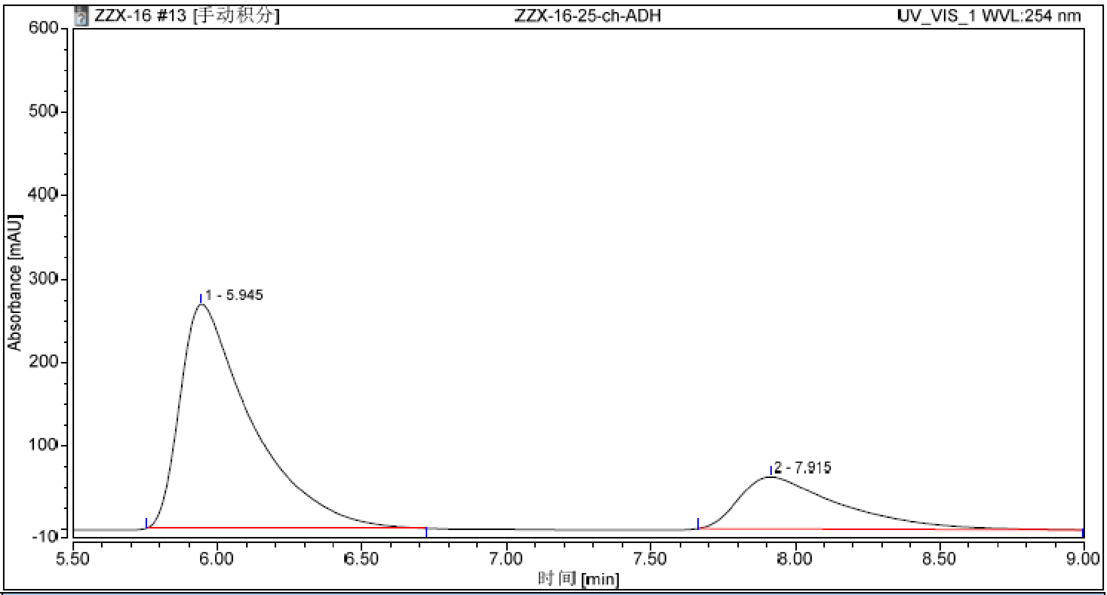

Integration Results

No.	Peak Name	Retention Time min	Area mAU*min	Height mAU	Relative Area %	Relative Height %	Amount n.a.
1		5.945	77.225	268.059	75.61	81.20	n.a.
2		7.915	24.911	62.046	24.39	18.80	n.a.
Total:			102.135	330.105	100.00	100.00	

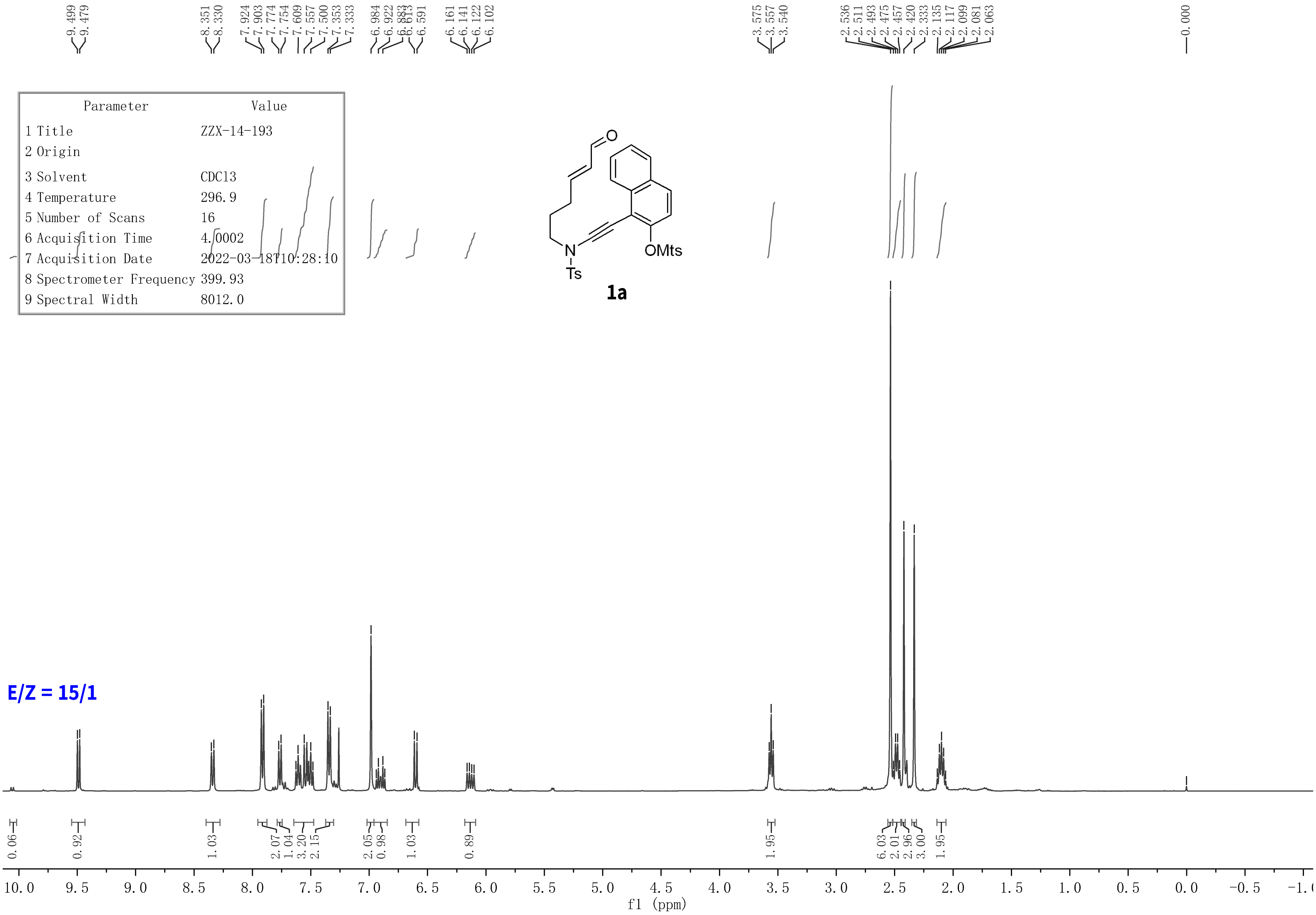

9.499
9.479
8.351
8.330
7.924
7.903
7.774
7.754
7.609
7.557
7.500
7.353
7.333
6.984
6.922
6.591
6.161
6.141
6.122
6.102
3.575
3.557
3.540
2.536
2.511
2.493
2.475
2.457
2.420
2.333
2.135
2.117
2.099
2.081
2.063
0.000
Parameter Value
1 Title ZZX-14-193
2 Origin
3 Solvent CDCl3
4 Temperature 296.9
5 Number of Scans 16
6 Acquisition Time 4.0002
7 Acquisition Date 2022-03-18T10:28:10
8 Spectrometer Frequency 399.93
9 Spectral Width 8012.0
O
N
Ts
OMts
1a
E/Z = 15/1
0.06
0.92
1.03
2.07
1.04
3.20
2.15
2.05
0.98
1.03
0.89
1.95
6.03
2.01
2.96
3.00
1.95
f1 (ppm)

Parameter	Value
1 Title	ZZX-14-193
2 Origin	
3 Solvent	CDCl3
4 Temperature	297.2
5 Number of Scans	200
6 Acquisition Time	1.0000
7 Acquisition Date	2022-03-18T10:35:25
8 Spectrometer Frequency	100.56
9 Spectral Width	26041.0

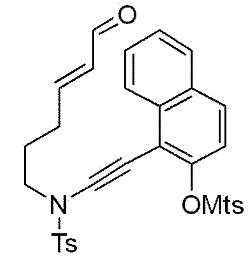

1a

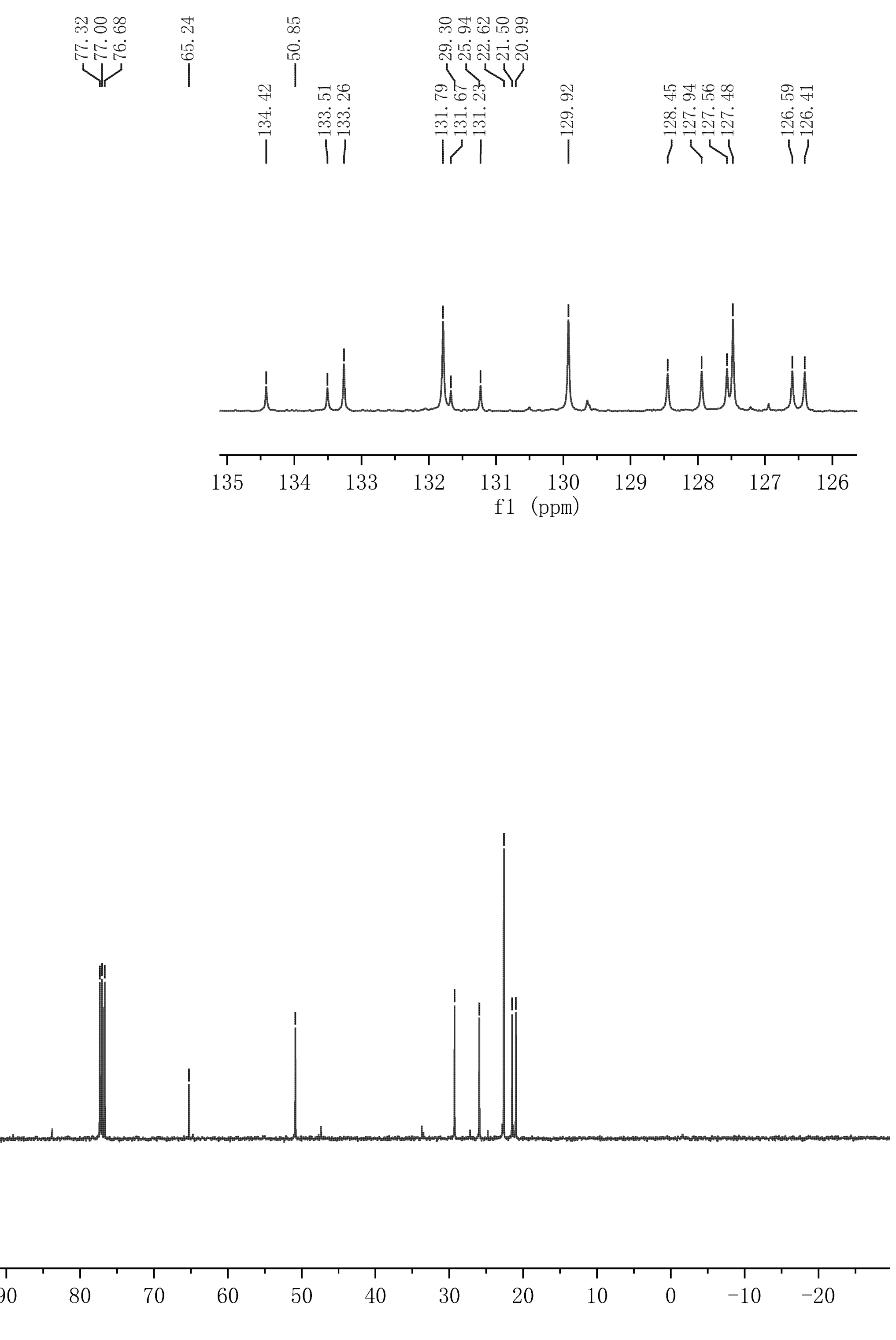

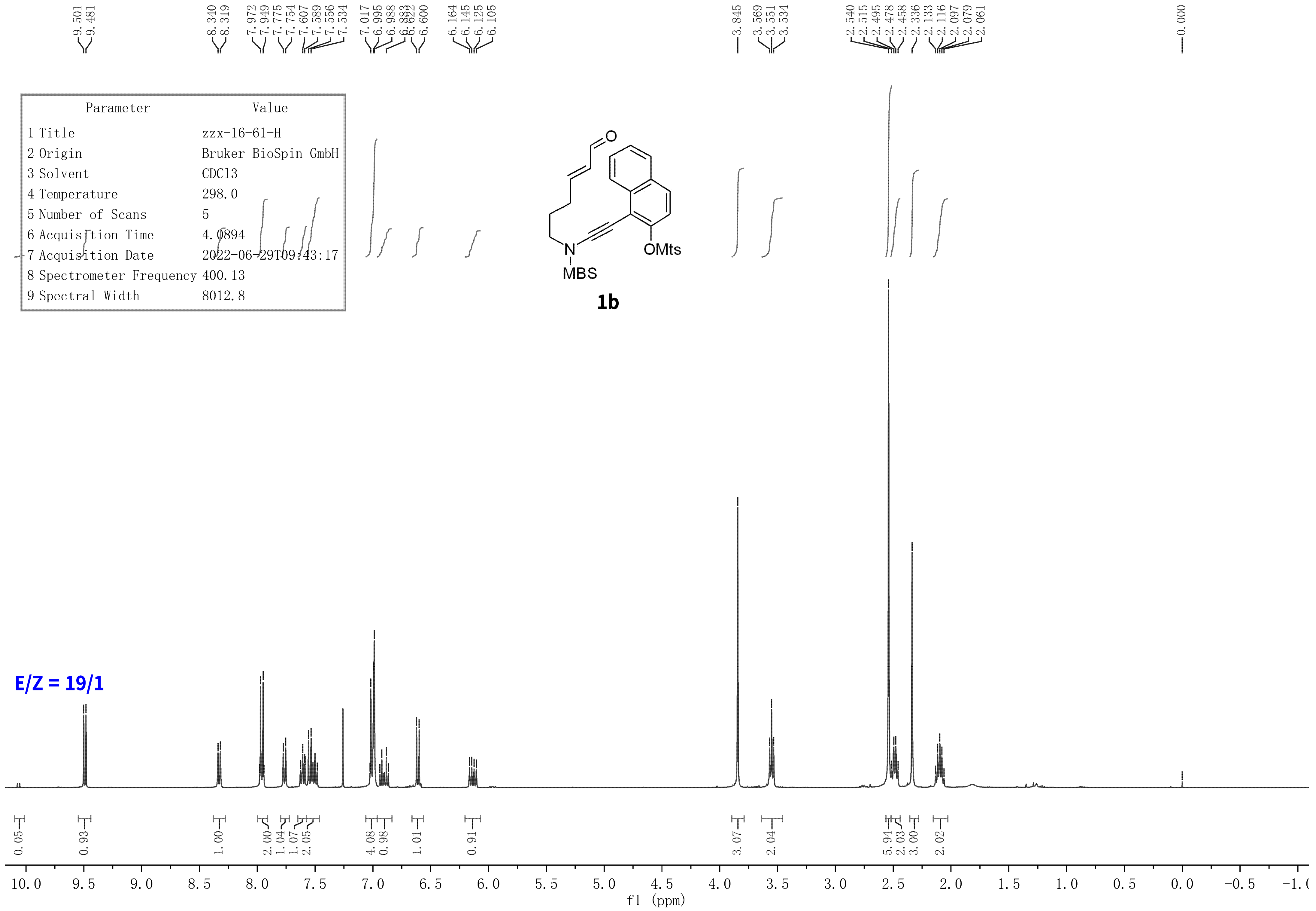

9.501
9.481
8.340
8.319
7.972
7.949
7.775
7.754
7.607
7.589
7.556
7.534
7.017
6.995
6.988
6.622
6.600
6.164
6.145
6.125
6.105
3.845
3.569
3.551
3.534
2.540
2.515
2.495
2.478
2.458
2.336
2.133
2.116
2.097
2.079
2.061
0.000
Parameter Value
1 Title zzx-16-61-H
2 Origin Bruker BioSpin GmbH
3 Solvent CDCl3
4 Temperature 298.0
5 Number of Scans 5
6 Acquisition Time 4.0894
7 Acquisition Date 2022-06-29T09:43:17
8 Spectrometer Frequency 400.13
9 Spectral Width 8012.8
O
N
MBS
OMts
1b
E/Z = 19/1
0.05
0.93
1.00
2.00
1.04
1.07
2.05
4.08
0.98
1.01
0.91
3.07
2.04
5.94
2.03
3.00
2.02
10.0
9.5
9.0
8.5
8.0
7.5
7.0
6.5
6.0
5.5
5.0
4.5
4.0
3.5
3.0
2.5
2.0
1.5
1.0
0.5
0.0
-0.5
f1 (ppm)

Parameter	Value
1 Title	zzx-16-61-C
2 Origin	Bruker BioSpin GmbH
3 Solvent	CDCl3
4 Temperature	300.0
5 Number of Scans	27
6 Acquisition Time	1.3631
7 Acquisition Date	2022-06-29T09:45:27
8 Spectrometer Frequency	100.61
9 Spectral Width	24038.5

1b

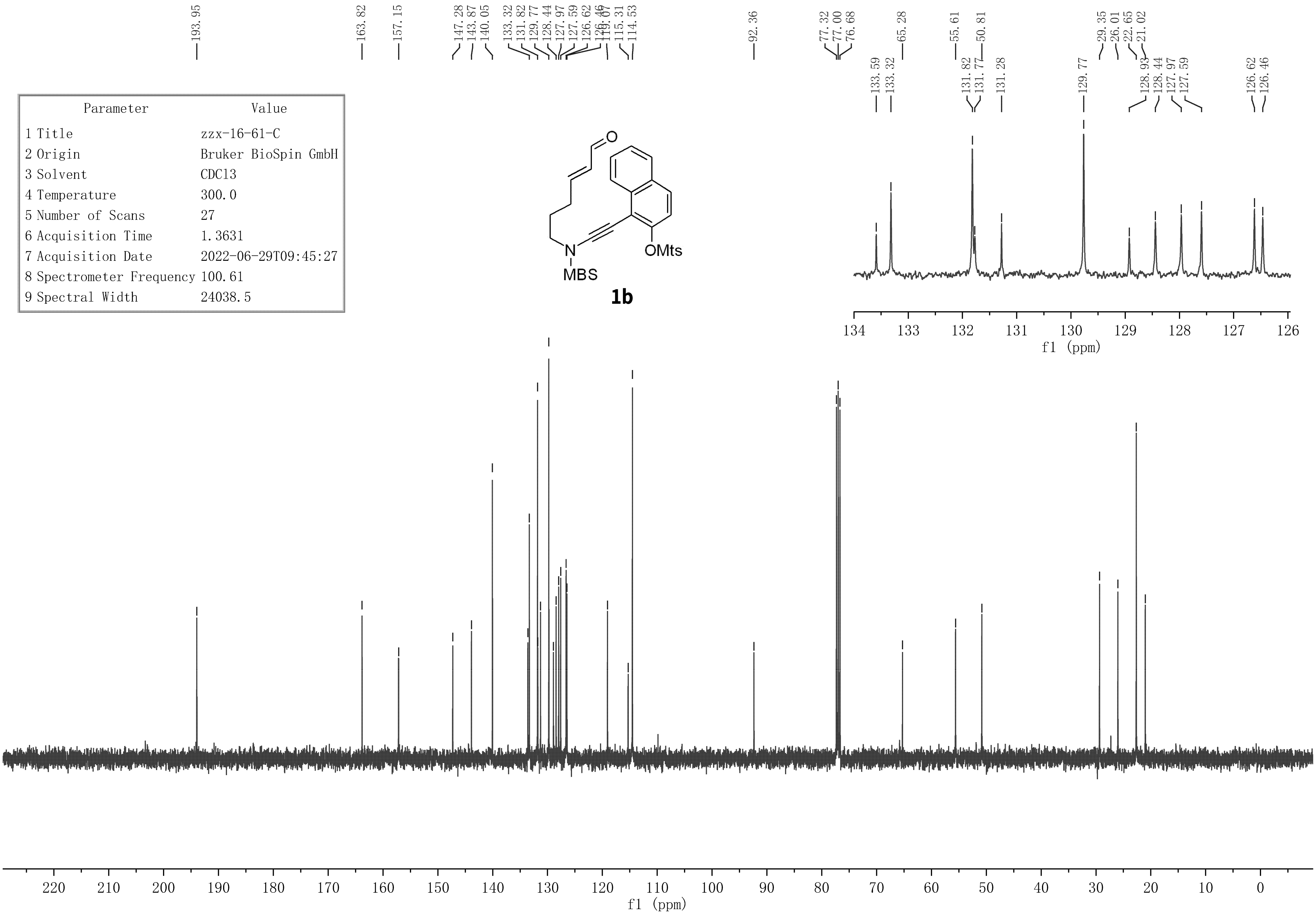

Parameter	Value
1 Title	zzx-16-60-H
2 Origin	Bruker BioSpin GmbH
3 Solvent	CDCl3
4 Temperature	298.0
5 Number of Scans	5
6 Acquisition Time	4.0894
7 Acquisition Date	2022-06-29T09:48:47
8 Spectrometer Frequency	400.13
9 Spectral Width	8012.8

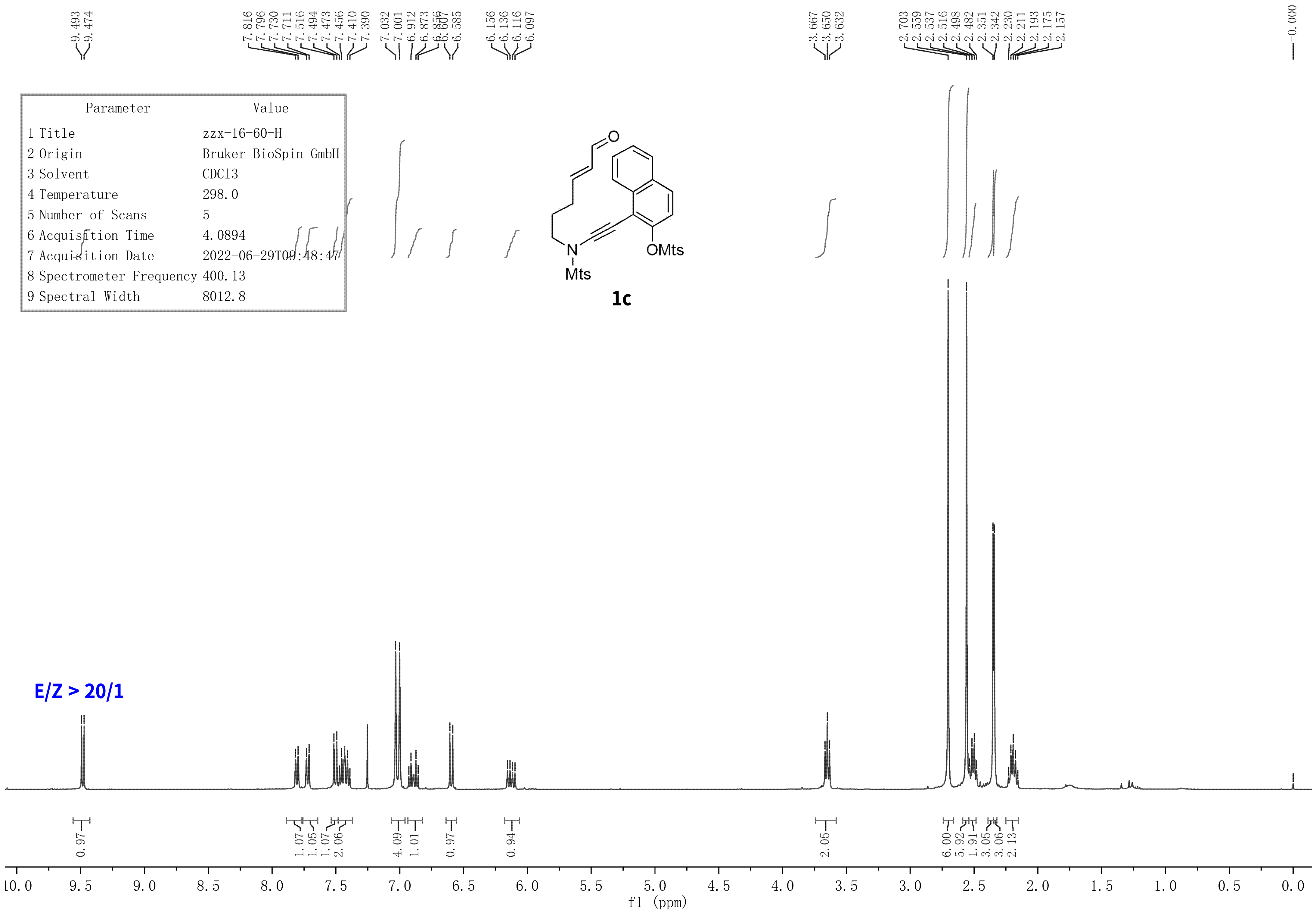

Parameter	Value
1 Title	zzx-16-60-C
2 Origin	Bruker BioSpin GmbH
3 Solvent	CDCl3
4 Temperature	300.0
5 Number of Scans	18
6 Acquisition Time	1.3631
7 Acquisition Date	2022-06-29T09:49:54
8 Spectrometer Frequency	100.61
9 Spectral Width	24038.5

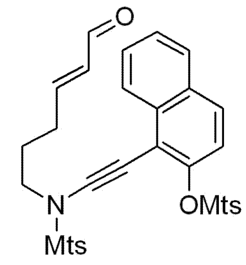

1c

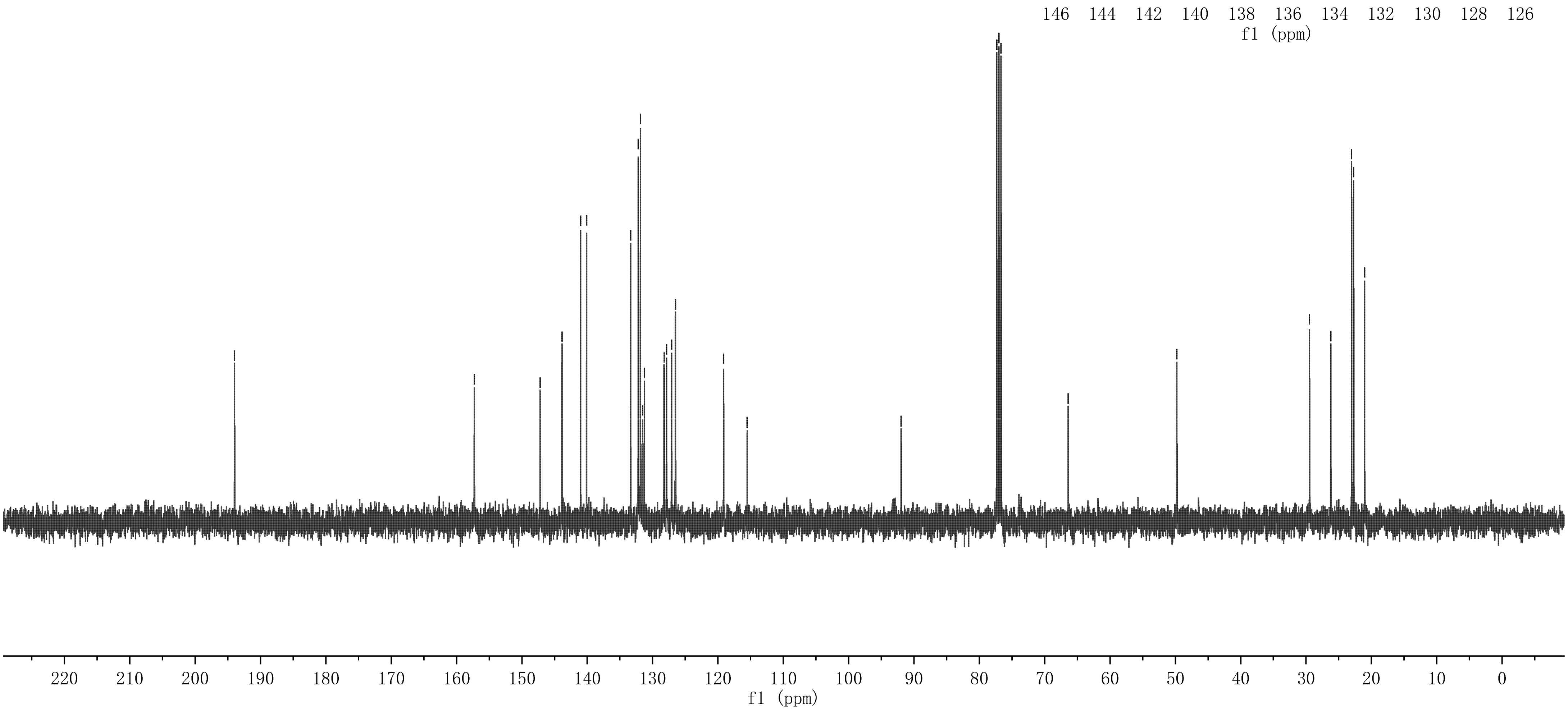

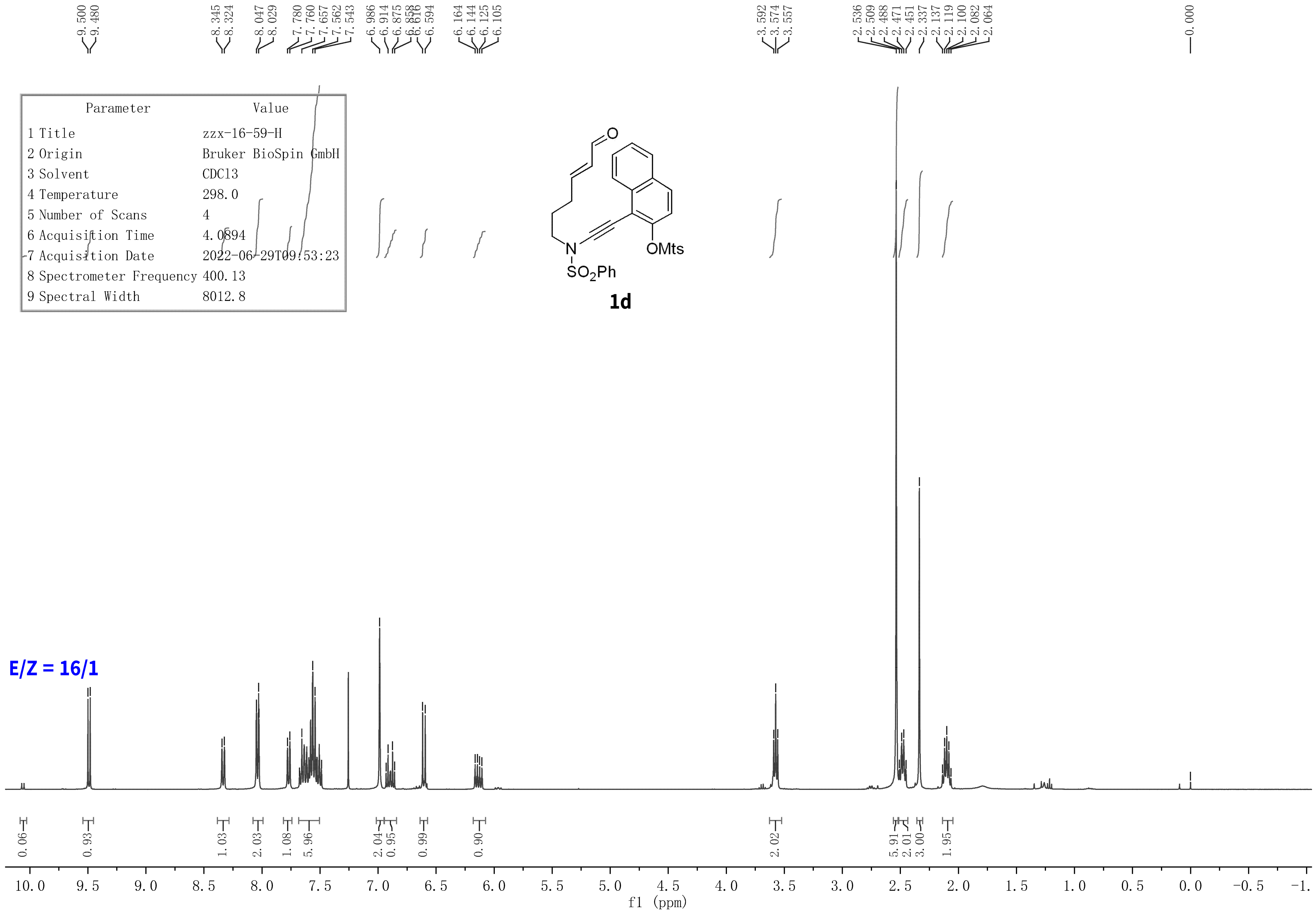
9.500
9.480
8.345
8.324
8.047
8.029
7.780
7.760
7.657
7.562
7.543
6.986
6.914
6.875
6.858
6.616
6.594
6.164
6.144
6.125
6.105
3.592
3.574
3.557
2.536
2.509
2.488
2.471
2.451
2.337
2.137
2.119
2.100
2.082
2.064
0.000
Parameter Value
1 Title zzx-16-59-H
2 Origin Bruker BioSpin GmbH
3 Solvent CDCl3
4 Temperature 298.0
5 Number of Scans 4
6 Acquisition Time 4.0894
7 Acquisition Date 2022-06-29T09:53:23
8 Spectrometer Frequency 400.13
9 Spectral Width 8012.8
O
N
SO2Ph
OMts
1d
E/Z = 16/1
0.06
0.93
1.03
2.03
1.08
5.96
2.04
0.95
0.99
0.90
2.02
5.91
2.01
3.00
1.95
f1 (ppm)

Parameter	Value
1 Title	zzx-16-59-C
2 Origin	Bruker BioSpin GmbH
3 Solvent	CDCl3
4 Temperature	300.0
5 Number of Scans	16
6 Acquisition Time	1.3631
7 Acquisition Date	2022-06-29T09:54:24
8 Spectrometer Frequency	100.61
9 Spectral Width	24038.5

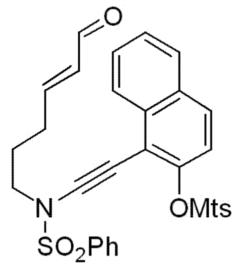

1d

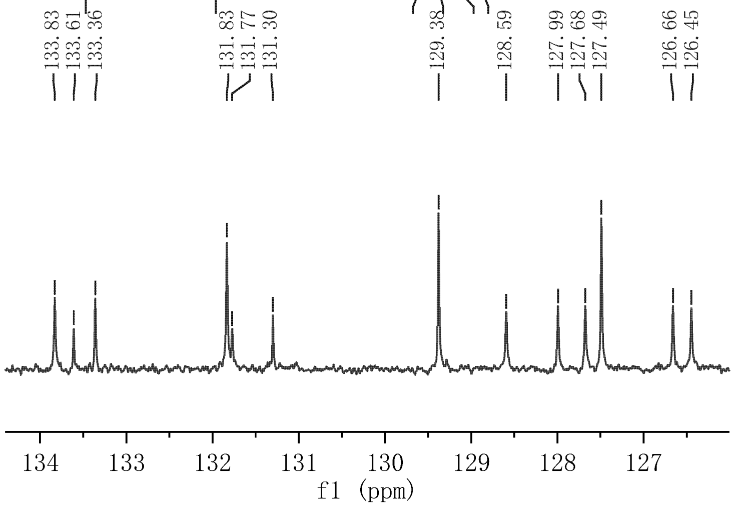

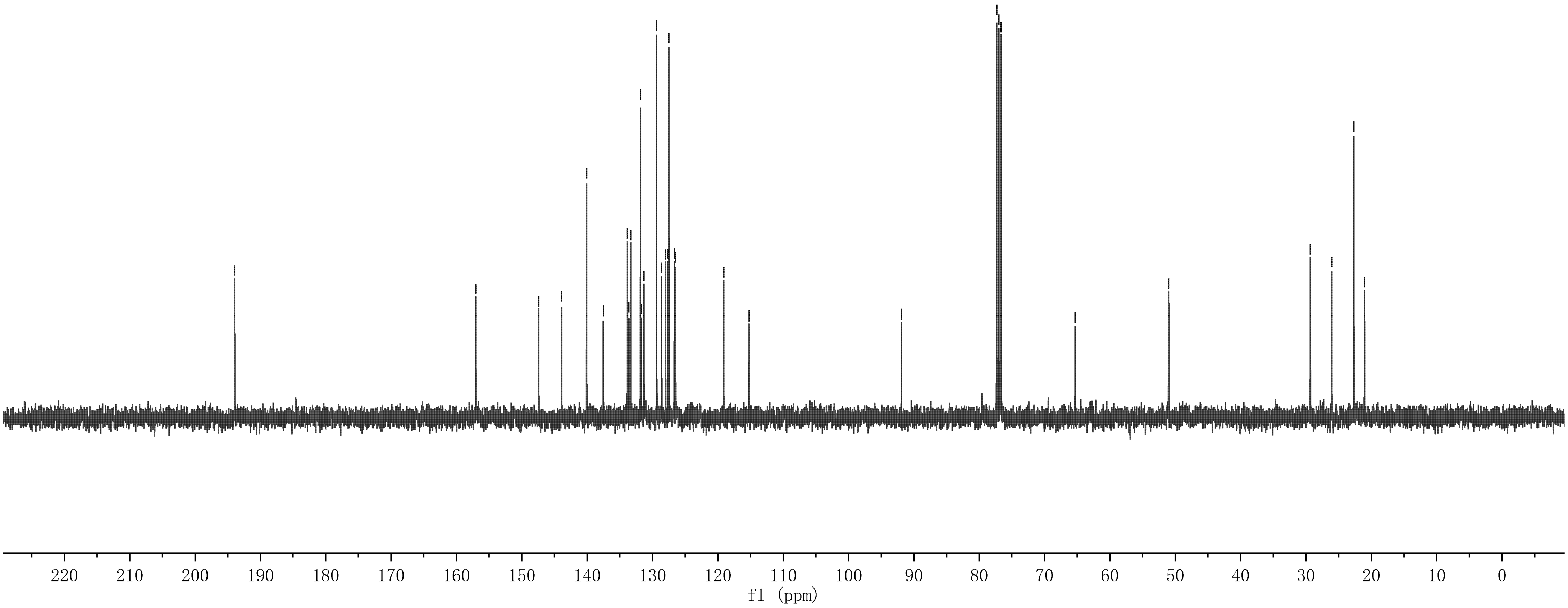

Parameter	Value
1 Title	ZZX-10-191
2 Origin	
3 Solvent	CDCl3
4 Temperature	296.9
5 Number of Scans	16
6 Acquisition Time	4.0002
7 Acquisition Date	2022-03-18T17:44:30
8 Spectrometer Frequency	399.93
9 Spectral Width	8012.0

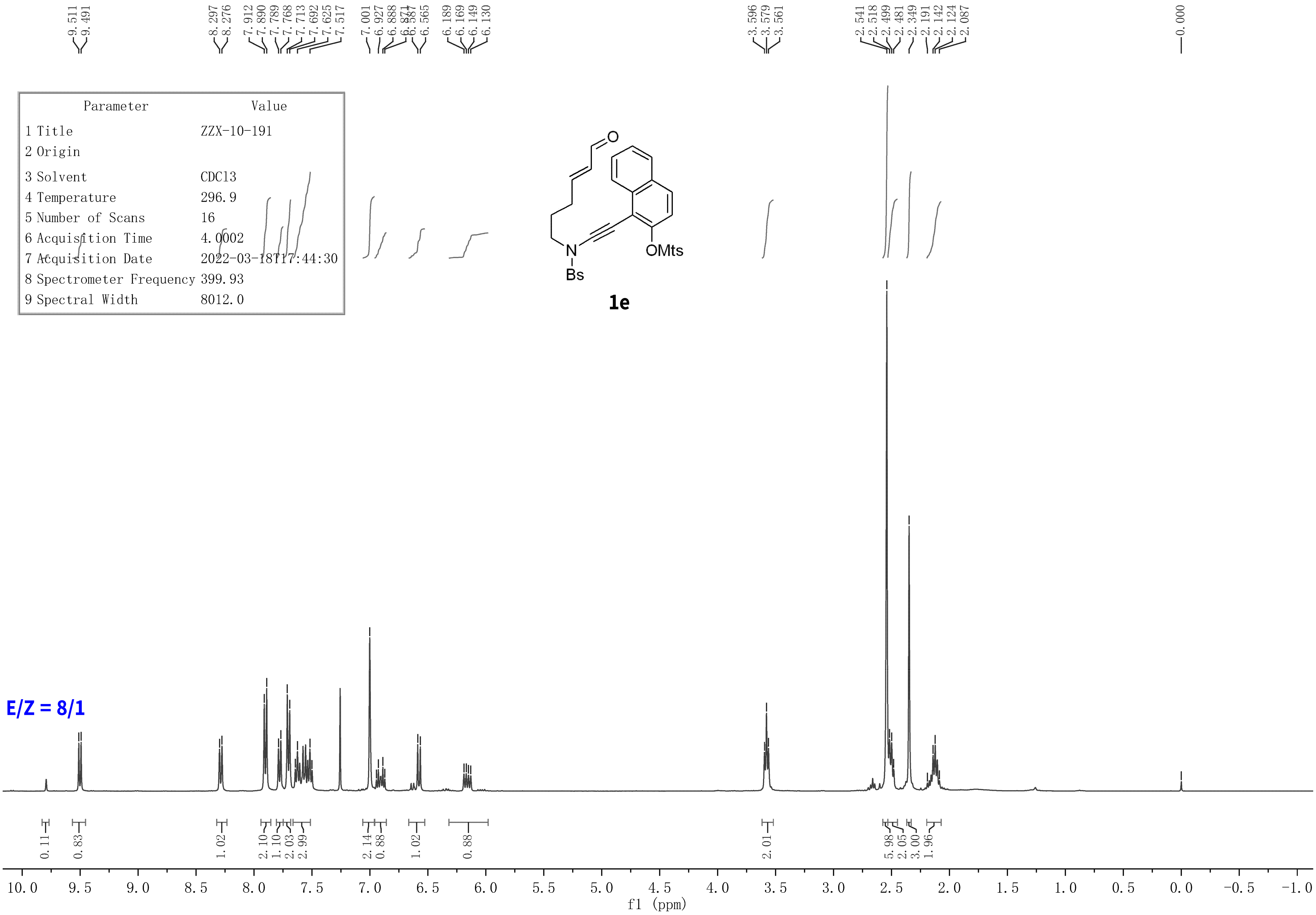

Parameter	Value
1 Title	ZZX-10-191
2 Origin	
3 Solvent	CDCl3
4 Temperature	297.3
5 Number of Scans	200
6 Acquisition Time	1.0000
7 Acquisition Date	2022-03-18T17:51:44
8 Spectrometer Frequency	100.56
9 Spectral Width	26041.0

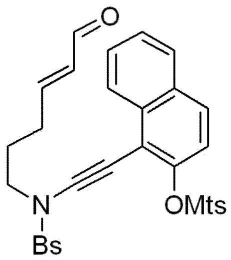

1e

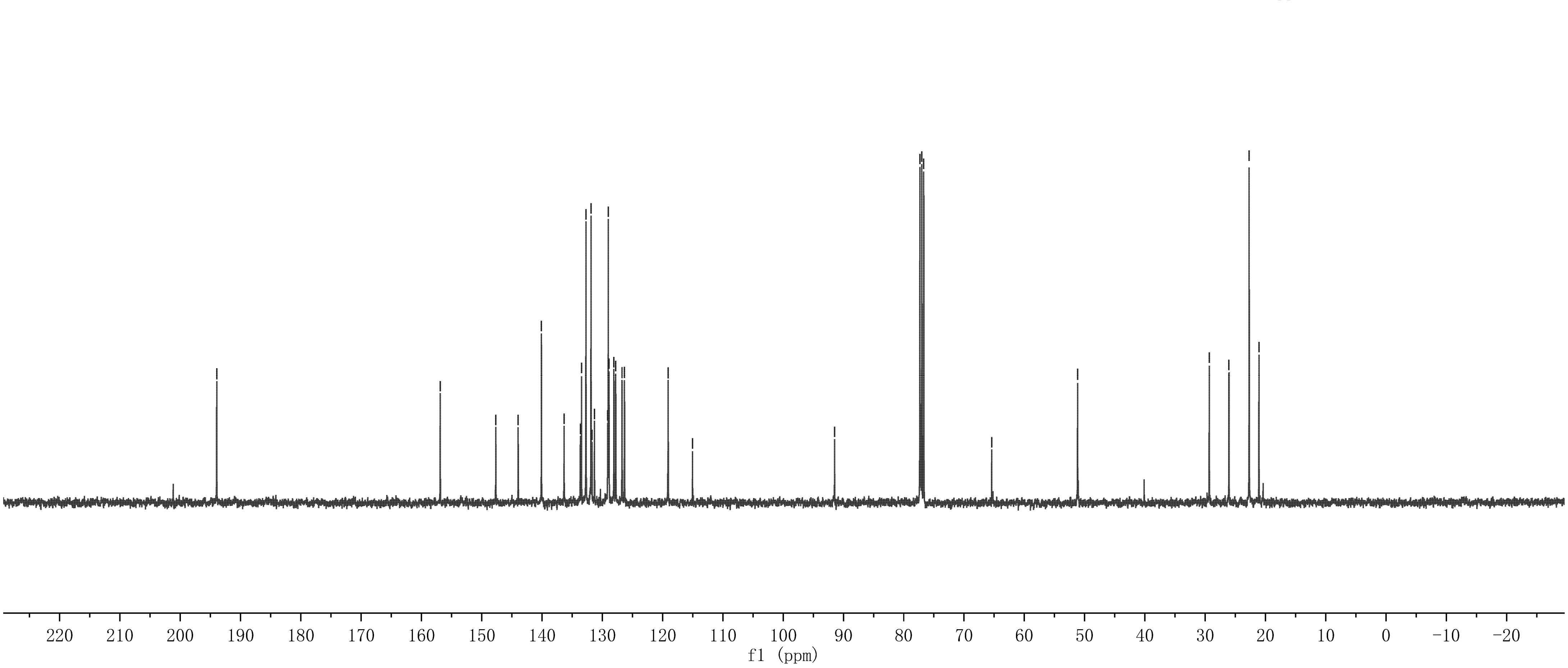

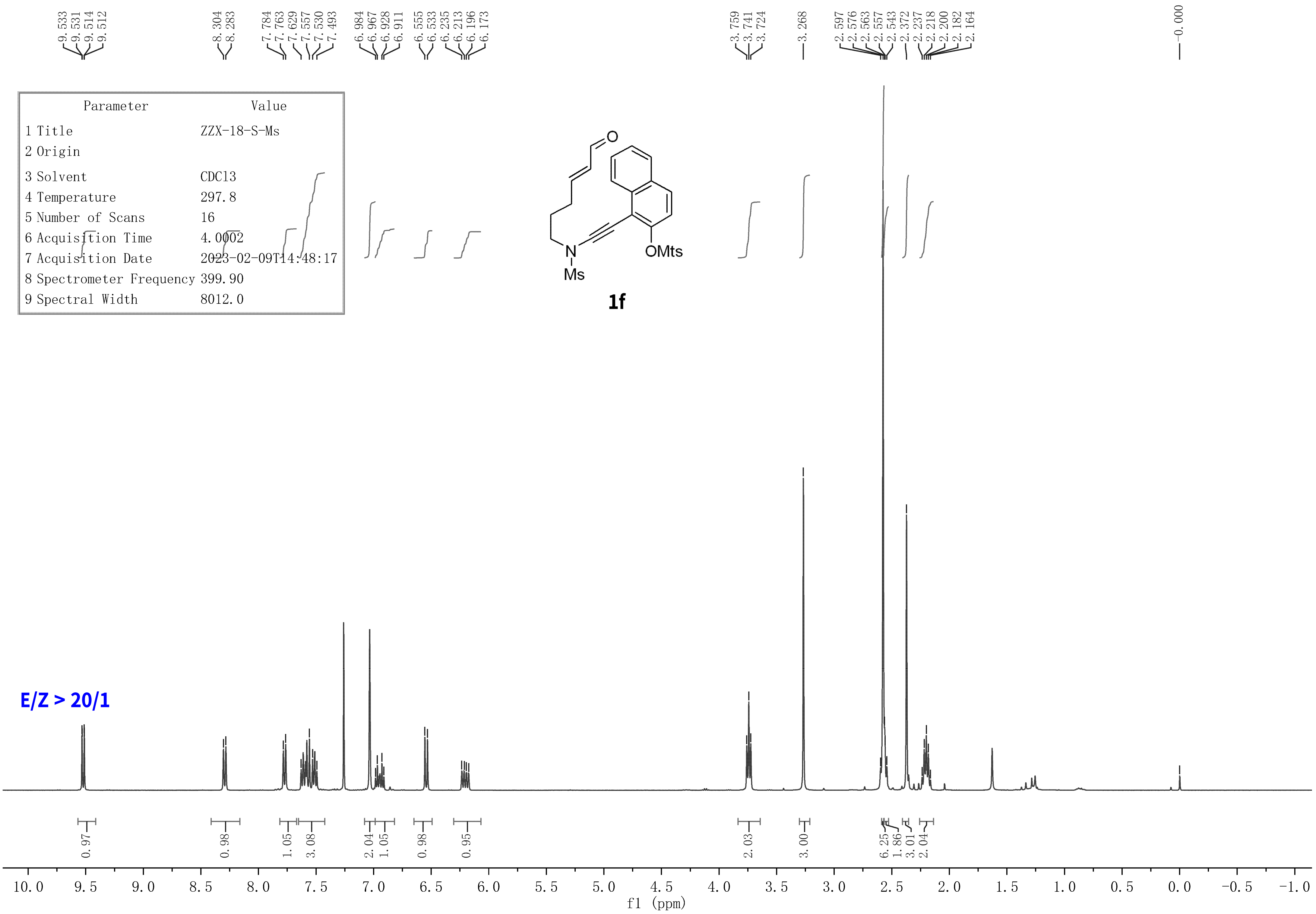

9.533 9.531 9.514 9.512
8.304 8.283
7.784 7.763 7.629 7.557 7.530 7.493
6.984 6.967 6.928 6.911
6.555 6.533 6.235 6.213 6.196 6.173
3.759 3.741 3.724
3.268
2.597 2.576 2.563 2.557 2.543 2.372 2.237 2.218 2.200 2.182 2.164
-0.000
Parameter Value
1 Title ZZX-18-S-Ms
2 Origin
3 Solvent CDCl3
4 Temperature 297.8
5 Number of Scans 16
6 Acquisition Time 4.0002
7 Acquisition Date 2023-02-09T14:48:17
8 Spectrometer Frequency 399.90
9 Spectral Width 8012.0
O
N
Ms
OMts
1f
E/Z > 20/1
0.97 0.98 1.05 3.08 2.04 1.05 0.98 0.95 2.03 3.00 6.25 1.86 3.01 2.04
10.0 9.5 9.0 8.5 8.0 7.5 7.0 6.5 6.0 5.5 5.0 4.5 4.0 3.5 3.0 2.5 2.0 1.5 1.0 0.5 0.0 -0.5 -1.0
f1 (ppm)

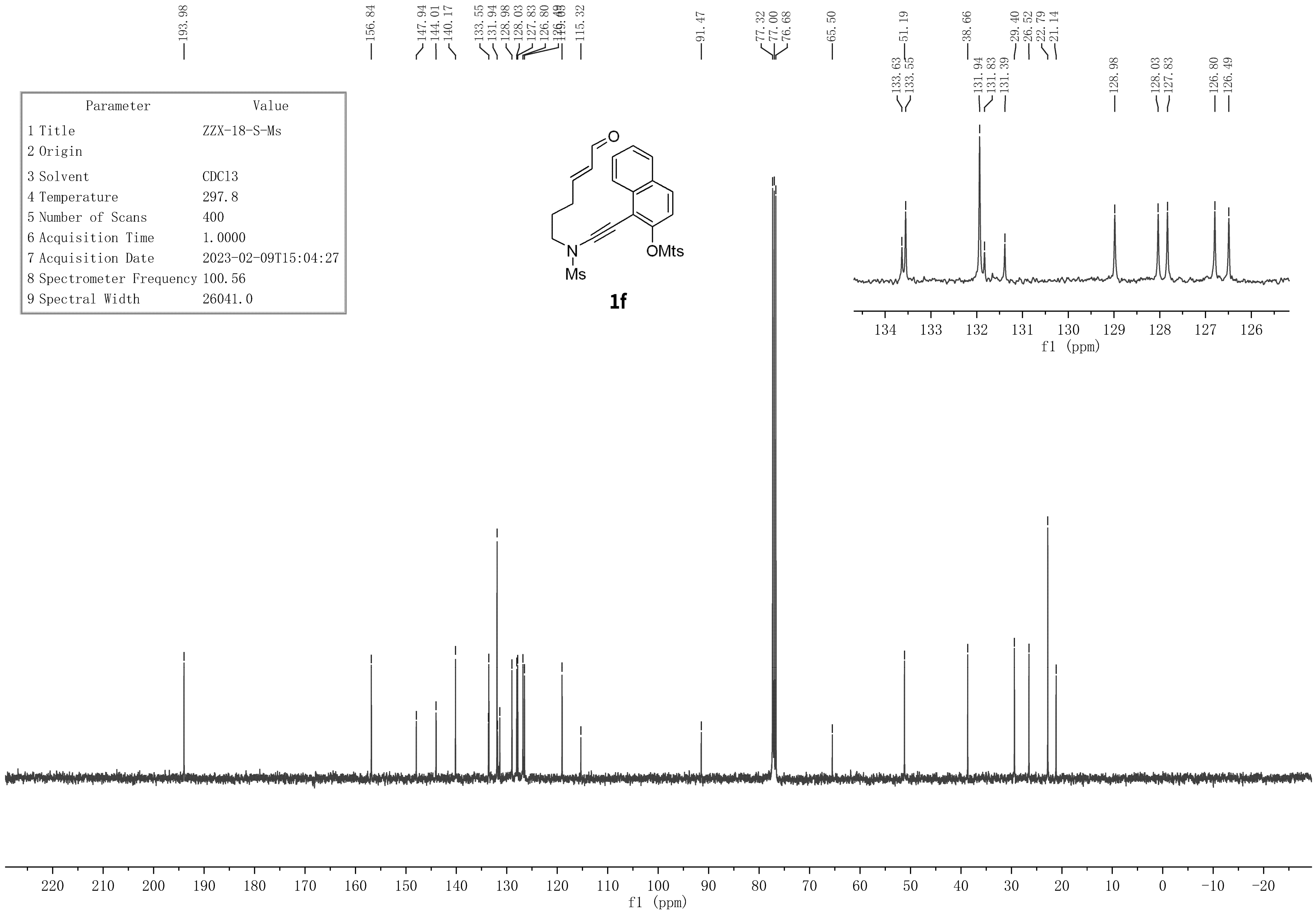

Parameter	Value
1 Title	ZZX-18-S-Ms
2 Origin	
3 Solvent	CDCl3
4 Temperature	297.8
5 Number of Scans	400
6 Acquisition Time	1.0000
7 Acquisition Date	2023-02-09T15:04:27
8 Spectrometer Frequency	100.56
9 Spectral Width	26041.0

1f

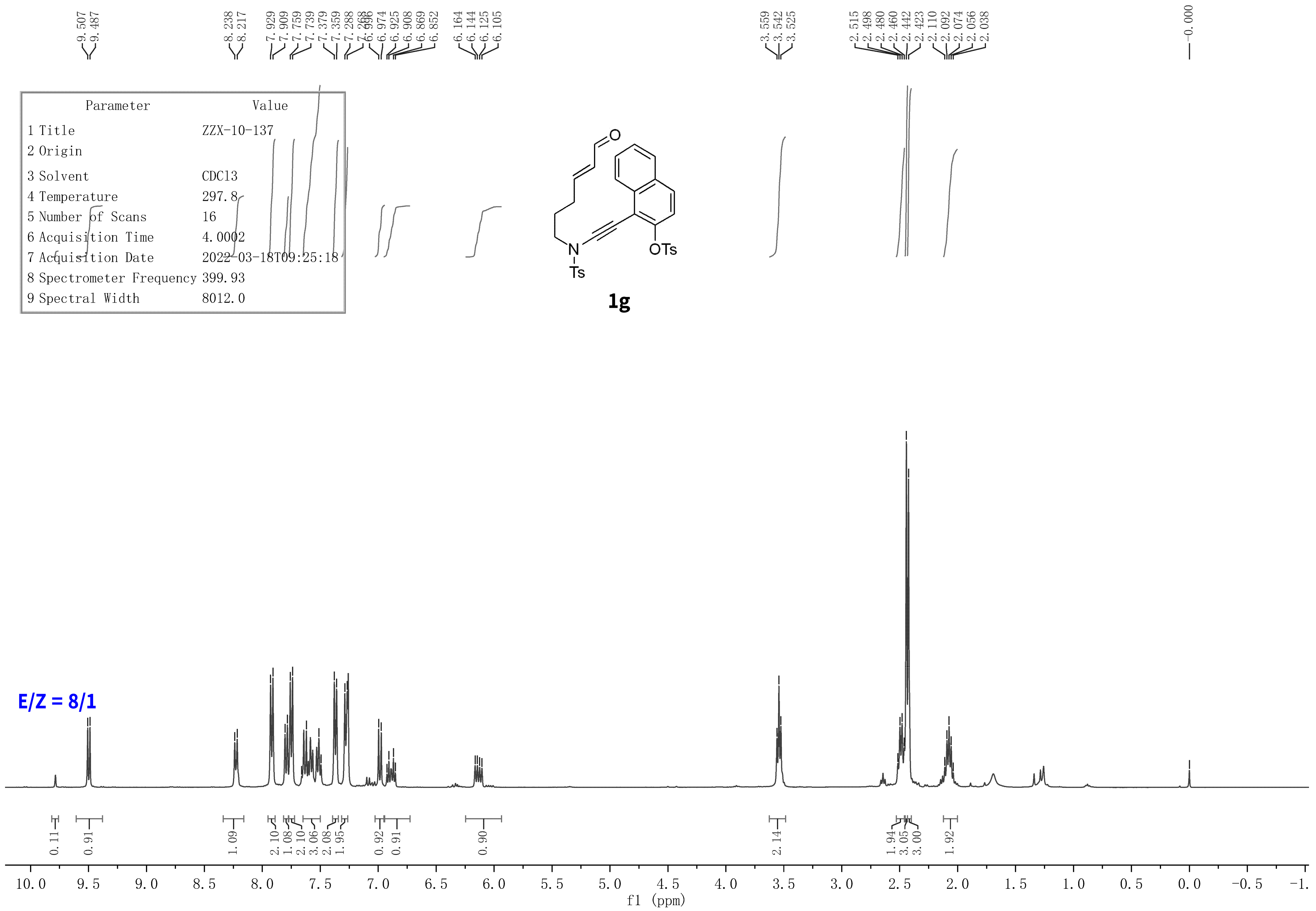

9.507
9.487
8.238
8.217
7.929
7.909
7.759
7.739
7.379
7.359
7.288
6.974
6.925
6.908
6.869
6.852
6.164
6.144
6.125
6.105
3.559
3.542
3.525
2.515
2.498
2.480
2.460
2.442
2.423
2.110
2.092
2.074
2.056
2.038
-0.000
Parameter Value
1 Title ZZX-10-137
2 Origin
3 Solvent CDCl3
4 Temperature 297.8
5 Number of Scans 16
6 Acquisition Time 4.0002
7 Acquisition Date 2022-03-18T09:25:18
8 Spectrometer Frequency 399.93
9 Spectral Width 8012.0
O
N
Ts
OTs
1g
E/Z = 8/1
0.11
0.91
1.09
2.10
1.08
2.10
3.06
2.08
1.95
0.92
0.91
0.90
2.14
1.94
3.05
3.00
1.92
10.0 9.5 9.0 8.5 8.0 7.5 7.0 6.5 6.0 5.5 5.0 4.5 4.0 3.5 3.0 2.5 2.0 1.5 1.0 0.5 0.0 -0.5 -1.
f1 (ppm)

Parameter	Value
1 Title	ZZX-10-137
2 Origin	
3 Solvent	CDCl3
4 Temperature	298.0
5 Number of Scans	200
6 Acquisition Time	1.0000
7 Acquisition Date	2022-03-18T09:32:32
8 Spectrometer Frequency	100.56
9 Spectral Width	26041.0

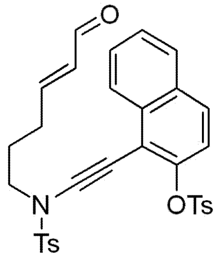

1g

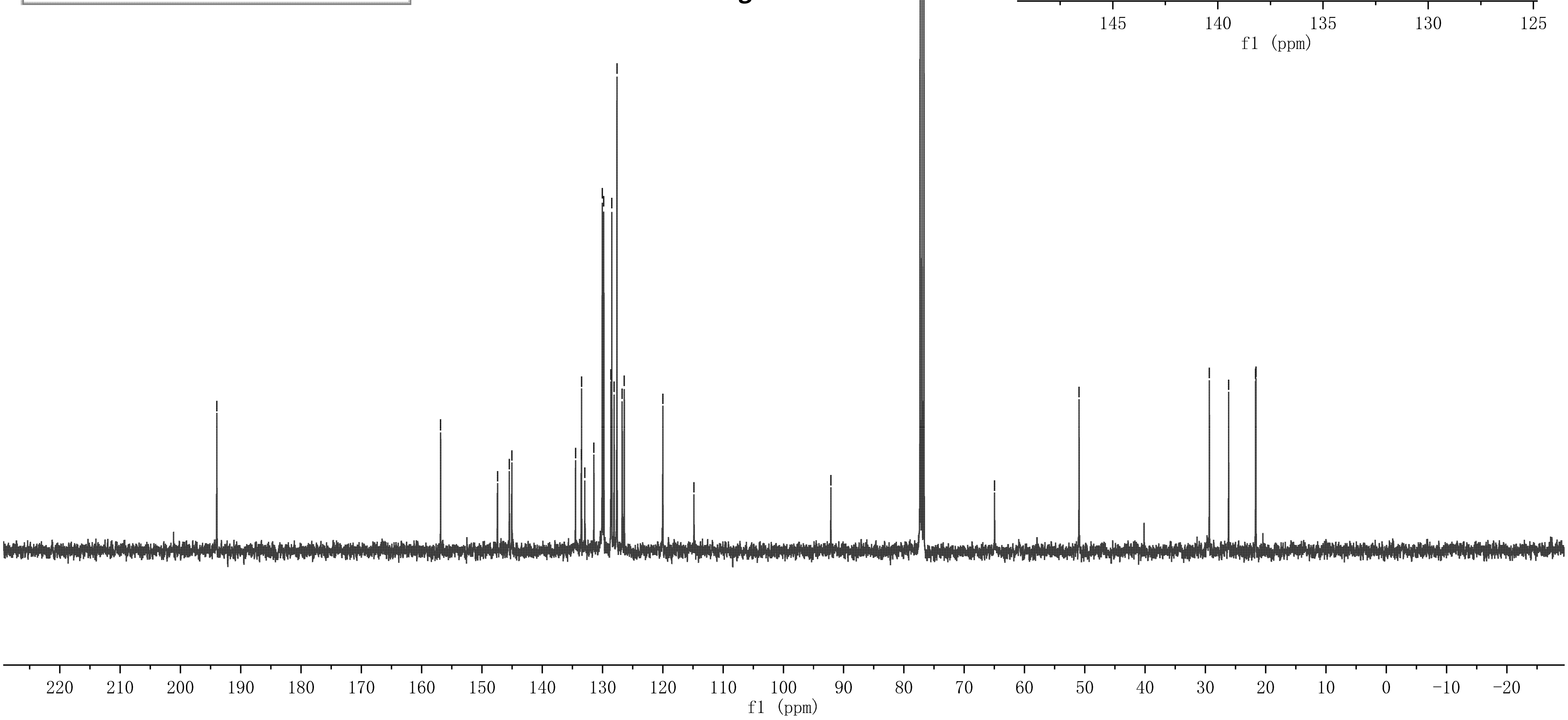

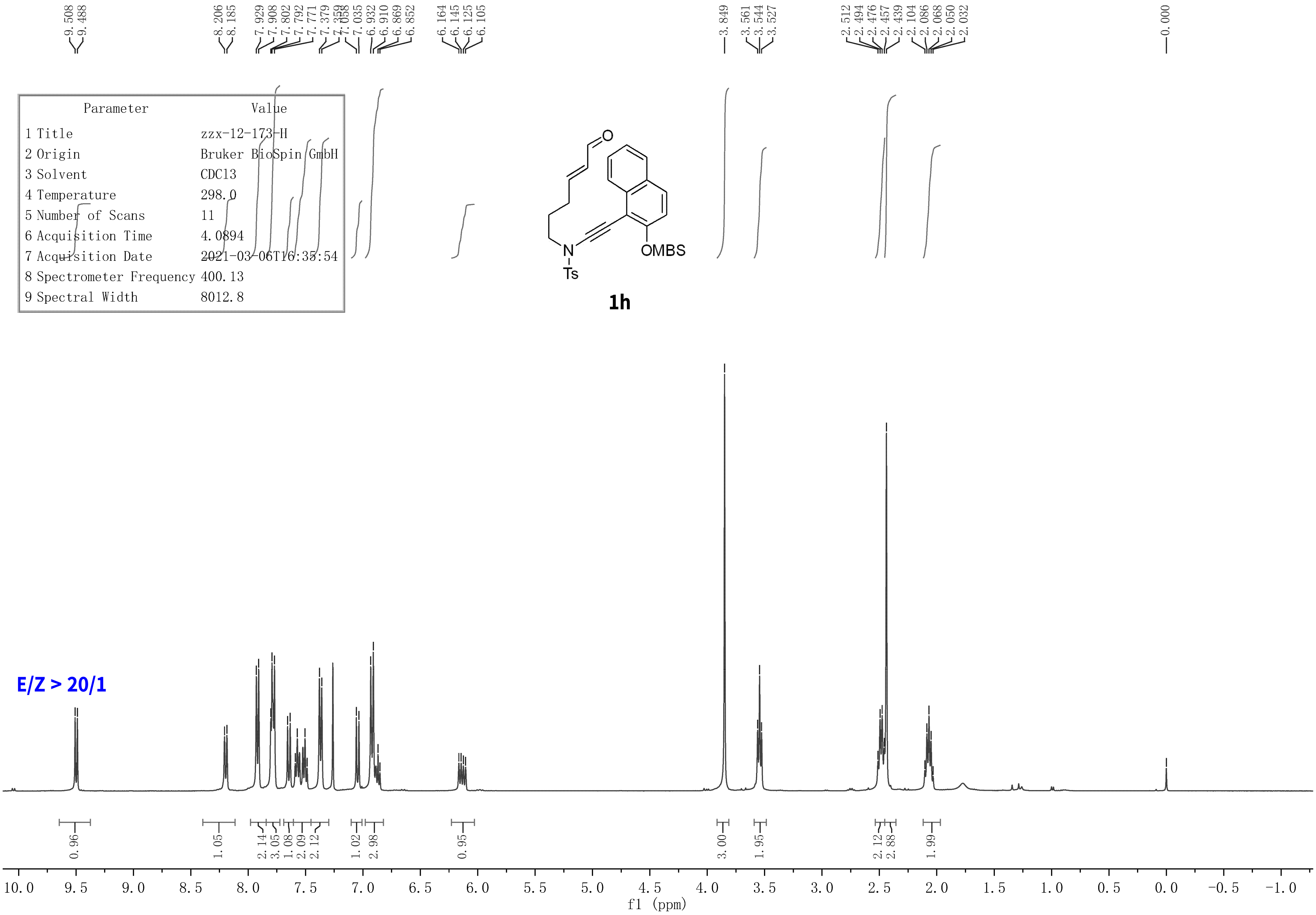
9.508
9.488
8.206
8.185
7.929
7.908
7.802
7.792
7.771
7.379
7.359
7.358
7.035
6.932
6.910
6.869
6.852
6.164
6.145
6.125
6.105
3.849
3.561
3.544
3.527
2.512
2.494
2.476
2.457
2.439
2.104
2.086
2.068
2.050
2.032
0.000
Parameter Value
1 Title zzx-12-173-H
2 Origin Bruker BioSpin GmbH
3 Solvent CDCl3
4 Temperature 298.0
5 Number of Scans 11
6 Acquisition Time 4.0894
7 Acquisition Date 2021-03-06T16:35:54
8 Spectrometer Frequency 400.13
9 Spectral Width 8012.8
O
N
Ts
OMBS
1h
E/Z > 20/1
0.96
1.05
2.14
3.05
1.08
2.09
2.12
1.02
2.98
0.95
3.00
1.95
2.12
2.88
1.99
10.0 9.5 9.0 8.5 8.0 7.5 7.0 6.5 6.0 5.5 5.0 4.5 4.0 3.5 3.0 2.5 2.0 1.5 1.0 0.5 0.0 -0.5 -1.0
f1 (ppm)

Parameter	Value
1 Title	zzx-12-173-C
2 Origin	Bruker BioSpin GmbH
3 Solvent	CDCl3
4 Temperature	300.0
5 Number of Scans	35
6 Acquisition Time	1.3631
7 Acquisition Date	2021-03-06T16:37:35
8 Spectrometer Frequency	100.61
9 Spectral Width	24038.5

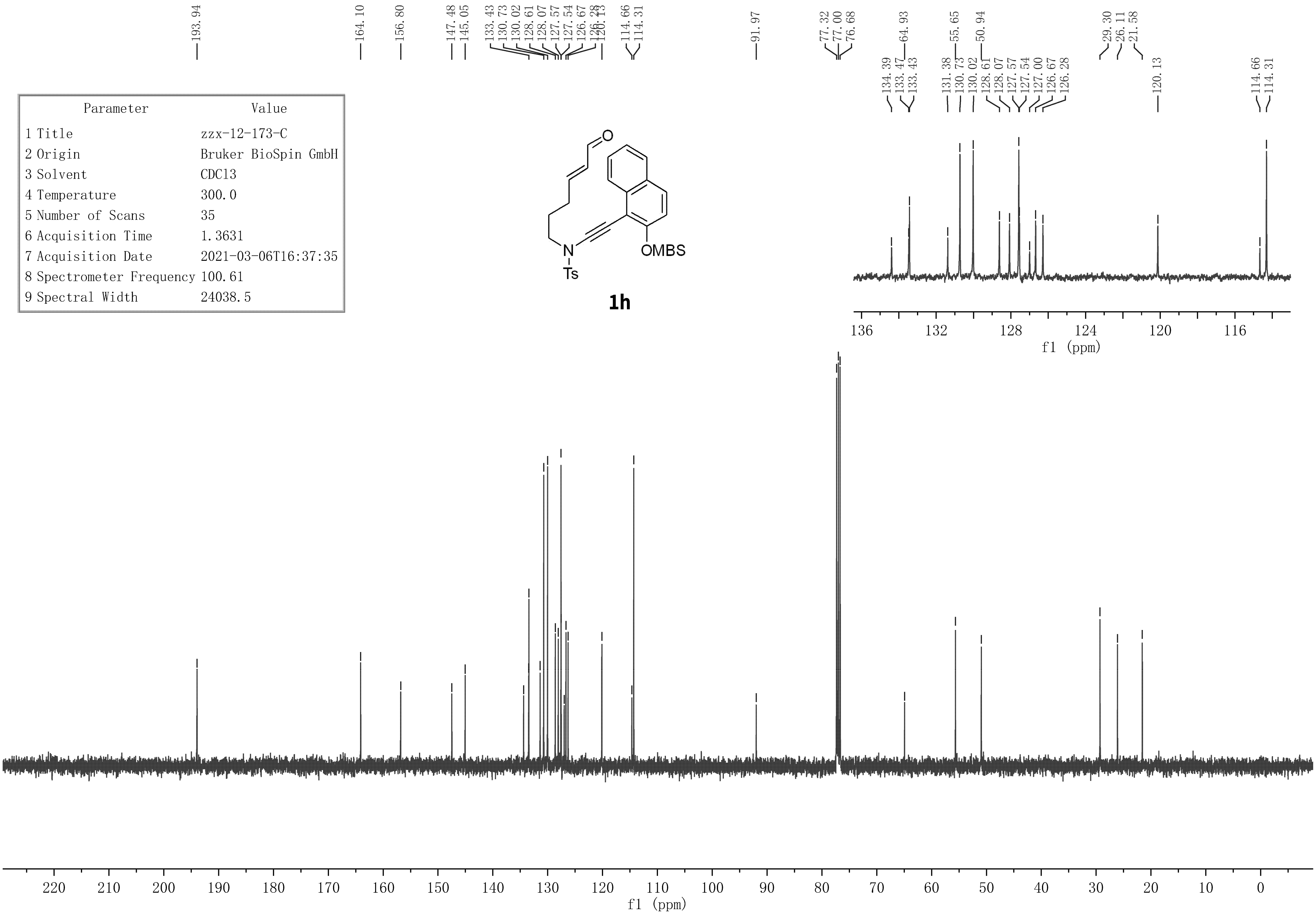

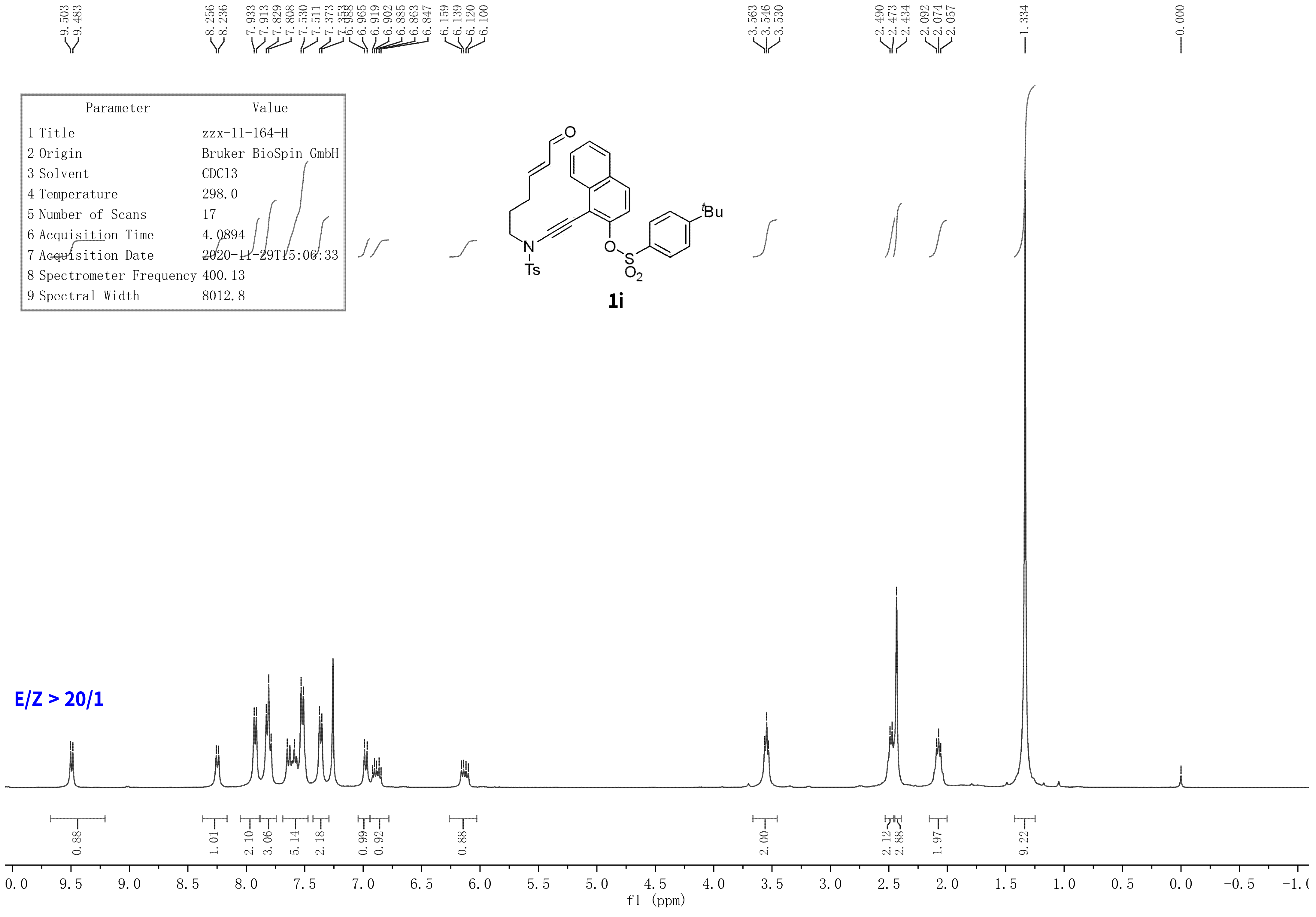

9.503
9.483
8.256
8.236
7.933
7.913
7.829
7.808
7.530
7.511
7.373
7.353
6.988
6.965
6.919
6.902
6.885
6.863
6.847
6.159
6.139
6.120
6.100
3.563
3.546
3.530
2.490
2.473
2.434
2.092
2.074
2.057
1.334
0.000
Parameter Value
1 Title zzx-11-164-H
2 Origin Bruker BioSpin GmbH
3 Solvent CDCl3
4 Temperature 298.0
5 Number of Scans 17
6 Acquisition Time 4.0894
7 Acquisition Date 2020-11-29T15:06:33
8 Spectrometer Frequency 400.13
9 Spectral Width 8012.8
O
N
Ts
O
S
O2
tBu
1i
E/Z > 20/1
0.88
1.01
2.10
3.06
5.14
2.18
0.99
0.92
0.88
2.00
2.12
2.88
1.97
9.22
f1 (ppm)

Parameter	Value
1 Title	zzx-11-164-C
2 Origin	Bruker BioSpin GmbH
3 Solvent	CDCl3
4 Temperature	300.0
5 Number of Scans	64
6 Acquisition Time	1.3631
7 Acquisition Date	2020-11-29T15:09:28
8 Spectrometer Frequency	100.61
9 Spectral Width	24038.5

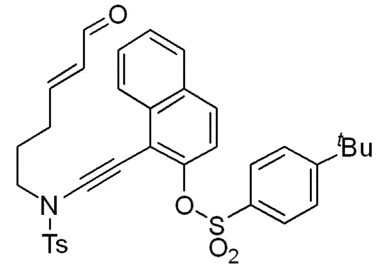

1i

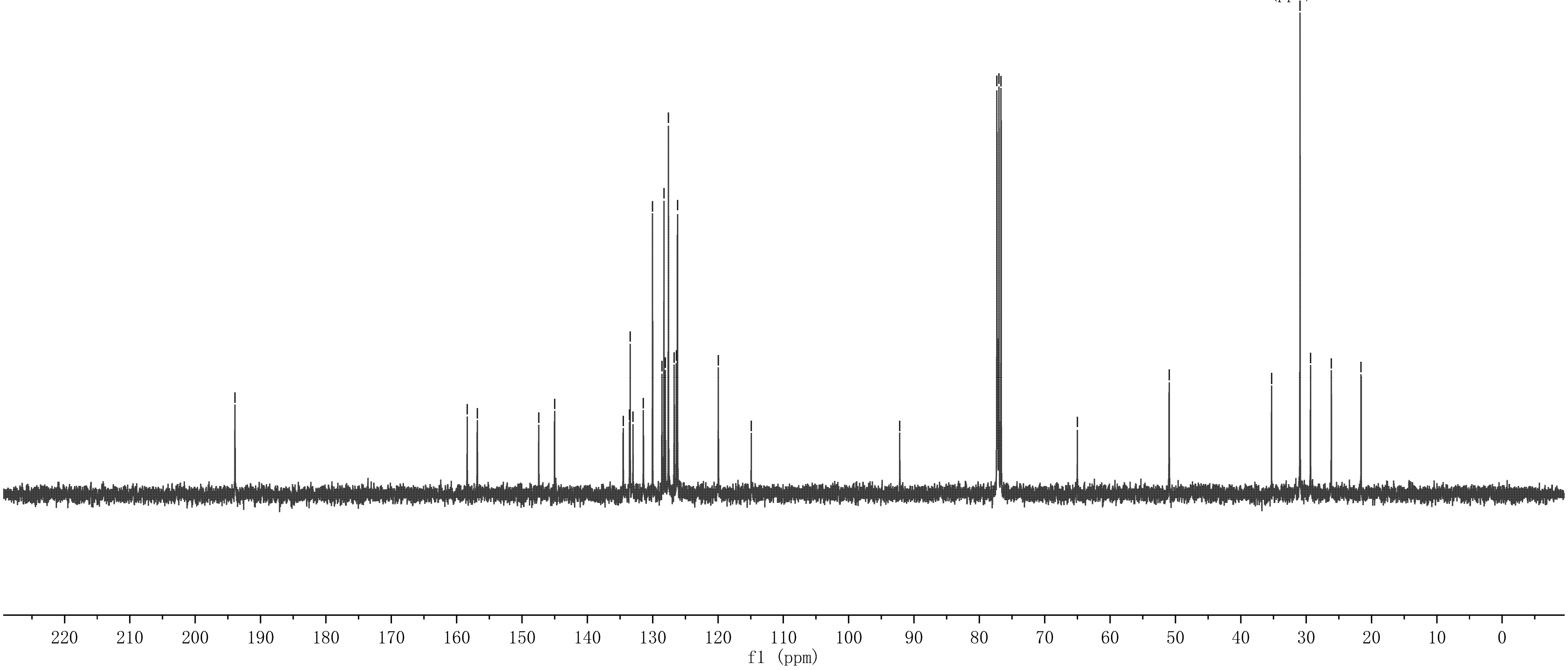

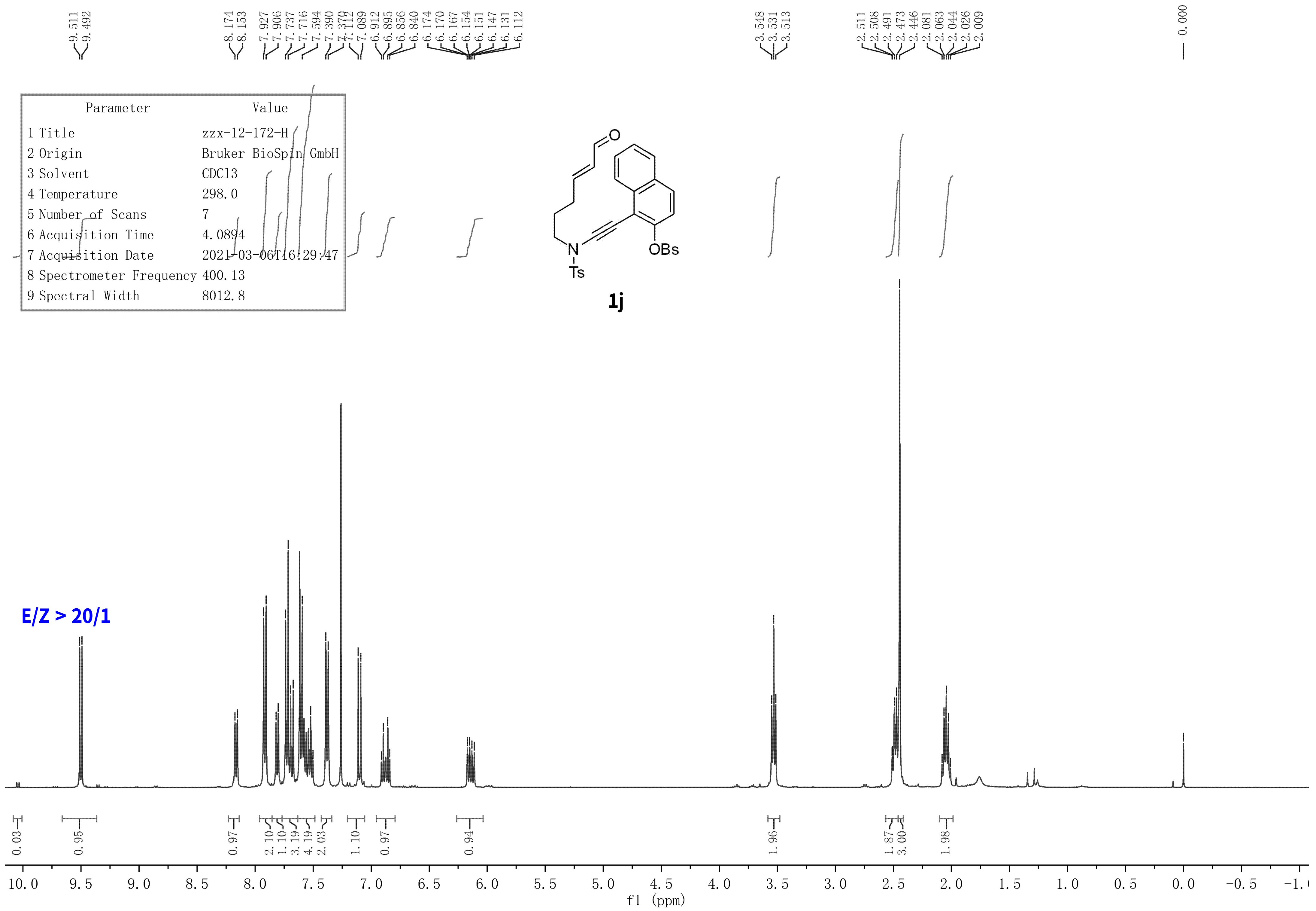

Parameter	Value
1 Title	zzx-12-172-H
2 Origin	Bruker BioSpin GmbH
3 Solvent	CDCl3
4 Temperature	298.0
5 Number of Scans	7
6 Acquisition Time	4.0894
7 Acquisition Date	2021-03-06T16:29:47
8 Spectrometer Frequency	400.13
9 Spectral Width	8012.8

Parameter	Value
1 Title	zzx-12-172-C
2 Origin	Bruker BioSpin GmbH
3 Solvent	CDCl3
4 Temperature	300.0
5 Number of Scans	22
6 Acquisition Time	1.3631
7 Acquisition Date	2021-03-06T16:31:03
8 Spectrometer Frequency	100.61
9 Spectral Width	24038.5

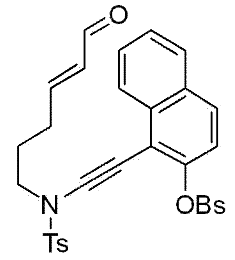

1j

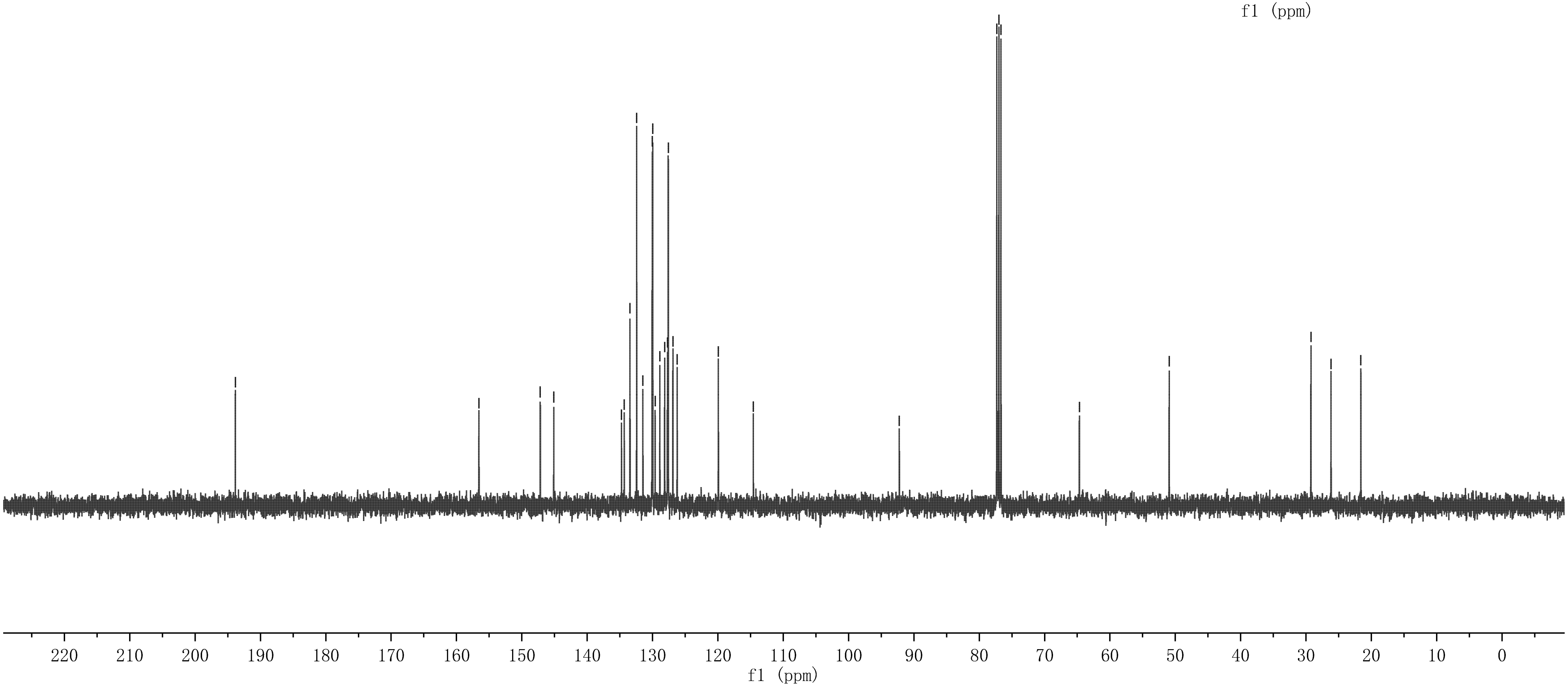

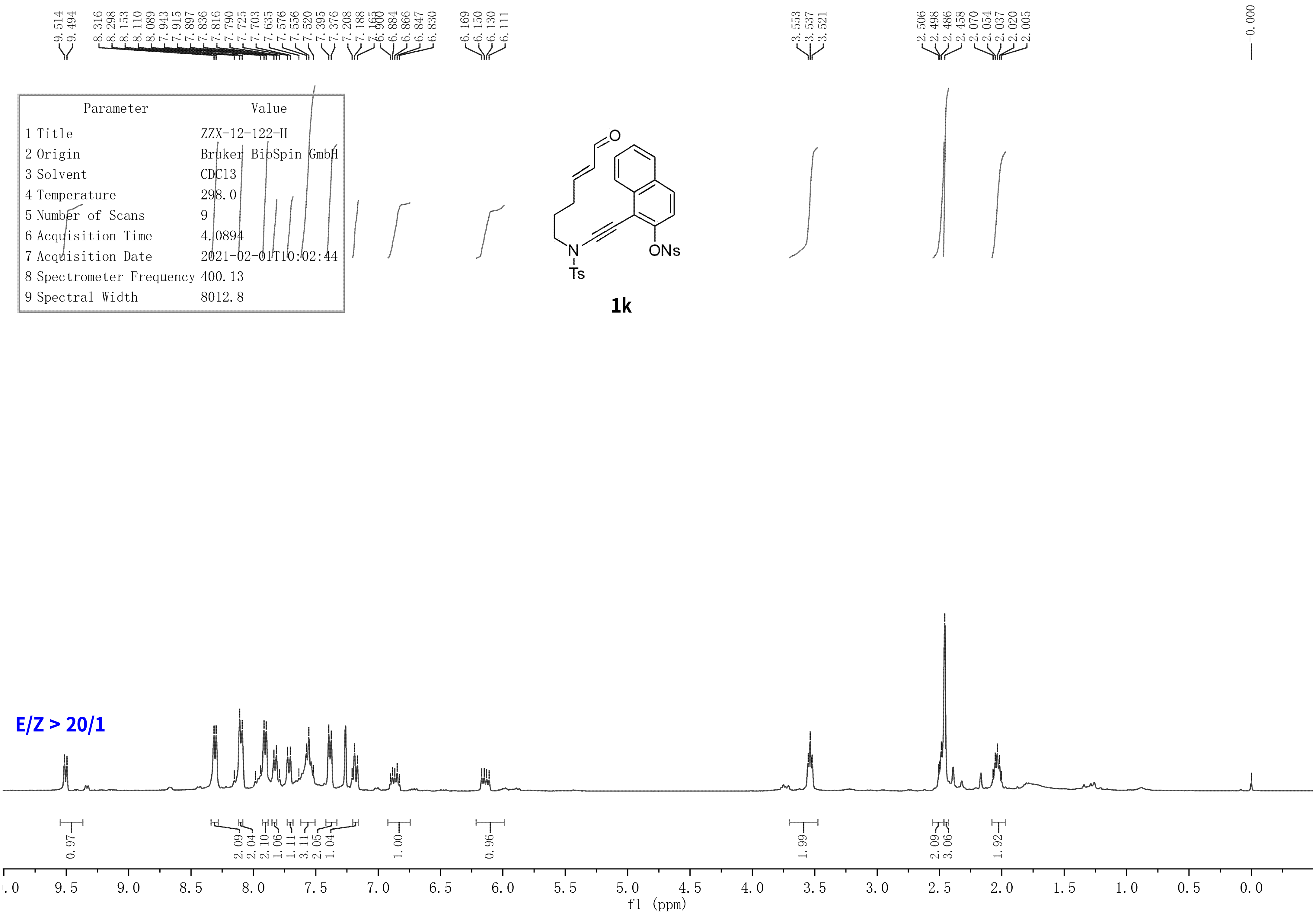

9.514
9.494
8.316
8.298
8.153
8.110
8.089
7.943
7.915
7.897
7.836
7.816
7.790
7.725
7.703
7.635
7.576
7.556
7.520
7.395
7.376
7.208
7.188
7.165
6.900
6.884
6.866
6.847
6.830
6.169
6.150
6.130
6.111
3.553
3.537
3.521
2.506
2.498
2.486
2.458
2.070
2.054
2.037
2.020
2.005
-0.000
Parameter Value
1 Title ZZX-12-122-H
2 Origin Bruker BioSpin GmbH
3 Solvent CDCl3
4 Temperature 298.0
5 Number of Scans 9
6 Acquisition Time 4.0894
7 Acquisition Date 2021-02-01T10:02:44
8 Spectrometer Frequency 400.13
9 Spectral Width 8012.8
O
N
Ts
ONs
1k
E/Z > 20/1
0.97
2.09
2.04
2.10
1.06
1.11
3.11
2.05
1.04
1.00
0.96
1.99
2.09
3.06
1.92
9.5
9.0
8.5
8.0
7.5
7.0
6.5
6.0
5.5
5.0
4.5
4.0
3.5
3.0
2.5
2.0
1.5
1.0
0.5
0.0
f1 (ppm)

	Parameter	Value
1	Title	ZZX-12-122-C
2	Origin	Bruker BioSpin GmbH
3	Solvent	CDCl3
4	Temperature	300.0
5	Number of Scans	134
6	Acquisition Time	1.3631
7	Acquisition Date	2021-02-01T10:06:38
8	Spectrometer Frequency	100.61
9	Spectral Width	24038.5

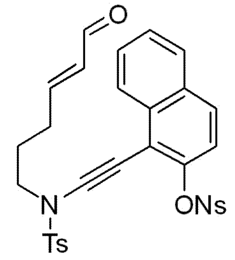

1k

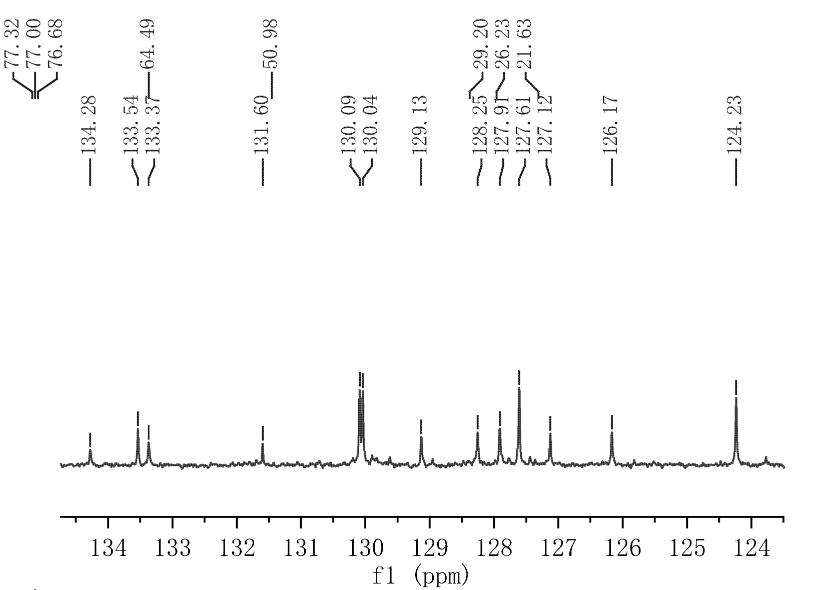

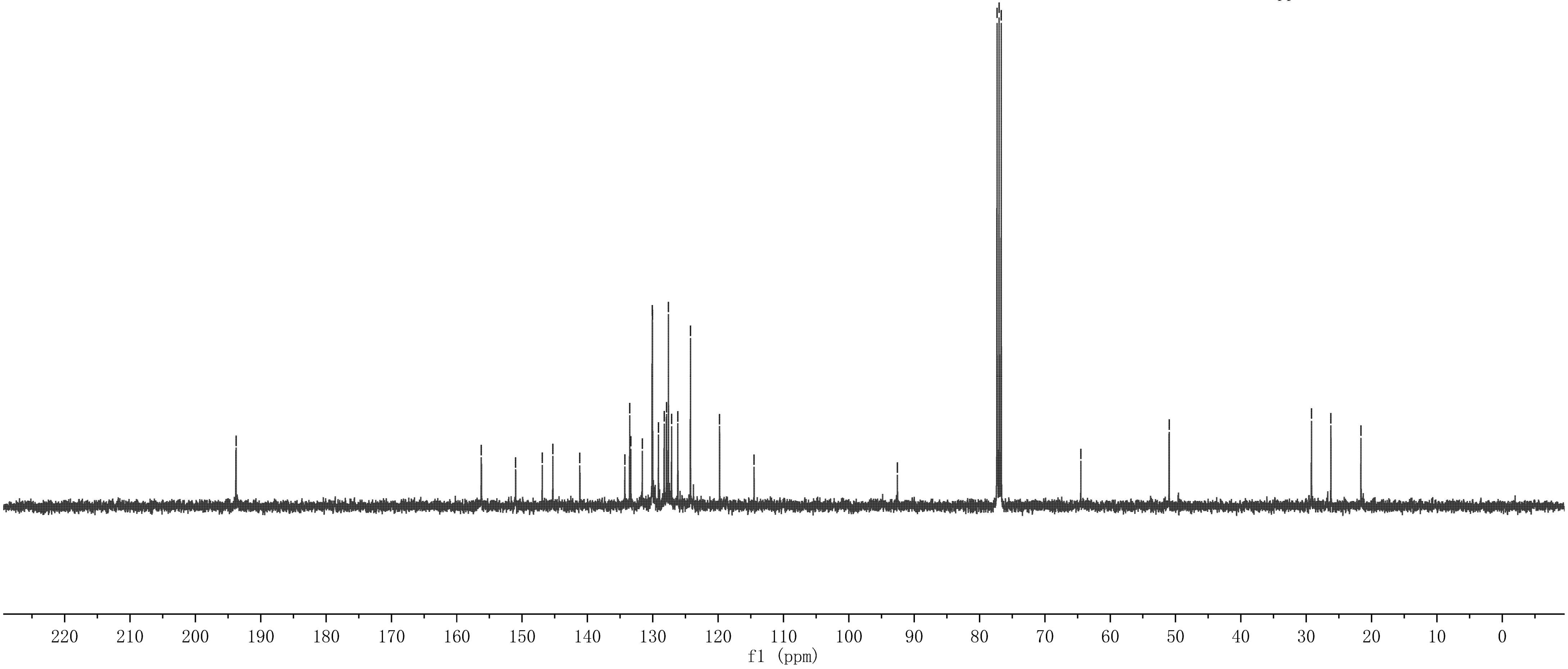

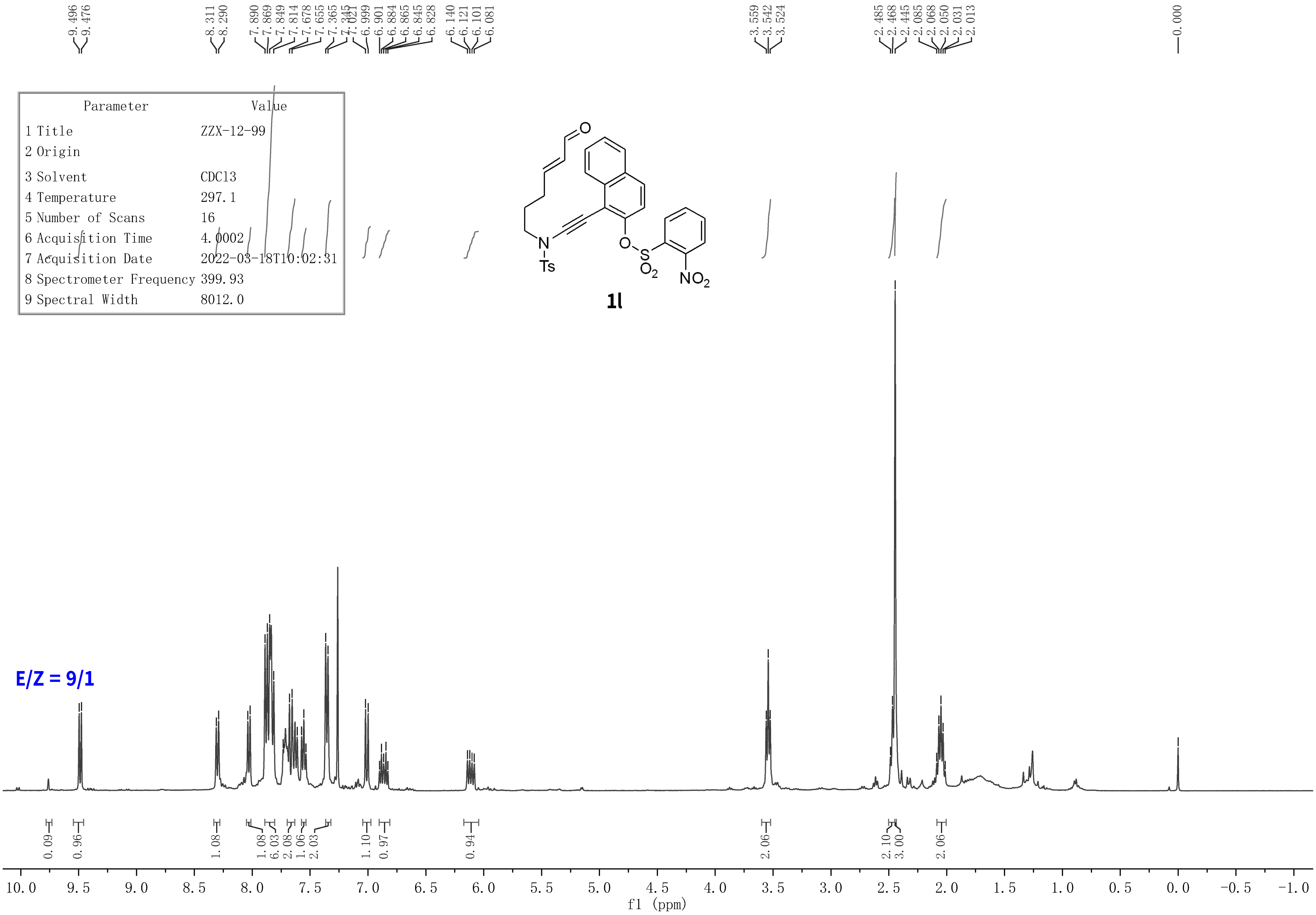
Parameter Value
1 Title ZZX-12-99
2 Origin
3 Solvent CDCl3
4 Temperature 297.1
5 Number of Scans 16
6 Acquisition Time 4.0002
7 Acquisition Date 2022-03-18T10:02:31
8 Spectrometer Frequency 399.93
9 Spectral Width 8012.0
1l
E/Z = 9/1
f1 (ppm)

Parameter	Value
1 Title	ZZX-12-99-C
2 Origin	
3 Solvent	CDCl3
4 Temperature	296.4
5 Number of Scans	1024
6 Acquisition Time	1.0000
7 Acquisition Date	2022-03-19T01:07:14
8 Spectrometer Frequency	100.56
9 Spectral Width	26041.0

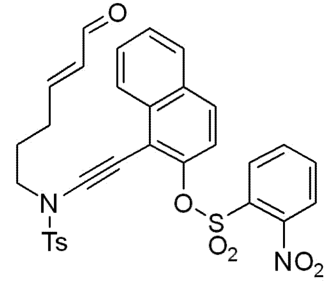

1l

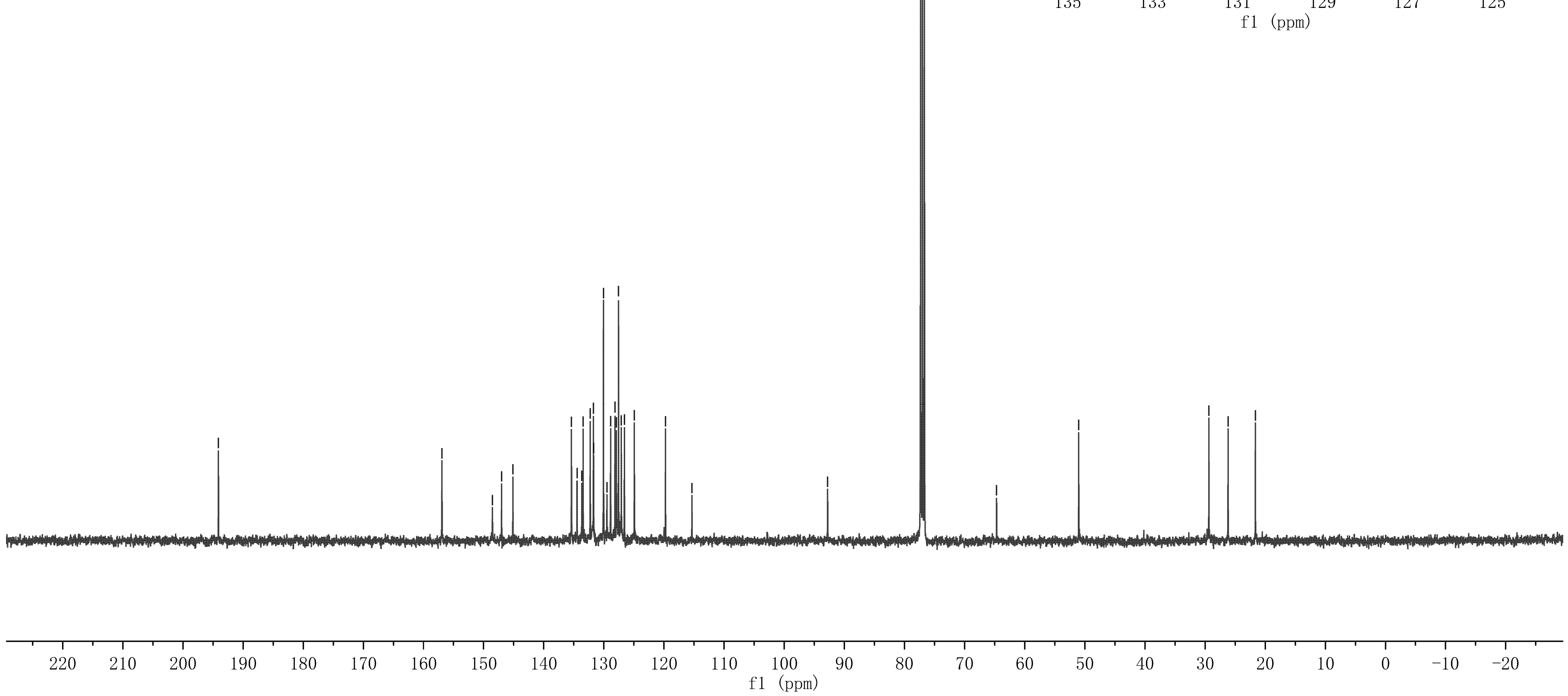

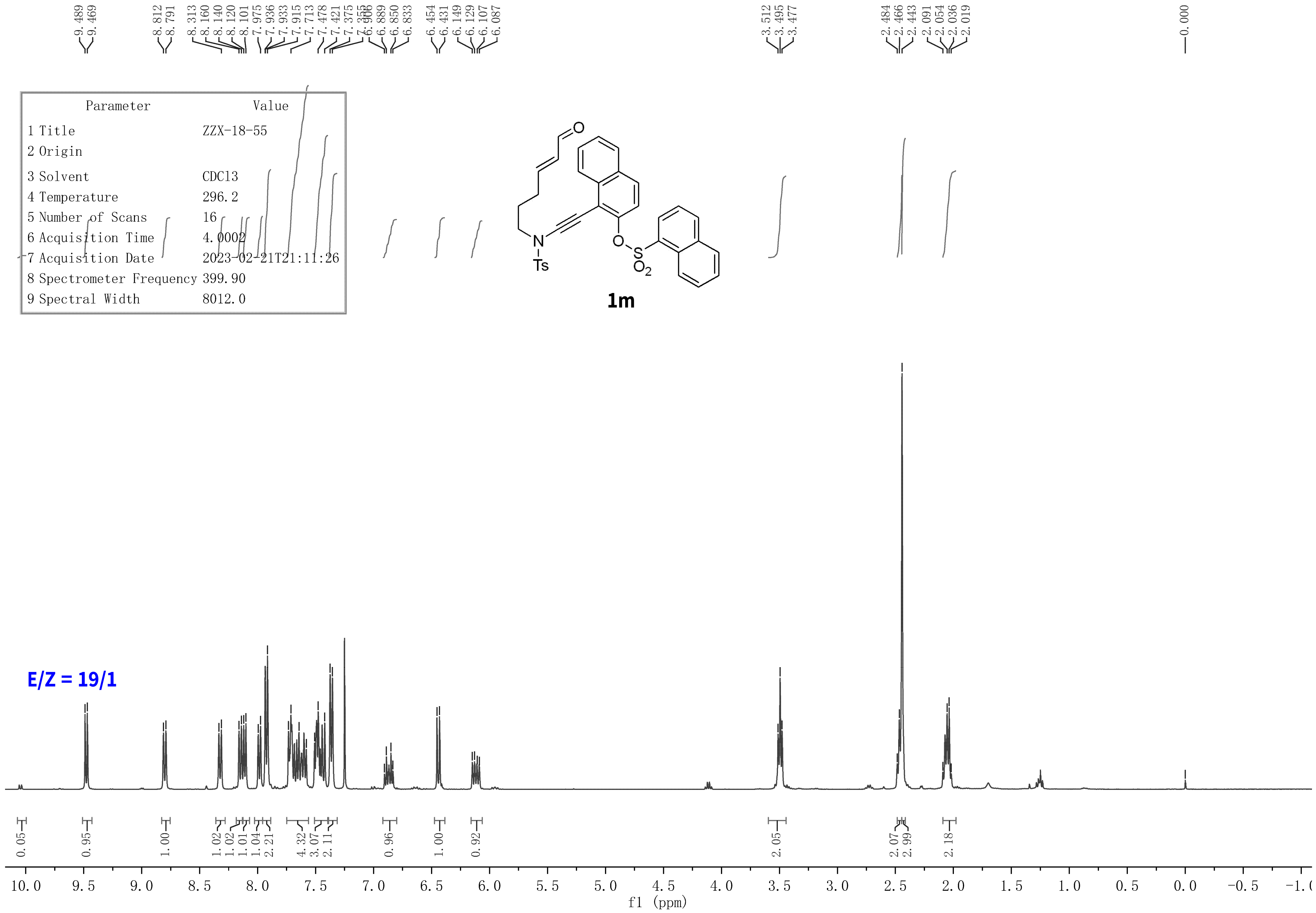

9.489
9.469
8.812
8.791
8.313
8.160
8.140
8.120
8.101
7.975
7.936
7.933
7.915
7.713
7.478
7.421
7.375
7.355
6.906
6.889
6.850
6.833
6.454
6.431
6.149
6.129
6.107
6.087
3.512
3.495
3.477
2.484
2.466
2.443
2.091
2.054
2.036
2.019
0.000
Parameter Value
1 Title ZZX-18-55
2 Origin
3 Solvent CDCl3
4 Temperature 296.2
5 Number of Scans 16
6 Acquisition Time 4.0002
7 Acquisition Date 2023-02-21T21:11:26
8 Spectrometer Frequency 399.90
9 Spectral Width 8012.0
O
N
Ts
O
S
O2
1m
E/Z = 19/1
0.05
0.95
1.00
1.02
1.02
1.01
1.04
2.21
4.32
3.07
2.11
0.96
1.00
0.92
2.05
2.07
2.99
2.18
10.0 9.5 9.0 8.5 8.0 7.5 7.0 6.5 6.0 5.5 5.0 4.5 4.0 3.5 3.0 2.5 2.0 1.5 1.0 0.5 0.0 -0.5
f1 (ppm)

Parameter	Value
1 Title	ZZX-18-55
2 Origin	
3 Solvent	CDCl3
4 Temperature	296.3
5 Number of Scans	500
6 Acquisition Time	1.0000
7 Acquisition Date	2023-02-21T21:30:39
8 Spectrometer Frequency	100.56
9 Spectral Width	26041.0

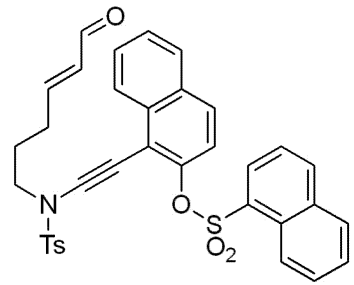

1m

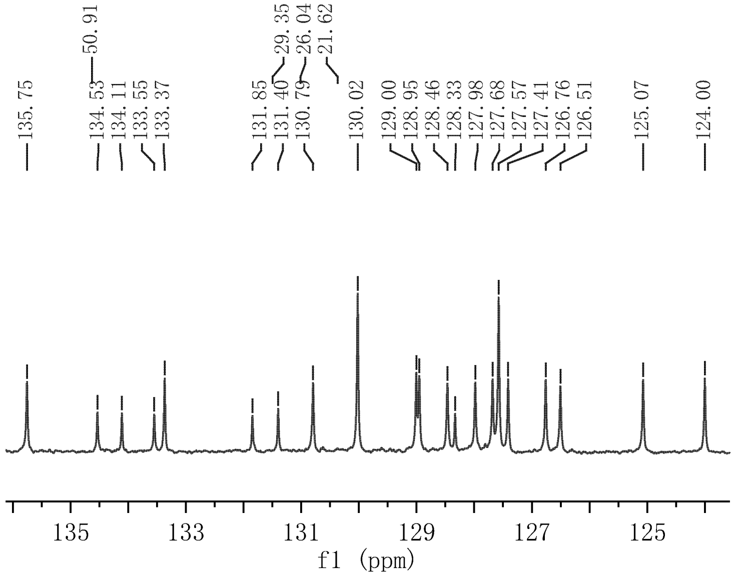

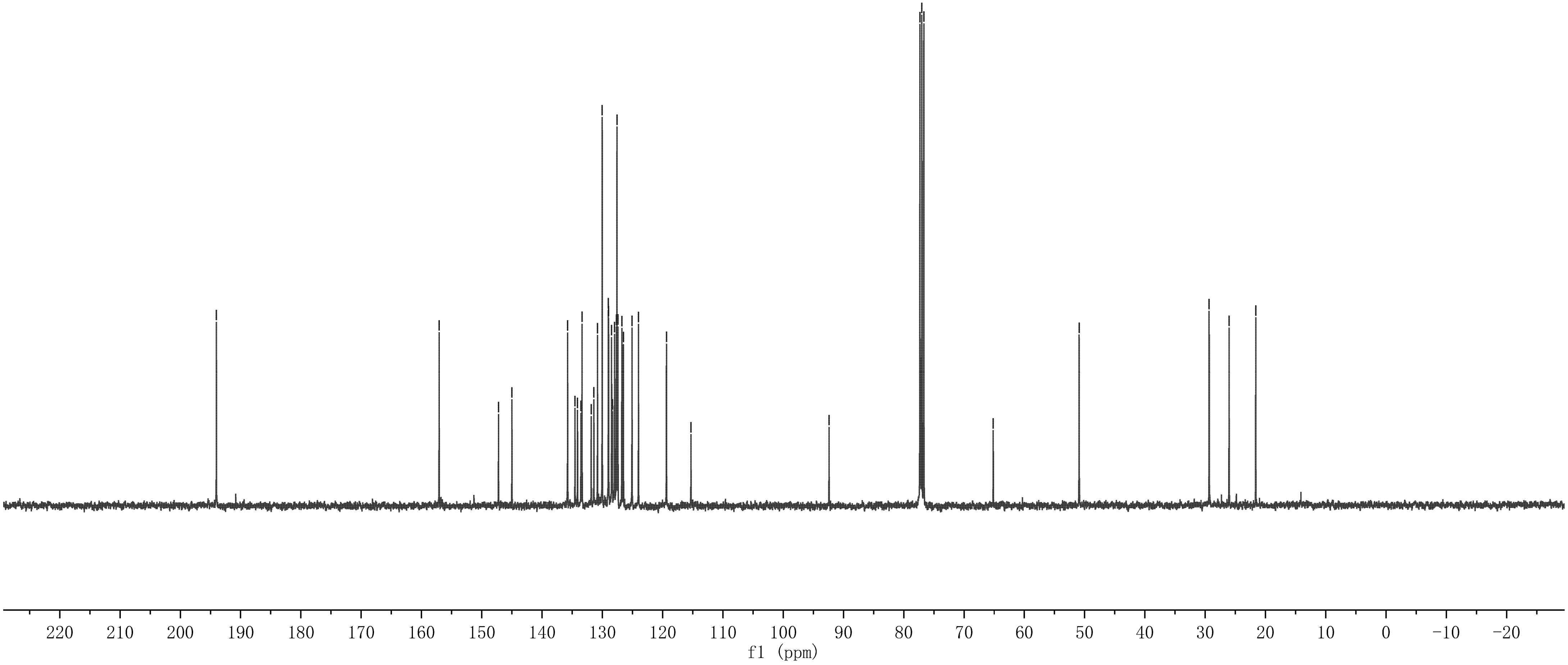

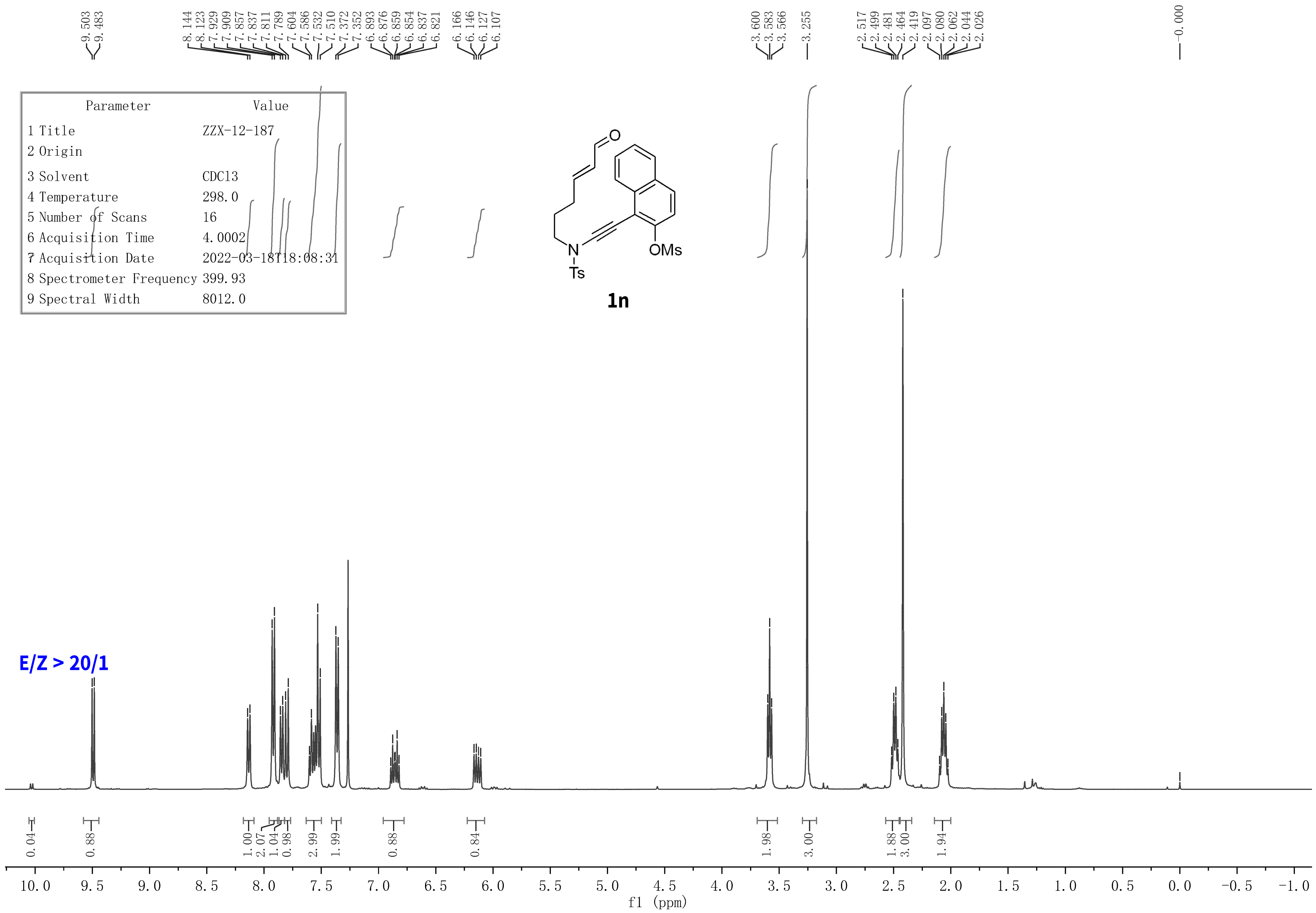
9.503
9.483
8.144
8.123
7.929
7.909
7.857
7.837
7.811
7.789
7.604
7.586
7.532
7.510
7.372
7.352
6.893
6.876
6.859
6.854
6.837
6.821
6.166
6.146
6.127
6.107
3.600
3.583
3.566
3.255
2.517
2.499
2.481
2.464
2.419
2.097
2.080
2.062
2.044
2.026
-0.000
Parameter Value
1 Title ZZX-12-187
2 Origin
3 Solvent CDCl3
4 Temperature 298.0
5 Number of Scans 16
6 Acquisition Time 4.0002
7 Acquisition Date 2022-03-18T18:08:31
8 Spectrometer Frequency 399.93
9 Spectral Width 8012.0
O
N
Ts
OMs
1n
E/Z > 20/1
0.04
0.88
1.00
2.07
1.04
0.98
2.99
1.99
0.88
0.84
1.98
3.00
1.88
3.00
1.94
10.0 9.5 9.0 8.5 8.0 7.5 7.0 6.5 6.0 5.5 5.0 4.5 4.0 3.5 3.0 2.5 2.0 1.5 1.0 0.5 0.0 -0.5 -1.0
f1 (ppm)

Parameter	Value
1 Title	ZZX-12-187
2 Origin	
3 Solvent	CDCl3
4 Temperature	298.0
5 Number of Scans	16
6 Acquisition Time	4.0002
7 Acquisition Date	2022-03-18T18:08:31
8 Spectrometer Frequency	399.93
9 Spectral Width	8012.0

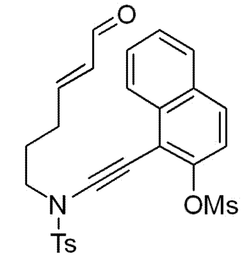

1n

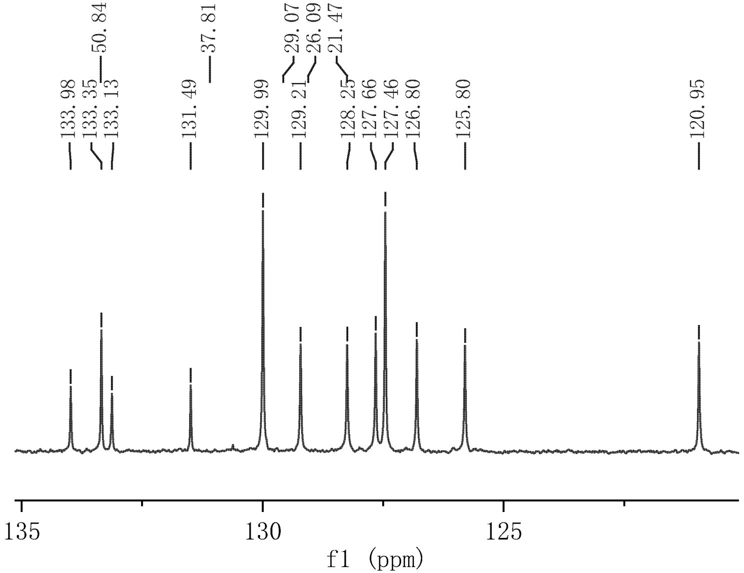

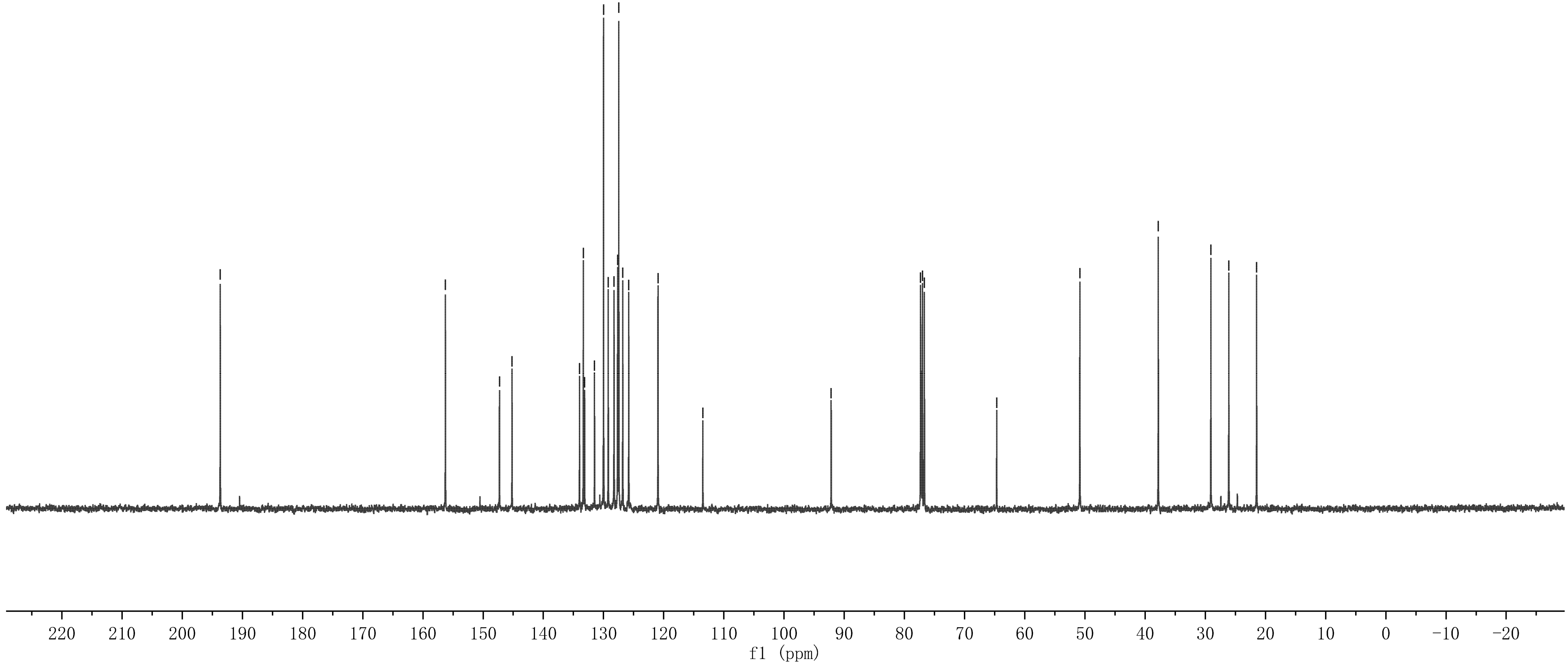

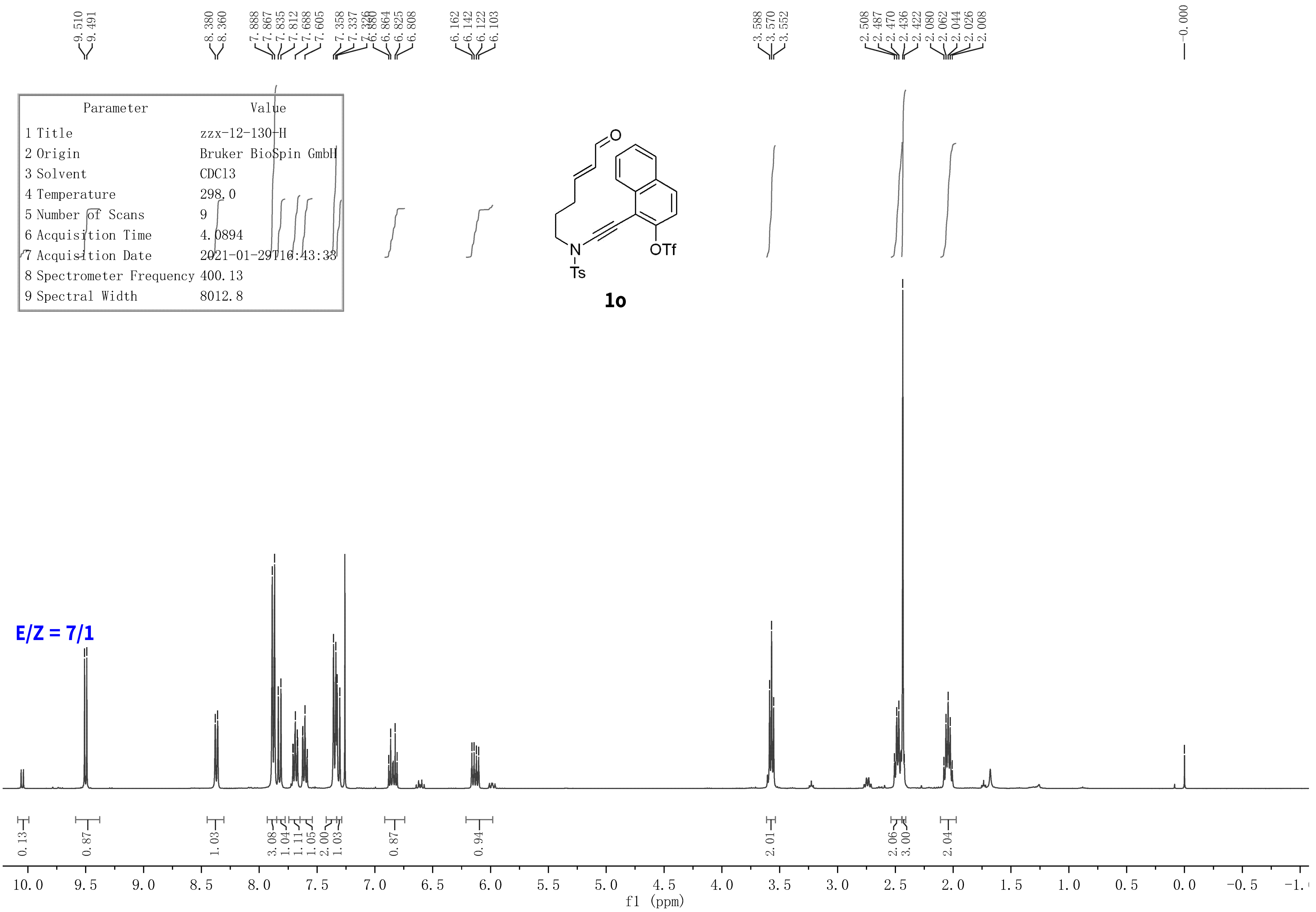
9.510
9.491
8.380
8.360
7.888
7.867
7.835
7.812
7.688
7.605
7.358
7.337
6.864
6.825
6.808
6.162
6.142
6.122
6.103
3.588
3.570
3.552
2.508
2.487
2.470
2.436
2.422
2.080
2.062
2.044
2.026
2.008
-0.000
Parameter Value
1 Title zzx-12-130-H
2 Origin Bruker BioSpin GmbH
3 Solvent CDCl3
4 Temperature 298.0
5 Number of Scans 9
6 Acquisition Time 4.0894
7 Acquisition Date 2021-01-29T16:43:33
8 Spectrometer Frequency 400.13
9 Spectral Width 8012.8
O
N
Ts
OTf
1o
E/Z = 7/1
0.13
0.87
1.03
3.08
1.04
1.11
1.05
2.00
1.03
0.87
0.94
2.01
2.06
3.00
2.04
10.0
9.5
9.0
8.5
8.0
7.5
7.0
6.5
6.0
5.5
5.0
4.5
4.0
3.5
3.0
2.5
2.0
1.5
1.0
0.5
0.0
-0.5
f1 (ppm)

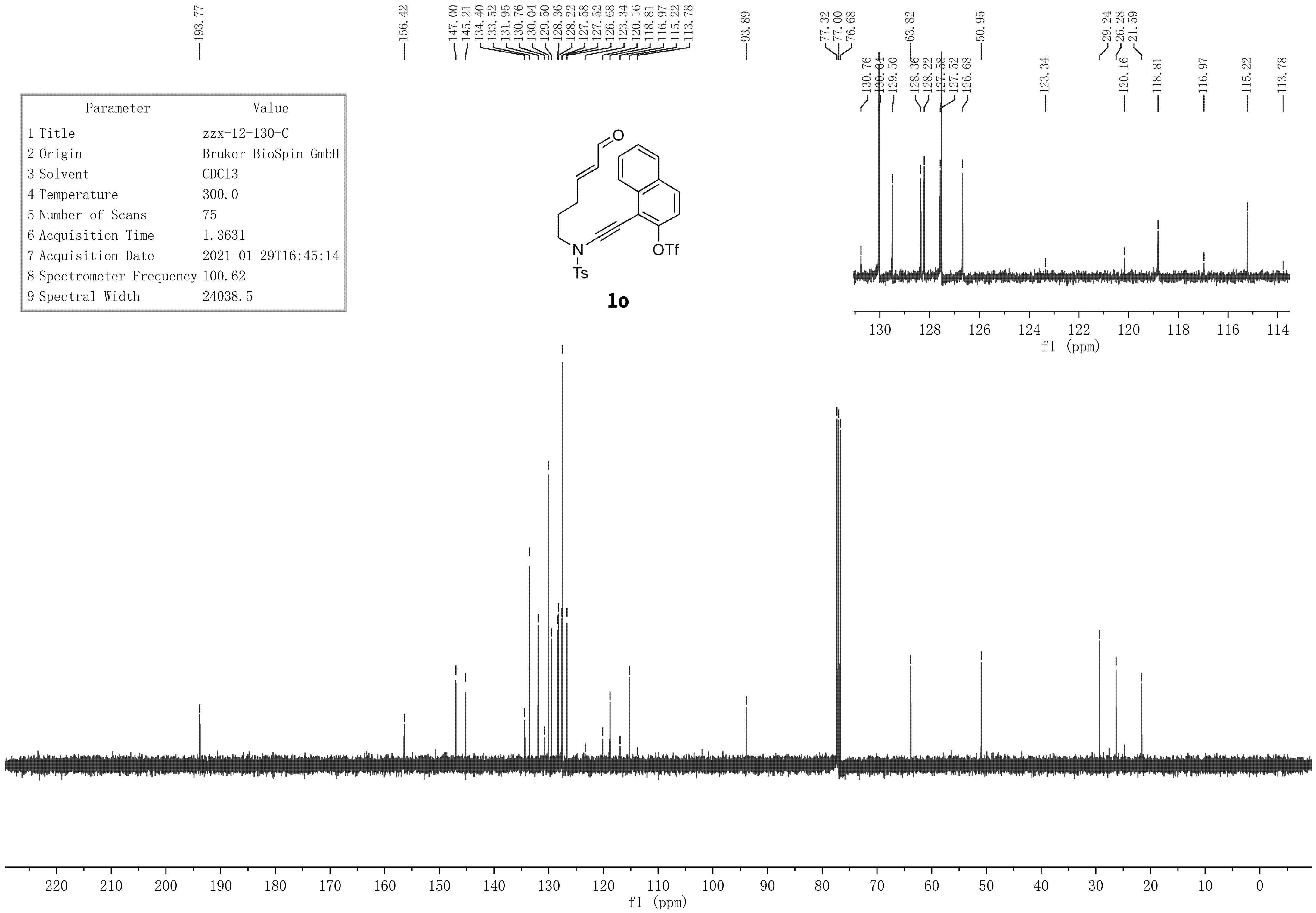

Parameter Value
1 Title zzx-12-130-C
2 Origin Bruker BioSpin GmbH
3 Solvent CDCl3
4 Temperature 300.0
5 Number of Scans 75
6 Acquisition Time 1.3631
7 Acquisition Date 2021-01-29T16:45:14
8 Spectrometer Frequency 100.62
9 Spectral Width 24038.5
O
N
Ts
OTf
1o
193.77
156.42
147.00
145.21
134.40
133.52
131.95
130.76
130.04
129.50
128.36
128.22
127.58
127.52
126.68
123.34
120.16
118.81
116.97
115.22
113.78
93.89
77.32
77.00
76.68
63.82
50.95
29.24
26.28
21.59
f1 (ppm)

Parameter	Value
1 Title	ZZX-18-S-OTf
2 Origin	
3 Solvent	CDCl3
4 Temperature	297.5
5 Number of Scans	16
6 Acquisition Time	1.0000
7 Acquisition Date	2023-02-09T11:31:45
8 Spectrometer Frequency	376.28
9 Spectral Width	96153.0

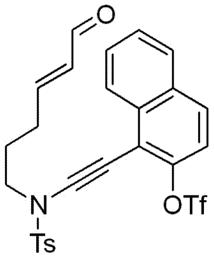

1o

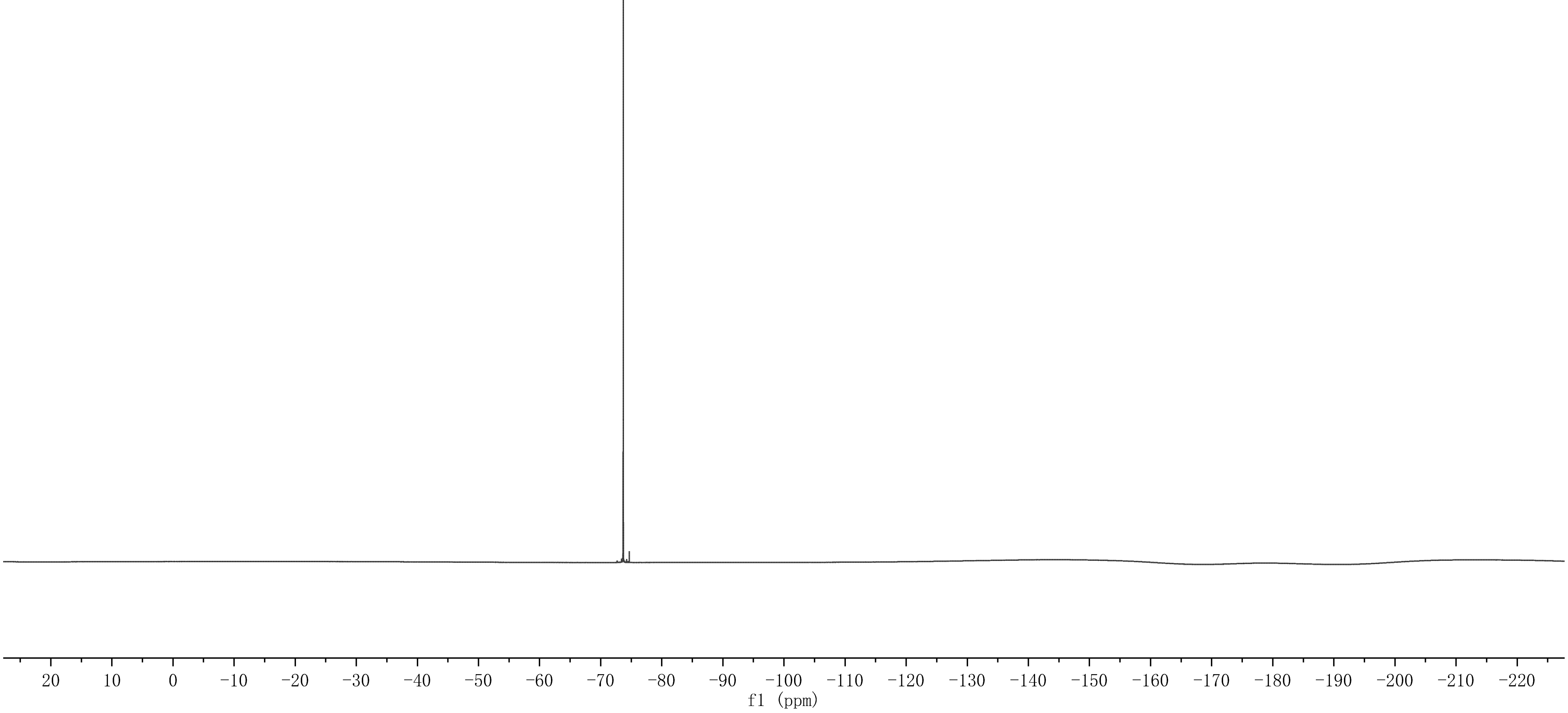

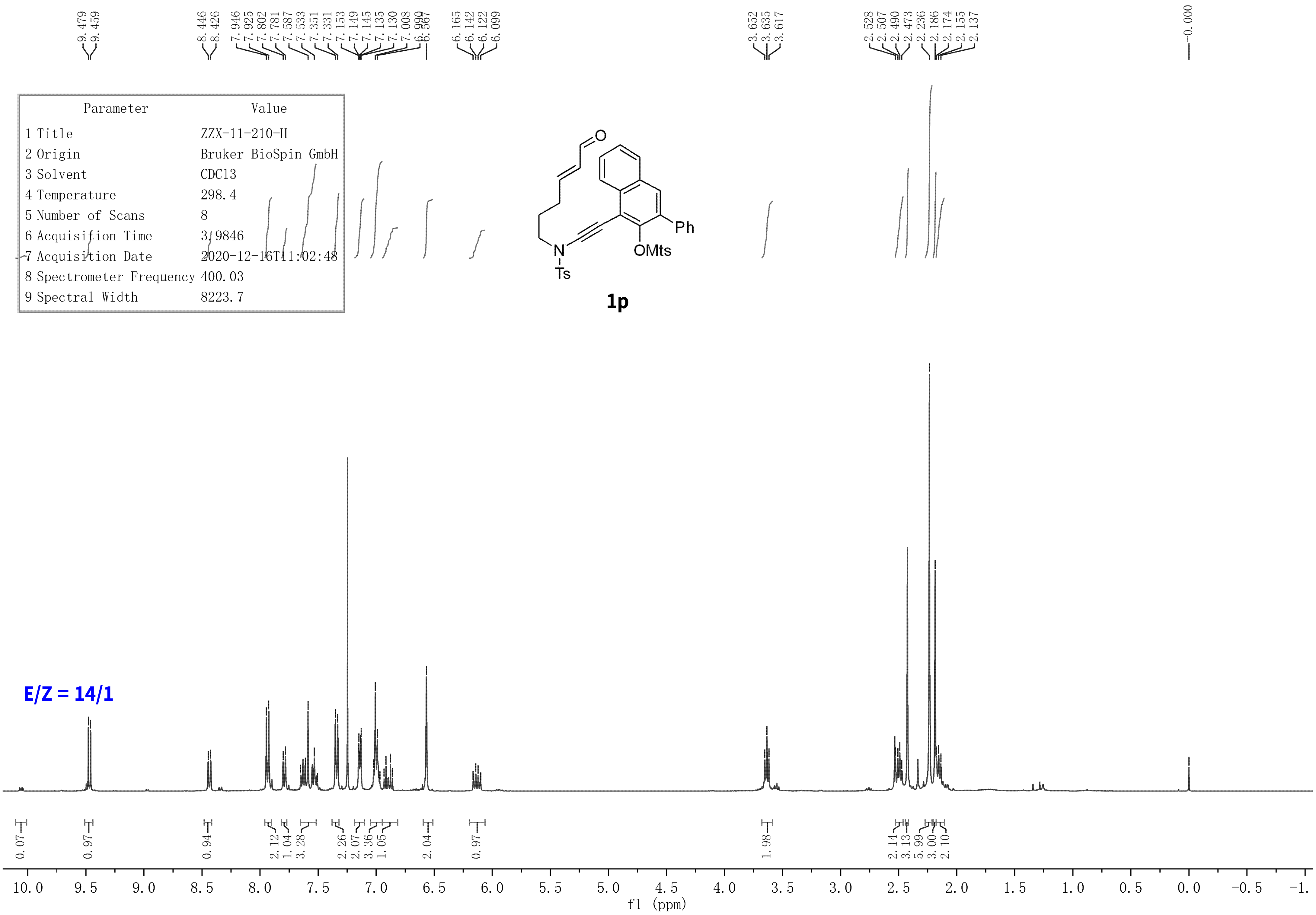

Parameter Value
1 Title ZZX-11-210-H
2 Origin Bruker BioSpin GmbH
3 Solvent CDCl3
4 Temperature 298.4
5 Number of Scans 8
6 Acquisition Time 3.9846
7 Acquisition Date 2020-12-16T11:02:48
8 Spectrometer Frequency 400.03
9 Spectral Width 8223.7
N
Ts
OMts
Ph
O
1p
E/Z = 14/1
9.479 9.459 8.446 8.426 7.946 7.925 7.802 7.781 7.587 7.533 7.351 7.331 7.153 7.149 7.145 7.135 7.130 7.008 6.990 6.567 6.165 6.142 6.122 6.099 3.652 3.635 3.617 2.528 2.507 2.490 2.473 2.236 2.186 2.174 2.155 2.137 -0.000
0.07 0.97 0.94 2.12 1.04 3.28 2.26 2.07 3.36 1.05 2.04 0.97 1.98 2.14 3.13 5.99 3.00 2.10
10.0 9.5 9.0 8.5 8.0 7.5 7.0 6.5 6.0 5.5 5.0 4.5 4.0 3.5 3.0 2.5 2.0 1.5 1.0 0.5 0.0 -0.5 -1.
f1 (ppm)

Parameter	Value
1 Title	ZZX-11-210-C
2 Origin	Bruker BioSpin GmbH
3 Solvent	CDCl3
4 Temperature	299.0
5 Number of Scans	61
6 Acquisition Time	1.3631
7 Acquisition Date	2020-12-16T11:06:27
8 Spectrometer Frequency	100.59
9 Spectral Width	24038.5

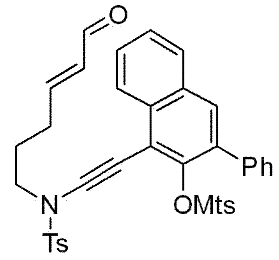

1p

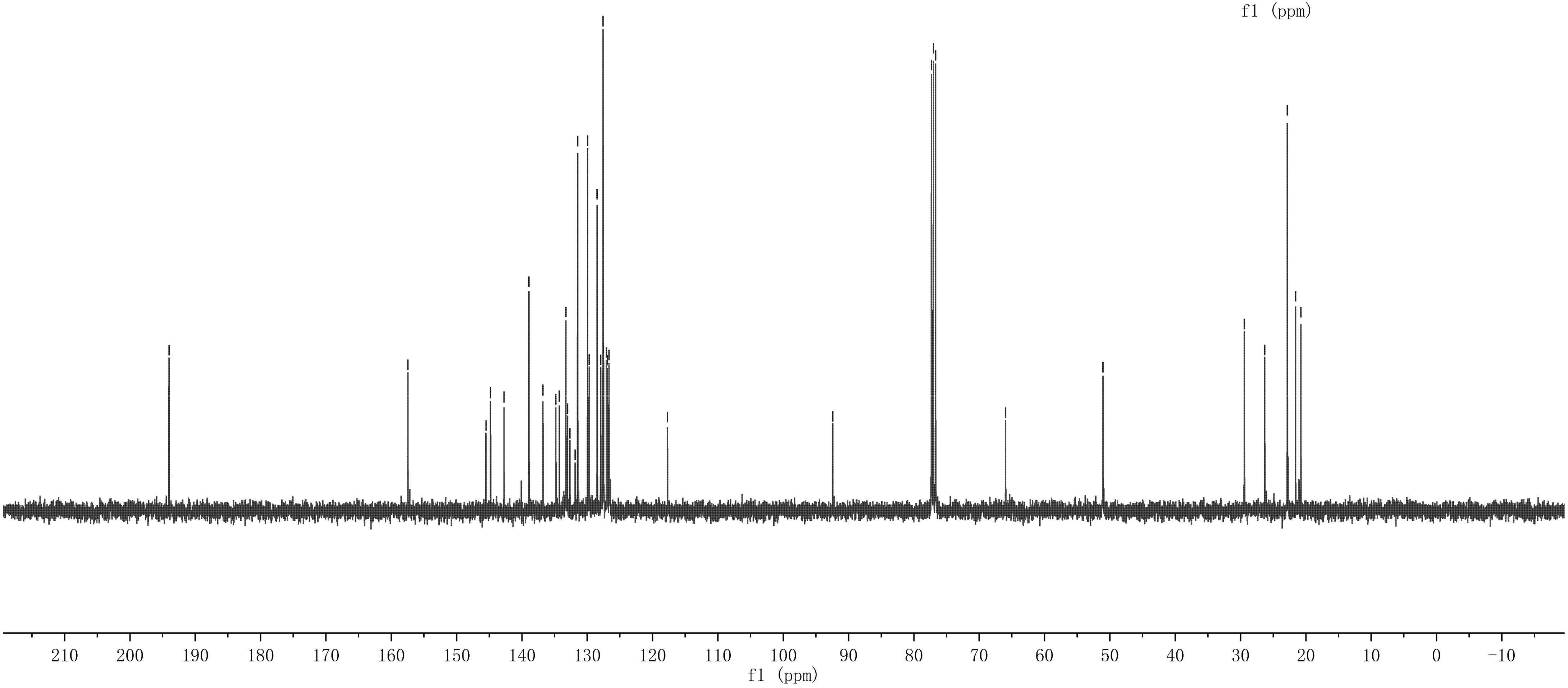

Parameter	Value
1 Title	zzx-18-s-3-OMe
2 Origin	Bruker BioSpin GmbH
3 Solvent	CDCl3
4 Temperature	298.0
5 Number of Scans	4
6 Acquisition Time	4.0894
7 Acquisition Date	2023-02-24T00:18:00
8 Spectrometer Frequency	400.13
9 Spectral Width	8012.8

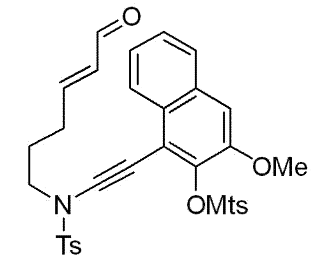

1q

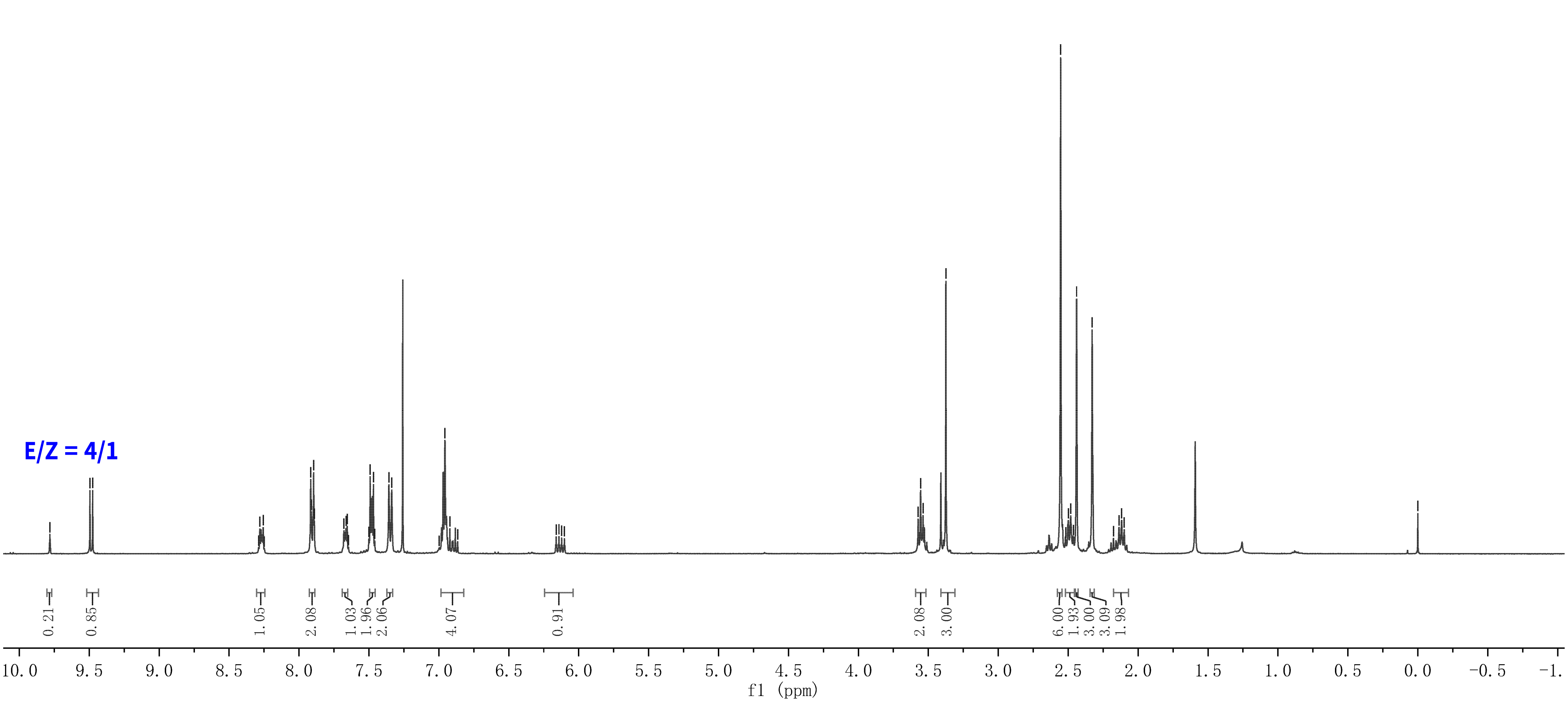

Parameter	Value
1 Title	ZZX-11.38
2 Origin	
3 Solvent	CDCl3
4 Temperature	297.8
5 Number of Scans	200
6 Acquisition Time	1.0000
7 Acquisition Date	2022-03-18T09:45:49
8 Spectrometer Frequency	100.56
9 Spectral Width	26041.0

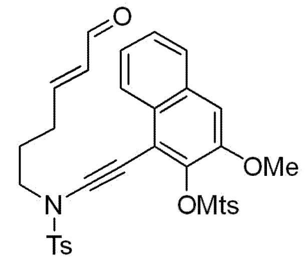

1q

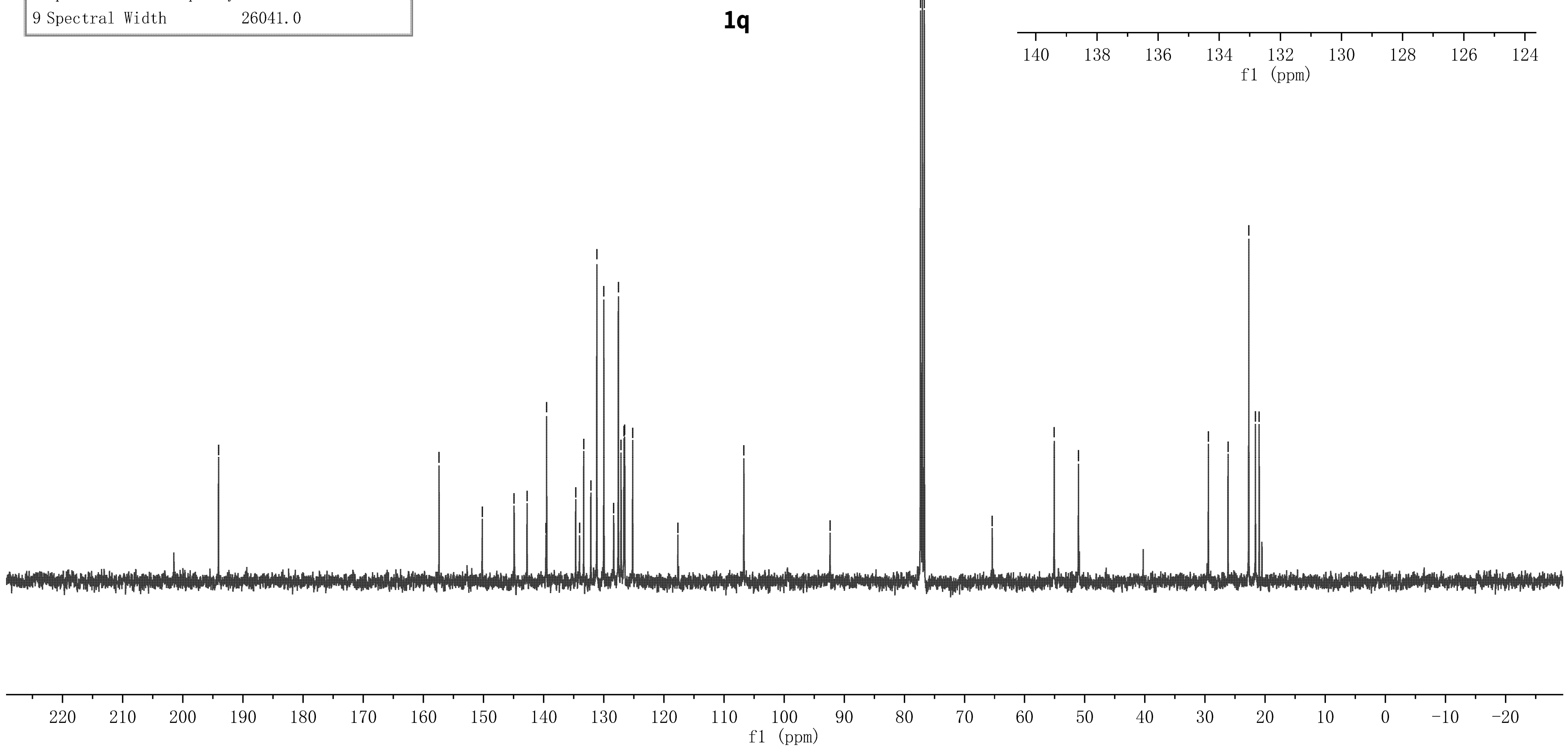

Parameter	Value
1 Title	zzx-11-84-H-1
2 Origin	Bruker BioSpin GmbH
3 Solvent	CDCl3
4 Temperature	298.0
5 Number of Scans	9
6 Acquisition Time	4.0894
7 Acquisition Date	2020-11-06T14:26:25
8 Spectrometer Frequency	400.13
9 Spectral Width	8012.8

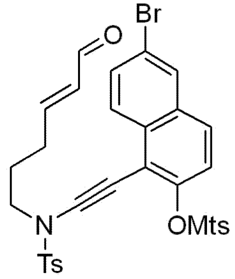

1r

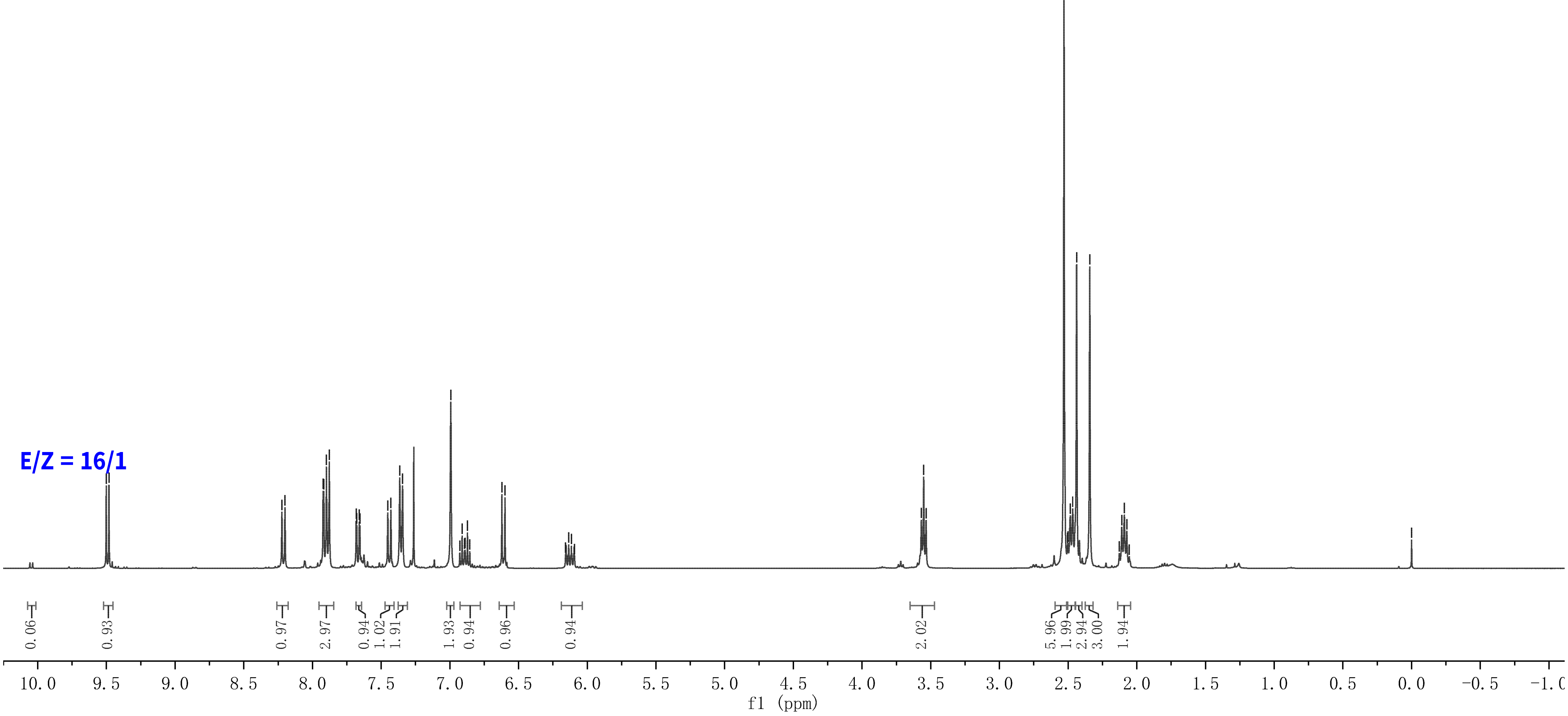

Parameter	Value
1 Title	zzx-11-84-C
2 Origin	Bruker BioSpin GmbH
3 Solvent	CDCl3
4 Temperature	300.0
5 Number of Scans	19
6 Acquisition Time	1.3631
7 Acquisition Date	2020-11-06T14:28:44
8 Spectrometer Frequency	100.61
9 Spectral Width	24038.5

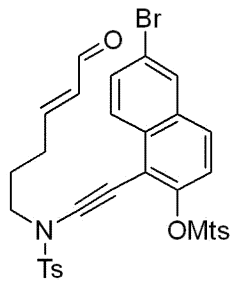

1r

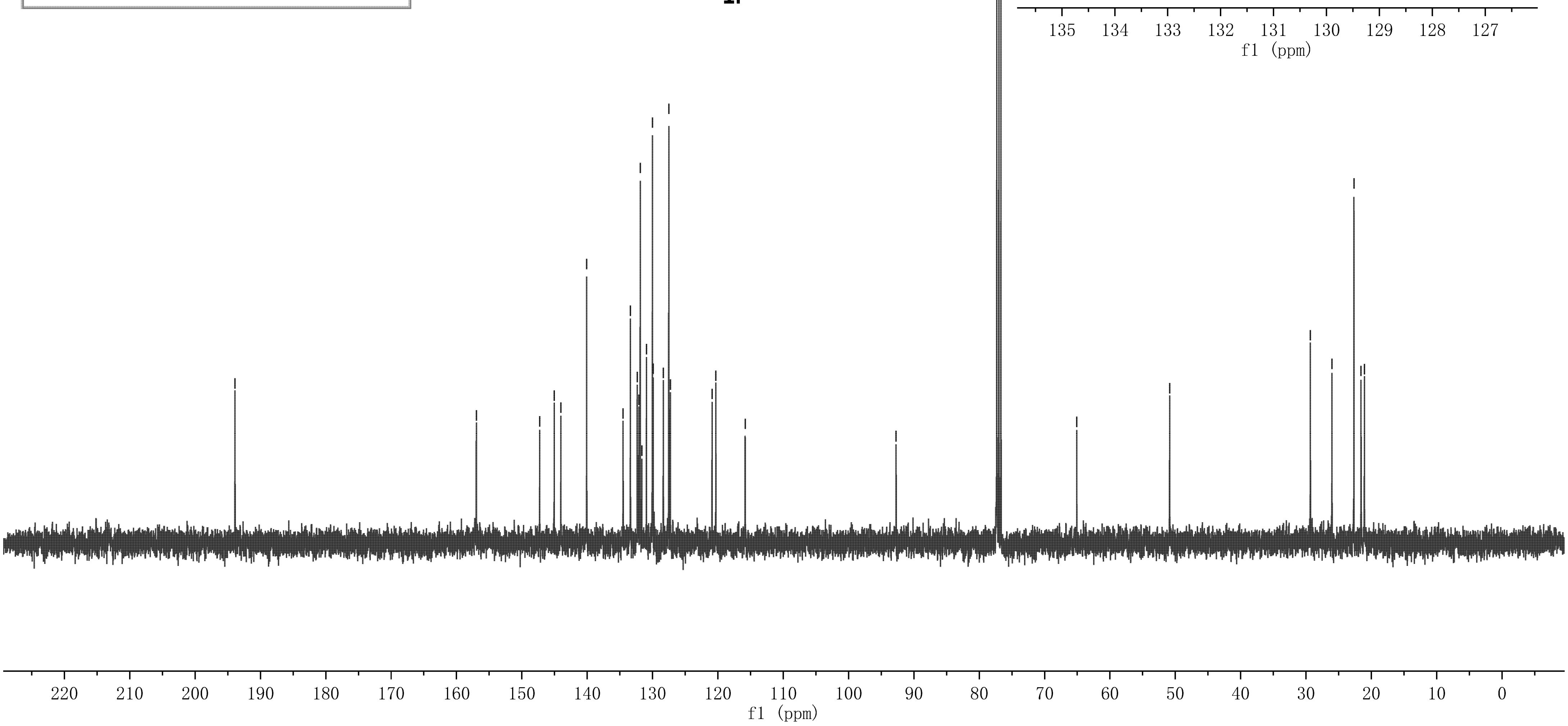

Parameter	Value
1 Title	zzx-11-162-H
2 Origin	Bruker BioSpin GmbH
3 Solvent	CDCl3
4 Temperature	298.0
5 Number of Scans	8
6 Acquisition Time	4.0894
7 Acquisition Date	2020-11-29T14:58:29
8 Spectrometer Frequency	400.13
9 Spectral Width	8012.8

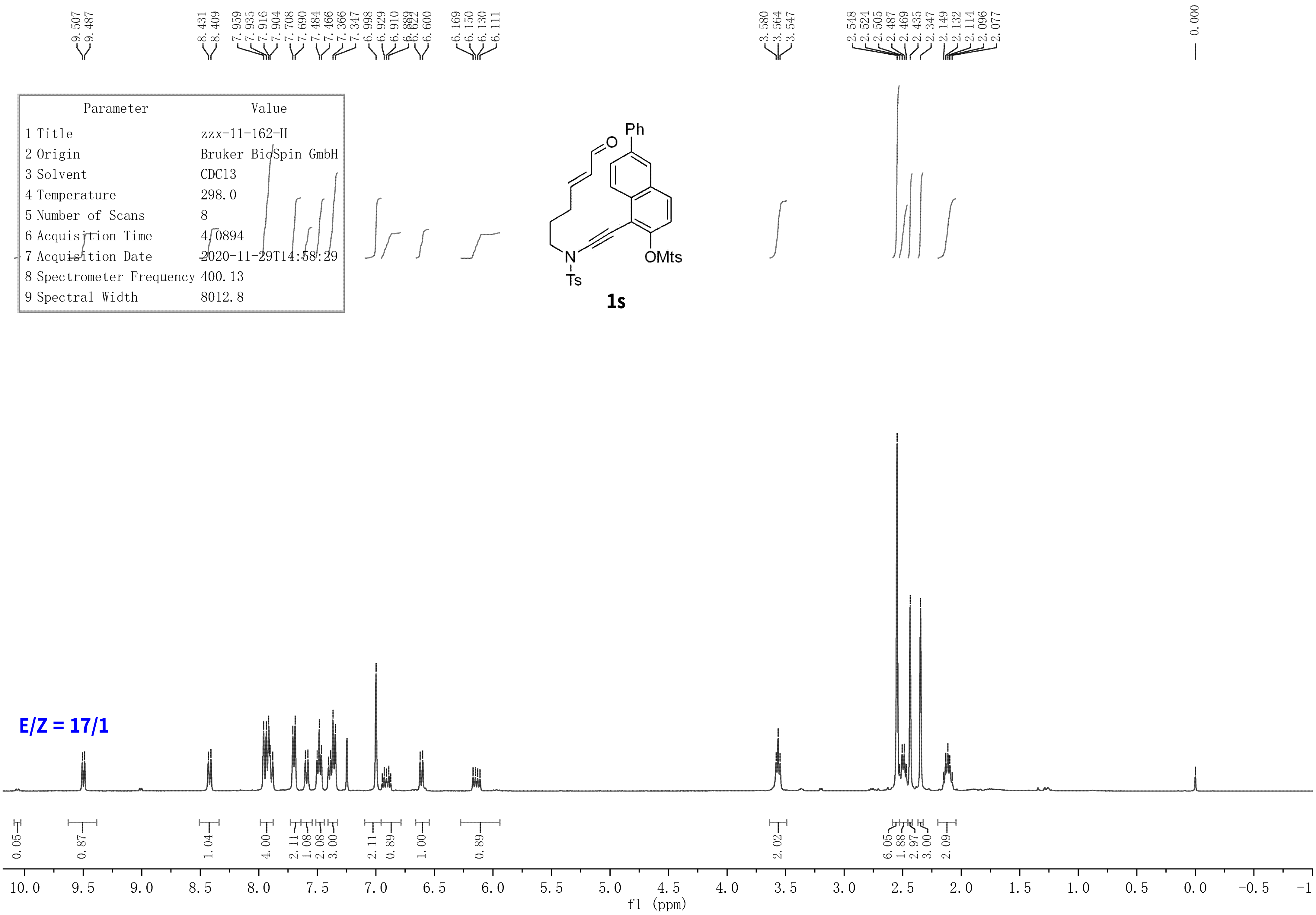

Parameter	Value
1 Title	zzx-11-162-C
2 Origin	Bruker BioSpin GmbH
3 Solvent	CDCl3
4 Temperature	300.0
5 Number of Scans	52
6 Acquisition Time	1.3631
7 Acquisition Date	2020-11-29T15:00:13
8 Spectrometer Frequency	100.61
9 Spectral Width	24038.5

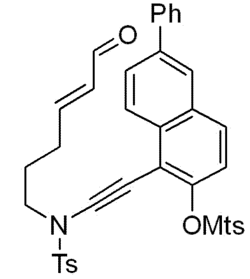

1s

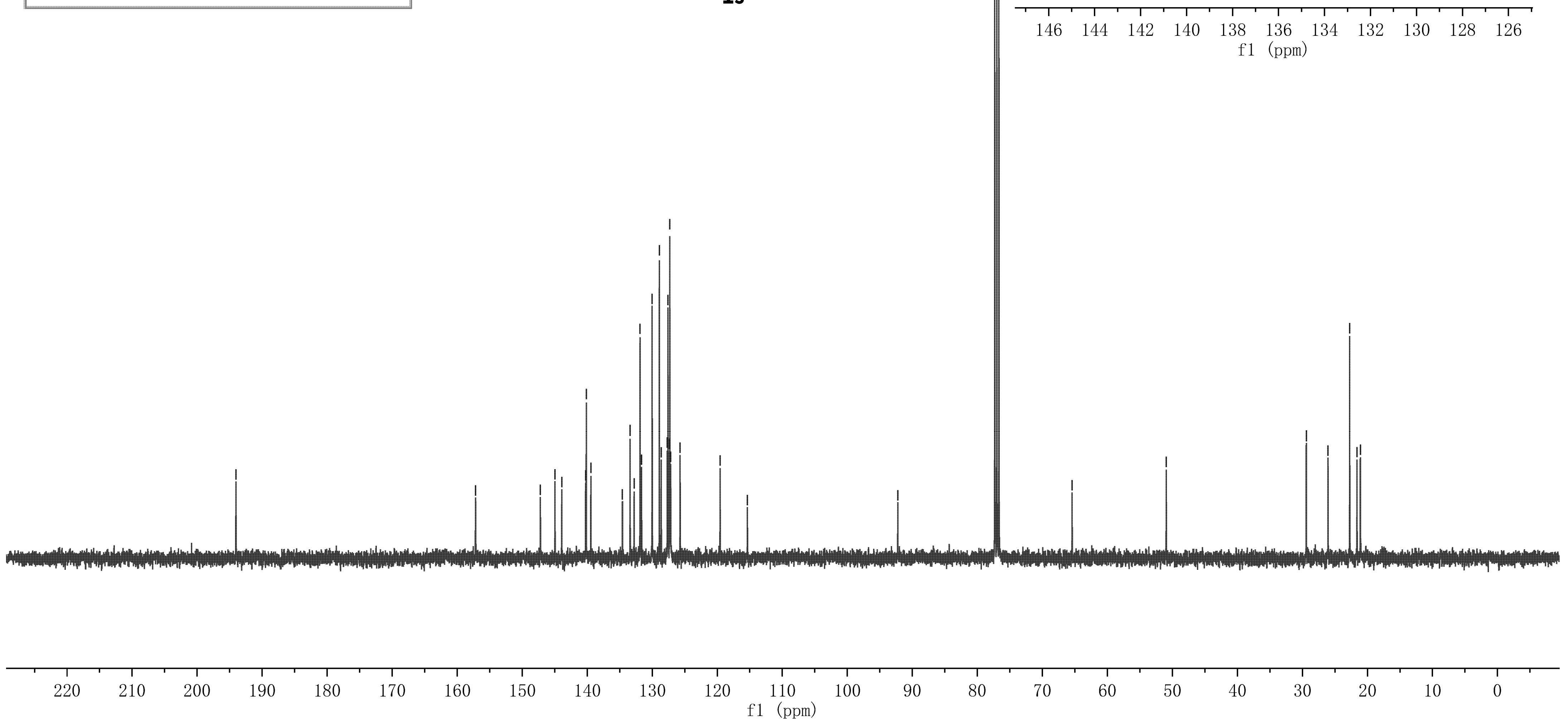

Parameter	Value
1 Title	ZZX-11-196-H
2 Origin	Bruker BioSpin GmbH
3 Solvent	CDCl3
4 Temperature	298.5
5 Number of Scans	12
6 Acquisition Time	3.9846
7 Acquisition Date	2020-12-16T11:23:10
8 Spectrometer Frequency	400.03
9 Spectral Width	8223.7

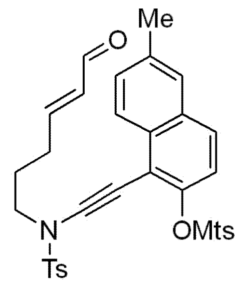

1t

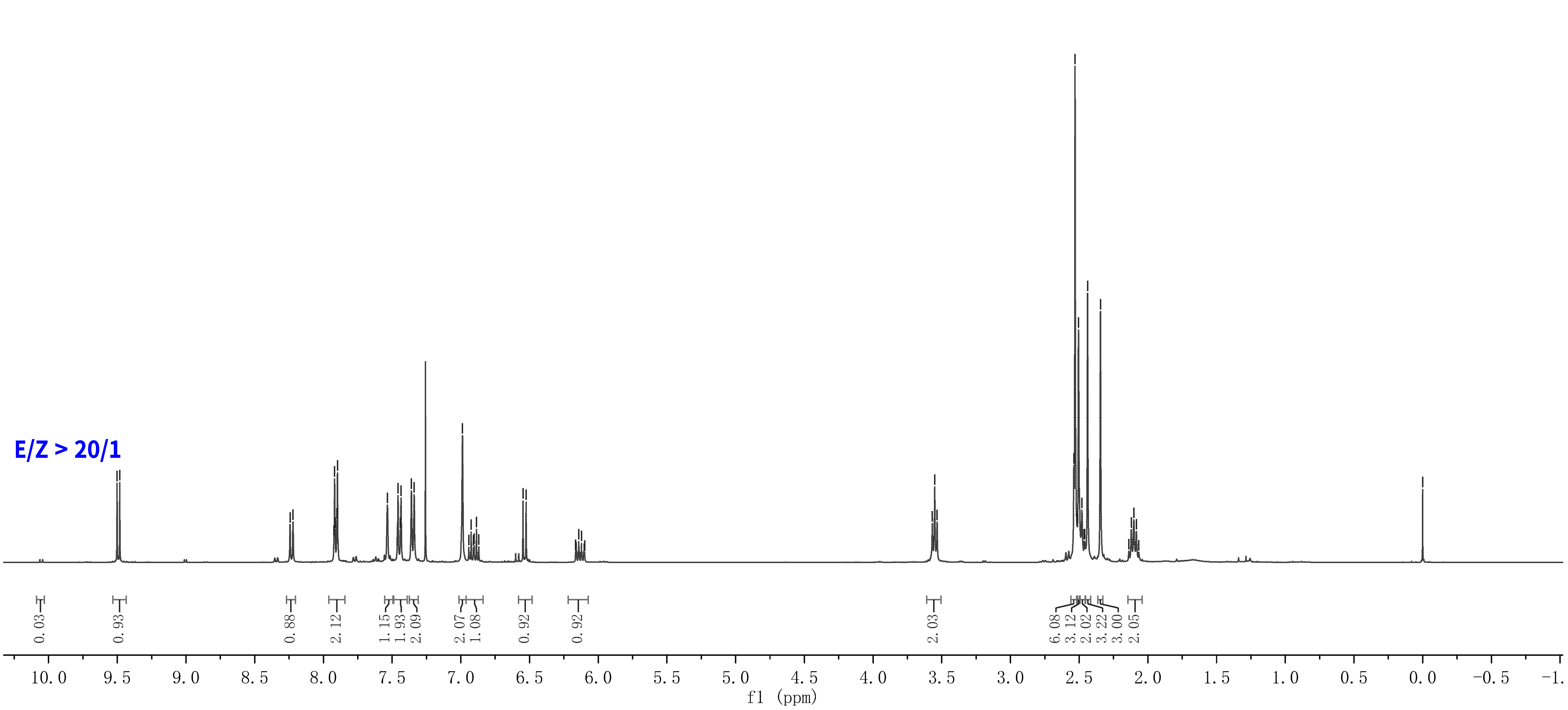

Parameter	Value
1 Title	ZZX-11-196-C
2 Origin	Bruker BioSpin GmbH
3 Solvent	CDCl3
4 Temperature	299.0
5 Number of Scans	104
6 Acquisition Time	1.3631
7 Acquisition Date	2020-12-16T11:25:03
8 Spectrometer Frequency	100.59
9 Spectral Width	24038.5

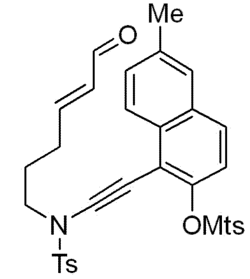

1t

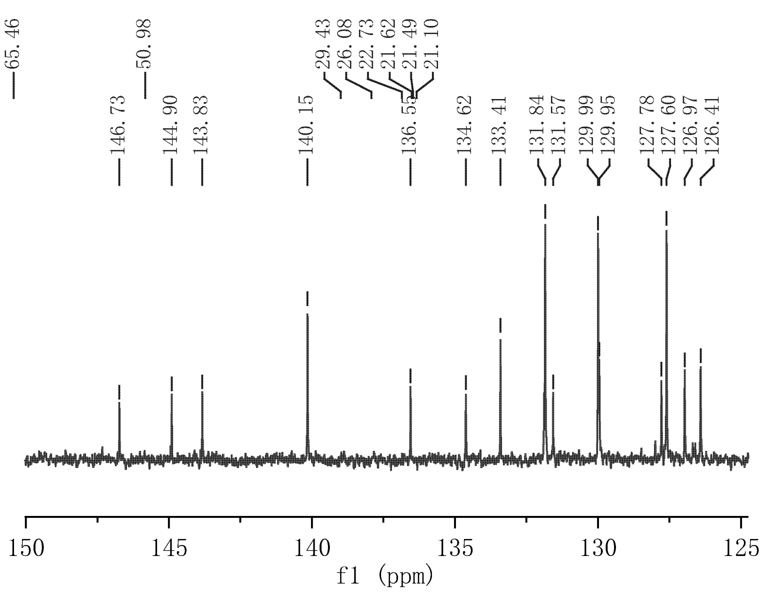

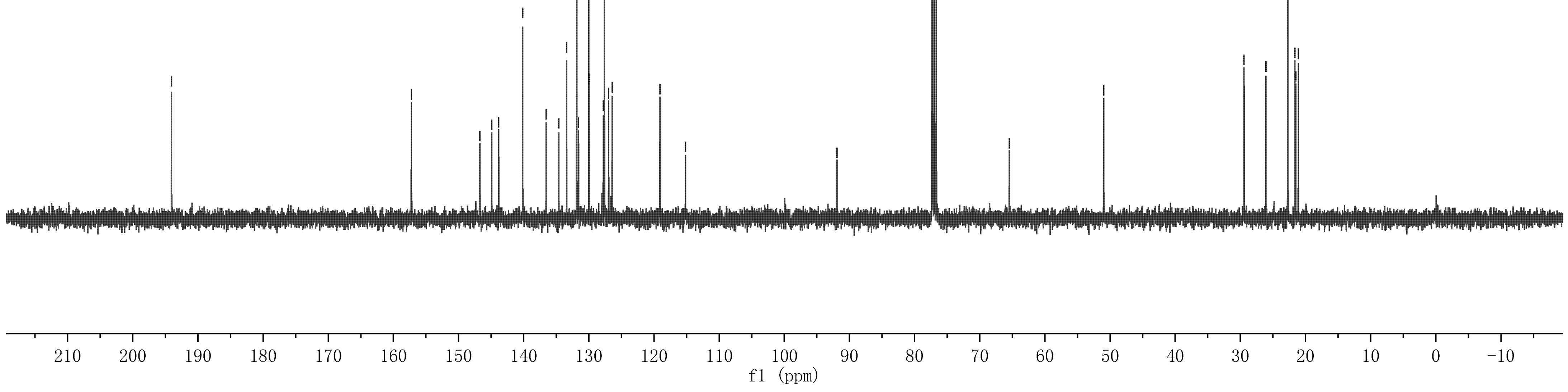

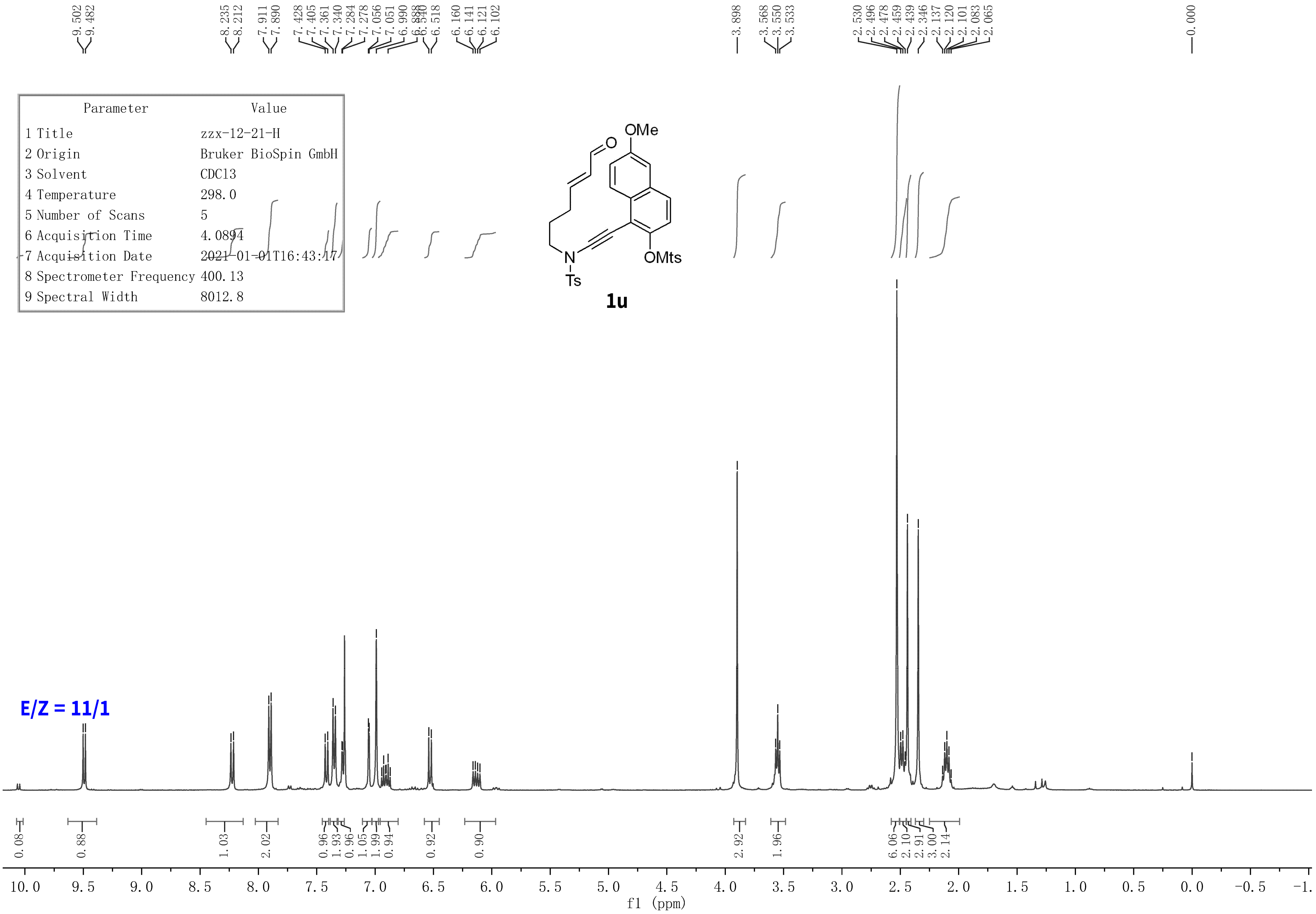

Parameter	Value
1 Title	zzx-12-21-H
2 Origin	Bruker BioSpin GmbH
3 Solvent	CDCl3
4 Temperature	298.0
5 Number of Scans	5
6 Acquisition Time	4.0894
7 Acquisition Date	2021-01-01T16:43:17
8 Spectrometer Frequency	400.13
9 Spectral Width	8012.8

Parameter	Value
1 Title	zzx-12-21-C
2 Origin	Bruker BioSpin GmbH
3 Solvent	CDCl3
4 Temperature	300.0
5 Number of Scans	39
6 Acquisition Time	1.3631
7 Acquisition Date	2021-01-01T16:44:40
8 Spectrometer Frequency	100.61
9 Spectral Width	24038.5

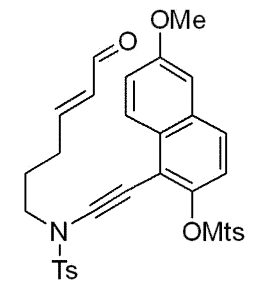

1u

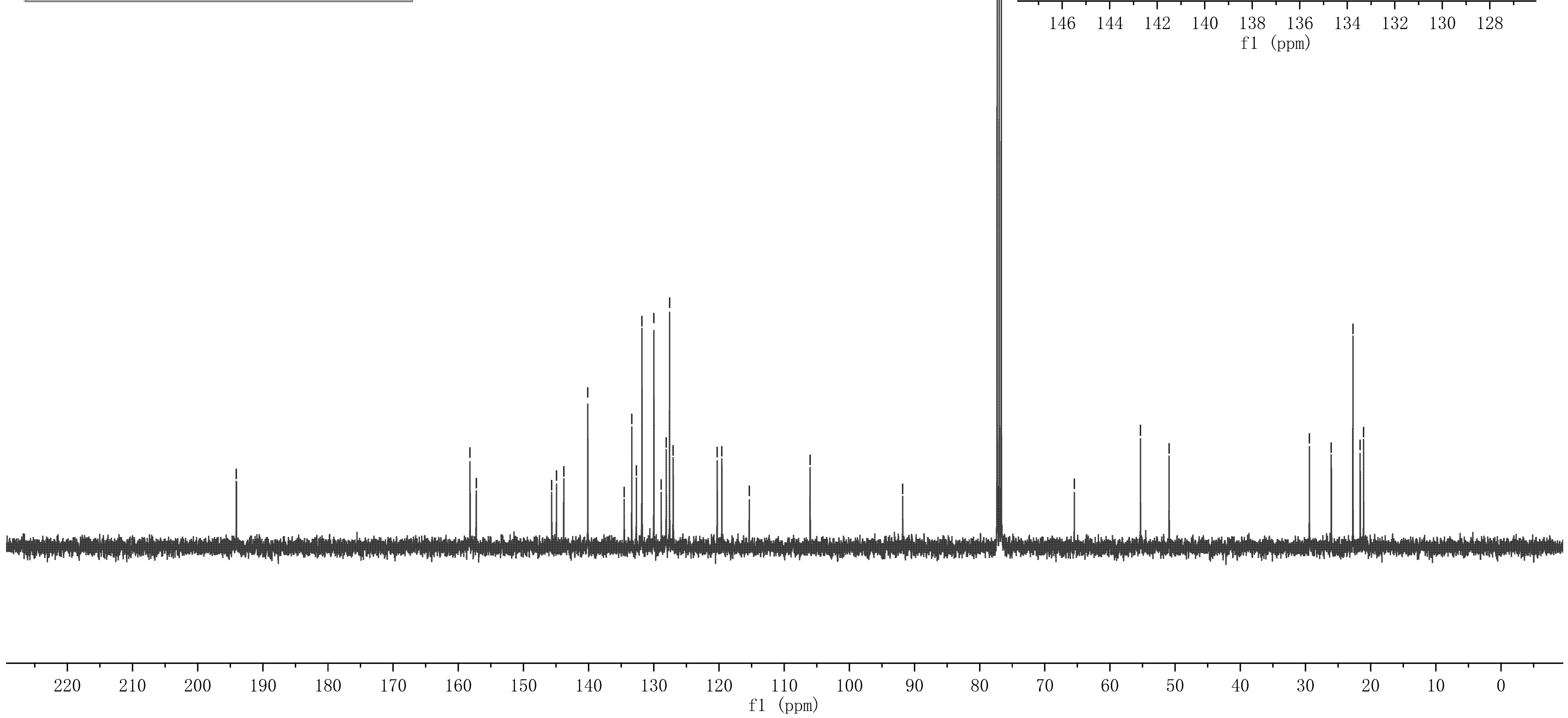

Parameter	Value
1 Title	zzx-11-118-H
2 Origin	Bruker BioSpin GmbH
3 Solvent	CDCl3
4 Temperature	298.0
5 Number of Scans	5
6 Acquisition Time	4.0894
7 Acquisition Date	2020-11-13T09:51:32
8 Spectrometer Frequency	400.13
9 Spectral Width	8012.8

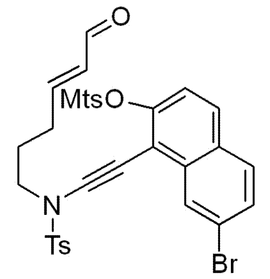

1v

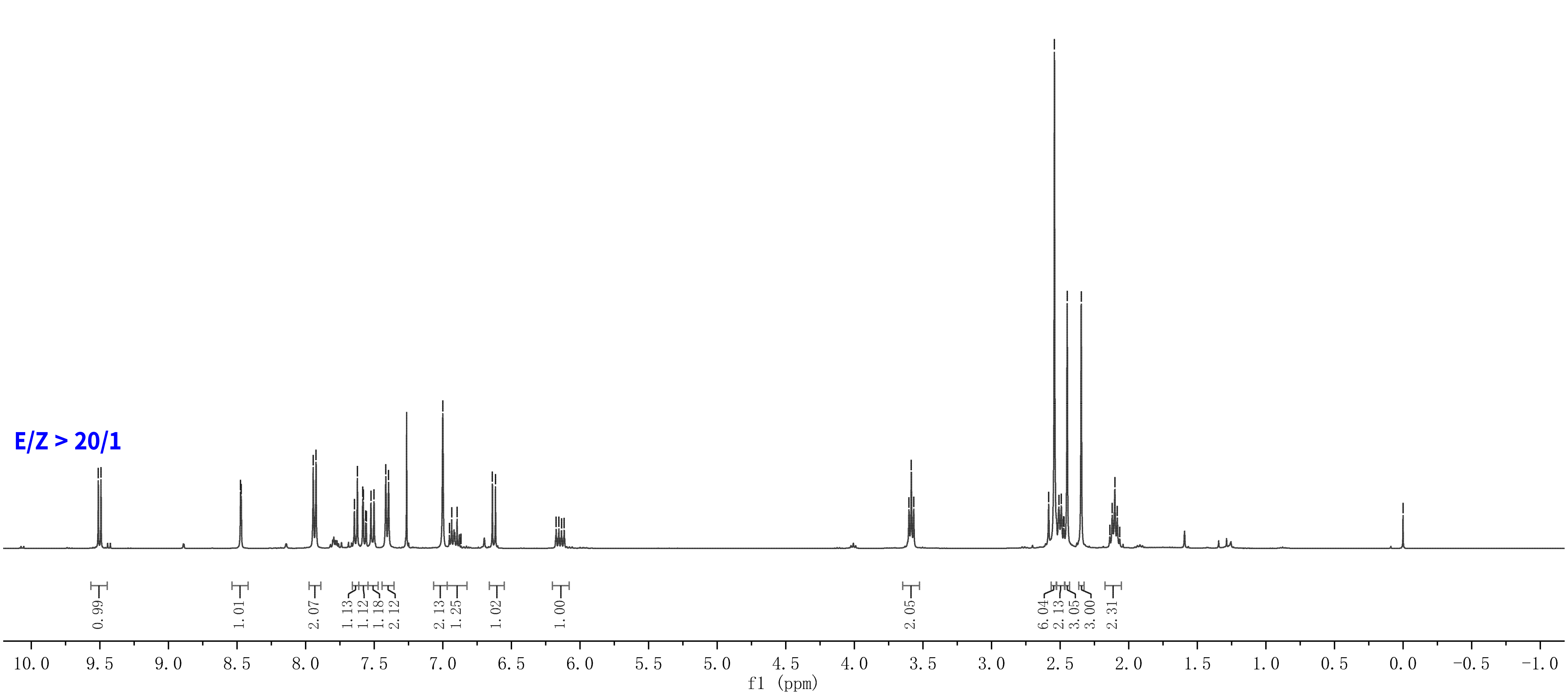

Parameter	Value
1 Title	zzx-11-118-C
2 Origin	Bruker BioSpin GmbH
3 Solvent	CDCl3
4 Temperature	300.0
5 Number of Scans	14
6 Acquisition Time	1.3631
7 Acquisition Date	2020-11-13T09:52:40
8 Spectrometer Frequency	100.61
9 Spectral Width	24038.5

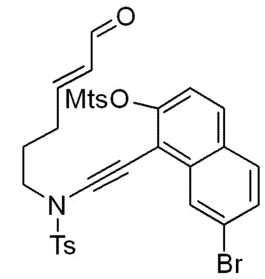

1v

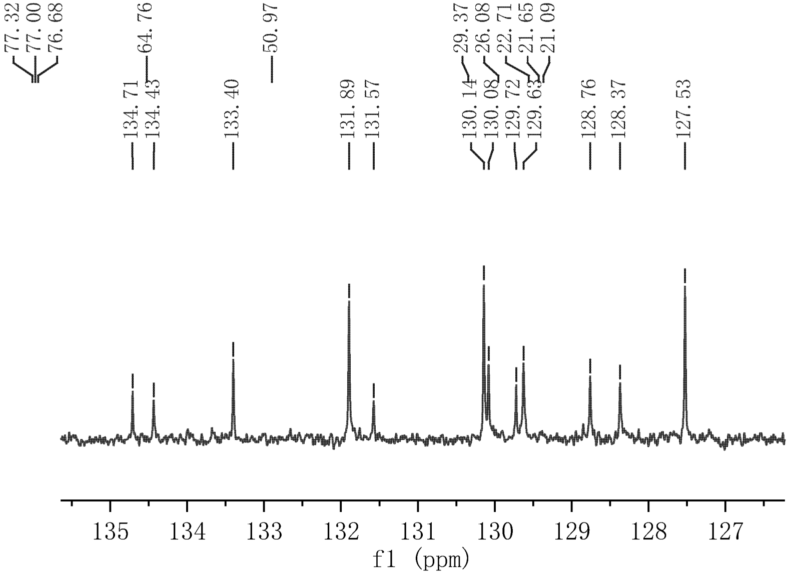

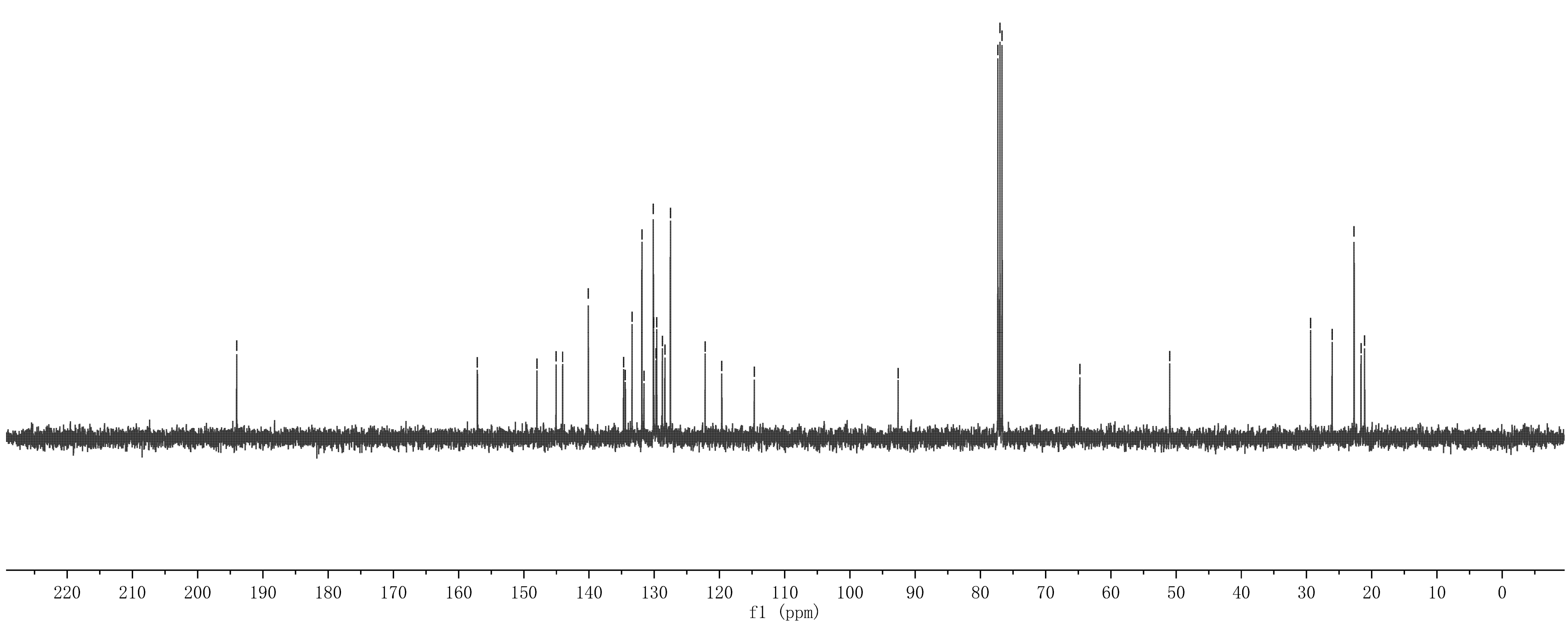

Parameter	Value
1 Title	zzx-11-163-H
2 Origin	Bruker BioSpin GmbH
3 Solvent	CDCl3
4 Temperature	298.0
5 Number of Scans	13
6 Acquisition Time	4.0894
7 Acquisition Date	2020-11-29T14:49:12
8 Spectrometer Frequency	400.13
9 Spectral Width	8012.8

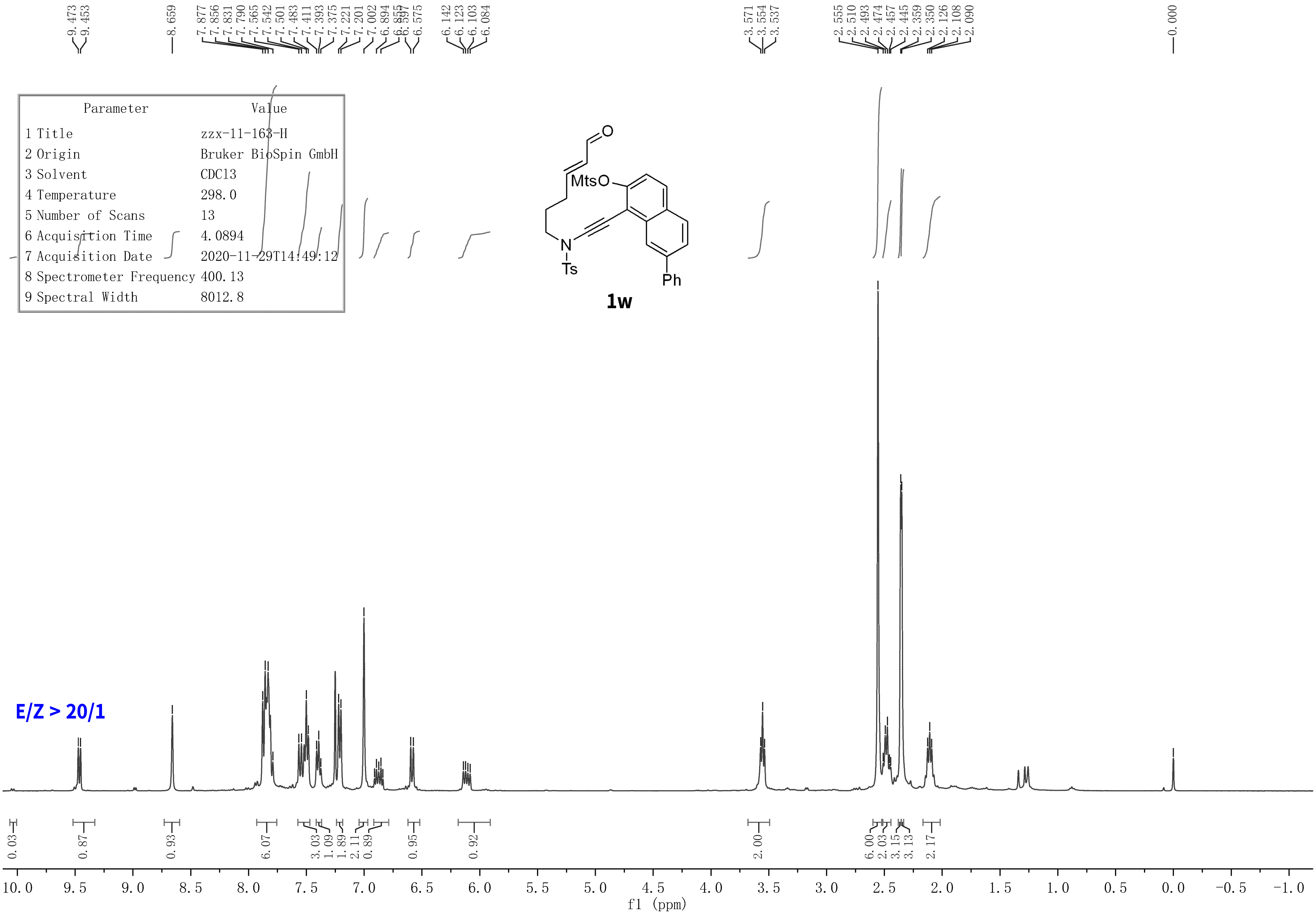

Parameter	Value
1 Title	zzx-11-163-C
2 Origin	Bruker BioSpin GmbH
3 Solvent	CDCl3
4 Temperature	300.0
5 Number of Scans	80
6 Acquisition Time	1.3631
7 Acquisition Date	2020-11-29T14:51:19
8 Spectrometer Frequency	100.61
9 Spectral Width	24038.5

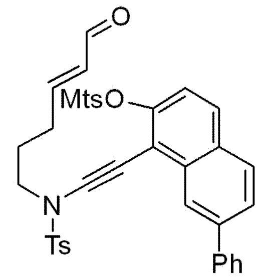

1w

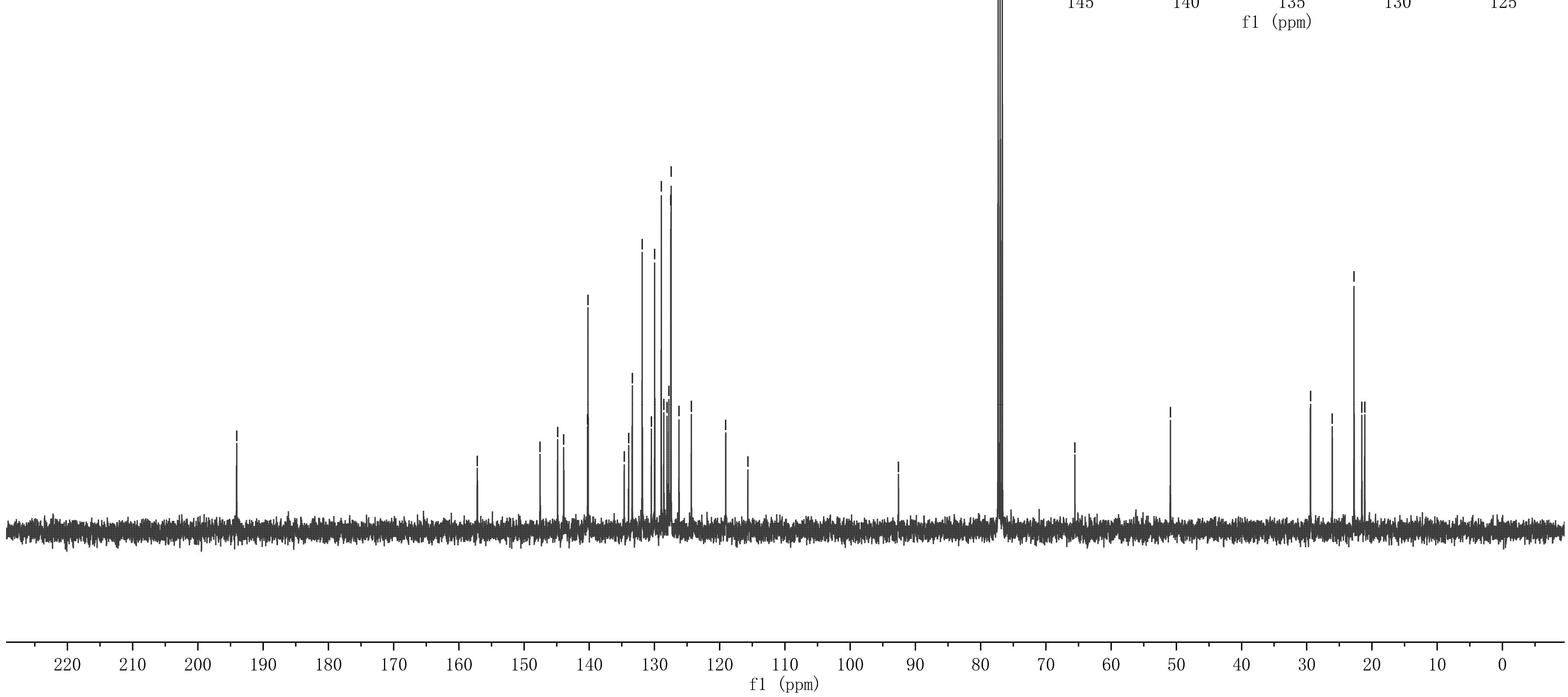

Parameter	Value
1 Title	ZZX-11-197-H
2 Origin	Bruker BioSpin GmbH
3 Solvent	CDCl3
4 Temperature	298.4
5 Number of Scans	10
6 Acquisition Time	3.9846
7 Acquisition Date	2020-12-16T11:13:00
8 Spectrometer Frequency	400.03
9 Spectral Width	8223.7

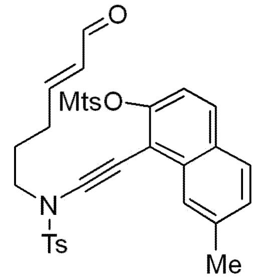

1x

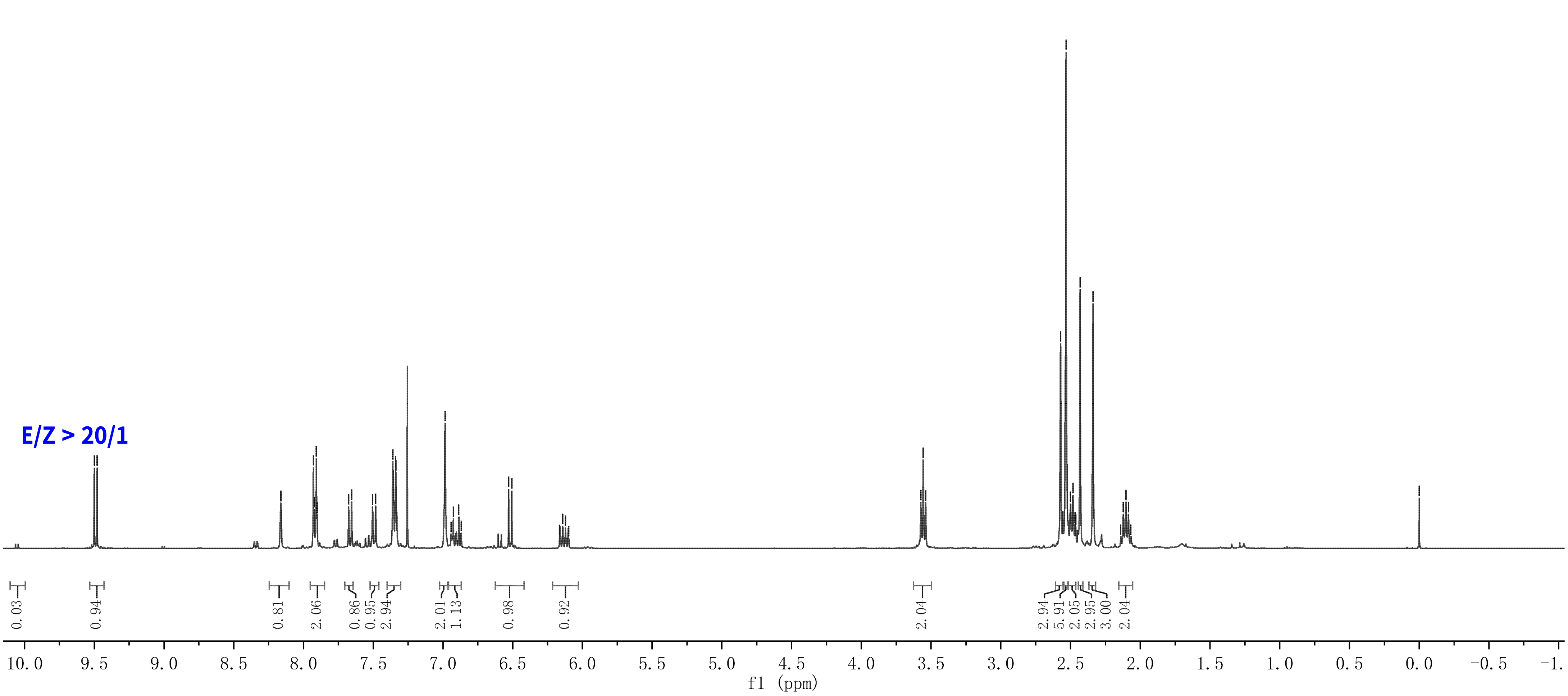

Parameter	Value
1 Title	ZZX-11-197-C
2 Origin	Bruker BioSpin GmbH
3 Solvent	CDCl3
4 Temperature	299.2
5 Number of Scans	71
6 Acquisition Time	1.3631
7 Acquisition Date	2020-12-16T11:16:00
8 Spectrometer Frequency	100.59
9 Spectral Width	24038.5

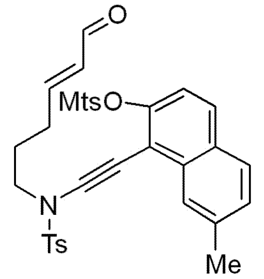

1x

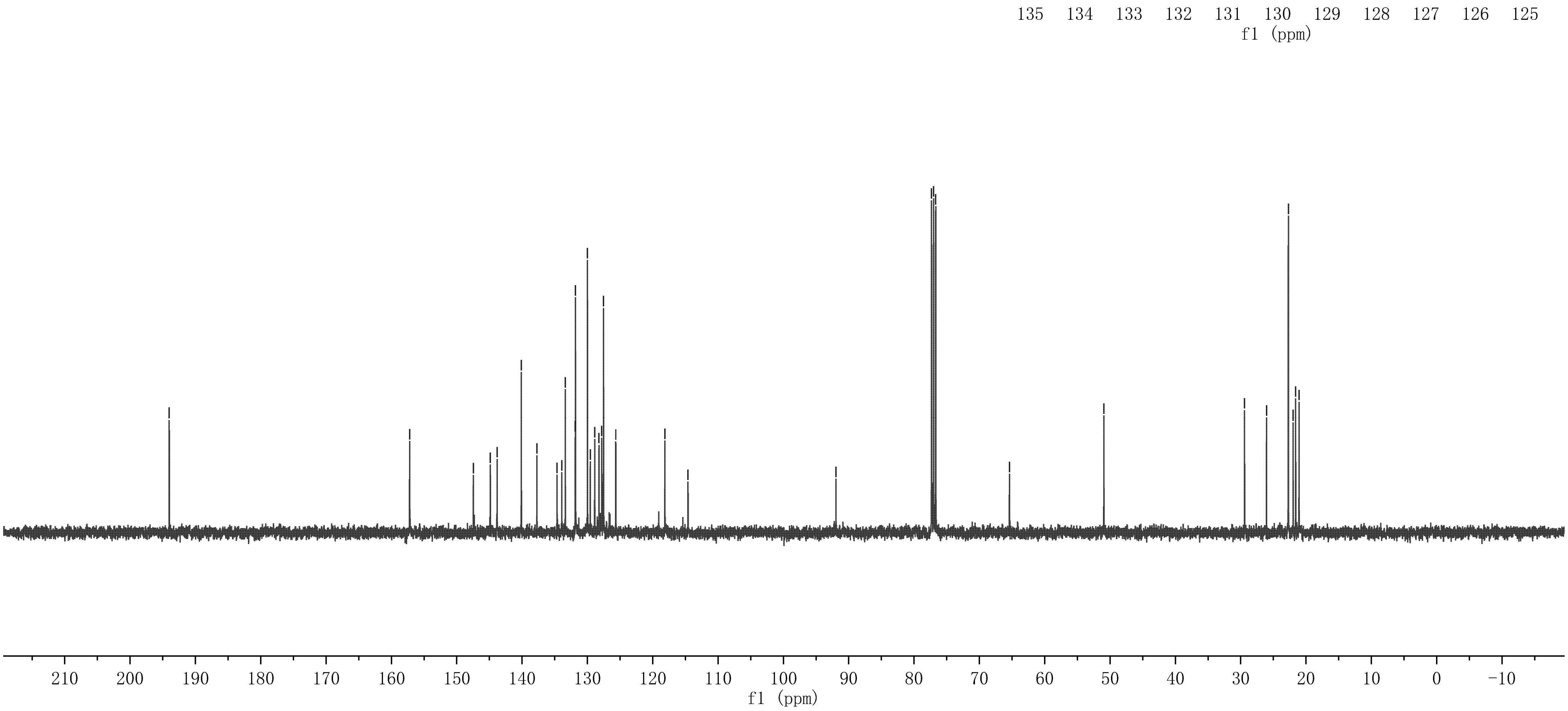

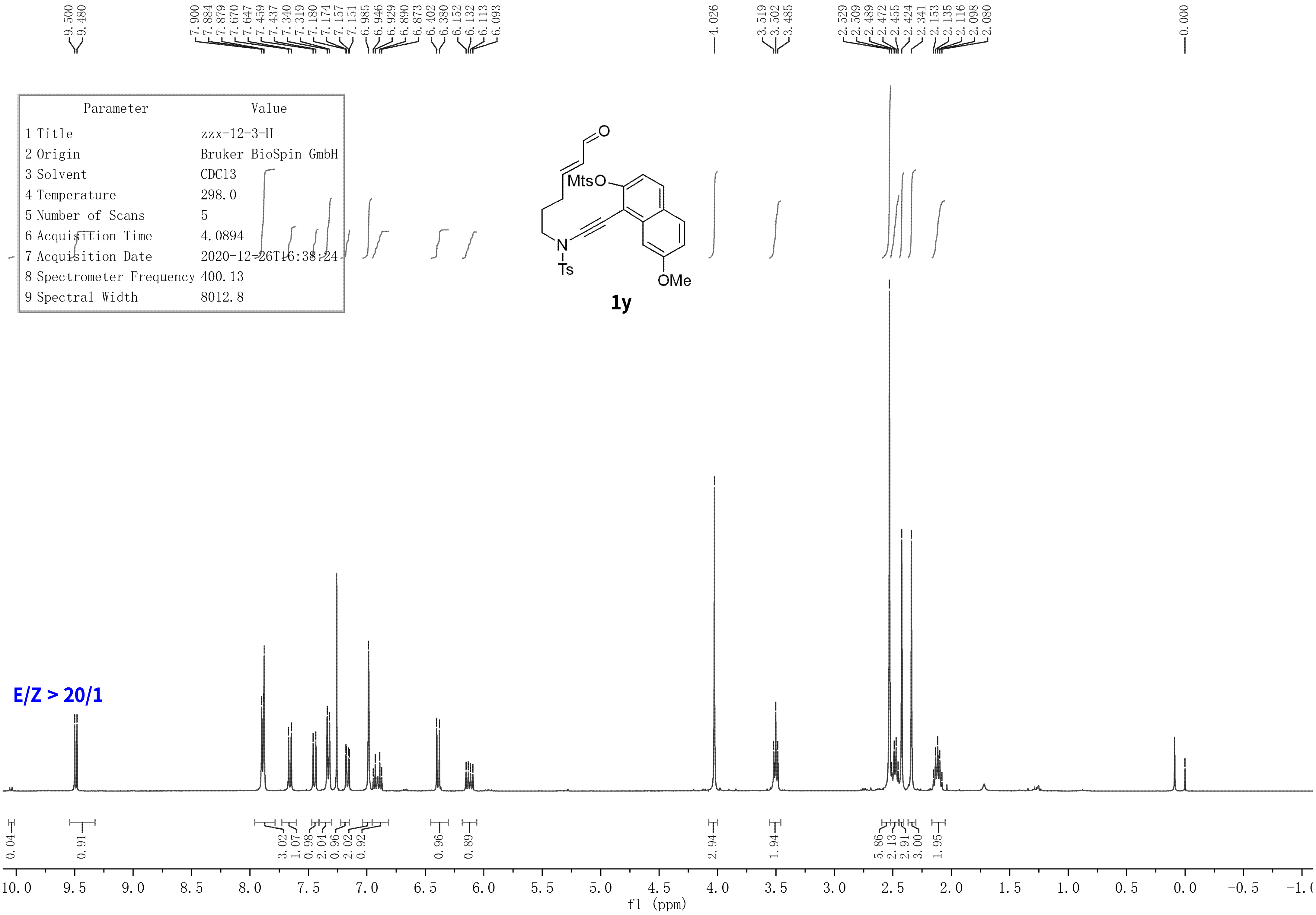
9.500
9.480
7.900
7.884
7.879
7.670
7.647
7.459
7.437
7.340
7.319
7.180
7.174
7.157
7.151
6.985
6.946
6.929
6.890
6.873
6.402
6.380
6.152
6.132
6.113
6.093
4.026
3.519
3.502
3.485
2.529
2.509
2.489
2.472
2.455
2.424
2.341
2.153
2.135
2.116
2.098
2.080
0.000
Parameter Value
1 Title zzx-12-3-H
2 Origin Bruker BioSpin GmbH
3 Solvent CDCl3
4 Temperature 298.0
5 Number of Scans 5
6 Acquisition Time 4.0894
7 Acquisition Date 2020-12-26T16:38:24
8 Spectrometer Frequency 400.13
9 Spectral Width 8012.8
O
MtsO
N
Ts
OMe
1y
E/Z > 20/1
0.04
0.91
3.02
1.07
0.98
2.04
0.96
2.02
0.92
0.96
0.89
2.94
1.94
5.86
2.13
2.91
3.00
1.95
10.0 9.5 9.0 8.5 8.0 7.5 7.0 6.5 6.0 5.5 5.0 4.5 4.0 3.5 3.0 2.5 2.0 1.5 1.0 0.5 0.0 -0.5 -1.0
f1 (ppm)

Parameter	Value
1 Title	zzx-12-3-C
2 Origin	Bruker BioSpin GmbH
3 Solvent	CDCl3
4 Temperature	300.0
5 Number of Scans	28
6 Acquisition Time	1.3631
7 Acquisition Date	2020-12-26T16:40:28
8 Spectrometer Frequency	100.61
9 Spectral Width	24038.5

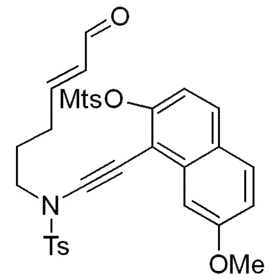

1y

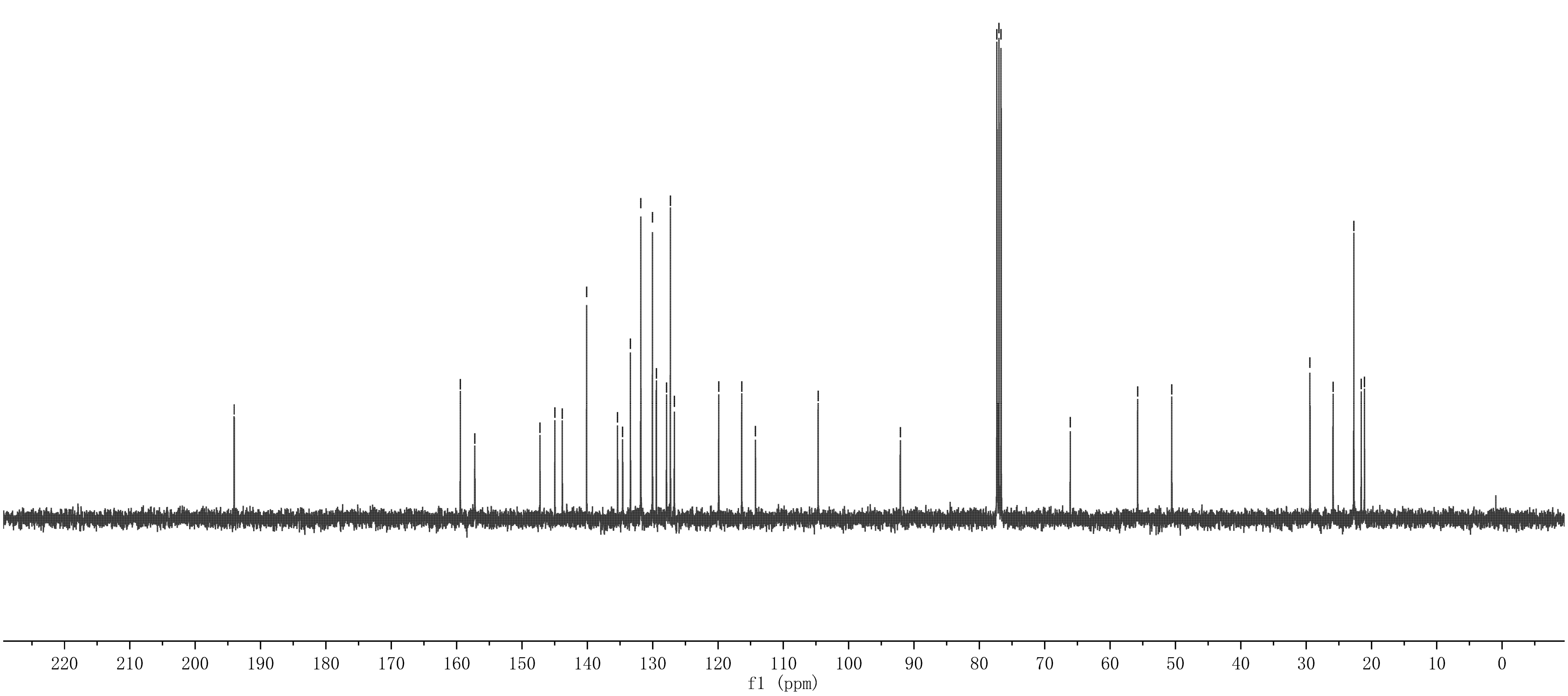

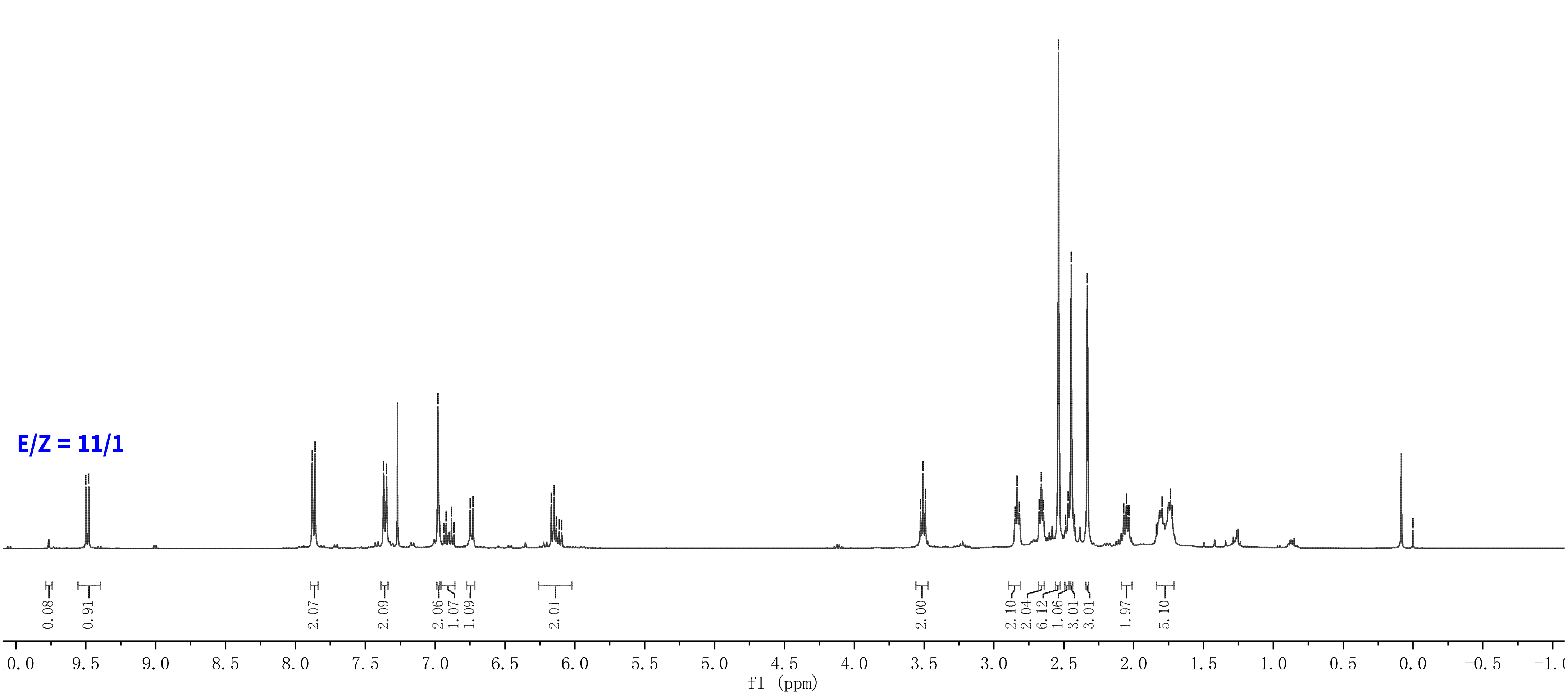

Parameter	Value
1 Title	zzx-12-2-H
2 Origin	Bruker BioSpin GmbH
3 Solvent	CDCl3
4 Temperature	298.0
5 Number of Scans	9
6 Acquisition Time	4.0894
7 Acquisition Date	2020-12-26T16:31:28
8 Spectrometer Frequency	400.13
9 Spectral Width	8012.8

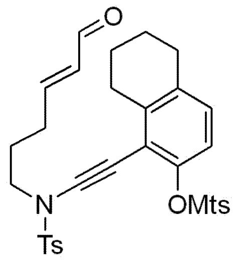

1z

Parameter	Value
1 Title	zzx-12-2-C
2 Origin	Bruker BioSpin GmbH
3 Solvent	CDCl3
4 Temperature	300.0
5 Number of Scans	40
6 Acquisition Time	1.3631
7 Acquisition Date	2020-12-26T16:34:21
8 Spectrometer Frequency	100.61
9 Spectral Width	24038.5

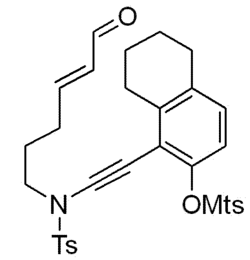

1z

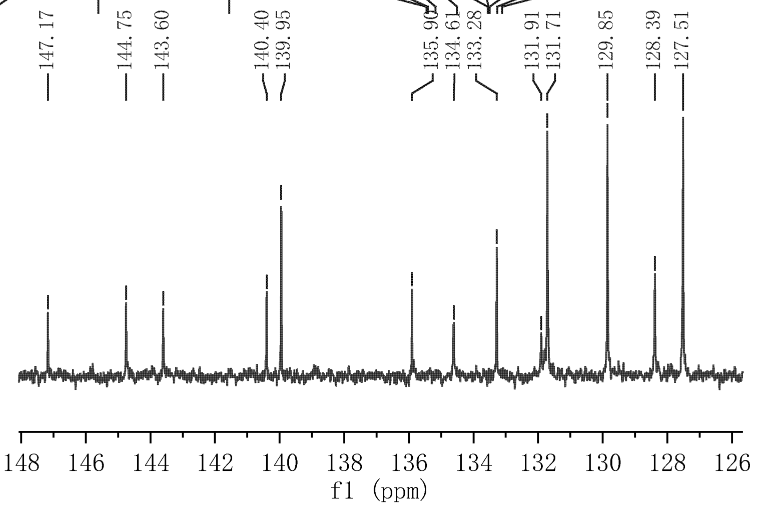

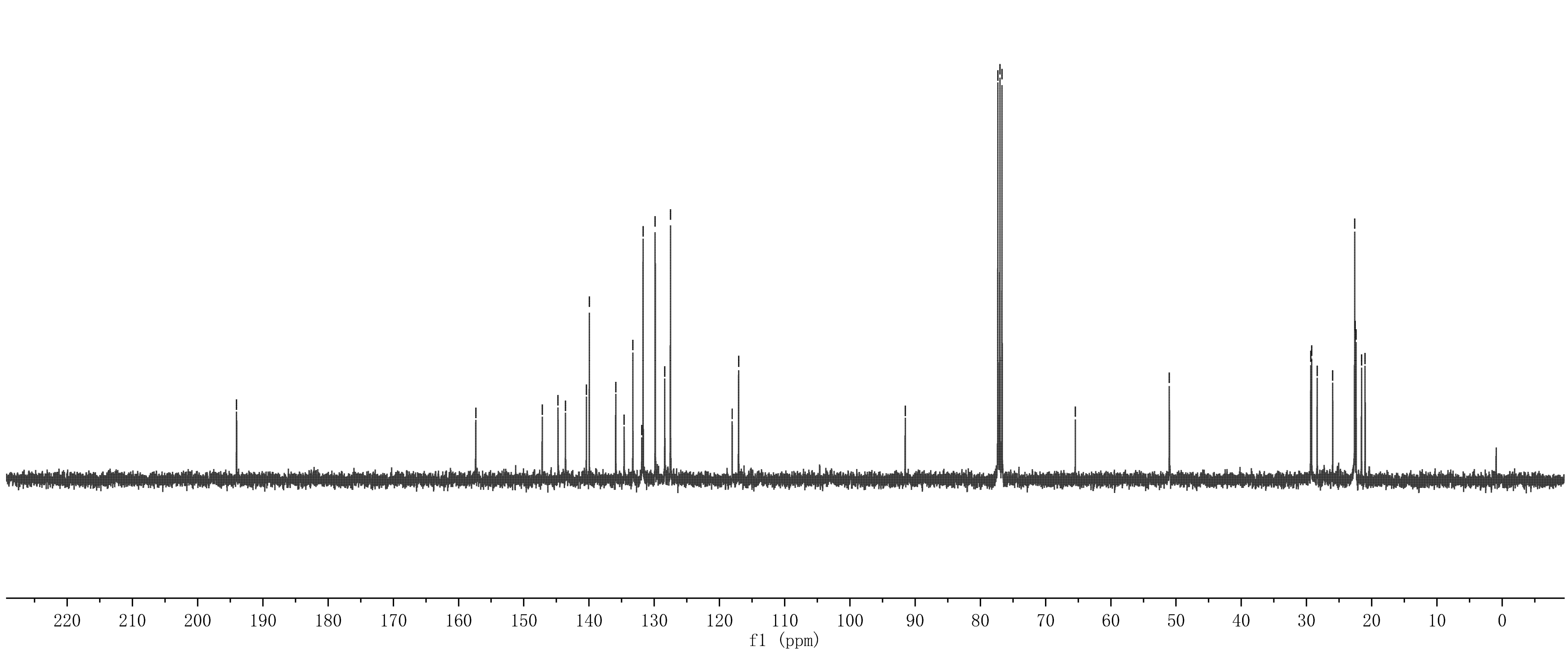

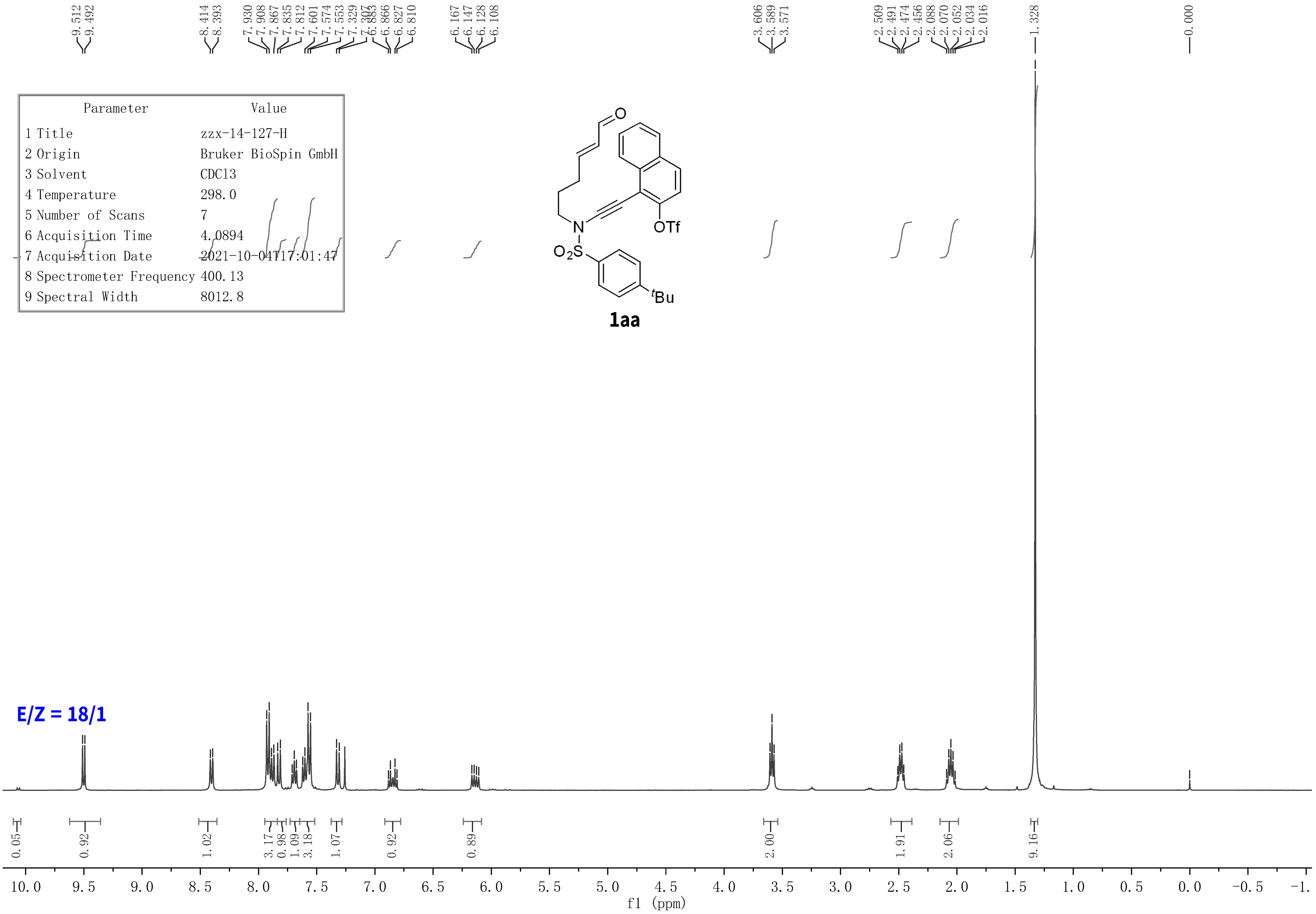

Parameter	Value
1 Title	zzx-14-127-H
2 Origin	Bruker BioSpin GmbH
3 Solvent	CDCl3
4 Temperature	298.0
5 Number of Scans	7
6 Acquisition Time	4.0894
7 Acquisition Date	2021-10-04T17:01:47
8 Spectrometer Frequency	400.13
9 Spectral Width	8012.8

1aa

Parameter	Value
1 Title	zzx-14-127-C
2 Origin	Bruker BioSpin GmbH
3 Solvent	CDCl3
4 Temperature	300.0
5 Number of Scans	23
6 Acquisition Time	1.3631
7 Acquisition Date	2021-10-04T17:03:37
8 Spectrometer Frequency	100.61
9 Spectral Width	24038.5

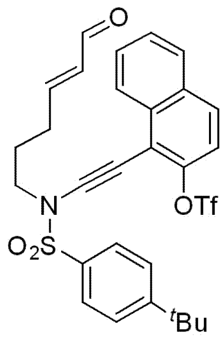

1aa

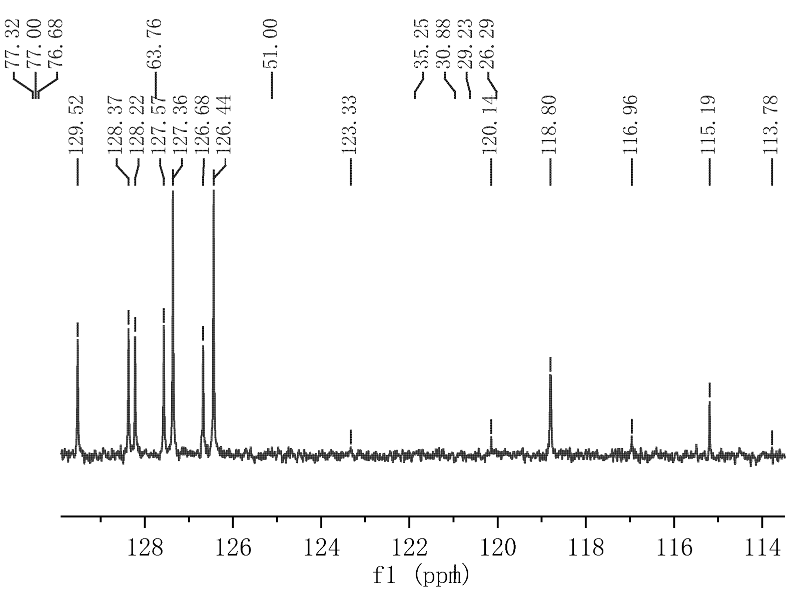

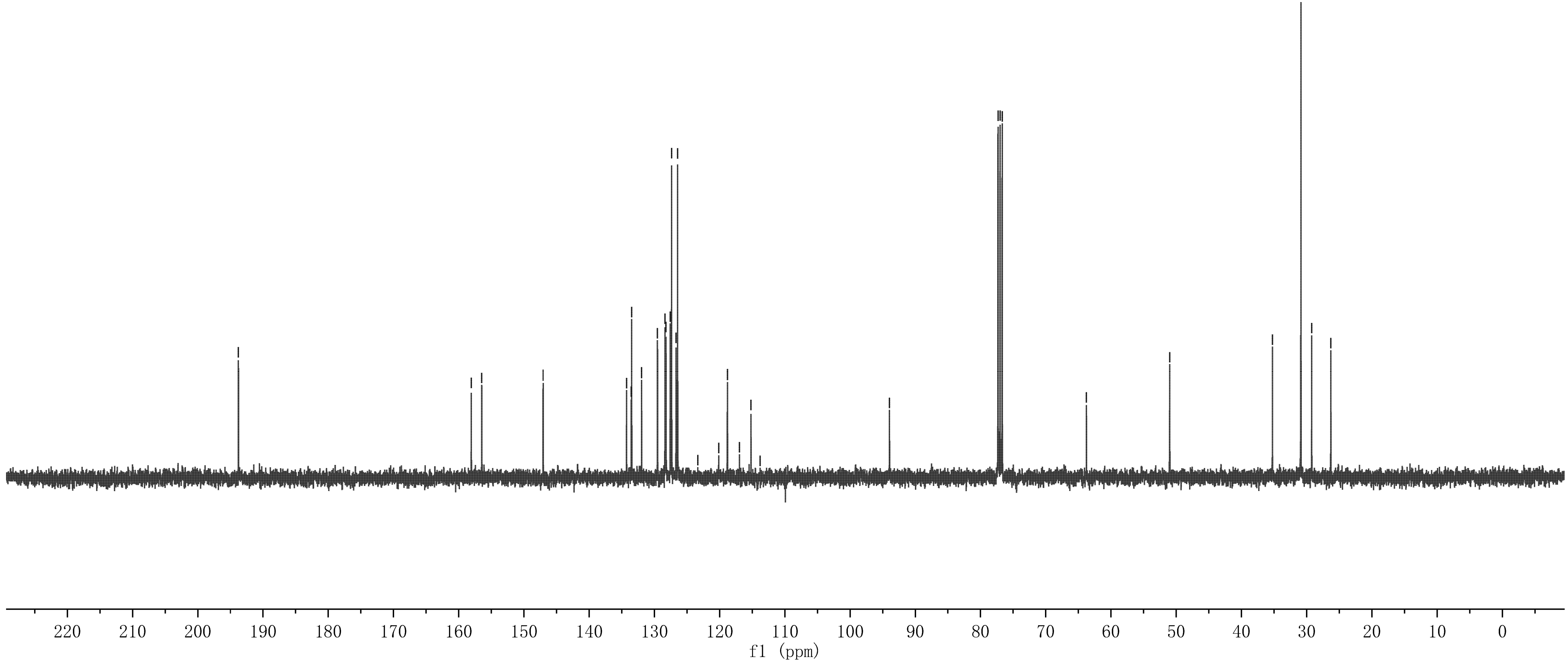

	Parameter	Value
1	Title	zzx-14-127-F
2	Origin	Bruker BioSpin GmbH
3	Solvent	CDC13
4	Temperature	301.2
5	Number of Scans	16
6	Acquisition Time	0.5767
7	Acquisition Date	2021-10-05T15:26:00
8	Spectrometer Frequency	470.63
9	Spectral Width	113636.4

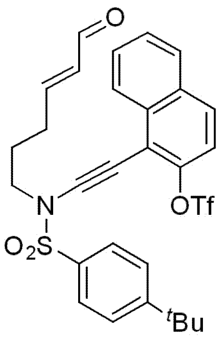

1aa

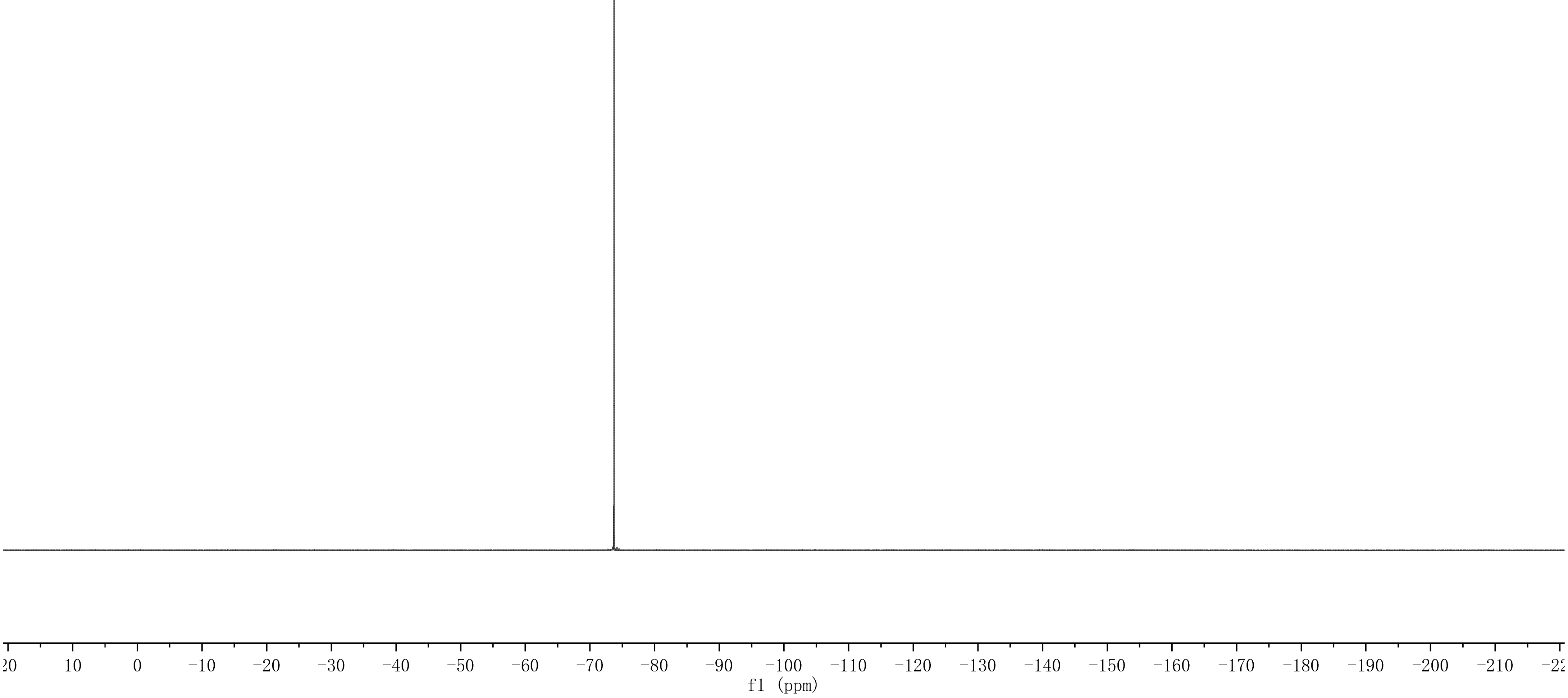

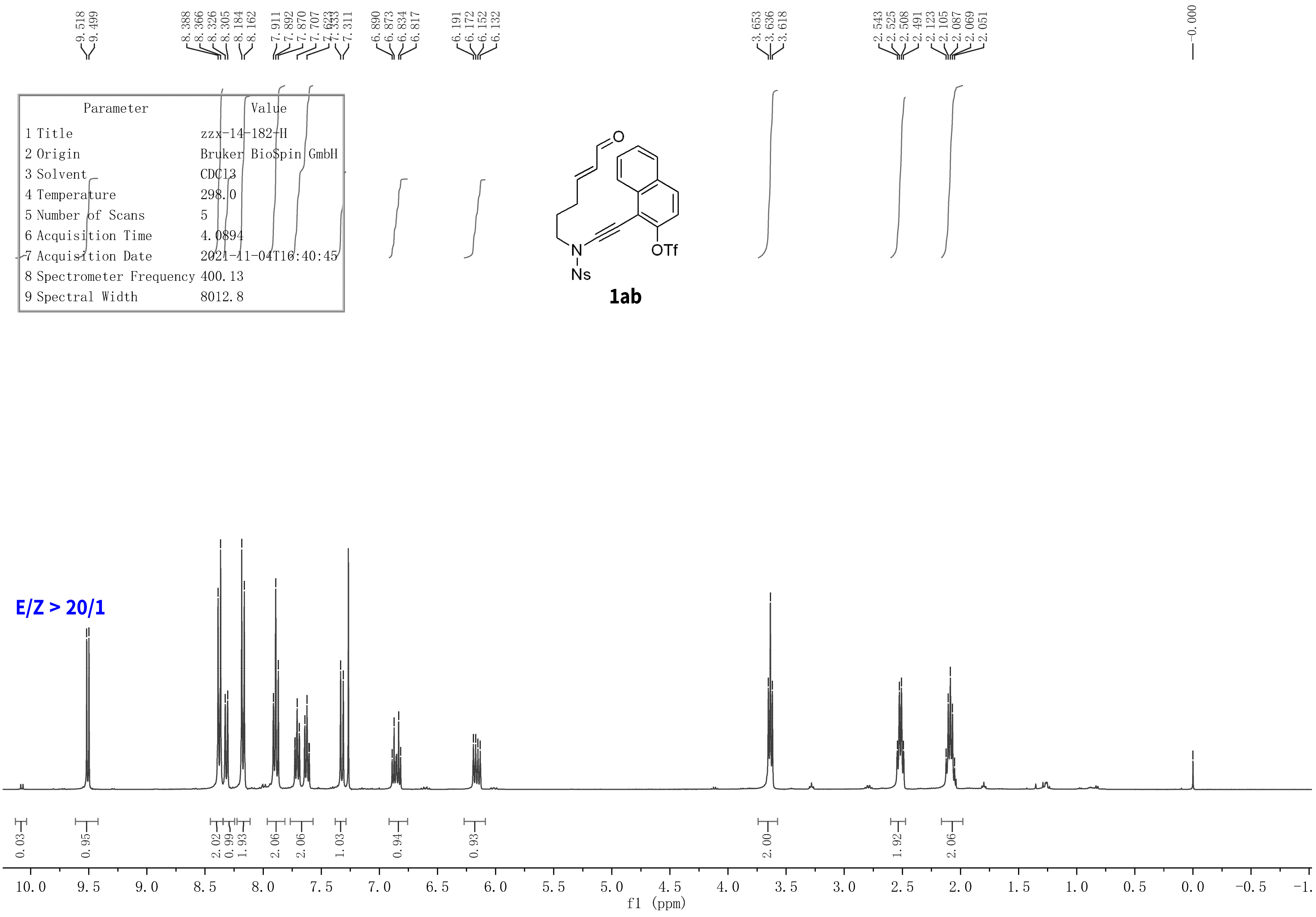

Parameter Value
1 Title zzx-14-182-H
2 Origin Bruker BioSpin GmbH
3 Solvent CDCl3
4 Temperature 298.0
5 Number of Scans 5
6 Acquisition Time 4.0894
7 Acquisition Date 2021-11-04T16:40:45
8 Spectrometer Frequency 400.13
9 Spectral Width 8012.8
O
N
Ns
OTf
1ab
E/Z > 20/1
9.518 9.499 8.388 8.366 8.326 8.305 8.184 8.162 7.911 7.892 7.870 7.707 7.311 6.890 6.873 6.834 6.817 6.191 6.172 6.152 6.132 3.653 3.636 3.618 2.543 2.525 2.508 2.491 2.123 2.105 2.087 2.069 2.051 -0.000
0.03 0.95 2.02 0.99 1.93 2.06 2.06 1.03 0.94 0.93 2.00 1.92 2.06
10.0 9.5 9.0 8.5 8.0 7.5 7.0 6.5 6.0 5.5 5.0 4.5 4.0 3.5 3.0 2.5 2.0 1.5 1.0 0.5 0.0 -0.5 -1.
f1 (ppm)

Parameter	Value
1 Title	zzx-14-182-C
2 Origin	Bruker BioSpin GmbH
3 Solvent	CDCl3
4 Temperature	300.0
5 Number of Scans	21
6 Acquisition Time	1.3631
7 Acquisition Date	2021-11-04T16:42:05
8 Spectrometer Frequency	100.61
9 Spectral Width	24038.5

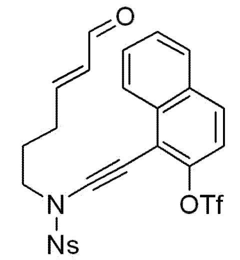

1ab

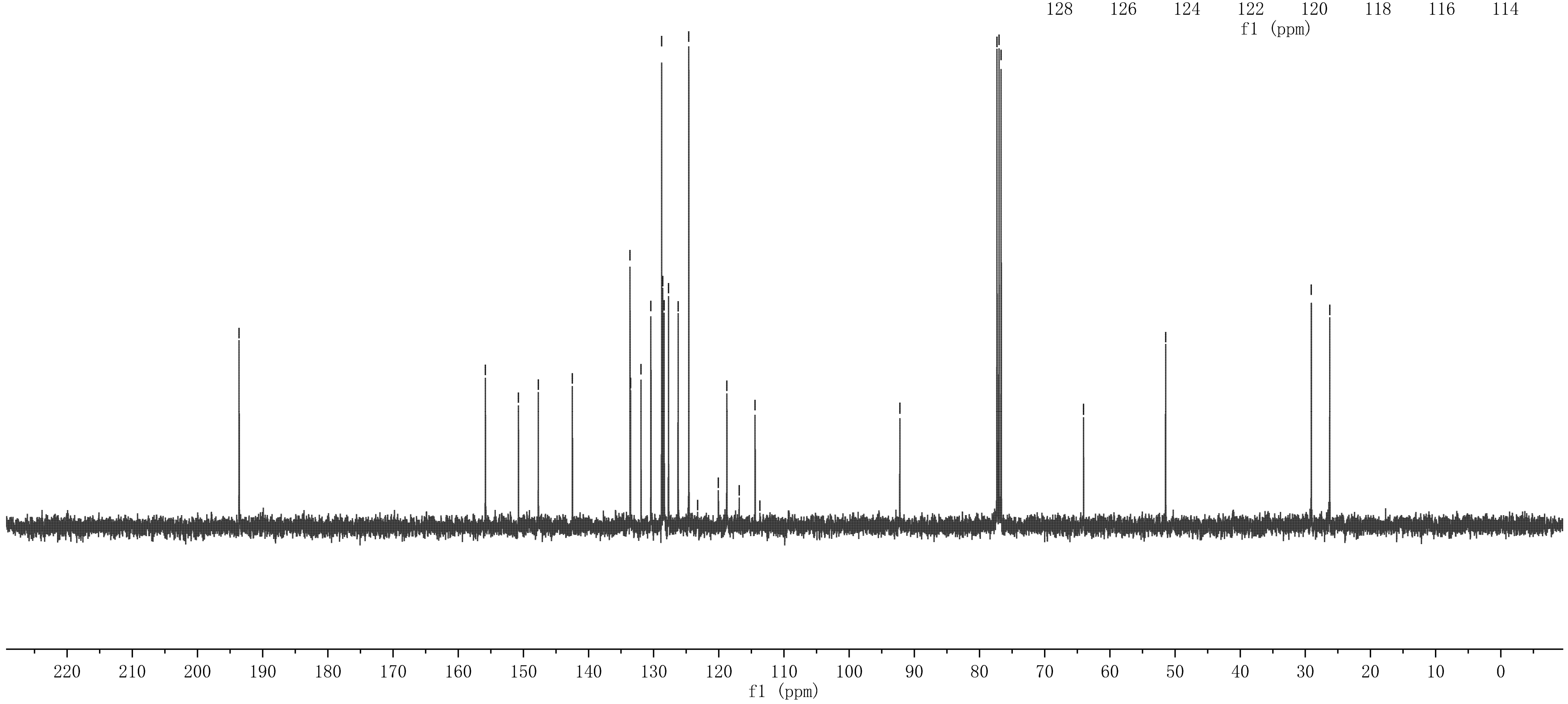

Parameter	Value
1 Title	ZZX-18-S-OTf-4-Ns
2 Origin	
3 Solvent	CDCl3
4 Temperature	297.5
5 Number of Scans	16
6 Acquisition Time	1.0000
7 Acquisition Date	2023-02-09T11:11:17
8 Spectrometer Frequency	376.28
9 Spectral Width	96153.0

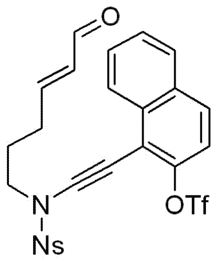

1ab

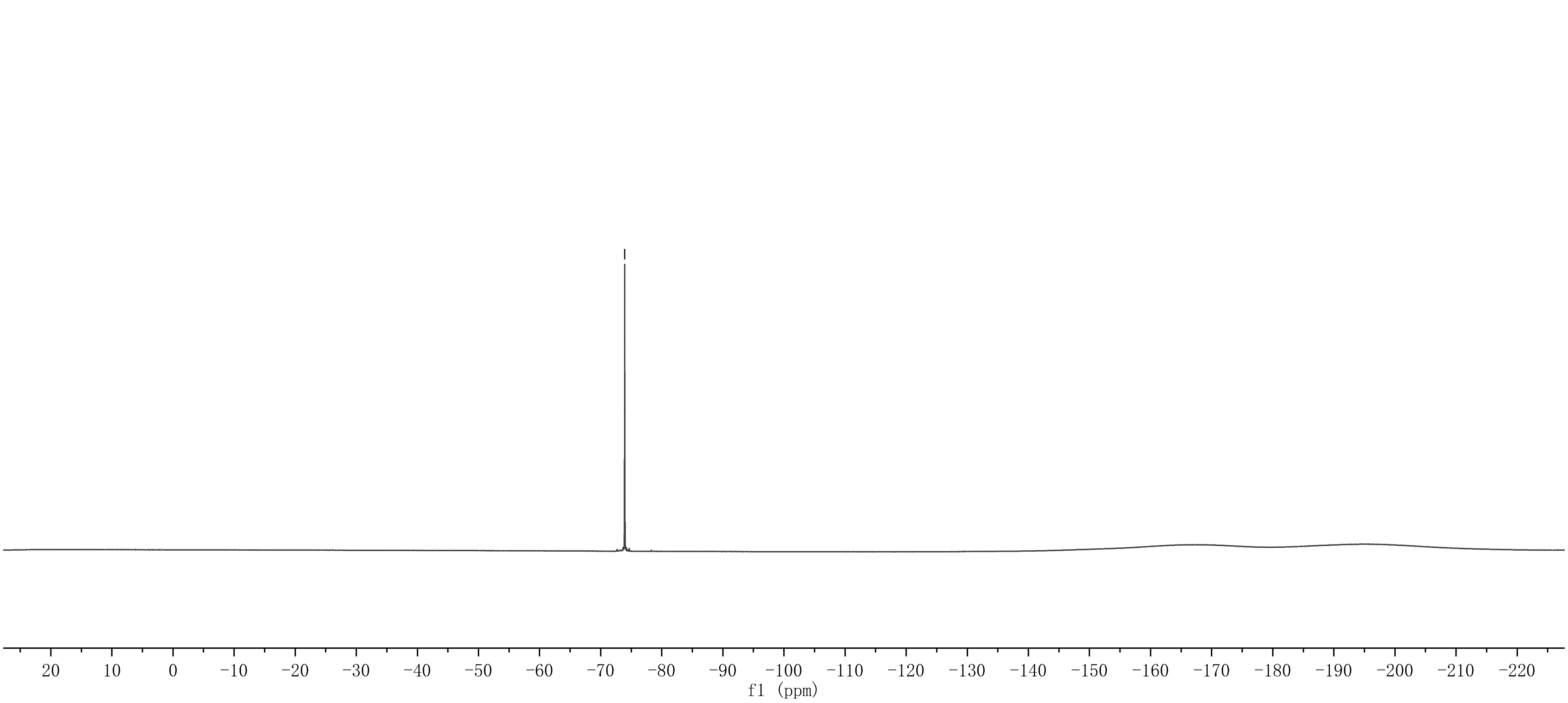

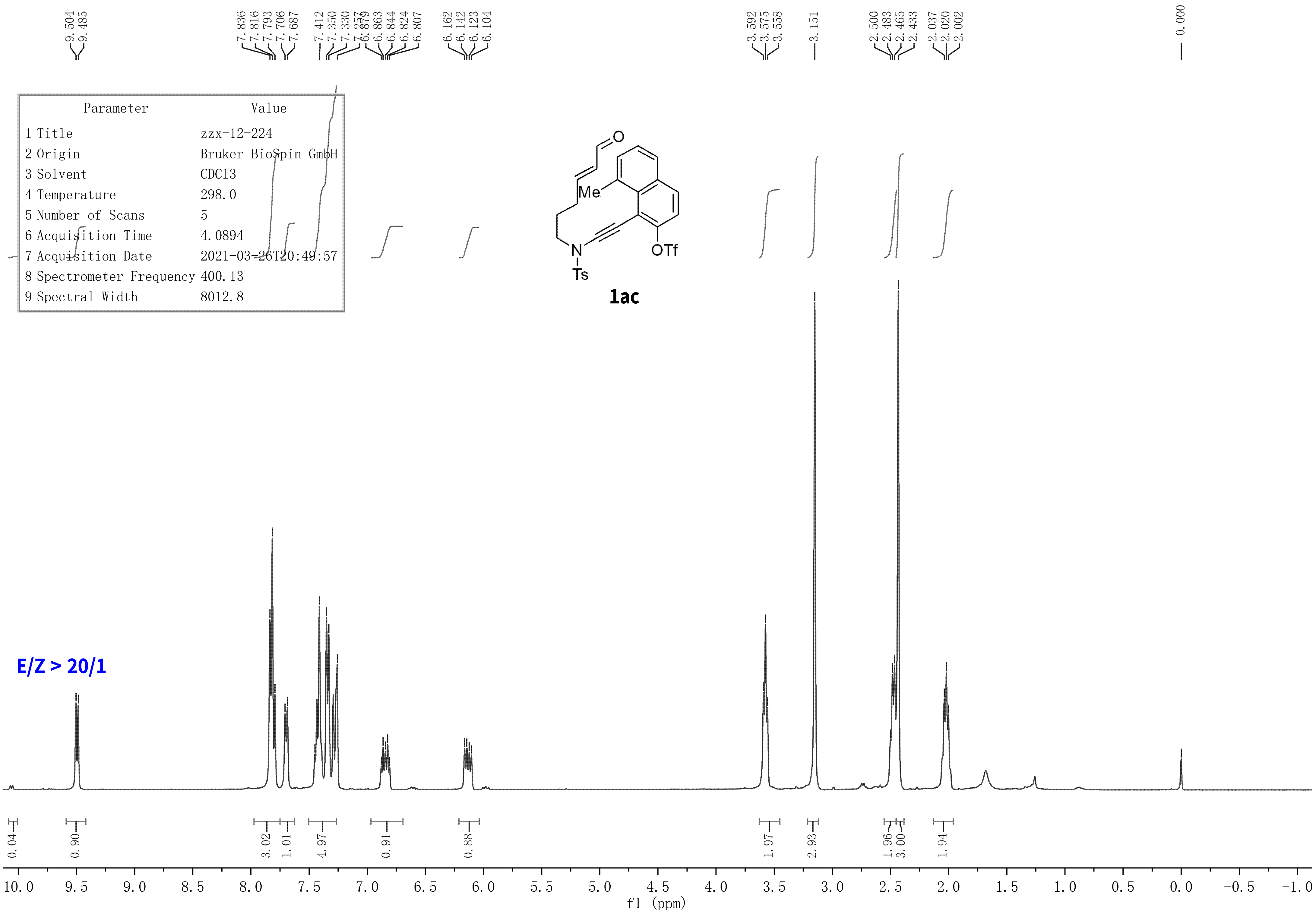

9.504
9.485
7.836
7.816
7.793
7.706
7.687
7.412
7.350
7.330
7.257
6.879
6.863
6.844
6.824
6.807
6.162
6.142
6.123
6.104
3.592
3.575
3.558
3.151
2.500
2.483
2.465
2.433
2.037
2.020
2.002
-0.000
Parameter Value
1 Title zzx-12-224
2 Origin Bruker BioSpin GmbH
3 Solvent CDCl3
4 Temperature 298.0
5 Number of Scans 5
6 Acquisition Time 4.0894
7 Acquisition Date 2021-03-26T20:49:57
8 Spectrometer Frequency 400.13
9 Spectral Width 8012.8
O
Me
N
Ts
OTf
1ac
E/Z > 20/1
0.04
0.90
3.02
1.01
4.97
0.91
0.88
1.97
2.93
1.96
3.00
1.94
10.0 9.5 9.0 8.5 8.0 7.5 7.0 6.5 6.0 5.5 5.0 4.5 4.0 3.5 3.0 2.5 2.0 1.5 1.0 0.5 0.0 -0.5 -1.0
f1 (ppm)

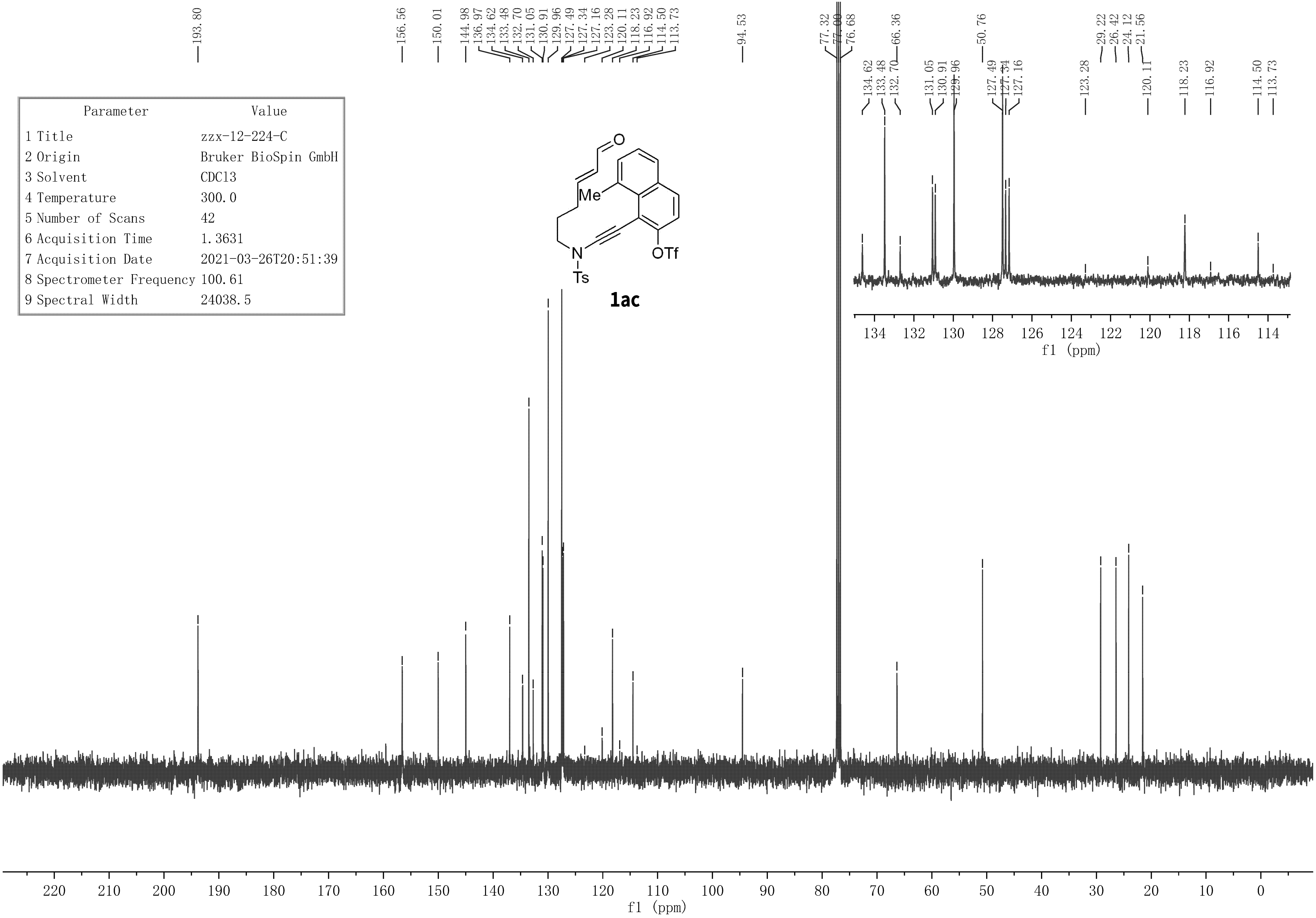

Parameter	Value
1 Title	zzx-12-224-C
2 Origin	Bruker BioSpin GmbH
3 Solvent	CDCl3
4 Temperature	300.0
5 Number of Scans	42
6 Acquisition Time	1.3631
7 Acquisition Date	2021-03-26T20:51:39
8 Spectrometer Frequency	100.61
9 Spectral Width	24038.5

1ac

Parameter	Value
1 Title	ZZX-18-S-OTf-8-Me
2 Origin	
3 Solvent	CDCl3
4 Temperature	297.5
5 Number of Scans	16
6 Acquisition Time	1.0000
7 Acquisition Date	2023-02-09T11:19:43
8 Spectrometer Frequency	376.28
9 Spectral Width	96153.0

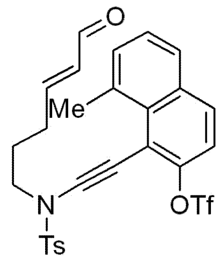

1ac

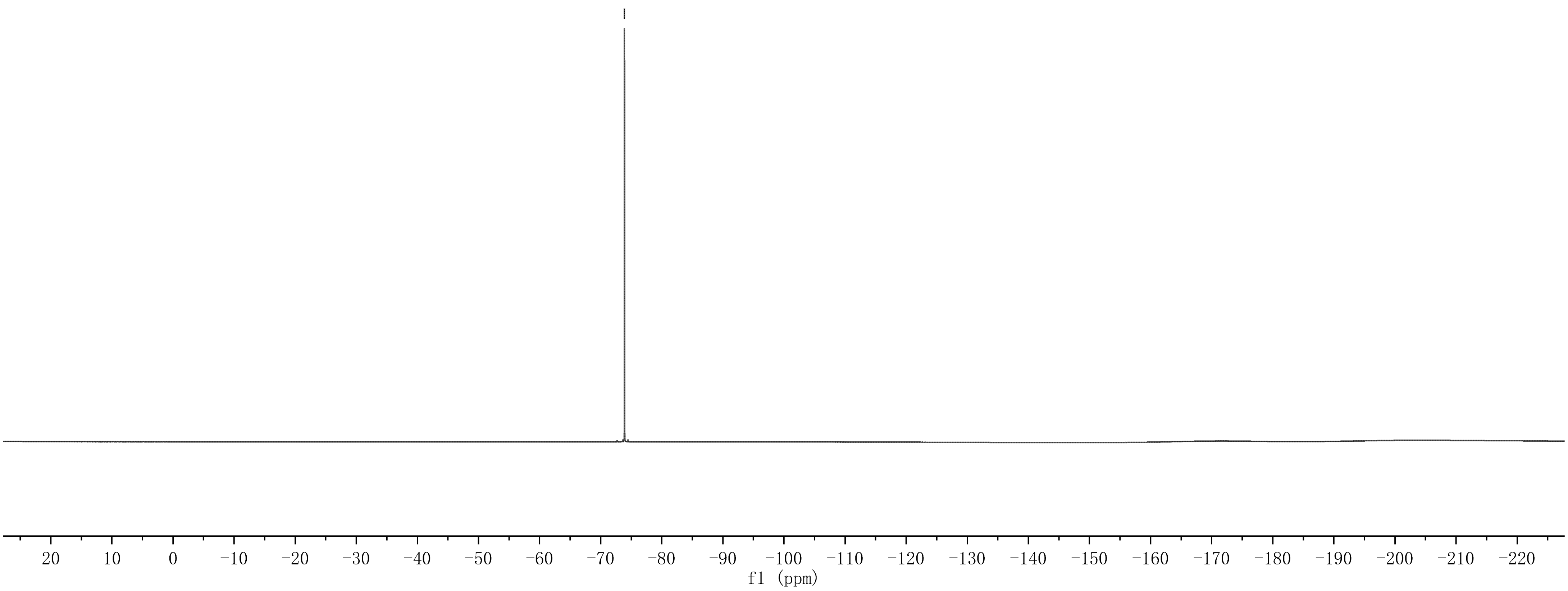

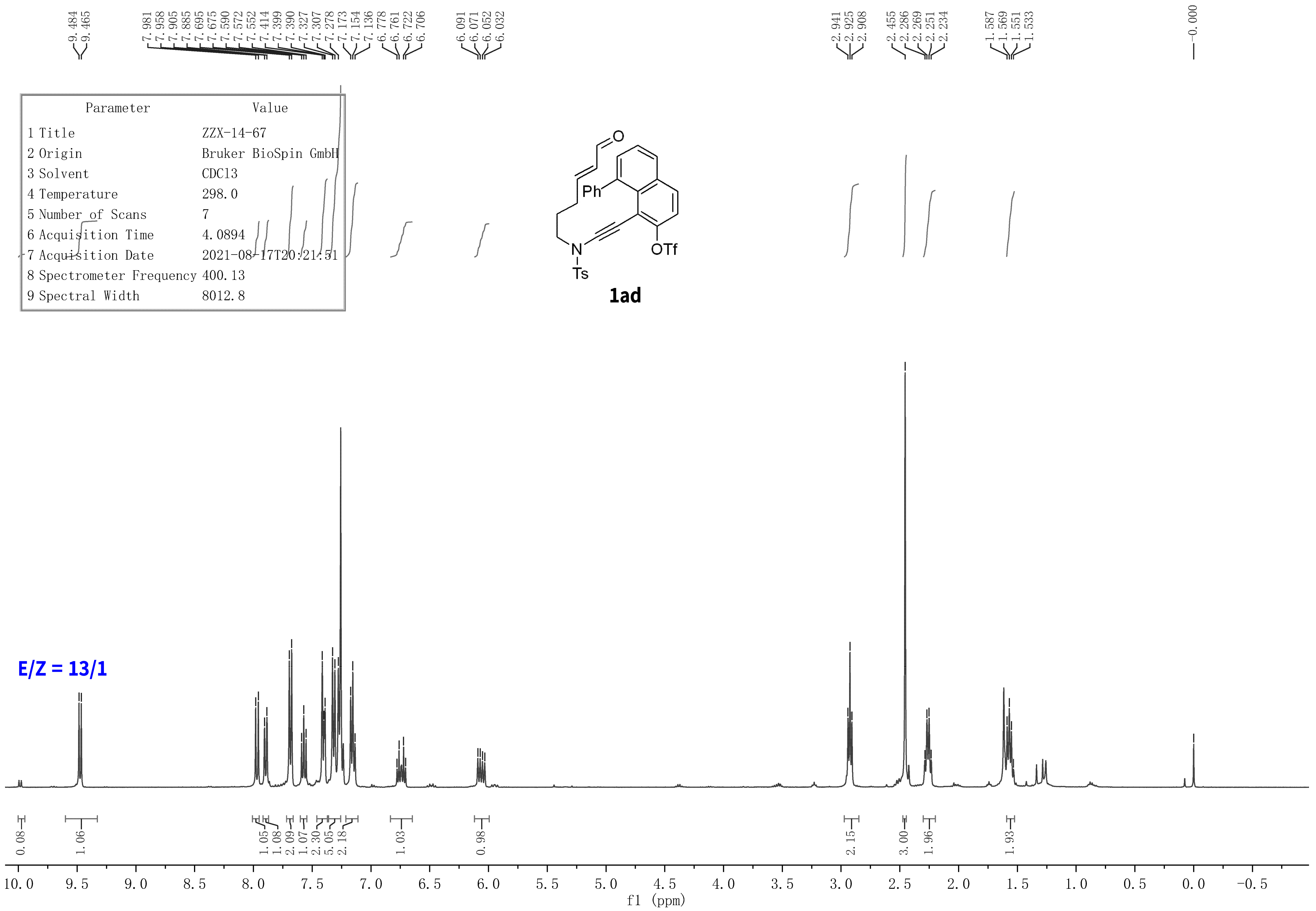
9.484
9.465
7.981
7.958
7.905
7.885
7.695
7.675
7.590
7.572
7.552
7.414
7.399
7.390
7.327
7.307
7.278
7.173
7.154
7.136
6.778
6.761
6.722
6.706
6.091
6.071
6.052
6.032
2.941
2.925
2.908
2.455
2.286
2.269
2.251
2.234
1.587
1.569
1.551
1.533
0.000
Parameter Value
1 Title ZZX-14-67
2 Origin Bruker BioSpin GmbH
3 Solvent CDCl3
4 Temperature 298.0
5 Number of Scans 7
6 Acquisition Time 4.0894
7 Acquisition Date 2021-08-17T20:21:51
8 Spectrometer Frequency 400.13
9 Spectral Width 8012.8
O
Ph
N
Ts
OTf
1ad
E/Z = 13/1
0.08
1.06
1.05
1.08
2.09
1.07
2.30
5.05
2.18
1.03
0.98
2.15
3.00
1.96
1.93
10.0 9.5 9.0 8.5 8.0 7.5 7.0 6.5 6.0 5.5 5.0 4.5 4.0 3.5 3.0 2.5 2.0 1.5 1.0 0.5 0.0 -0.5
f1 (ppm)

Parameter	Value
1 Title	ZZX-14-67-C
2 Origin	Bruker BioSpin GmbH
3 Solvent	CDCl3
4 Temperature	300.0
5 Number of Scans	81
6 Acquisition Time	1.3631
7 Acquisition Date	2021-08-17T20:23:44
8 Spectrometer Frequency	100.61
9 Spectral Width	24038.5

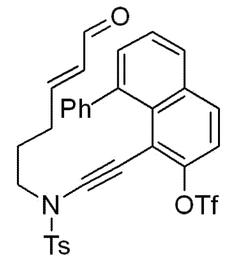

1ad

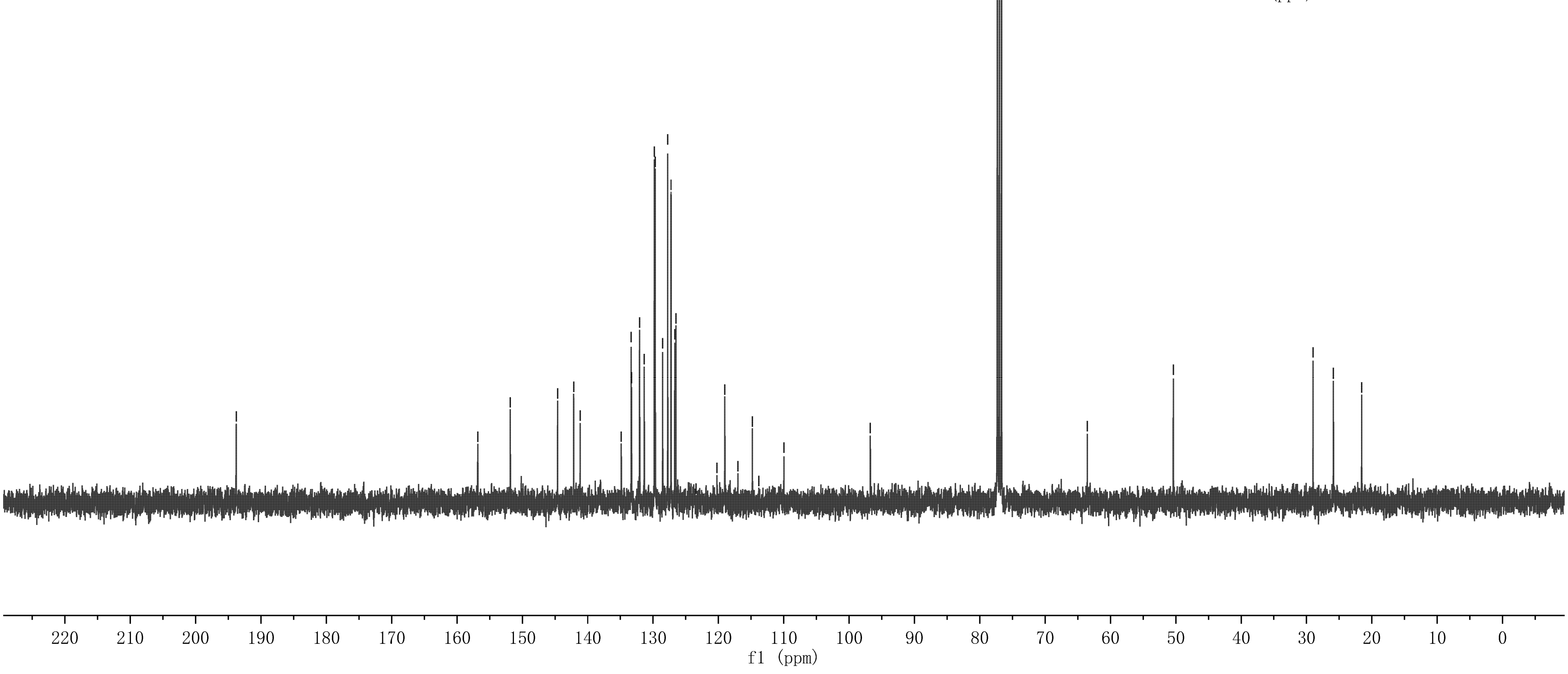

Parameter	Value
1 Title	ZZX-18-S-OTf-8-Ph
2 Origin	
3 Solvent	CDCl3
4 Temperature	297.5
5 Number of Scans	16
6 Acquisition Time	1.0000
7 Acquisition Date	2023-02-09T11:27:31
8 Spectrometer Frequency	376.28
9 Spectral Width	96153.0

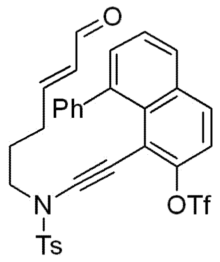

1ad

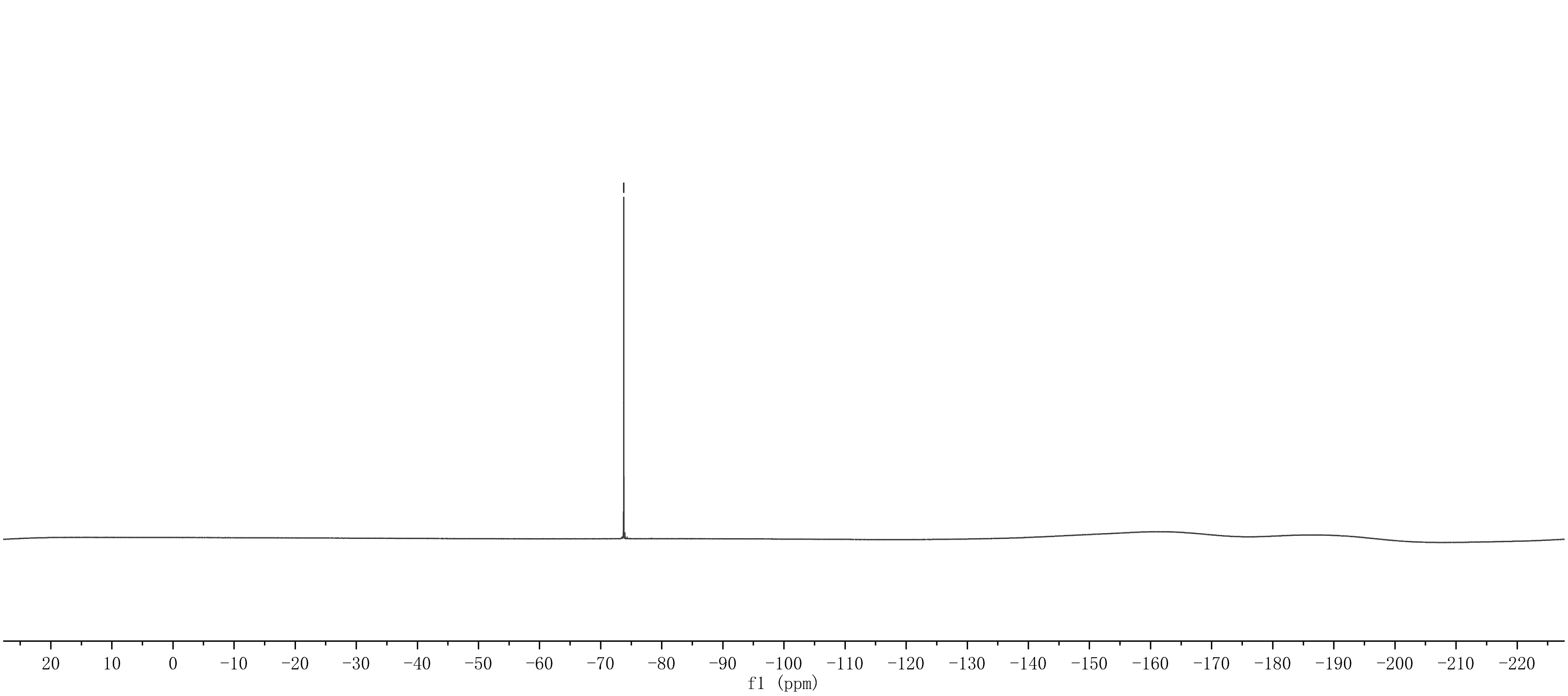

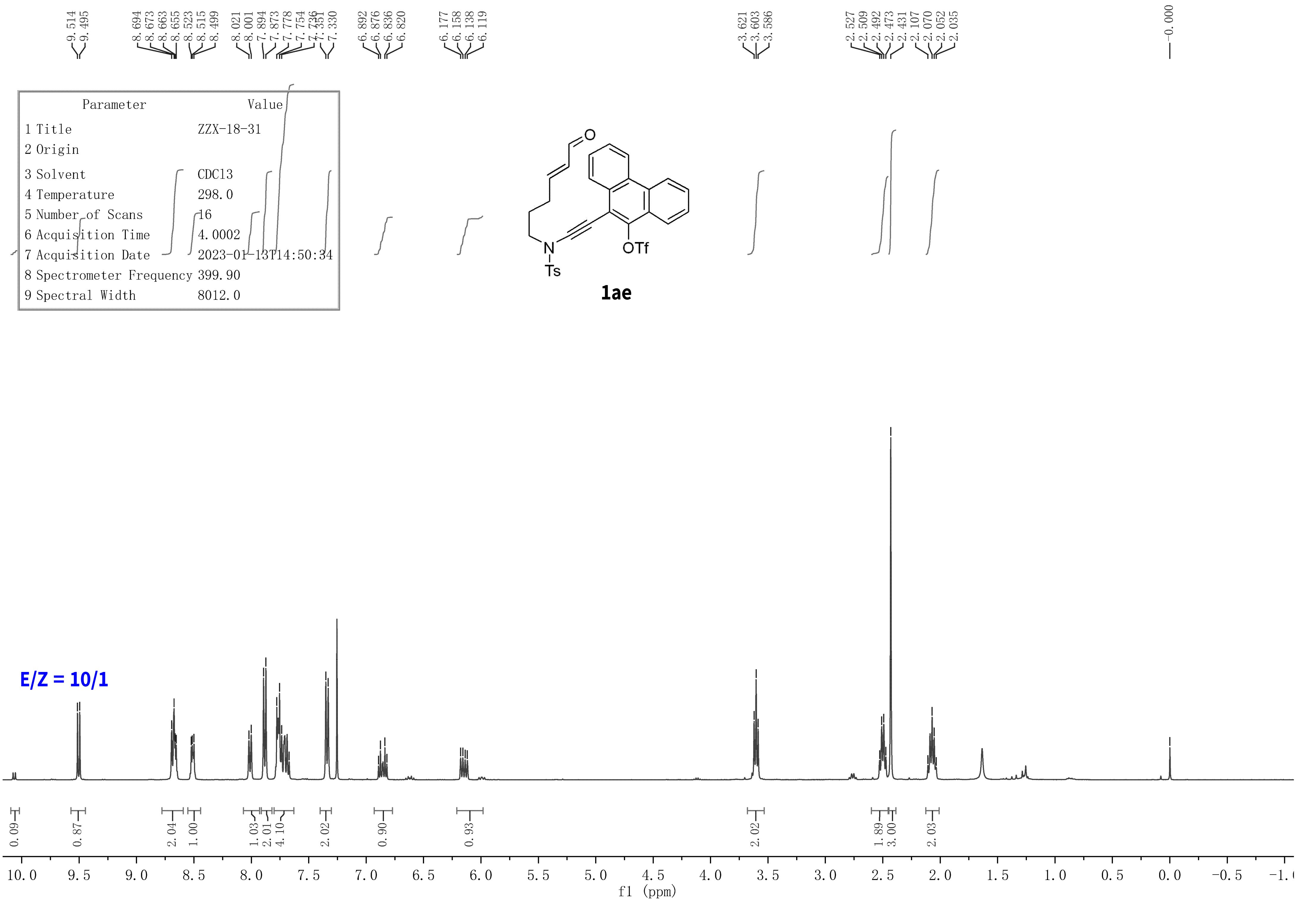

9.514
9.495
8.694
8.673
8.663
8.655
8.523
8.515
8.499
8.021
8.001
7.894
7.873
7.778
7.754
7.736
7.351
7.330
6.892
6.876
6.836
6.820
6.177
6.158
6.138
6.119
3.621
3.603
3.586
2.527
2.509
2.492
2.473
2.431
2.107
2.070
2.052
2.035
-0.000
Parameter Value
1 Title ZZX-18-31
2 Origin
3 Solvent CDCl3
4 Temperature 298.0
5 Number of Scans 16
6 Acquisition Time 4.0002
7 Acquisition Date 2023-01-13T14:50:34
8 Spectrometer Frequency 399.90
9 Spectral Width 8012.0
O
N
Ts
OTf
1ae
E/Z = 10/1
0.09
0.87
2.04
1.00
1.03
2.01
4.10
2.02
0.90
0.93
2.02
1.89
3.00
2.03
10.0 9.5 9.0 8.5 8.0 7.5 7.0 6.5 6.0 5.5 5.0 4.5 4.0 3.5 3.0 2.5 2.0 1.5 1.0 0.5 0.0 -0.5 -1.0
f1 (ppm)

Parameter	Value
1 Title	ZZX-18-31
2 Origin	
3 Solvent	CDCl3
4 Temperature	298.1
5 Number of Scans	600
6 Acquisition Time	1.0000
7 Acquisition Date	2023-01-13T15:13:18
8 Spectrometer Frequency	100.56
9 Spectral Width	26041.0

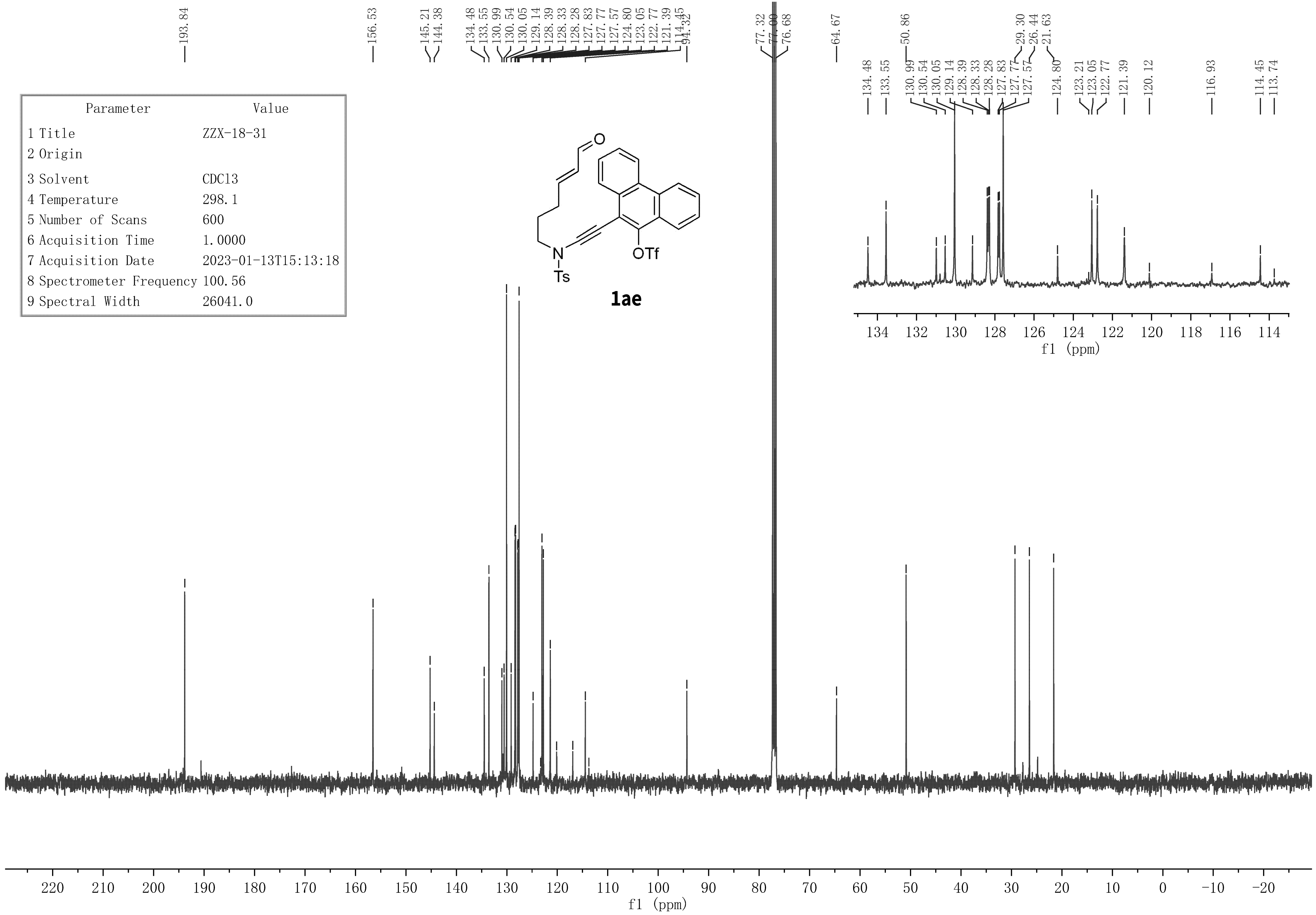

Parameter	Value
1 Title	ZZX-18-31
2 Origin	
3 Solvent	CDCl3
4 Temperature	297.8
5 Number of Scans	16
6 Acquisition Time	1.0000
7 Acquisition Date	2023-01-13T15:16:18
8 Spectrometer Frequency	376.28
9 Spectral Width	96153.0

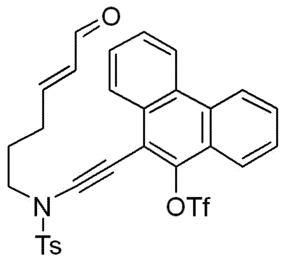

1ae

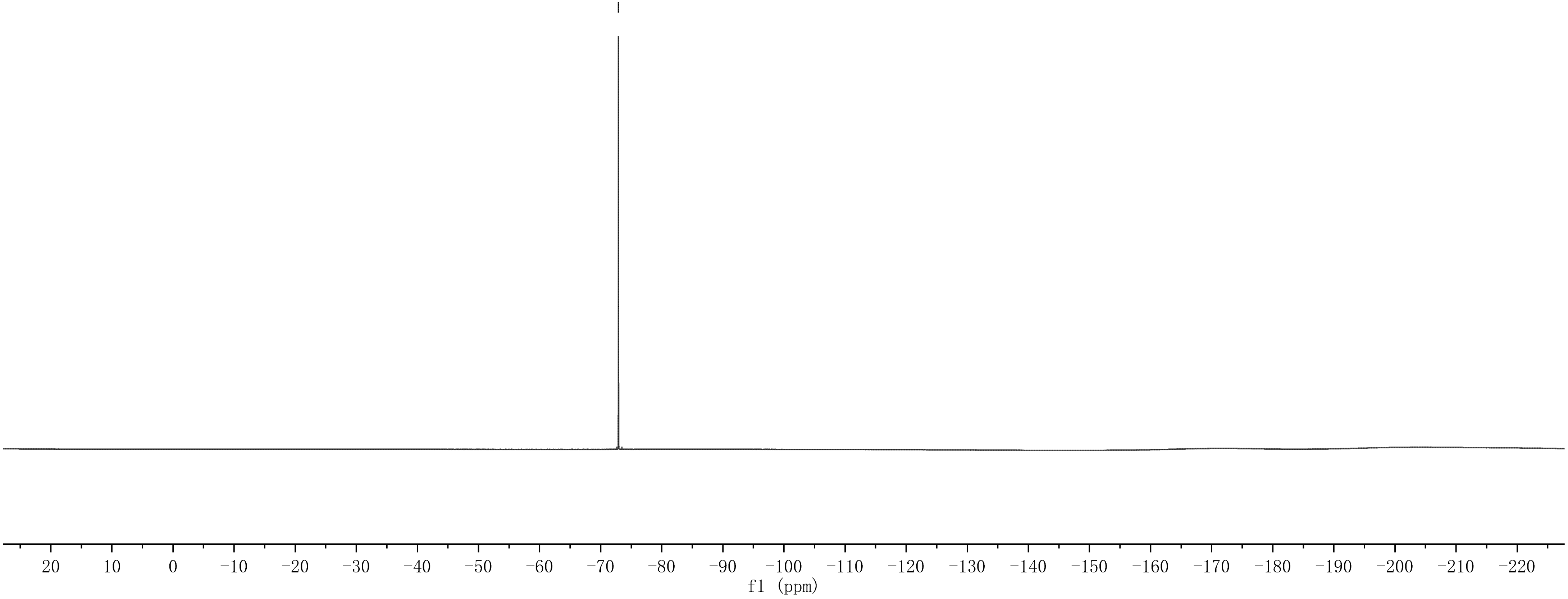

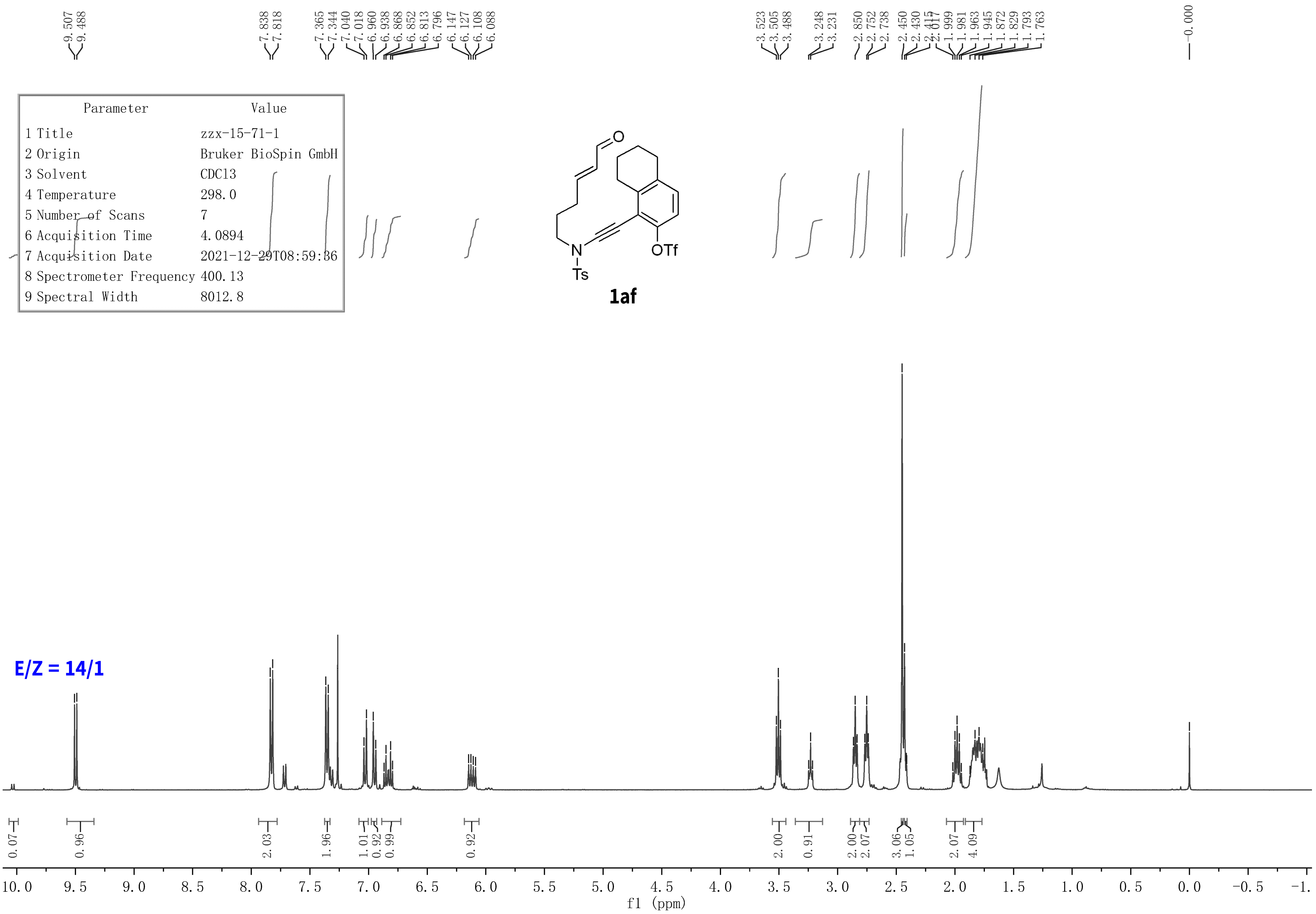

9.507
9.488
7.838
7.818
7.365
7.344
7.040
7.018
6.960
6.938
6.868
6.852
6.813
6.796
6.147
6.127
6.108
6.088
3.523
3.505
3.488
3.248
3.231
2.850
2.752
2.738
2.450
2.430
2.415
2.017
1.999
1.981
1.963
1.945
1.872
1.829
1.793
1.763
-0.000
Parameter Value
1 Title zzx-15-71-1
2 Origin Bruker BioSpin GmbH
3 Solvent CDCl3
4 Temperature 298.0
5 Number of Scans 7
6 Acquisition Time 4.0894
7 Acquisition Date 2021-12-29T08:59:36
8 Spectrometer Frequency 400.13
9 Spectral Width 8012.8
O
N
Ts
OTf
1af
E/Z = 14/1
0.07
0.96
2.03
1.96
1.01
0.92
0.99
0.92
2.00
0.91
2.00
2.07
3.06
1.05
2.07
4.09
10.0 9.5 9.0 8.5 8.0 7.5 7.0 6.5 6.0 5.5 5.0 4.5 4.0 3.5 3.0 2.5 2.0 1.5 1.0 0.5 0.0 -0.5 -1.
f1 (ppm)

Parameter	Value
1 Title	zzx-15-71-1-C
2 Origin	Bruker BioSpin GmbH
3 Solvent	CDCl3
4 Temperature	300.0
5 Number of Scans	106
6 Acquisition Time	1.3631
7 Acquisition Date	2021-12-29T09:01:08
8 Spectrometer Frequency	100.61
9 Spectral Width	24038.5

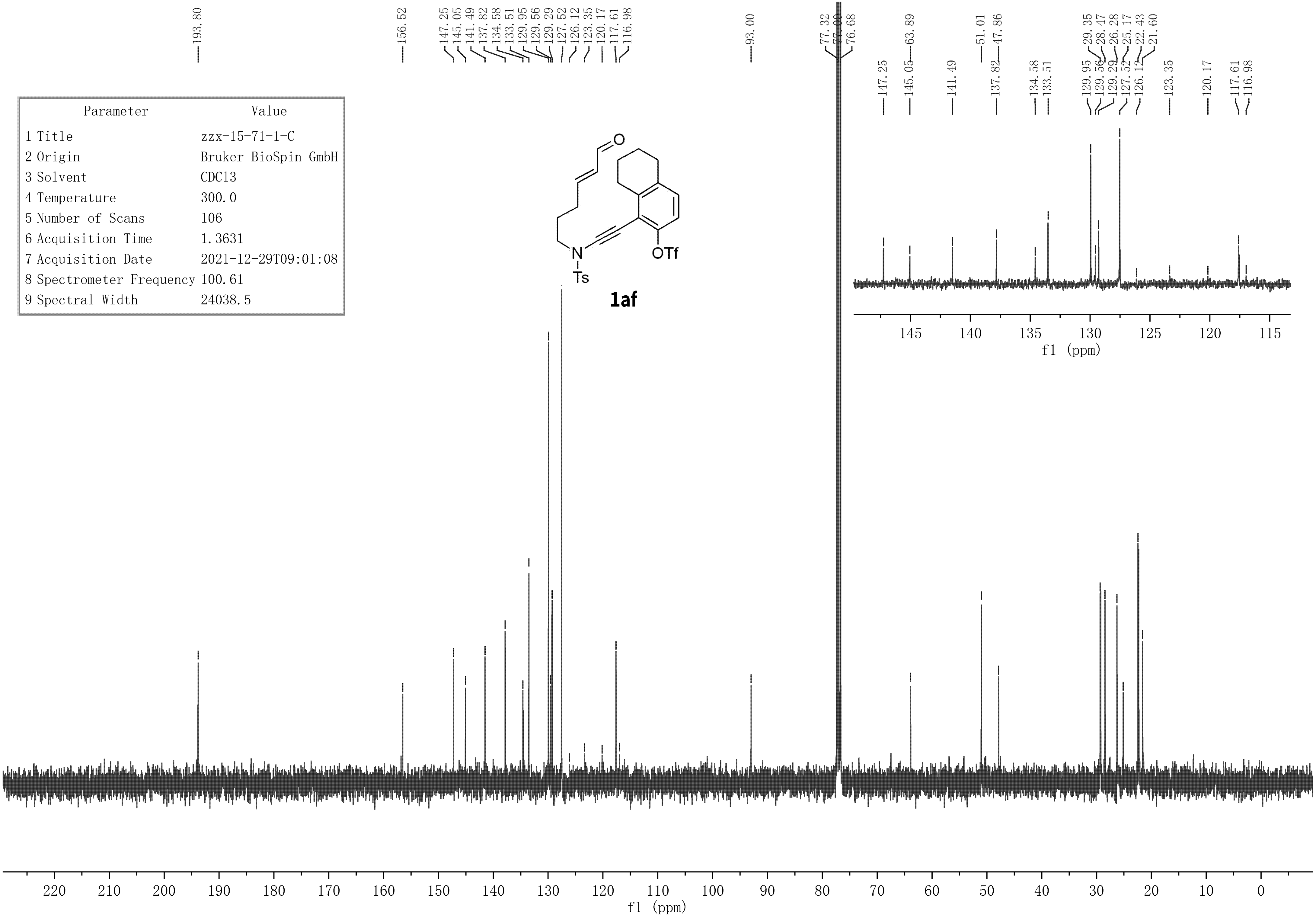

Parameter	Value
1 Title	ZZX-18-S-OTf-4H-naph
2 Origin	
3 Solvent	CDCl3
4 Temperature	297.5
5 Number of Scans	16
6 Acquisition Time	1.0000
7 Acquisition Date	2023-02-09T11:23:35
8 Spectrometer Frequency	376.28
9 Spectral Width	96153.0

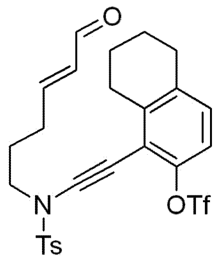

1af

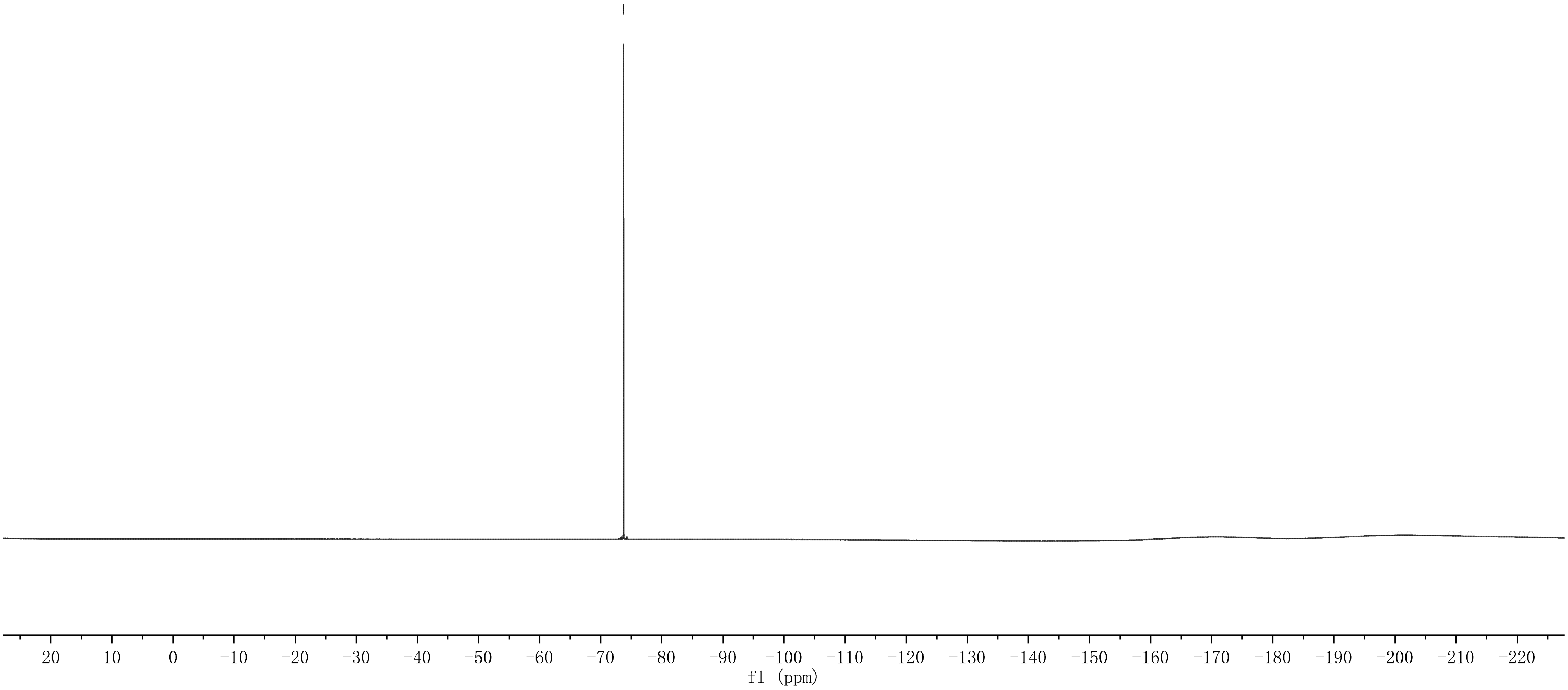

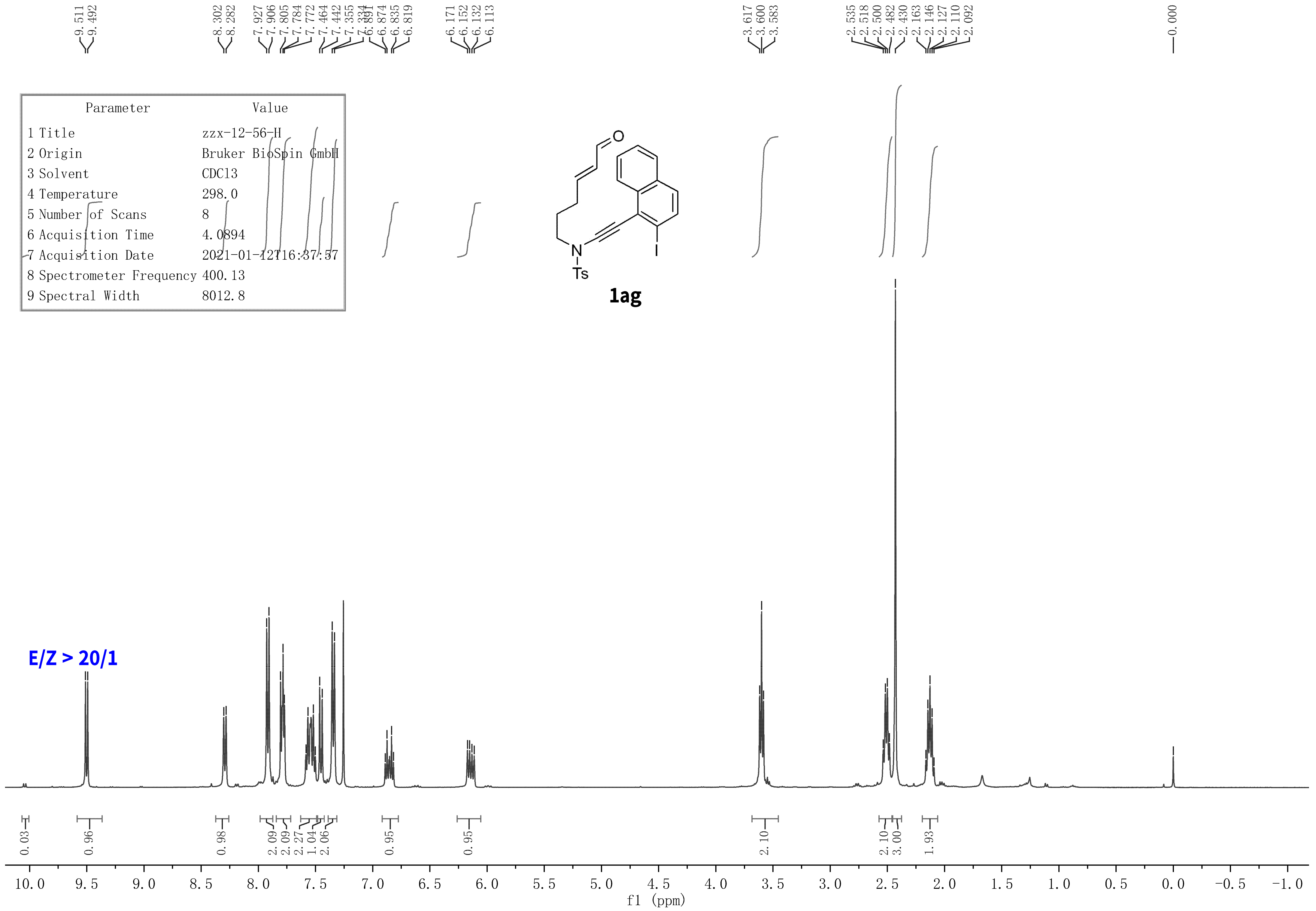
9.511
9.492
8.302
8.282
7.927
7.906
7.805
7.784
7.772
7.464
7.442
7.355
7.334
6.891
6.874
6.835
6.819
6.171
6.152
6.132
6.113
3.617
3.600
3.583
2.535
2.518
2.500
2.482
2.430
2.163
2.146
2.127
2.110
2.092
0.000
Parameter
Value
1 Title
zzx-12-56-H
2 Origin
Bruker BioSpin GmbH
3 Solvent
CDCl3
4 Temperature
298.0
5 Number of Scans
8
6 Acquisition Time
4.0894
7 Acquisition Date
2021-01-12T16:37:57
8 Spectrometer Frequency
400.13
9 Spectral Width
8012.8
O
N
Ts
I
1ag
E/Z > 20/1
0.03
0.96
0.98
2.09
2.09
2.27
1.04
2.06
0.95
0.95
2.10
2.10
3.00
1.93
f1 (ppm)

Parameter	Value
1 Title	zzx-12-56-C-1
2 Origin	Bruker BioSpin GmbH
3 Solvent	CDCl3
4 Temperature	300.0
5 Number of Scans	94
6 Acquisition Time	1.3631
7 Acquisition Date	2021-01-12T16:41:50
8 Spectrometer Frequency	100.61
9 Spectral Width	24038.5

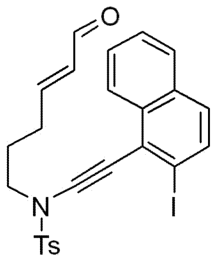

1ag

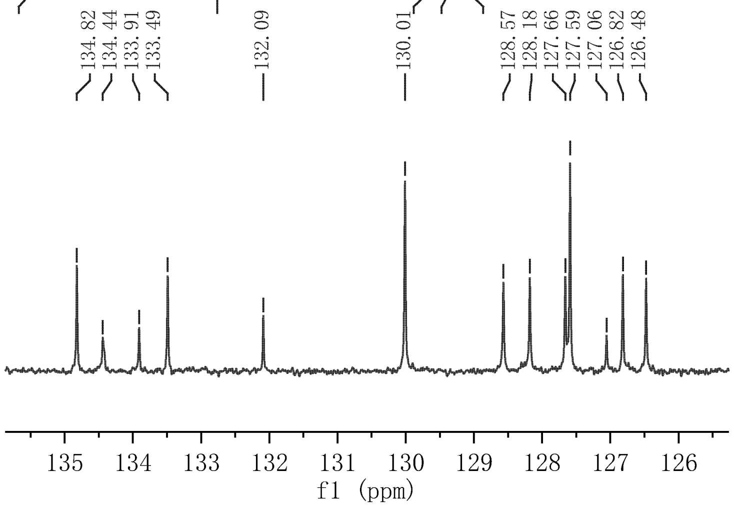

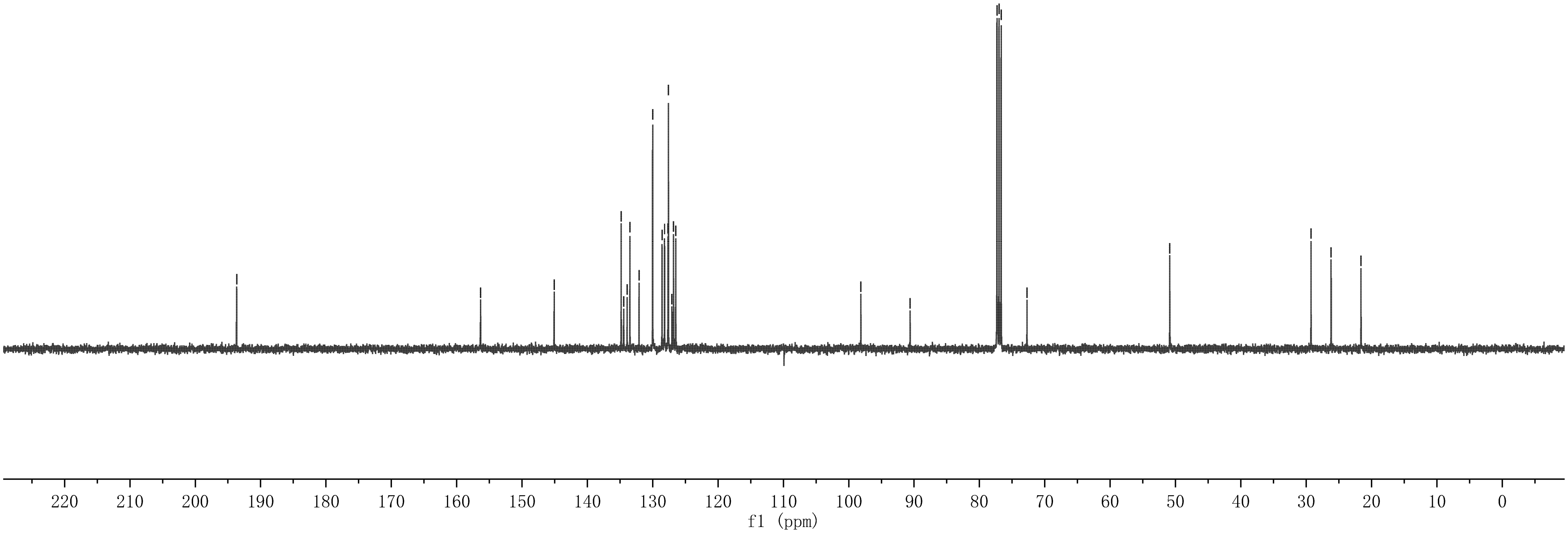

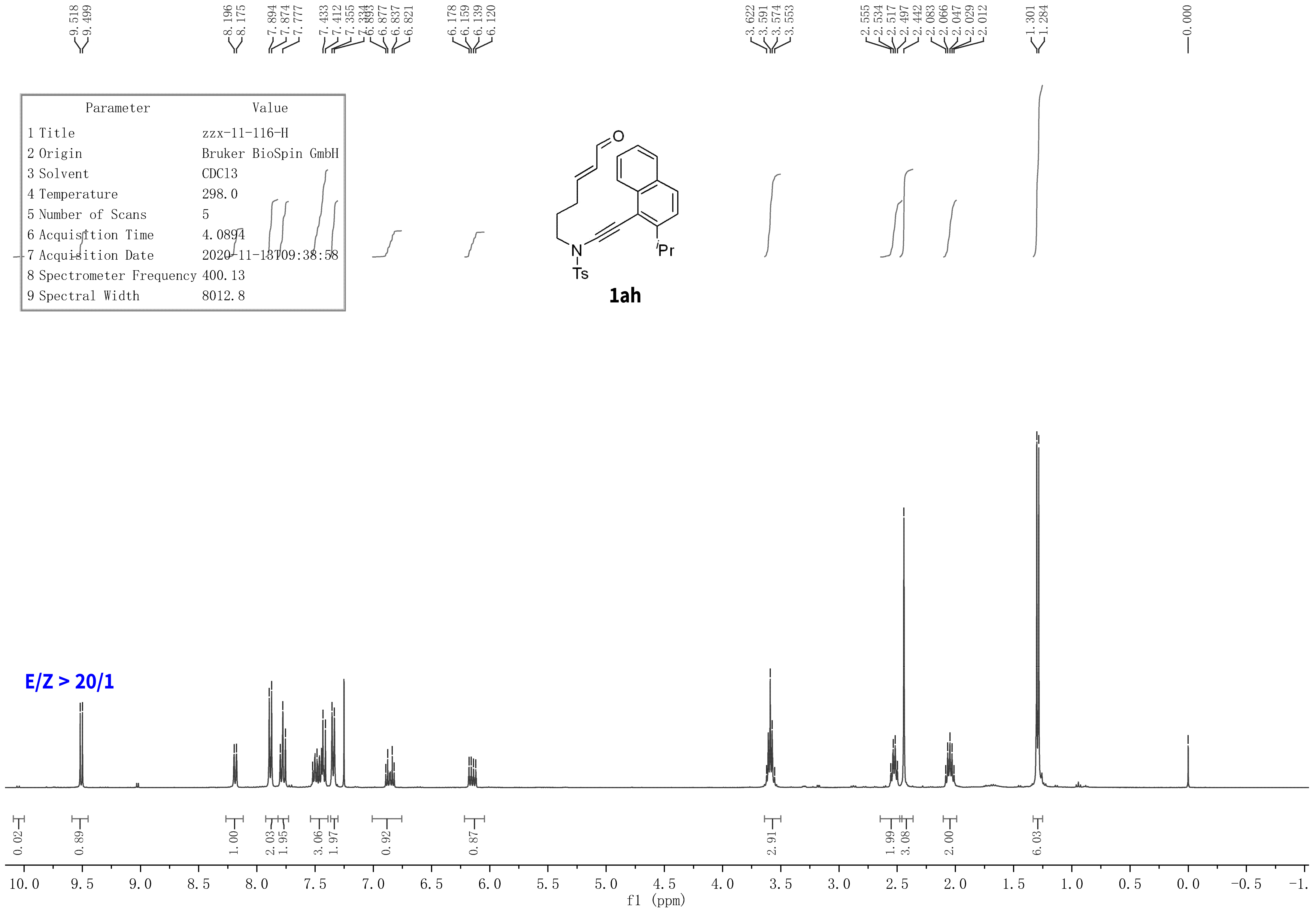

9.518
9.499
8.196
8.175
7.894
7.874
7.777
7.433
7.412
7.355
6.877
6.837
6.821
6.178
6.159
6.139
6.120
3.622
3.591
3.574
3.553
2.555
2.534
2.517
2.497
2.442
2.083
2.066
2.047
2.029
2.012
1.301
1.284
0.000
Parameter Value
1 Title zzx-11-116-H
2 Origin Bruker BioSpin GmbH
3 Solvent CDCl3
4 Temperature 298.0
5 Number of Scans 5
6 Acquisition Time 4.0894
7 Acquisition Date 2020-11-13T09:38:58
8 Spectrometer Frequency 400.13
9 Spectral Width 8012.8
O
N
Ts
iPr
1ah
E/Z > 20/1
0.02
0.89
1.00
2.03
1.95
3.06
1.97
0.92
0.87
2.91
1.99
3.08
2.00
6.03
f1 (ppm)

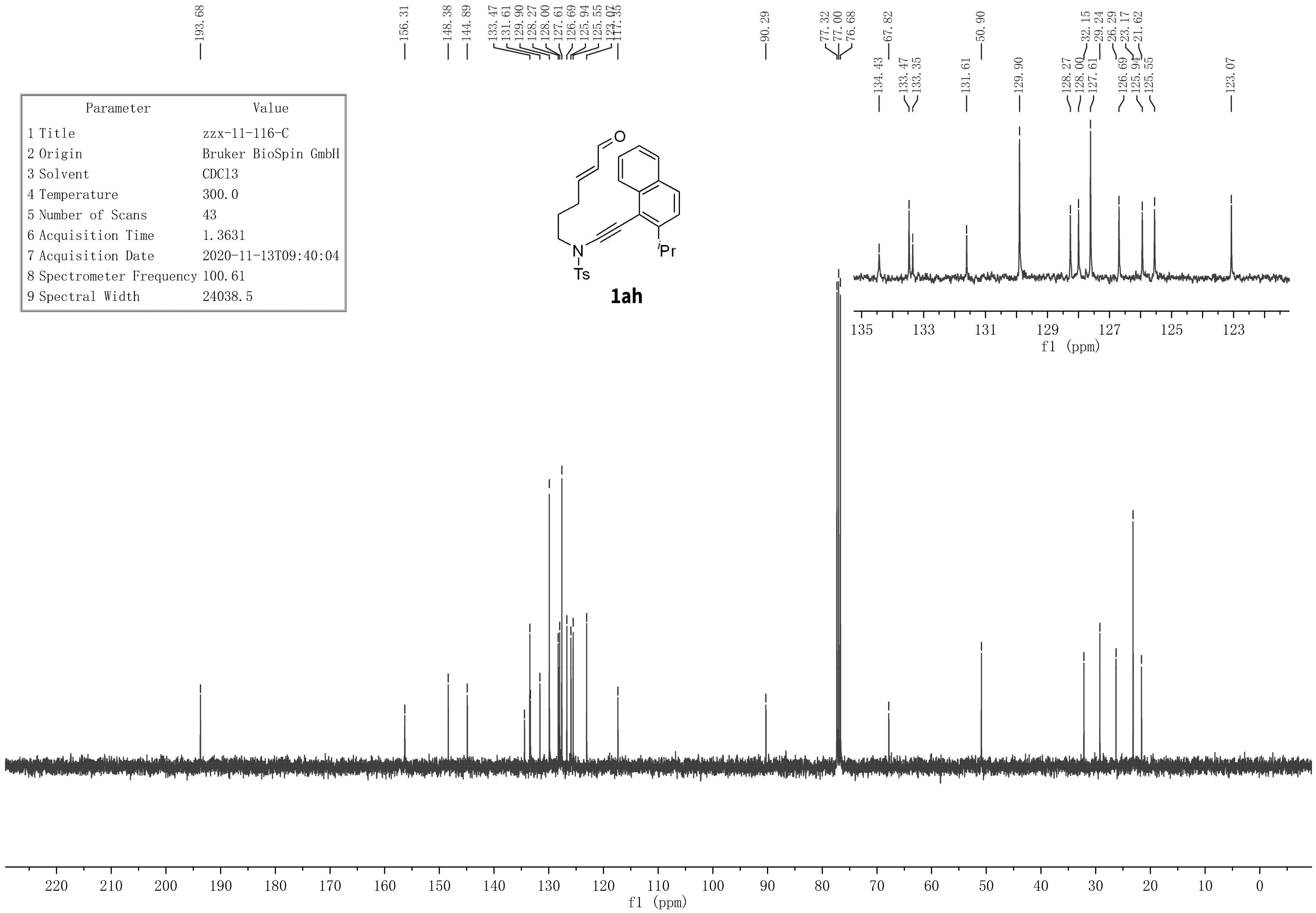

Parameter Value
1 Title zzx-11-116-C
2 Origin Bruker BioSpin GmbH
3 Solvent CDCl3
4 Temperature 300.0
5 Number of Scans 43
6 Acquisition Time 1.3631
7 Acquisition Date 2020-11-13T09:40:04
8 Spectrometer Frequency 100.61
9 Spectral Width 24038.5
O
N
Ts
iPr
1ah
193.68
156.31
148.38
144.89
133.47
131.61
129.90
128.27
128.00
127.61
126.69
125.94
125.55
123.07
117.35
90.29
77.32
77.00
76.68
67.82
50.90
32.15
29.24
26.29
23.17
21.62
134.43
133.47
133.35
131.61
129.90
128.27
128.00
127.61
126.69
125.94
125.55
123.07
135 133 131 129 127 125 123
f1 (ppm)
220 210 200 190 180 170 160 150 140 130 120 110 100 90 80 70 60 50 40 30 20 10 0
f1 (ppm)

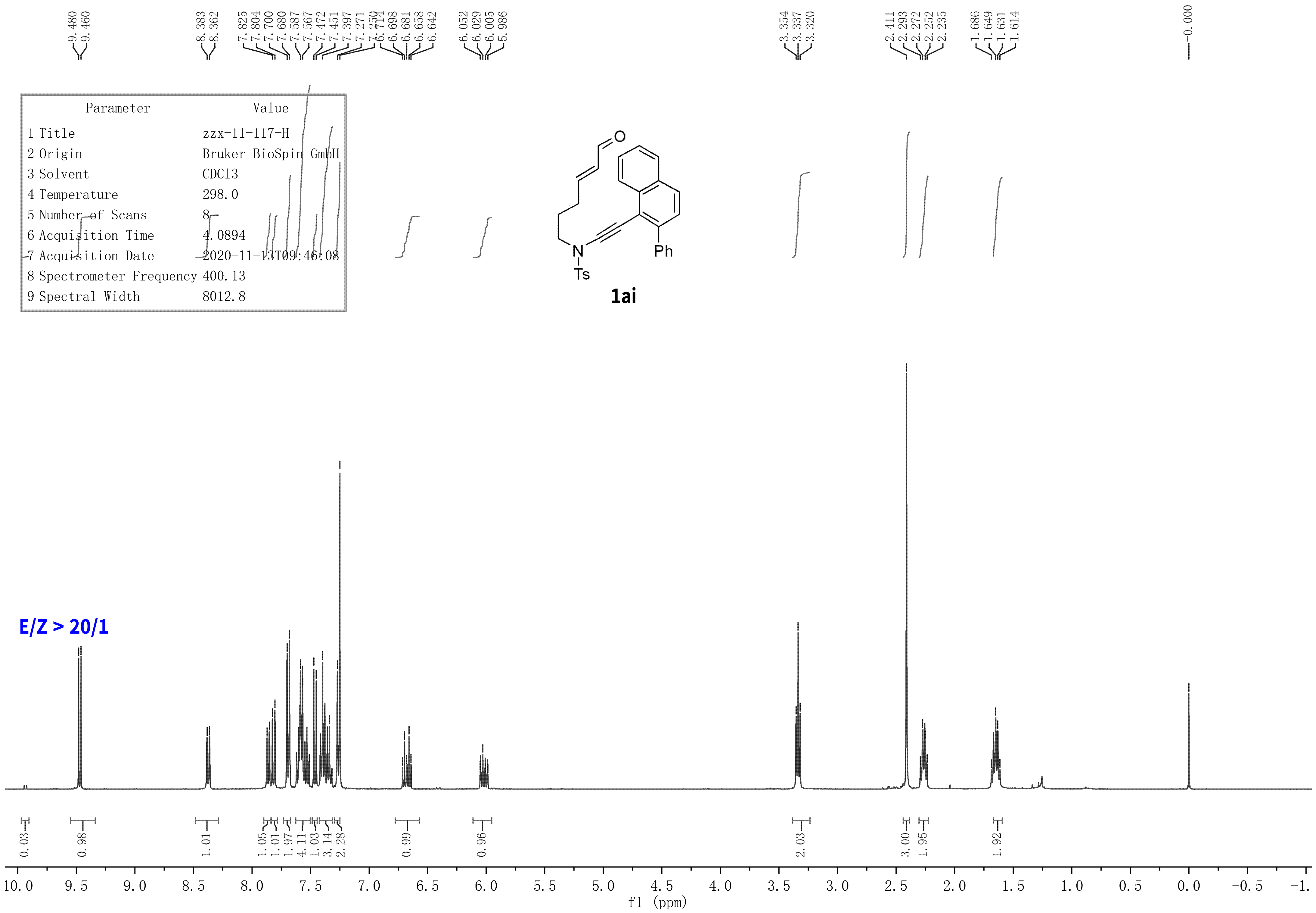

Parameter Value
1 Title zzx-11-117-H
2 Origin Bruker BioSpin GmbH
3 Solvent CDCl3
4 Temperature 298.0
5 Number of Scans 8
6 Acquisition Time 4.0894
7 Acquisition Date 2020-11-13T09:46:08
8 Spectrometer Frequency 400.13
9 Spectral Width 8012.8
9.480 9.460
8.383 8.362
7.825 7.804 7.700 7.680 7.587 7.567 7.472 7.451 7.397 7.271 7.250 6.714 6.698 6.681 6.658 6.642
6.052 6.029 6.005 5.986
3.354 3.337 3.320
2.411 2.293 2.272 2.252 2.235
1.686 1.649 1.631 1.614
0.000
O
N
Ts
Ph
1ai
E/Z > 20/1
0.03 0.98 1.01 1.05 1.01 1.97 4.11 1.03 3.14 2.28 0.99 0.96 2.03 3.00 1.95 1.92
10.0 9.5 9.0 8.5 8.0 7.5 7.0 6.5 6.0 5.5 5.0 4.5 4.0 3.5 3.0 2.5 2.0 1.5 1.0 0.5 0.0 -0.5 -1.
f1 (ppm)

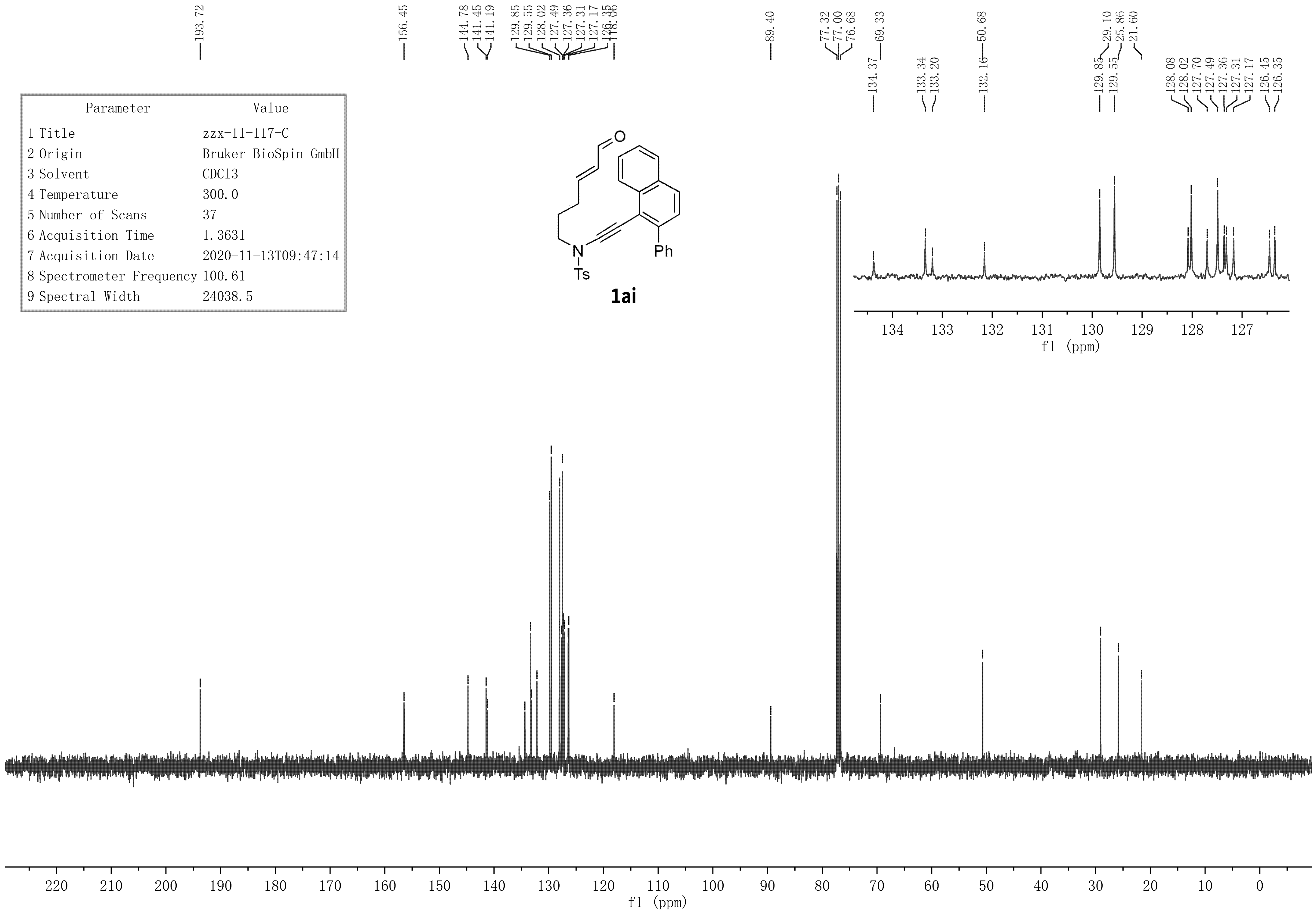

193.72
156.45
144.78
141.45
141.19
129.85
129.55
128.02
127.49
127.36
127.31
127.17
126.35
118.06
89.40
77.32
77.00
76.68
69.33
50.68
29.10
25.86
21.60
134.37
133.34
133.20
132.16
129.85
129.55
128.08
128.02
127.70
127.49
127.36
127.31
127.17
126.45
126.35
Parameter Value
1 Title zzx-11-117-C
2 Origin Bruker BioSpin GmbH
3 Solvent CDCl3
4 Temperature 300.0
5 Number of Scans 37
6 Acquisition Time 1.3631
7 Acquisition Date 2020-11-13T09:47:14
8 Spectrometer Frequency 100.61
9 Spectral Width 24038.5
O
N
Ts
Ph
1ai
134 133 132 131 130 129 128 127
f1 (ppm)
220 210 200 190 180 170 160 150 140 130 120 110 100 90 80 70 60 50 40 30 20 10 0
f1 (ppm)

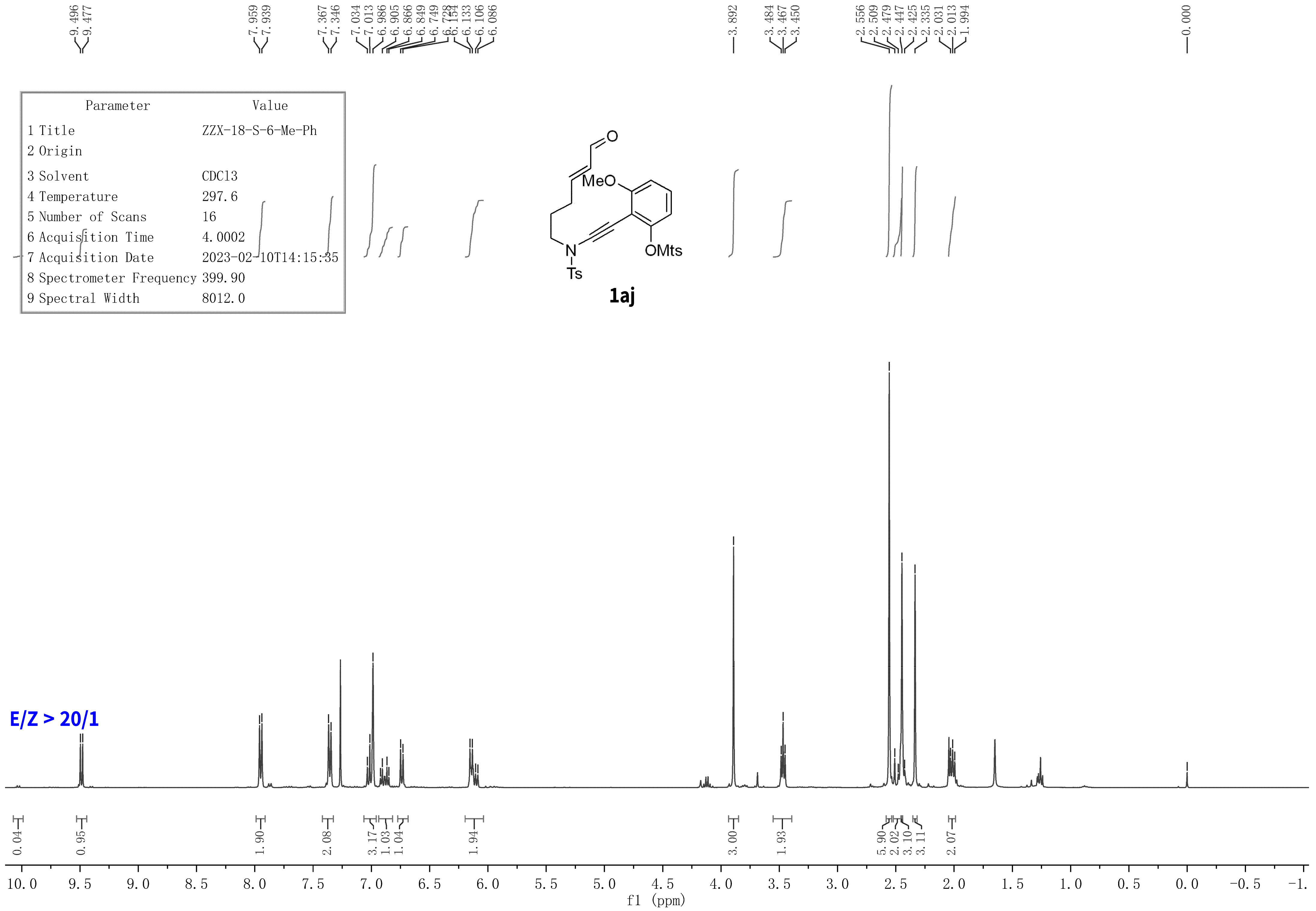

1aj

	Parameter	Value
1	Title	ZZX-18-S-6-Me-Ph
2	Origin	
3	Solvent	CDCl3
4	Temperature	297.6
5	Number of Scans	16
6	Acquisition Time	4.0002
7	Acquisition Date	2023-02-10T14:15:35
8	Spectrometer Frequency	399.90
9	Spectral Width	8012.0

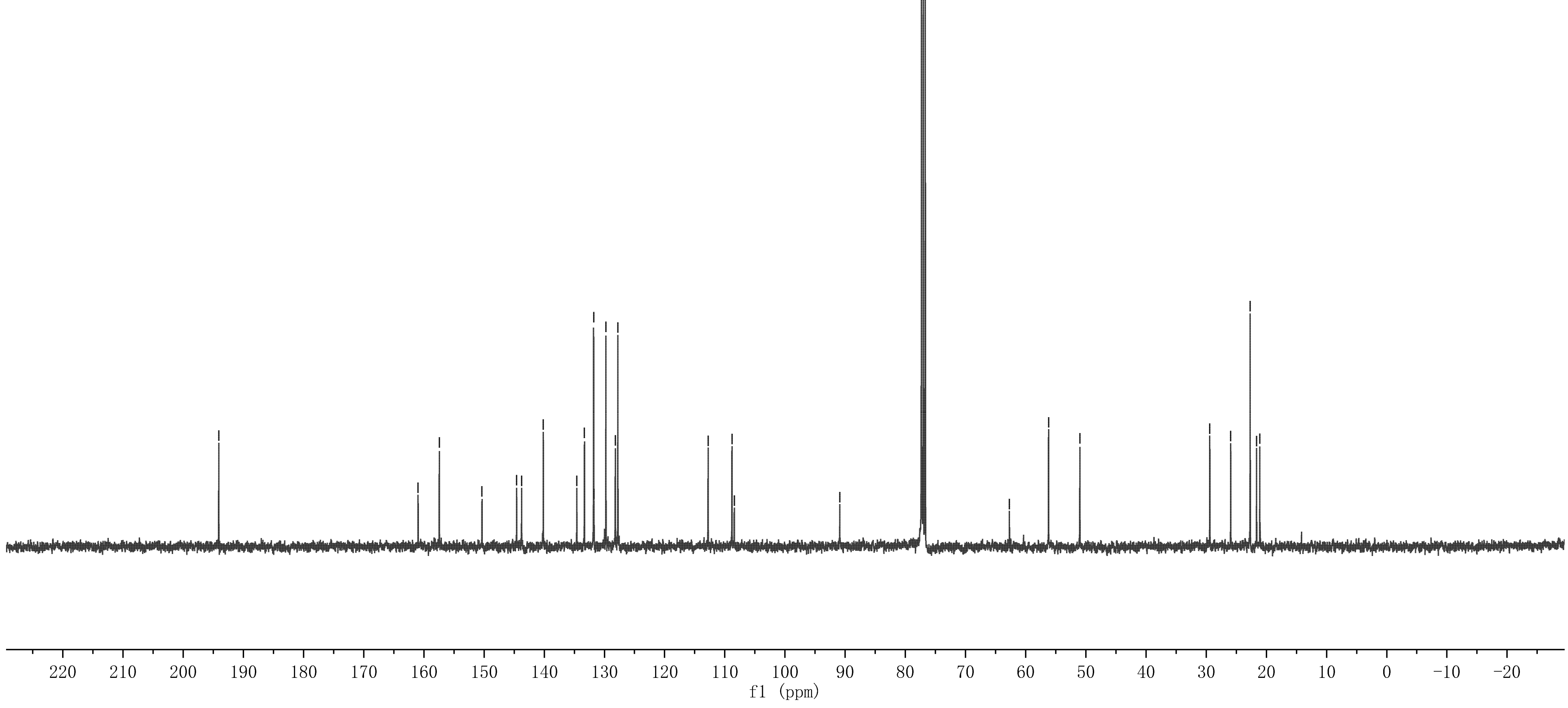

Parameter	Value
1 Title	ZZX-18-S-6-Me-Ph
2 Origin	
3 Solvent	CDCl3
4 Temperature	297.6
5 Number of Scans	500
6 Acquisition Time	1.0000
7 Acquisition Date	2023-02-10T14:34:51
8 Spectrometer Frequency	100.56
9 Spectral Width	26041.0

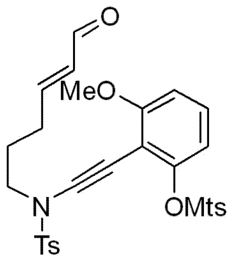

1aj

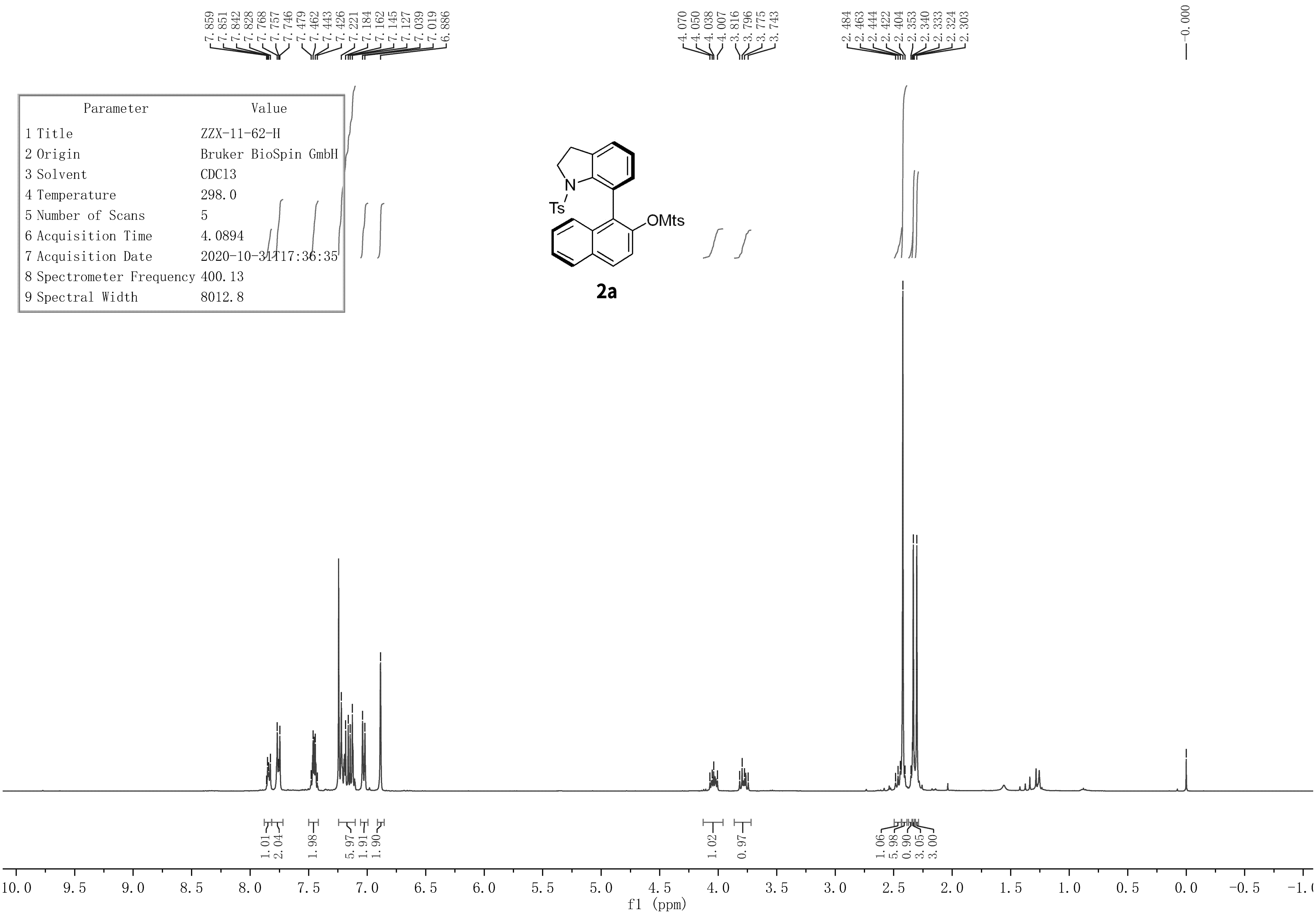
7.859
7.851
7.842
7.828
7.768
7.757
7.746
7.479
7.462
7.443
7.426
7.221
7.184
7.162
7.145
7.127
7.039
7.019
6.886
4.070
4.050
4.038
4.007
3.816
3.796
3.775
3.743
2.484
2.463
2.444
2.422
2.404
2.353
2.340
2.333
2.324
2.303
-0.000
Parameter
Value
1 Title
ZZX-11-62-H
2 Origin
Bruker BioSpin GmbH
3 Solvent
CDCl3
4 Temperature
298.0
5 Number of Scans
5
6 Acquisition Time
4.0894
7 Acquisition Date
2020-10-31T17:36:35
8 Spectrometer Frequency
400.13
9 Spectral Width
8012.8
N
Ts
OMts
2a
1.01
2.04
1.98
5.97
1.91
1.90
1.02
0.97
1.06
5.98
0.90
3.05
3.00
10.0
9.5
9.0
8.5
8.0
7.5
7.0
6.5
6.0
5.5
5.0
4.5
4.0
3.5
3.0
2.5
2.0
1.5
1.0
0.5
0.0
-0.5
f1 (ppm)

Parameter	Value
1 Title	ZZX-11-62-C-1
2 Origin	Bruker BioSpin GmbH
3 Solvent	CDCl3
4 Temperature	300.0
5 Number of Scans	24
6 Acquisition Time	1.3631
7 Acquisition Date	2020-10-31T17:39:29
8 Spectrometer Frequency	100.61
9 Spectral Width	24038.5

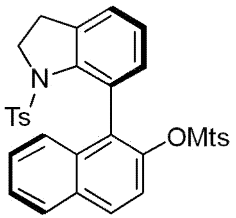

2a

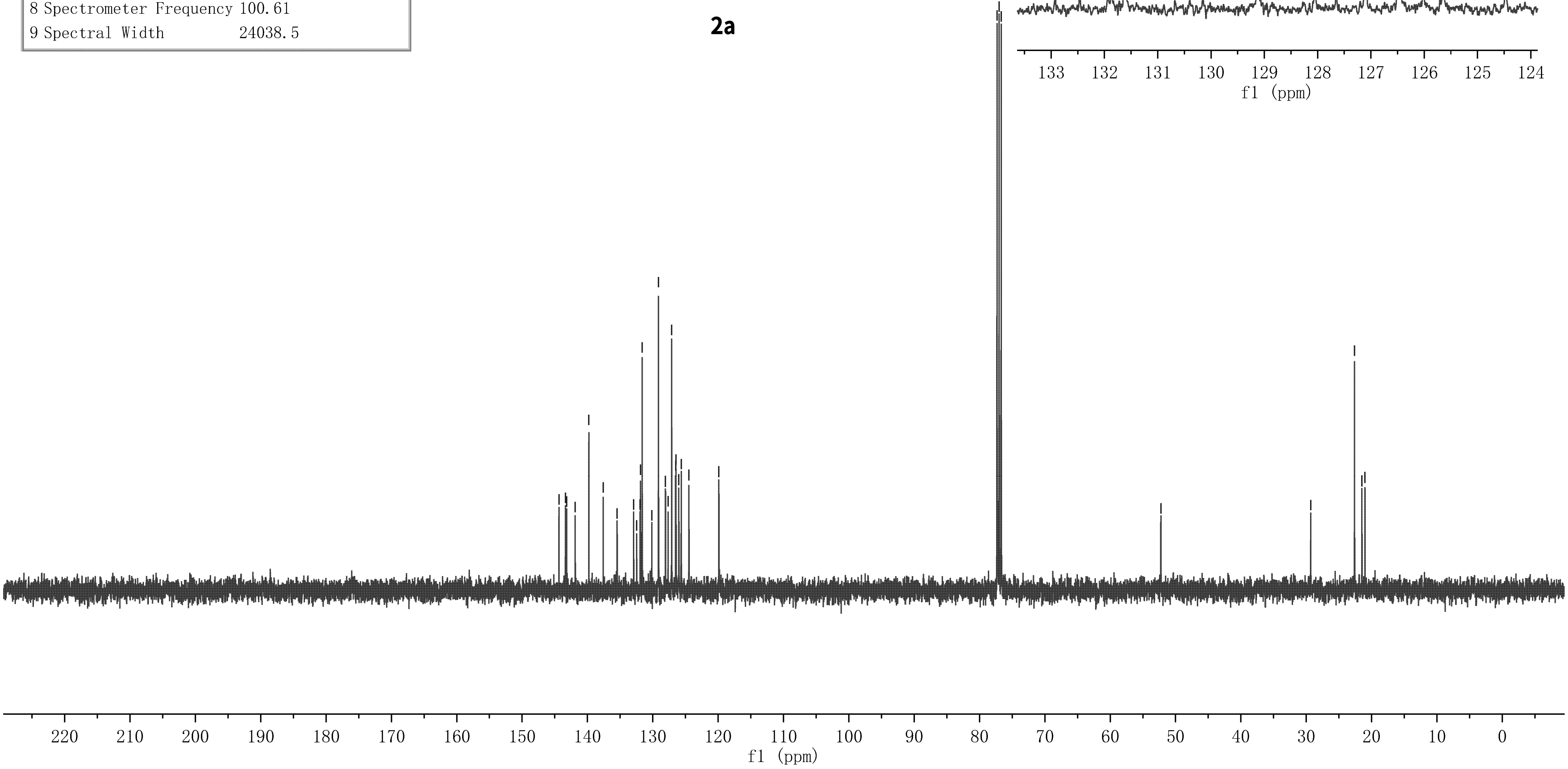

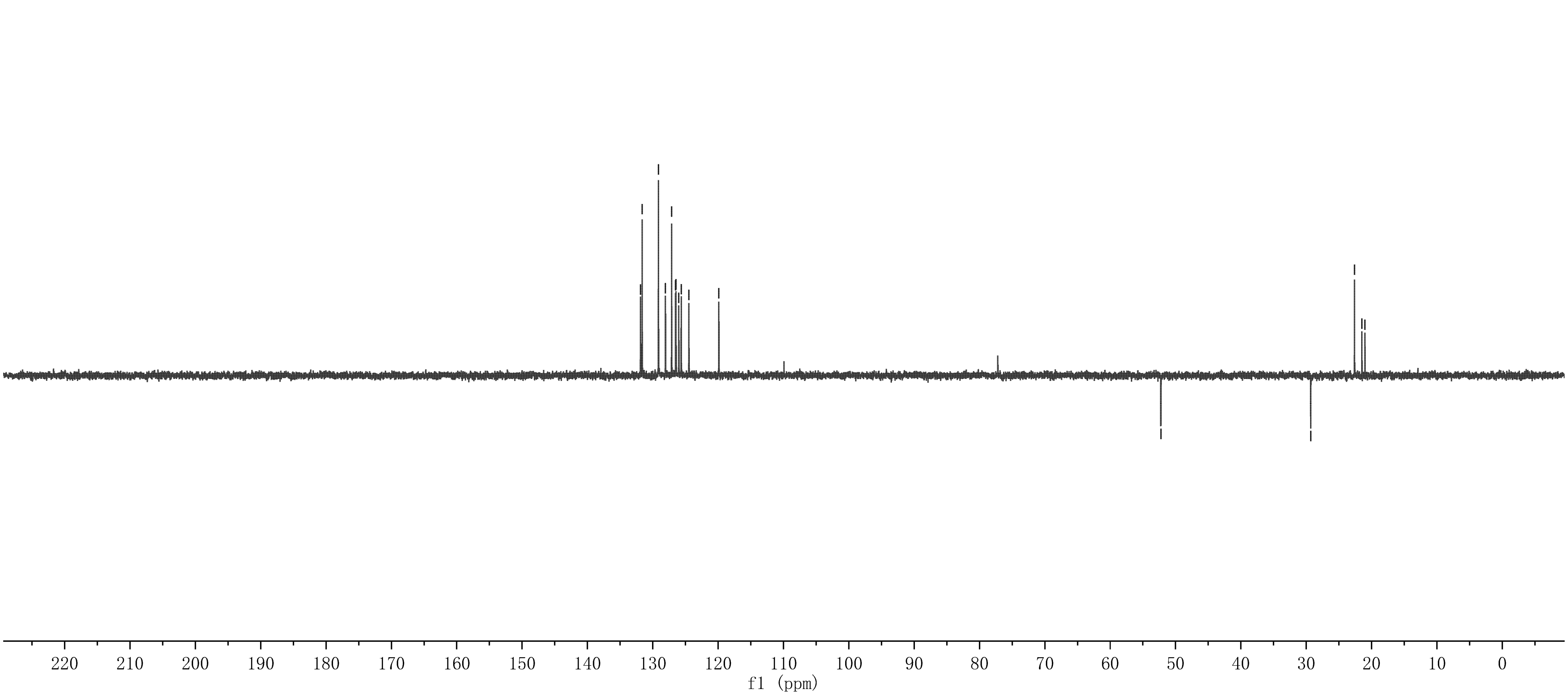

Parameter	Value
1 Title	ZZX-11-62-DEPT
2 Origin	Bruker BioSpin GmbH
3 Solvent	CDCl3
4 Temperature	300.0
5 Number of Scans	21
6 Acquisition Time	1.3631
7 Acquisition Date	2020-10-31T17:42:02
8 Spectrometer Frequency	100.61
9 Spectral Width	24038.5

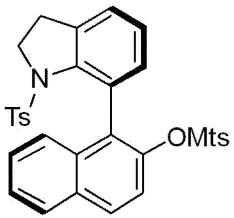

2a

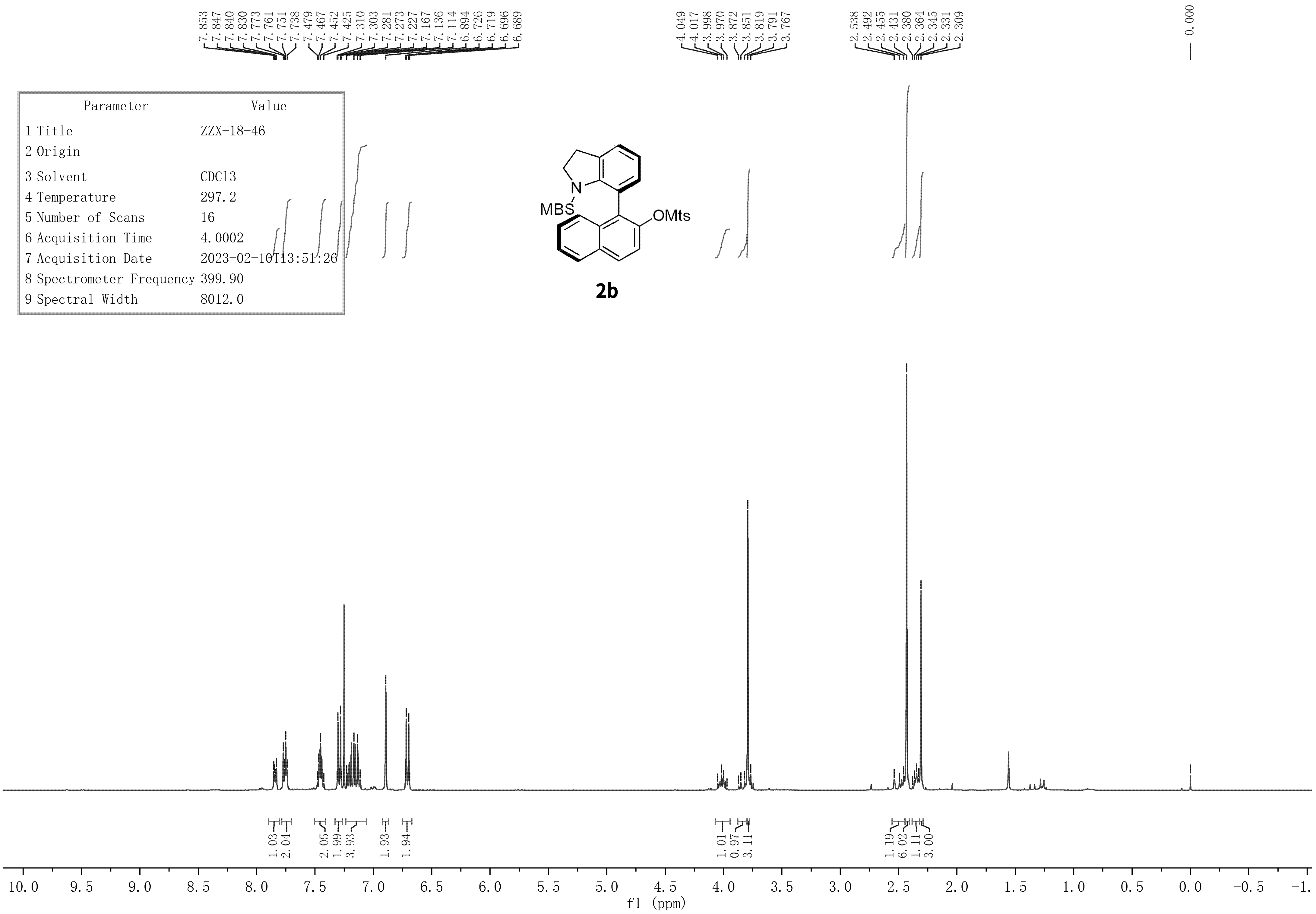

Parameter	Value
1 Title	ZZX-18-46
2 Origin	
3 Solvent	CDCl3
4 Temperature	297.2
5 Number of Scans	16
6 Acquisition Time	4.0002
7 Acquisition Date	2023-02-10T13:51:26
8 Spectrometer Frequency	399.90
9 Spectral Width	8012.0

2b

Parameter	Value
1 Title	ZZX-18-46
2 Origin	
3 Solvent	CDCl3
4 Temperature	297.0
5 Number of Scans	500
6 Acquisition Time	1.0000
7 Acquisition Date	2023-02-10T14:10:36
8 Spectrometer Frequency	100.56
9 Spectral Width	26041.0

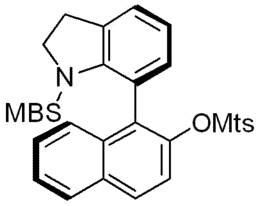

2b

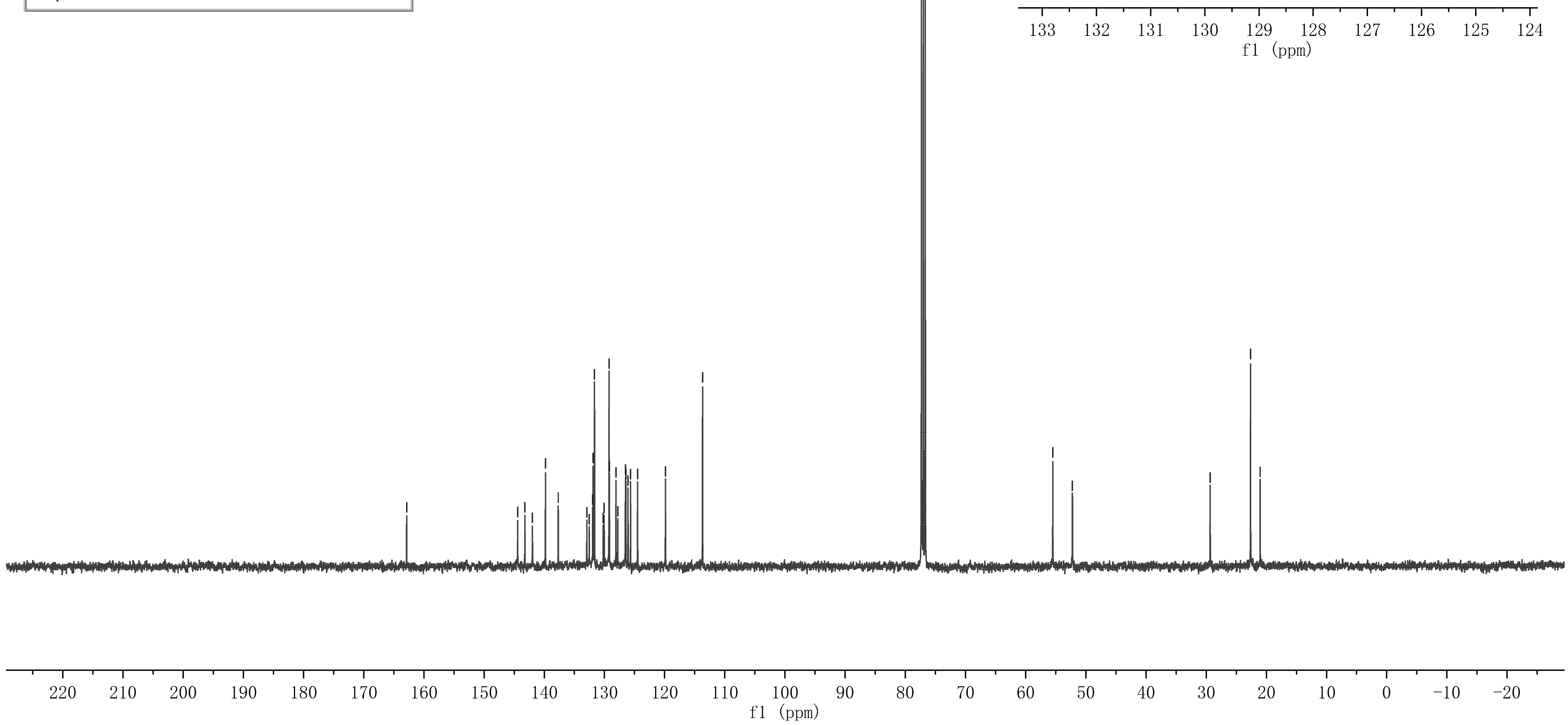

Parameter	Value
1 Title	ZZX-11-140
2 Origin	
3 Solvent	CDCl3
4 Temperature	297.1
5 Number of Scans	16
6 Acquisition Time	4.0002
7 Acquisition Date	2022-03-18T23:11:00
8 Spectrometer Frequency	399.93
9 Spectral Width	8012.0

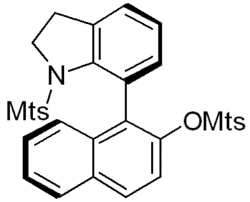

2c

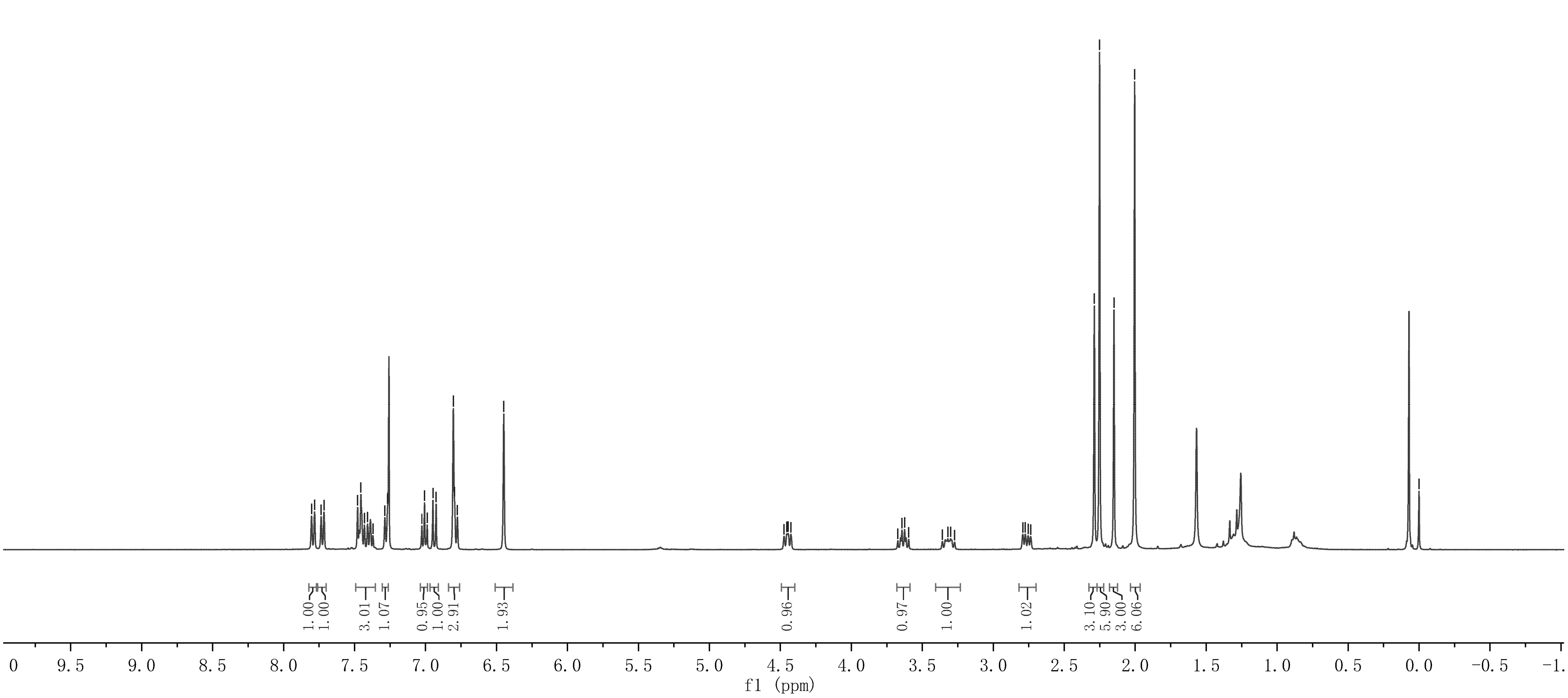

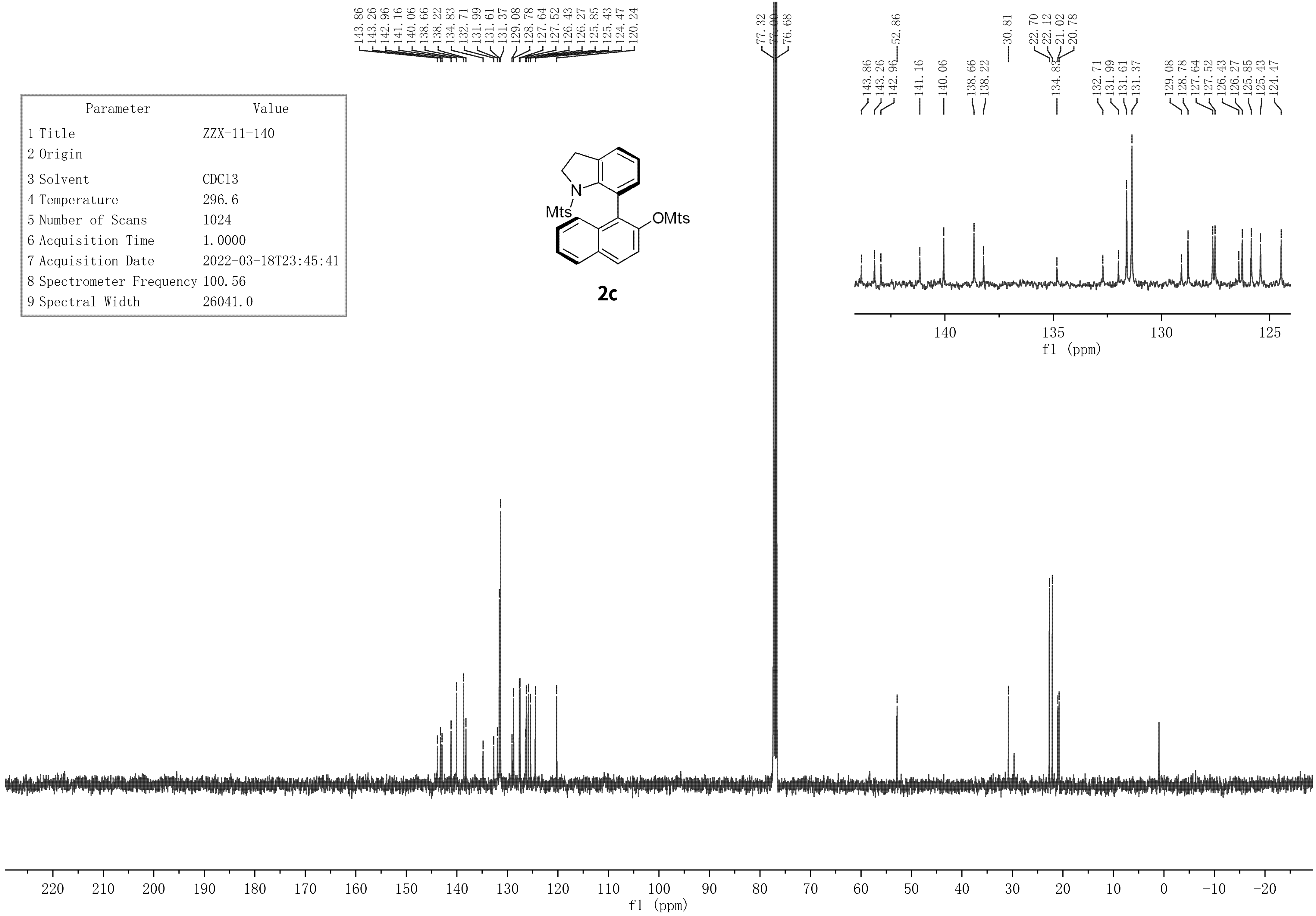
Parameter Value
1 Title ZZX-11-140
2 Origin
3 Solvent CDCl3
4 Temperature 296.6
5 Number of Scans 1024
6 Acquisition Time 1.0000
7 Acquisition Date 2022-03-18T23:45:41
8 Spectrometer Frequency 100.56
9 Spectral Width 26041.0
N
Mts
OMts
2c
143.86 143.26 142.96 141.16 140.06 138.66 138.22 134.83 132.71 131.99 131.61 131.37 129.08 128.78 127.64 127.52 126.43 126.27 125.85 125.43 124.47 120.24
77.32 77.00 76.68
52.86
30.81
22.70 22.12 21.02 20.78
f1 (ppm)

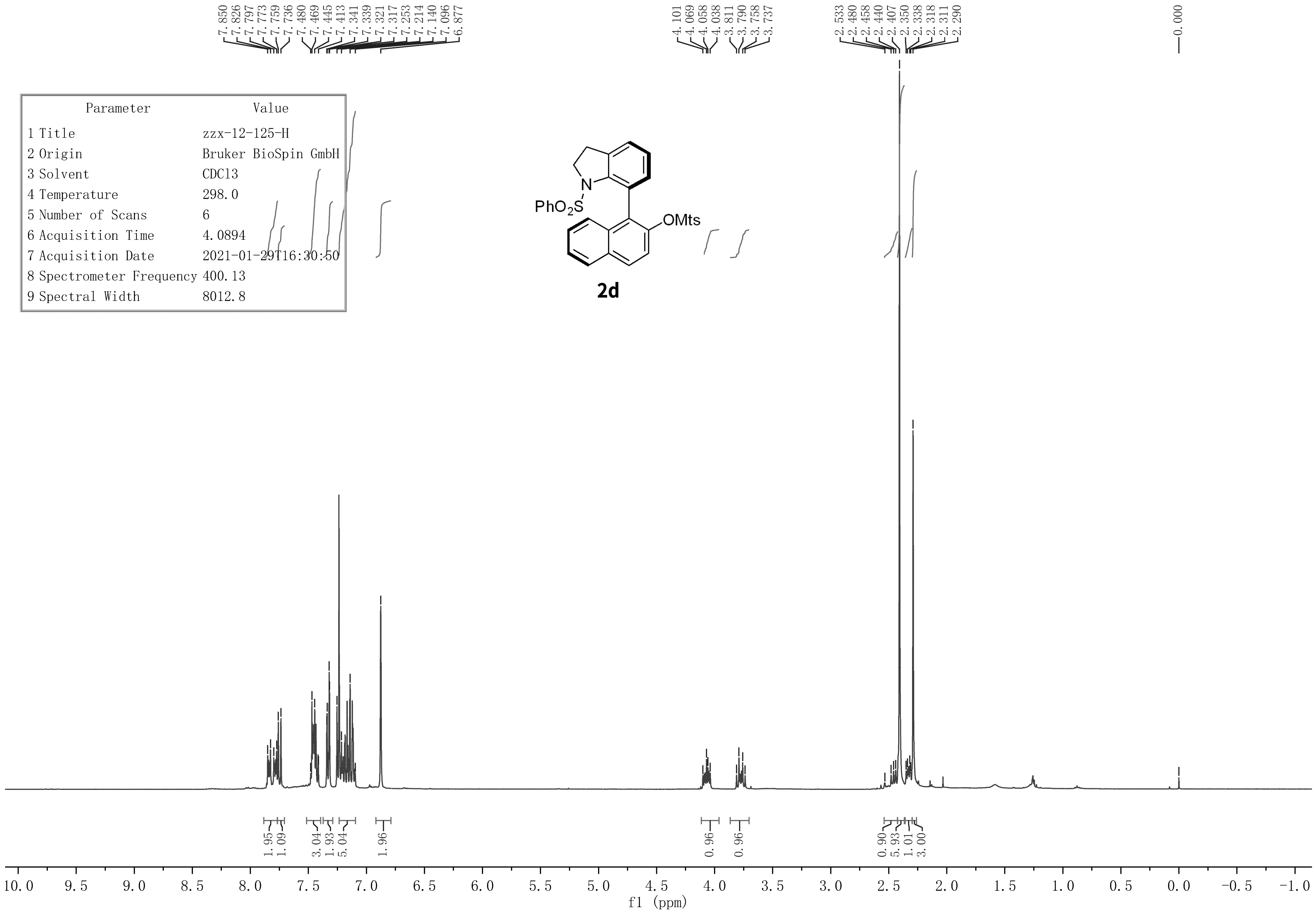
7.850 7.826 7.797 7.773 7.759 7.736 7.480 7.469 7.445 7.413 7.341 7.339 7.321 7.317 7.253 7.214 7.140 7.096 6.877
4.101 4.069 4.058 4.038 3.811 3.790 3.758 3.737
2.533 2.480 2.458 2.440 2.407 2.350 2.338 2.318 2.311 2.290
0.000
Parameter | Value
1 Title | zzx-12-125-H
2 Origin | Bruker BioSpin GmbH
3 Solvent | CDCl3
4 Temperature | 298.0
5 Number of Scans | 6
6 Acquisition Time | 4.0894
7 Acquisition Date | 2021-01-29T16:30:50
8 Spectrometer Frequency | 400.13
9 Spectral Width | 8012.8
N
PhO2S
OMts
2d
1.95 1.09 3.04 1.93 5.04 1.96 0.96 0.96 0.90 5.93 1.01 3.00
10.0 9.5 9.0 8.5 8.0 7.5 7.0 6.5 6.0 5.5 5.0 4.5 4.0 3.5 3.0 2.5 2.0 1.5 1.0 0.5 0.0 −0.5 −1.0
f1 (ppm)

Parameter	Value
1 Title	zzx-12-125-C
2 Origin	Bruker BioSpin GmbH
3 Solvent	CDCl3
4 Temperature	300.0
5 Number of Scans	27
6 Acquisition Time	1.3631
7 Acquisition Date	2021-01-29T16:32:00
8 Spectrometer Frequency	100.62
9 Spectral Width	24038.5

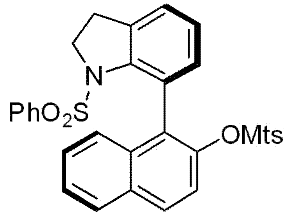

2d

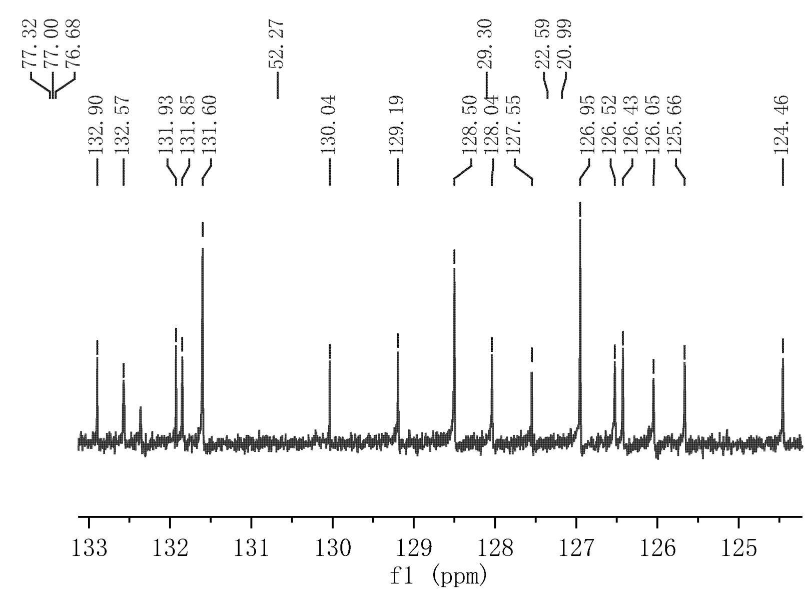

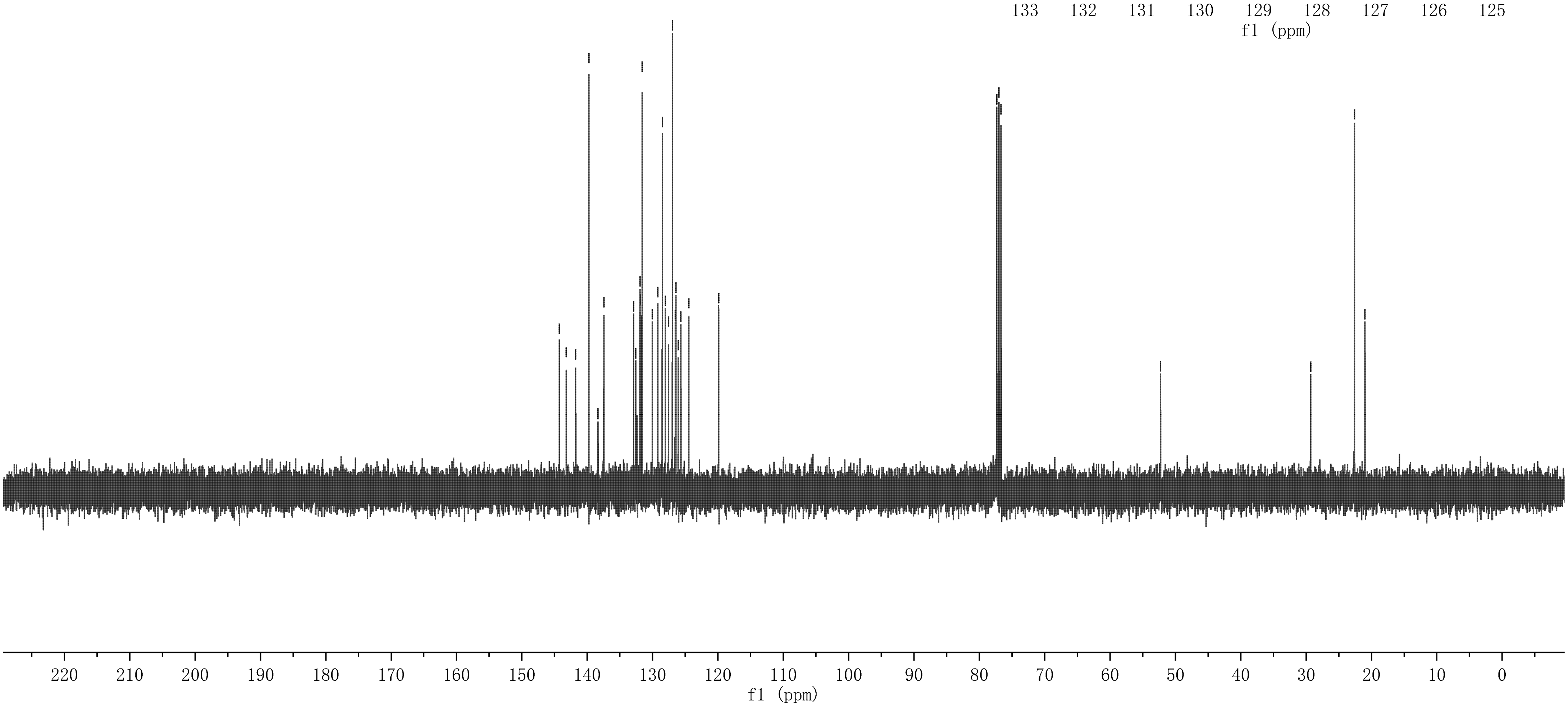

Parameter	Value
1 Title	ZZX-11-139-H
2 Origin	Bruker BioSpin GmbH
3 Solvent	CDCl3
4 Temperature	298.0
5 Number of Scans	6
6 Acquisition Time	4.0894
7 Acquisition Date	2020-11-24T09:14:01
8 Spectrometer Frequency	400.13
9 Spectral Width	8012.8

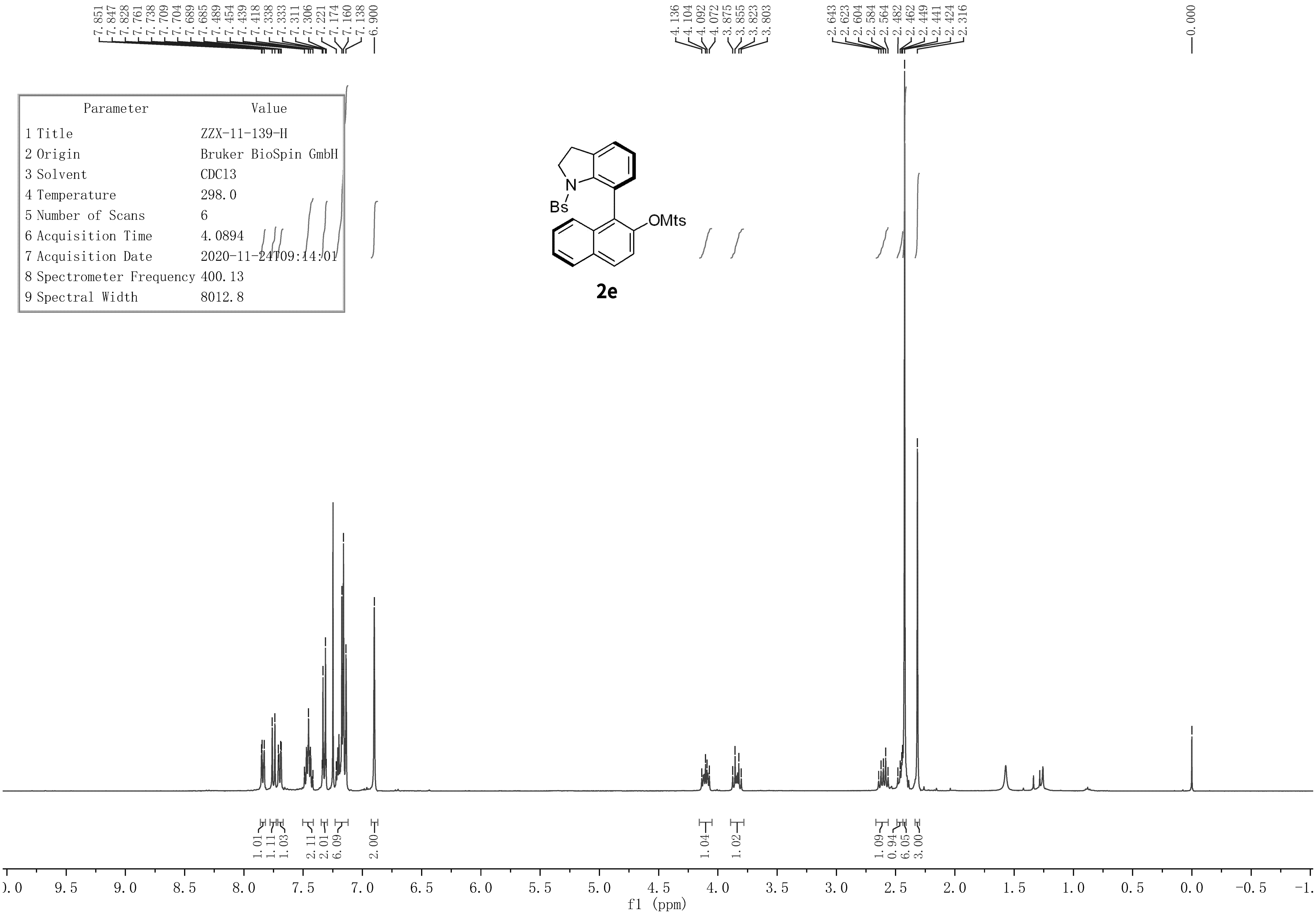

2e

Parameter	Value
1 Title	ZZX-11-139-C
2 Origin	Bruker BioSpin GmbH
3 Solvent	CDCl3
4 Temperature	300.0
5 Number of Scans	65
6 Acquisition Time	1.3631
7 Acquisition Date	2020-11-24T09:15:58
8 Spectrometer Frequency	100.61
9 Spectral Width	24038.5

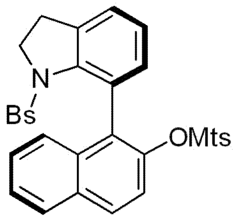

2e

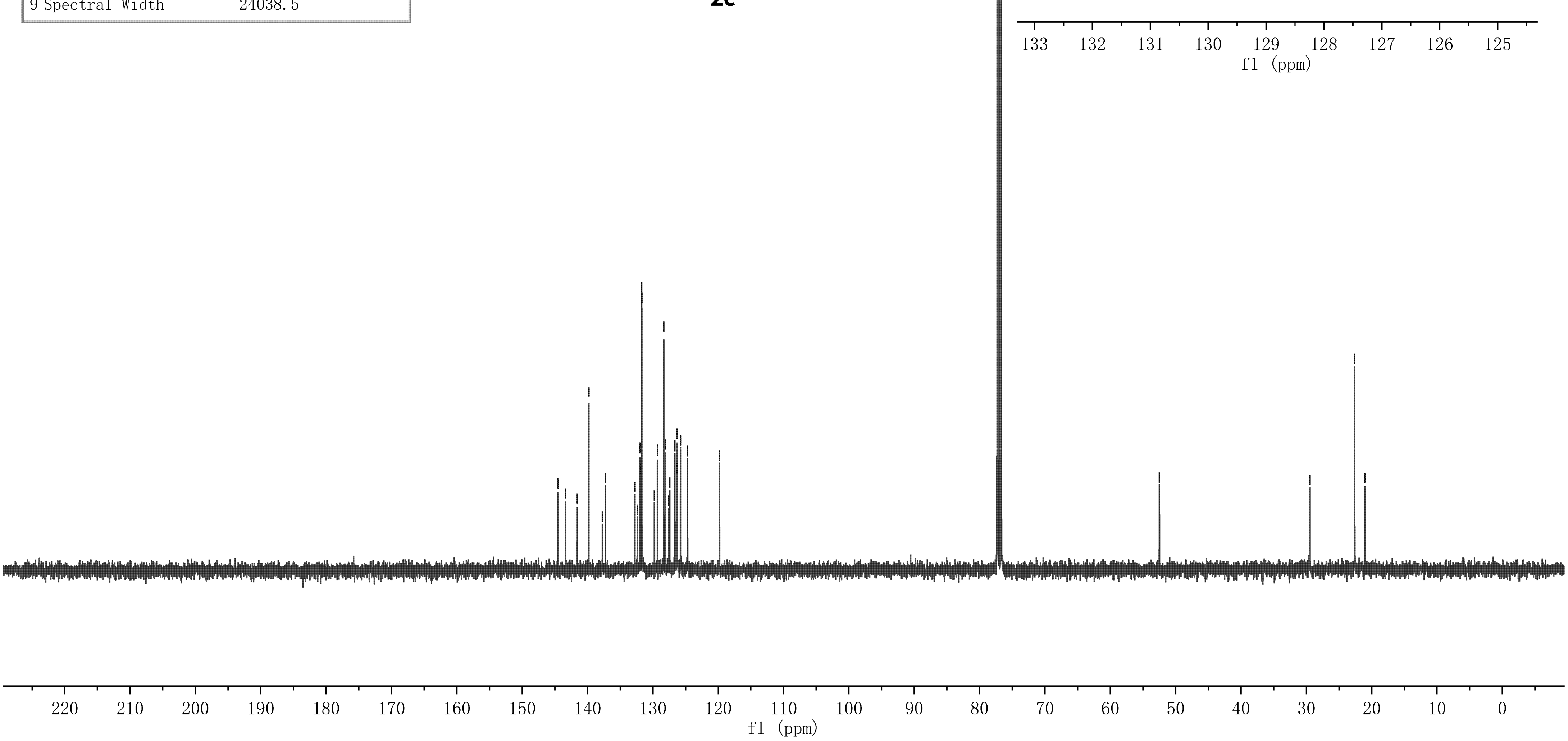

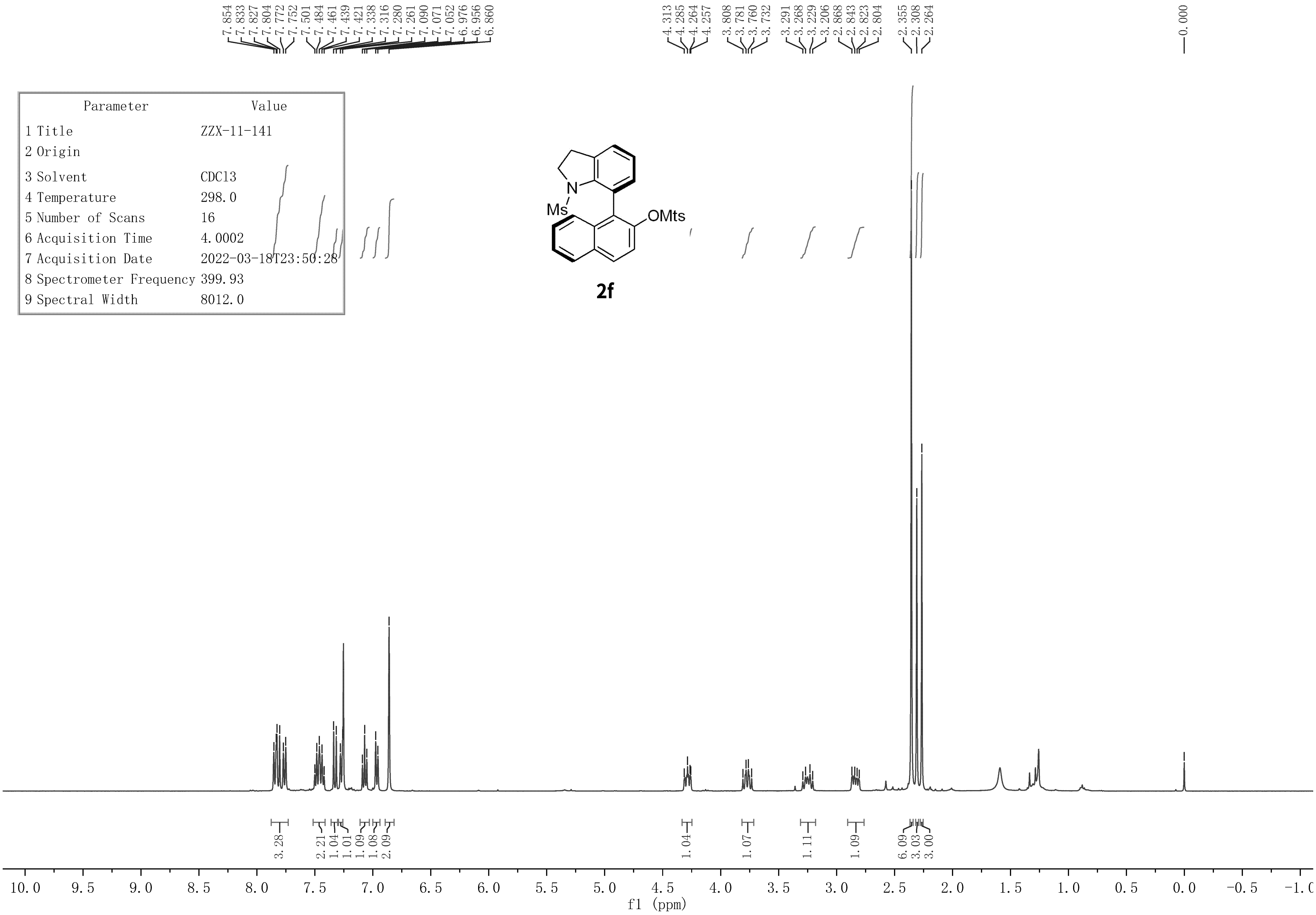
7.854 7.833 7.827 7.804 7.772 7.752 7.501 7.484 7.461 7.439 7.421 7.338 7.316 7.280 7.261 7.090 7.071 7.052 6.976 6.956 6.860
4.313 4.285 4.264 4.257 3.808 3.781 3.760 3.732 3.291 3.268 3.229 3.206 2.868 2.843 2.823 2.804 2.355 2.308 2.264
0.000
Parameter Value
1 Title ZZX-11-141
2 Origin
3 Solvent CDCl3
4 Temperature 298.0
5 Number of Scans 16
6 Acquisition Time 4.0002
7 Acquisition Date 2022-03-18T23:50:28
8 Spectrometer Frequency 399.93
9 Spectral Width 8012.0
N
Ms
OMts
2f
3.28 2.21 1.04 1.01 1.09 1.08 2.09 1.04 1.07 1.11 1.09 6.09 3.03 3.00
10.0 9.5 9.0 8.5 8.0 7.5 7.0 6.5 6.0 5.5 5.0 4.5 4.0 3.5 3.0 2.5 2.0 1.5 1.0 0.5 0.0 -0.5 -1.0
f1 (ppm)

Parameter	Value
1 Title	ZZX-11-141
2 Origin	
3 Solvent	CDCl3
4 Temperature	298.1
5 Number of Scans	500
6 Acquisition Time	1.0000
7 Acquisition Date	2022-03-19T00:07:42
8 Spectrometer Frequency	100.56
9 Spectral Width	26041.0

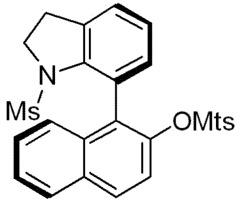

2f

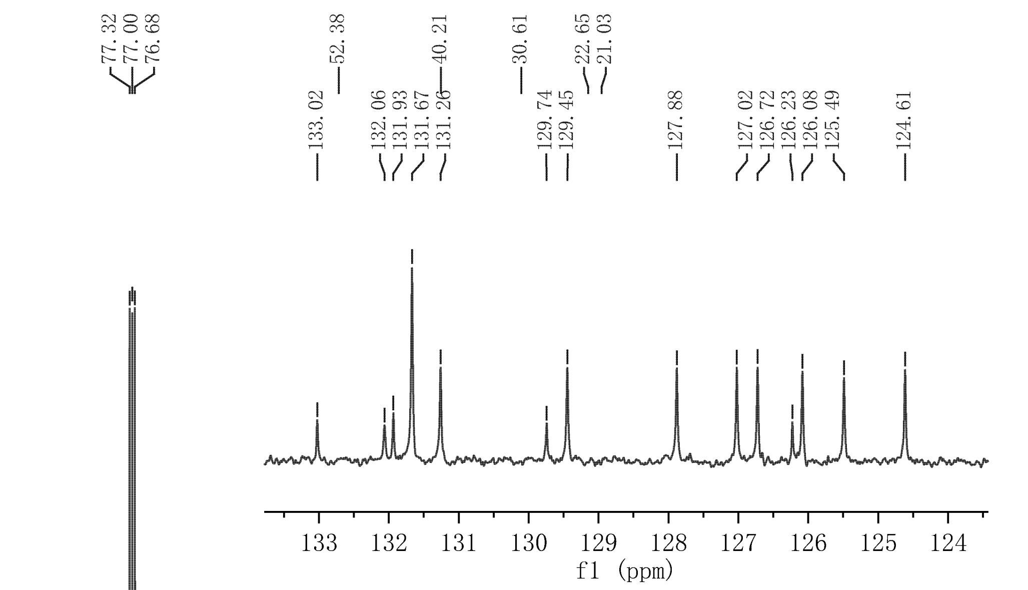

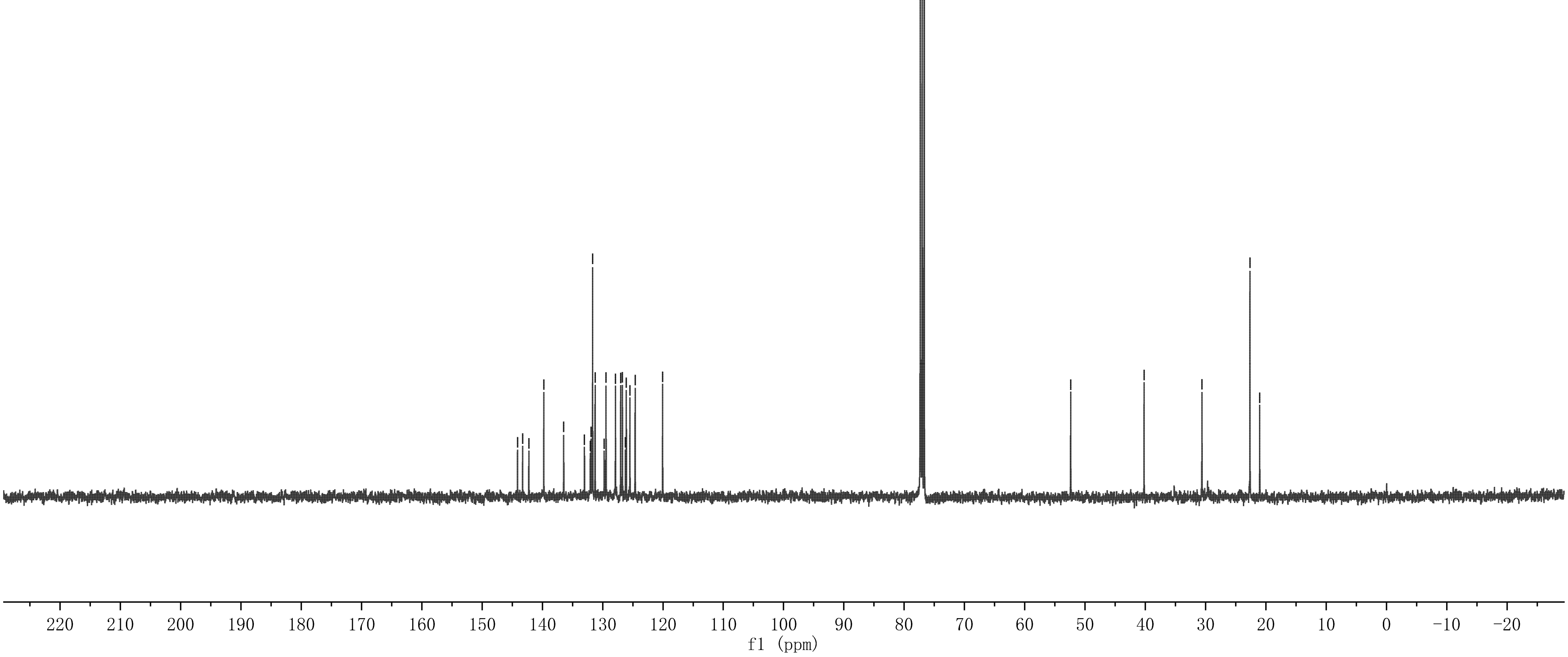

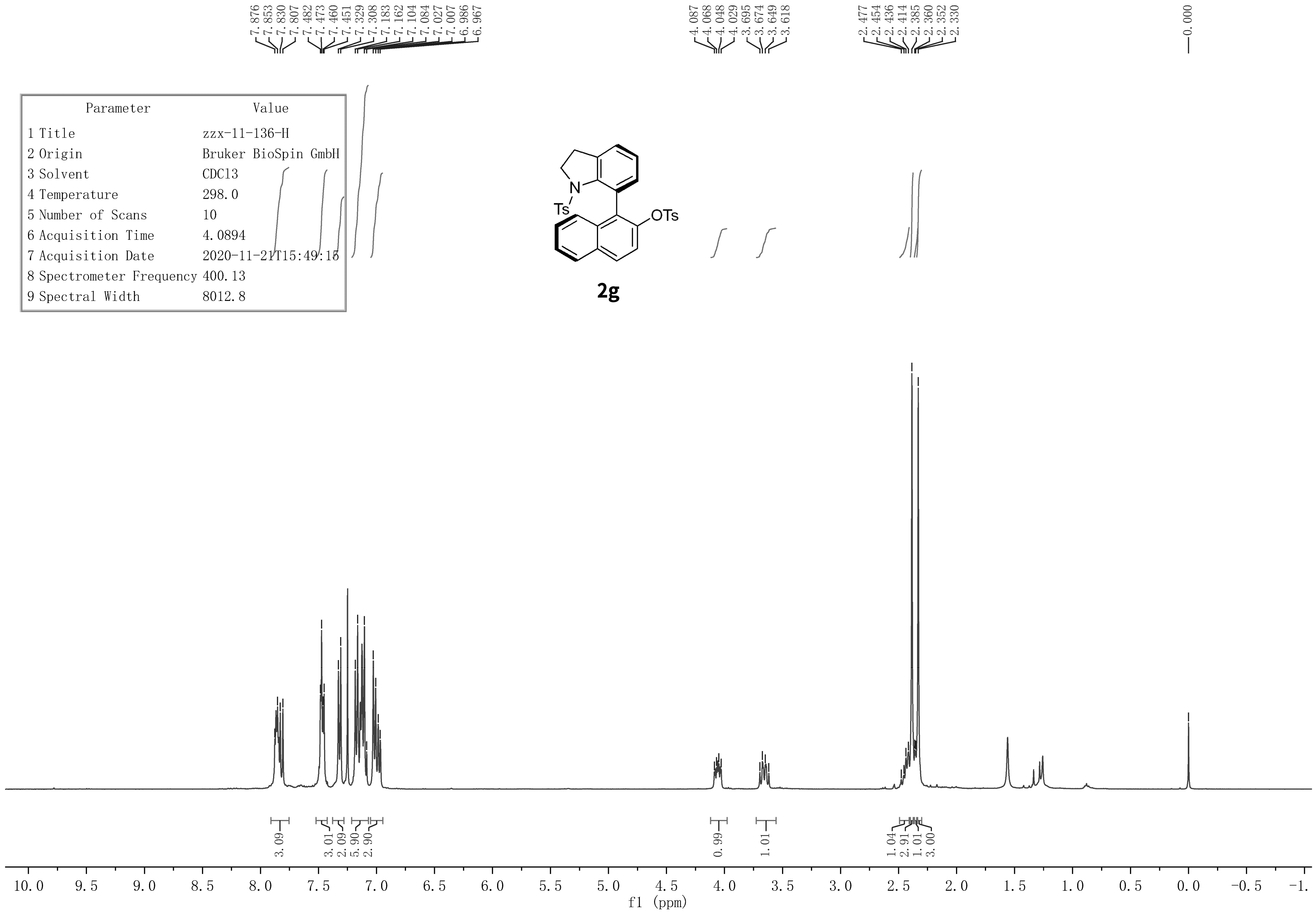

7.876
7.853
7.830
7.807
7.482
7.473
7.460
7.451
7.329
7.308
7.183
7.162
7.104
7.084
7.027
7.007
6.986
6.967
4.087
4.068
4.048
4.029
3.695
3.674
3.649
3.618
2.477
2.454
2.436
2.414
2.385
2.360
2.352
2.330
0.000
Parameter Value
1 Title zzx-11-136-H
2 Origin Bruker BioSpin GmbH
3 Solvent CDCl3
4 Temperature 298.0
5 Number of Scans 10
6 Acquisition Time 4.0894
7 Acquisition Date 2020-11-21T15:49:15
8 Spectrometer Frequency 400.13
9 Spectral Width 8012.8
N
Ts
OTs
2g
3.09
3.01
2.09
5.90
2.90
0.99
1.01
1.04
2.91
1.01
3.00
10.0
9.5
9.0
8.5
8.0
7.5
7.0
6.5
6.0
5.5
5.0
4.5
4.0
3.5
3.0
2.5
2.0
1.5
1.0
0.5
0.0
-0.5
-1.
f1 (ppm)

Parameter	Value
1 Title	zzx-11-136-C
2 Origin	Bruker BioSpin GmbH
3 Solvent	CDCl3
4 Temperature	300.0
5 Number of Scans	90
6 Acquisition Time	1.3631
7 Acquisition Date	2020-11-21T15:50:43
8 Spectrometer Frequency	100.61
9 Spectral Width	24038.5

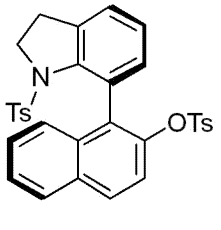

2g

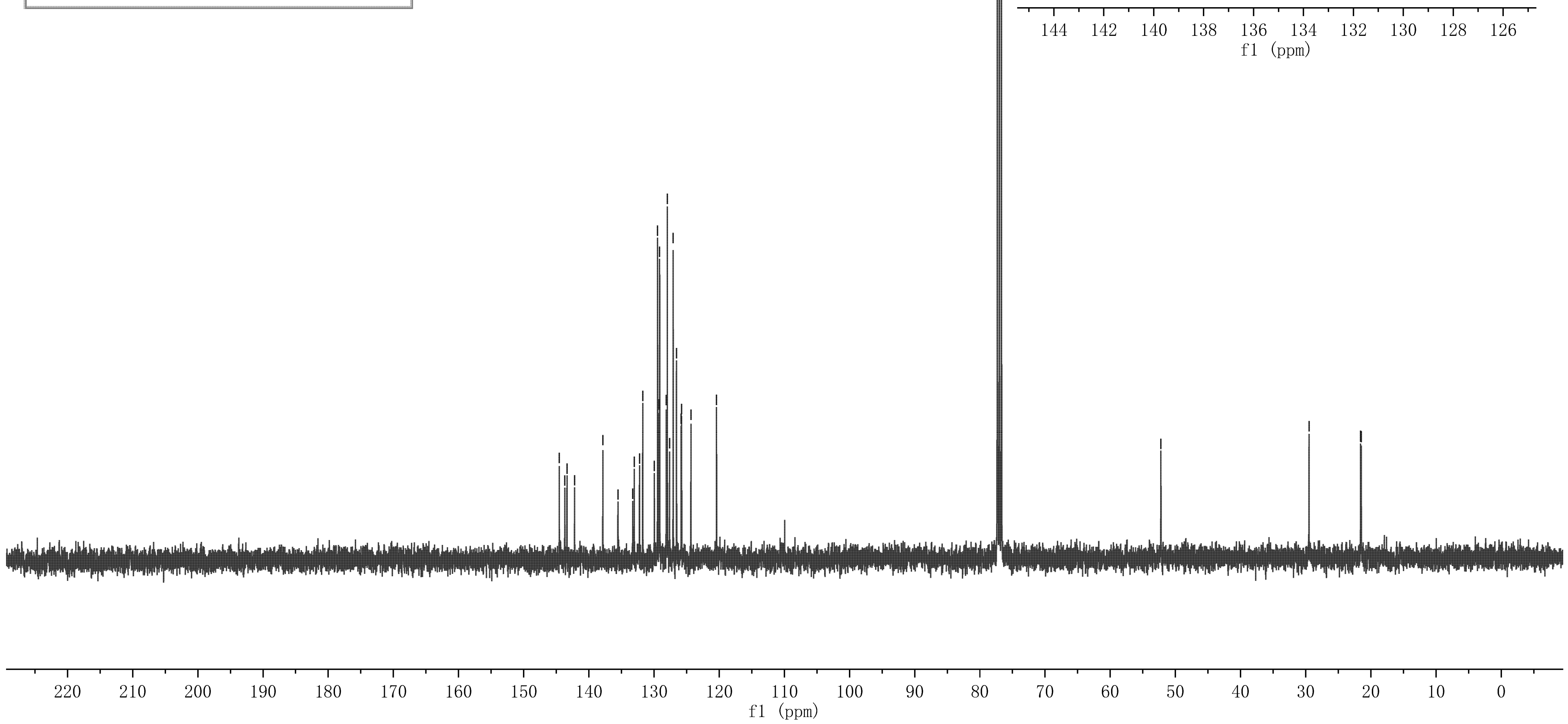

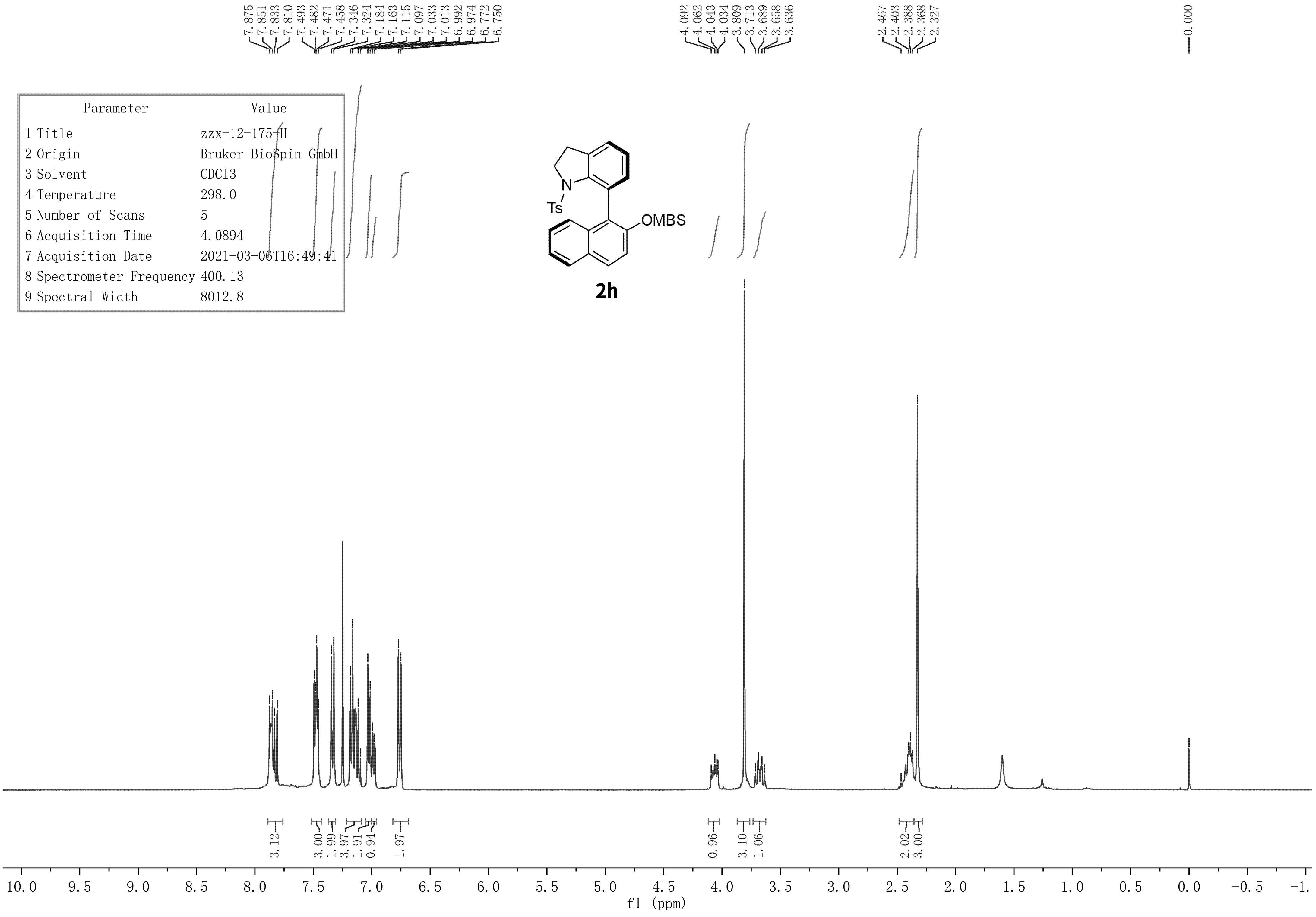

Parameter	Value
1 Title	zzx-12-175-H
2 Origin	Bruker BioSpin GmbH
3 Solvent	CDCl3
4 Temperature	298.0
5 Number of Scans	5
6 Acquisition Time	4.0894
7 Acquisition Date	2021-03-06T16:49:41
8 Spectrometer Frequency	400.13
9 Spectral Width	8012.8

2h

Parameter	Value
1 Title	zzx-12-175-C
2 Origin	Bruker BioSpin GmbH
3 Solvent	CDCl3
4 Temperature	300.0
5 Number of Scans	53
6 Acquisition Time	1.3631
7 Acquisition Date	2021-03-06T16:51:32
8 Spectrometer Frequency	100.61
9 Spectral Width	24038.5

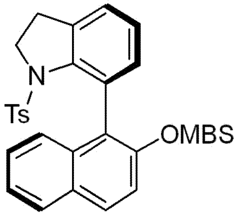

2h

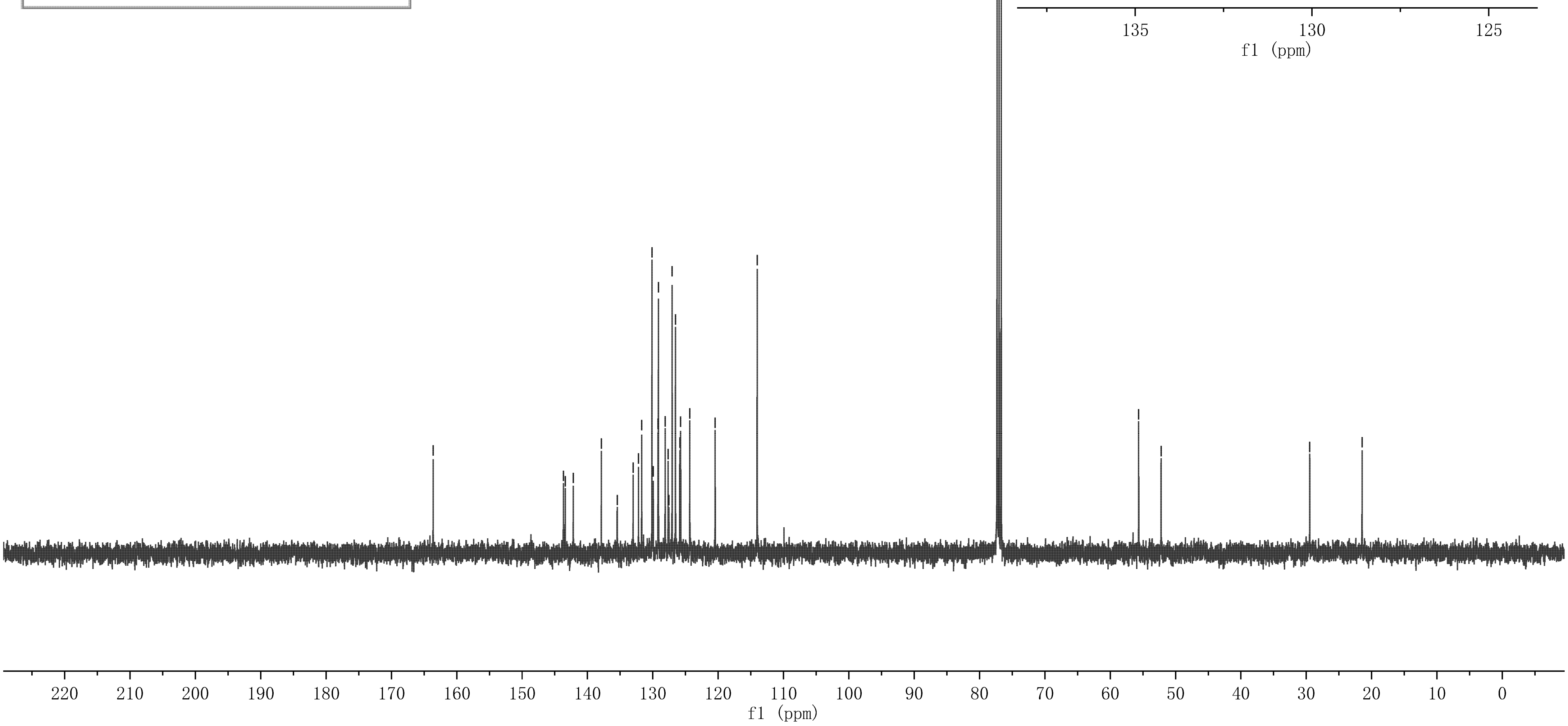

Parameter	Value
1 Title	zzx-11-165-H
2 Origin	Bruker BioSpin GmbH
3 Solvent	CDCl3
4 Temperature	298.0
5 Number of Scans	10
6 Acquisition Time	4.0894
7 Acquisition Date	2020-11-29T14:41:44
8 Spectrometer Frequency	400.13
9 Spectral Width	8012.8

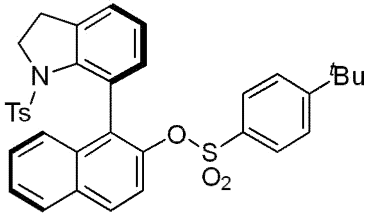

2i

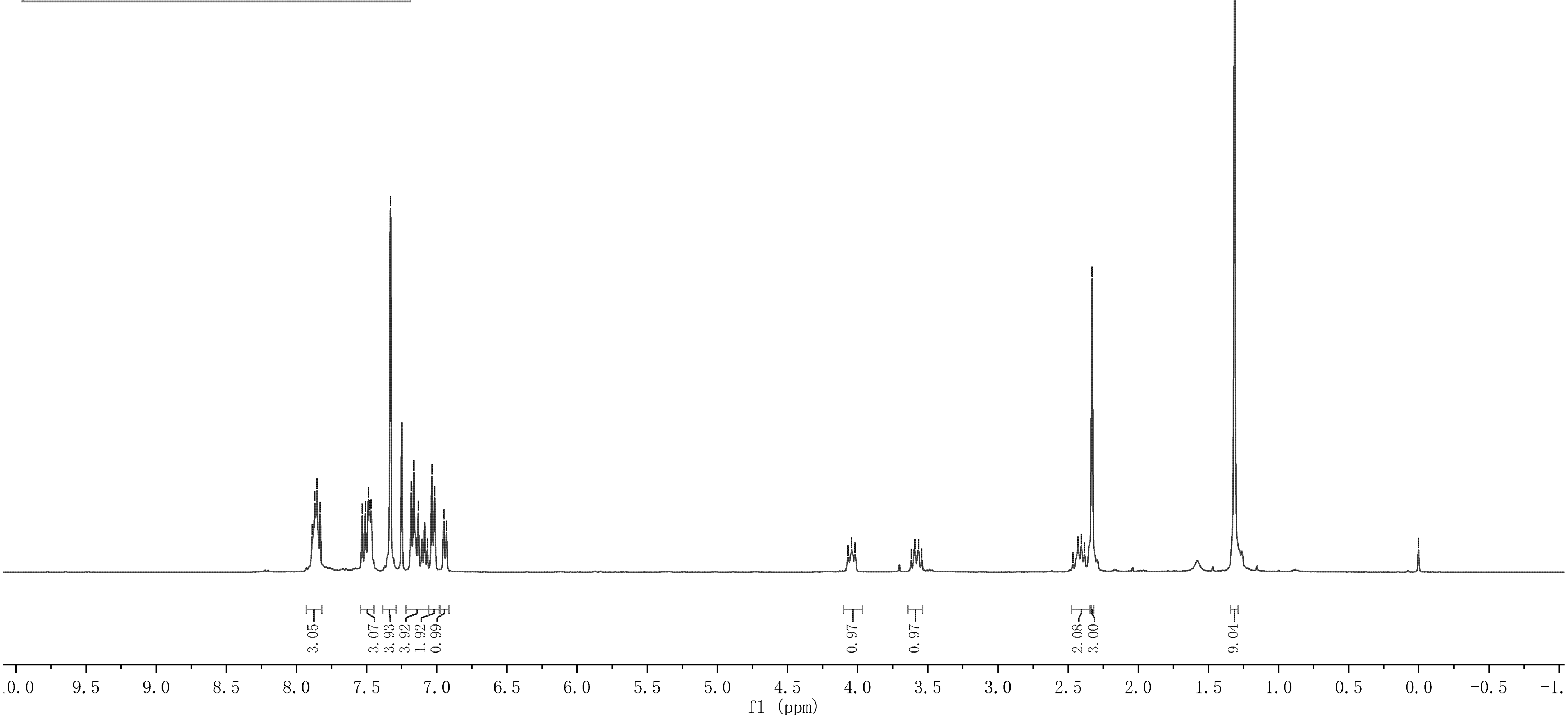

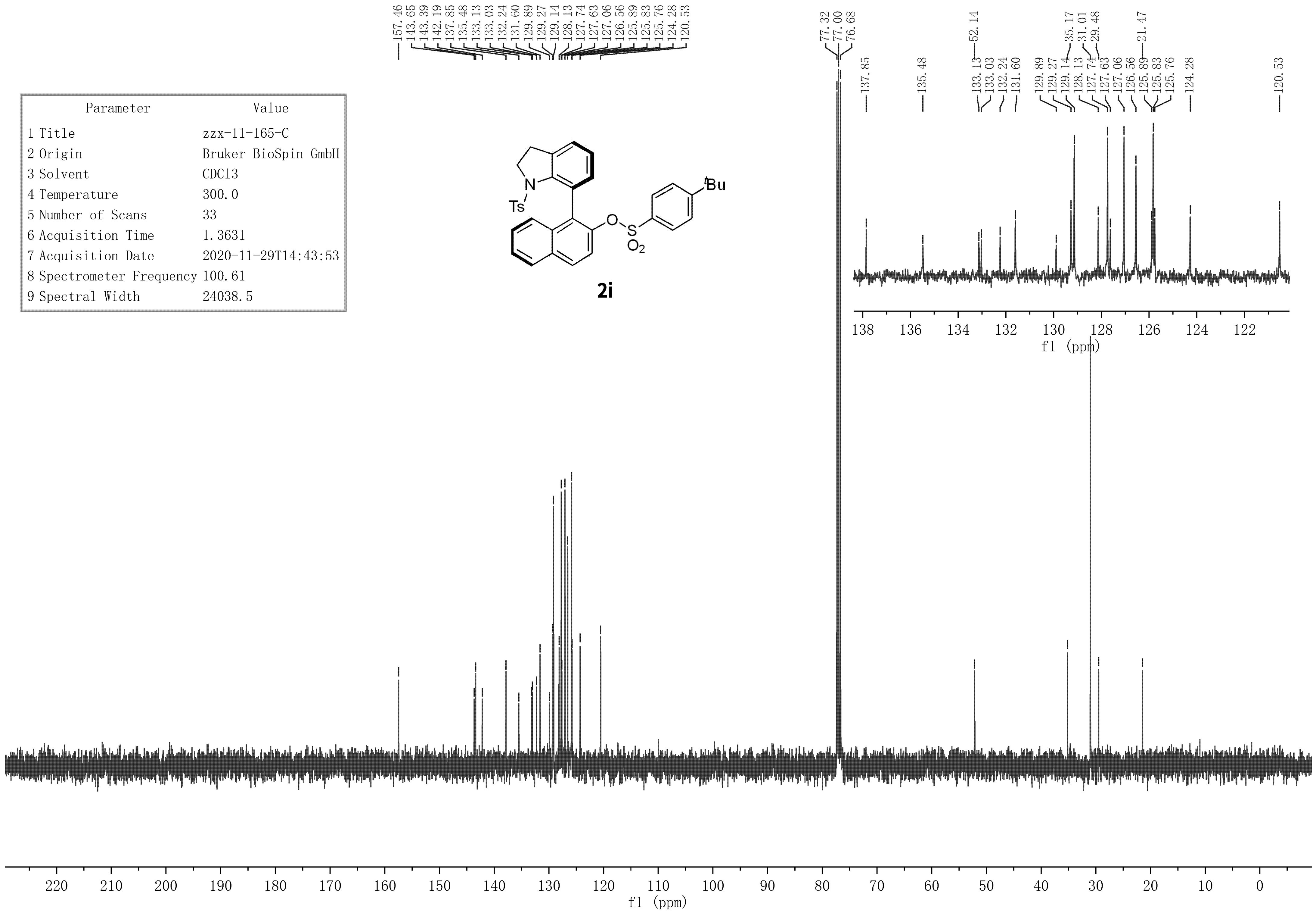
157.46
143.65
143.39
142.19
137.85
135.48
133.13
133.03
132.24
131.60
129.89
129.27
129.14
128.13
127.74
127.63
127.06
126.56
125.89
125.83
125.76
124.28
120.53
77.32
77.00
76.68
52.14
35.17
31.01
29.48
21.47
Parameter Value
1 Title zzx-11-165-C
2 Origin Bruker BioSpin GmbH
3 Solvent CDCl3
4 Temperature 300.0
5 Number of Scans 33
6 Acquisition Time 1.3631
7 Acquisition Date 2020-11-29T14:43:53
8 Spectrometer Frequency 100.61
9 Spectral Width 24038.5
N
Ts
O
S
O2
tBu
2i
f1 (ppm)
f1 (ppm)

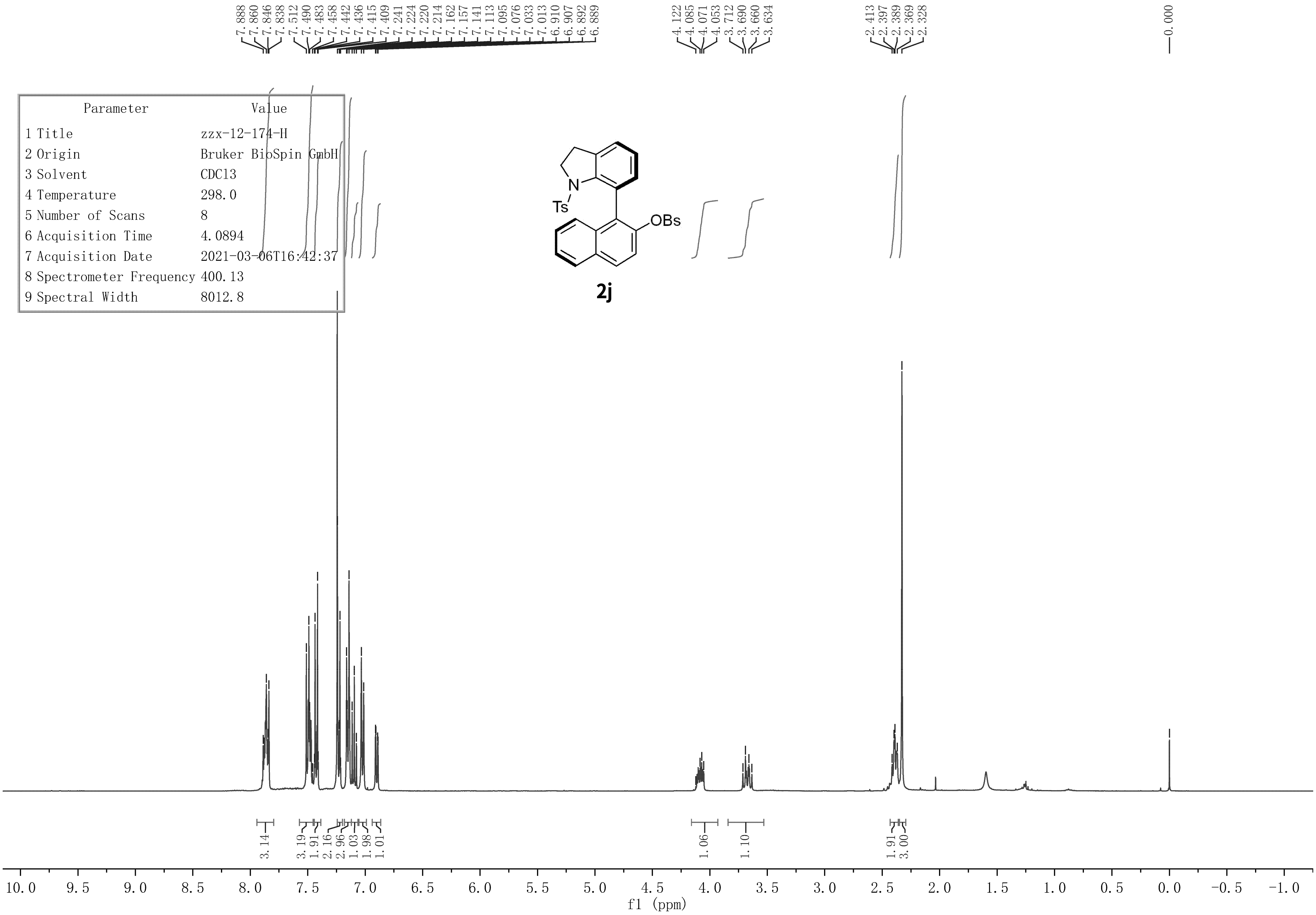
7.888
7.860
7.846
7.838
7.512
7.490
7.483
7.458
7.442
7.436
7.415
7.409
7.241
7.224
7.220
7.214
7.162
7.157
7.141
7.113
7.095
7.076
7.033
7.013
6.910
6.907
6.892
6.889
4.122
4.085
4.071
4.053
3.712
3.690
3.660
3.634
2.413
2.397
2.389
2.369
2.328
0.000
Parameter Value
1 Title zzx-12-174-H
2 Origin Bruker BioSpin GmbH
3 Solvent CDCl3
4 Temperature 298.0
5 Number of Scans 8
6 Acquisition Time 4.0894
7 Acquisition Date 2021-03-06T16:42:37
8 Spectrometer Frequency 400.13
9 Spectral Width 8012.8
N
Ts
OBs
2j
3.14
3.19
1.91
2.16
2.96
1.03
1.98
1.01
1.06
1.10
1.91
3.00
10.0 9.5 9.0 8.5 8.0 7.5 7.0 6.5 6.0 5.5 5.0 4.5 4.0 3.5 3.0 2.5 2.0 1.5 1.0 0.5 0.0 -0.5 -1.0
f1 (ppm)

Parameter	Value
1 Title	zzx-12-174-C
2 Origin	Bruker BioSpin GmbH
3 Solvent	CDCl3
4 Temperature	300.0
5 Number of Scans	39
6 Acquisition Time	1.3631
7 Acquisition Date	2021-03-06T16:44:53
8 Spectrometer Frequency	100.61
9 Spectral Width	24038.5

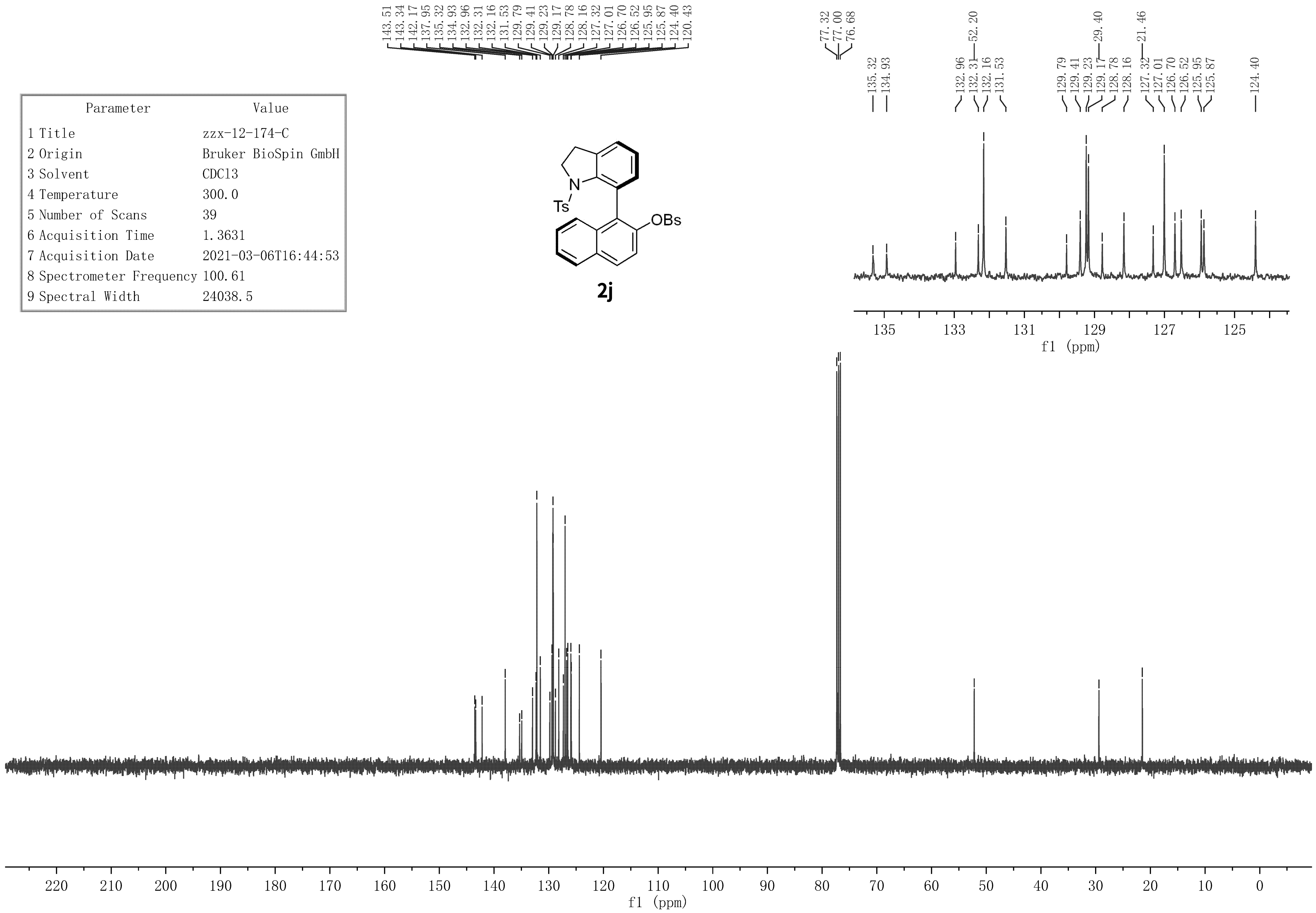

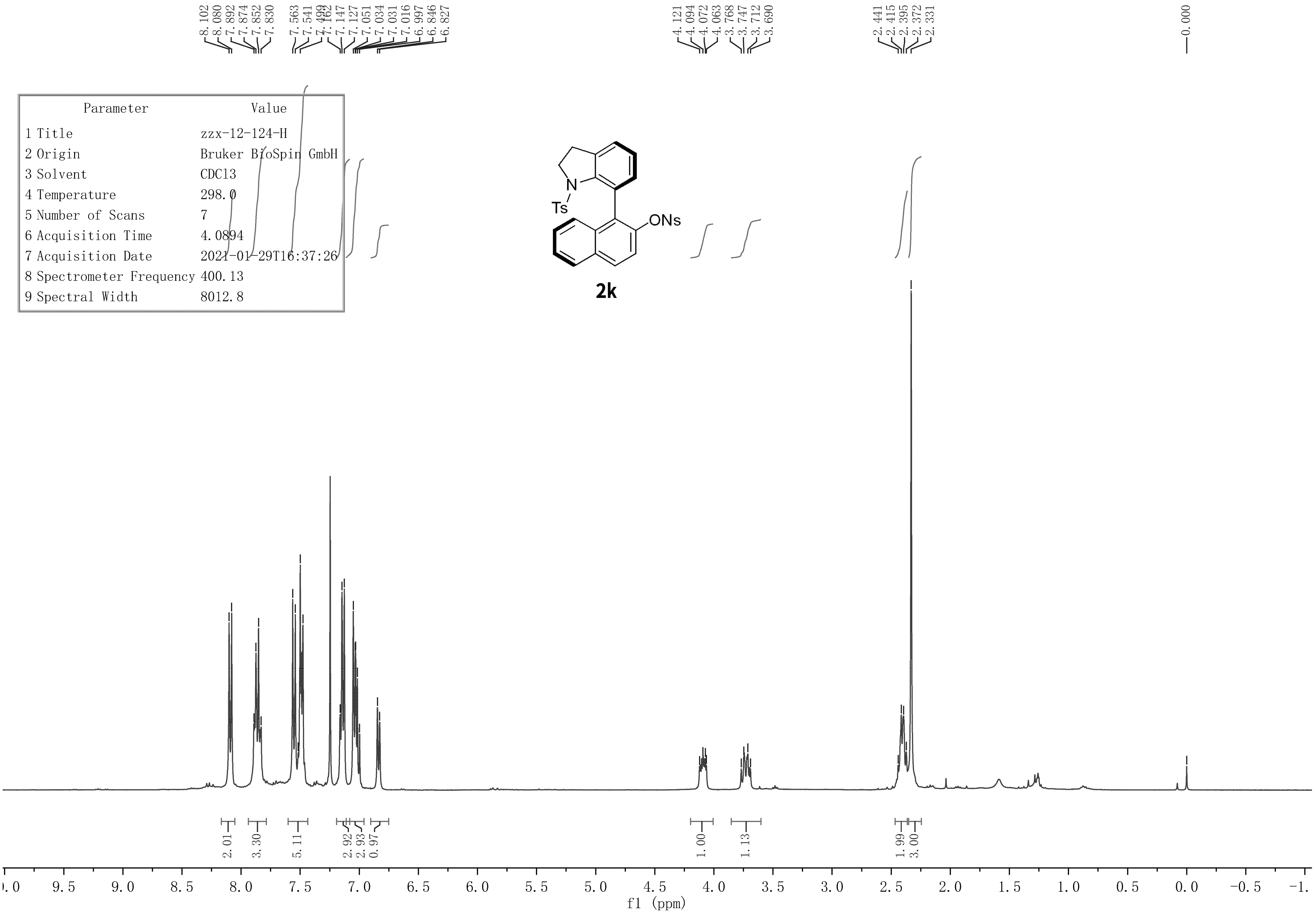

Parameter	Value
1 Title	zzx-12-124-H
2 Origin	Bruker BioSpin GmbH
3 Solvent	CDCl3
4 Temperature	298.0
5 Number of Scans	7
6 Acquisition Time	4.0894
7 Acquisition Date	2021-01-29T16:37:26
8 Spectrometer Frequency	400.13
9 Spectral Width	8012.8

Parameter	Value
1 Title	zzx-12-124-C
2 Origin	Bruker BioSpin GmbH
3 Solvent	CDCl3
4 Temperature	300.0
5 Number of Scans	29
6 Acquisition Time	1.3631
7 Acquisition Date	2021-01-29T16:38:34
8 Spectrometer Frequency	100.61
9 Spectral Width	24038.5

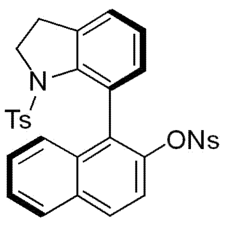

2k

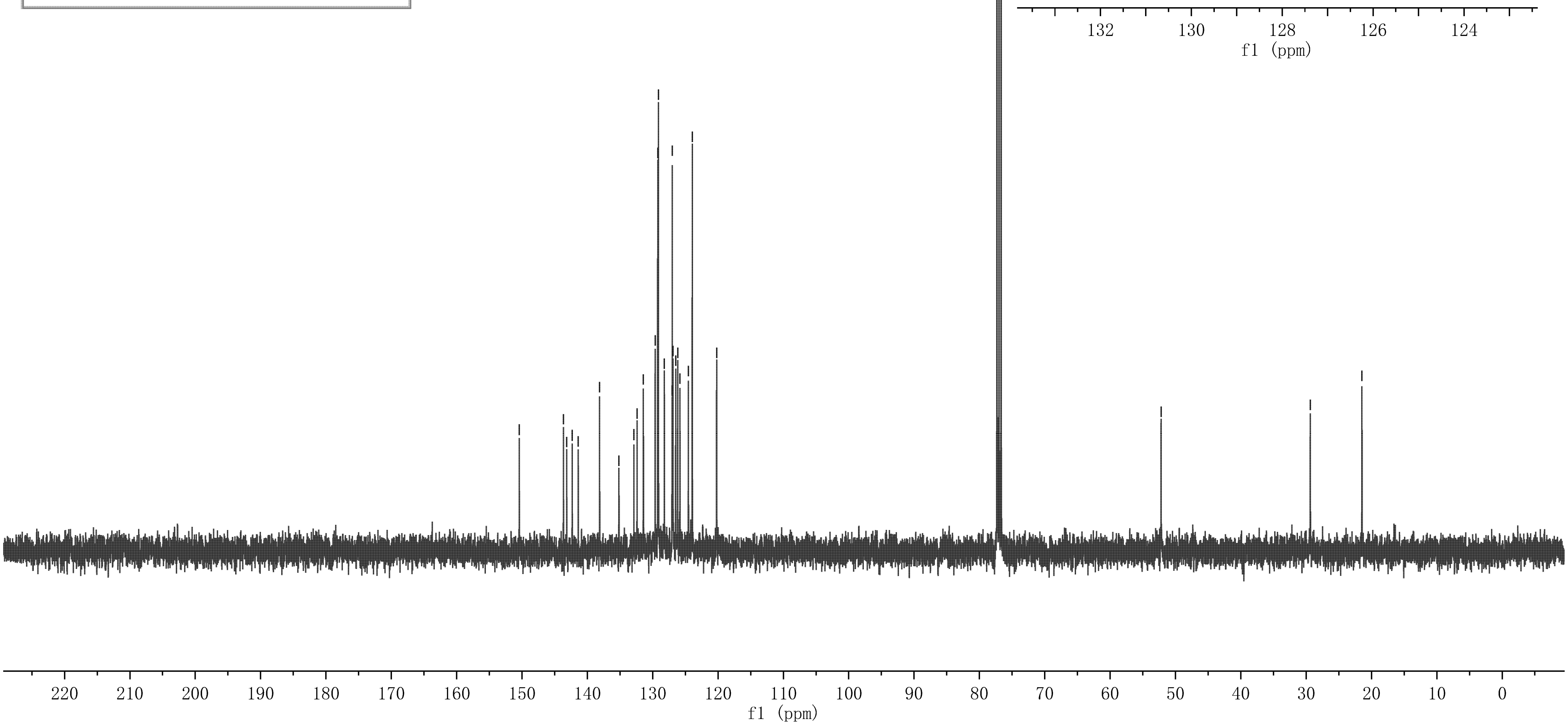

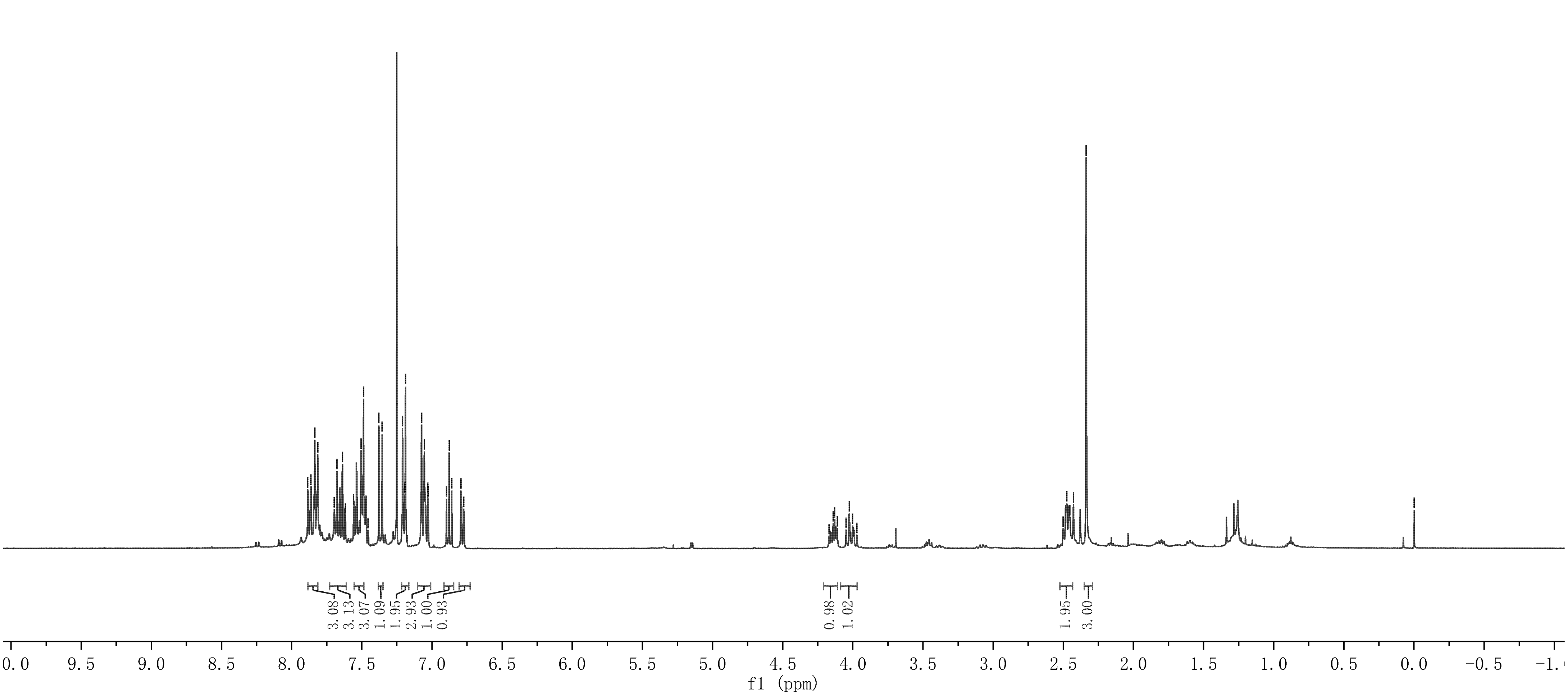

Parameter	Value
1 Title	zzx-12-101-H
2 Origin	Bruker BioSpin GmbH
3 Solvent	CDCl3
4 Temperature	298.0
5 Number of Scans	10
6 Acquisition Time	4.0894
7 Acquisition Date	2021-01-29T16:53:14
8 Spectrometer Frequency	400.13
9 Spectral Width	8012.8

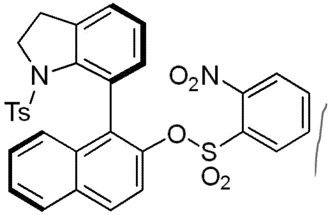

2l

Parameter	Value
1 Title	zzx-12-101-C
2 Origin	Bruker BioSpin GmbH
3 Solvent	CDCl3
4 Temperature	300.0
5 Number of Scans	100
6 Acquisition Time	1.3631
7 Acquisition Date	2021-01-29T16:54:56
8 Spectrometer Frequency	100.62
9 Spectral Width	24038.5

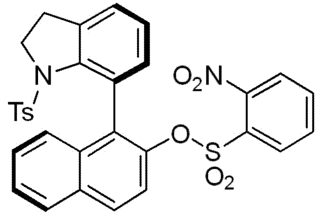

2l

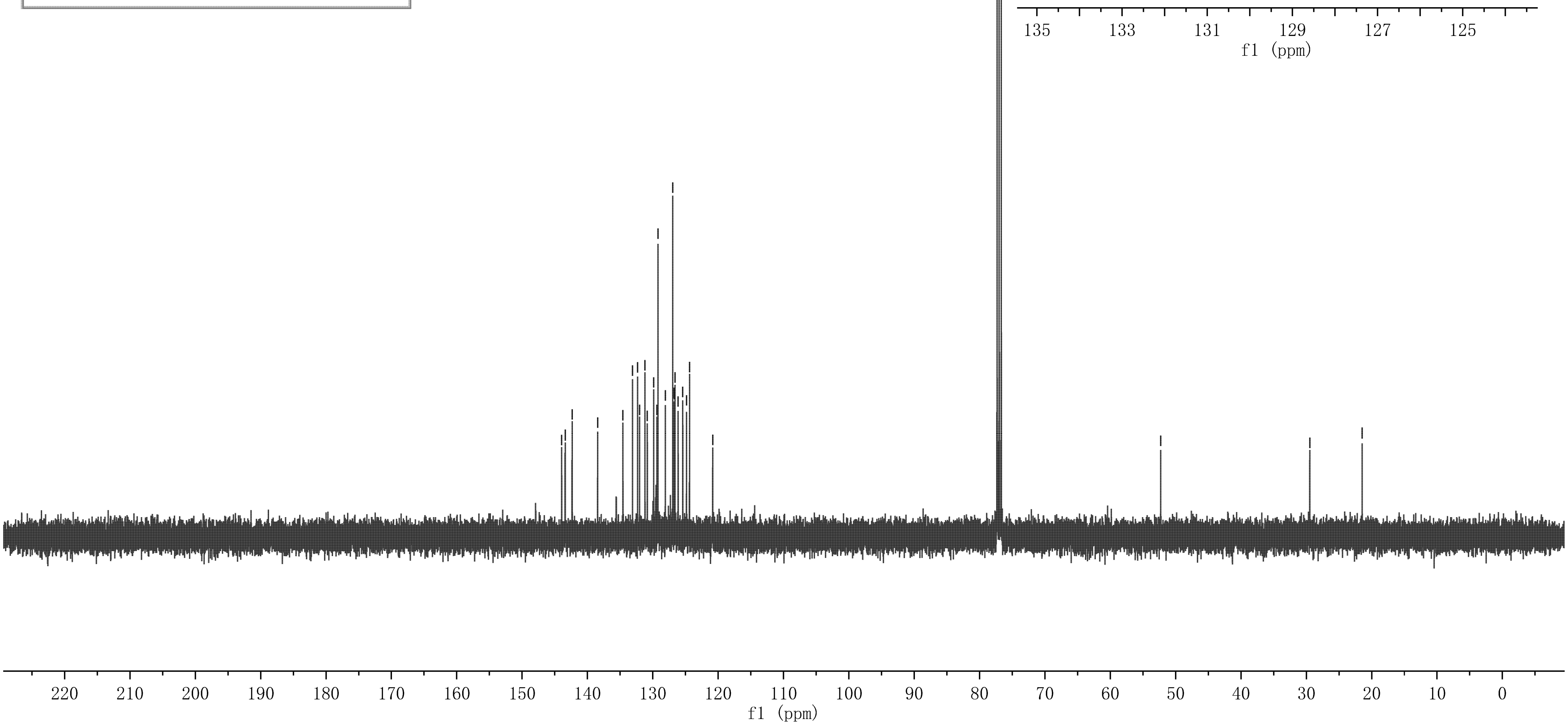

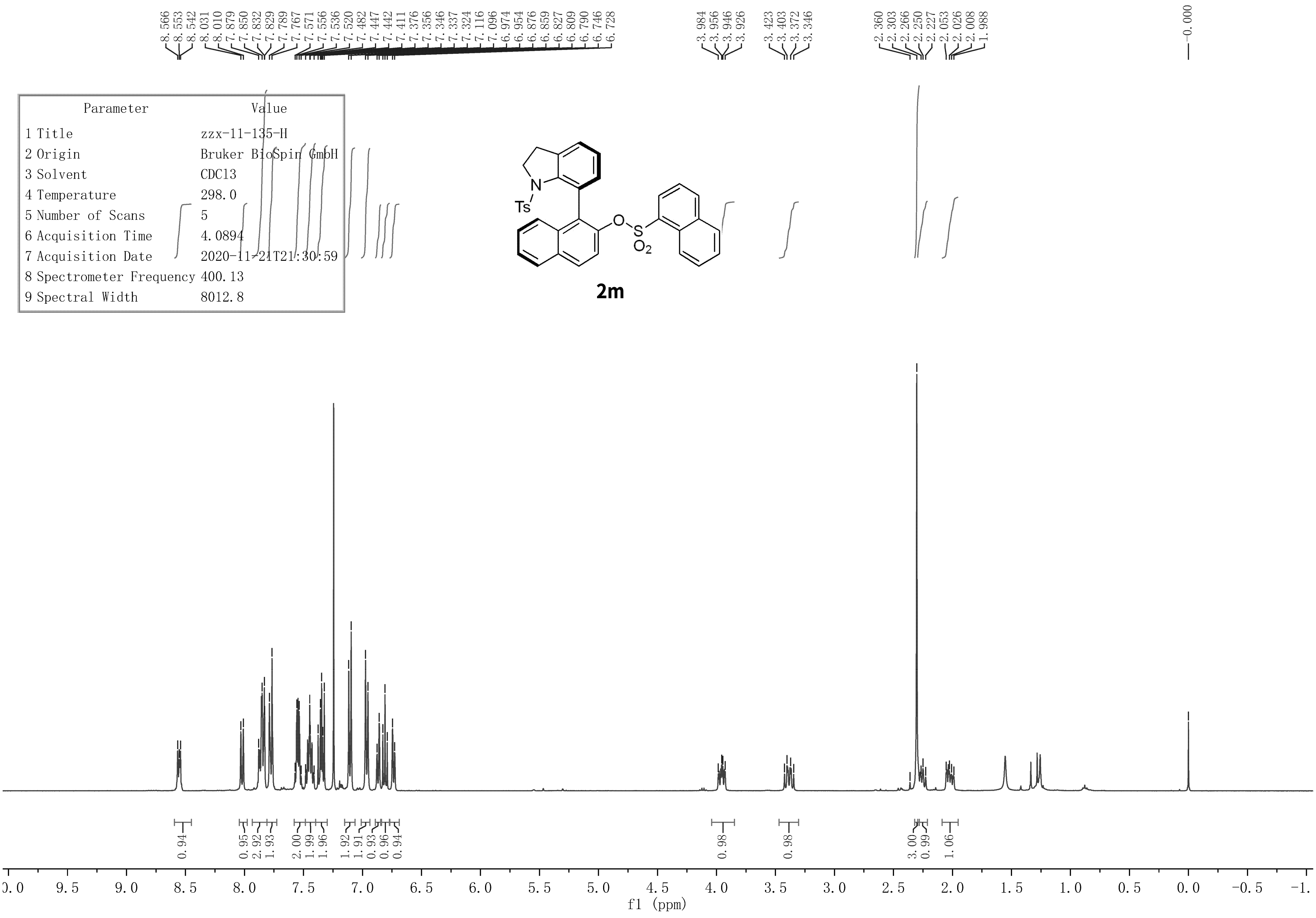
8.566
8.553
8.542
8.031
8.010
7.879
7.850
7.832
7.829
7.789
7.767
7.571
7.556
7.536
7.520
7.482
7.447
7.442
7.411
7.376
7.356
7.346
7.337
7.324
7.116
7.096
6.974
6.954
6.876
6.859
6.827
6.809
6.790
6.746
6.728
3.984
3.956
3.946
3.926
3.423
3.403
3.372
3.346
2.360
2.303
2.266
2.250
2.227
2.053
2.026
2.008
1.988
-0.000
Parameter Value
1 Title zzx-11-135-H
2 Origin Bruker BioSpin GmbH
3 Solvent CDCl3
4 Temperature 298.0
5 Number of Scans 5
6 Acquisition Time 4.0894
7 Acquisition Date 2020-11-21T21:30:59
8 Spectrometer Frequency 400.13
9 Spectral Width 8012.8
0.94
0.95
2.92
1.93
2.00
1.99
1.96
1.92
1.91
0.93
0.96
0.94
0.98
0.98
3.00
0.99
1.06
0.0
9.5
9.0
8.5
8.0
7.5
7.0
6.5
6.0
5.5
5.0
4.5
4.0
3.5
3.0
2.5
2.0
1.5
1.0
0.5
0.0
-0.5
-1.
f1 (ppm)
N
Ts
O
S
O2

2m

Parameter	Value
1 Title	zzx-11-135-C
2 Origin	Bruker BioSpin GmbH
3 Solvent	CDCl3
4 Temperature	300.0
5 Number of Scans	71
6 Acquisition Time	1.3631
7 Acquisition Date	2020-11-21T21:32:26
8 Spectrometer Frequency	100.61
9 Spectral Width	24038.5

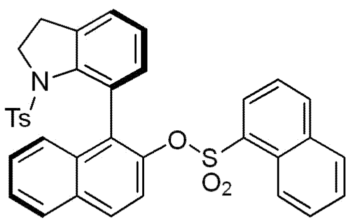

2m

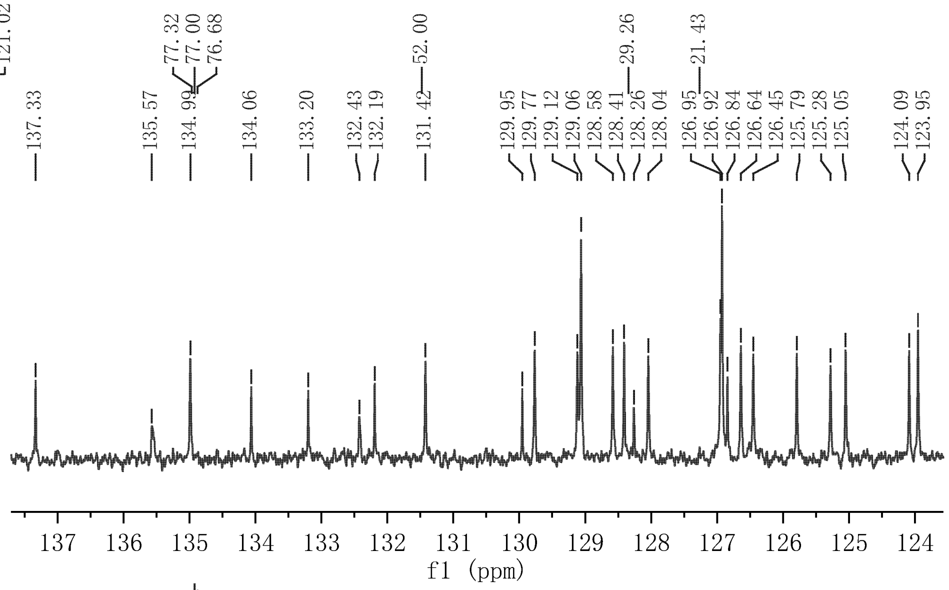

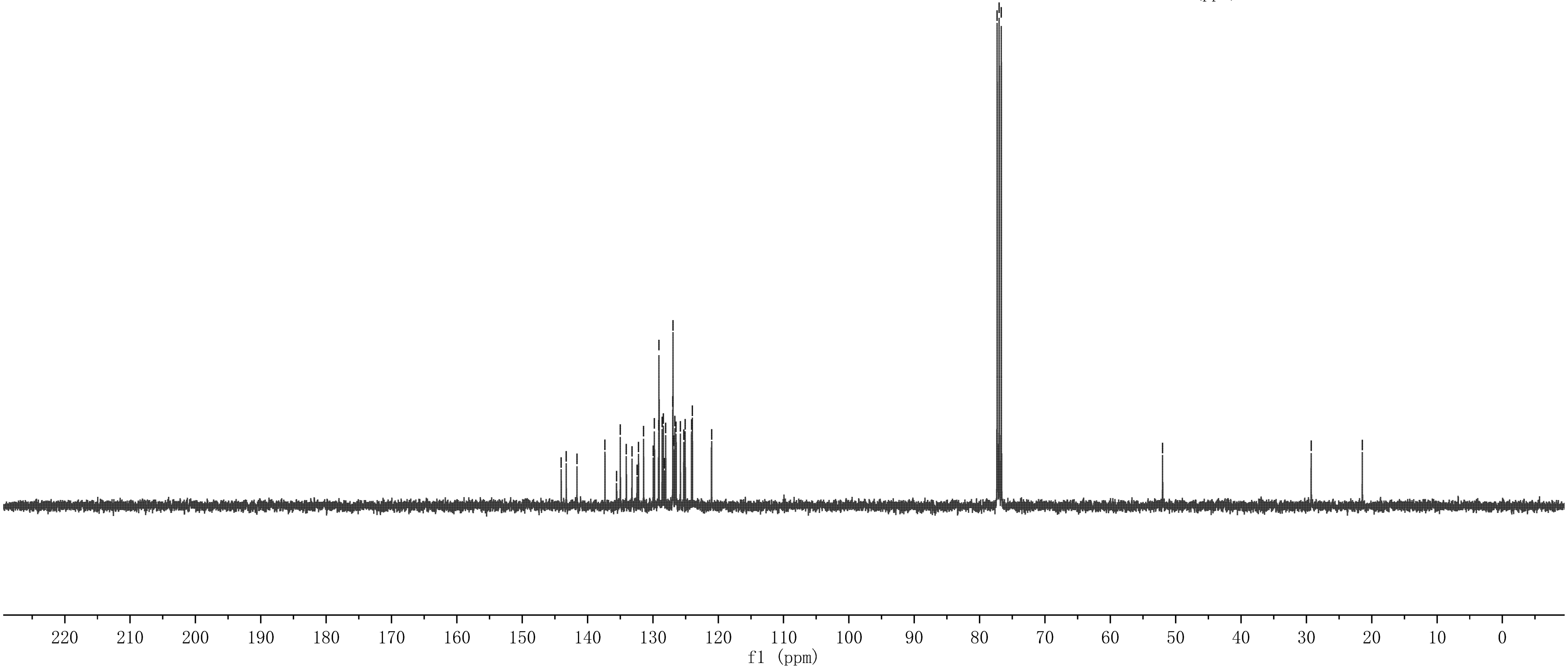

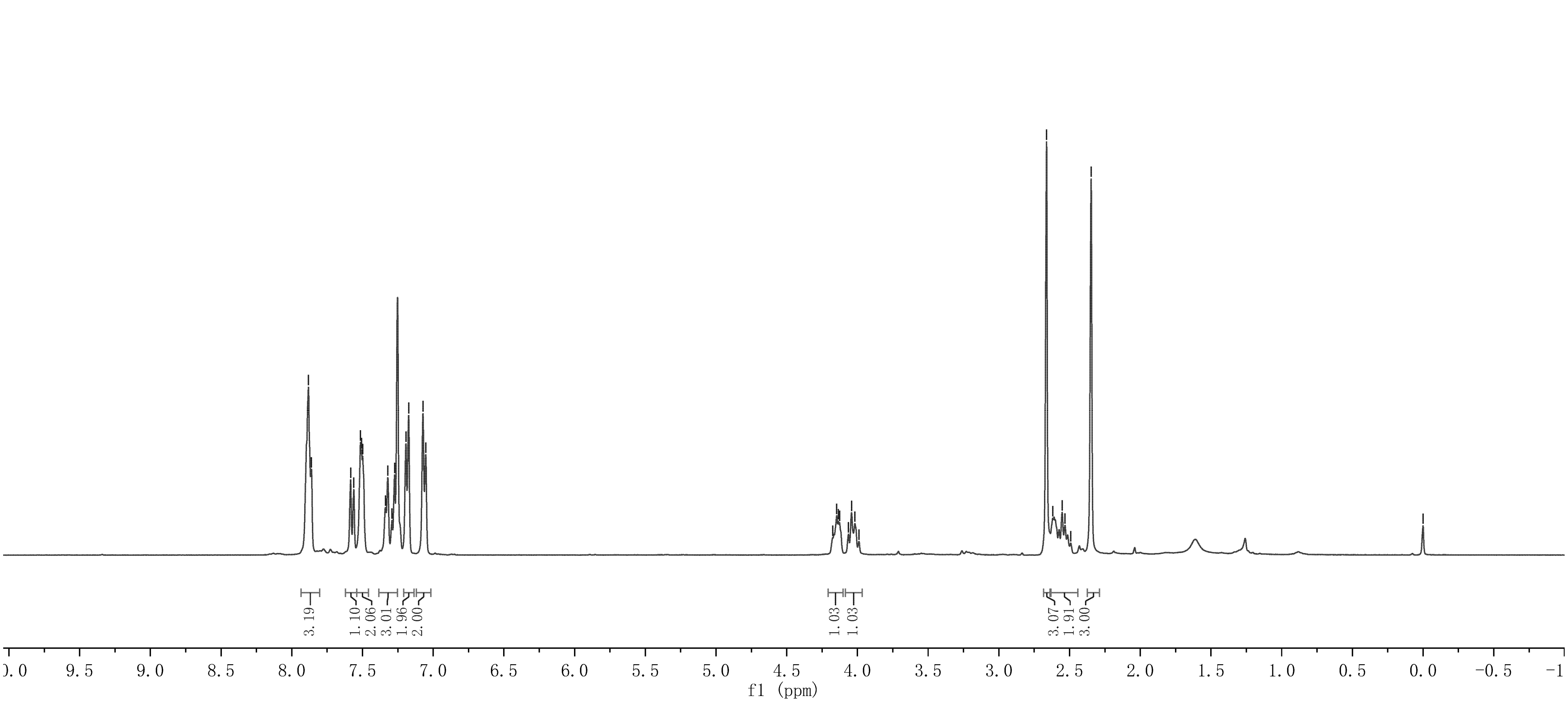

Parameter	Value
1 Title	zzx-12-193
2 Origin	Bruker BioSpin GmbH
3 Solvent	CDCl3
4 Temperature	298.0
5 Number of Scans	7
6 Acquisition Time	4.0894
7 Acquisition Date	2021-03-15T16:19:21
8 Spectrometer Frequency	400.13
9 Spectral Width	8012.8

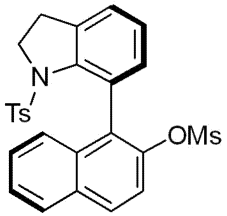

2n

Parameter	Value
1 Title	zzx-12-193-C
2 Origin	Bruker BioSpin GmbH
3 Solvent	CDCl3
4 Temperature	300.0
5 Number of Scans	86
6 Acquisition Time	1.3631
7 Acquisition Date	2021-03-15T16:21:26
8 Spectrometer Frequency	100.61
9 Spectral Width	24038.5

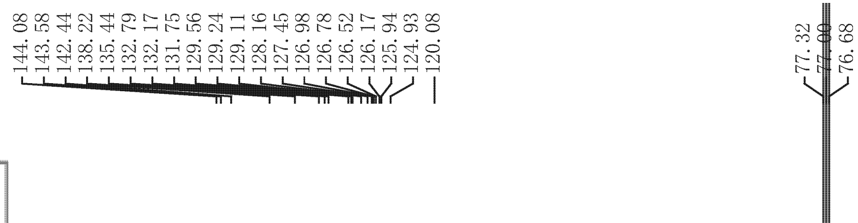

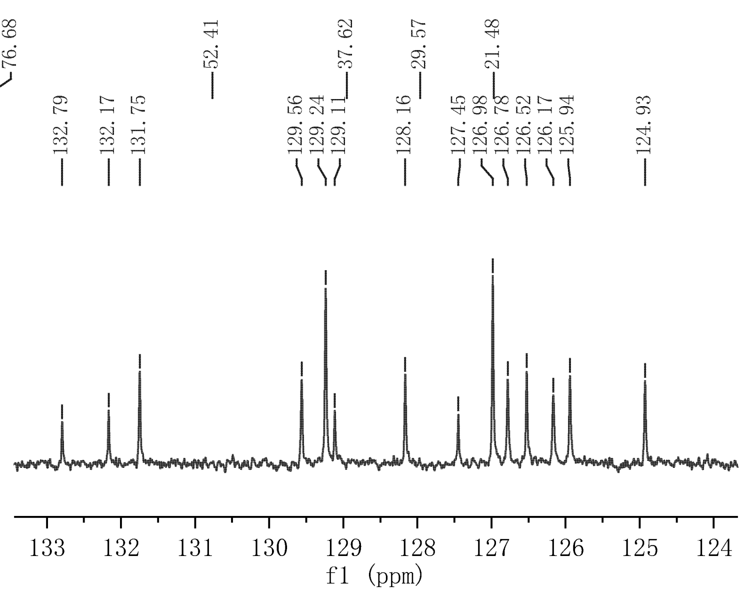

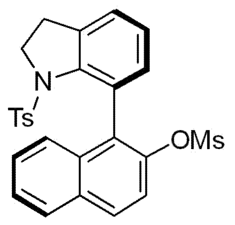

2n

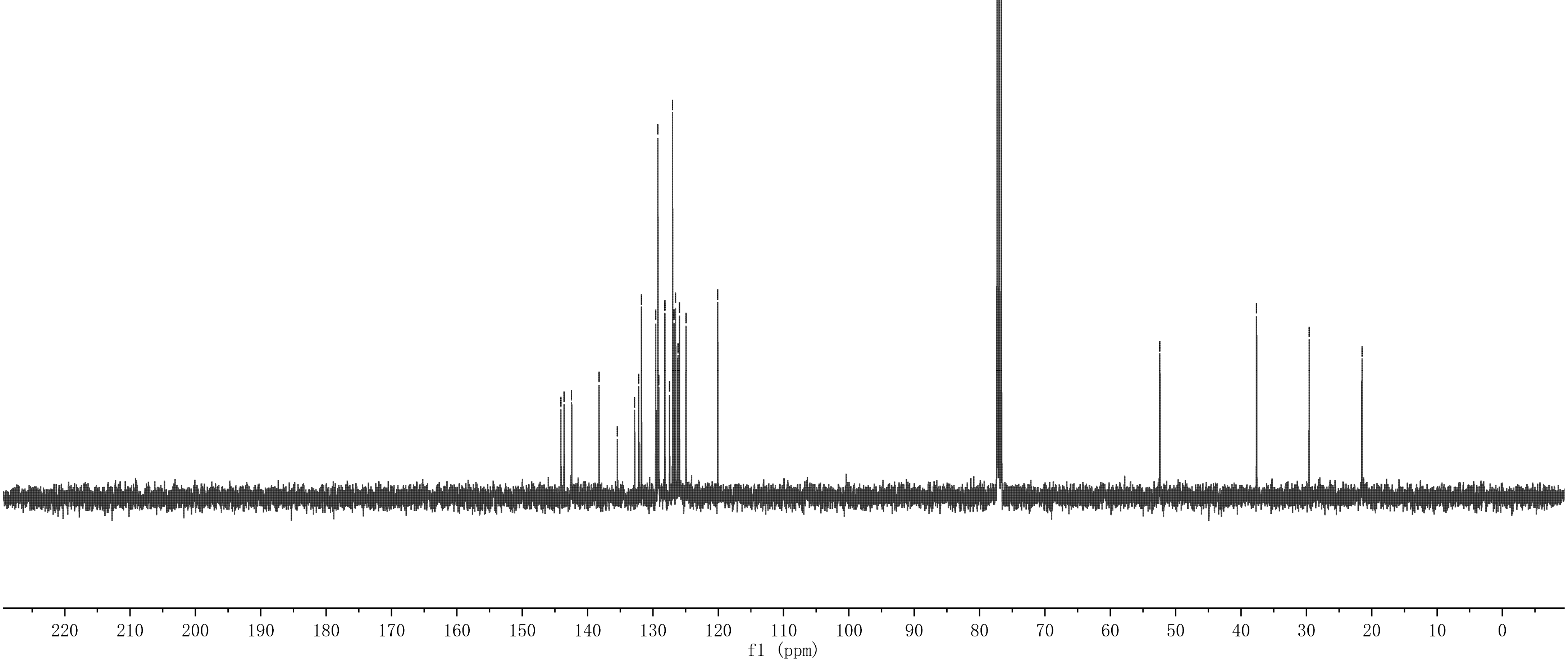

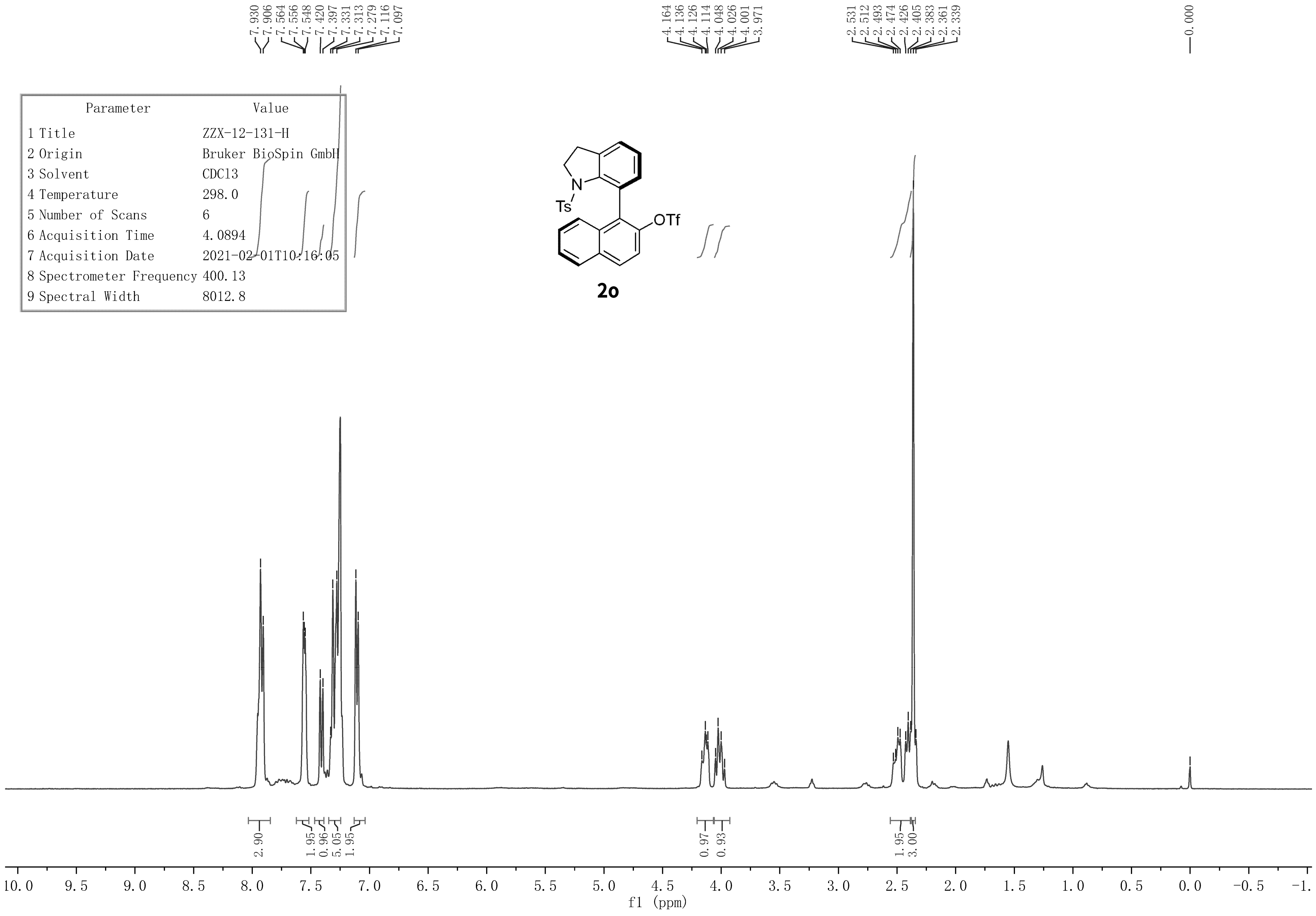
7.930
7.906
7.564
7.556
7.548
7.420
7.397
7.331
7.313
7.279
7.116
7.097
4.164
4.136
4.126
4.114
4.048
4.026
4.001
3.971
2.531
2.512
2.493
2.474
2.426
2.405
2.383
2.361
2.339
0.000
Parameter Value
1 Title ZZX-12-131-H
2 Origin Bruker BioSpin GmbH
3 Solvent CDCl3
4 Temperature 298.0
5 Number of Scans 6
6 Acquisition Time 4.0894
7 Acquisition Date 2021-02-01T10:16:05
8 Spectrometer Frequency 400.13
9 Spectral Width 8012.8
N
Ts
OTf
2o
2.90
1.95
0.96
5.05
1.95
0.97
0.93
1.95
3.00
10.0 9.5 9.0 8.5 8.0 7.5 7.0 6.5 6.0 5.5 5.0 4.5 4.0 3.5 3.0 2.5 2.0 1.5 1.0 0.5 0.0 -0.5 -1.
f1 (ppm)

Parameter	Value
1 Title	ZZX-12-131-C
2 Origin	Bruker BioSpin GmbH
3 Solvent	CDCl3
4 Temperature	300.0
5 Number of Scans	91
6 Acquisition Time	1.3631
7 Acquisition Date	2021-02-01T10:19:19
8 Spectrometer Frequency	100.61
9 Spectral Width	24038.5

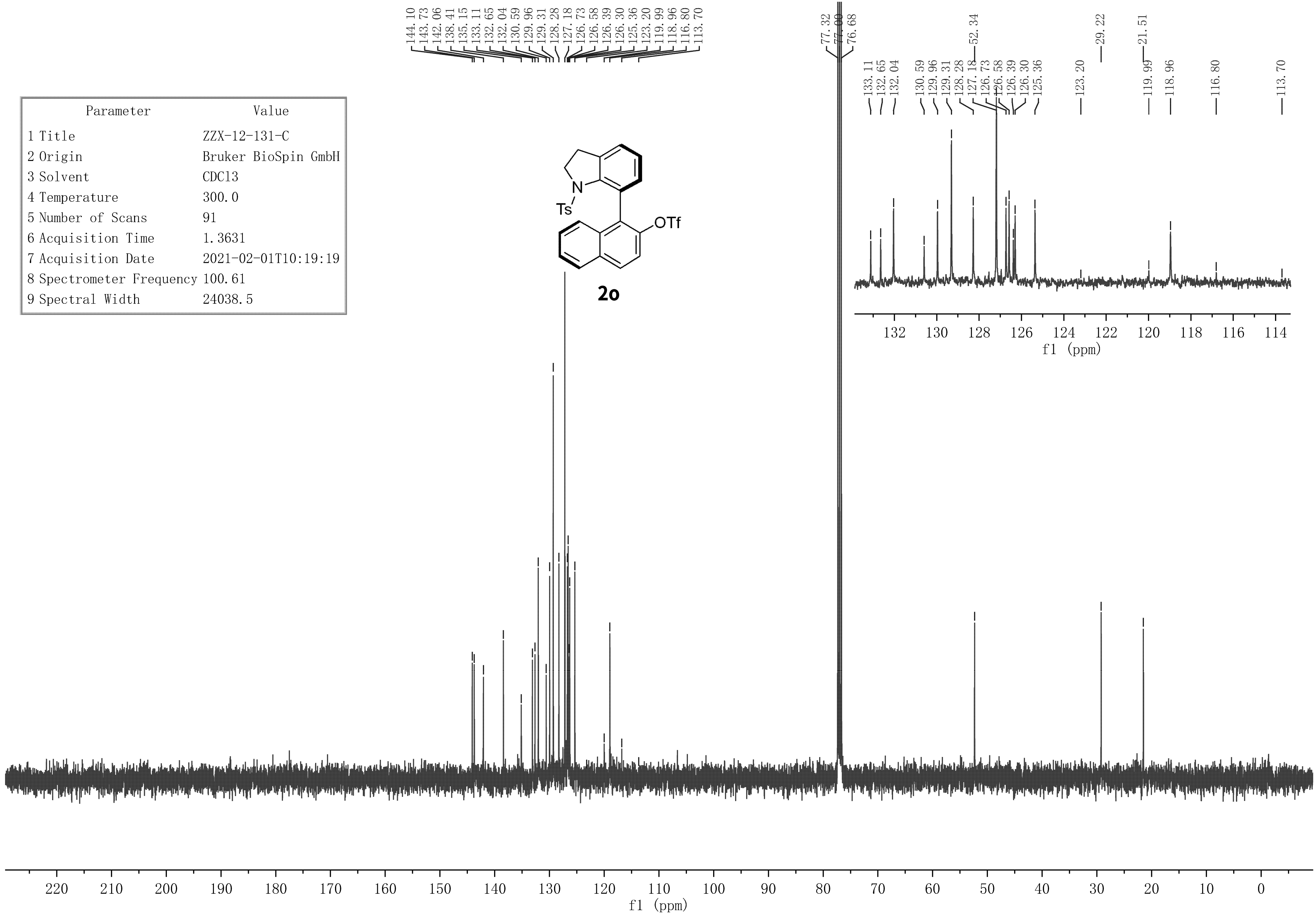

−74.46

Parameter	Value
1 Title	zzx-13-211-F-1
2 Origin	Bruker BioSpin GmbH
3 Solvent	CDCl3
4 Temperature	294.9
5 Number of Scans	16
6 Acquisition Time	0.7340
7 Acquisition Date	2021-11-27T10:11:10
8 Spectrometer Frequency	376.31
9 Spectral Width	89285.7

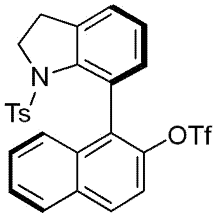

2o

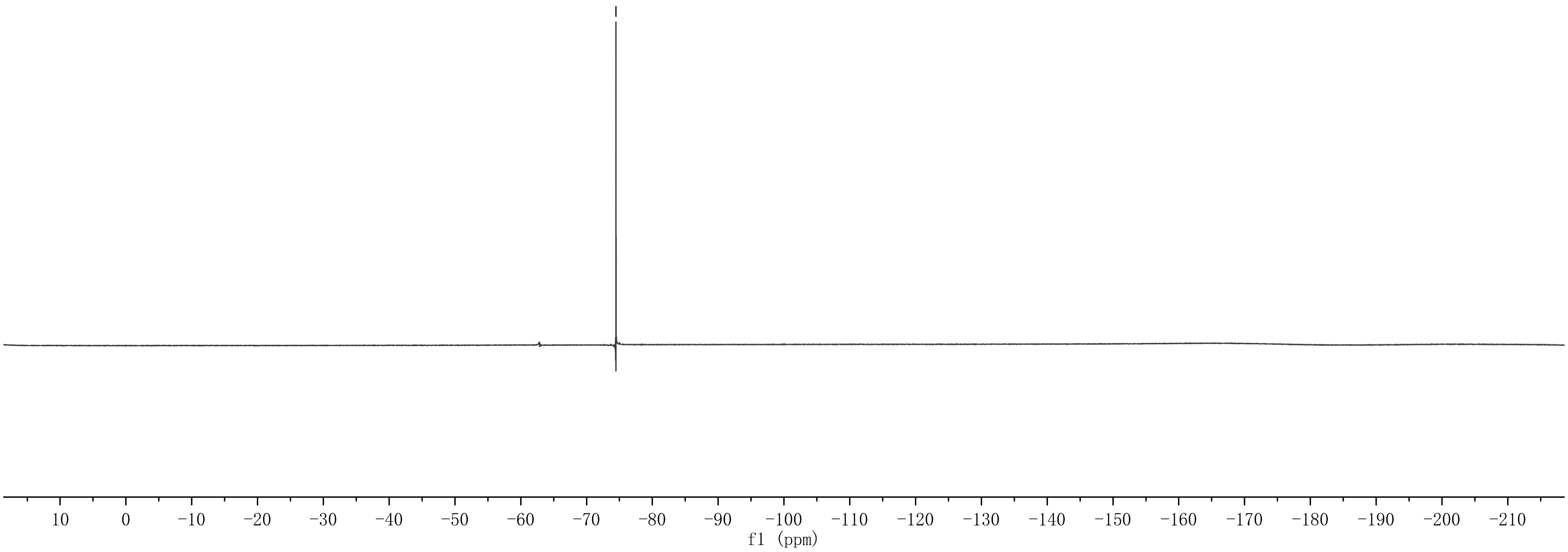

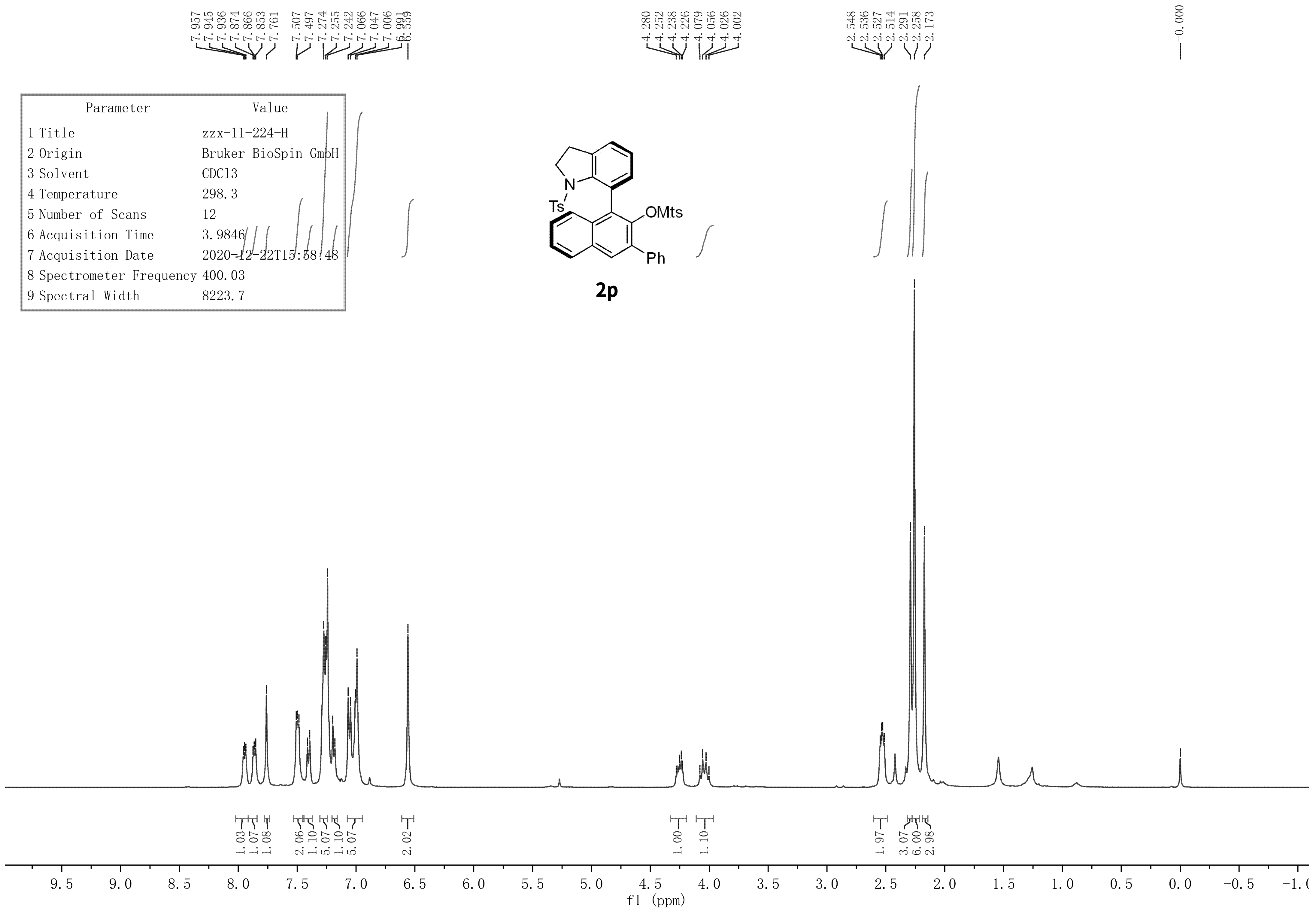
7.957
7.945
7.936
7.874
7.866
7.853
7.761
7.507
7.497
7.274
7.255
7.242
7.066
7.047
7.006
6.991
6.559
4.280
4.252
4.238
4.226
4.079
4.056
4.026
4.002
2.548
2.536
2.527
2.514
2.291
2.258
2.173
-0.000
Parameter Value
1 Title zzx-11-224-H
2 Origin Bruker BioSpin GmbH
3 Solvent CDCl3
4 Temperature 298.3
5 Number of Scans 12
6 Acquisition Time 3.9846
7 Acquisition Date 2020-12-22T15:58:48
8 Spectrometer Frequency 400.03
9 Spectral Width 8223.7
N
Ts
OMts
Ph
2p
1.03
1.07
1.08
2.06
1.10
5.07
1.10
5.07
2.02
1.00
1.10
1.97
3.07
6.00
2.98
9.5 9.0 8.5 8.0 7.5 7.0 6.5 6.0 5.5 5.0 4.5 4.0 3.5 3.0 2.5 2.0 1.5 1.0 0.5 0.0 -0.5 -1.0
f1 (ppm)

Parameter	Value
1 Title	zzx-11-224-C-1
2 Origin	Bruker BioSpin GmbH
3 Solvent	CDCl3
4 Temperature	299.2
5 Number of Scans	73
6 Acquisition Time	1.3631
7 Acquisition Date	2020-12-22T16:02:54
8 Spectrometer Frequency	100.59
9 Spectral Width	24038.5

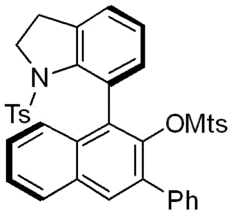

2p

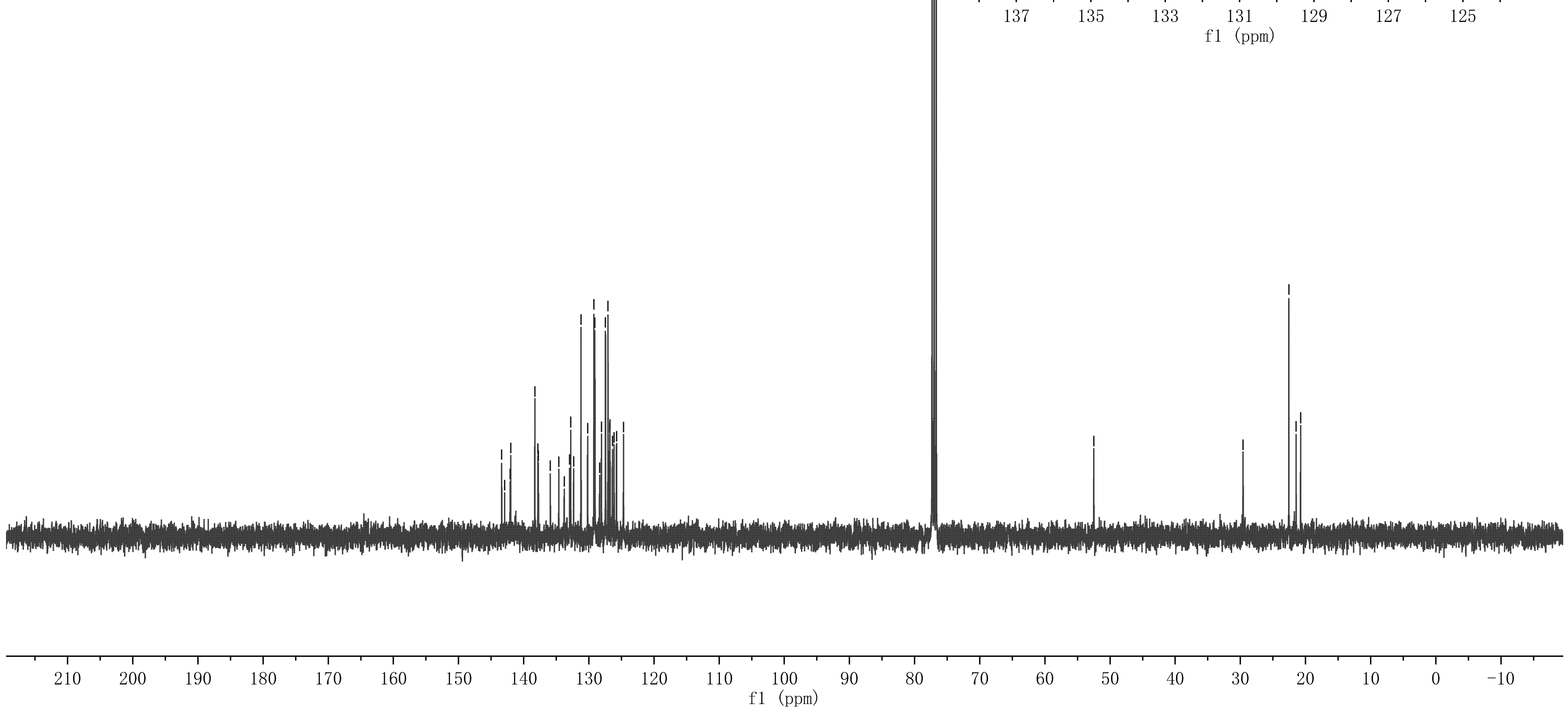

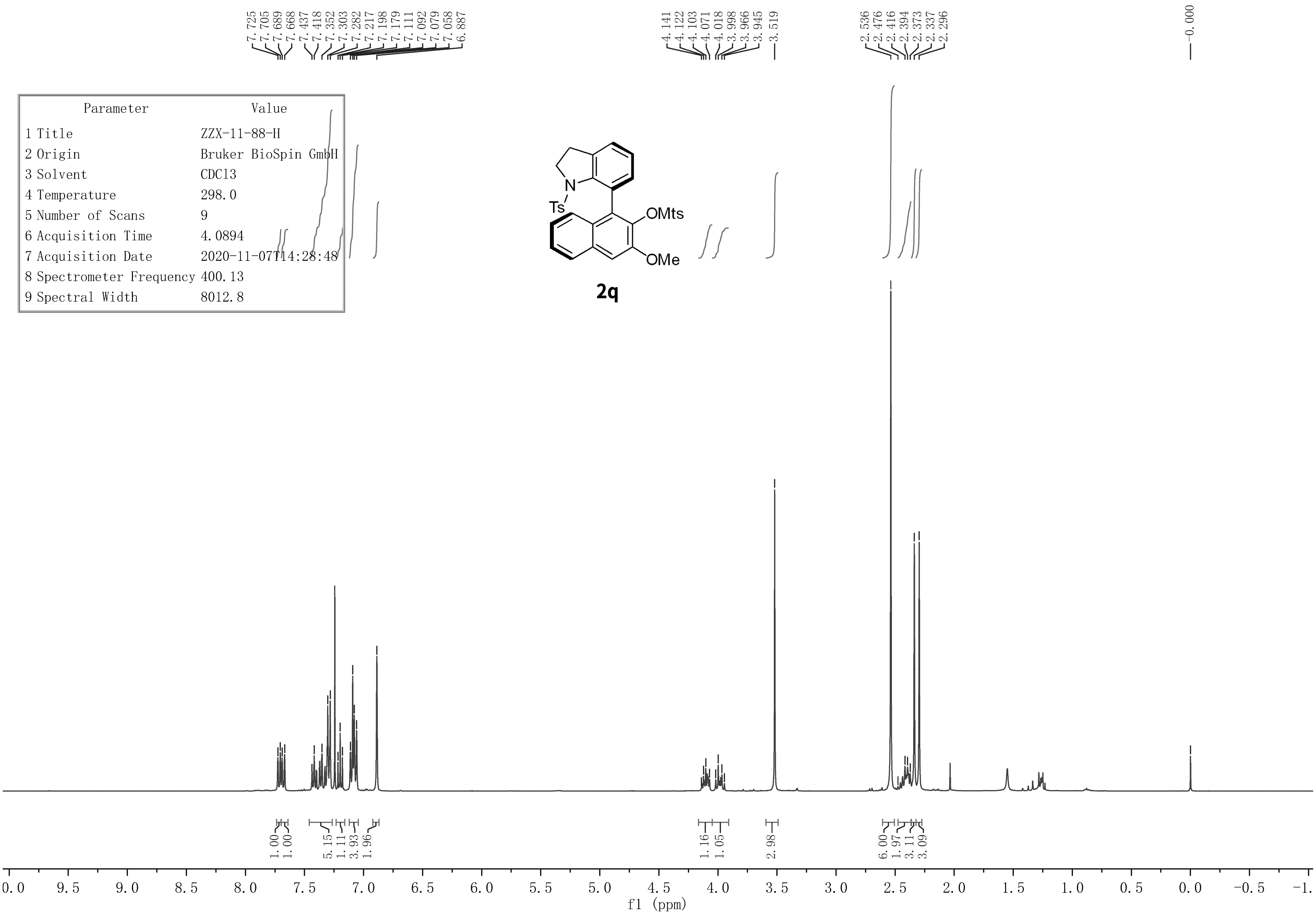
Parameter Value
1 Title ZZX-11-88-H
2 Origin Bruker BioSpin GmbH
3 Solvent CDCl3
4 Temperature 298.0
5 Number of Scans 9
6 Acquisition Time 4.0894
7 Acquisition Date 2020-11-07T14:28:48
8 Spectrometer Frequency 400.13
9 Spectral Width 8012.8
7.725 7.705 7.689 7.668 7.437 7.418 7.352 7.303 7.282 7.217 7.198 7.179 7.111 7.092 7.079 7.058 6.887
4.141 4.122 4.103 4.071 4.018 3.998 3.966 3.945 3.519
2.536 2.476 2.416 2.394 2.373 2.337 2.296
0.000
N
Ts
OMts
OMe
2q
1.00 1.00 5.15 1.11 3.93 1.96 1.16 1.05 2.98 6.00 1.97 3.11 3.09
0.0 9.5 9.0 8.5 8.0 7.5 7.0 6.5 6.0 5.5 5.0 4.5 4.0 3.5 3.0 2.5 2.0 1.5 1.0 0.5 0.0 -0.5 -1.
f1 (ppm)

Parameter	Value
1 Title	ZZX-11-88-C
2 Origin	Bruker BioSpin GmbH
3 Solvent	CDCl3
4 Temperature	300.0
5 Number of Scans	35
6 Acquisition Time	1.3631
7 Acquisition Date	2020-11-07T14:30:33
8 Spectrometer Frequency	100.61
9 Spectral Width	24038.5

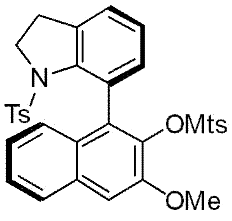

2q

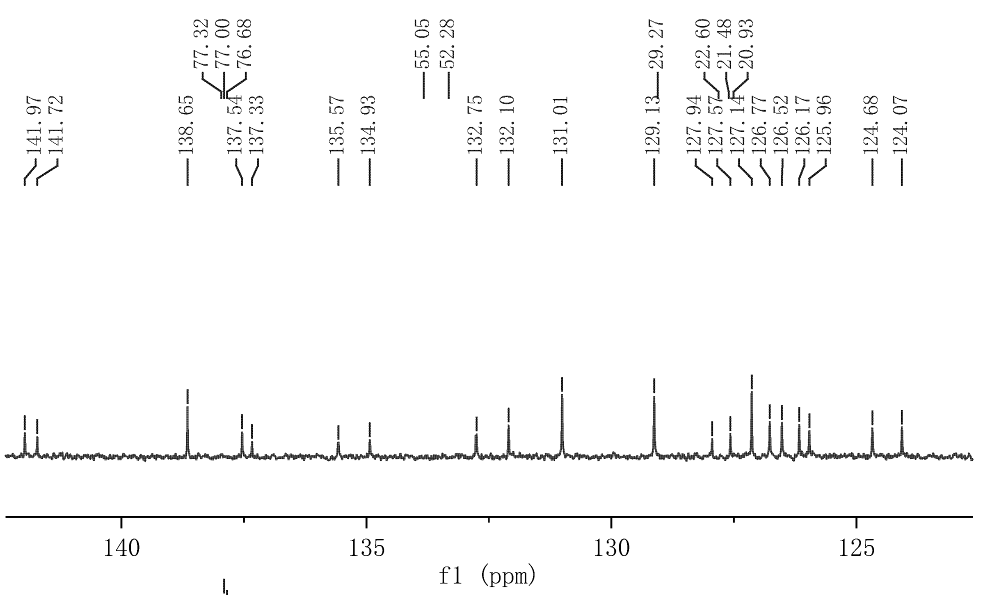

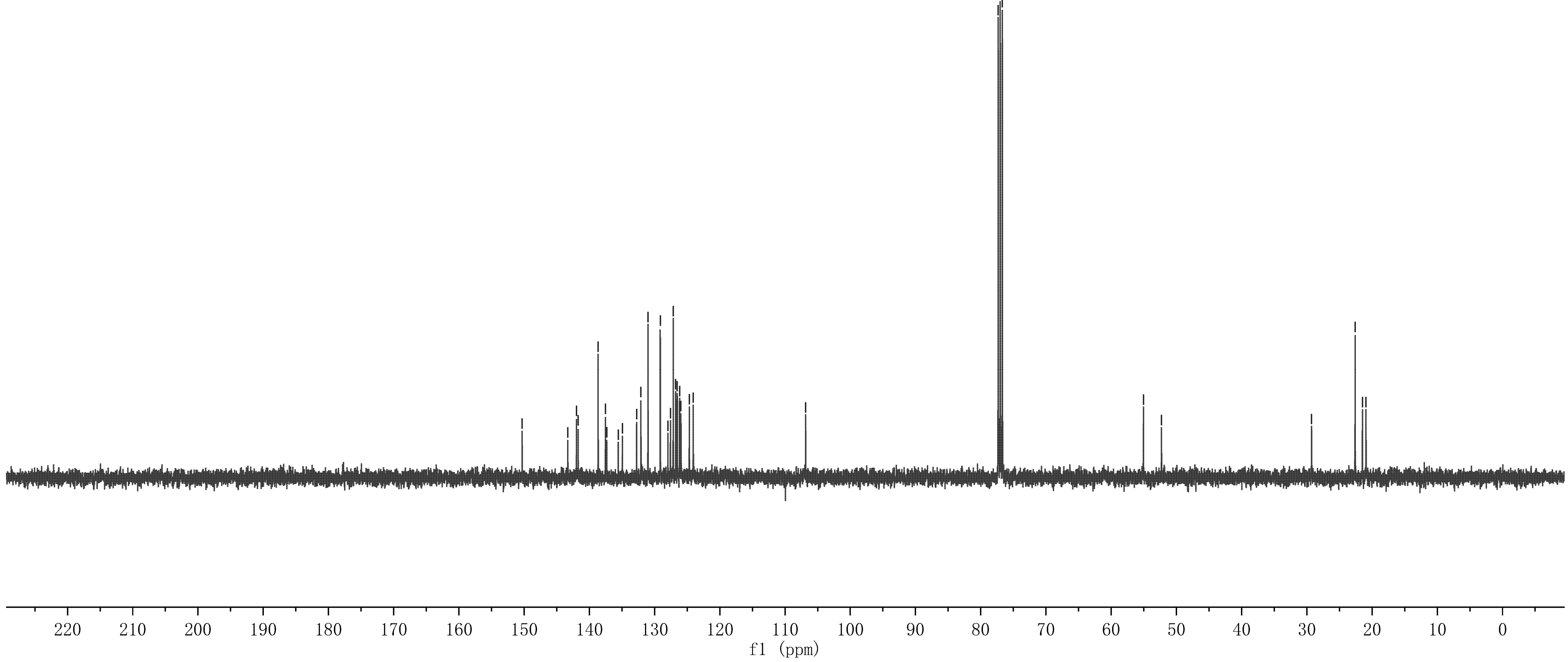

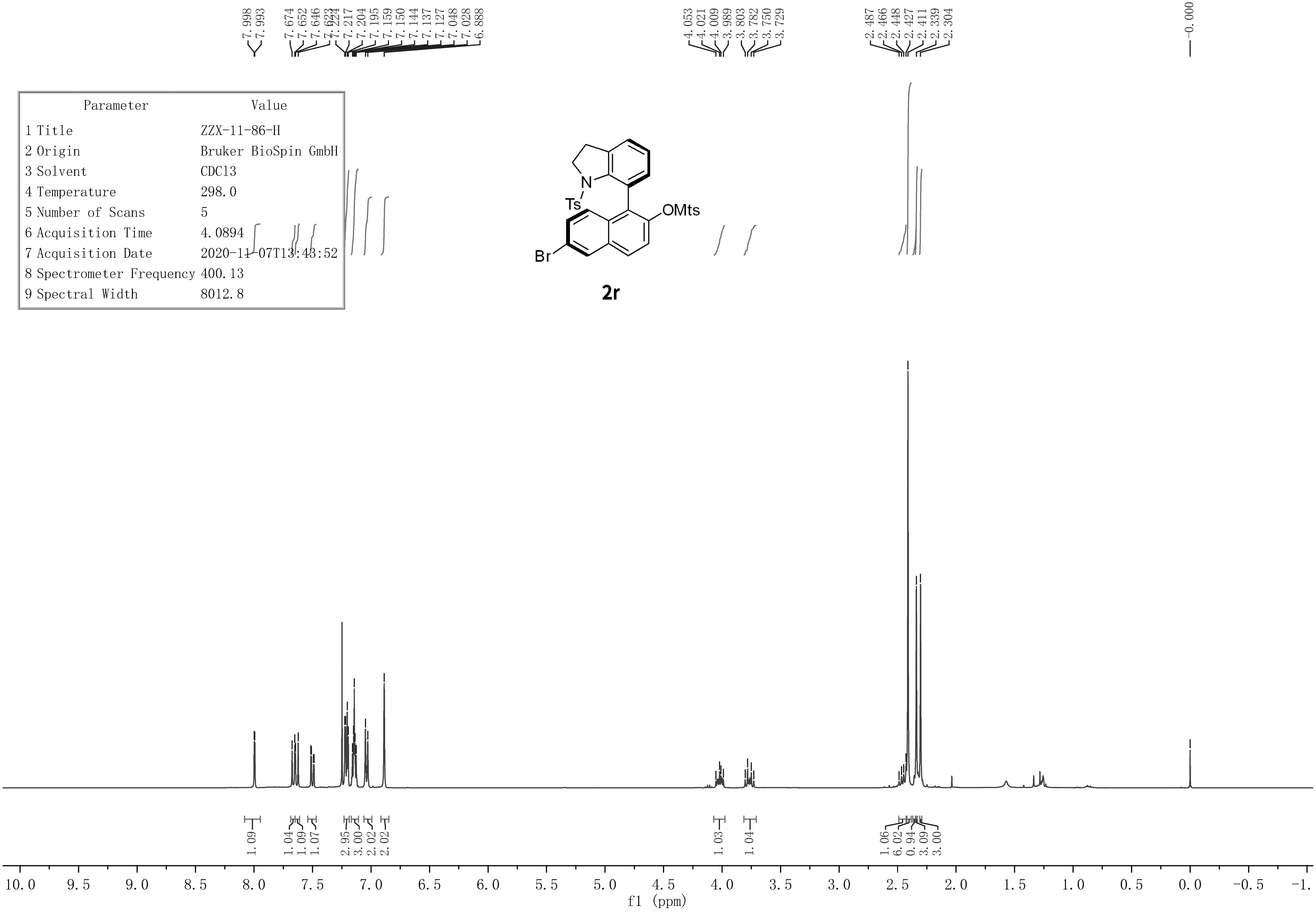

Parameter	Value
1 Title	ZZX-11-86-H
2 Origin	Bruker BioSpin GmbH
3 Solvent	CDCl3
4 Temperature	298.0
5 Number of Scans	5
6 Acquisition Time	4.0894
7 Acquisition Date	2020-11-07T13:43:52
8 Spectrometer Frequency	400.13
9 Spectral Width	8012.8

Parameter	Value
1 Title	ZZX-11-86-C
2 Origin	Bruker BioSpin GmbH
3 Solvent	CDCl3
4 Temperature	300.0
5 Number of Scans	25
6 Acquisition Time	1.3631
7 Acquisition Date	2020-11-07T13:45:24
8 Spectrometer Frequency	100.61
9 Spectral Width	24038.5

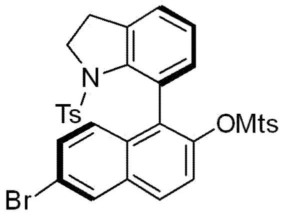

2r

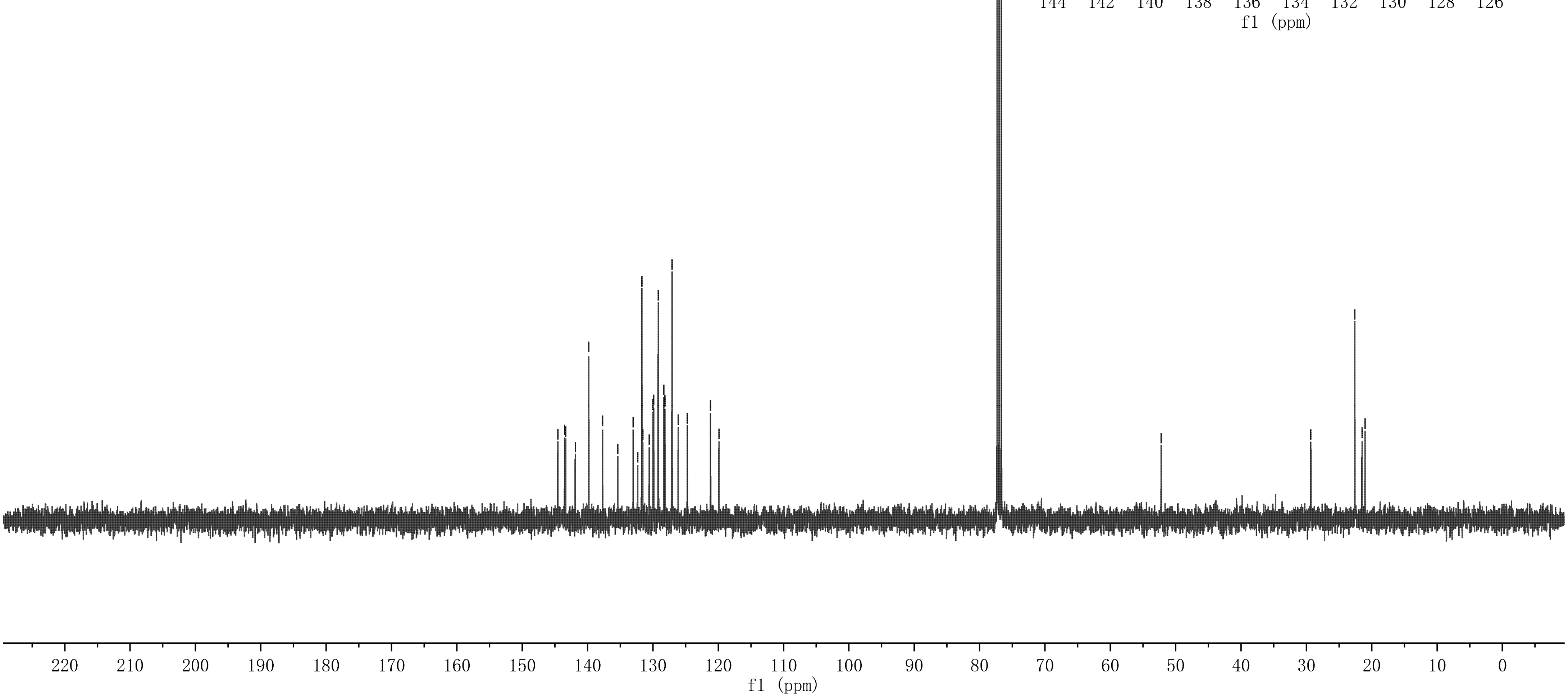

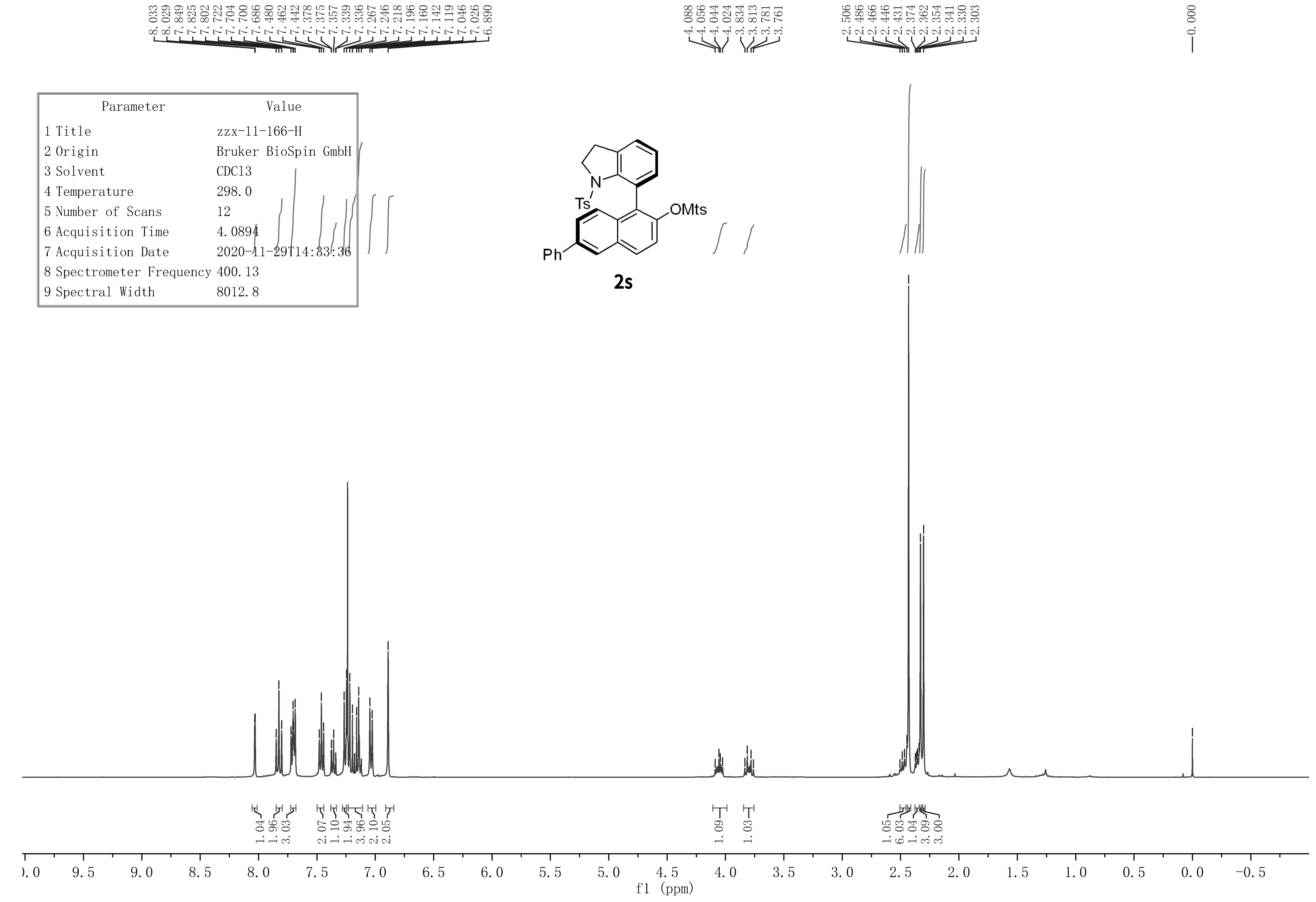
8.033
8.029
7.849
7.825
7.802
7.722
7.704
7.700
7.686
7.480
7.462
7.442
7.378
7.375
7.357
7.339
7.336
7.267
7.246
7.218
7.196
7.160
7.142
7.119
7.046
7.026
6.890
4.088
4.056
4.044
4.024
3.834
3.813
3.781
3.761
2.506
2.486
2.466
2.446
2.431
2.374
2.362
2.354
2.341
2.330
2.303
0.000
Parameter Value
1 Title zzx-11-166-H
2 Origin Bruker BioSpin GmbH
3 Solvent CDCl3
4 Temperature 298.0
5 Number of Scans 12
6 Acquisition Time 4.0894
7 Acquisition Date 2020-11-29T14:33:36
8 Spectrometer Frequency 400.13
9 Spectral Width 8012.8
N
Ts
OMts
Ph
2s
1.04
1.96
3.03
2.07
1.10
1.94
3.96
2.10
2.05
1.09
1.03
1.05
6.03
1.04
3.09
3.00
9.5
9.0
8.5
8.0
7.5
7.0
6.5
6.0
5.5
5.0
4.5
4.0
3.5
3.0
2.5
2.0
1.5
1.0
0.5
0.0
-0.5
f1 (ppm)

Parameter	Value
1 Title	zzx-11-166-C-1
2 Origin	Bruker BioSpin GmbH
3 Solvent	CDCl3
4 Temperature	300.0
5 Number of Scans	64
6 Acquisition Time	1.3631
7 Acquisition Date	2020-11-29T14:37:15
8 Spectrometer Frequency	100.61
9 Spectral Width	24038.5

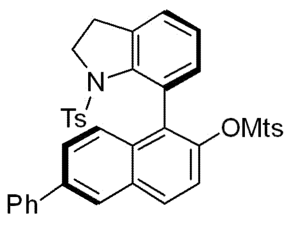

2s

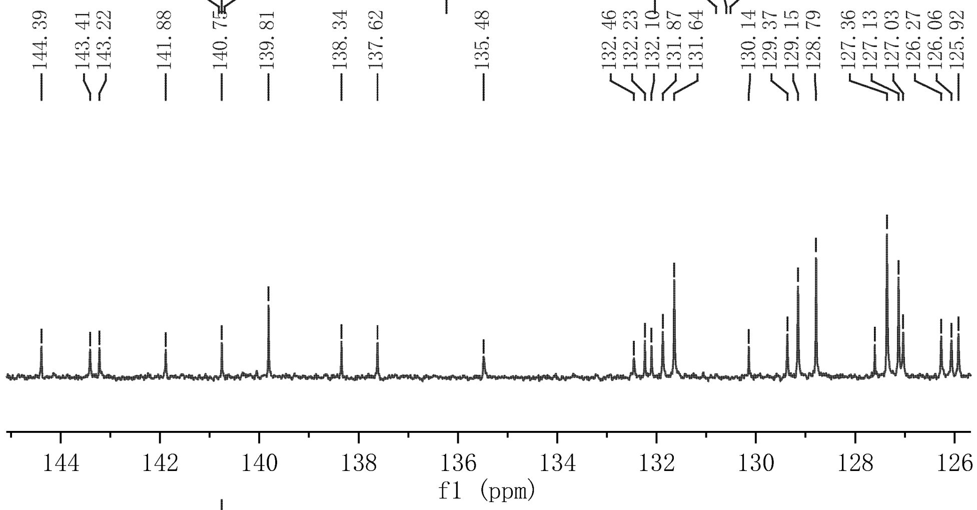

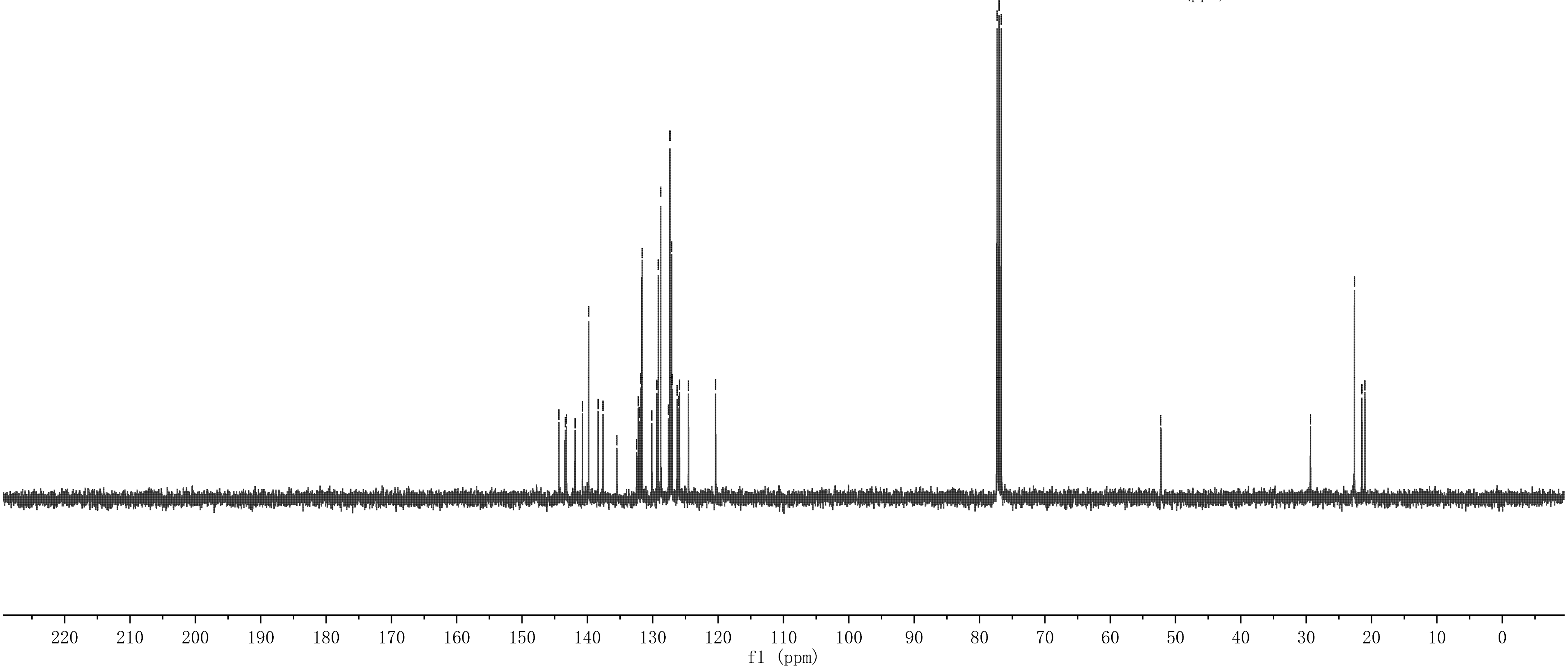

Parameter	Value
1 Title	zzx-11-198-H
2 Origin	Bruker BioSpin GmbH
3 Solvent	CDCl3
4 Temperature	298.4
5 Number of Scans	11
6 Acquisition Time	3.9846
7 Acquisition Date	2020-12-22T15:35:22
8 Spectrometer Frequency	400.03
9 Spectral Width	8223.7

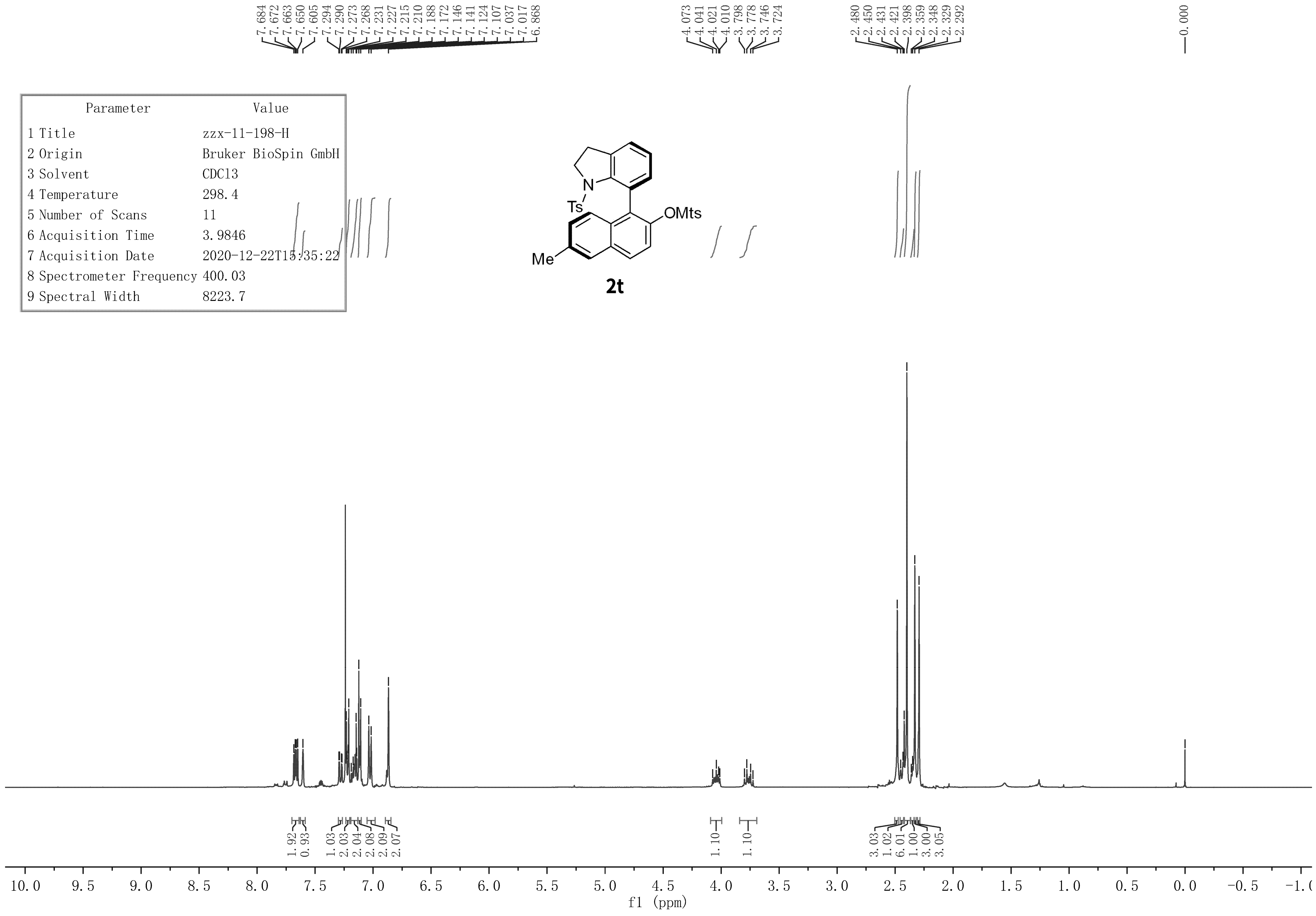

2t

Parameter	Value
1 Title	zzx-11-198-C
2 Origin	Bruker BioSpin GmbH
3 Solvent	CDCl3
4 Temperature	299.0
5 Number of Scans	59
6 Acquisition Time	1.3631
7 Acquisition Date	2020-12-22T15:38:43
8 Spectrometer Frequency	100.59
9 Spectral Width	24038.5

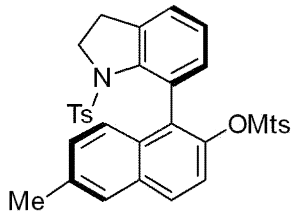

2t

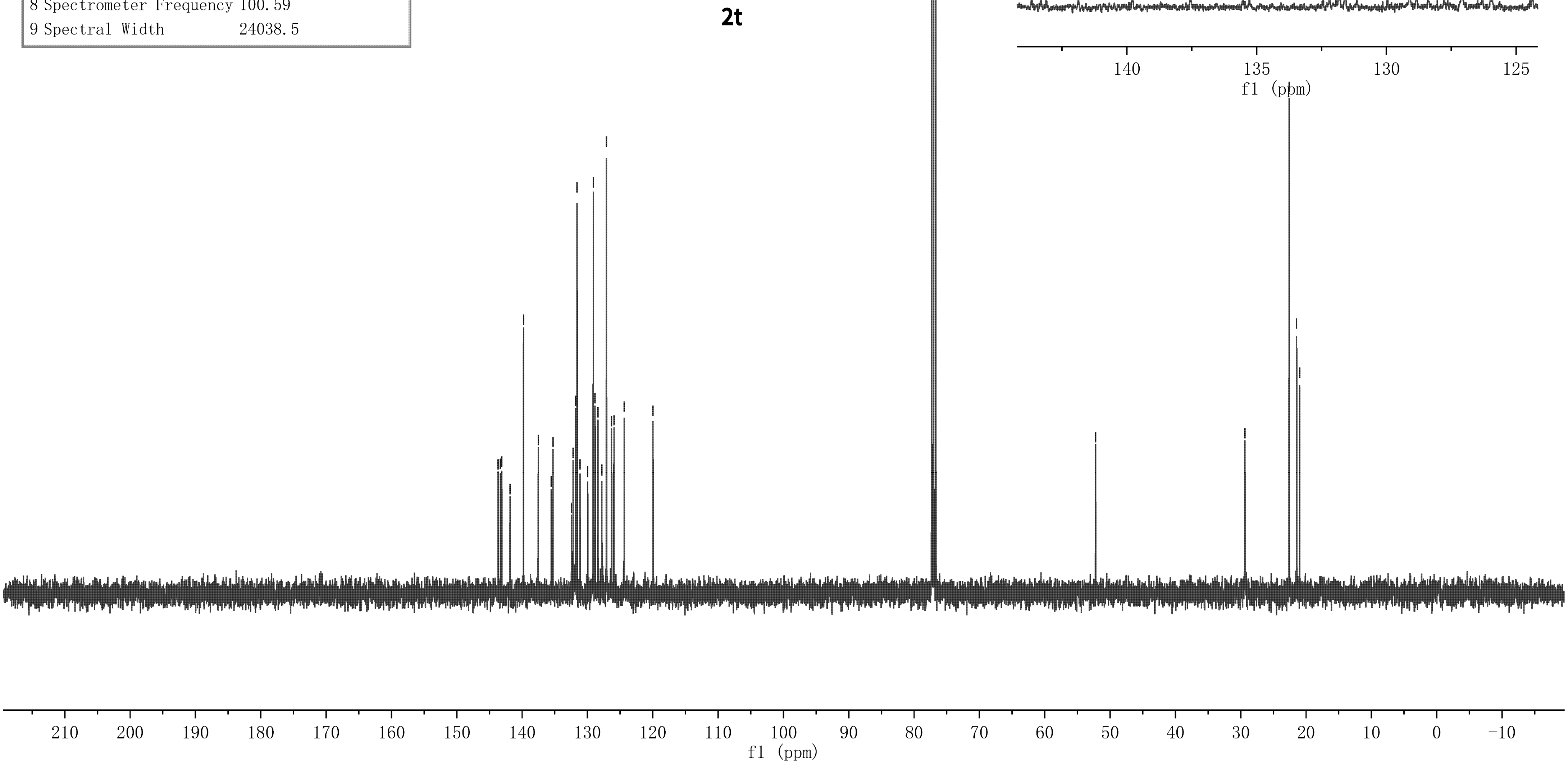

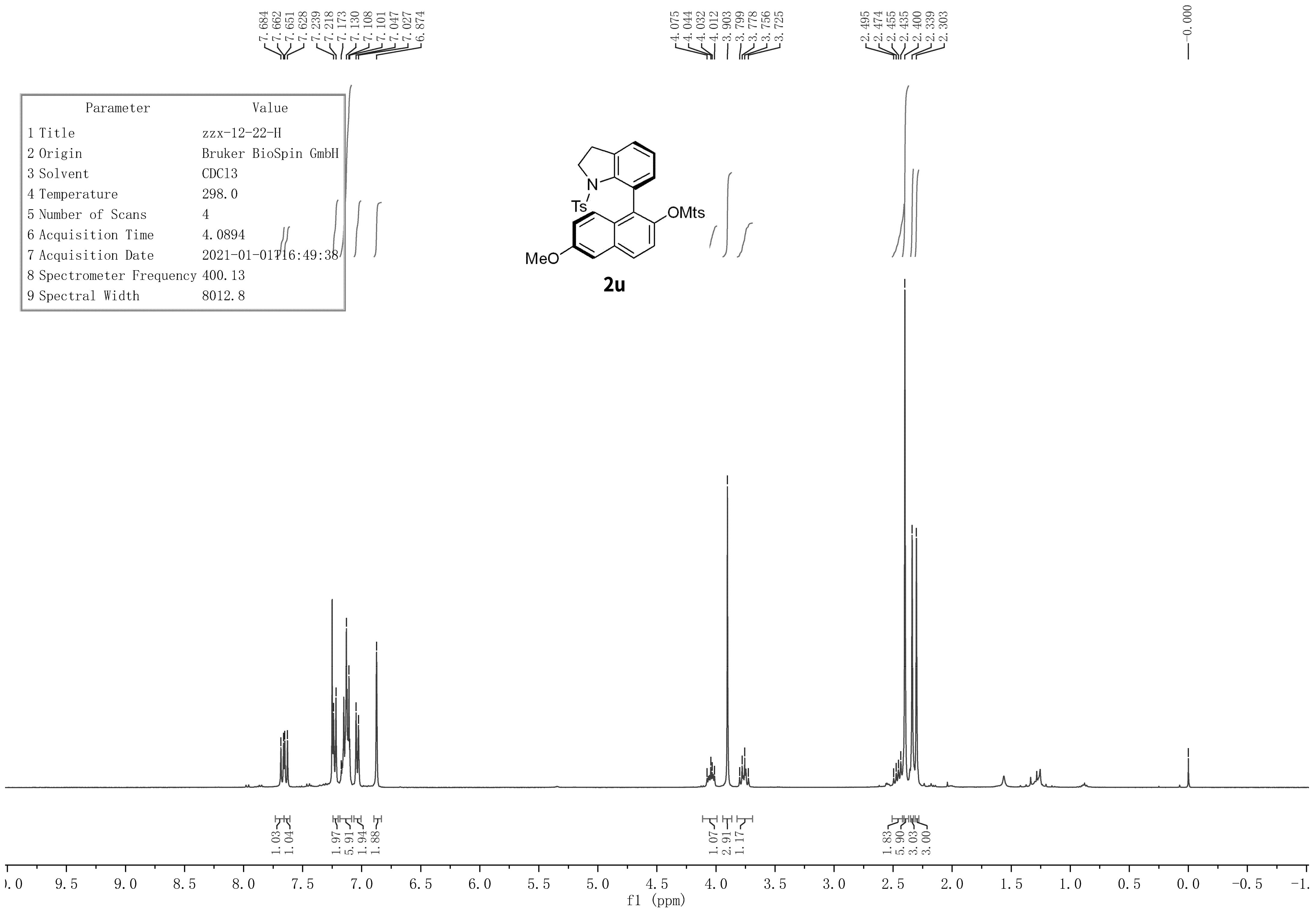

Parameter Value
1 Title zzx-12-22-H
2 Origin Bruker BioSpin GmbH
3 Solvent CDCl3
4 Temperature 298.0
5 Number of Scans 4
6 Acquisition Time 4.0894
7 Acquisition Date 2021-01-01T16:49:38
8 Spectrometer Frequency 400.13
9 Spectral Width 8012.8
N
Ts
OMts
MeO
2u
7.684 7.662 7.651 7.628 7.239 7.218 7.173 7.130 7.108 7.101 7.047 7.027 6.874
4.075 4.044 4.032 4.012 3.903 3.799 3.778 3.756 3.725
2.495 2.474 2.455 2.435 2.400 2.339 2.303
0.000
1.03 1.04 1.97 5.91 1.94 1.88
1.07 2.91 1.17
1.83 5.90 3.03 3.00
9.5 9.0 8.5 8.0 7.5 7.0 6.5 6.0 5.5 5.0 4.5 4.0 3.5 3.0 2.5 2.0 1.5 1.0 0.5 0.0 -0.5
f1 (ppm)

Parameter	Value
1 Title	zzx-12-22-C
2 Origin	Bruker BioSpin GmbH
3 Solvent	CDCl3
4 Temperature	300.0
5 Number of Scans	136
6 Acquisition Time	1.3631
7 Acquisition Date	2021-01-01T16:51:15
8 Spectrometer Frequency	100.61
9 Spectral Width	24038.5

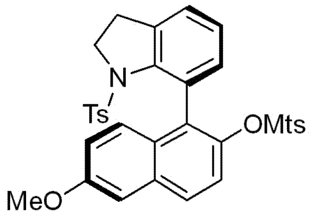

2u

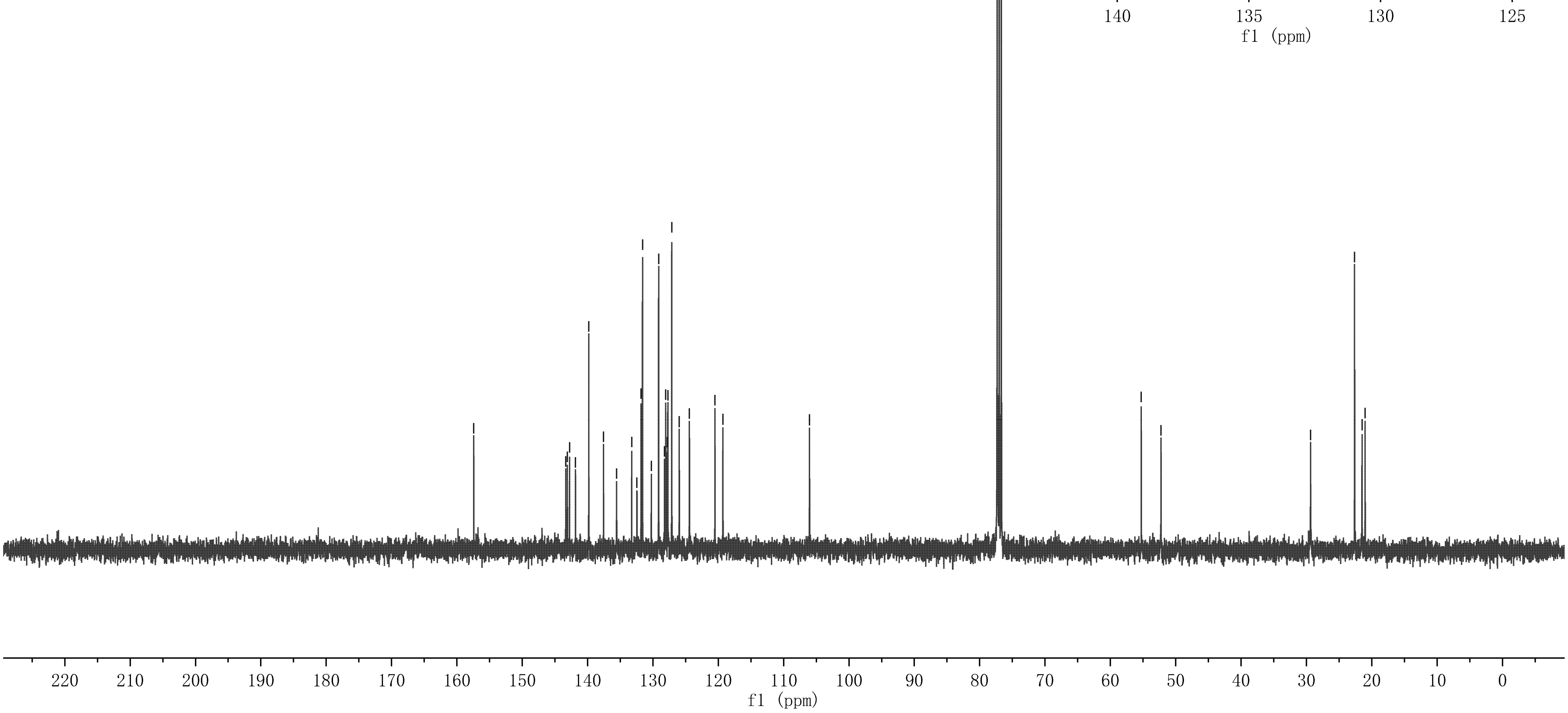

Parameter	Value
1 Title	zzx-11-121-H
2 Origin	Bruker BioSpin GmbH
3 Solvent	CDCl3
4 Temperature	298.0
5 Number of Scans	7
6 Acquisition Time	4.0894
7 Acquisition Date	2020-11-14T15:20:35
8 Spectrometer Frequency	400.13
9 Spectral Width	8012.8

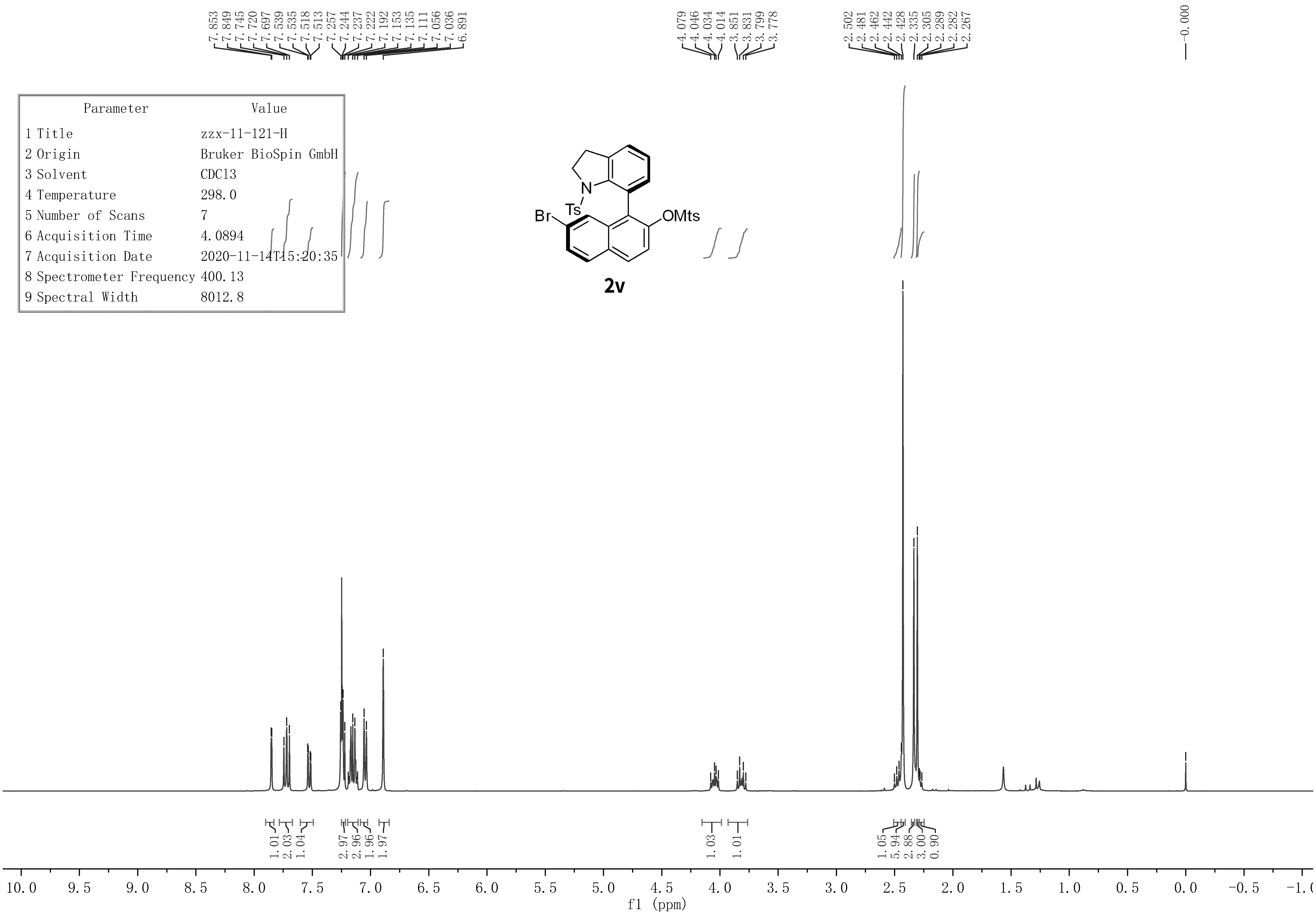

Parameter	Value
1 Title	zzx-11-121-C
2 Origin	Bruker BioSpin GmbH
3 Solvent	CDCl3
4 Temperature	300.0
5 Number of Scans	41
6 Acquisition Time	1.3631
7 Acquisition Date	2020-11-14T15:22:06
8 Spectrometer Frequency	100.61
9 Spectral Width	24038.5

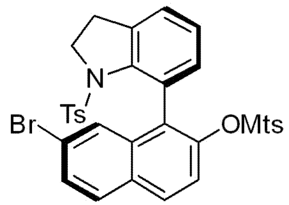

2v

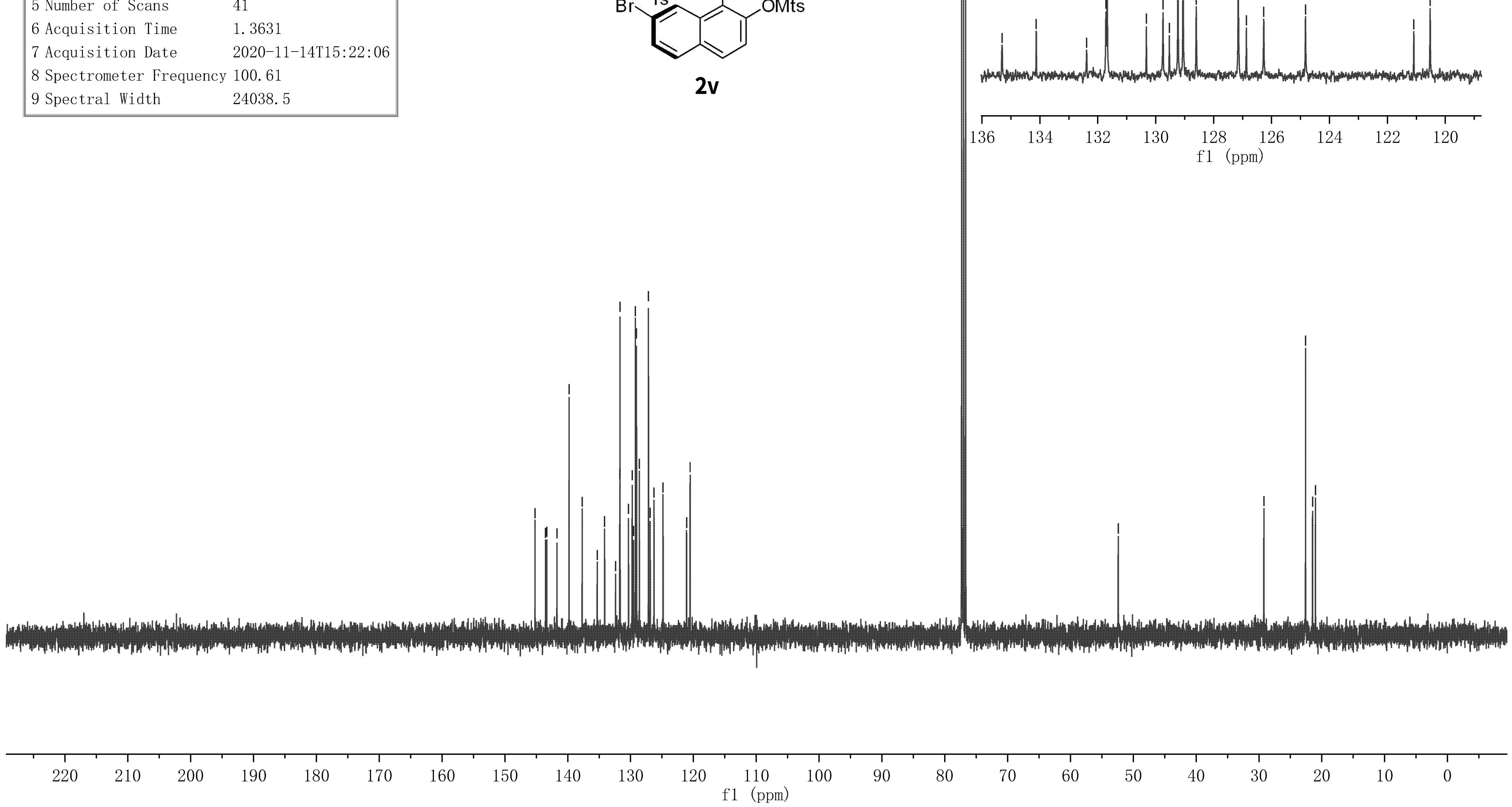

Parameter	Value
1 Title	zzx-11-167-H
2 Origin	Bruker BioSpin GmbH
3 Solvent	CDCl3
4 Temperature	298.0
5 Number of Scans	8
6 Acquisition Time	4.0894
7 Acquisition Date	2020-11-29T14:19:41
8 Spectrometer Frequency	400.13
9 Spectral Width	8012.8

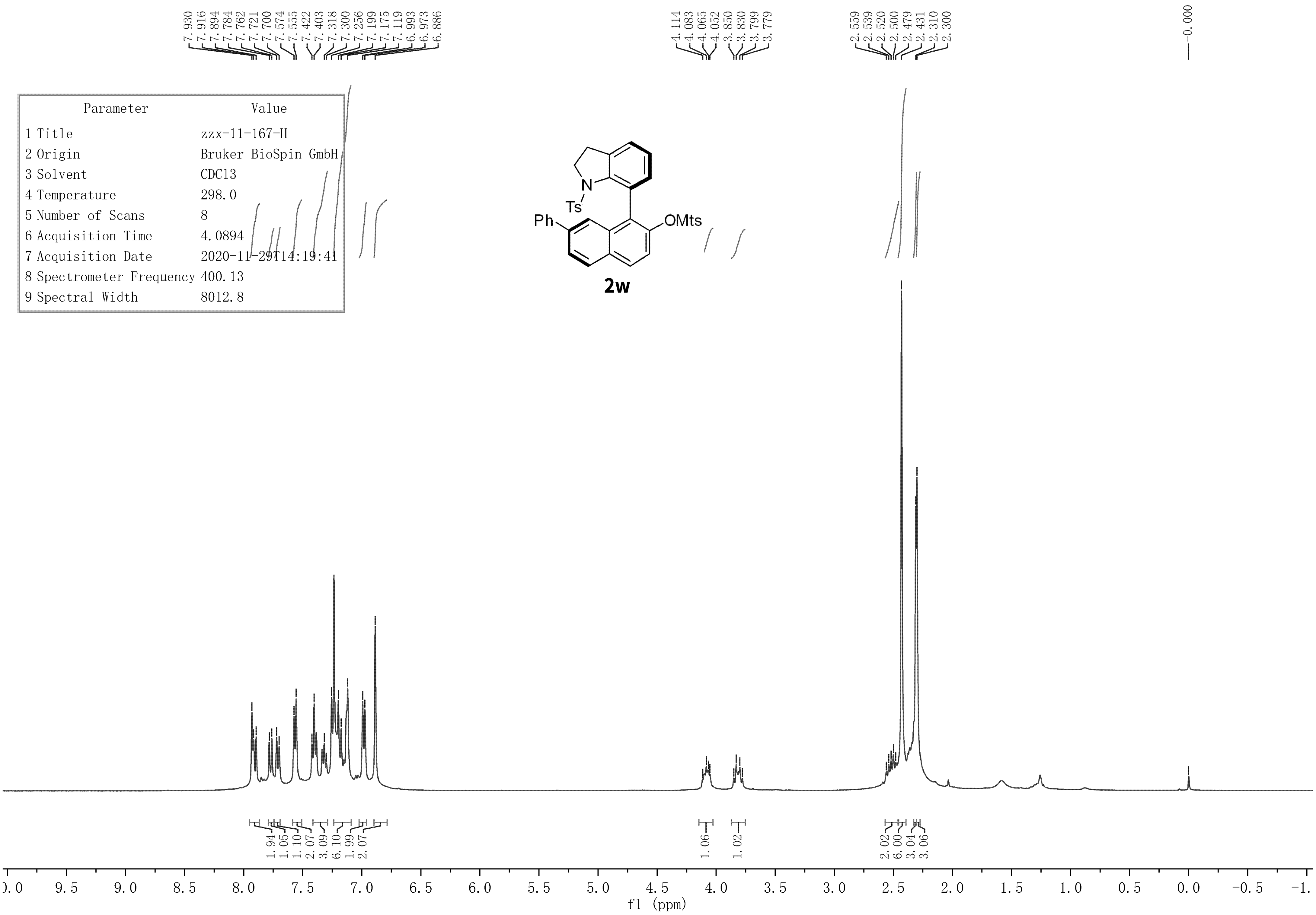

Parameter	Value
1 Title	zzx-11-167-C
2 Origin	Bruker BioSpin GmbH
3 Solvent	CDCl3
4 Temperature	300.0
5 Number of Scans	129
6 Acquisition Time	1.3631
7 Acquisition Date	2020-11-29T14:22:25
8 Spectrometer Frequency	100.61
9 Spectral Width	24038.5

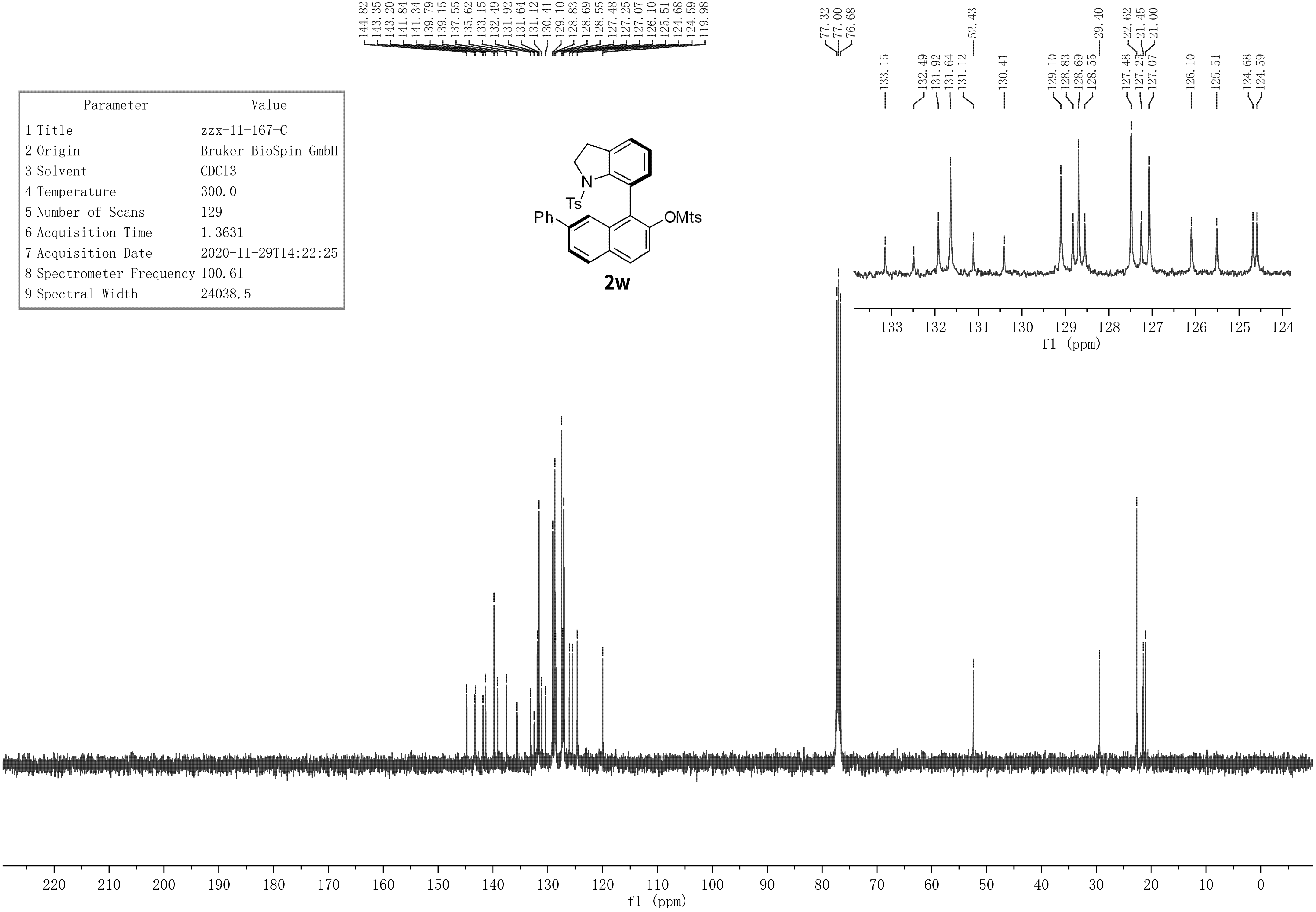

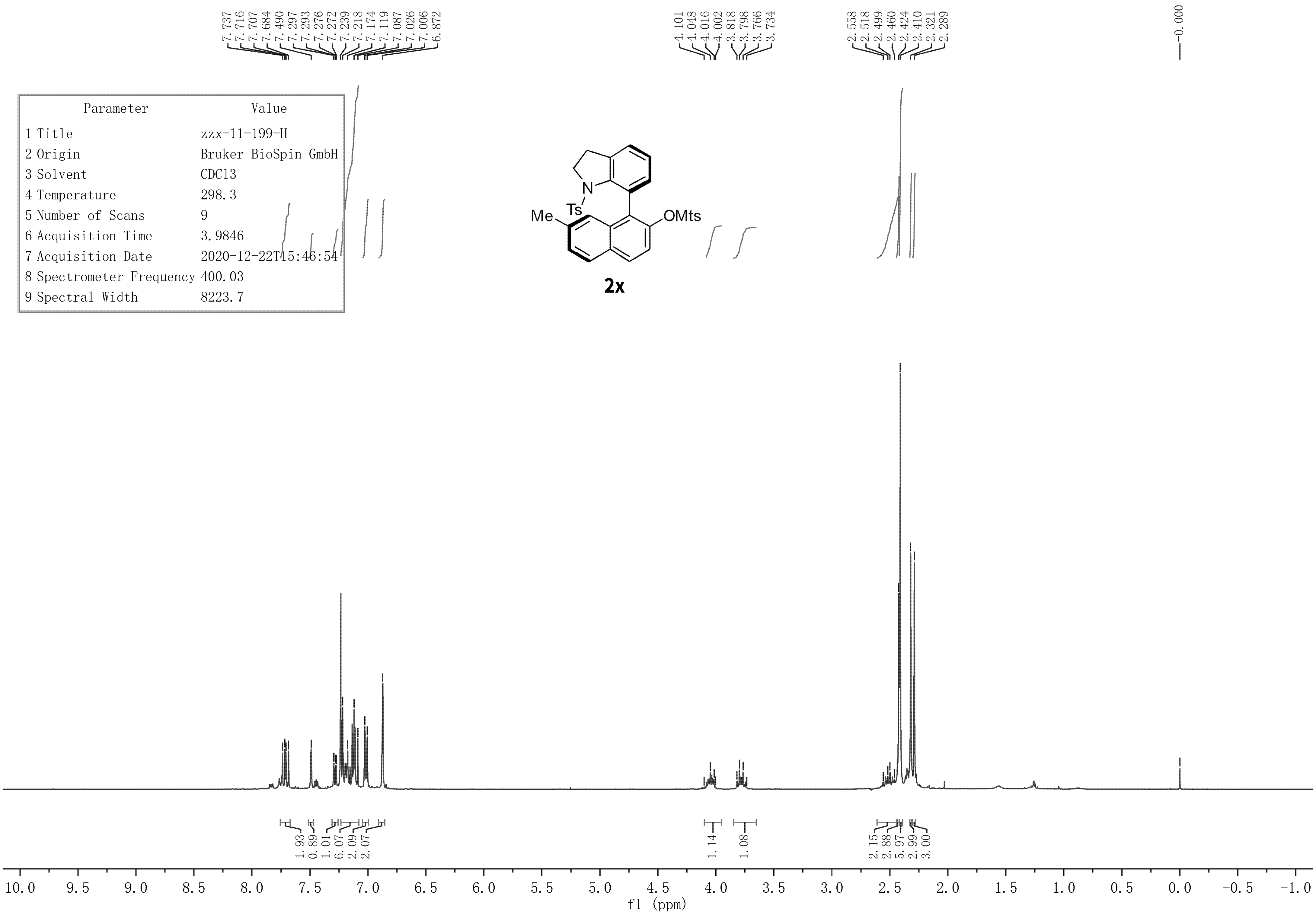

7.737 7.716 7.707 7.684 7.490 7.297 7.293 7.276 7.272 7.239 7.218 7.174 7.119 7.087 7.026 7.006 6.872
4.101 4.048 4.016 4.002 3.818 3.798 3.766 3.734
2.558 2.518 2.499 2.460 2.424 2.410 2.321 2.289
-0.000
Parameter Value
1 Title zzx-11-199-H
2 Origin Bruker BioSpin GmbH
3 Solvent CDCl3
4 Temperature 298.3
5 Number of Scans 9
6 Acquisition Time 3.9846
7 Acquisition Date 2020-12-22T15:46:54
8 Spectrometer Frequency 400.03
9 Spectral Width 8223.7
N
Ts
Me
OMts
2x
1.93 0.89 1.01 6.07 2.09 2.07
1.14
1.08
2.15 2.88 5.97 2.99 3.00
10.0 9.5 9.0 8.5 8.0 7.5 7.0 6.5 6.0 5.5 5.0 4.5 4.0 3.5 3.0 2.5 2.0 1.5 1.0 0.5 0.0 -0.5 -1.0
f1 (ppm)

Parameter	Value
1 Title	zzx-11-199-C
2 Origin	Bruker BioSpin GmbH
3 Solvent	CDCl3
4 Temperature	298.7
5 Number of Scans	76
6 Acquisition Time	1.3631
7 Acquisition Date	2020-12-22T15:49:11
8 Spectrometer Frequency	100.59
9 Spectral Width	24038.5

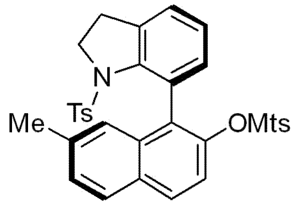

2x

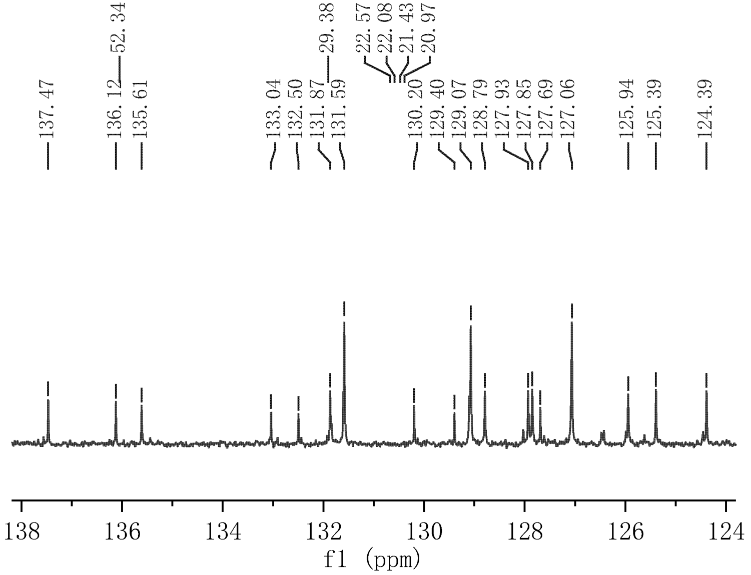

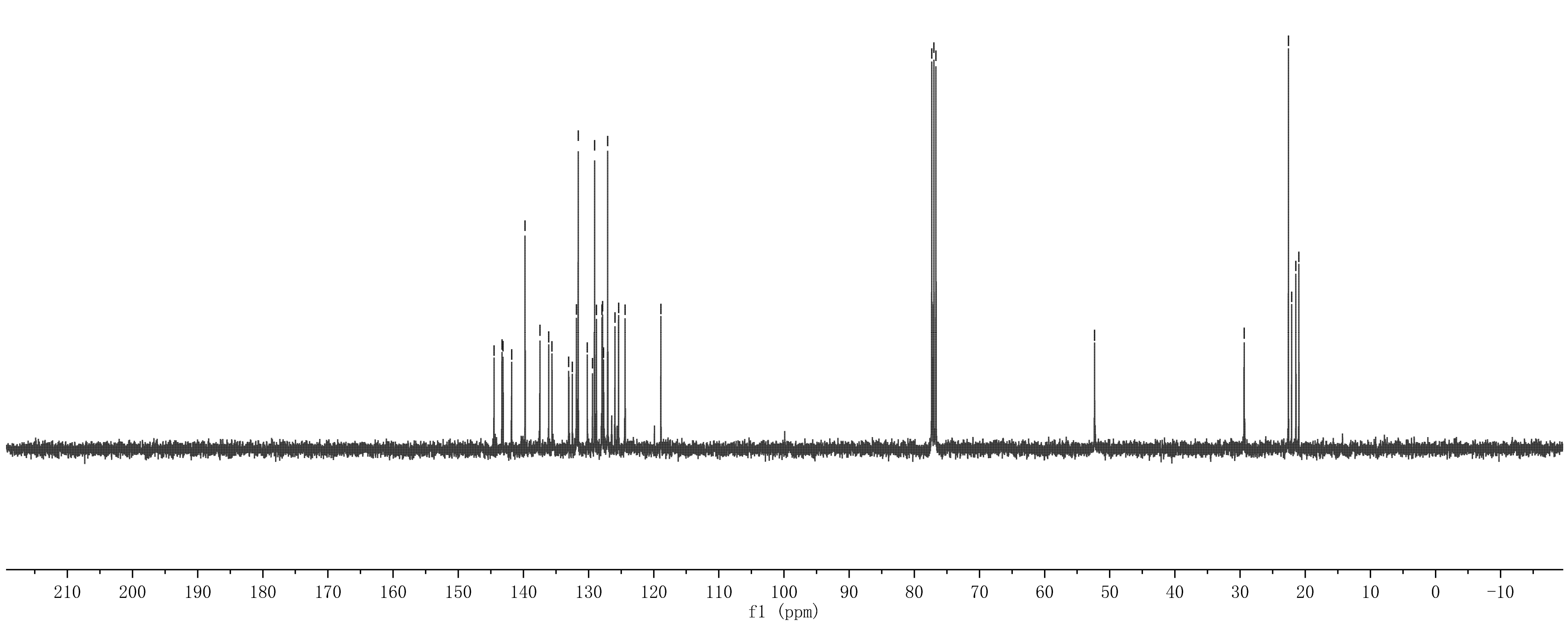

Parameter	Value
1 Title	zzx-12-5-H
2 Origin	Bruker BioSpin GmbH
3 Solvent	CDCl3
4 Temperature	298.0
5 Number of Scans	4
6 Acquisition Time	4.0894
7 Acquisition Date	2020-12-31T09:15:03
8 Spectrometer Frequency	400.13
9 Spectral Width	8012.8

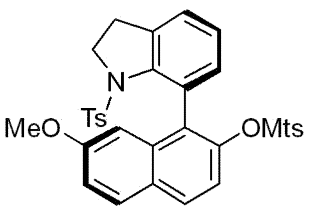

2y

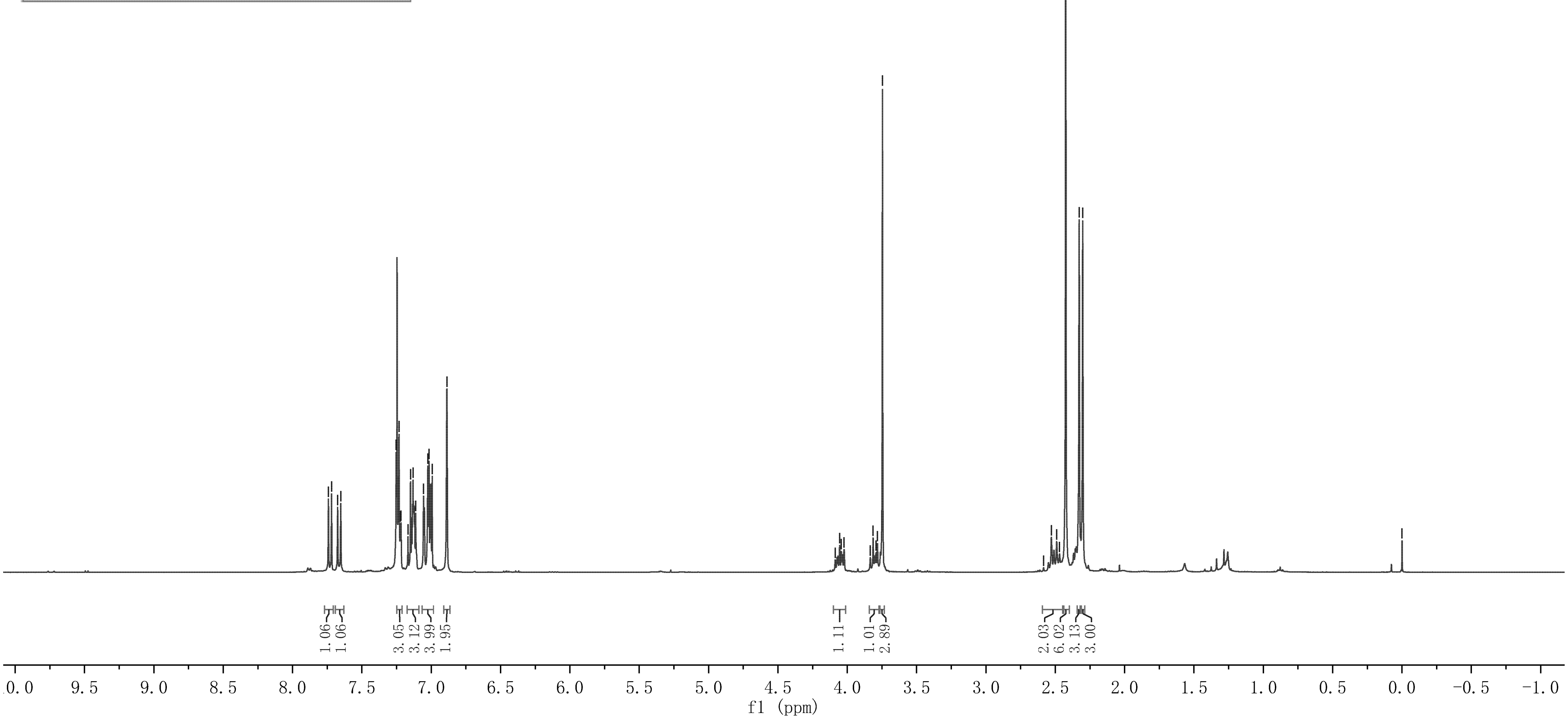

Parameter	Value
1 Title	zzx-12-5-C
2 Origin	Bruker BioSpin GmbH
3 Solvent	CDCl3
4 Temperature	300.0
5 Number of Scans	50
6 Acquisition Time	1.3631
7 Acquisition Date	2020-12-31T09:16:20
8 Spectrometer Frequency	100.61
9 Spectral Width	24038.5

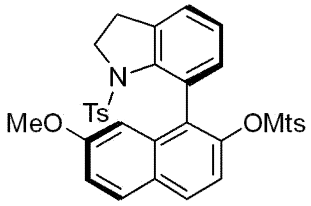

2y

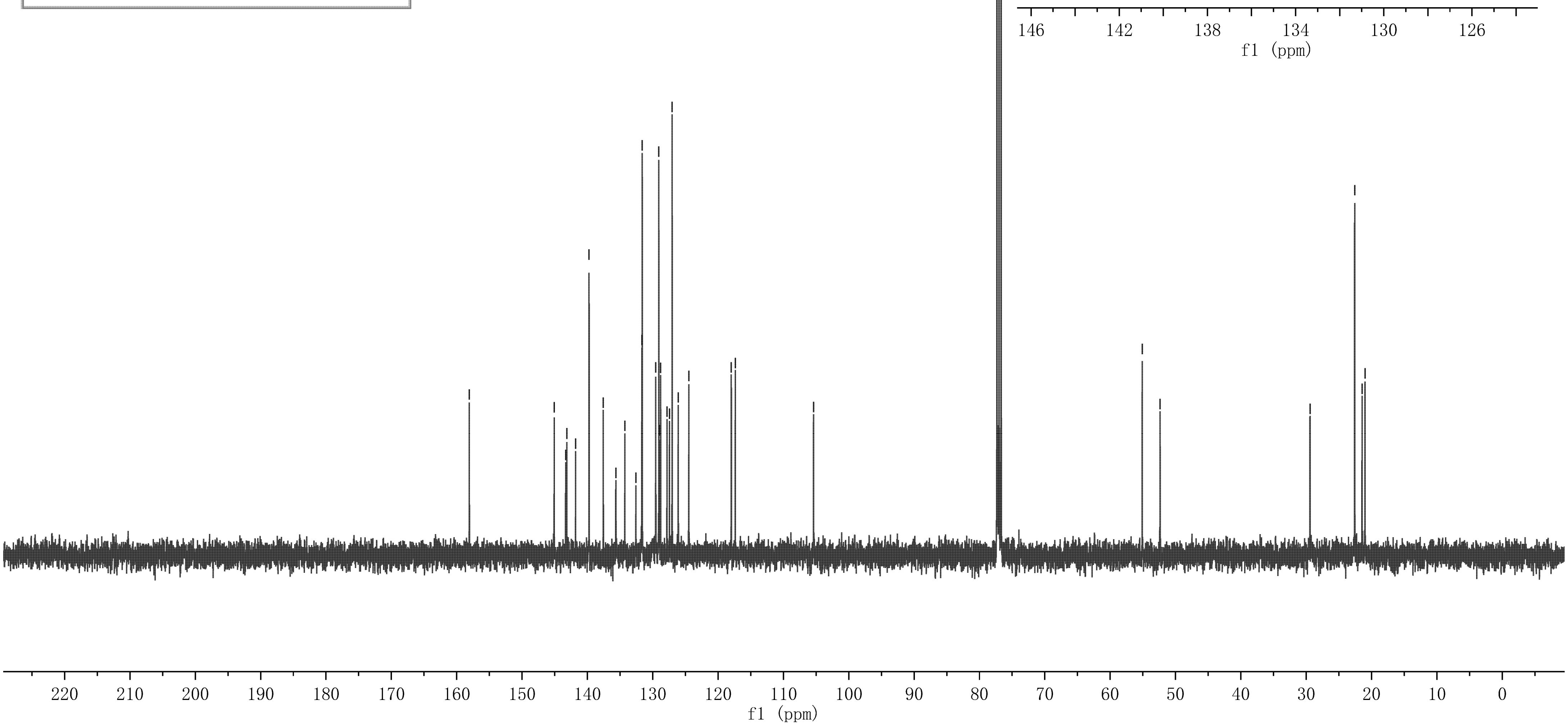

Parameter	Value
1 Title	zzx-12-4-H
2 Origin	Bruker BioSpin GmbH
3 Solvent	CDCl3
4 Temperature	298.0
5 Number of Scans	13
6 Acquisition Time	4.0894
7 Acquisition Date	2020-12-28T10:12:28
8 Spectrometer Frequency	400.13
9 Spectral Width	8012.8

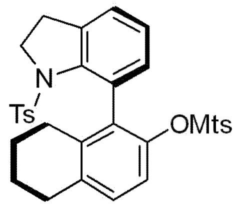

2z

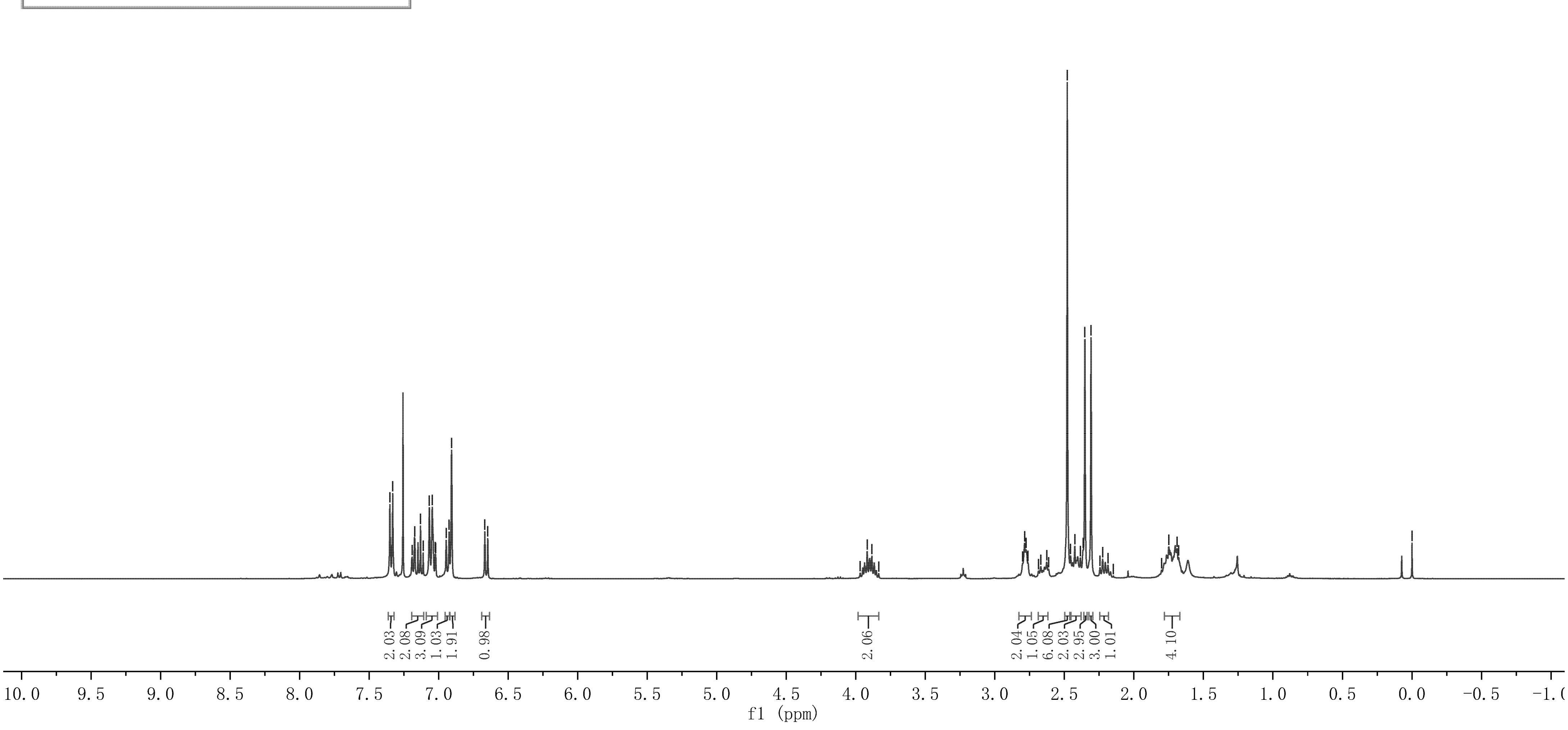

Parameter	Value
1 Title	zzx-12-4-C
2 Origin	Bruker BioSpin GmbH
3 Solvent	CDCl3
4 Temperature	300.0
5 Number of Scans	71
6 Acquisition Time	1.3631
7 Acquisition Date	2020-12-28T10:15:19
8 Spectrometer Frequency	100.61
9 Spectral Width	24038.5

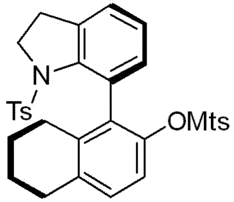

2z

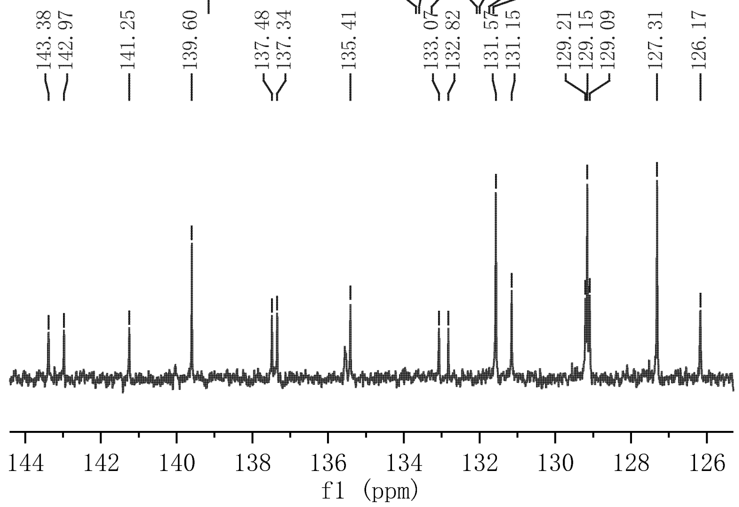

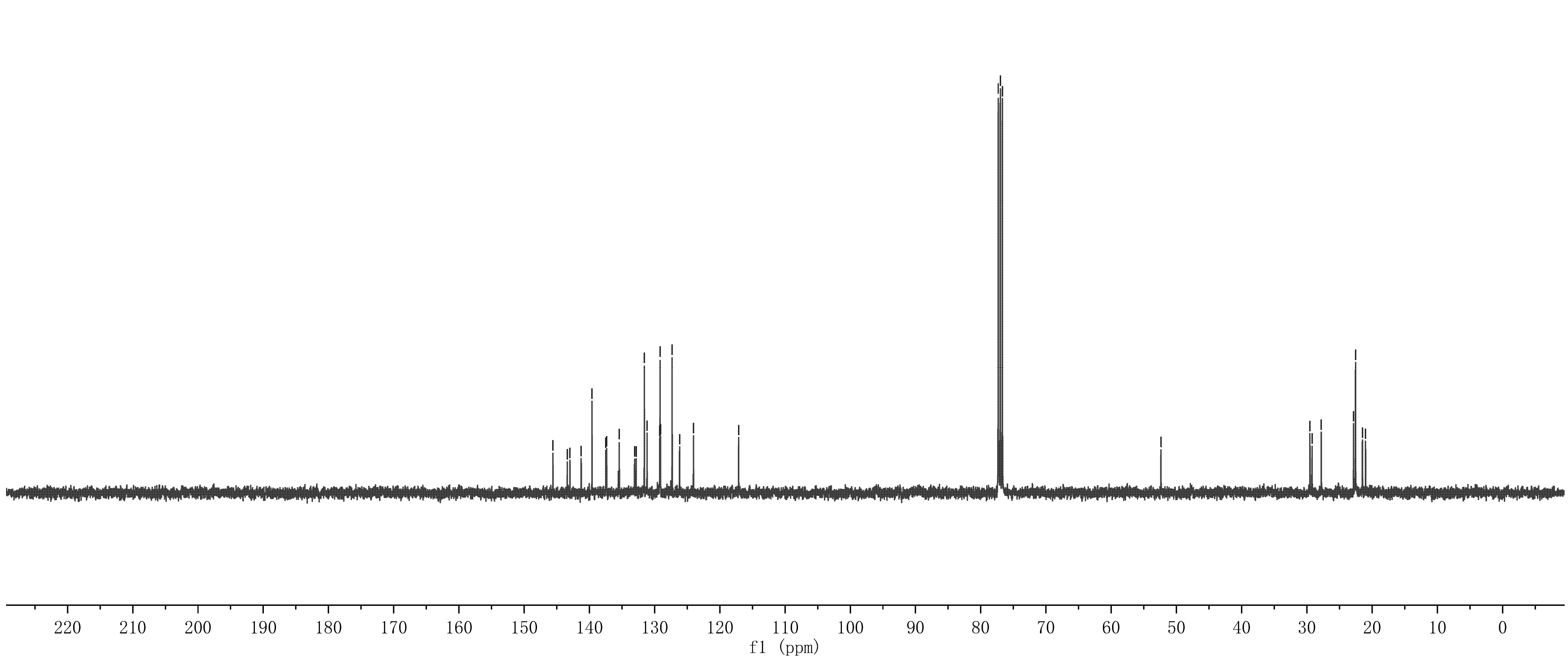

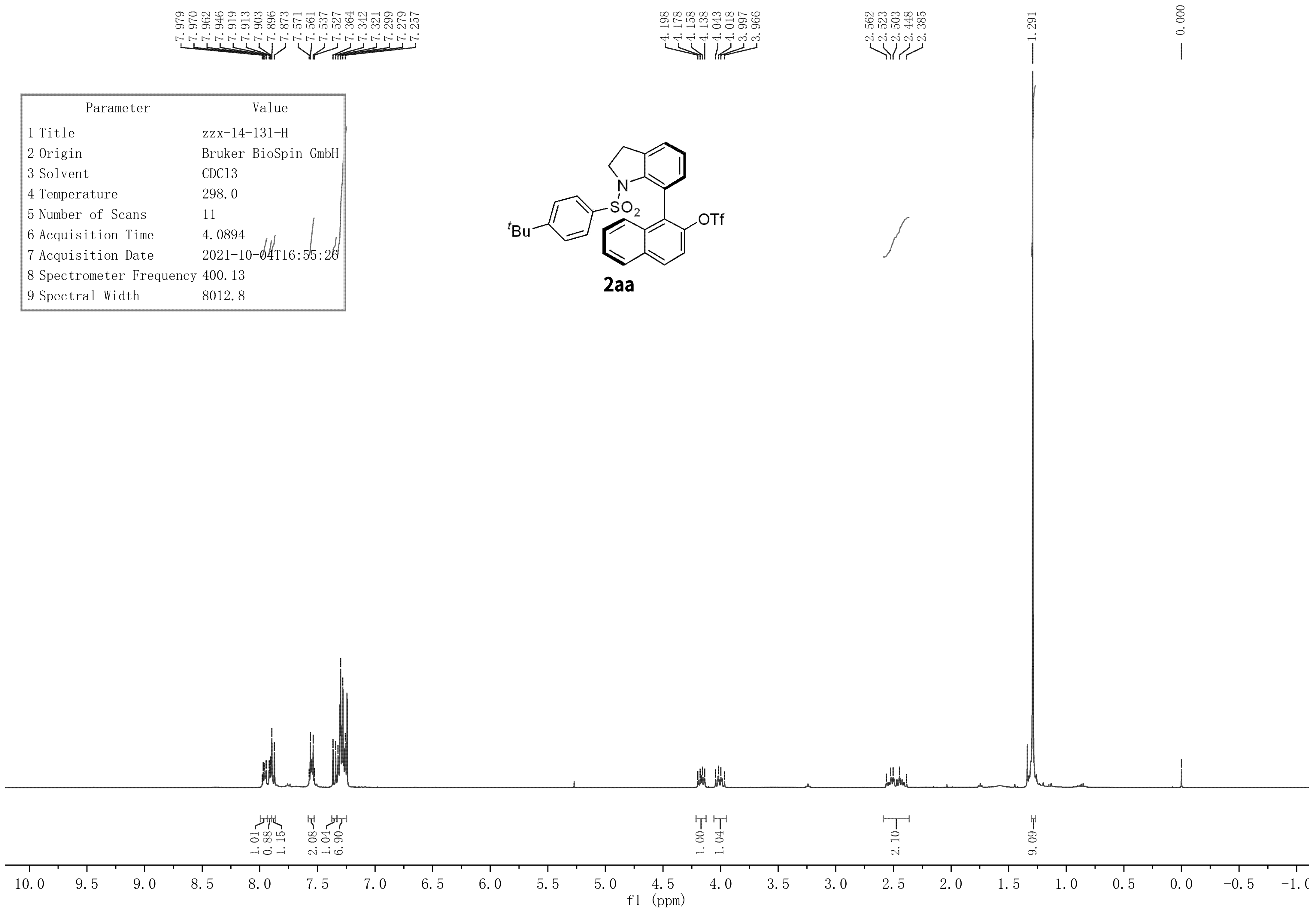

Parameter	Value
1 Title	zzx-14-131-H
2 Origin	Bruker BioSpin GmbH
3 Solvent	CDCl3
4 Temperature	298.0
5 Number of Scans	11
6 Acquisition Time	4.0894
7 Acquisition Date	2021-10-04T16:55:26
8 Spectrometer Frequency	400.13
9 Spectral Width	8012.8

2aa

Parameter	Value
1 Title	zzx-14-131-C
2 Origin	Bruker BioSpin GmbH
3 Solvent	CDCl3
4 Temperature	300.0
5 Number of Scans	41
6 Acquisition Time	1.3631
7 Acquisition Date	2021-10-04T16:56:59
8 Spectrometer Frequency	100.61
9 Spectral Width	24038.5

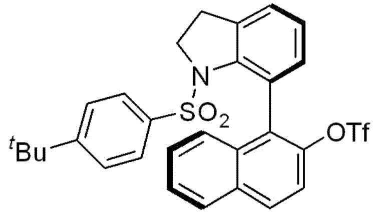

2aa

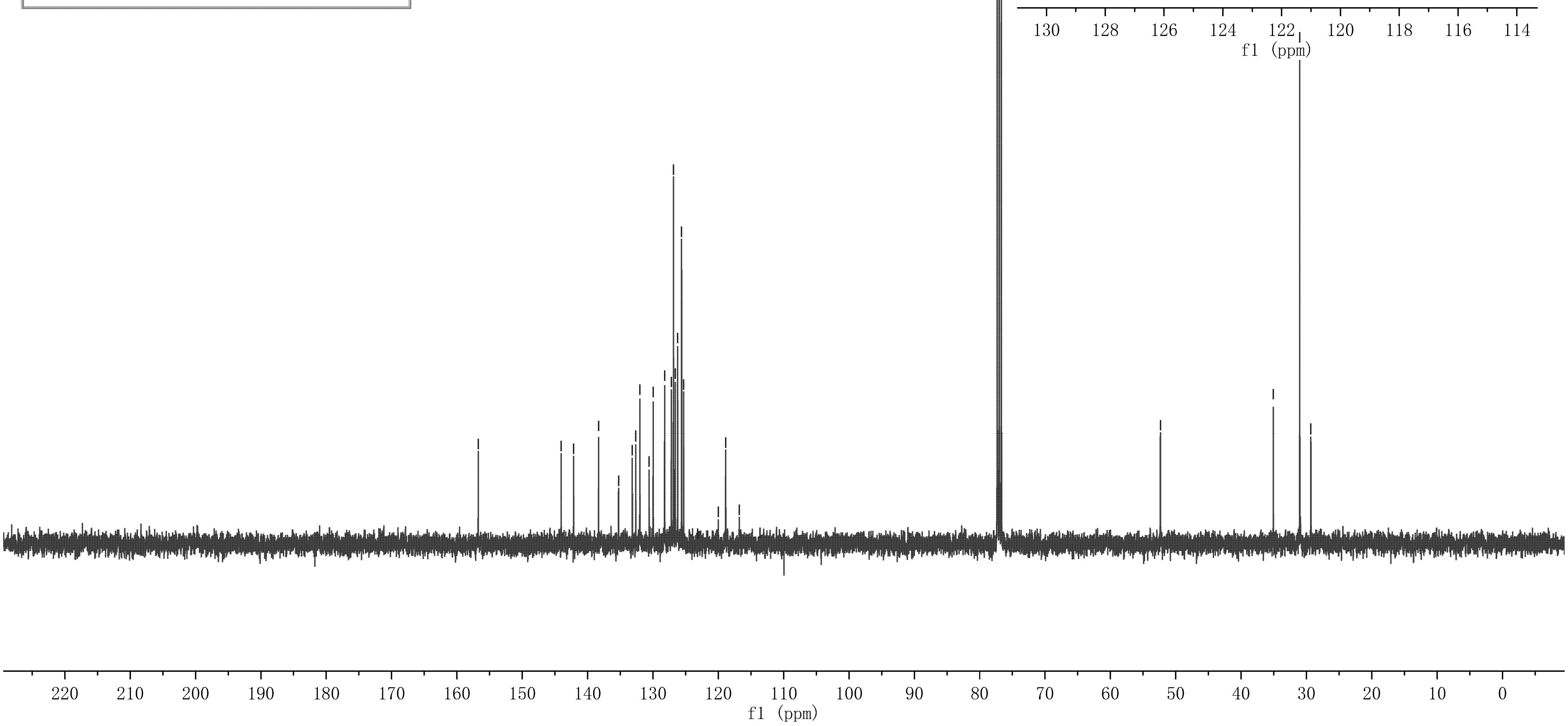

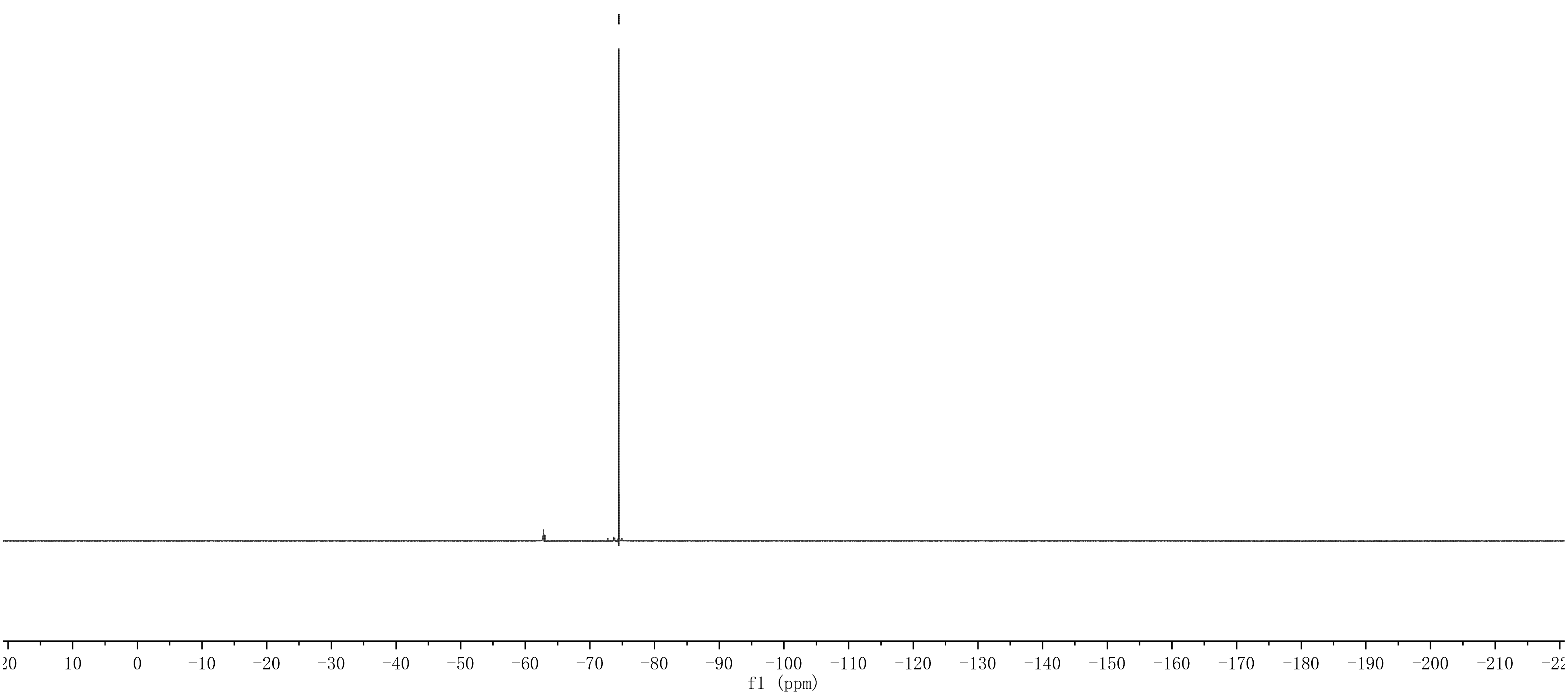

Parameter	Value
1 Title	zzx-14-131-F
2 Origin	Bruker BioSpin GmbH
3 Solvent	CDCl3
4 Temperature	301.2
5 Number of Scans	16
6 Acquisition Time	0.5767
7 Acquisition Date	2021-10-05T15:29:00
8 Spectrometer Frequency	470.63
9 Spectral Width	113636.4

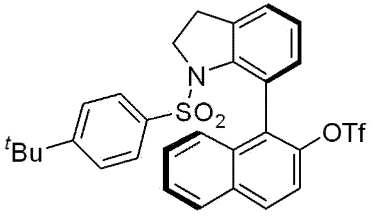

2aa

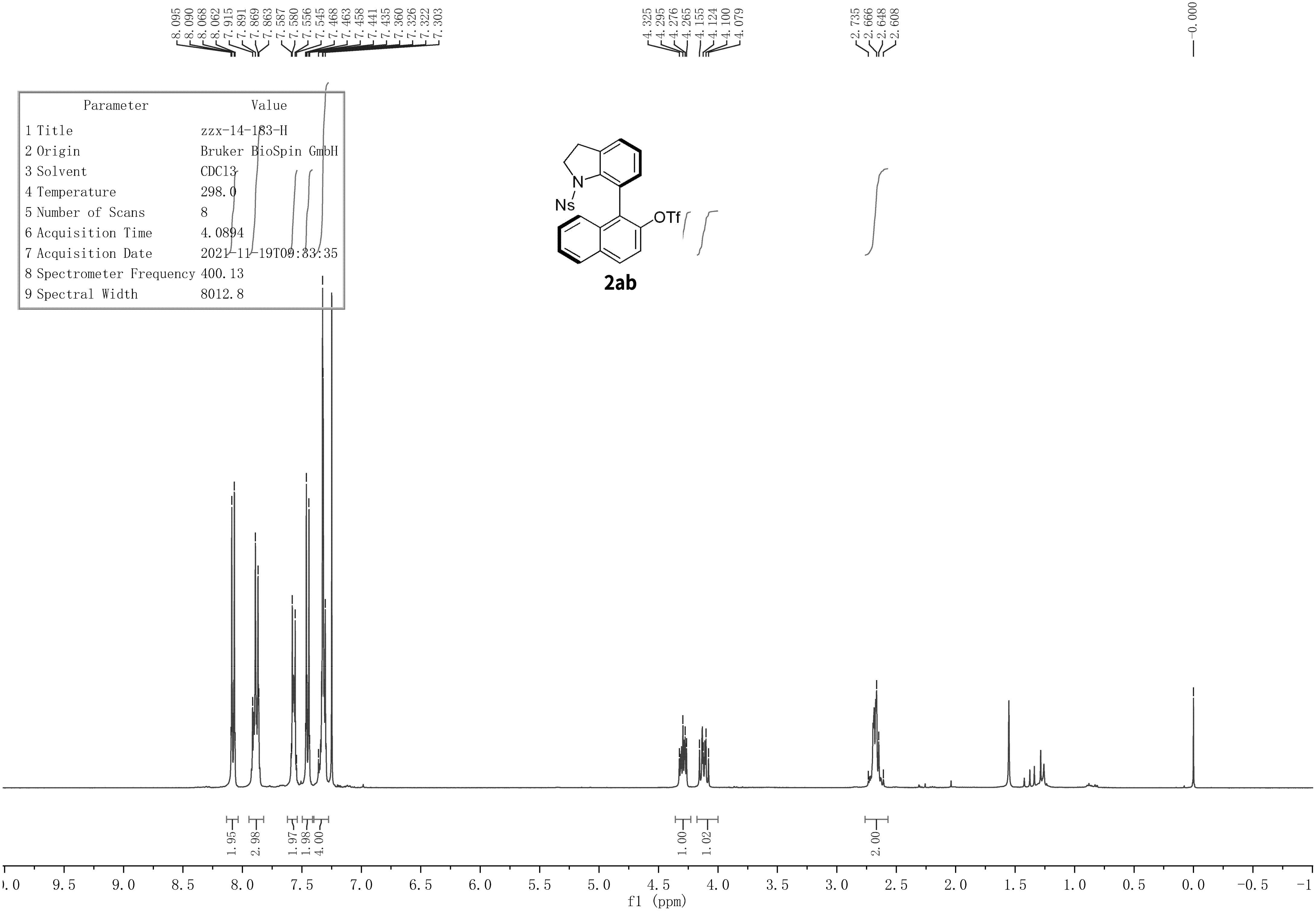
8.095 8.090 8.068 8.062 7.915 7.891 7.869 7.863 7.587 7.580 7.556 7.545 7.468 7.463 7.458 7.441 7.435 7.360 7.326 7.322 7.303
4.325 4.295 4.276 4.265 4.155 4.124 4.100 4.079
2.735 2.666 2.648 2.608
0.000
Parameter Value
1 Title zzx-14-183-H
2 Origin Bruker BioSpin GmbH
3 Solvent CDCl3
4 Temperature 298.0
5 Number of Scans 8
6 Acquisition Time 4.0894
7 Acquisition Date 2021-11-19T09:33:35
8 Spectrometer Frequency 400.13
9 Spectral Width 8012.8
N
Ns
OTf
2ab
1.95 2.98 1.97 1.98 4.00 1.00 1.02 2.00
10.0 9.5 9.0 8.5 8.0 7.5 7.0 6.5 6.0 5.5 5.0 4.5 4.0 3.5 3.0 2.5 2.0 1.5 1.0 0.5 0.0 -0.5 -1
f1 (ppm)

Parameter	Value
1 Title	zzx-14-183-C
2 Origin	Bruker BioSpin GmbH
3 Solvent	CDCl3
4 Temperature	300.0
5 Number of Scans	56
6 Acquisition Time	1.3631
7 Acquisition Date	2021-11-19T09:35:12
8 Spectrometer Frequency	100.61
9 Spectral Width	24038.5

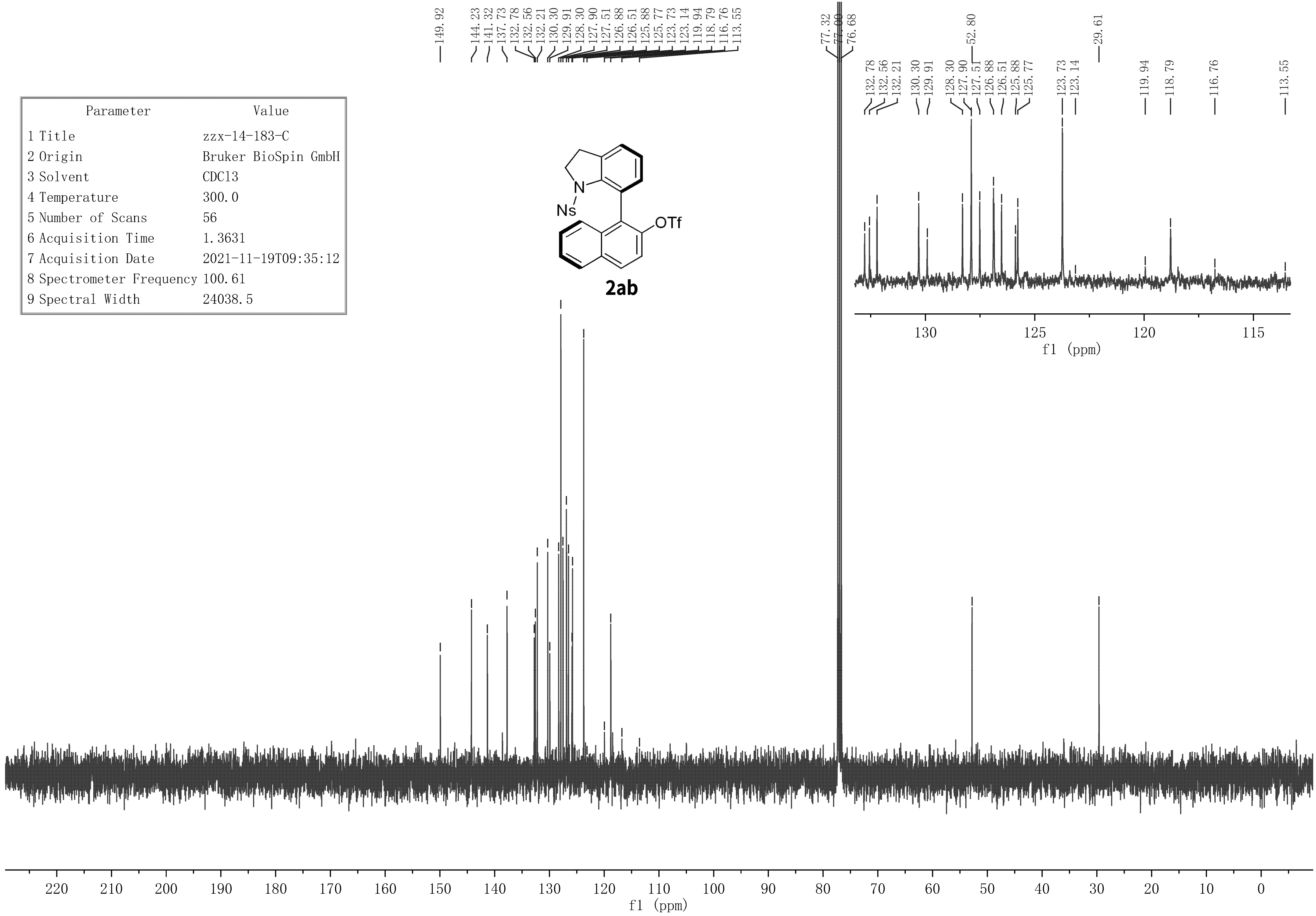

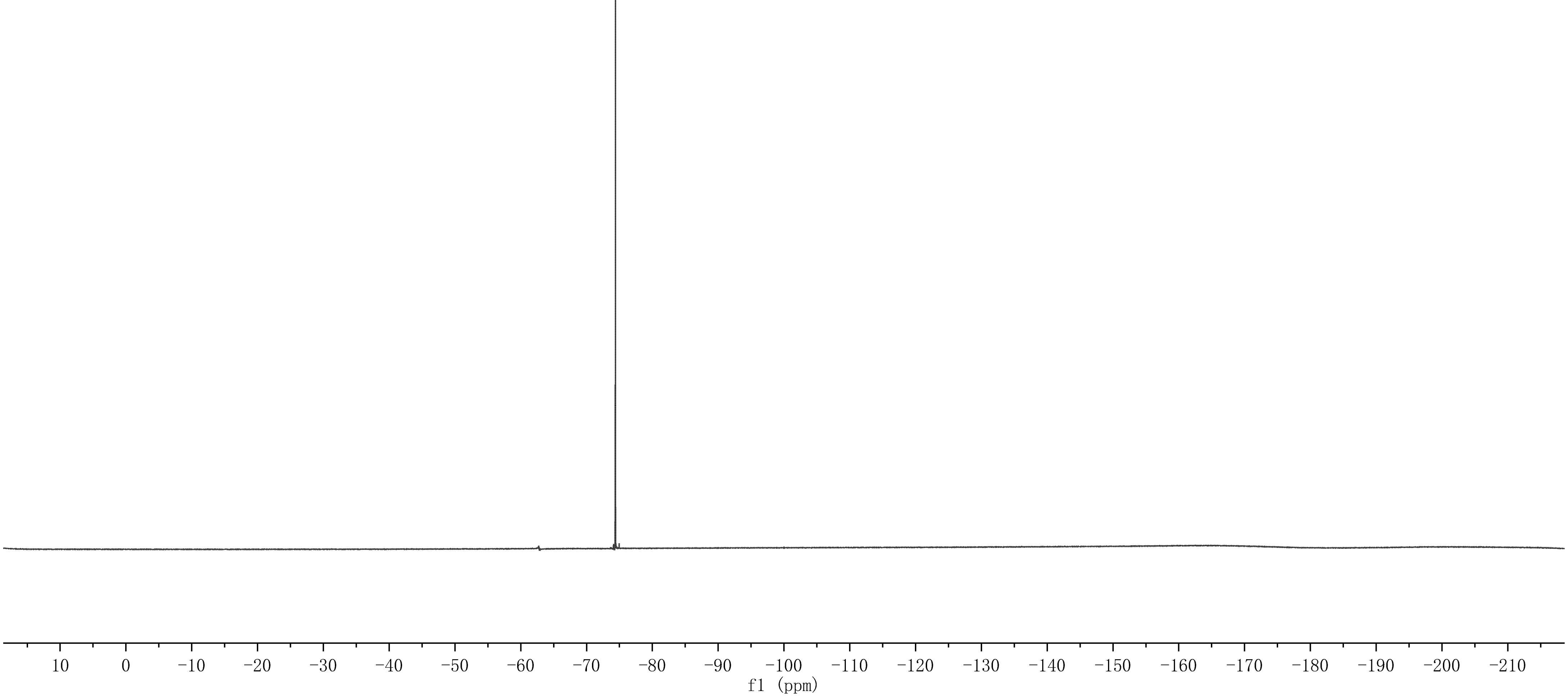

	Parameter	Value
1	Title	zzx-14-183-F
2	Origin	Bruker BioSpin GmbH
3	Solvent	CDCl3
4	Temperature	295.3
5	Number of Scans	12
6	Acquisition Time	0.7340
7	Acquisition Date	2021-11-27T10:23:45
8	Spectrometer Frequency	376.31
9	Spectral Width	89285.7

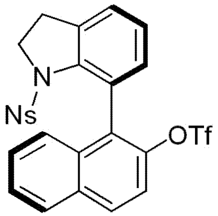

2ab

Parameter	Value
1 Title	zzx-18-8-Me-OTf
2 Origin	Bruker BioSpin GmbH
3 Solvent	CDCl3
4 Temperature	298.0
5 Number of Scans	9
6 Acquisition Time	4.0894
7 Acquisition Date	2023-02-24T10:07:41
8 Spectrometer Frequency	400.13
9 Spectral Width	8012.8

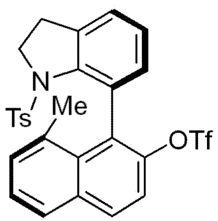

2ac

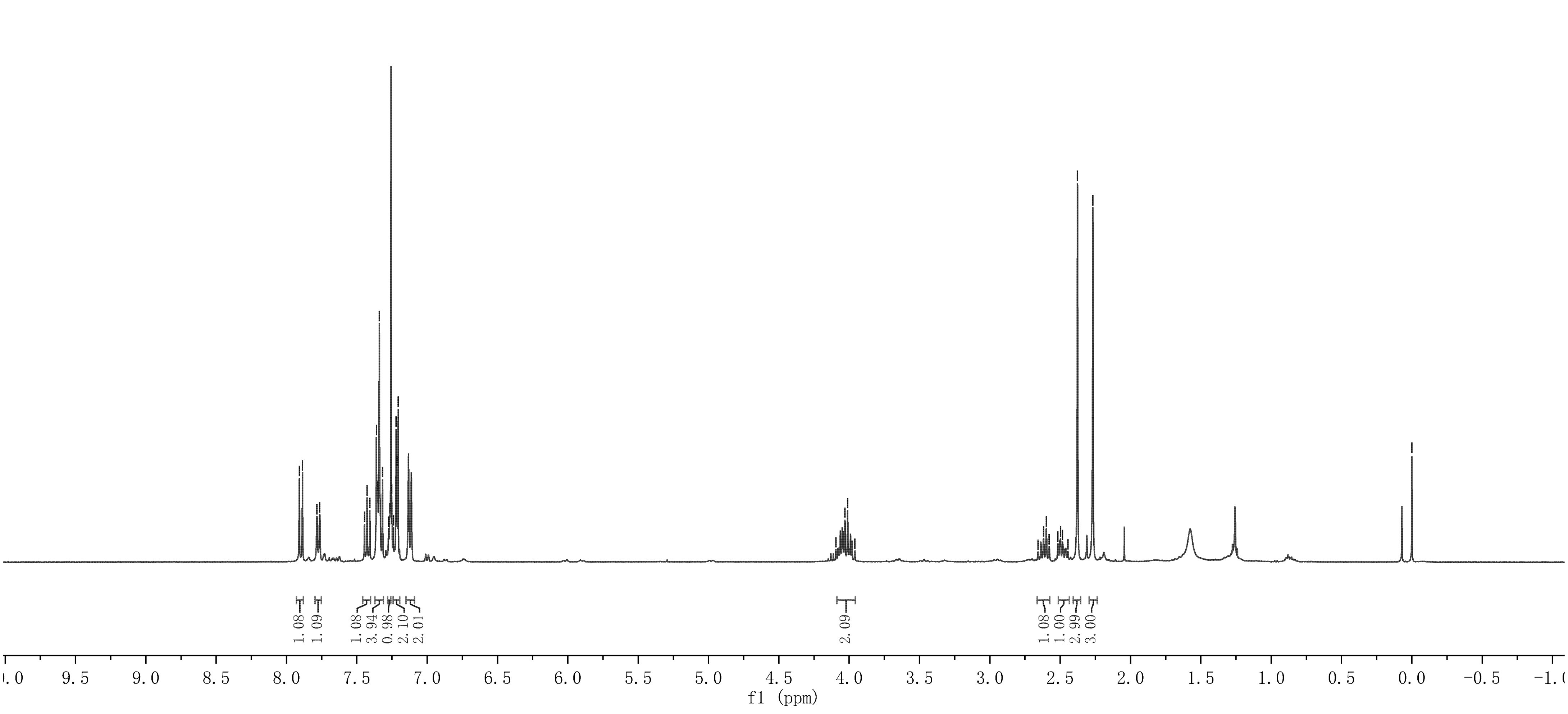

Parameter	Value
1 Title	zzx-12-225-C
2 Origin	Bruker BioSpin GmbH
3 Solvent	CDCl3
4 Temperature	300.0
5 Number of Scans	102
6 Acquisition Time	1.3631
7 Acquisition Date	2021-03-27T21:55:32
8 Spectrometer Frequency	100.61
9 Spectral Width	24038.5

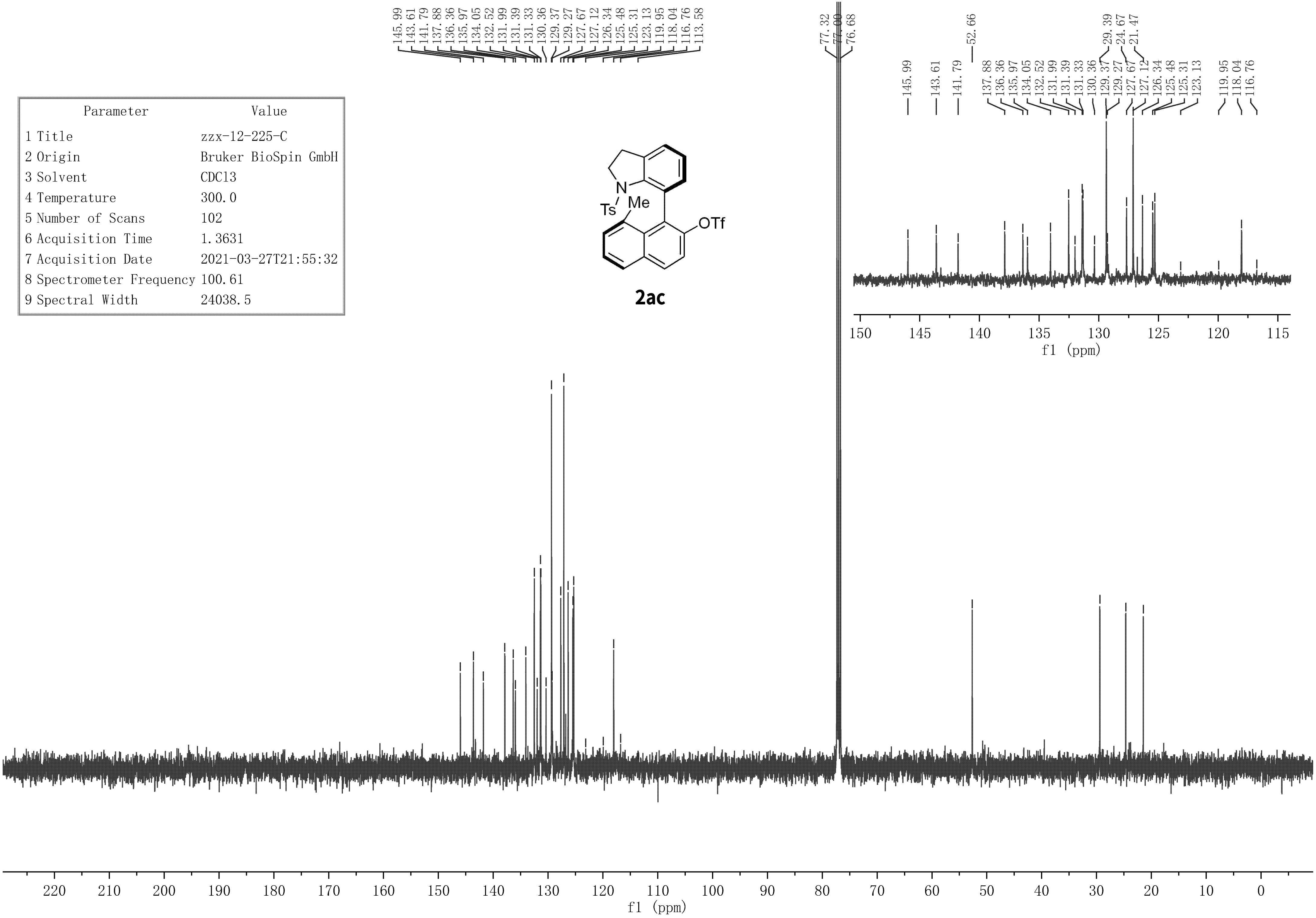

Parameter	Value
1 Title	zzx-18-8-Me-OTf-F
2 Origin	
3 Solvent	CDCl3
4 Temperature	296.4
5 Number of Scans	16
6 Acquisition Time	1.0000
7 Acquisition Date	2023-02-25T04:42:30
8 Spectrometer Frequency	376.28
9 Spectral Width	96153.0

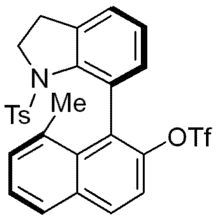

2ac

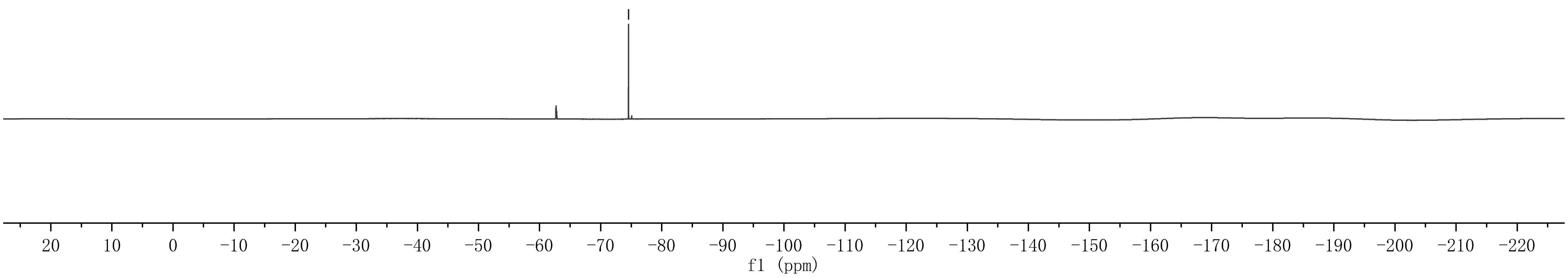

Parameter	Value
1 Title	ZZX-14-69
2 Origin	
3 Solvent	CDCl3
4 Temperature	298.0
5 Number of Scans	16
6 Acquisition Time	4.0002
7 Acquisition Date	2022-03-19T01:12:01
8 Spectrometer Frequency	399.93
9 Spectral Width	8012.0

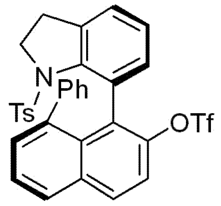

2ad

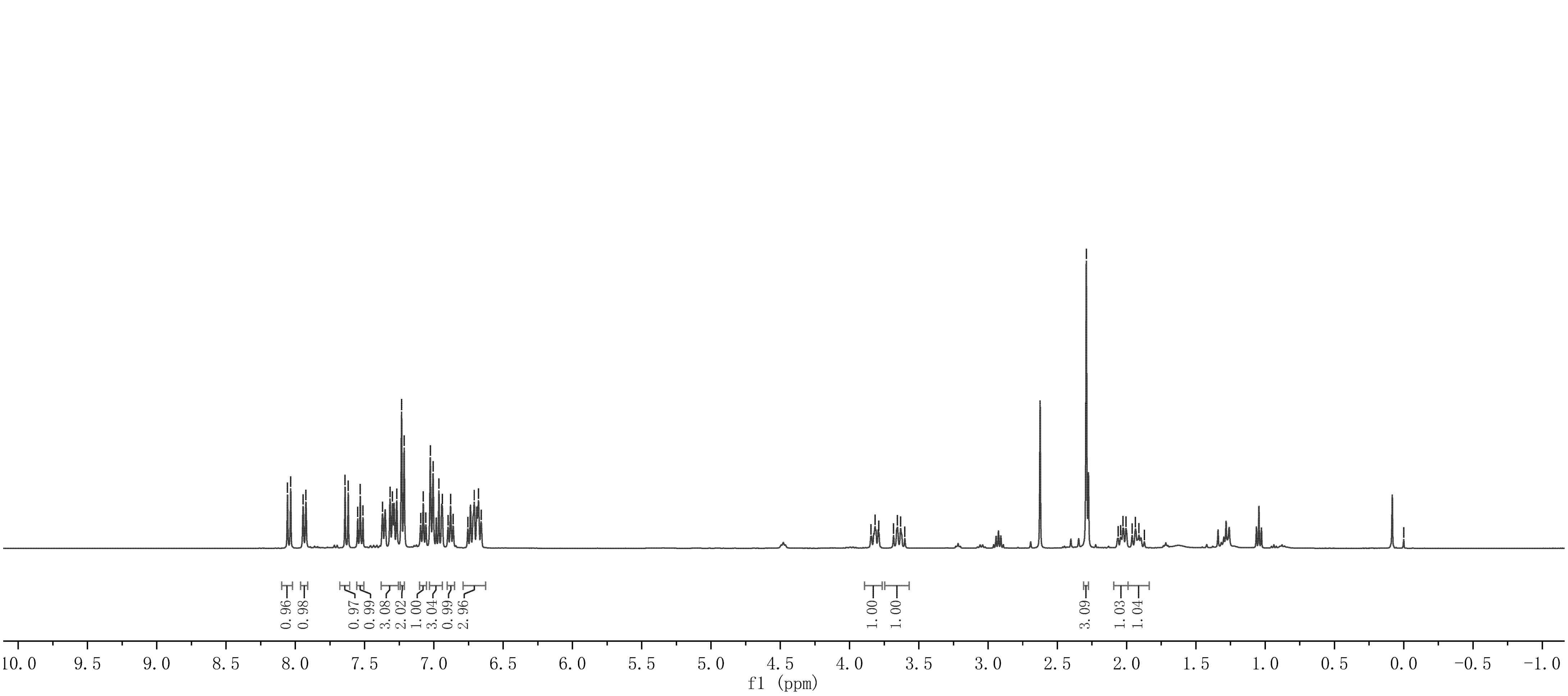

Parameter	Value
1 Title	ZZX-14-69
2 Origin	
3 Solvent	CDCl3
4 Temperature	297.9
5 Number of Scans	500
6 Acquisition Time	1.0000
7 Acquisition Date	2022-03-19T01:29:15
8 Spectrometer Frequency	100.56
9 Spectral Width	26041.0

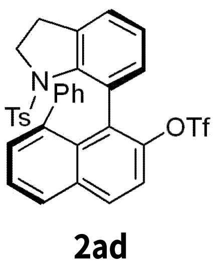

2ad

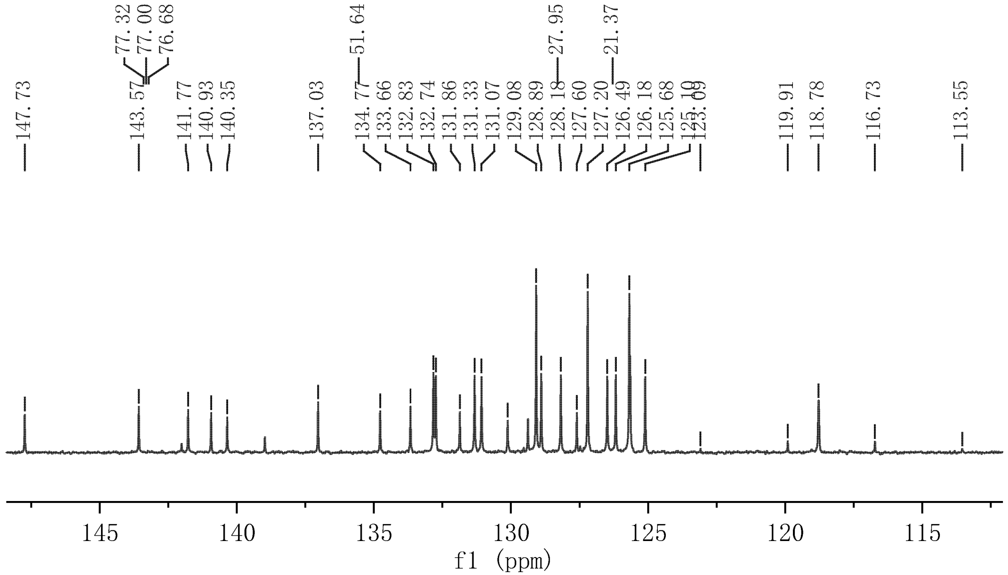

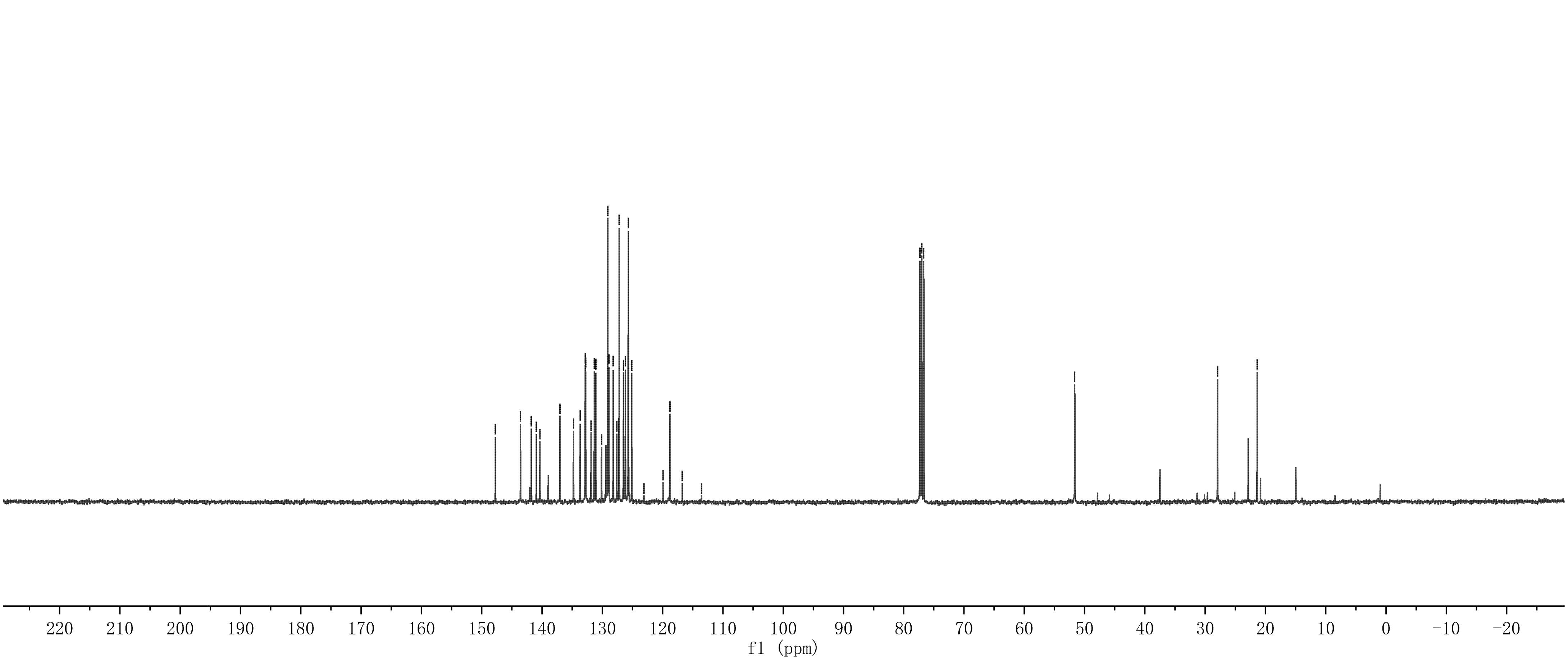

Parameter	Value
1 Title	ZZX-18-P-OTf-8-Ph
2 Origin	
3 Solvent	CDCl3
4 Temperature	297.4
5 Number of Scans	16
6 Acquisition Time	1.0000
7 Acquisition Date	2023-02-09T11:02:07
8 Spectrometer Frequency	376.28
9 Spectral Width	96153.0

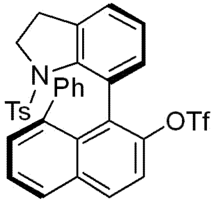

2ad

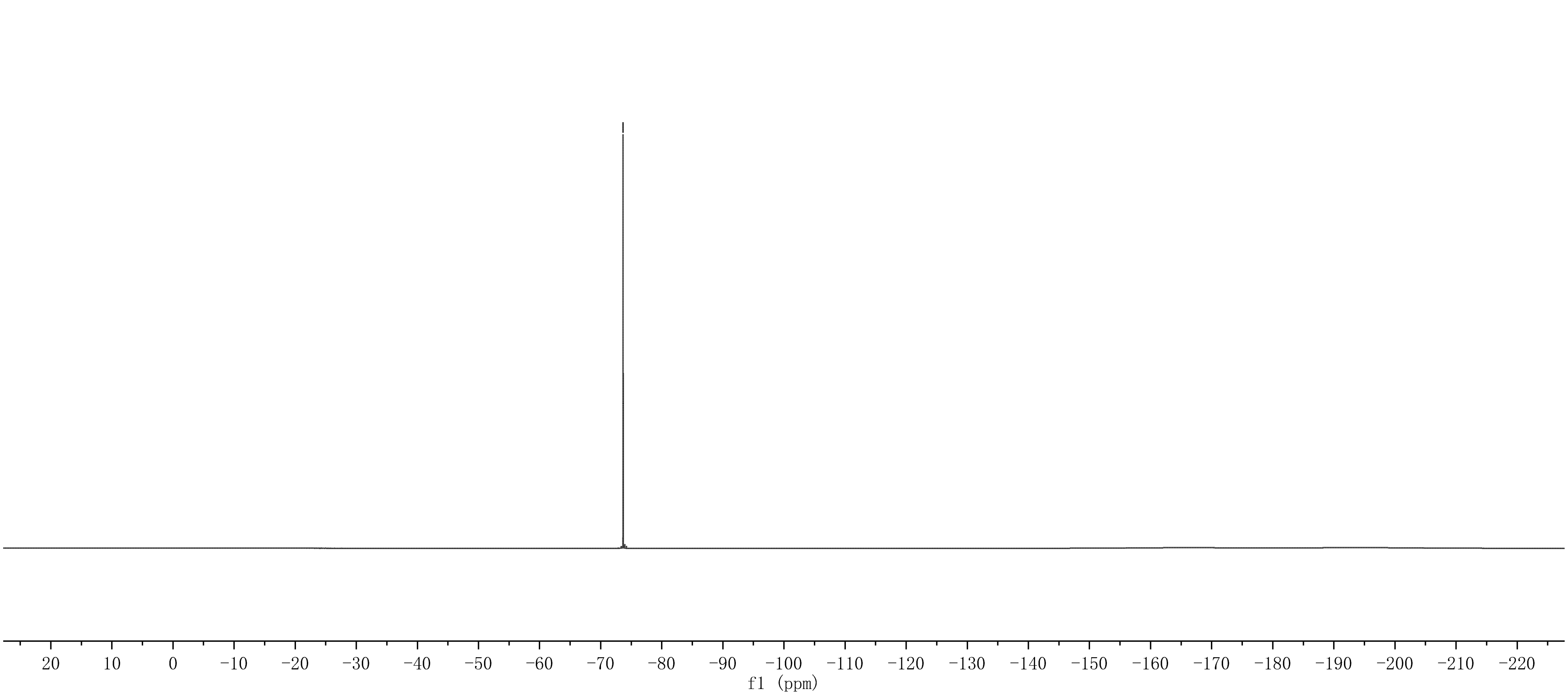

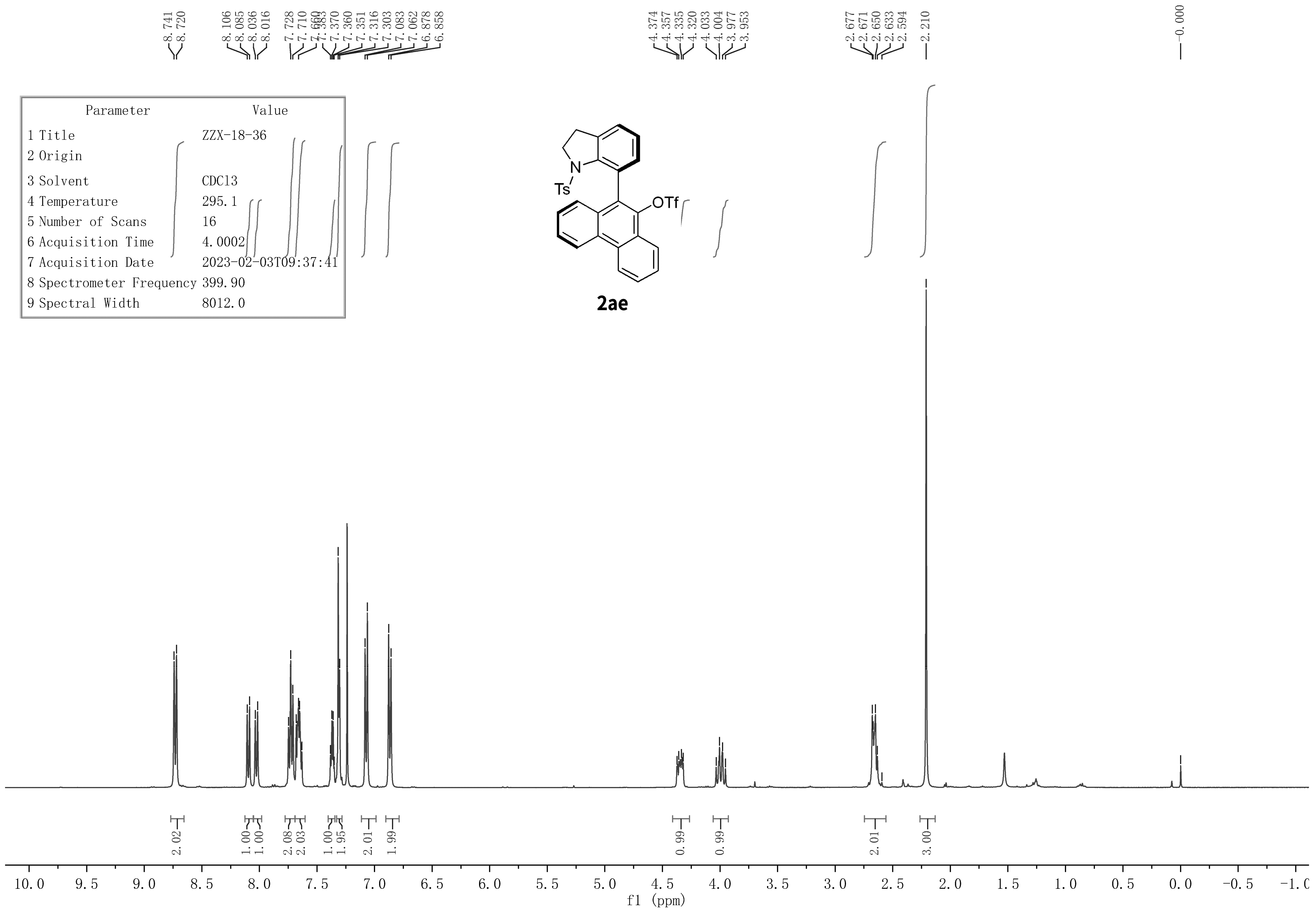
Parameter Value
1 Title ZZX-18-36
2 Origin
3 Solvent CDCl3
4 Temperature 295.1
5 Number of Scans 16
6 Acquisition Time 4.0002
7 Acquisition Date 2023-02-03T09:37:41
8 Spectrometer Frequency 399.90
9 Spectral Width 8012.0
N
Ts
OTf
2ae
8.741
8.720
8.106
8.085
8.036
8.016
7.728
7.710
[illegible]
7.383
7.370
7.360
7.351
7.316
7.303
7.083
7.062
6.878
6.858
4.374
4.357
4.335
4.320
4.033
4.004
3.977
3.953
2.677
2.671
2.650
2.633
2.594
2.210
0.000
2.02
1.00
1.00
2.08
2.03
1.00
1.95
2.01
1.99
0.99
0.99
2.01
3.00
10.0 9.5 9.0 8.5 8.0 7.5 7.0 6.5 6.0 5.5 5.0 4.5 4.0 3.5 3.0 2.5 2.0 1.5 1.0 0.5 0.0 -0.5 -1.0
f1 (ppm)

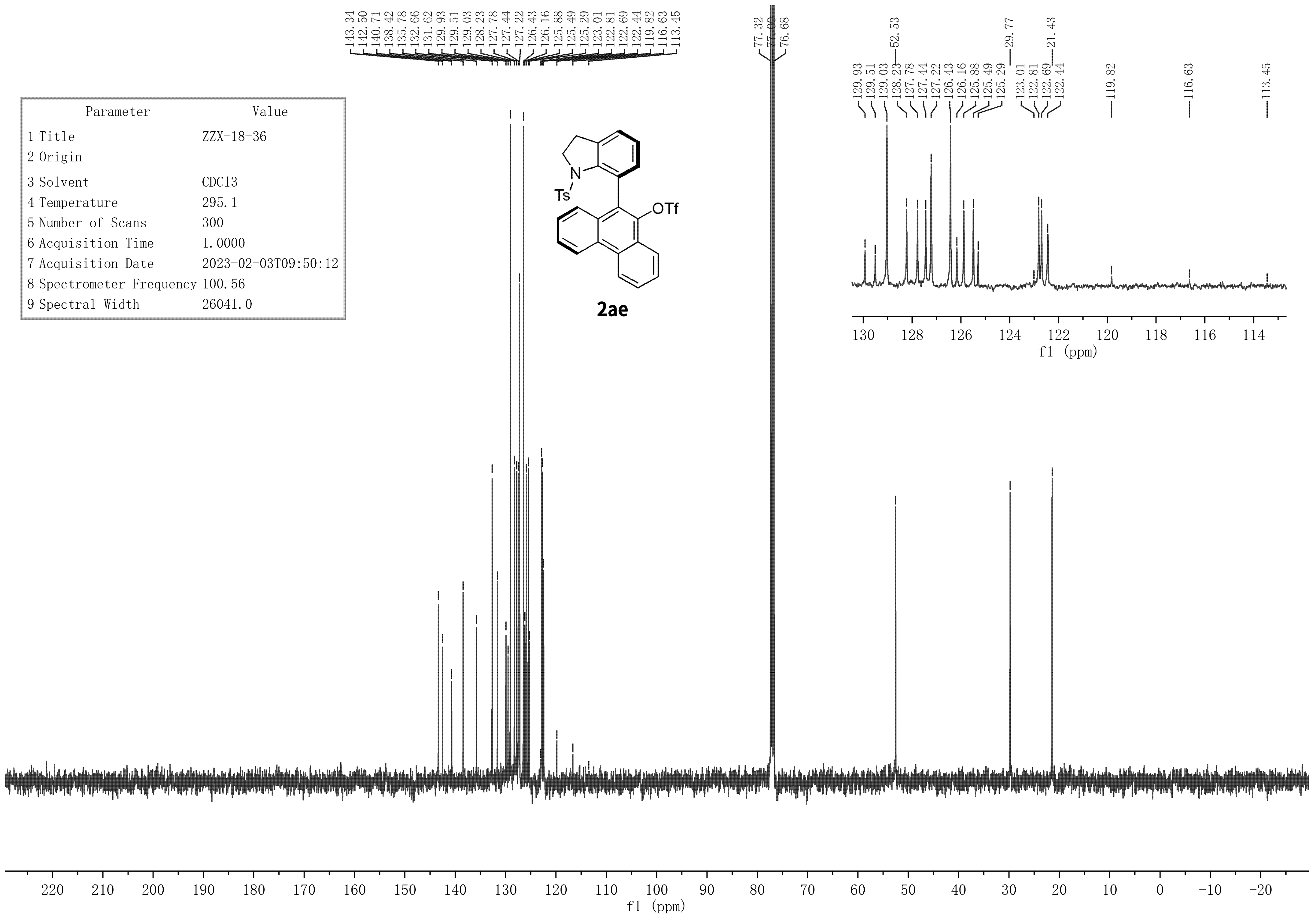

143.34
142.50
140.71
138.42
135.78
132.66
131.62
129.93
129.51
129.03
128.23
127.78
127.44
127.22
126.43
126.16
125.88
125.49
125.29
123.01
122.81
122.69
122.44
119.82
116.63
113.45
77.32
77.00
76.68
52.53
29.77
21.43
Parameter Value
1 Title ZZX-18-36
2 Origin
3 Solvent CDCl3
4 Temperature 295.1
5 Number of Scans 300
6 Acquisition Time 1.0000
7 Acquisition Date 2023-02-03T09:50:12
8 Spectrometer Frequency 100.56
9 Spectral Width 26041.0
N
Ts
OTf
f1 (ppm)

2ae

Parameter	Value
1 Title	ZZX-18-36
2 Origin	
3 Solvent	CDCl3
4 Temperature	295.2
5 Number of Scans	16
6 Acquisition Time	1.0000
7 Acquisition Date	2023-02-03T09:53:26
8 Spectrometer Frequency	376.28
9 Spectral Width	96153.0

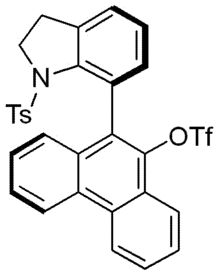

2ae

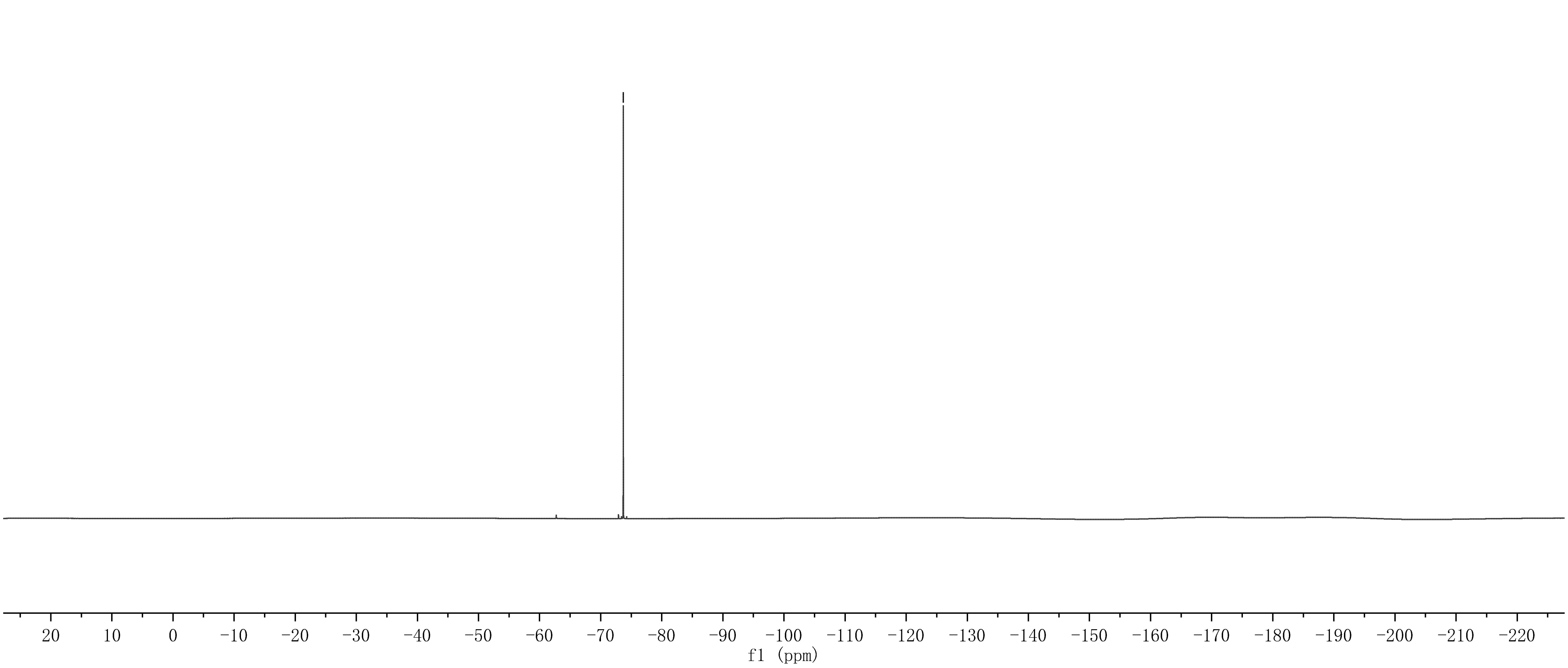

Parameter	Value
1 Title	ZZX-15-36
2 Origin	
3 Solvent	CDCl3
4 Temperature	297.2
5 Number of Scans	16
6 Acquisition Time	4.0002
7 Acquisition Date	2022-03-19T01:34:07
8 Spectrometer Frequency	399.93
9 Spectral Width	8012.0

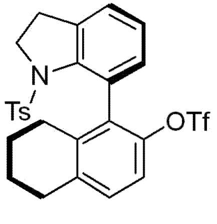

2af

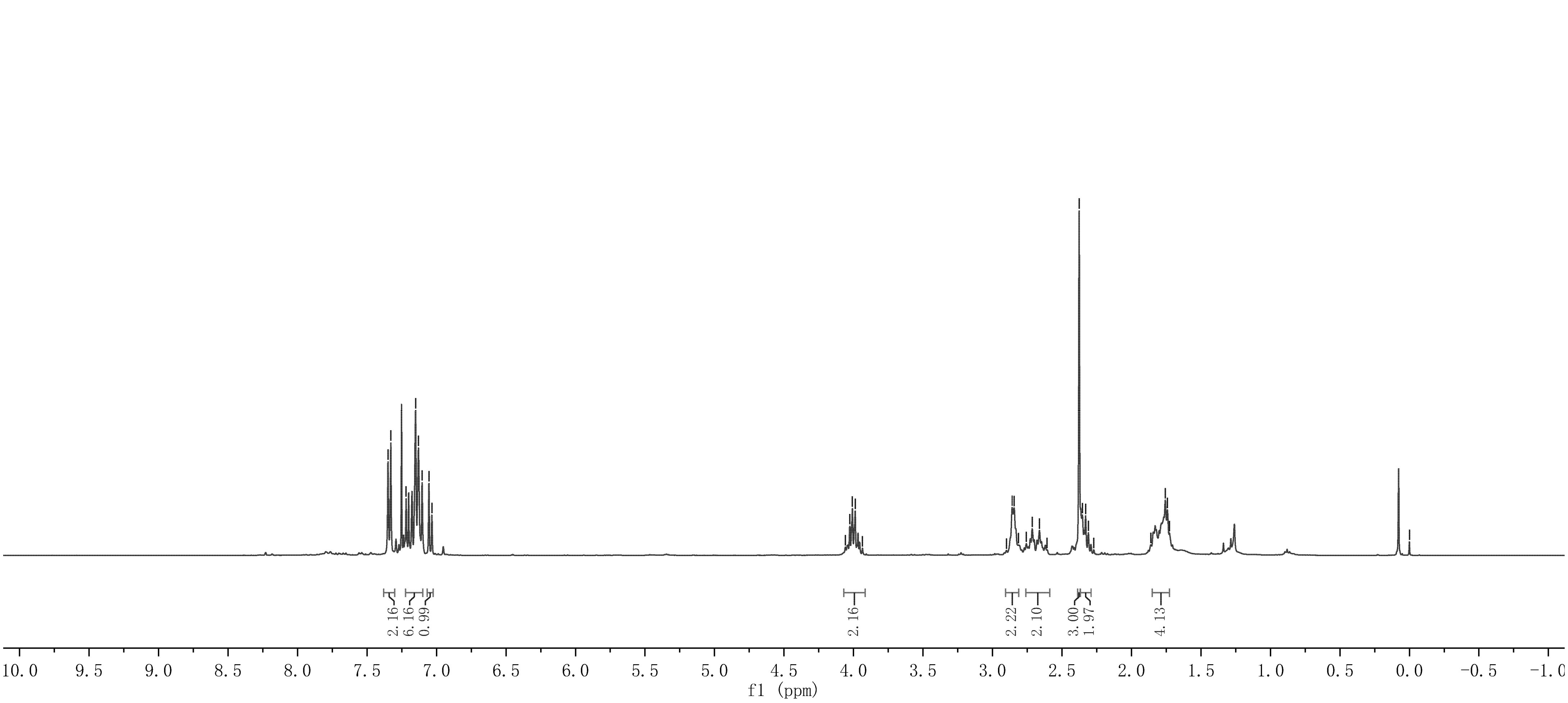

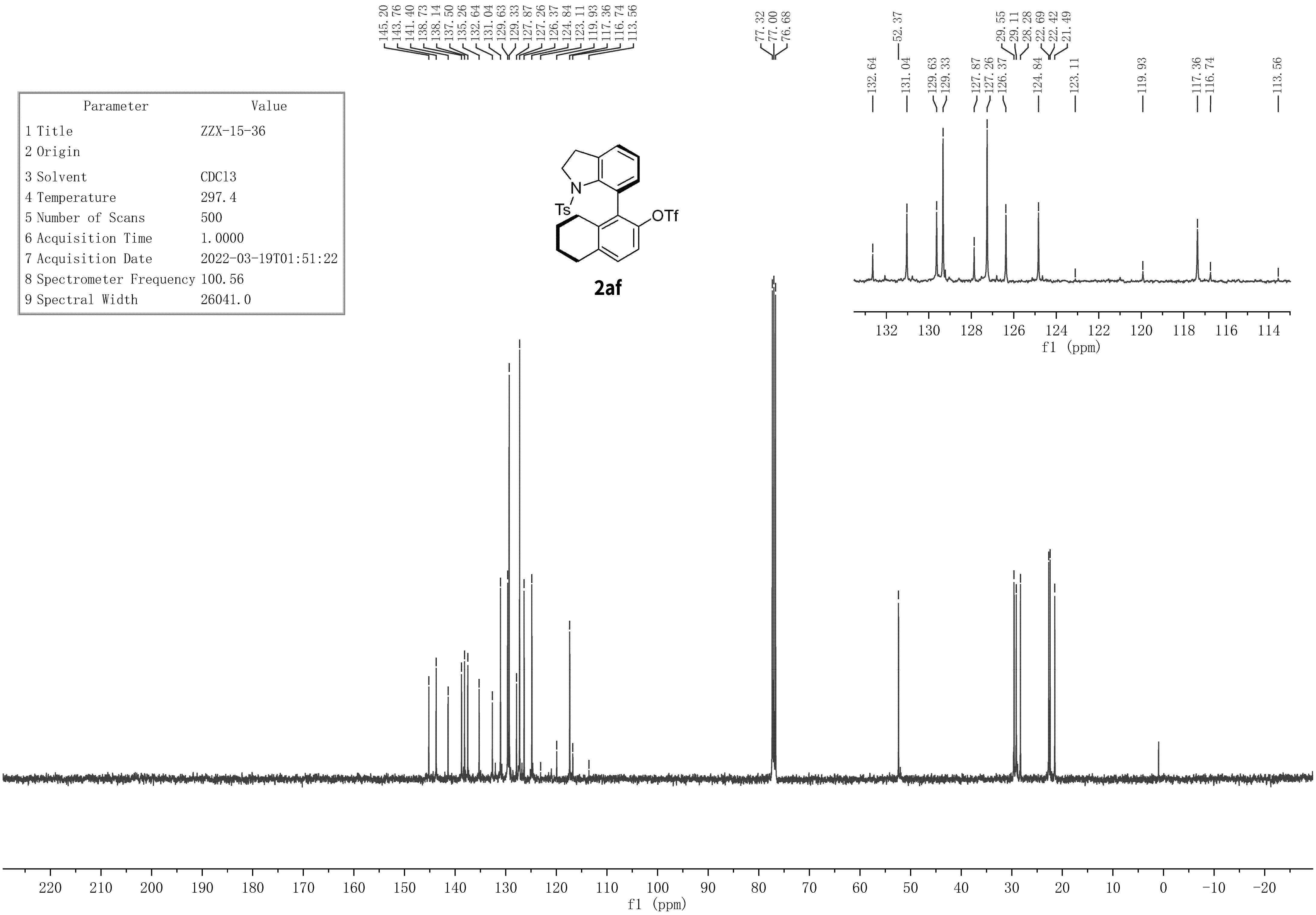

Parameter Value
1 Title ZZX-15-36
2 Origin
3 Solvent CDCl3
4 Temperature 297.4
5 Number of Scans 500
6 Acquisition Time 1.0000
7 Acquisition Date 2022-03-19T01:51:22
8 Spectrometer Frequency 100.56
9 Spectral Width 26041.0
N
Ts
OTf
2af
145.20 143.76 141.40 138.73 138.14 137.50 135.26 132.64 131.04 129.63 129.33 127.87 127.26 126.37 124.84 123.11 119.93 117.36 116.74 113.56
77.32 77.00 76.68
52.37
29.55 29.11 28.28 22.69 22.42 21.49
132.64 131.04 129.63 129.33 127.87 127.26 126.37 124.84 123.11 119.93 117.36 116.74 113.56
132 130 128 126 124 122 120 118 116 114
f1 (ppm)
220 210 200 190 180 170 160 150 140 130 120 110 100 90 80 70 60 50 40 30 20 10 0 -10 -20
f1 (ppm)

Parameter	Value
1 Title	ZZX-18-P-OTf-4H-naph
2 Origin	
3 Solvent	CDCl3
4 Temperature	297.4
5 Number of Scans	16
6 Acquisition Time	1.0000
7 Acquisition Date	2023-02-09T11:15:30
8 Spectrometer Frequency	376.28
9 Spectral Width	96153.0

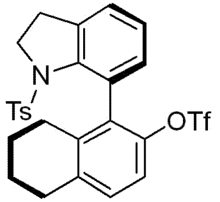

2af

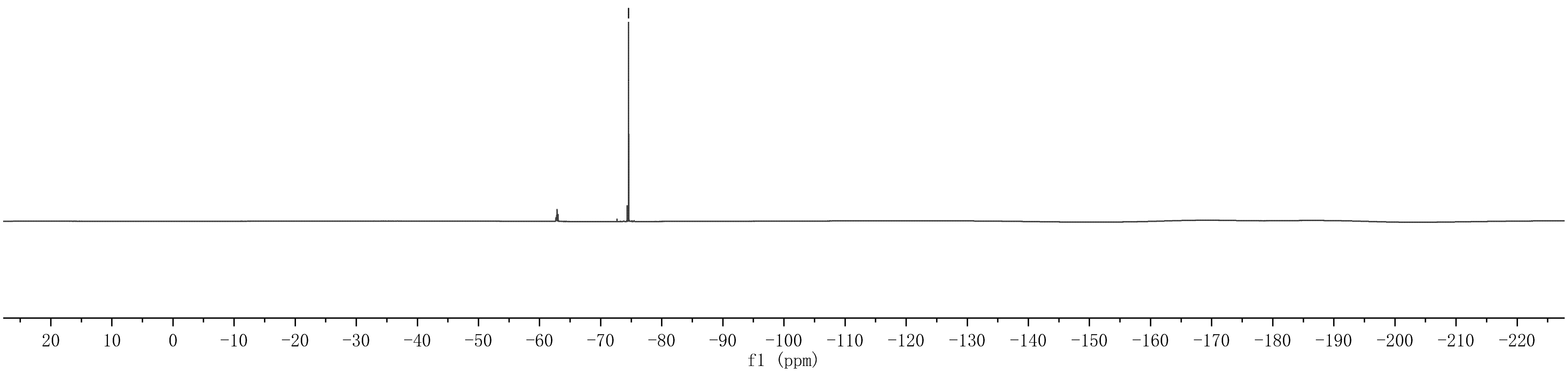

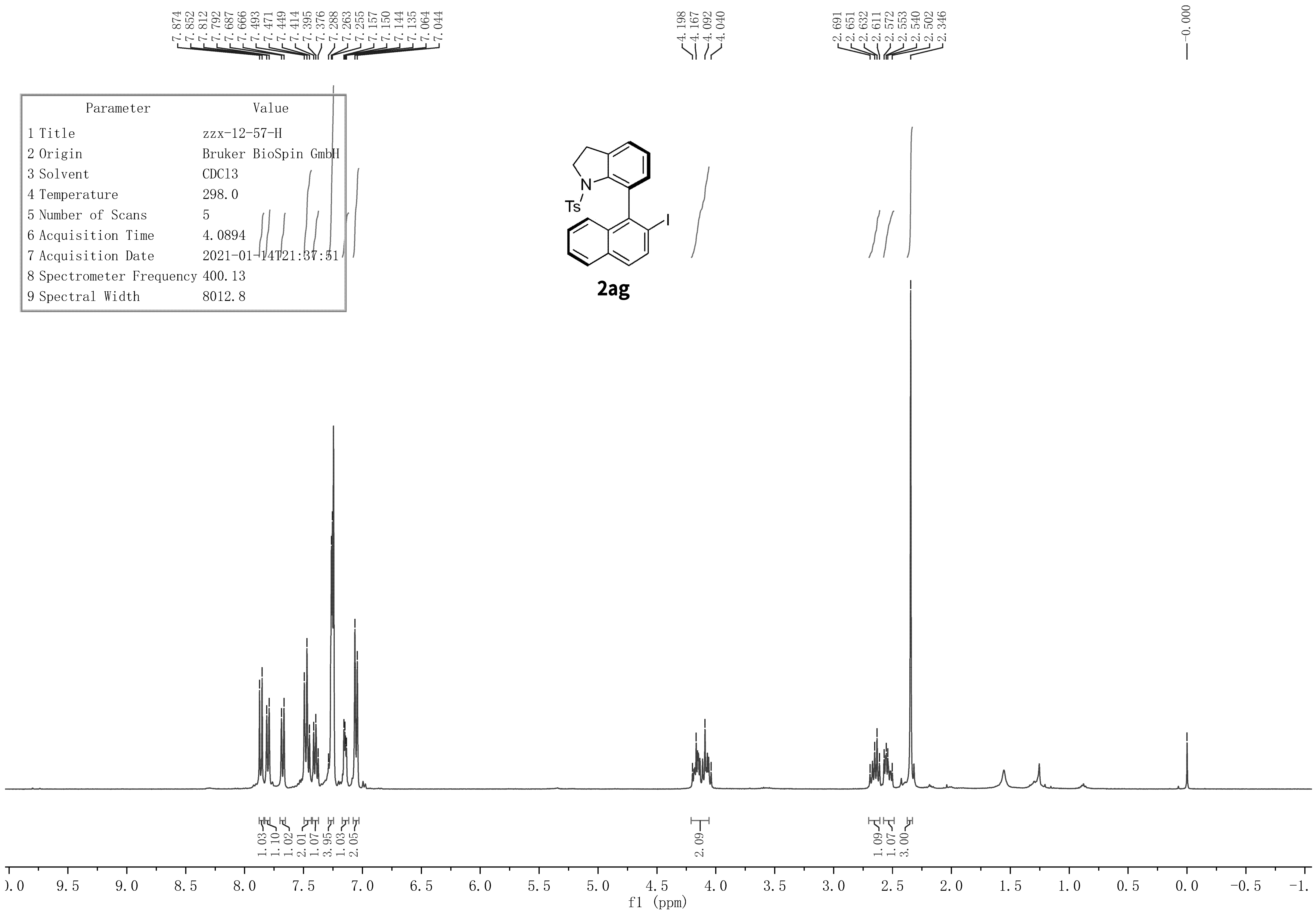
7.874 7.852 7.812 7.792 7.687 7.666 7.493 7.471 7.449 7.414 7.395 7.376 7.288 7.263 7.255 7.157 7.150 7.144 7.135 7.064 7.044
4.198 4.167 4.092 4.040
2.691 2.651 2.632 2.611 2.572 2.553 2.540 2.502 2.346
-0.000
Parameter Value
1 Title zzx-12-57-H
2 Origin Bruker BioSpin GmbH
3 Solvent CDCl3
4 Temperature 298.0
5 Number of Scans 5
6 Acquisition Time 4.0894
7 Acquisition Date 2021-01-14T21:37:51
8 Spectrometer Frequency 400.13
9 Spectral Width 8012.8
N
Ts
I
2ag
1.03 1.10 1.02 2.01 1.07 3.95 1.03 2.05
2.09
1.09 1.07 3.00
9.5 9.0 8.5 8.0 7.5 7.0 6.5 6.0 5.5 5.0 4.5 4.0 3.5 3.0 2.5 2.0 1.5 1.0 0.5 0.0 -0.5
f1 (ppm)

Parameter	Value
1 Title	zzx-12-57-C
2 Origin	Bruker BioSpin GmbH
3 Solvent	CDCl3
4 Temperature	300.0
5 Number of Scans	159
6 Acquisition Time	1.3631
7 Acquisition Date	2021-01-14T21:39:54
8 Spectrometer Frequency	100.61
9 Spectral Width	24038.5

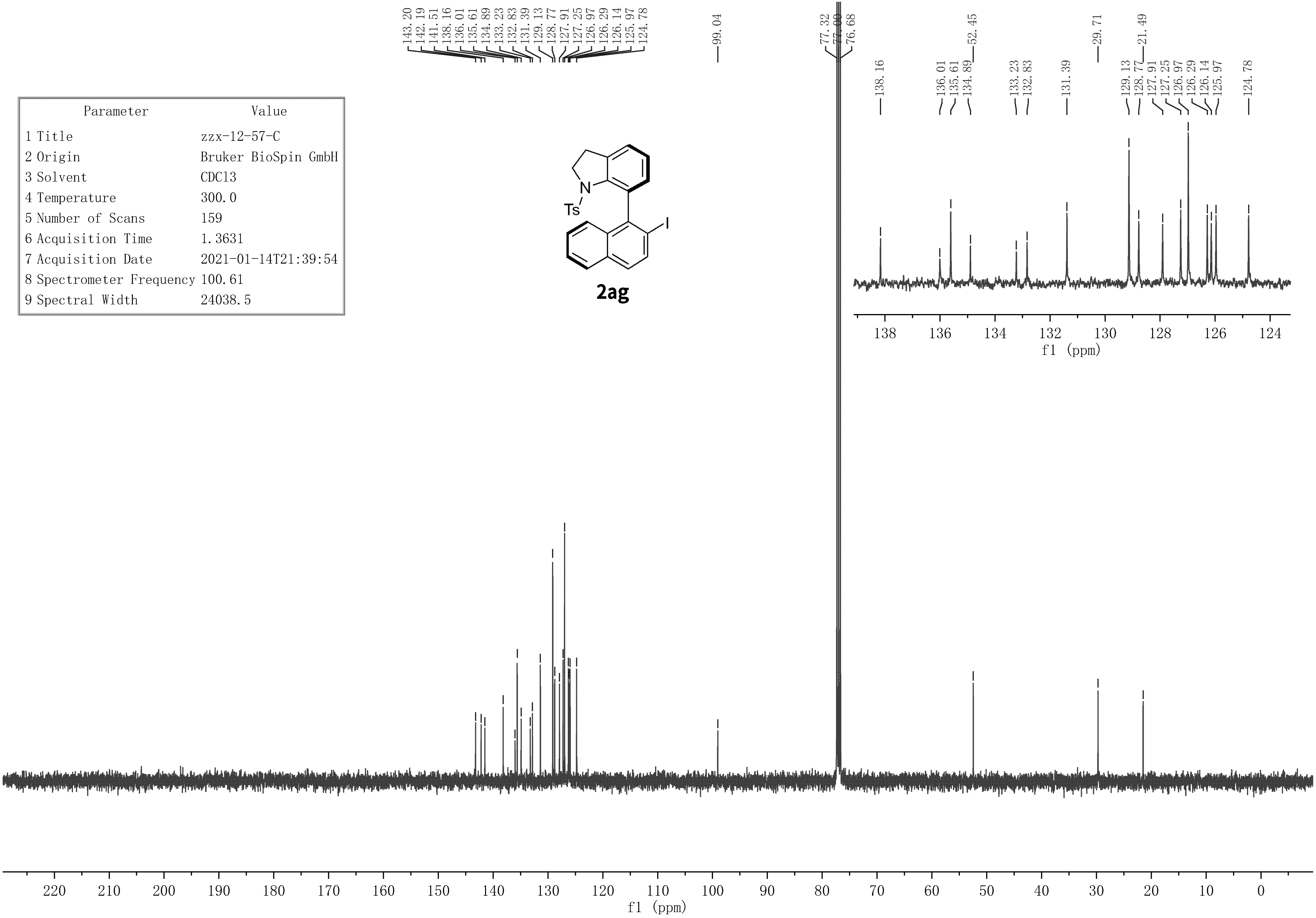

2ag

Parameter	Value
1 Title	zzx-11-119-H
2 Origin	Bruker BioSpin GmbH
3 Solvent	CDCl3
4 Temperature	298.0
5 Number of Scans	6
6 Acquisition Time	4.0894
7 Acquisition Date	2020-11-14T14:28:55
8 Spectrometer Frequency	400.13
9 Spectral Width	8012.8

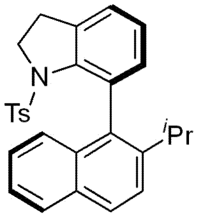

2ah

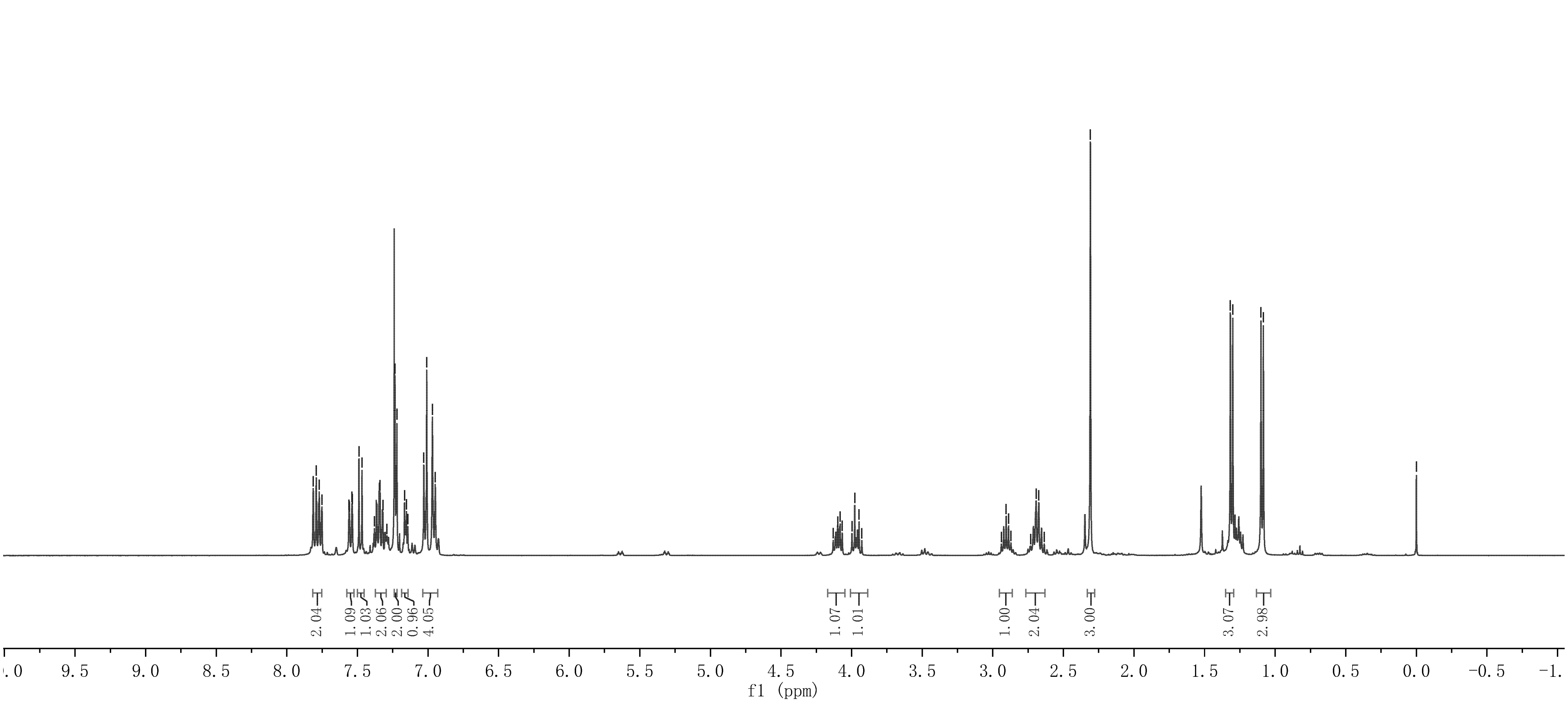

Parameter	Value
1 Title	zzx-11-119-C
2 Origin	Bruker BioSpin GmbH
3 Solvent	CDCl3
4 Temperature	300.0
5 Number of Scans	74
6 Acquisition Time	1.3631
7 Acquisition Date	2020-11-14T14:30:10
8 Spectrometer Frequency	100.61
9 Spectral Width	24038.5

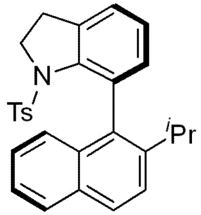

2ah

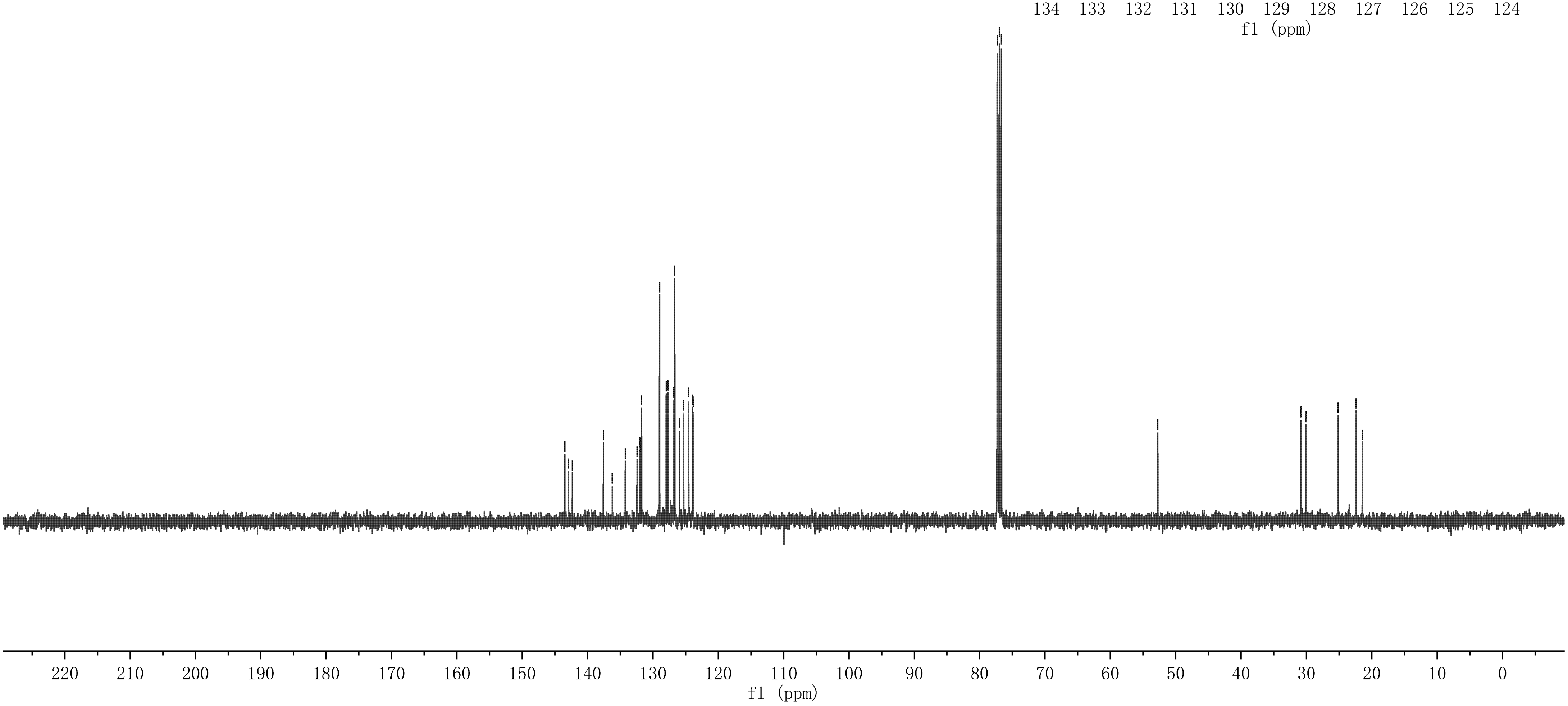

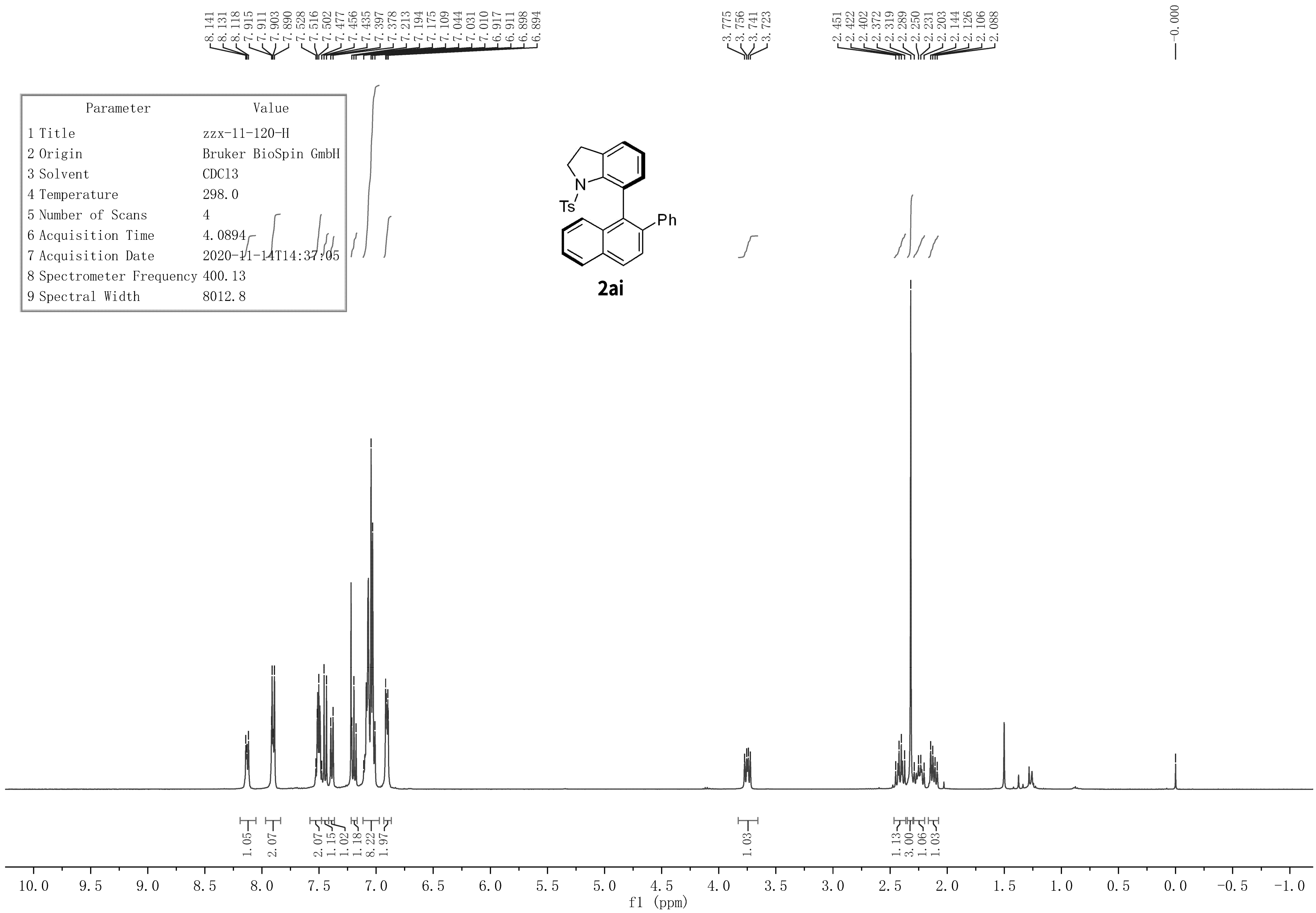
8.141 8.131 8.118 7.915 7.911 7.903 7.890 7.528 7.516 7.502 7.477 7.456 7.435 7.397 7.378 7.213 7.194 7.175 7.109 7.044 7.031 7.010 6.917 6.911 6.898 6.894
3.775 3.756 3.741 3.723
2.451 2.422 2.402 2.372 2.319 2.289 2.250 2.231 2.203 2.144 2.126 2.106 2.088
-0.000
Parameter Value
1 Title zzx-11-120-H
2 Origin Bruker BioSpin GmbH
3 Solvent CDCl3
4 Temperature 298.0
5 Number of Scans 4
6 Acquisition Time 4.0894
7 Acquisition Date 2020-11-14T14:37:05
8 Spectrometer Frequency 400.13
9 Spectral Width 8012.8
N
Ts
Ph
2ai
1.05 2.07 2.07 1.15 1.02 1.18 8.22 1.97
1.03
1.13 3.00 1.06 1.03
10.0 9.5 9.0 8.5 8.0 7.5 7.0 6.5 6.0 5.5 5.0 4.5 4.0 3.5 3.0 2.5 2.0 1.5 1.0 0.5 0.0 -0.5 -1.0
f1 (ppm)

Parameter	Value
1 Title	zzx-11-120-C
2 Origin	Bruker BioSpin GmbH
3 Solvent	CDCl3
4 Temperature	300.0
5 Number of Scans	21
6 Acquisition Time	1.3631
7 Acquisition Date	2020-11-14T14:38:45
8 Spectrometer Frequency	100.61
9 Spectral Width	24038.5

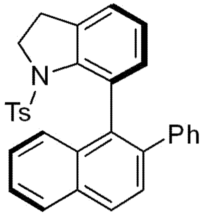

2ai

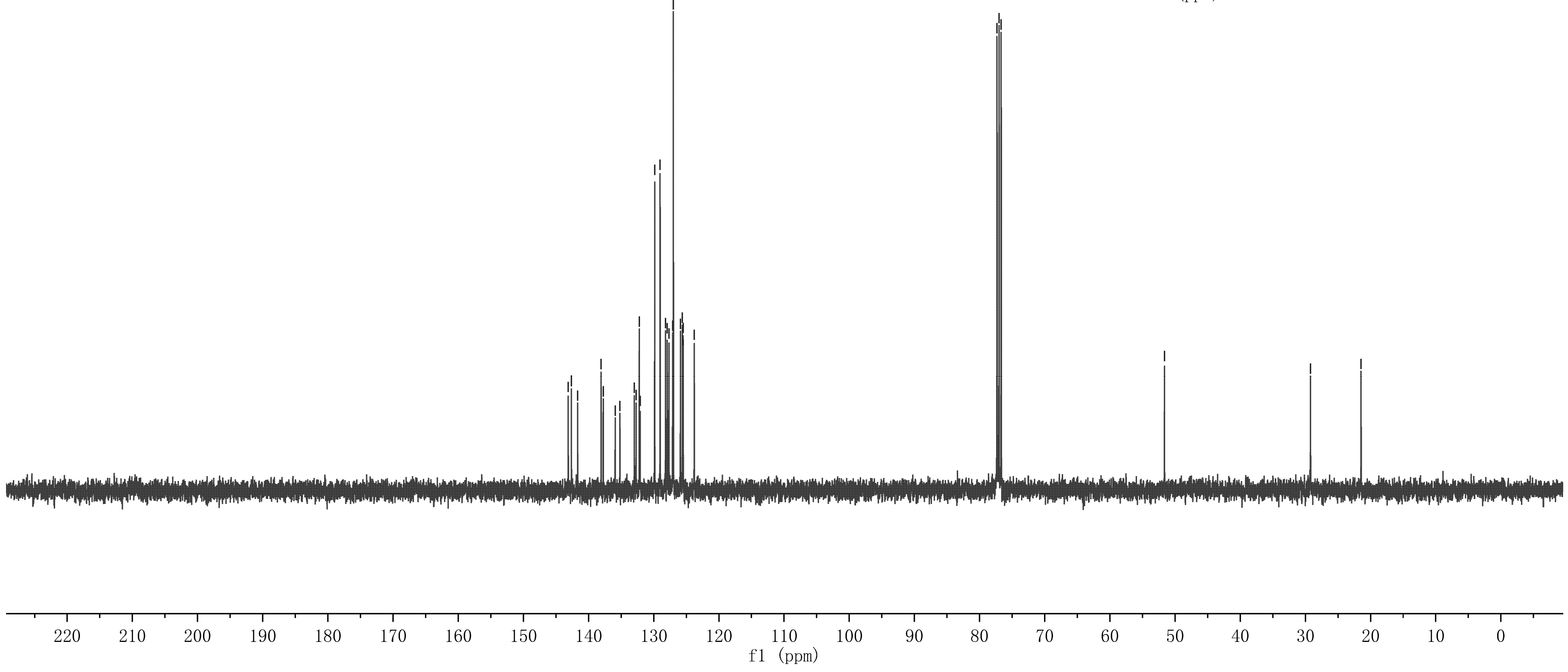

Parameter	Value
1 Title	ZZX-11-225
2 Origin	
3 Solvent	CDCl3
4 Temperature	297.2
5 Number of Scans	16
6 Acquisition Time	4.0002
7 Acquisition Date	2022-03-19T00:12:25
8 Spectrometer Frequency	399.93
9 Spectral Width	8012.0

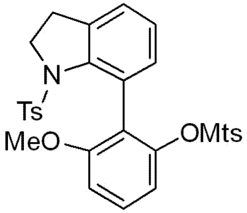

2aj

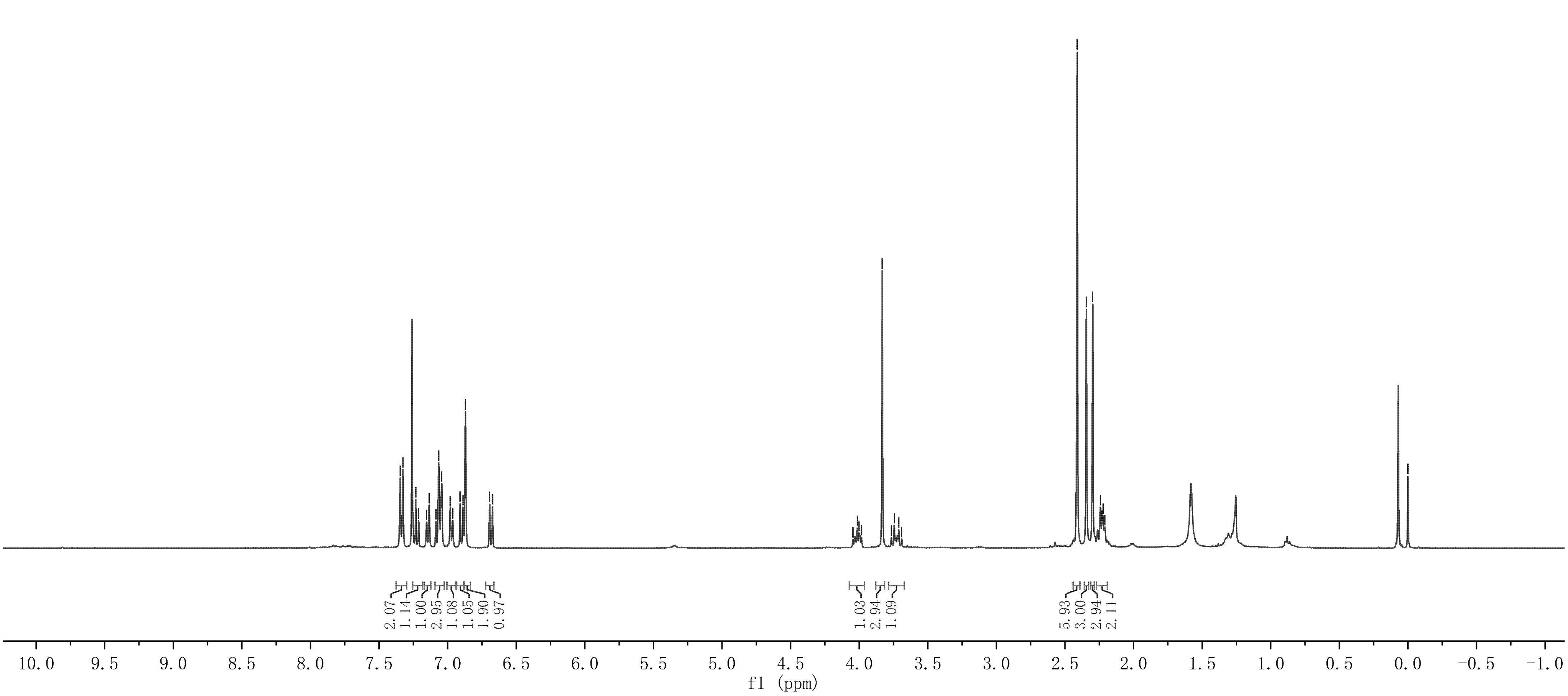

Parameter	Value
1 Title	ZZX-11-225
2 Origin	
3 Solvent	CDCl3
4 Temperature	297.4
5 Number of Scans	500
6 Acquisition Time	1.0000
7 Acquisition Date	2022-03-19T00:29:40
8 Spectrometer Frequency	100.56
9 Spectral Width	26041.0

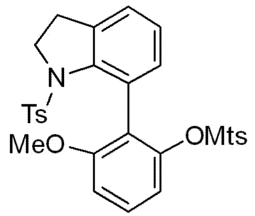

2aj

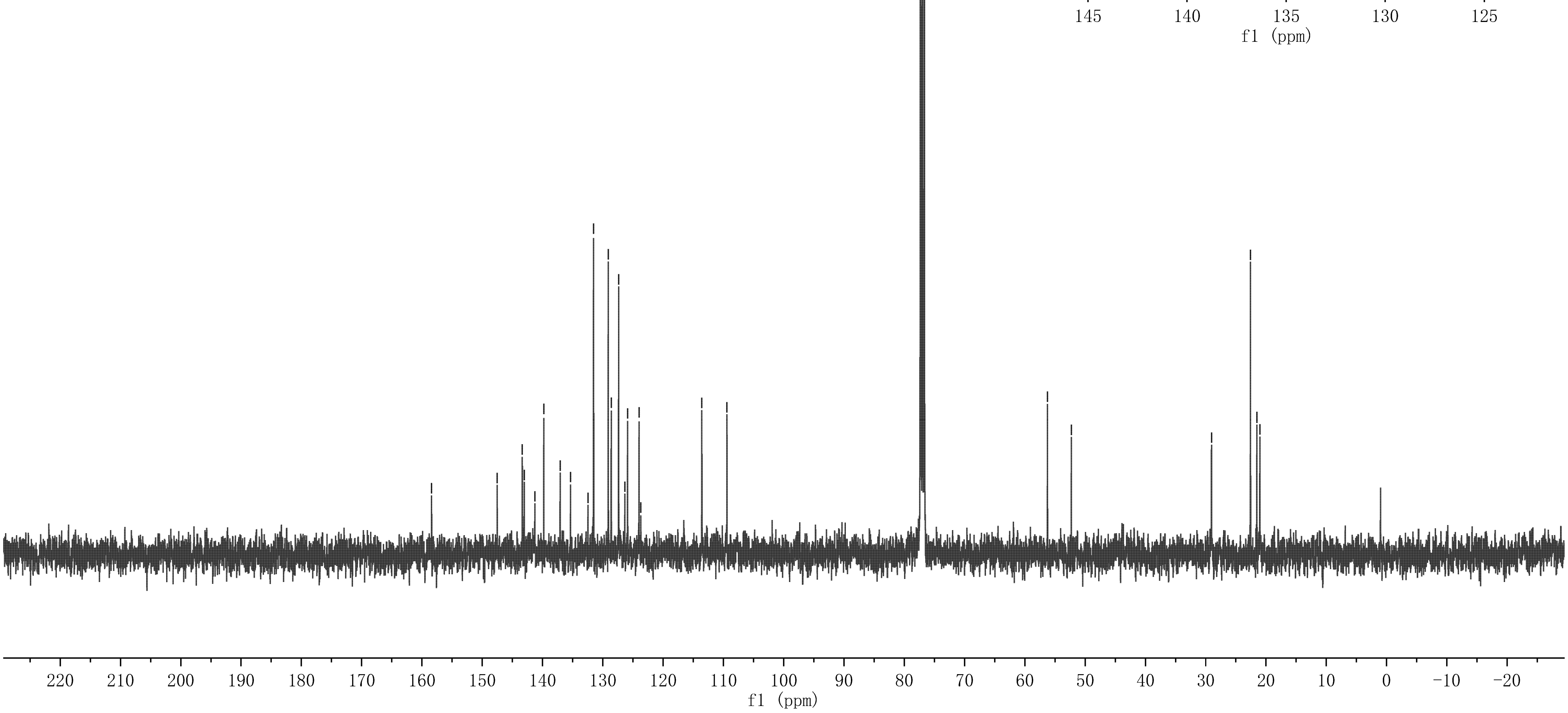

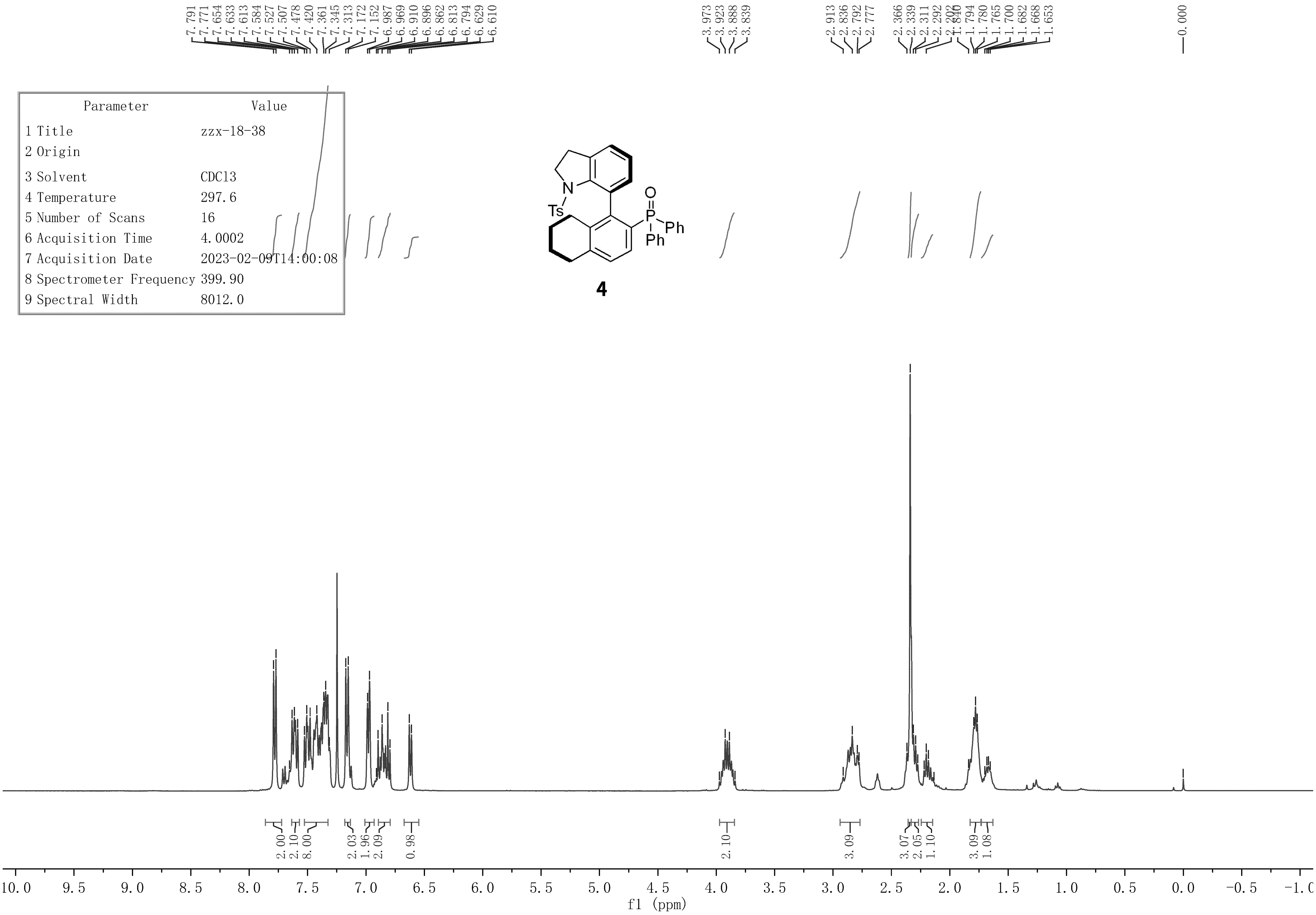
7.791
7.771
7.654
7.633
7.613
7.584
7.527
7.507
7.478
7.420
7.361
7.345
7.313
7.172
7.152
6.987
6.969
6.910
6.896
6.862
6.813
6.794
6.629
6.610
3.973
3.923
3.888
3.839
2.913
2.836
2.792
2.777
2.366
2.339
2.311
2.292
1.794
1.780
1.765
1.700
1.682
1.668
1.653
0.000
Parameter Value
1 Title zzx-18-38
2 Origin
3 Solvent CDCl3
4 Temperature 297.6
5 Number of Scans 16
6 Acquisition Time 4.0002
7 Acquisition Date 2023-02-09T14:00:08
8 Spectrometer Frequency 399.90
9 Spectral Width 8012.0
N
Ts
O
P
Ph
Ph
4
2.00
2.10
8.00
2.03
1.96
2.09
0.98
2.10
3.09
3.07
2.05
1.10
3.09
1.08
10.0 9.5 9.0 8.5 8.0 7.5 7.0 6.5 6.0 5.5 5.0 4.5 4.0 3.5 3.0 2.5 2.0 1.5 1.0 0.5 0.0 -0.5
f1 (ppm)

Parameter	Value
1 Title	zzx-18-38
2 Origin	
3 Solvent	CDCl3
4 Temperature	297.6
5 Number of Scans	600
6 Acquisition Time	1.0000
7 Acquisition Date	2023-02-09T14:22:52
8 Spectrometer Frequency	100.56
9 Spectral Width	26041.0

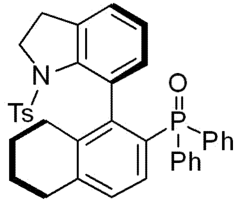

4

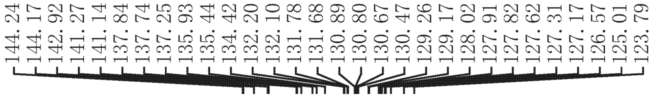

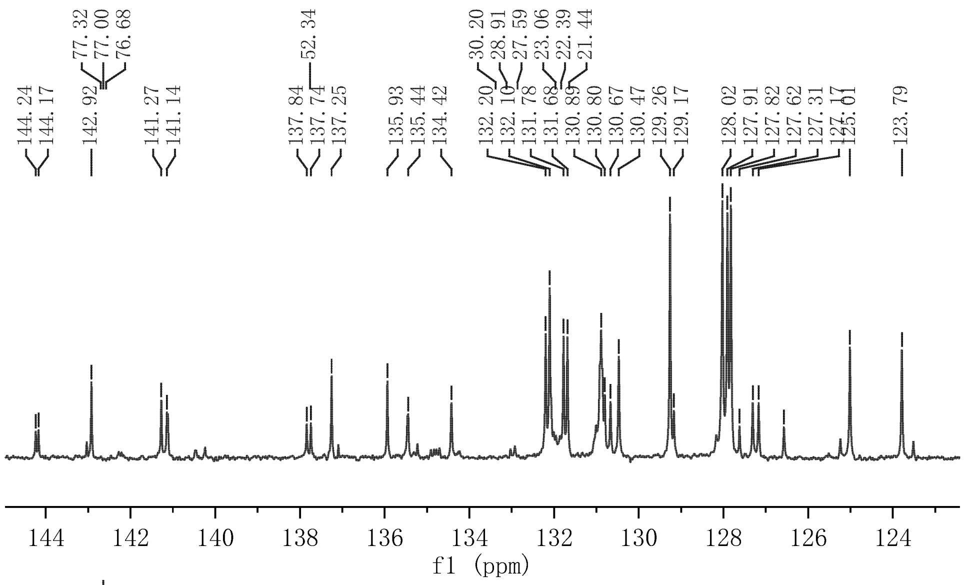

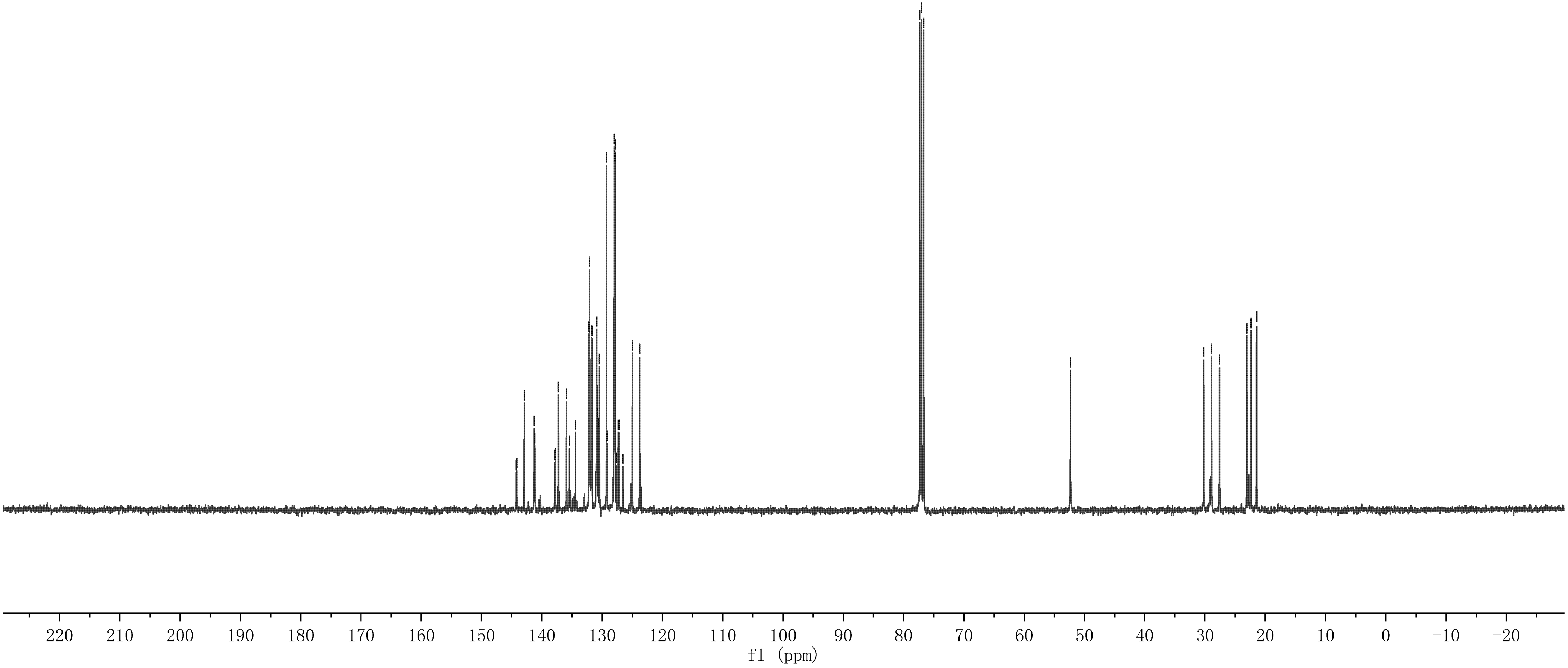

Parameter	Value
1 Title	zzx-15-229-dept
2 Origin	Bruker BioSpin GmbH
3 Solvent	CDCl3
4 Temperature	300.0
5 Number of Scans	34
6 Acquisition Time	1.3631
7 Acquisition Date	2022-04-19T20:25:25
8 Spectrometer Frequency	100.61
9 Spectral Width	24038.5

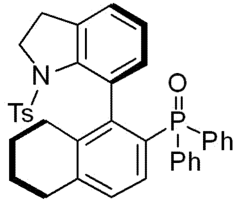

4

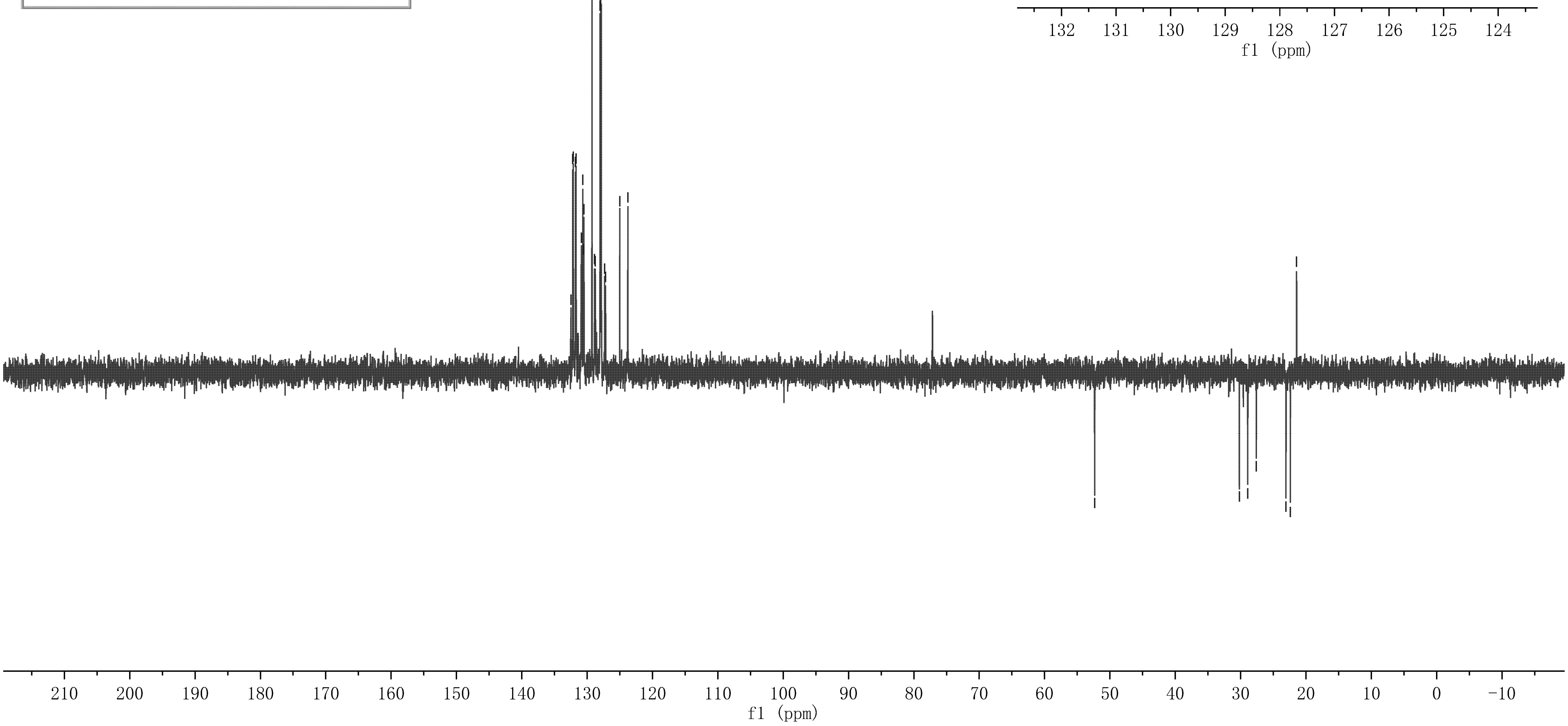

Parameter	Value
1 Title	ZZX-15-229
2 Origin	
3 Solvent	CDCl3
4 Temperature	297.8
5 Number of Scans	32
6 Acquisition Time	1.0000
7 Acquisition Date	2022-04-19T22:16:22
8 Spectrometer Frequency	161.89
9 Spectral Width	66371.0

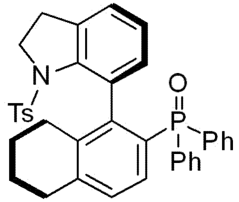

4

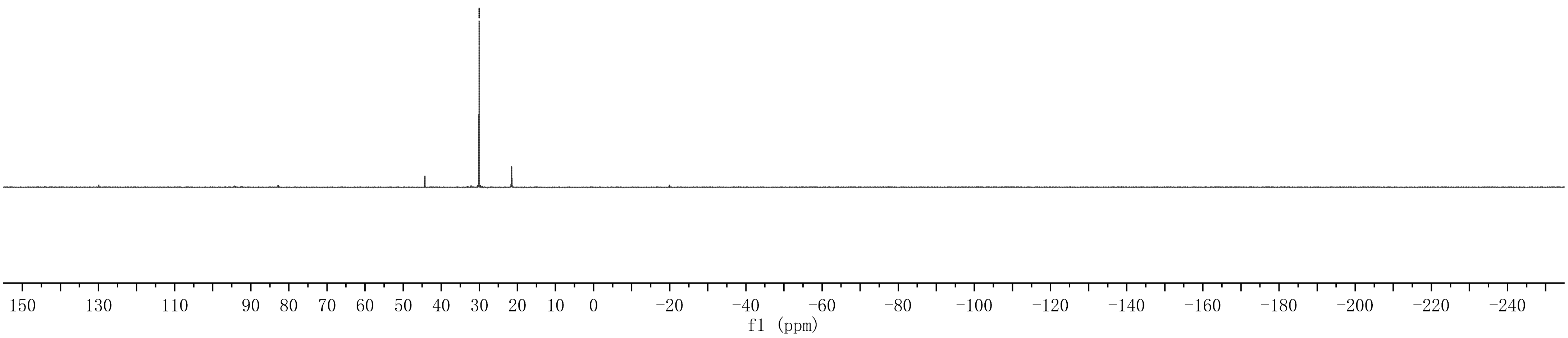

Parameter	Value
1 Title	zzx-8-48-H
2 Origin	Bruker BioSpin GmbH
3 Solvent	CDCl3
4 Temperature	298.0
5 Number of Scans	10
6 Acquisition Time	4.0894
7 Acquisition Date	2023-02-19T20:07:14
8 Spectrometer Frequency	400.13
9 Spectral Width	8012.8

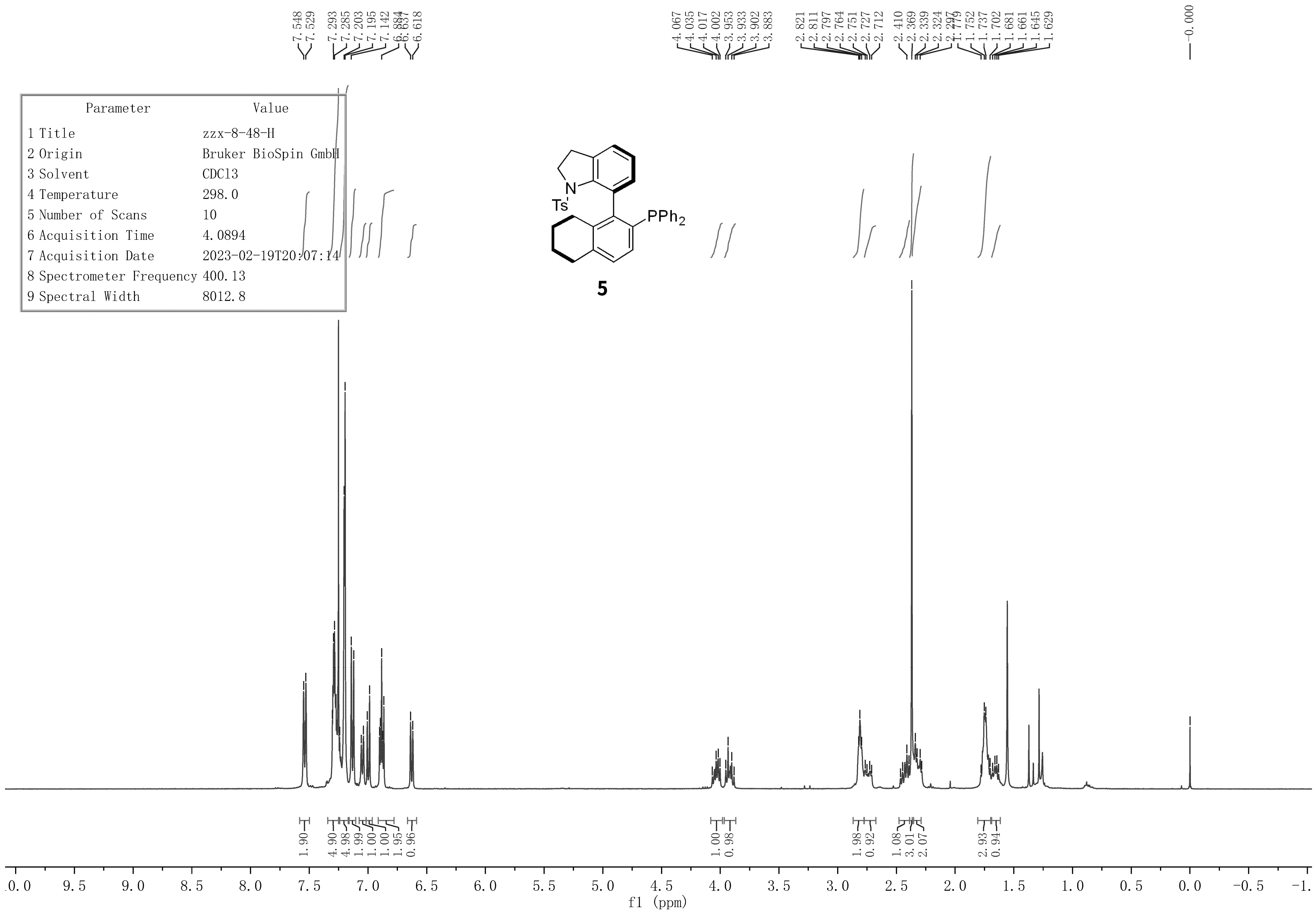

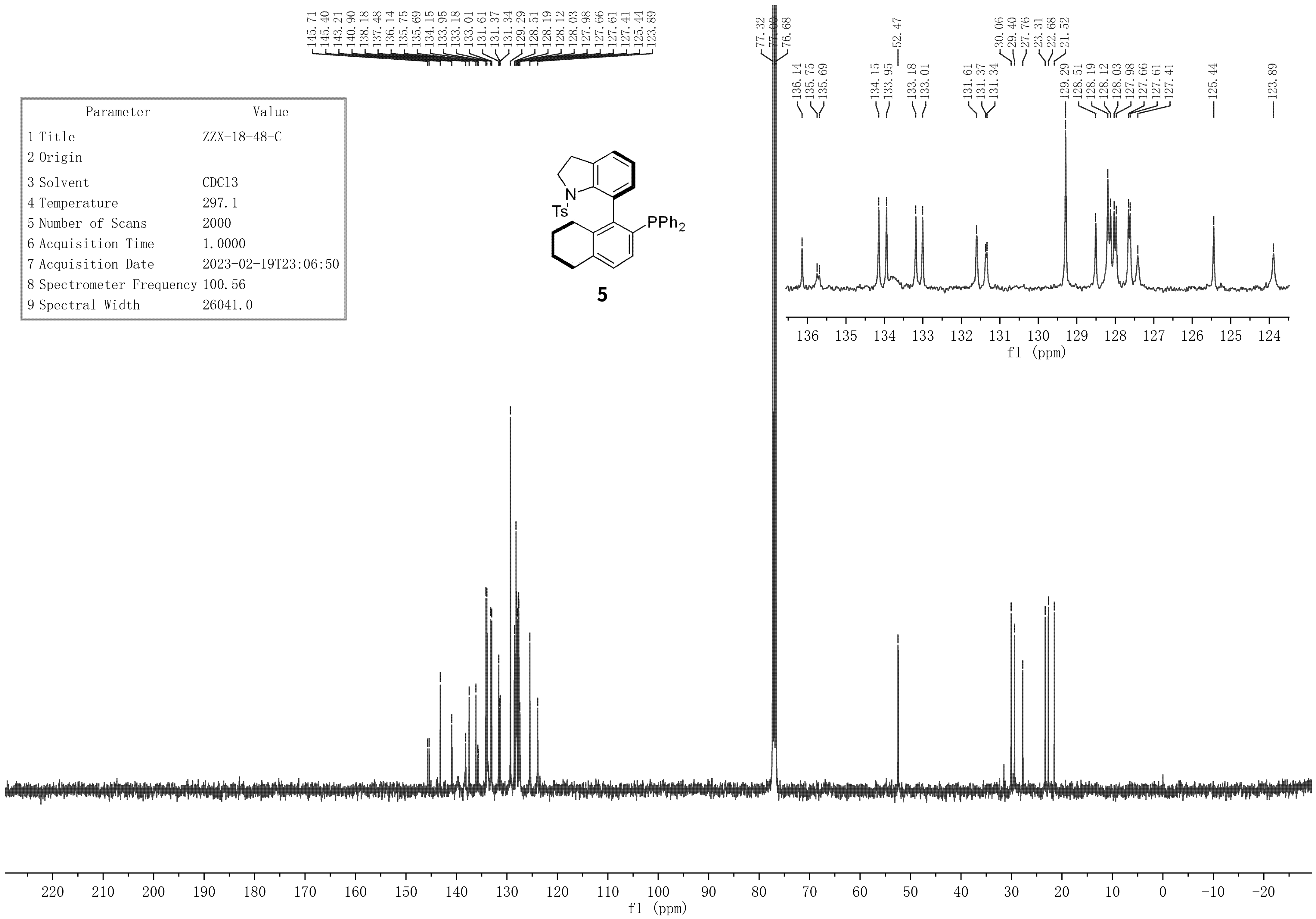
145.71
145.40
143.21
140.90
138.18
137.48
136.14
135.75
135.69
134.15
133.95
133.18
133.01
131.61
131.37
131.34
129.29
128.51
128.19
128.12
128.03
127.98
127.66
127.61
127.41
125.44
123.89
77.32
77.00
76.68
52.47
30.06
29.40
27.76
23.31
22.68
21.52
Parameter Value
1 Title ZZX-18-48-C
2 Origin
3 Solvent CDCl3
4 Temperature 297.1
5 Number of Scans 2000
6 Acquisition Time 1.0000
7 Acquisition Date 2023-02-19T23:06:50
8 Spectrometer Frequency 100.56
9 Spectral Width 26041.0
N
Ts
PPh2
5
f1 (ppm)
f1 (ppm)

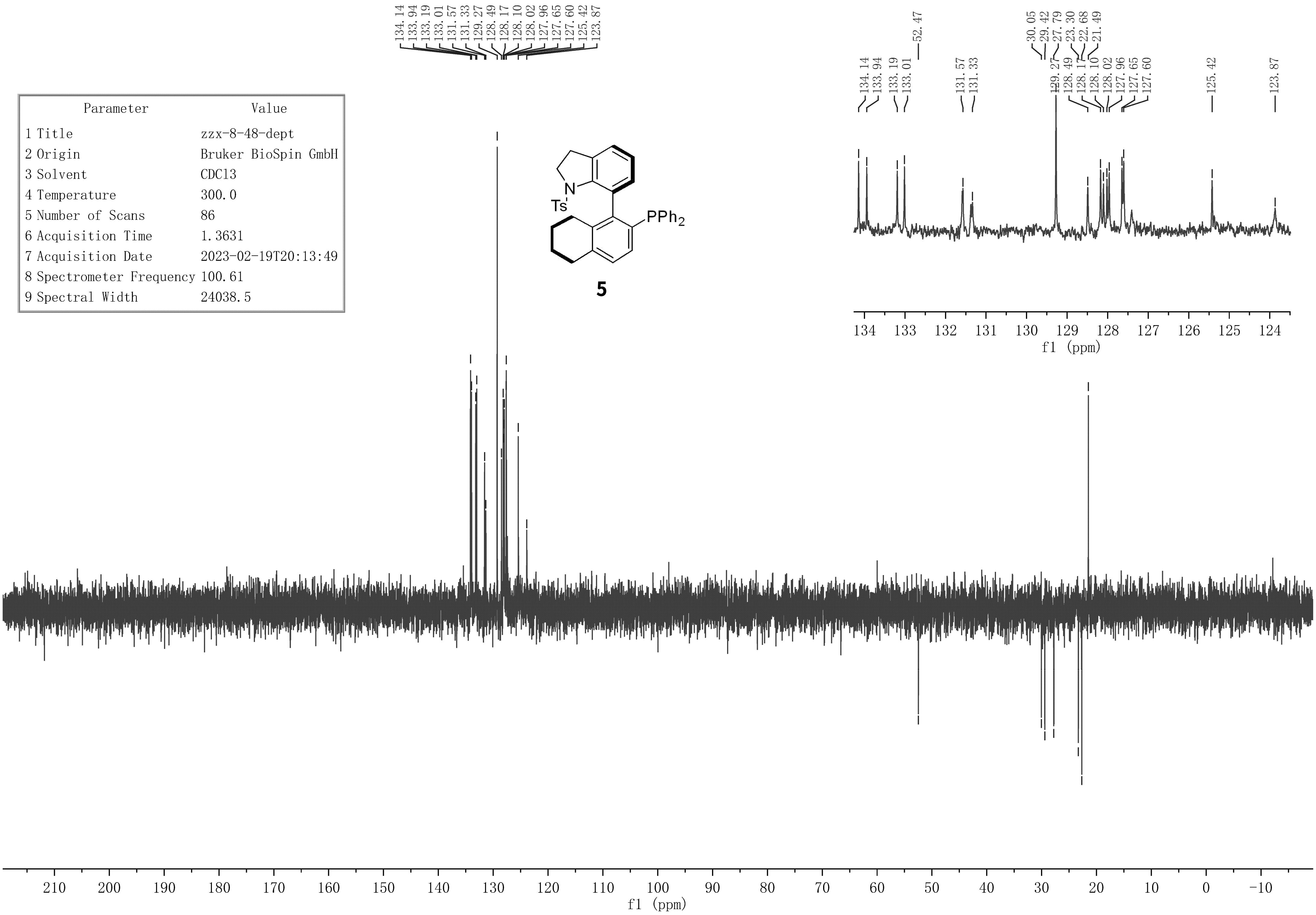

Parameter	Value
1 Title	zzx-8-48-dept
2 Origin	Bruker BioSpin GmbH
3 Solvent	CDCl3
4 Temperature	300.0
5 Number of Scans	86
6 Acquisition Time	1.3631
7 Acquisition Date	2023-02-19T20:13:49
8 Spectrometer Frequency	100.61
9 Spectral Width	24038.5

Parameter	Value
1 Title	zzx-15-239
2 Origin	
3 Solvent	CDCl3
4 Temperature	297.6
5 Number of Scans	32
6 Acquisition Time	1.0000
7 Acquisition Date	2022-04-21T01:09:13
8 Spectrometer Frequency	161.89
9 Spectral Width	66371.0

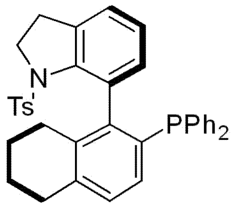

5

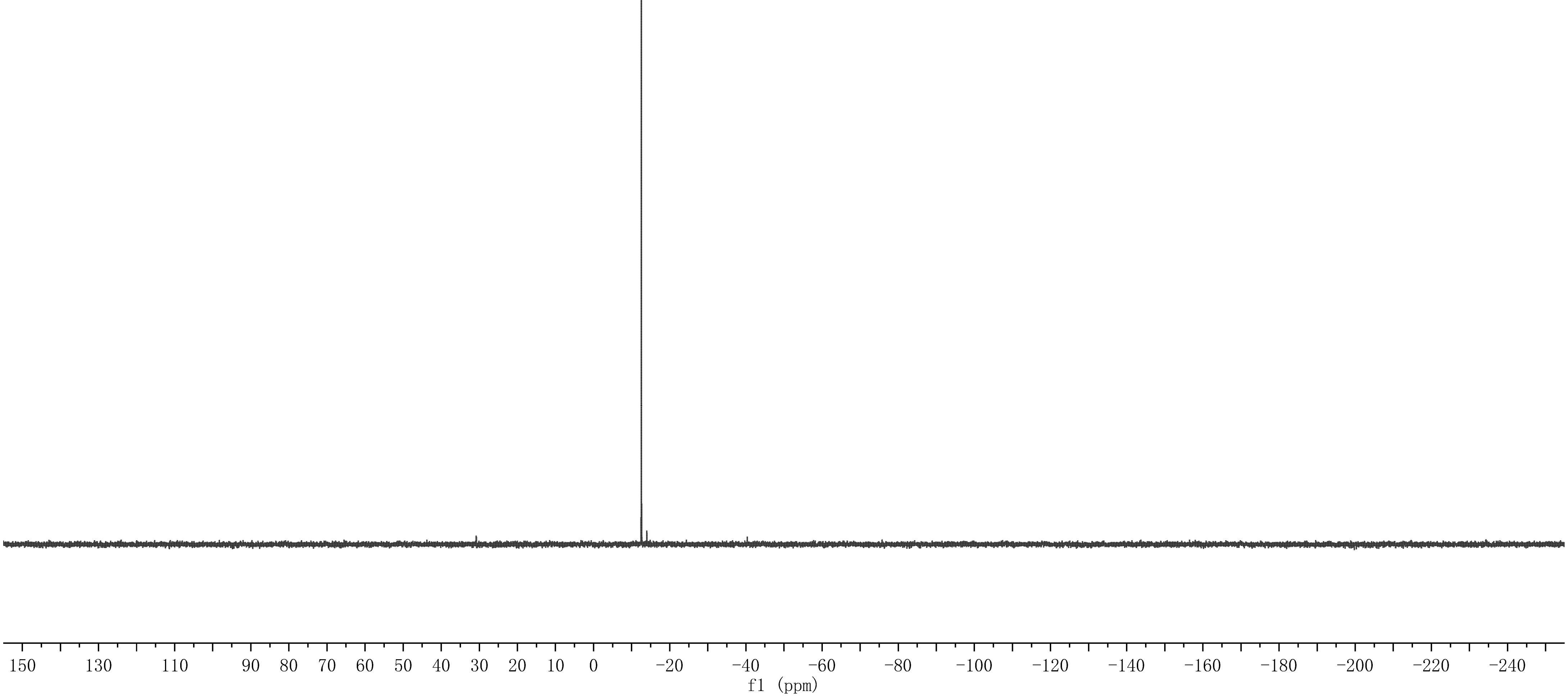

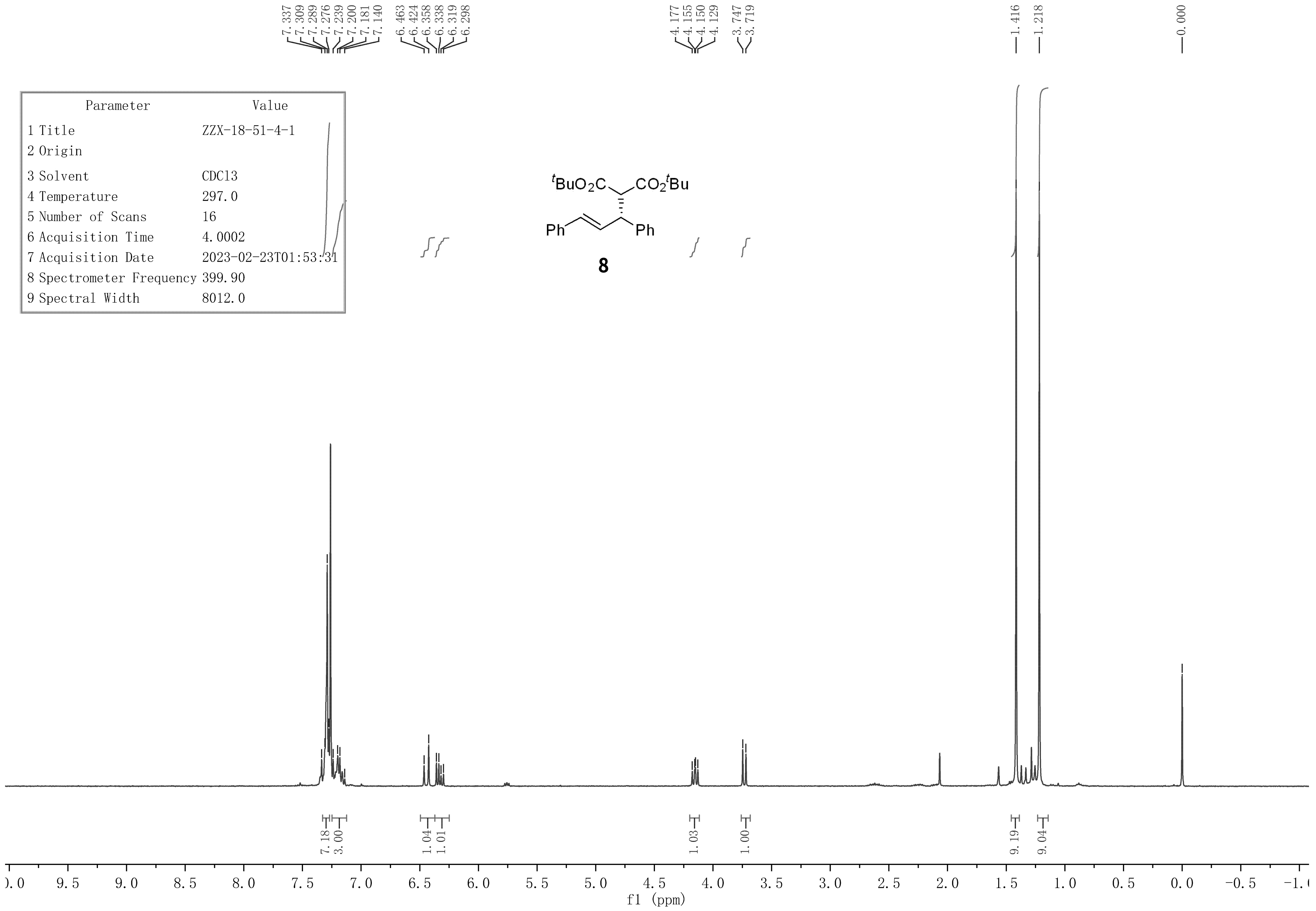

7.337
7.309
7.289
7.276
7.239
7.200
7.181
7.140
6.463
6.424
6.358
6.338
6.319
6.298
4.177
4.155
4.150
4.129
3.747
3.719
1.416
1.218
0.000
Parameter Value
1 Title ZZX-18-51-4-1
2 Origin
3 Solvent CDCl3
4 Temperature 297.0
5 Number of Scans 16
6 Acquisition Time 4.0002
7 Acquisition Date 2023-02-23T01:53:31
8 Spectrometer Frequency 399.90
9 Spectral Width 8012.0
tBuO2C
CO2tBu
Ph
Ph
8
7.18
3.00
1.04
1.01
1.03
1.00
9.19
9.04
9.5
9.0
8.5
8.0
7.5
7.0
6.5
6.0
5.5
5.0
4.5
4.0
3.5
3.0
2.5
2.0
1.5
1.0
0.5
0.0
-0.5
f1 (ppm)

	Parameter	Value
1	Title	ZZX-18-51-4-1
2	Origin	
3	Solvent	CDCl3
4	Temperature	297.0
5	Number of Scans	3000
6	Acquisition Time	1.0000
7	Acquisition Date	2023-02-23T03:36:25
8	Spectrometer Frequency	100.56
9	Spectral Width	26041.0

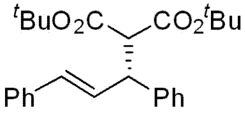

8

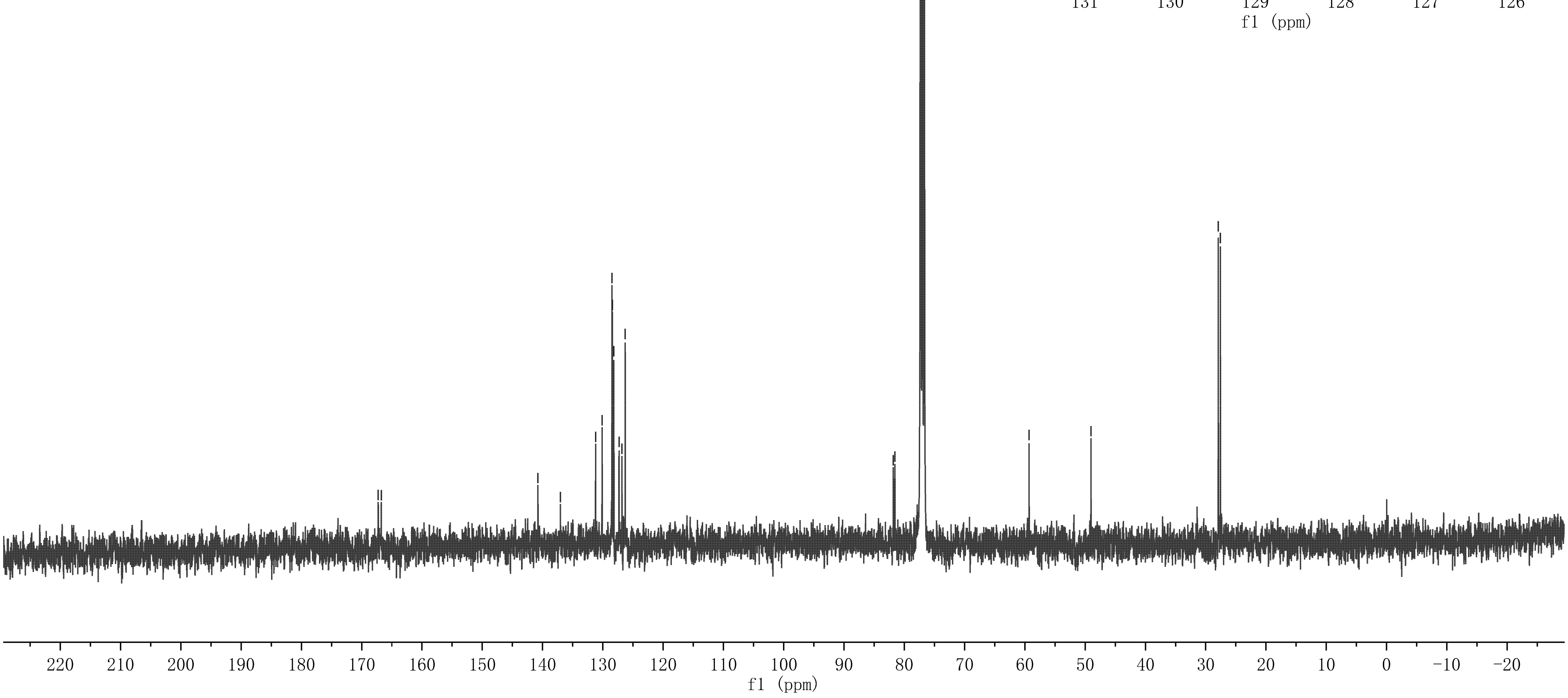

Supplement: SC-014-D3SC01880F-s001 [file SC-014-D3SC01880F-s001.pdf]
